# Programmable synthesis of difluorinated hydrocarbons from alkenes through a photocatalytic linchpin strategy†

**DOI:** 10.1039/d3sc03951j

**Published:** 2023-10-11

**Authors:** Zhi-Qi Zhang, Cheng-Qiang Wang, Long-Ji Li, Jared L. Piper, Zhi-Hui Peng, Jun-An Ma, Fa-Guang Zhang, Jie Wu

**Affiliations:** a Joint School of National University of Singapore and Tianjin University, International Campus of Tianjin University Binhai New City Fuzhou 350207 P. R. China majun_an68@tju.edu.cn; b Department of Chemistry, Tianjin Key Laboratory of Molecular Optoelectronic Sciences, Frontiers Science Center for Synthetic Biology (Ministry of Education), Tianjin University Tianjin 300072 P. R. China zhangfg1987@tju.edu.cn; c Department of Chemistry, National University of Singapore 3 Science Drive 3 Singapore 117543 Republic of Singapore chmjie@nus.edu.sg; d Pfizer Worldwide Research and Development Medicine Eastern Point Rd, Groton CT 06340 USA Zhihui.Peng@pfizer.com

## Abstract

The introduction of difluoromethylene moieties into organic molecules has garnered significant attention due to their profound influence on the physicochemical and biological properties of compounds. Nonetheless, the existing approaches for accessing difluoroalkanes from readily available feedstock chemicals remain limited. In this study, we present an efficient and modular protocol for the synthesis of difluorinated compounds from alkenes, employing the readily accessible reagent, ClCF_2_SO_2_Na, as a versatile “difluoromethylene” linchpin. By means of an organophotoredox-catalysed hydrochlorodifluoromethylation of alkenes, followed by a ligated boryl radical-facilitated halogen atom transfer (XAT) process, we have successfully obtained various difluorinated compounds, including *gem*-difluoroalkanes, *gem*-difluoroalkenes, difluoromethyl alkanes, and difluoromethyl alkenes, with satisfactory yields. The practical utility of this linchpin strategy has been demonstrated through the successful preparation of CF_2_-linked derivatives of complex drugs and natural products. This method opens up new avenues for the synthesis of structurally diverse difluorinated hydrocarbons and highlights the utility of ligated boryl radicals in organofluorine chemistry.

## Abstract 2

We have devised a general and versatile approach for the synthesis of diverse difluorinated alkanes and alkenes by utilizing readily accessible ClCF_2_SO_2_Na as a practical difluoromethylene linchpin.

## Graphical abstract

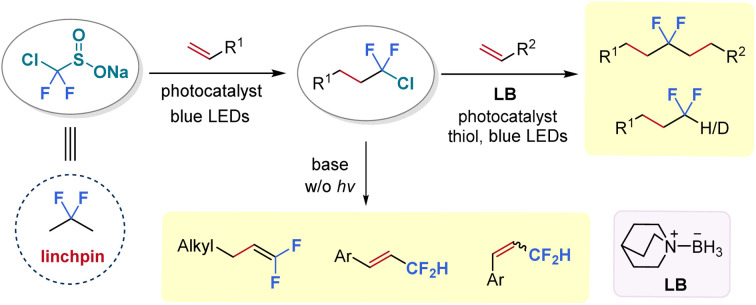

## Introduction

The incorporation of difluoroalkyl groups into organic molecules has garnered significant and sustained attention due to their impact on the physicochemical and biological properties of these molecules.^1–6^ To date, most efforts to construct difluorinated scaffolds rely on deoxyfluorination of carbonyl compounds^7–9^ or transition metal or Lewis acid-catalysed/mediated difluoroalkylation and difluorination reactions,^10–13^ with a particular focus on aryl-CF_2_ systems. However, synthetic approaches to access *gem*-difluoroalkanes {C(sp^3^)–CF_2_–C(sp^3^)} from abundant feedstock chemicals remain scarce.^14–20^ Although a few examples have been reported to form aliphatic difluorinated moieties from unactivated alkenes, these approaches have been limited to using alkyl CF_2_–Br/I as the starting CF_2_ precursors.^21,22^ Thus, there is a pressing need for novel, efficient, and robust methods to expand the diversity of *gem*-difluorinated compounds, particularly those derived from unactivated aliphatic systems.

**Fig. 1:**
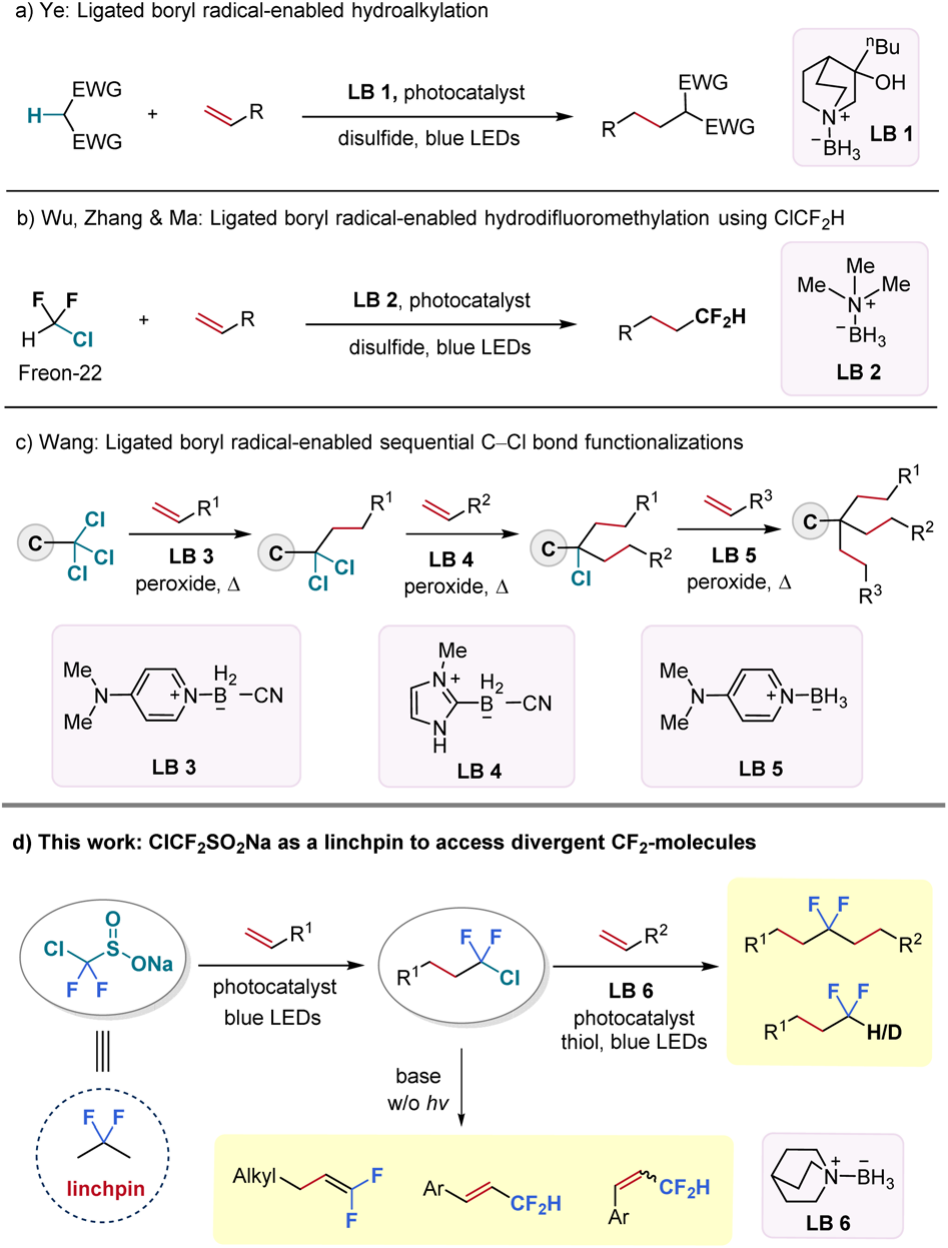
Ligated borane-enabled HAT and XAT processes.

Ligated boryl radicals, denoted by the general formula L^+^-R_2_B˙^−^, are stabilized by coordination to amines, phosphines, sulphides, or *N*-heterocyclic carbenes (NHCs) and share seven electrons in their valence shell.^23–25^ Notably, ligated boryl radicals exhibit a strong nucleophilic character and offer considerable potential for halogen atom transfer (XAT) and hydrogen atom transfer (HAT) processes by tuning the Lewis base motifs.^26–30^ For instance, the Ye group successfully activated electron-deficient C–H bonds by employing a quinuclidine-borane (LB 1) as the HAT reagent to realize a radical hydroalkylation reaction of unactivated alkenes in 2021 (Fig. 1a).^31^ However, the XAT process triggered by ligated-boryl radicals was only known to activate alkyl iodides and bromides for decades,^32–36^ until recently, when our group successfully activated the C–Cl bond (BDE_cal._ = 87 kcal mol^−1^) from chlorodifluoromethane (ClCF_2_H, Freon-22) under blue light irradiation, using commercially available and inexpensive trimethylamine-borane (LB 2) as the XAT reagent (Fig. 1b).^37^ At the same time, the Wang group reported a three-step process for sequential C–Cl bond functionalization of activated trichloromethyl groups with the choice of an appropriate ligated borane reagent in each step (LB 3–5) (Fig. 1c).^38^

Encouraged by these recent scientific advancements, we aimed to establish a protocol for ligated boryl radical-promoted C–Cl bond functionalization of C(sp^3^)–CF_2_–Cl substrates to provide a novel and streamlined method for accessing *gem*-difluorinated aliphatic backbones, which were previously challenging to obtain. Along this endeavour, we turned our attention to an easily accessible reagent—sodium chlorodifluoromethylsulfinate (ClCF_2_SO_2_Na) as the CF_2_-precursor.^39–41^ The proposed sequence involves two radical carbon–carbon bond formation transformations and has the potential to connect two alkene fragments through the valuable CF_2_-unit (Fig. 1d). If successful, this protocol would open an interesting avenue for synthesizing structurally divergent difluorinated hydrocarbons, with ClCF_2_SO_2_Na serving as a practical “linchpin”.^42–48^ While the “linchpin” strategy has been explored in recent years to link two molecular skeletons through linkers such as alkyne, alkene, and sulfone groups, its application in organofluorine chemistry remains underdeveloped.^49–51^ Herein, we collaborated with colleagues from Pfizer to prepare ClCF_2_SO_2_Na in kilogram-scale. By merging radical chlorodifluoromethylation of alkenes with a subsequent ligated boryl radical-facilitated hydrodifluoroalkylation of different alkenes, we were able to easily access a wide variety of internal *gem*-difluoro alkanes under mild visible-light irradiation conditions. Furthermore, photo-induced hydrodechlorination and base-promoted elimination reactions enabled the synthesis of a broad range of terminal *gem*-difluorinated alkanes and alkenes (Fig. 1d).

## Results and discussion

### Development of hydrochlorodifluoromethylation of alkenes

**Fig. 2:**
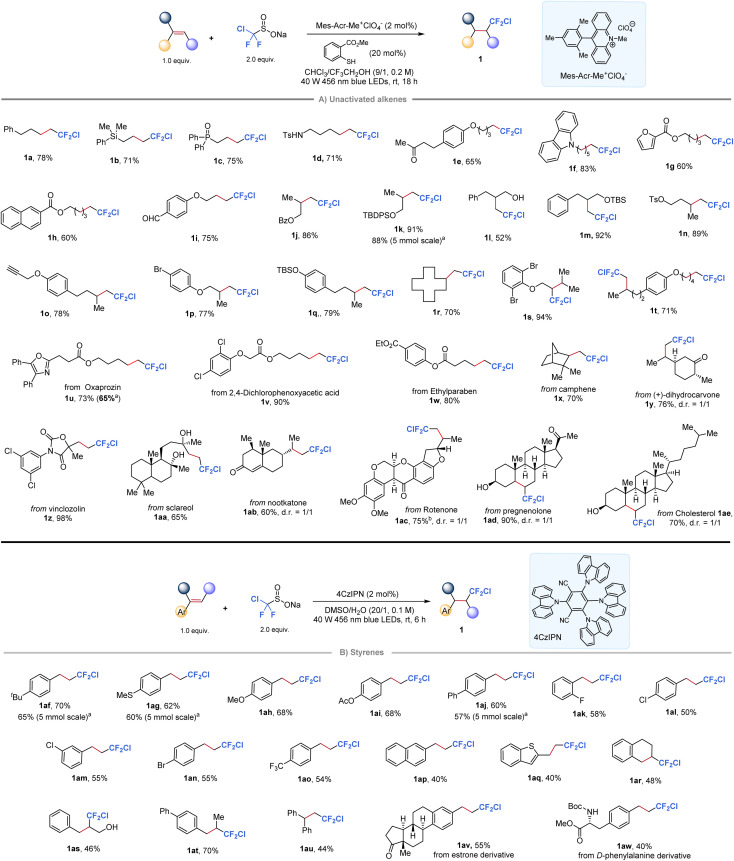
Hydrochlorodifluoromethylation of alkenes with ClCF_2_SO_2_Na. ^a^5.0 mmol of alkenes, 3.0 equiv. of ClCF_2_SO_2_Na; ^b^3.0 equiv. of ClCF_2_SO_2_Na.

Our investigation began with the development of a radical chlorodifluoromethylation protocol for alkenes with ClCF_2_SO_2_Na. A screening of photocatalysts, thiols, and solvents established that the desired product 1j was obtained in up to 86% isolated yield by conducting the reaction with Mes-Acr-Me^+^ClO_4_^−^ as the photocatalyst and methyl thiosalicylate as the HAT mediator in a mixed solvent of CHCl_3_/CF_3_CH_2_OH (9/1, 0.2 M, see the ESI Table 1† for optimization details). As shown in Fig. 2A, this protocol accommodated a wide range of unactivated mono-, di-, and trisubstituted alkenes (1a–1s) with good to high yields. Notably, the reaction conditions tolerated various functional groups, including those bearing silicon (1b), phosphine (1c), amine (1d), silyl ether (1k and 1m), free hydroxyl group (1l), aldehyde (1i), tosylate (1n), hetero/aliphatic-cyclic rings (1f, 1g, and 1r), alkyne (1k), and bromide (1p). Additionally, a substrate bearing two olefin motifs was fully chlorodifluoromethylated at both sites in a 71% yield (1t). Notably, selected complex alkenes derived from natural products or drug molecules were found to be well-tolerated under standard conditions, affording the corresponding difluorinated derivatives (1u–1ae) in good yields.

When the scope of this protocol was extended to styrenes, however, low yields were observed for the target chlorodifluoromethylation products due to undesired polymerization side reactions in the presence of the acridinium photocatalyst. Notably, cyanoarenes have been previously shown to serve as effective organophotocatalysts for the hydrofluoroalkylation of styrenes, with the solvent choice playing a critical role.^52^ Taking this into account, we investigated various organic photocatalysts and solvents, ultimately finding that the desired reaction could be achieved using 1,2,3,5-tetrakis(carbazol-9-yl)-4,6-dicyanobenzene (4CzIPN) in DMSO (Fig. 2B, see the ESI Table 2† for screening details). The protocol was shown to be effective for a range of styrenes substituted at *ortho*-, *meta*-, or *para*-positions of the phenyl ring, yielding the desired products in moderate to good yields (1af–1ao). Furthermore, the protocol successfully delivered chlorodifluoromethylation products from substrates including 2-naphthyl (1ap), heteroaromatic (1aq), cyclic internal alkene (1ar), 1,2-disubstituted (1ar–1at) and 1,1-disubstituted (1au) styrene derivatives. Importantly, the reaction could be easily scaled up to gram-scale with minimal loss (1k, 1af, 1ag, and 1aj), highlighting the practical synthetic utility of this protocol. In addition, relatively complex styrenes derived from estrone or phenylalanine are also accommodated in this reaction to give 1av and 1aw in moderate yields.

### Synthesis of *gem*-difluorinated alkanes from alkenes

**Fig. 3:**
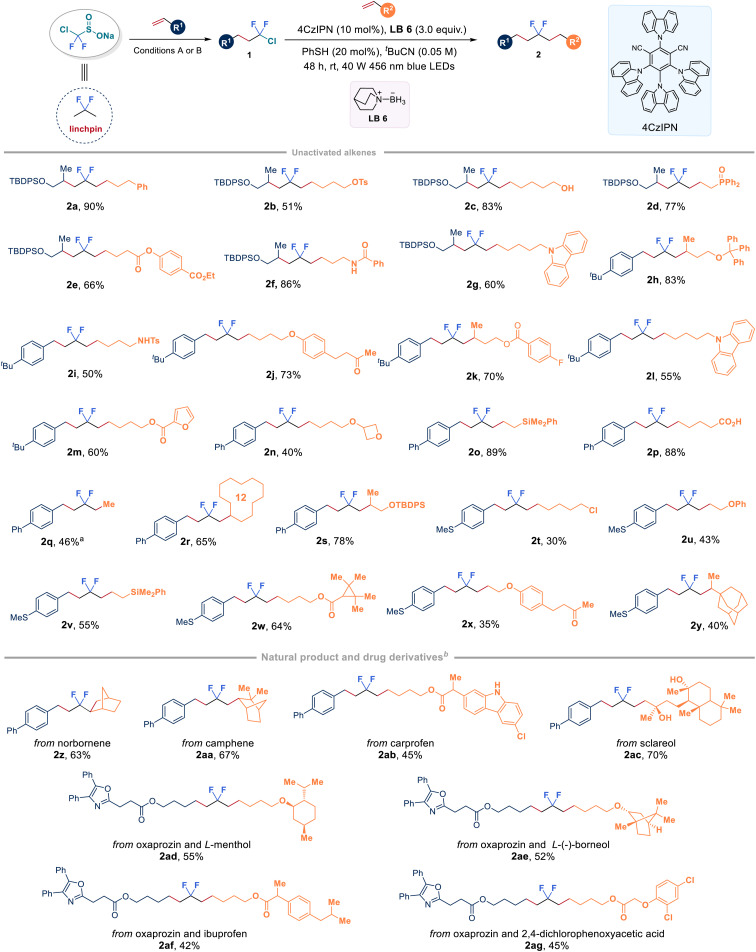
Synthesis of internal *gem*-difluoro alkanes from two alkenes and ClCF_2_SO_2_Na. ^a^1aj (0.2 mmol, 1.0 equiv.), 1 atm balloon of ethylene. ^b^50 equiv. of H_2_O was added.

With a series of chlorodifluoromethyl alkanes in hand, our study then focused on the selective activation of the C–Cl bond by choosing compound 1k as the model substrate with unactivated alkenes. A systematic optimization of the reaction parameters, including photocatalysts, ligated boranes (XAT reagents), disulfides (HAT reagents), solvents, and light source, revealed that the desired product 2a could be obtained in 90% yield by utilizing quinuclidine-ligated borane LB 6 in combination with a simple phenyl thiol under blue light irradiation conditions (see the ESI Table 3† for optimization details). Fig. 3 illustrates the assembly of CF_2_-linked aliphatic hydrocarbons through an organophotoredox-catalysed radical chlorodifluoromethylation coupled with ligated boryl radical-enabled hydrodifluoroalkylation, starting from ClCF_2_SO_2_Na. As a proof of concept study, we briefly explored the scope of ClCF_2_–alkanes (1k, 1af, 1ag, and 1aj) to connect with unactivated alkenes. The study found that a wide range of mono-substituted alkenes with various functional groups, such as tosylate (2b), free hydroxyl group (2c), ester (2e and 2k), amide (2f), carbonyl group (2j and 2x), chloride (2t), and free carboxylic acid (2p), were all compatible to deliver the corresponding difluorinated products in moderate to high yields. Phosphorus (2d), nitrogen (2i), oxygen (2u), and silicon (2o and 2v) atom-tethered alkenes were also competent reaction partners. Furthermore, cyclic structures, including carbazole (2g and 2l), furan (2m), oxetane (2n), cyclododecane (2r) and adamantane (2y), underwent the reaction smoothly. It should be noted that the reaction between ethylene, the largest-volume and cost-effective industrial chemical that has an annual production of over 150 million tons, and 1y led to the formation of *gem*-difluoroalkane (2q) with a yield of 46%. Additionally, the linchpin strategy demonstrated effective utilization in the late-stage functionalization of complex alkenes derived from pharmaceutical molecules and natural products. Unactivated alkenes containing the core structures of norborene (2z), camphene (2aa), carprofen (2ab), or sclareol (2ac) smoothly reacted with 1y with good yields. Importantly, it was feasible to use the linchpin strategy to connect two bioactive olefins derived from oxaprozin and *L*-menthol (2ad), or *L*-(−)-borneol (2ae), or ibuprofen (2af) or 2,4-dichlorophenoxyacetic acid (2ag), which clearly indicates the broad applicability of this strategy. Among the tested alkenes, relatively lower yields were observed for substrates containing allylic alcohols or silanes (*i.e.*, 2u, 2v and 2x) due to the simultaneous formation of C=C bond-reduced by-products. Lower conversions accounted for the moderate yields of some substrates (*i.e.*, 2b, 2i and 2y), and dechlorination of intermediate 1 would be the major process after prolonging the reaction time. Alternatively, low solubility of some alkene starting materials or alkyl-CF_2_Cl intermediates in ^*t*^BuCN might result in moderate yields of some products (*i.e.*, 2ab, 2ad–2ag).

**Fig. 4:**
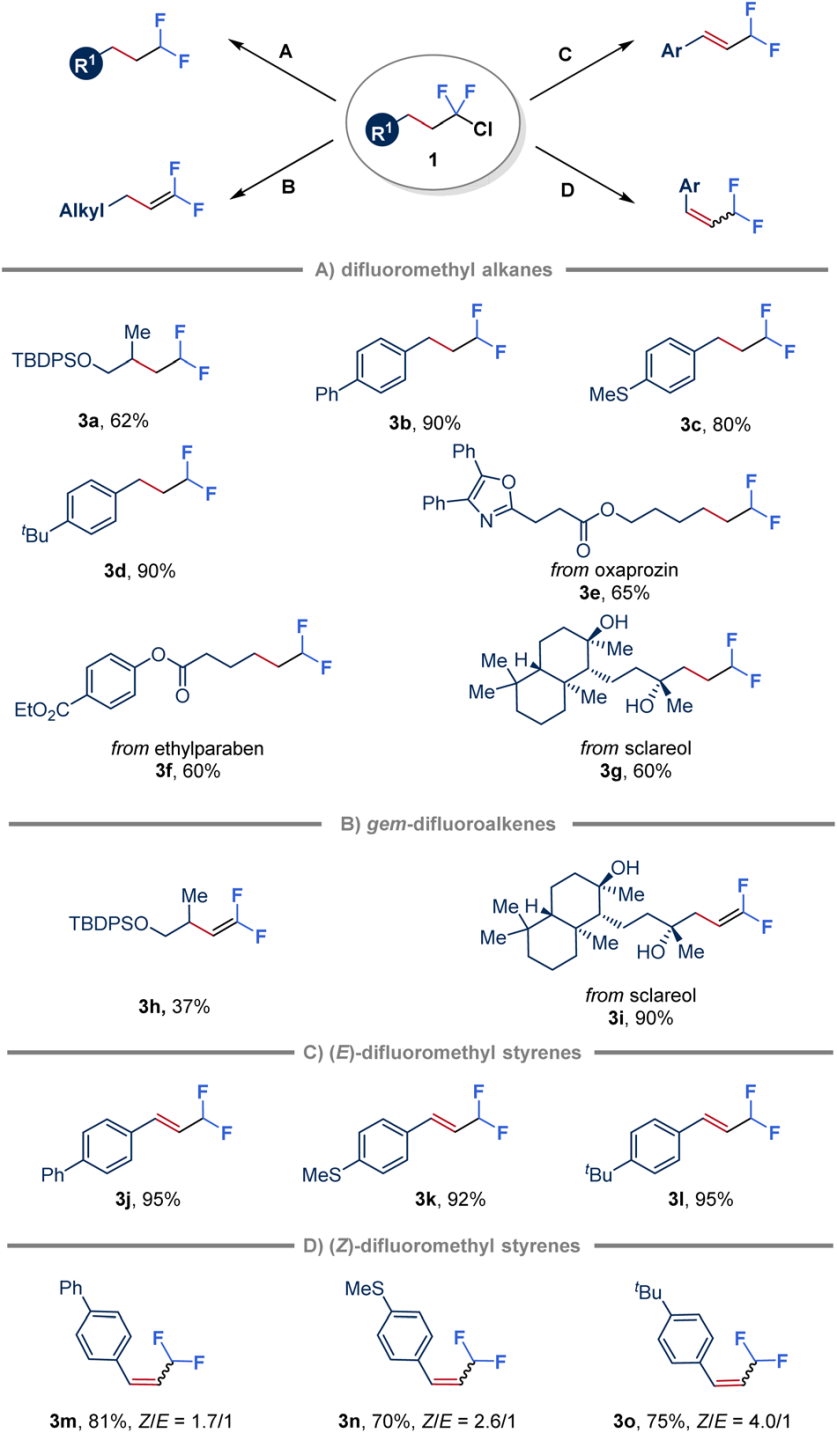
Divergent synthesis of difluorinated hydrocarbons and alkenes. (Condition A) 4CzIPN (10 mol%), LB 6 (1.5 equiv.), (TRIPS)_2_ (10 mol%), ^*t*^BuCN (0.1 M), 40 W 456 nm blue LEDs, rt, 48 h; (Conditions B and C) ^*t*^BuOK (4.0 equiv.), ^*t*^BuCN (0.1 M), rt, 12 h; (Condition D) Ir(ppy)_3_ (1 mol%), ^*t*^BuOK (4.0 equiv.), ^*t*^BuCN (0.1 M), 40 W 456 nm blue LEDs, rt, 12 h.

To further demonstrate the synthetic application of the ClCF_2_–alkane intermediates, various transformations have been explored (Fig. 4). Initially, in the absence of an alkene, hydrodechlorination triggered by quinuclidine-ligated borane (LB 6) was achieved by employing a more sterically hindered hydrogen atom donor, bis(2,4,6-triisopropylphenyl) disulfide (TRIPS)_2_ (Fig. 4A). Substrates derived from both unactivated alkenes and styrenes were found to be suitable for this transformation (3a–3d). Moreover, ClCF_2_–alkanes obtained from natural products, such as oxaprozin, ethylparaben and sclareol, were also efficiently converted to the corresponding products through hydrodechlorination with moderate yields (3e–3g). Furthermore, *gem*-difluoroalkenes (3h and 3i) were obtained in variable yields by simply treating ClCF_2_–alkane 1 with ^*t*^BuOK (Fig. 4B).^53,54^ Notably, ClCF_2_–alkanes derived from styrenes reacted with ^*t*^BuOK to give conjugated (*E*)-β-difluoromethyl styrenes (3j–3l) in high yields attributed to the C=C bond migration to more conjugated systems (Fig. 4C). In addition, inspired by recent advances in *E* to *Z* photoisomerization of alkenes through an energy transfer process,^55^ adding Ir(ppy)_3_ as a triplet photosensitizer in a one-pot two-step manner led to the formation of (*Z*)-β-difluoromethyl styrenes as the major products (3m–3o) with a *Z*/*E* ratio of up to 4 : 1 (Fig. 4D).

### Mechanistic considerations

**Fig. 5:**
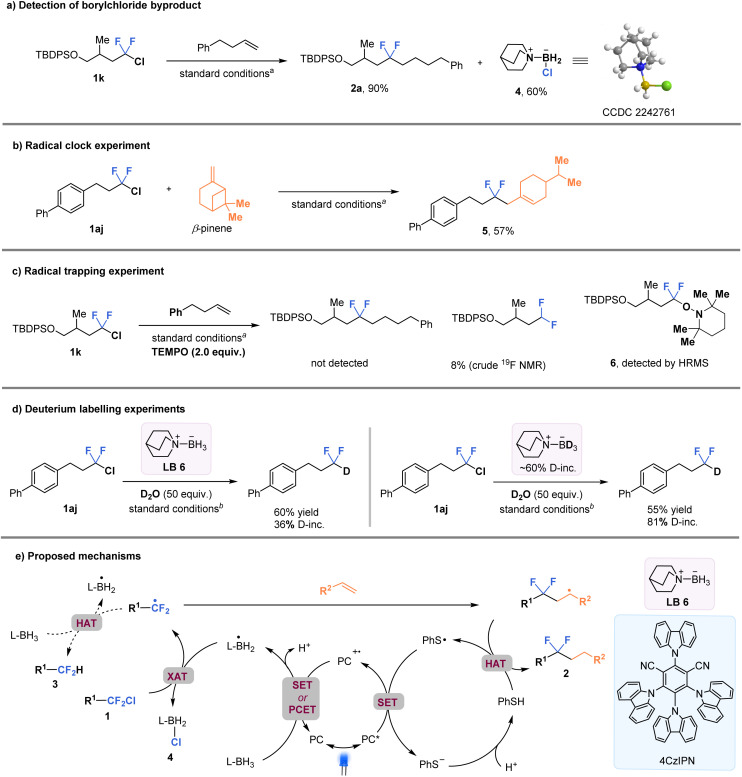
Control experiments for mechanism studies and proposed mechanisms. ^a^Standard conditions in Fig. 3. ^b^Standard conditions in Fig. 4A.

In order to gain a better understanding of the C–Cl bond activation mechanism, a series of experiments were conducted (Fig. 5). First, the isolation of byproduct quinuclidine-BH_2_Cl 4 (confirmed by the X-ray crystallographic analysis) provides support for the proposed key XAT process (Fig. 5a). A radical clock experiment using β-pinene resulted in the formation of the ring-opened product 5 in 57% yield (Fig. 5b). In addition, a radical trapping experiment was also performed using 2,2,6,6-tetramethylpiperidine-1-oxyl (TEMPO), which completely inhibited the formation of the difluoroalkylation product 2a. Instead, the TEMPO-intercepted species 6 was detected by high resolution mass spectrometry (HRMS) (Fig. 5c). These results strongly suggest the involvement of alkyl-CF_2_ radicals in the reaction process. Furthermore, two parallel reactions were conducted using quinuclidine-BH_2_Cl and quinuclidine-BD_2_Cl (about 60% deuterated) with excess deuterium oxide as an additive (Fig. 5d). The results showed that product 3b, with much higher deuterium incorporation, was observed in the latter case (36% *vs.* 81% deuterium incorporation, respectively). This finding suggests that the hydrogen atom of the CF_2_H-product was most likely derived from quinuclidine-BH_3_ through a HAT process. In addition, the very low value of quantum yield (*Φ* = 0.0013) supports the possibility of an in-cage mechanism (See the ESI† for details). Stern–Volmer quenching experiments indicated that the excited photocatalyst could be quenched more effectively by PhSSPh than by ligated borane or the alkyl-CF_2_Cl intermediate (See the ESI† for details).

A tentative mechanism is proposed based on all the experimental data and previous literature reports^31,37,38^ (Fig. 5e). The excited-state photocatalyst [4CzIPN]* [*E*_1/2_ (PC˙^+^/PC*) = −1.18 V *vs.* SCE in MeCN] is presumed to reduce the aryl thiol radical to thiol anion species [E(PhS˙/PhS^−^) = 0.16 V *vs.* SCE]. Then PC˙^+^ would trigger the formation of the key ligated boryl radical intermediate (L-BH_2_˙) through either a single electron transfer (SET)/deprotonation sequence or a concerted proton-coupled electron transfer (PCET) process. Subsequently, the nucleophilic boryl radical activates the C–Cl bond of substrate 1 through a XAT process, resulting in the formation of the corresponding CF_2_-alkyl radical (R^1^-CF_2_˙). The R^1^-CF_2_˙ undergoes intermolecular radical addition with the alkene substrate, followed by a HAT process with thiophenol, to generate the desired *gem*-difluoroalkane 2. Alternatively, the CF_2_-alkyl radical may undergo HAT with amine-borane, leading to the formation of difluoromethyl alkane 3 in the absence of alkenes.

## Conclusions

In summary, we have successfully developed a general and widely applicable method for synthesizing diverse difluorinated alkanes and alkenes by leveraging easily accessible ClCF_2_SO_2_Na as a practical difluoromethylene linchpin. This strategy involves an organophotoredox-catalysed hydrochlorodifluoromethylation reaction, followed by a tertiary amine-borane-triggered XAT process under blue light irradiation conditions. Our approach offers several advantages, including broad substrate scope, excellent functional group tolerance, metal-free character, mild reaction conditions, and CF_2_-link-derivatization of complex bioactive alkenes, which demonstrate the potential utilities of this linchpin protocol. Our ongoing research involves merging ligated boryl radicals with transition metal catalysis and exploring its potential applications.

## Data availability

The ESI† contains method description, product characterization data, NMR spectra, and mechanism study details.

## Author contributions

Z. Q. Z., J. A. M., F. G. Z. and J. W. conceived and designed the investigations. Z. Q. Z., C. Q. W. and L. J. L. performed the experiments. J. L. P. and Z. H. P. gave the guidance. Z. Q. Z., J. A. M., F. G. Z. and J. W. wrote the manuscript.

## Conflicts of interest

There are no conflicts to declare.

## Supplementary Material

SC-014-D3SC03951J-s001

SC-014-D3SC03951J-s002
