# Supplementary material for: Programmable synthesis of difluorinated hydrocarbons from alkenes through a photocatalytic linchpin strategy
Source: Chem Sci. 2023 Oct 11;14(41):11546–53. doi: 10.1039/d3sc03951j (PMC10599468; doi:10.1039/d3sc03951j)

Electronic Supplementary Material (ESI) for Chemical Science.
This journal is © The Royal Society of Chemistry 2023

Supplementary Information

Programmable synthesis of difluorinated hydrocarbons from alkenes through a photocatalytic linchpin strategy

Zhi-Qi Zhang,[1,2,3] Cheng-Qiang Wang,[3] Long-Ji Li,[1,3] Jared L. Piper,[4] Zhi-Hui Peng,[4]* Jun-An Ma,[1,2]* Fa-Guang Zhang,[2]* & Jie Wu[3]*

[1]Joint School of National University of Singapore and Tianjin University, International Campus of Tianjin University, Binhai New City, Fuzhou 350207, P. R. of China
[2]Department of Chemistry, Tianjin Key Laboratory of Molecular Optoelectronic Sciences, Frontiers Science Center for Synthetic Biology (Ministry of Education), Tianjin University, Tianjin 300072, P. R. of China
[3]Department of Chemistry, National University of Singapore, 3 Science Drive 3, Singapore 117543, Republic of Singapore
[4]Pfizer Worldwide Research and Development Medicine, Eastern Point Rd, Groton, CT 06340, USA

Corresponding Authors
Jie Wu–chmjie@nus.edu.sg, Fa-Guang Zhang–zhangfg1987@tju.edu.cn, Jun-An Ma–majun_an68@tju.edu.cn, Email: Zhihui.Peng@pfizer.com

Table of contents

I. General information 3
II. General procedures 3
III. Preparation of $ClCF_2SO_2Na$ in kilogram scale 6
IV. Evaluation of reaction conditions 8
V. Unsuccessful examples of chlorodifluoromathylation 11
VI. Mechanistic investigation 12
- a)Identification of the reaction mass balance 12
- b) Radical trapping experiments 12
- c) Radical clock experiments 13
- d) Deuterium labelling study 13
- e) Stern-volmer fluorescence quenching studies 14
- f) Determination of photochemical quantum yields 16

VII. X-Ray crystallographic data 18
VIII. Product characterizations 27
IX. Supplementary references 64
X. NMR spectra for product characterization 65

I. General information

Chemicals and solvents were purchased from commercial suppliers (Sigma Aldrich, TCI, BLD or Oakwood) and used as received. Commercial unavailable unactivated alkenes and 4CzIPN were synthesized according to literatures.[1-3] Kessil PR160 blue LED lamps (456 nm, 40 W) were used as the light source. The purification of the products was performed by flash column chromatography using silica gel 60 (63-200 µm) from SANPONT. ^{1}H NMR, ^{19}F NMR, ^{11}B NMR, ^{31}P NMR, ^{13}C NMR spectra were recorded on a Bruker AV-III400 (400 MHZ) or AMX500 (500 MHz) spectrometer and no reference was used in ^{19}F NMR, ^{11}B NMR, ^{31}P NMR. Chemical shifts were calibrated using residual undeuterated solvent as an internal reference ($CDCl_3$: 7.26 ppm ^{1}H NMR, 77.16 ppm ^{13}C NMR). Multiplicity was indicated as follows: s (singlet), d (doublet), t (triplet), q (quartet), m (multiplet), dd (doublet of doublet), dt (doublet of triplet), td (triplet of doublet), tt (triplet of triplet), ddd (doublet of doublet of doublet), dddd (doublet of doublet of doublet of doublet). High-resolution mass spectra (HRMS) were obtained on a Finnigan/MAT 95XL-T spectrometer.

II. General procedures

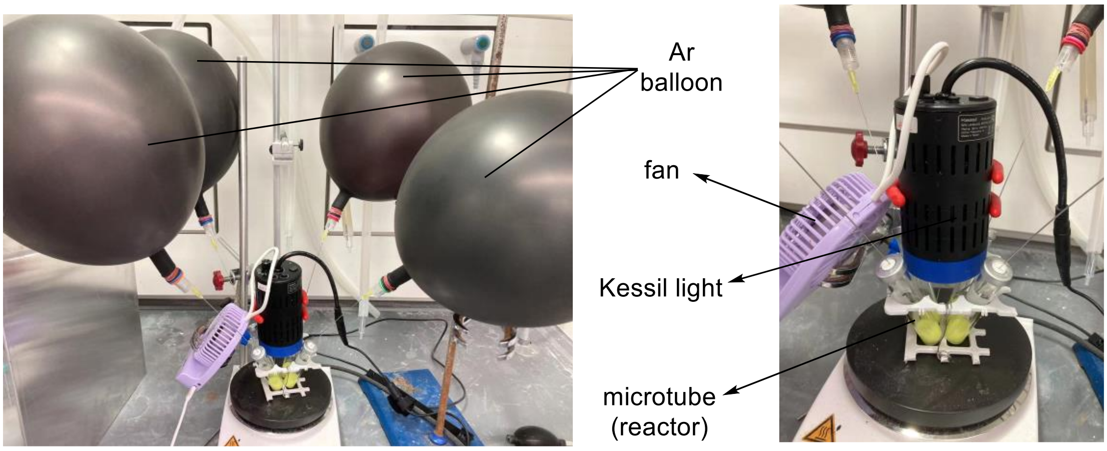

Supplementary Fig. 1. Reaction set-up.

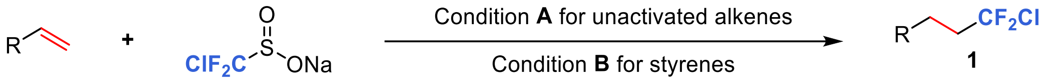

General procedure A

At argon atmosphere, an 8 mL vial equipped with a stir bar was added Mes-Acr-$Me^{+}ClO_4^{-}$ (1.6 mg, 2 mol%), $ClCF_2SO_2Na$ (68.8 mg, 2.0 equiv.), 2-CO_2Me-PhSH (6.7 mg, 20 mol%), alkenes (0.2 mmol), followed by mixture solvent ($CHCl_3$/TFE = 9/1, 1.0 mL) for a reaction concentration of 0.2 M relative to alkenes. Then the vial was sealed with an argon balloon. The resulting mixture was stirred and irradiated with 40 W 456 nm blue LEDs for 18 hours (with a fan, reaction temperature approximately 25 - 30 ^{o}C). After that, the reaction mixture was concentrated under reduced pressure, purified by column chromatography over silica gel to obtain the desired chlorodifluoromethylation products.

General procedure B

At argon atmosphere, an 8 mL vial equipped with a stir bar was added 4CzIPN (3.2 mg, 2 mol%), $ClCF_2SO_2Na$ (68.8 mg, 2.0 equiv.), styrenes (0.2 mmol), followed by mixture solvent (DMSO/H_2O = 20/1, 2.0 mL) for a reaction concentration of 0.1 M relative to styrenes. Then the vial was sealed with an argon balloon. The resulting mixture was stirred and irradiated with 40 W 456 nm blue LED for 6 hours. After that, the reaction mixture was concentrated under reduced pressure, purified by column chromatography over silica gel to obtain the desired chlorodifluoromethylation products.

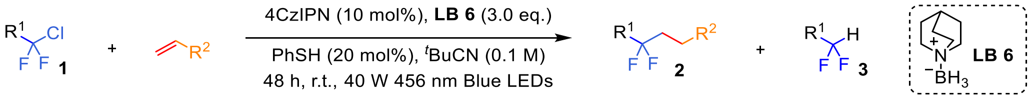

General procedure C

An 8 mL vial equipped with stir bar was added 4CzIPN (8.0 mg, 10 mol%), **LB 6** (37.5 mg, 3 equiv.), compound **1** (0.2 mmol, 2.0 equiv.), followed by ^{t}BuCN (2.0 mL) for a reaction concentration of 0.05 M relative to alkenes. Then the vial was sealed, and the mixture was bubbled with an argon balloon for about 5 mins at room temperature. Next, alkenes (0.1 mmol) and PhSH (20 mol%) were added. The resulting mixture was stirred and irradiated with blue LEDs (40 W, 456 nm) for 48 hours. After that, the reaction mixture was concentrated under reduced pressure, purified by column chromatography over silica gel to obtain the desired hydrodifluoroalkylation products.

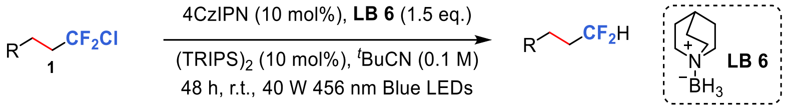

General procedure D

At argon atmosphere, an 8 mL vial equipped with a stir bar was added 4CzIPN (8 mg, 10 mol%), compound **1** (0.1 mmol, 1.0 equiv.), $(TRIPS)_2$ (4.7 mg, 10 mol%), **LB 6** (1.5 equiv.), followed by *t*BuCN (1.0 mL). Then the vial was sealed with an argon balloon. The resulting mixture was stirred and irradiated with blue LEDs (40 W, 456 nm) for 6 hours. After that, the reaction mixture was concentrated under reduced pressure, purified by column chromatography over silica gel to obtain the desired dechlorination products.

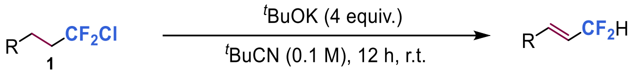

General procedure E

An 8 mL vial equipped with a stir bar was added compound **1** (0.2mmol, 1.0 equiv.), *t*BuOK (89.2 mg, 4 equiv.), followed by *t*BuCN (2.0 mL). The resulting mixture was stirred for 12 hours at rt. After that, the reaction mixture was concentrated under reduced pressure, purified by column chromatography over silica gel to obtain the desired products.

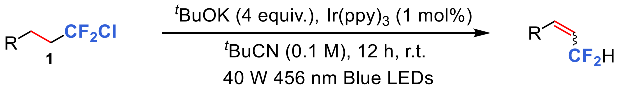

General procedure F

An 8 mL vial equipped with a stir bar was added $Ir(ppy)_3$ (1.3 mg, 1 mol%), compound **1** (0.2 mmol, 1.0 equiv.), *t*BuOK (89.2 mg, 4 equiv.), followed by *t*BuCN (2.0 mL). The resulting mixture was stirred and irradiated with blue LEDs (40 W, 456 nm) for 12 hours. After that, the reaction mixture was concentrated under reduced pressure, purified by column chromatography over silica gel to obtain the desired products.

III. Preparation of $ClCF_2SO_2Na$ in kilogram scale

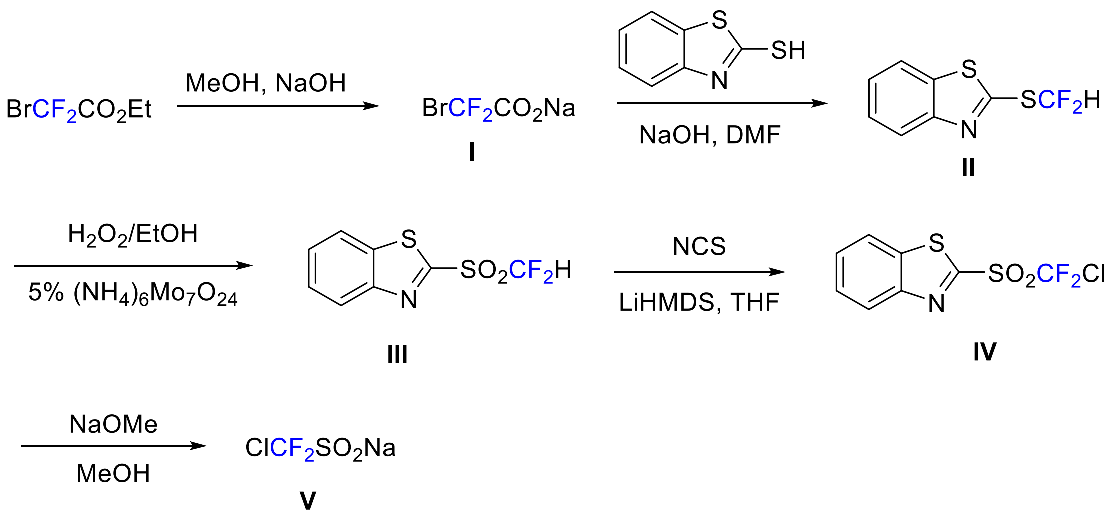

$ClCF_2SO_2Na$ was prepared based on a reported procedure from Hu.[4] Methanol (116 kg) and sodium hydroxide (9.3 kg, 1.0 equiv.) were charged to a reactor (500 L), and the solution was cooled to 0±5 °C. Ethyl bromodifluoroacetate (49 kg, 1.0 equiv.) was added slowly to keep the reaction temperature below 10 °C. After charge, the reaction was allowed to warm to 25±5 °C, and stirred for 3 hrs. The mixture was concentrated under vacuum to obtain a solution of the product in methanol. The solution of the product is used directly for next step. The purity of **I** is 98.8%.

Preparation of $BTSCF_2H$ (**II**): DMF (370 kg), BTSH (39 kg, 1.0 equiv.), and NaOH (9.4 kg, 1.01 equiv.) were added into a reactor and stirred for 20 h at 20-30 °C. The solution of **I** in methanol (assuming quantitative yield in the first step) was added slowly to the reactor. The batch was heated to 40-45 °C and stirred until IPC showed complete conversion. Water (7 volumes) was added into the reactor at 20±5 °C and the mixture was concentrated under reduced pressure to remove DMF and water. MTBE (5 volumes) and water (9 volumes) were added and the layers separated. The aqueous layer was back extracted with MTBE (3 volumes) and the combined organic layer was concentrated under reduced pressure to obtain **II** (46.5 kg, yield 85%, purity 83.4%) as a light brown oil.

Preparation of $BTSO_2CF_2H$ (**III**): EtOH (171 kg), **II** (46.5 kg), and ammonium molybdate tetrahydrate (12.2 kg, 0.05 equiv.) was added to a reactor (500 L) at 20±5 °C. 28% Hydrogen peroxide (105 kg, 4.26 equiv.) was added slowly at 25±5 °C. The reaction was stirred at 25±5 °C for 24 h and at 40-45 °C for 18h. After cooling to 0-5 °C, the reaction was quenched with aqueous sodium sulfite (18 wt%). The resulting mixture was filtered, and washed with water (4 volumes ×3) to get the crude product. The crude product was recrystallized from EtOH (8 wt equiv.) and filtered at 0-5 °C. Later, the product was washed with EtOH (1 volume) and then dried at 45-55 °C

under vacuum oven to obtain **III** as a white solid (32.5 kg; purity 99.64%; yield 66%).

Preparation of $BTSO_2CF_2Cl$ (**IV**): THF (266 kg) was added to a reactor(1000 L) and cooled to -70 ~ -75 ^oC. TEMPO (0.61 kg), **III** (32.5 kg), NCS (52 kg) were added to the reaction. LiHMDS (365.9 kg, 1 M) was added slowly at this temperature. After addition, the mixture was stirred for 2 h at this temperature. Upon completion of the reaction, the reaction mixture was transferred to a mixture of 20% aq. NH_4Cl (10 wt), ethyl acetate and TEMPO to quench the reaction. The layers were separated and the aqueous phase was back extracted with EtOAc once. The combined organic phase was washed with water (6 wt×2), concentrated, and crystallized from EtOH (5.5 wt). The slurry was cooled to 0-5 ^oC, filtered, washed with EtOH (2 wt), and dried at 40-50 °C to obtain **IV** (20.84 kg, purity 99.84%, yield 56%) as a white solid.

Preparation of $ClCF_2SO_2Na$ (**V**): MeOH (49 kg) and **IV** (20.84 kg) were added to a reactor (200 L), and cooled to -10 ~ -5 ^oC. Sodium methoxide (29.3 wt%) in methanol (13.5 kg) was added dropwise at -10 ~ -5 ^oC and reacted at this temperature for 2 h. The mixture was allowed to warm to 20±5 ^oC and stirred at 20 ±5 ^oC for 90 min. The reaction mixture was washed with heptane (3 volumes×3), and treated with activated carbon at 20±5 ^oC for 1h. The filtrate was concentrated and the MeOH solvent was replaced with toluene. MTBE (2 volumes) was added, and the resulting slurry was filtered, washed and dried to obtain **V** (11.42 kg, yield 90%, purity 94.2%) as a white solid.

IV. Evaluation of reaction conditions

Supplementary Table 1: Evaluation of chlorodifluoromathylation with unactivated alkenes

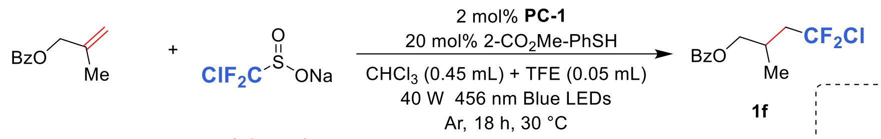

alkene, 0.2 mmol   0.4 mmol

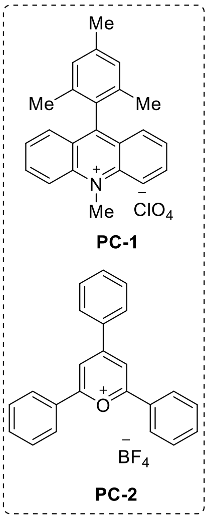

Entry	Deviation from standard conditions	Conversion[a]	Yield[a]
1	None	100%	91%(86%)[b]
2	Rose Bengal instead of **PC-1**	< 5%	trace
3	**PC-2** instead of **PC-1**	< 5%	trace
4	4-CzIPN instead of **PC-1**	100%	82%
5	No **PC-1**	0%	NR
6	In the dark	10%	6%
7	No 2-CO_2Me-PhSH	100%	61%
8	No TFE	76%	60%

[a]Using 1,1,2,2-tetrachloroethane as the internal standard. [b]Isolated yield.

Supplementary Table 2: Evaluation of chlorodifluoromathylation with styrenes

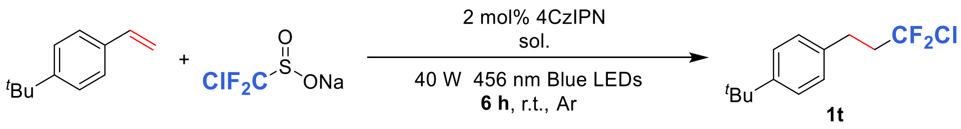

0.2 mmol   0.4 mmol

Entry	Solvent	Conversion[a]	Yield[a]
1[b]	--	100%	n.d.
2	DMSO	40%	20%
3	DMSO/H_2O (20/1)	100%	73% (70%[c])
4	DMSO/H_2O (4/1)	100%	51%
5	MeCN/H_2O (20/1)	100%	15%

[a]Using $PhCF_3$ as the external standard; [b]Condition: Mes-Acr-$Me^+ClO_4^-$ (2 mol%), $CHCl_3$/TFE (9/1, 0.2 M), r. t., 18 h; [c]Isolated yield.

Supplementary Table 3: Evaluation of difluoromathylation with unactivated alkenes

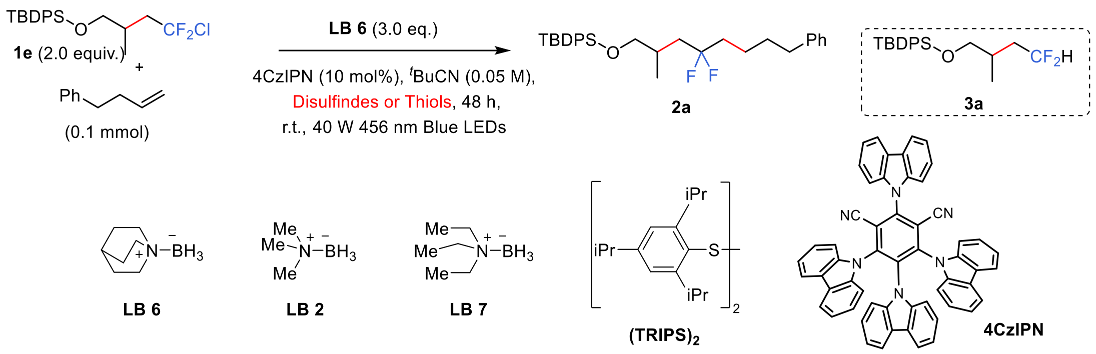

Entry	Disulfides (10 mol%) or Thiols (20 mol%)	Recovery of **1**[a]	Recovery of **alkene**[a]	Yield of **2**[a]	Ratio **(2/3)**[a]
1[c]	PhSSPh	64%	24%	75%	1.25/1
2[d]	PhSSPh	78%	35%	29%	1/1.27
3[e]	PhSSPh	175%	20%	trace	--
4	PhSSPh	36%	9%	86%	1.23/1
5	$(TRIPS)_2$	99%	60%	4%	1/23
6[f]	PhSSPh	95%	17%	70%	2/1
7	PhSH	20%	<1%	95% (90%)[b]	2/1
8	^{t}BuSH	30%	<1%	94%	1.96/1
9	$(^i Pr)_3SiSH$	81%	3%	64%	1.16/1
10	Et_2OCCH_2SH	54%	4%	86%	1.53/1
11	$(3,5\text{-diCF}_3)PhSH$	36%	<1%	86%	2.15/1

[a]Using CH_2Br_2 and $PhCF_3$ as the external standards; [b]Isolated yield; [c]**LB 6** (2.0 equiv.); [d]**LB 2** (2.0 equiv.) instead of **LB 6**; [e]**LB 7** (2.0 equiv.) instead of **LB 6**; [f]Adding H_2O (50 equiv.).

Supplementary Table 4: Evaluation of the dechlorination

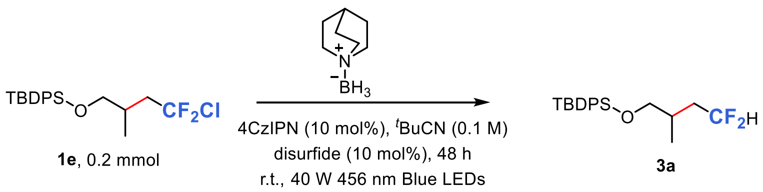

Entry	Disulfides	**L B 4** (equiv.)	Recovery of **L B 4**[a]	Recovery of **1**[a]	Yield of **3**[a]
1	PhSSPh	1.5	0.68 equiv.	14%	44%
2	$(TRIPS)_2$	1.5	0	16%	65% (62%)[b]
3	$(TRIPS)_2$	2	0.2 equiv.	10%	65%
4	$(TRIPS)_2$	3	1.4 equiv.	12%	68%
5	Without	--	0.16 equiv.	15%	35%

[a]Using CH_2Br_2 and $PhCF_3$ as the external standard; [b]Isolated yield.

Supplementary Table 5: Evaluation of dehydrochlorination

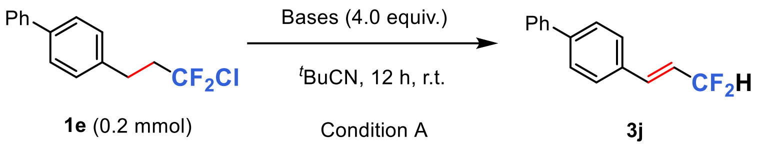

Entry	Bases (4.0 equiv.)	Recovery of **1e**[a]	Yield of **3**[a]
1	^{t}BuOK	0%	98% (95%)[b]
2	other bases[c]	> 90%	0%

[a]Using $PhCF_3$ as the external standard; [b]Isolated yield;
[c]Other bases: NaOH, Na_2CO_3, $NaHCO_3$, K_3PO_4, K_2HPO_4, DBU.

Supplementary Table 6: Evaluation of dehydrochlorination

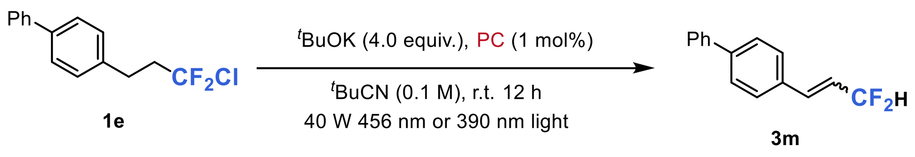

Entry	PC (1 mol%)	EnT (Kcal/mol)	Recovery of **1e**[a]	Yield of **3m** (Z/E)[a]
1	4CzIPN	56.4	0	46% (1.3/1)
2[c]	4CzIPN	56.4	30%	54% (1/99)
3	xanthone	70.0	0	44% (1/2)
4	benzophenone	69.1	0	60% (1/3)
5	fluorenone	50.5	0	55% (1/4.6)
6	benzil	53.3	0	60% (1/9)
7	Riboflavin	50.4	0	61% (1/2.4)
8	$Ir(ppy)_3$	55.2	10%	84% (1.7/1), 81%[b]
9[d]	$Ir(ppy)_3$	55.2	0	65% (2/1)
10	$[Ir(ppy)_2(dtbbpy)]PF_6$	51.0	8%	43% (1.7/1)
11	$[Ir(dFCF_3ppy)_2(bpy)]PF_6$	62.0	0	92% (1/11)

[a]Using $PhCF_3$ as the external standard; [b]Isolated yield; [c]Adding H_2O (50 equiv.); [d]24 h.

V. Unsuccessful examples of chlorodifluoromathylation

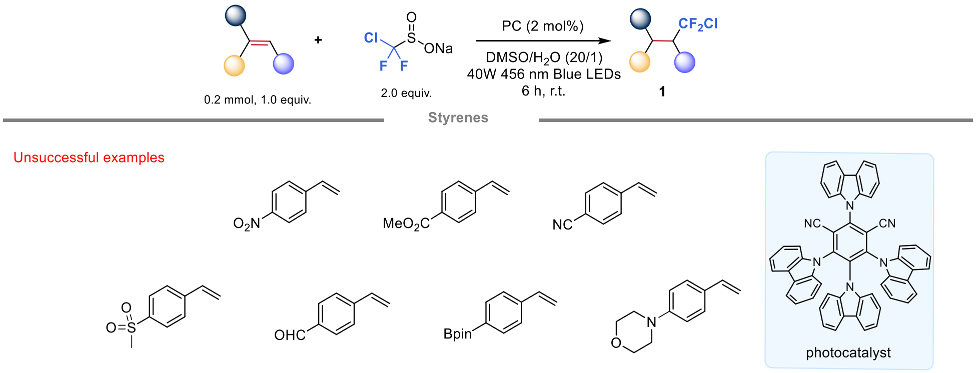

Supplementary Fig. 2: Unsuccessful examples

VI. Mechanistic investigation

a) Identification of the reaction mass balance

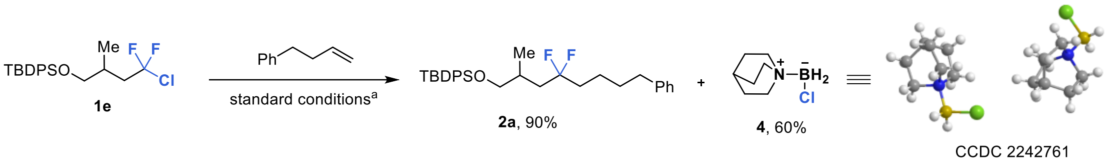

Follow the general procedure C, L-BH_2Cl **4** was isolated by flash column chromatography over silica gel (white solid, 28.6 mg, 60%, m.p. 126.3-127.0 ^{0}C), determined by NMR and single crystal.

Compound **4** (m.p. 126.3-127.6 ^{0}C). **^{1}H NMR** (400 MHz, $CDCl_3$) δ 3.27 – 2.89 (m, 6H), 2.80 – 1.94 (m, 3H), 1.89 – 1.61 (m, 6H). **^{11}B NMR** (128 MHz, $CDCl_3$) δ -1.33 (t, J = 122.2 Hz). **^{13}C NMR** (126 MHz, $CDCl_3$) δ 50.4, 24.4, 20.6.

b) Radical trapping experiments

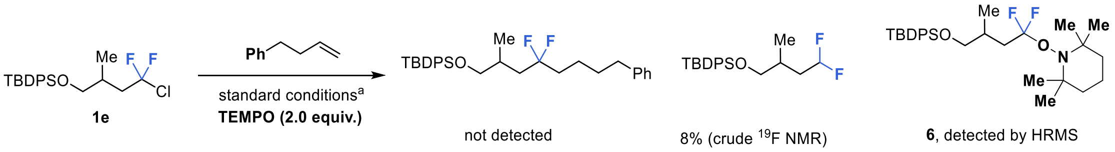

Following the general procedure **C**, adding 2.0 equivalents of TEMPO into the reaction mixture, the desired addition product was not detected, whereas the decholorination and RCF_2H-TEMPO compounds was detected by ^{19}F NMR or HRMS, indicating the formation of RCF_2 radical during the reaction process.

RCF_2H-TEMPO HRMS-ESI (m/z): Calcd for $C_{30}H_{46}F_2NO_2Si^+$ $[M+H]^+$ 518.3260, found 518.3262

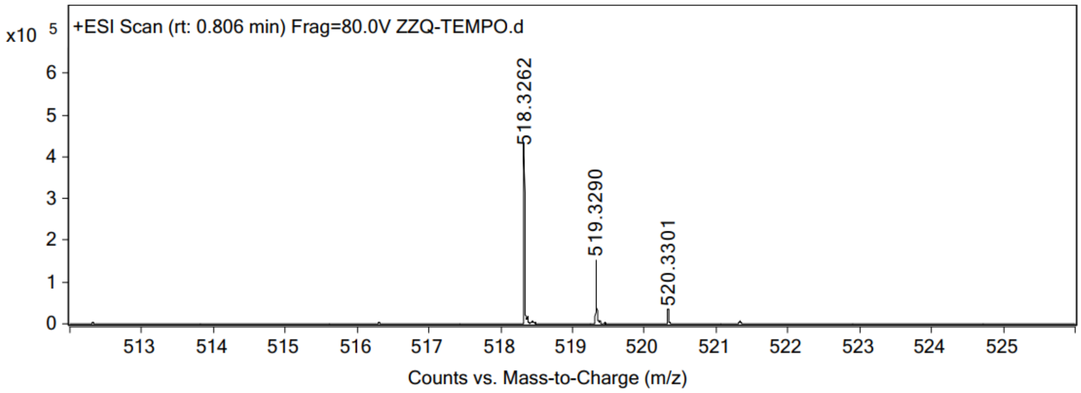

c) Radical clock experiments

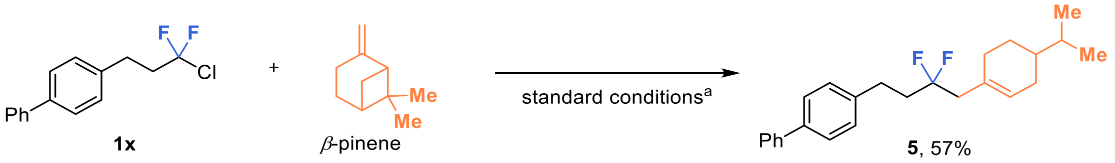

Following the general procedure **C**, the ring-opening product **5** (21.0 mg, white solid, m.p. 53.1-54.5 °C) was obtained in 57% yield. **^{1}H NMR** (400 MHz, $CDCl_3$) δ 7.61 – 7.56 (m, 2H), 7.56 – 7.51 (m, 2H), 7.46 – 7.40 (m, 2H), 7.36 – 7.31 (m, 1H), 7.30 – 7.26 (m, 2H), 5.59 (s, 1H), 3.01 – 2.73 (m, 2H), 2.55 (t, J = 16.4 Hz, 2H), 2.22 – 2.03 (m, 5H), 1.84 – 1.73 (m, 2H), 1.54 – 1.41 (m, 1H), 1.29 – 1.16 (m, 2H), 0.89 (dd, J = 6.8, 5.0 Hz, 6H). **^{19}F NMR** (377 MHz, $CDCl_3$) δ -94.51 – -95.93 (m). **^{13}C NMR** (126 MHz, $CDCl_3$) δ 141.1, 140.2, 139.3, 130.8 (t, J = 4.1 Hz), 128.9, 127.8, 127.4, 127.3, 127.2, 124.6 (t, J = 242.8 Hz), 45.1 (t, J = 25.1 Hz), 39.8, 38.0 (t, J = 25.1 Hz), 32.3, 30.1, 29.3, 28.2 (t, J = 4.8 Hz), 26.6, 20.1, 19.8. HRMS-EI (m/z): Calcd for $C_{25}H_{30}F_2^+$ $[M]^+$ 368.2310, found 368.2314.

d) Deuterium labelling study

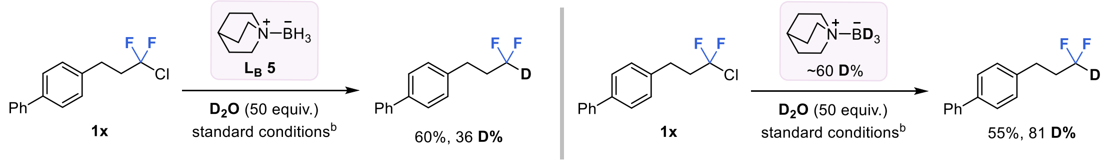

Following the general procedure **C**, Reaction 1) was added 50 equivalents of D_2O into the reaction mixture; Reaction 2) was added 50 equivalents of D_2O into the reaction mixture and used L-BD_3 (~ 60% D-inc.) with L-BH_3. The desired products were obtained inabout 60% yield, however, the deuteration incorporation of the latter product was significantly higher than the former one (81 % or 36%, respectively).

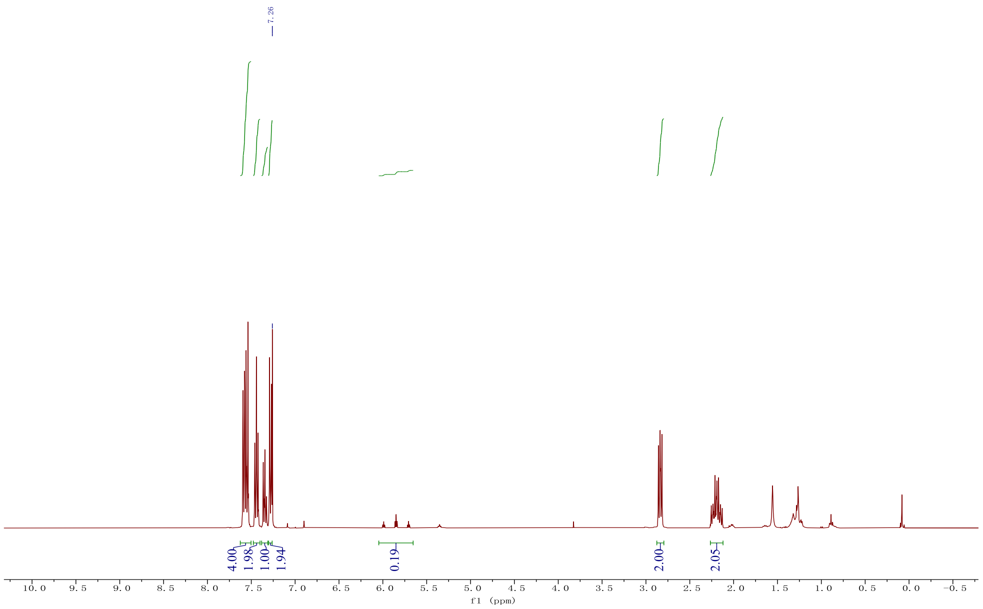

e) Stern-volmer fluorescence quenching studies

Stern-Volmer luminescence quenching experiments were carried out with freshly prepared solutions of PC (10^{-5} M) in tBuCN at room temperature. The solutions were irradiated at 440 nm and the luminescence was measured at 450 nm.

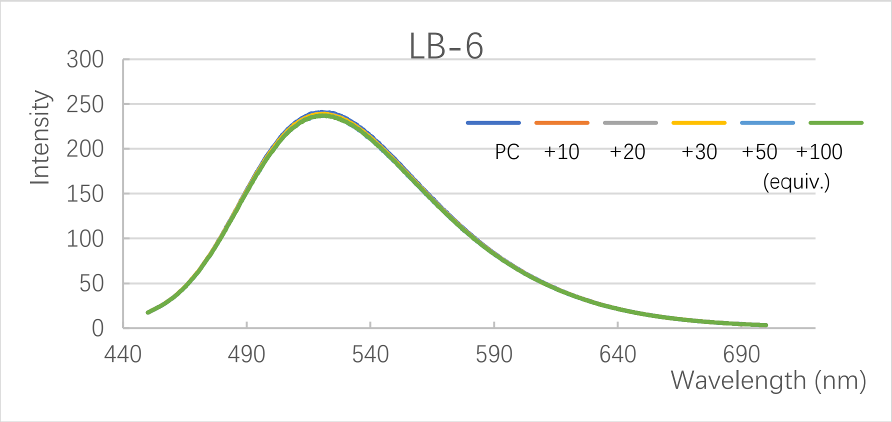

Supplementary Fig. 3: Fluorescence quenching study of 4CzIPN with different equivalents of **LB-6**

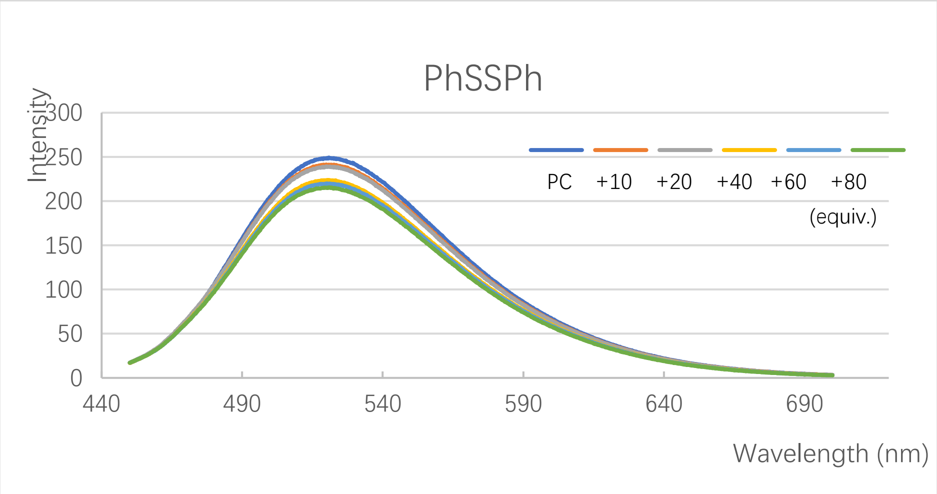

Supplementary Fig. 4: Fluorescence quenching study of 4CzIPN with different equivalents of **PhSSPh**

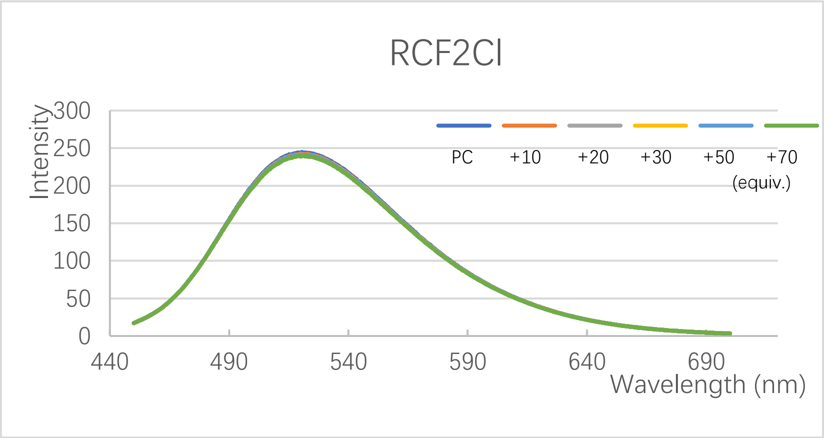

Supplementary Fig. 5: Fluorescence quenching study of 4CzIPN with different equivalents of **RCF_2Cl**

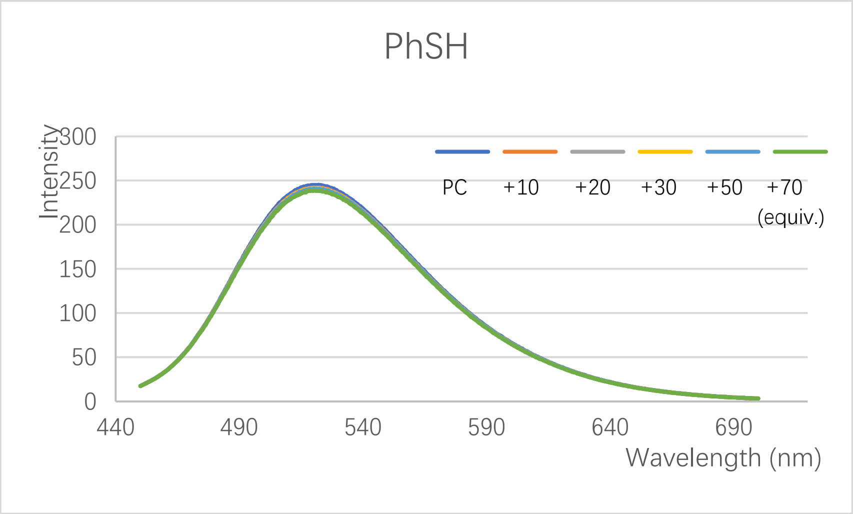

Supplementary Fig. 6: Fluorescence quenching study of 4CzIPN with different equivalents of **PhSH**

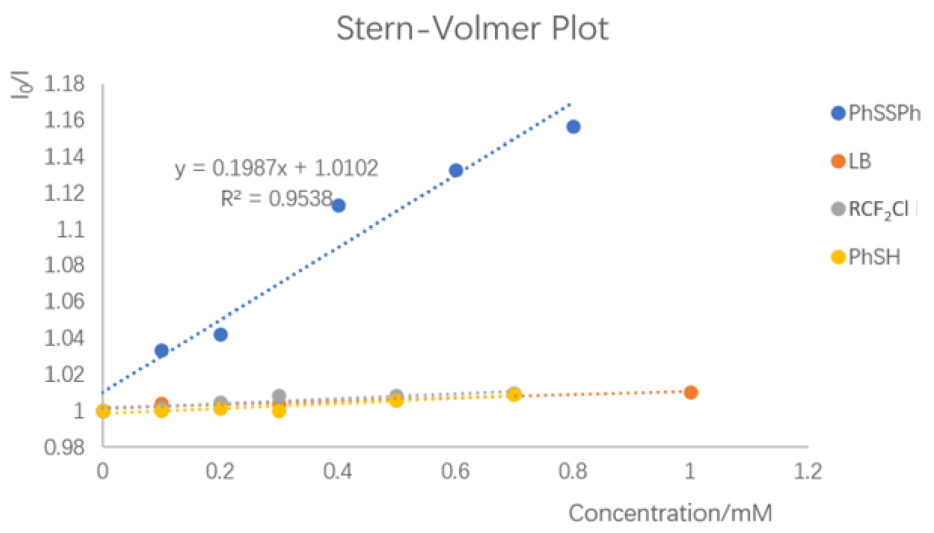

Supplementary Fig. 7: Combined quenching data.

f) Determination of photochemical quantum yields

Determination of Photochemical Quantum Yields Follow McMullen's procedure for photon flux[7], the following solutions were prepared ahead of time:

1. Ferrioxalate solution A 0.15 M solution of potassium ferrioxolate was prepared by dissolving potassium ferrioxolate ($K_3Fe(C_2O_4)_3$*$3H_2O$) (1.842 g, 3.75 mmol) with the 0.05 M sulfuric acid solution prepared in a 25 mL volumetric flask. Make every precaution to prepare and store the solution in the dark.
2. Developer solution 67.8 g of sodium acetate was dissolved in 500 ml of 0.5 M sulfuric acid. 5 g of 1,10- phenanthroline was added to this solution. Store in the dark.

To determine the photon flux of the Kessil lamp, 2.0 mL of the ferrioxalate solution was placed in a 10 mL microwave tube and irradiated at λ = 456 nm with an emission slit width of 10.0 nm. After irradiation, 10 µL aliquots of the solution were taken at different time points between 0.5 and 3 minutes of irradiation. This aliquot is immediately added to 5 mL of the developer solution and the flask is wrapped in aluminum foil. A blank sample is prepared by adding 10 µL of the ferrioxalate solution to 5 mL of developer solution. The solutions were left in the dark for one hour, eventually becoming bright red. Solutions were transferred to a separate cuvette and the absorbance spectrum of the $Fe(phen)_3^{2+}$ complex was obtained. The absorbance at 510 nm (ε = 11,100 $M^{-1}cm^{-1}$) was measured for each sample. The conversion was calculated using **eq 1.**

$$\text{mol } Fe^{2+} = \frac{V_1 \cdot V_2 \cdot \Delta A}{V_2 \cdot l \cdot \varepsilon} \qquad \textbf{eq 1}$$

ΔA = the difference between the absorbance between the sample and the blank as measured at 510 nm.

l = the path length of the cuvette (1 cm)

ε = the extinction coefficient of $Fe(phen)_3{}^{2+}$ complex at 510 nm (11,100 $M^{-1}cm^{-1}$)
V_1 = the total volume of the irradiated solution (2 mL; 2 x 10^{-3} L)
V_2 = the volume of the aliquot removed from solution (10 μL; 1 x 10^{-5} L)
V_3 = the volume that aliquots are diluted with (5 mL; 5 x 10^{-3} L)

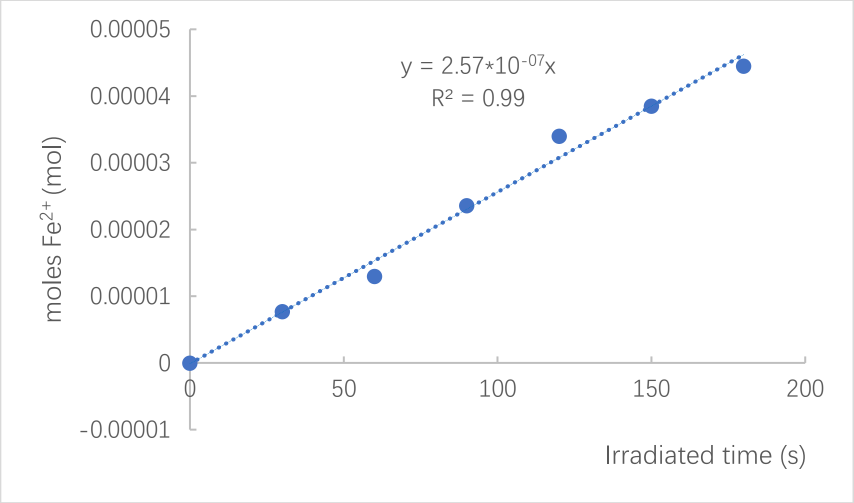

Supplementary Fig. 9: Compiled linear fits for the photon flux

A plot of moles Fe^{2+} as a function of time yields a linear equation with an intercept at zero. The value of the slopes collected is 2.57 x 10^{-7} $mol^{-1}s^{-1}$. The photon flux can be calculated using **eq 2**.

$$\text{Photo flux} = \frac{\text{mol } Fe^{2+}}{\Phi \cdot t \cdot f} \qquad \textbf{eq 2}$$

The documented quantum yield of the actinometer (Φ = 0.84 at 458 nm)[8] and f is the fraction of light absorbed at λ = 456 nm (0.95, vide infra)[9]. The photon flux in einsteins s^{-1}.

$$\text{Photo flux} = \frac{2.57 \times 10^{-7}}{0.84 \times 0.95} = 3.22 \times 10^{-7}$$

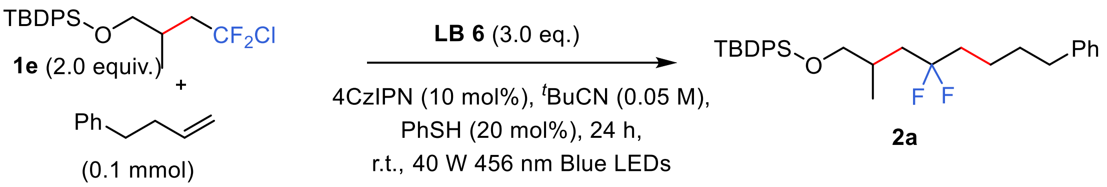

Following the general procedure **C**, the reaction time was shorted to 24 hours. The quantum yield was determined using **eq 3**. Essentially, all incident light (f = 1, vide infra) is absorbed by the 4CzIPN at the reaction conditions described above.

$$\Phi = \frac{\text{mol product}}{\text{flux} \cdot t \cdot f} \qquad \textbf{eq 3}$$

Experiment: RCF_2Cl compond (**1e**, 0.2 mmol), but-3-en-1-ylbenzene (0.10 mmol), **LB-6** (0.3 mmol), 4CzIPN (0.01 mmol) and PhSH (0.02 mmol) in ^{t}BuCN (2.0 mL) after 36000 s yielded 15% of **2a**. Φ = 0.0013.

$$\Phi = \frac{1.5 \times 10^{-5}}{3.22 \times 10^{-7} \times 36000 \times 1.00} = 0.0013$$

VII. X-Ray crystallographic data

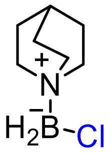

4

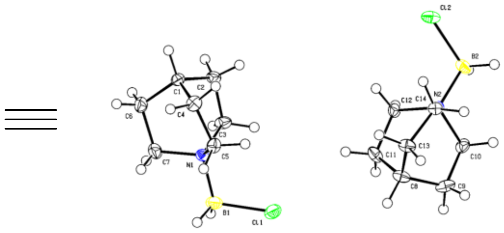

CCDC 2242761

Supplementary Table 7. Crystal data and structure refinement for M445.

Identification code	M445	
Empirical formula	C7 H15 B Cl N	
Formula weight	159.46	
Temperature	100(2) K	
Wavelength	0.71073 Å	
Crystal system	Monoclinic	
Space group	$P2_1/c$	
Unit cell dimensions	a = 12.9872(8) Å	α= 90°.
	b = 12.2112(7) Å	β= 91.520(2)°.
	c = 10.7243(6) Å	γ = 90°.
Volume	1700.16(17) $Å^3$	
Z	8	
Density (calculated)	1.246 Mg/m^3	
Absorption coefficient	0.374 mm^{-1}	
F(000)	688	
Crystal size	0.376 x 0.372 x 0.170 mm^3	
Theta range for data collection	2.528 to 28.282°.	
Index ranges	-17<=h<=17, -15<=k<=16, -14<=l<=14	
Reflections collected	41178	
Independent reflections	4222 [R(int) = 0.0448]	

Completeness to theta = 25.242°	99.9 %
Absorption correction	Semi-empirical from equivalents
Max. and min. transmission	0.7459 and 0.6996
Refinement method	Full-matrix least-squares on F^2
Data / restraints / parameters	4222 / 0 / 193
Goodness-of-fit on F^2	1.060
Final R indices [I>2sigma(I)]	R1 = 0.0406, wR2 = 0.1082
R indices (all data)	R1 = 0.0438, wR2 = 0.1112
Extinction coefficient	n/a
Largest diff. peak and hole	0.973 and -0.449 e.Å^{-3}

Supplementary Table 8. Atomic coordinates (x 10^4) and equivalent isotropic displacement parameters (Å^{2}x 10^3)
for M445. U(eq) is defined as one third of the trace of the orthogonalized U^{ij} tensor.

	x	y	z	U(eq)
Cl(1)	1821(1)	7813(1)	5497(1)	29(1)
Cl(2)	3162(1)	2568(1)	9542(1)	26(1)
N(1)	3471(1)	8982(1)	6697(1)	16(1)
N(2)	1512(1)	3224(1)	7880(1)	14(1)
C(1)	4693(1)	9081(1)	8641(1)	20(1)
C(2)	4774(1)	7960(1)	8009(1)	21(1)
C(3)	4149(1)	7987(1)	6770(1)	23(1)
C(4)	3574(1)	9264(1)	9010(1)	23(1)
C(5)	2863(1)	9044(1)	7874(1)	20(1)
C(6)	4962(1)	9973(1)	7706(2)	26(1)
C(7)	4142(1)	9987(1)	6649(1)	24(1)
C(8)	470(1)	4868(1)	6995(1)	22(1)
C(9)	62(1)	3791(1)	6447(1)	26(1)
C(10)	813(1)	2867(1)	6813(1)	21(1)
C(11)	1606(1)	4975(1)	6675(1)	25(1)
C(12)	2223(1)	4092(1)	7394(1)	18(1)
C(13)	387(1)	4811(1)	8417(1)	21(1)
C(14)	869(1)	3726(1)	8876(1)	16(1)
B(1)	2753(1)	8998(1)	5456(2)	25(1)
B(2)	2123(1)	2159(1)	8362(2)	22(1)

Supplementary Table 9. Bond lengths [Å] and angles [°] for M445.

Cl(1)-B(1)	1.8882(18)
Cl(2)-B(2)	1.8925(17)
N(1)-C(3)	1.5018(16)
N(1)-C(7)	1.5066(18)
N(1)-C(5)	1.5073(17)
N(1)-B(1)	1.6047(19)
N(2)-C(14)	1.5033(15)
N(2)-C(10)	1.5067(16)
N(2)-C(12)	1.5083(16)
N(2)-B(2)	1.6021(18)
C(1)-C(6)	1.528(2)
C(1)-C(2)	1.5320(19)
C(1)-C(4)	1.5323(19)
C(1)-H(1)	1.0000
C(2)-C(3)	1.5386(19)
C(2)-H(2A)	0.9900
C(2)-H(2B)	0.9900
C(3)-H(3A)	0.9900
C(3)-H(3B)	0.9900
C(4)-C(5)	1.5330(19)
C(4)-H(4A)	0.9900
C(4)-H(4B)	0.9900
C(5)-H(5A)	0.9900
C(5)-H(5B)	0.9900
C(6)-C(7)	1.535(2)
C(6)-H(6A)	0.9900
C(6)-H(6B)	0.9900
C(7)-H(7A)	0.9900
C(7)-H(7B)	0.9900
C(8)-C(11)	1.530(2)
C(8)-C(9)	1.530(2)
C(8)-C(13)	1.5331(19)
C(8)-H(8)	1.0000
C(9)-C(10)	1.536(2)
C(9)-H(9A)	0.9900
C(9)-H(9B)	0.9900
C(10)-H(10A)	0.9900

C(10)-H(10B)	0.9900
C(11)-C(12)	1.5375(19)
C(11)-H(11A)	0.9900
C(11)-H(11B)	0.9900
C(12)-H(12A)	0.9900
C(12)-H(12B)	0.9900
C(13)-C(14)	1.5404(18)
C(13)-H(13A)	0.9900
C(13)-H(13B)	0.9900
C(14)-H(14A)	0.9900
C(14)-H(14B)	0.9900
B(1)-H(1A)	1.07(2)
B(1)-H(1B)	1.26(2)
B(2)-H(2C)	1.14(2)
B(2)-H(2D)	1.08(2)

C(3)-N(1)-C(7)	108.80(11)
C(3)-N(1)-C(5)	108.45(11)
C(7)-N(1)-C(5)	107.68(11)
C(3)-N(1)-B(1)	112.34(11)
C(7)-N(1)-B(1)	106.56(11)
C(5)-N(1)-B(1)	112.83(11)
C(14)-N(2)-C(10)	108.85(10)
C(14)-N(2)-C(12)	108.43(10)
C(10)-N(2)-C(12)	107.63(10)
C(14)-N(2)-B(2)	112.42(10)
C(10)-N(2)-B(2)	107.03(10)
C(12)-N(2)-B(2)	112.32(10)
C(6)-C(1)-C(2)	109.11(12)
C(6)-C(1)-C(4)	107.51(11)
C(2)-C(1)-C(4)	108.70(11)
C(6)-C(1)-H(1)	110.5
C(2)-C(1)-H(1)	110.5
C(4)-C(1)-H(1)	110.5
C(1)-C(2)-C(3)	108.82(11)
C(1)-C(2)-H(2A)	109.9
C(3)-C(2)-H(2A)	109.9
C(1)-C(2)-H(2B)	109.9

C(3)-C(2)-H(2B)	109.9
H(2A)-C(2)-H(2B)	108.3
N(1)-C(3)-C(2)	110.92(11)
N(1)-C(3)-H(3A)	109.5
C(2)-C(3)-H(3A)	109.5
N(1)-C(3)-H(3B)	109.5
C(2)-C(3)-H(3B)	109.5
H(3A)-C(3)-H(3B)	108.0
C(1)-C(4)-C(5)	108.93(11)
C(1)-C(4)-H(4A)	109.9
C(5)-C(4)-H(4A)	109.9
C(1)-C(4)-H(4B)	109.9
C(5)-C(4)-H(4B)	109.9
H(4A)-C(4)-H(4B)	108.3
N(1)-C(5)-C(4)	110.89(10)
N(1)-C(5)-H(5A)	109.5
C(4)-C(5)-H(5A)	109.5
N(1)-C(5)-H(5B)	109.5
C(4)-C(5)-H(5B)	109.5
H(5A)-C(5)-H(5B)	108.0
C(1)-C(6)-C(7)	109.01(11)
C(1)-C(6)-H(6A)	109.9
C(7)-C(6)-H(6A)	109.9
C(1)-C(6)-H(6B)	109.9
C(7)-C(6)-H(6B)	109.9
H(6A)-C(6)-H(6B)	108.3
N(1)-C(7)-C(6)	110.88(11)
N(1)-C(7)-H(7A)	109.5
C(6)-C(7)-H(7A)	109.5
N(1)-C(7)-H(7B)	109.5
C(6)-C(7)-H(7B)	109.5
H(7A)-C(7)-H(7B)	108.1
C(11)-C(8)-C(9)	108.23(12)
C(11)-C(8)-C(13)	108.70(12)
C(9)-C(8)-C(13)	108.06(12)
C(11)-C(8)-H(8)	110.6
C(9)-C(8)-H(8)	110.6
C(13)-C(8)-H(8)	110.6

C(8)-C(9)-C(10)	108.84(11)
C(8)-C(9)-H(9A)	109.9
C(10)-C(9)-H(9A)	109.9
C(8)-C(9)-H(9B)	109.9
C(10)-C(9)-H(9B)	109.9
H(9A)-C(9)-H(9B)	108.3
N(2)-C(10)-C(9)	110.29(11)
N(2)-C(10)-H(10A)	109.6
C(9)-C(10)-H(10A)	109.6
N(2)-C(10)-H(10B)	109.6
C(9)-C(10)-H(10B)	109.6
H(10A)-C(10)-H(10B)	108.1
C(8)-C(11)-C(12)	108.74(11)
C(8)-C(11)-H(11A)	109.9
C(12)-C(11)-H(11A)	109.9
C(8)-C(11)-H(11B)	109.9
C(12)-C(11)-H(11B)	109.9
H(11A)-C(11)-H(11B)	108.3
N(2)-C(12)-C(11)	110.49(10)
N(2)-C(12)-H(12A)	109.6
C(11)-C(12)-H(12A)	109.6
N(2)-C(12)-H(12B)	109.6
C(11)-C(12)-H(12B)	109.6
H(12A)-C(12)-H(12B)	108.1
C(8)-C(13)-C(14)	108.58(11)
C(8)-C(13)-H(13A)	110.0
C(14)-C(13)-H(13A)	110.0
C(8)-C(13)-H(13B)	110.0
C(14)-C(13)-H(13B)	110.0
H(13A)-C(13)-H(13B)	108.4
N(2)-C(14)-C(13)	110.63(10)
N(2)-C(14)-H(14A)	109.5
C(13)-C(14)-H(14A)	109.5
N(2)-C(14)-H(14B)	109.5
C(13)-C(14)-H(14B)	109.5
H(14A)-C(14)-H(14B)	108.1
N(1)-B(1)-Cl(1)	109.29(10)
N(1)-B(1)-H(1A)	104.4(11)

Cl(1)-B(1)-H(1A)	108.5(11)
N(1)-B(1)-H(1B)	119.4(9)
Cl(1)-B(1)-H(1B)	100.1(10)
H(1A)-B(1)-H(1B)	114.7(15)
N(2)-B(2)-Cl(2)	109.84(10)
N(2)-B(2)-H(2C)	107.7(10)
Cl(2)-B(2)-H(2C)	109.6(10)
N(2)-B(2)-H(2D)	109.1(11)
Cl(2)-B(2)-H(2D)	108.9(10)
H(2C)-B(2)-H(2D)	111.7(15)

Supplementary Table 10. Anisotropic displacement parameters ($Å^2$x 10^3) for M445. The anisotropic displacement factor exponent takes the form: $-2\pi^2[h^2 a^{*2}U^{11} + ... + 2 h k a^* b^* U^{12}]$

	U^{11}	U^{22}	U^{33}	U^{23}	U^{13}	U^{12}
Cl(1)	24(1)	24(1)	38(1)	-7(1)	-11(1)	4(1)
Cl(2)	20(1)	34(1)	22(1)	6(1)	-1(1)	8(1)
N(1)	15(1)	15(1)	19(1)	0(1)	0(1)	4(1)
N(2)	15(1)	13(1)	13(1)	-1(1)	1(1)	-1(1)
C(1)	20(1)	17(1)	22(1)	-2(1)	-5(1)	1(1)
C(2)	21(1)	17(1)	23(1)	-1(1)	-5(1)	5(1)
C(3)	22(1)	20(1)	26(1)	-7(1)	-7(1)	10(1)
C(4)	24(1)	25(1)	19(1)	-4(1)	2(1)	0(1)
C(5)	15(1)	22(1)	23(1)	-1(1)	4(1)	0(1)
C(6)	18(1)	21(1)	38(1)	4(1)	-1(1)	-4(1)
C(7)	24(1)	22(1)	28(1)	7(1)	2(1)	-3(1)
C(8)	25(1)	24(1)	18(1)	2(1)	0(1)	10(1)
C(9)	20(1)	39(1)	17(1)	-4(1)	-5(1)	5(1)
C(10)	24(1)	22(1)	16(1)	-4(1)	-3(1)	-4(1)
C(11)	29(1)	20(1)	26(1)	9(1)	4(1)	1(1)
C(12)	15(1)	18(1)	20(1)	3(1)	3(1)	-3(1)
C(13)	24(1)	23(1)	17(1)	-3(1)	0(1)	9(1)
C(14)	16(1)	20(1)	14(1)	-1(1)	2(1)	2(1)
B(1)	25(1)	25(1)	24(1)	2(1)	-6(1)	5(1)
B(2)	29(1)	16(1)	21(1)	1(1)	0(1)	6(1)

Supplementary Table 11. Hydrogen coordinates (x 10^4) and isotropic displacement parameters ($Å^2$x 10^{3})

for M445.

	x	y	z	U(eq)
H(1)	5165	9119	9392	24
H(2A)	4500	7384	8560	25
H(2B)	5504	7790	7850	25
H(3A)	4626	7992	6065	27
H(3B)	3718	7319	6699	27
H(4A)	3483	10026	9301	28
H(4B)	3400	8763	9699	28
H(5A)	2491	8346	7993	24
H(5B)	2347	9638	7794	24
H(6A)	5648	9826	7360	31
H(6B)	4987	10695	8127	31
H(7A)	3709	10650	6724	29
H(7B)	4482	10017	5835	29
H(8)	66	5502	6652	27
H(9A)	0	3848	5527	31
H(9B)	-628	3632	6772	31
H(10A)	420	2210	7060	25
H(10B)	1231	2670	6087	25
H(11A)	1863	5711	6911	30
H(11B)	1689	4878	5767	30
H(12A)	2727	3752	6835	22
H(12B)	2610	4434	8100	22
H(13A)	-345	4845	8649	26
H(13B)	754	5437	8810	26
H(14A)	1305	3864	9631	20
H(14B)	316	3210	9102	20
H(1A)	2324(16)	9747(18)	5520(19)	37
H(1B)	3149(15)	8834(18)	4418(18)	37
H(2C)	1548(15)	1597(17)	8820(18)	33
H(2D)	2481(15)	1766(17)	7577(18)	33

Supplementary Table 12. Torsion angles [°] for M445.

C(6)-C(1)-C(2)-C(3)	-51.00(15)
C(4)-C(1)-C(2)-C(3)	65.96(15)
C(7)-N(1)-C(3)-C(2)	65.96(15)
C(5)-N(1)-C(3)-C(2)	-50.90(15)

B(1)-N(1)-C(3)-C(2)	-176.29(12)
C(1)-C(2)-C(3)-N(1)	-12.87(16)
C(6)-C(1)-C(4)-C(5)	67.07(15)
C(2)-C(1)-C(4)-C(5)	-50.91(15)
C(3)-N(1)-C(5)-C(4)	66.54(14)
C(7)-N(1)-C(5)-C(4)	-51.04(14)
B(1)-N(1)-C(5)-C(4)	-168.35(12)
C(1)-C(4)-C(5)-N(1)	-13.28(16)
C(2)-C(1)-C(6)-C(7)	65.58(15)
C(4)-C(1)-C(6)-C(7)	-52.13(15)
C(3)-N(1)-C(7)-C(6)	-50.98(15)
C(5)-N(1)-C(7)-C(6)	66.37(14)
B(1)-N(1)-C(7)-C(6)	-172.33(12)
C(1)-C(6)-C(7)-N(1)	-12.51(17)
C(11)-C(8)-C(9)-C(10)	48.87(15)
C(13)-C(8)-C(9)-C(10)	-68.68(14)
C(14)-N(2)-C(10)-C(9)	48.26(14)
C(12)-N(2)-C(10)-C(9)	-69.06(13)
B(2)-N(2)-C(10)-C(9)	170.00(11)
C(8)-C(9)-C(10)-N(2)	17.06(16)
C(9)-C(8)-C(11)-C(12)	-67.78(15)
C(13)-C(8)-C(11)-C(12)	49.36(16)
C(14)-N(2)-C(12)-C(11)	-68.05(13)
C(10)-N(2)-C(12)-C(11)	49.54(14)
B(2)-N(2)-C(12)-C(11)	167.11(11)
C(8)-C(11)-C(12)-N(2)	15.90(16)
C(11)-C(8)-C(13)-C(14)	-67.46(15)
C(9)-C(8)-C(13)-C(14)	49.78(14)
C(10)-N(2)-C(14)-C(13)	-67.49(13)
C(12)-N(2)-C(14)-C(13)	49.33(13)
B(2)-N(2)-C(14)-C(13)	174.11(11)
C(8)-C(13)-C(14)-N(2)	15.47(15)
C(3)-N(1)-B(1)-Cl(1)	64.80(13)
C(7)-N(1)-B(1)-Cl(1)	-176.14(10)
C(5)-N(1)-B(1)-Cl(1)	-58.17(13)
C(14)-N(2)-B(2)-Cl(2)	-68.95(12)
C(10)-N(2)-B(2)-Cl(2)	171.59(9)
C(12)-N(2)-B(2)-Cl(2)	53.67(13)

VIII. Product characterizations

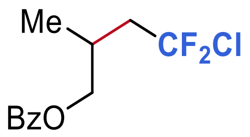

4-chloro-4,4-difluoro-2-methylbutyl benzoate (**1a**)

Following the general procedure **A**, the title compound (46.1 mg, colorless liquid) was obtained in 86% yield. **^{1}H NMR** (400 MHz, $CDCl_3$) δ 8.04 (dd, *J* = 8.4, 1.4 Hz, 2H), 7.74 – 7.53 (m, 1H), 7.52 – 7.42 (m, 2H), 4.37 – 4.06 (m, 2H), 2.59 (qd, *J* = 14.8, 4.9 Hz, 1H), 2.45 (dq, *J* = 12.4, 6.2 Hz, 1H), 2.29 (tdd, *J* = 14.8, 11.2, 7.6 Hz, 1H), 1.19 (d, *J* = 6.8 Hz, 3H). **^{19}F NMR** (377 MHz, $CDCl_3$) δ -47.65 (ddd, *J* = 161.5, 15.0, 11.3 Hz), -49.10 (dt, *J* = 161.5, 14.9 Hz). **^{13}C NMR** (126 MHz, $CDCl_3$) δ 166.5, 133.3, 130.1, 129.7 (t, *J* = 292.6 Hz), 129.7, 128.6, 68.5, 45.3, 29.3 (t, *J* = 2.0 Hz), 17.4. HRMS-EI (m/z): Calcd for $C_{12}H_{13}ClF_2O_2^+$ $[M]^+$ 262.0567, found 262.0567.

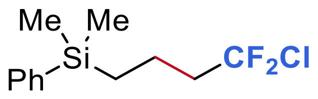

(4-chloro-4,4-difluorobutyl)dimethyl(phenyl)silane (**1b**)

Following the general procedure **A**, the title compound (37.2 mg, colourless liquid) was obtained in 71% yield.

^{1}H NMR (400 MHz, $CDCl_3$) δ 7.55 – 7.48 (m, 2H), 7.41 – 7.34 (m, 3H), 2.35 – 2.24 (m, 2H), 1.69 – 1.60 (m, 2H), 0.84 – 0.78 (m, 2H), 0.31 (s, 6H). **^{19}F NMR** (377 MHz, $CDCl_3$) δ -50.50 (td, *J* = 13.3, 2.7 Hz). **^{13}C NMR** (126 MHz, $CDCl_3$) δ 138.7, 133.6, 129.9 (t, *J* = 292.6 Hz),129.2, 128.0, 45.4 (t, *J* = 23.2 Hz), 18.1 (t, *J* = 3.2 Hz), 15.2, -3.1. HRMS-EI (m/z): Calcd for $C_{11}H_{14}ClF_2Si^+$ $[M-15]^+$ 247.0516, found 247.0523.

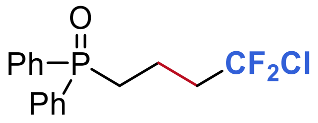

(4-chloro-4,4-difluorobutyl)diphenylphosphine oxide (**1c**)

Following the general procedure **A**, the title compound (49.2 mg, white solid, m.p. 92.0-92.8 ^oC) was obtained in 75% yield,

^{1}H NMR (400 MHz, $CDCl_3$) δ 7.87 – 7.63 (m, 4H), 7.59 – 7.41 (m, 6H), 2.51 – 2.39 (m, 2H), 2.38 – 2.29 (m, 2H), 2.01 – 1.90 (m, 2H). **^{19}F NMR** (377 MHz, $CDCl_3$) δ -50.57 (tt, *J* = 12.9, 2.1 Hz). **^{31}P NMR** (162 MHz, $CDCl_3$) δ 31.58. **^{13}C NMR** (126 MHz, $CDCl_3$) δ 132.5 (d, *J* = 99.1 Hz) 132.1 (d, *J* = 2.8 Hz), 130.8 (d, *J* = 9.4 Hz), 129.5 (td, *J* = 291.4 Hz, 1.2 Hz), 128.9 (d, *J* = 11.7 Hz), 42.4 (td, *J* = 24.4, 13.4 Hz), 28.8 (d, *J* = 71.8 Hz), 16.3 (q, *J* = 3.3 Hz). HRMS-EI (m/z): Calcd for $C_{16}H_{15}ClF_2OP$ $[M-H]^+$ 327.0512, found 327.0498.

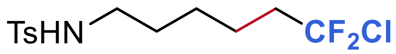

N-(6-chloro-6,6-difluorohexyl)-4-methylbenzenesulfonamide (**1d**)
Following the general procedure **A**, the title compound (46.2 mg, colourless liquid) was obtained in 71% yield.
^{1}H NMR (400 MHz, $CDCl_3$) δ 8.00 – 7.67 (m, 2H), 7.39 – 7.23 (m, 2H), 4.80 (t, *J* = 6.1 Hz, 1H), 2.93 (q, *J* = 6.8 Hz, 2H), 2.42 (s, 3H), 2.29 – 2.11 (m, 2H), 1.57 – 1.41 (m, 4H), 1.39 – 1.28 (m, 2H). **^{19}F NMR** (377 MHz, $CDCl_3$) δ -50.65 (td, *J* = 13.0, 2.8 Hz). **^{13}C NMR** (126 MHz, $CDCl_3$) δ 143.6, 137.0, 132.2 (t, *J* = 293.0 Hz)., 129.9, 127.2, 42.9, 41.7 (t, *J* = 23.9 Hz), 29.3, 25.5, 22.9 (t, *J* = 3.0 Hz), 21.6. HRMS-EI (m/z): Calcd for $C_{13}H_{18}ClF_2NO_2S^+$ $[M]^+$ 325.0709, found 325.0707.

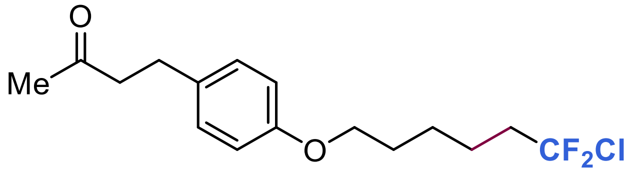

4-(4-((6-chloro-6,6-difluorohexyl)oxy)phenyl)butan-2-one (**1ae**)
Following the general procedure **A**, the title compound (41.4 mg, colourless liquid) was obtained in 65% yield.
^{1}H NMR (400 MHz, $CDCl_3$) δ 7.13 – 7.05 (m, 2H), 6.85 – 6.76 (m, 2H), 3.94 (t, *J* = 6.3 Hz, 2H), 2.92 – 2.67 (m, 4H), 2.40 – 2.24 (m, 2H), 2.13 (s, 3H), 1.80 (dq, *J* = 7.8, 6.3 Hz, 2H), 1.75 – 1.63 (m, 2H), 1.61 – 1.51 (m, 2H). **^{19}F NMR** (377 MHz, $CDCl_3$) δ -50.57 (td, *J* = 12.9, 2.7 Hz). **^{13}C NMR** (126 MHz, $CDCl_3$) δ 208.3, 157.5, 133.2, 130.1 (t, *J* = 291.7 Hz), 129.4, 114.6, 67.6, 45.6, 41.9 (t, *J* = 23.8 Hz), 30.3, 29.1, 29.0, 25.4, 23.2 (t, *J* = 3.1 Hz). HRMS-EI (m/z): Calcd for $C_{16}H_{21}ClF_2O_2^+$ $[M]^+$ 318.1193, found 318.1196.

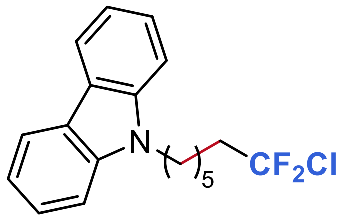

9-(7-chloro-7,7-difluoroheptyl)-9H-carbazole (**1f**)
Following the general procedure **A**, the title compound (55.6 mg, colourless liquid) was obtained in 83 % yield.
^{1}H NMR (400 MHz, $CDCl_3$) δ 8.14 (dt, *J* = 7.8, 1.0 Hz, 2H), 7.50 (ddd, *J* = 8.2, 7.0, 1.2 Hz, 2H), 7.44 – 7.39 (m, 2H), 7.27 (ddd, *J* = 8.0, 7.0, 1.0 Hz, 2H), 4.32 (t, *J* = 7.1 Hz, 2H), 2.32 – 2.16 (m, 2H), 1.91 (pd, *J* = 7.2, 4.0 Hz, 2H), 1.65 – 1.53 (m, 2H), 1.41 (p, *J* = 2.8 Hz, 4H). **^{19}F NMR** (377 MHz, $CDCl_3$) δ -50.58 (td, *J* = 13.2, 3.2 Hz). **^{13}C NMR** (126 MHz, $CDCl_3$) δ 140.5, 130.0 (t, *J* = 291.9 Hz), 125.8, 123.0, 120.5, 118.9, 108.7, 43.0, 41.8 (t, *J* = 23.8 Hz), 28.9, 28.5, 27.1, 23.3 (t, *J* = 3.0 Hz). HRMS-EI (m/z): Calcd for $C_{19}H_{20}ClF_2N^+$ $[M]^+$ 335.1252, found 335.1254.

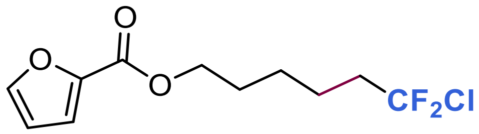

6-chloro-6,6-difluorohexyl furan-2-carboxylate (**1g**)

Following the general procedure **A**, the title compound (31.9 mg, colourless liquid) was obtained in 60% yield.

^{1}H NMR (400 MHz, $CDCl_3$) δ 7.57 (dd, *J* = 1.8, 0.9 Hz, 1H), 7.17 (dd, *J* = 3.5, 0.9 Hz, 1H), 6.51 (dd, *J* = 3.5, 1.8 Hz, 1H), 4.31 (t, *J* = 6.5 Hz, 2H), 2.60 – 2.12 (m, 2H), 1.85 – 1.74 (m, 2H), 1.73 – 1.63 (m, 2H), 1.58 – 1.46 (m, 2H). **^{19}F NMR** (377 MHz, $CDCl_3$) δ -50.63 (td, *J* = 12.9, 2.7 Hz). **^{13}C NMR** (126 MHz, $CDCl_3$) δ 158.9, 146.4, 130.0 (t, *J* = 291.6 Hz), 118.0, 112.0, 64.7, 41.8 (t, *J* = 23.9 Hz), 28.5, 25.2, 23.1 (t, *J* = 3.0 Hz). HRMS-EI (m/z): Calcd for $C_{11}H_{13}ClF_2O_3^+$ [M $^+$ 266.0516, found 266.0515.

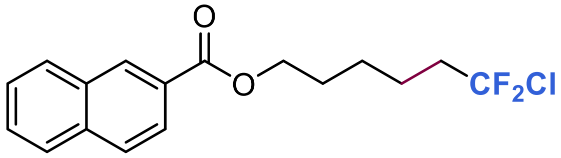

6-chloro-6,6-difluorohexyl 2-naphthoate (**1ap**)

Following the general procedure **A**, the title compound (39.1 mg, white solid, m.p. 49.0-50.0 ^{o}C) was obtained in 60% yield.

^{1}H NMR (400 MHz, $CDCl_3$) δ 8.60 (d, *J* = 1.6 Hz, 1H), 8.06 (dd, *J* = 8.5, 1.7 Hz, 1H), 7.96 (d, *J* = 8.1 Hz, 1H), 7.89 (d, *J* = 8.7 Hz, 2H), 7.65 – 7.51 (m, 2H), 4.40 (t, *J* = 6.5 Hz, 2H), 2.42 – 2.26 (m, 2H), 1.87 (dq, *J* = 8.4, 6.5 Hz, 2H), 1.74 (tt, *J* = 8.0, 6.3 Hz, 2H), 1.64 – 1.54 (m, 2H). **^{19}F NMR** (377 MHz, $CDCl_3$) δ -50.57 (td, *J* = 13.0, 2.8 Hz). **^{13}C NMR** (126 MHz, $CDCl_3$) δ 166.9, 135.7, 132.6, 131.1, 130.0, 129.5, 128.4, 128.3, 127.9, 127.7, 126.8, 125.3, 64.9, 41.9 (t, *J* = 23.9 Hz), 28.6, 25.4, 23.2 (t, *J* = 3.1 Hz). HRMS-EI (m/z): Calcd for $C_{17}H_{17}ClF_2O_2^+$ $[M]^+$ 326.0880, found 326.0878.

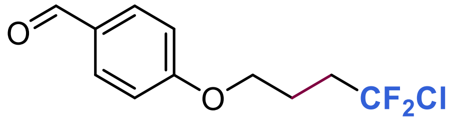

4-(4-chloro-4,4-difluorobutoxy)benzaldehyde (**1i**)

Following the general procedure **A**, the title compound (31.9 mg, colourless liquid) was obtained in 75% yield.

^{1}H NMR (400 MHz, $CDCl_3$) δ 9.89 (s, 1H), 7.84 (d, *J* = 8.8 Hz, 2H), 6.99 (d, *J* = 8.7 Hz, 2H), 4.12 (t, *J* = 6.0 Hz, 2H), 2.69 – 2.39 (m, 2H), 2.30 – 1.84 (m, 2H). **^{19}F NMR** (377 MHz, $CDCl_3$) δ -50.84 (td, *J* = 12.9, 2.7 Hz). **^{13}C NMR** (126 MHz, $CDCl_3$) δ 190.9, 163.7, 132.2, 130.4, 129.8 (t, *J* = 291.8 Hz), 114.8, 66.5, 38.8 (t, *J* = 24.7 Hz), 23.5 (t, *J* = 3.2 Hz). HRMS-EI (m/z): Calcd for $C_{11}H_{11}ClF_2O_2^+$ $[M]^+$ 248.0410, found 248.0412.

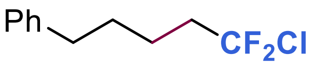

(5-chloro-5,5-difluoropentyl)benzene (**1j**)

Following the general procedure **A**, the title compound (34.0 mg, colorless liquid) was obtained in 78% yield. **^{1}H NMR** (400 MHz, $CDCl_3$) δ 7.35 – 7.27 (m, 2H), 7.25 – 7.15 (m, 3H), 2.66 (t, *J* = 7.1 Hz, 2H), 2.41 – 2.22 (m, 2H), 1.79 – 1.62 (m, 4H). **^{19}F NMR** (377 MHz, $CDCl_3$) δ -50.50 (td, *J* = 12.9, 2.7 Hz). **^{13}C NMR** (126 MHz, $CDCl_3$) δ 141.8, 131.3 (t, *J* = 291.8 Hz), 128.6,128.5,126.1, 41.8 (t, *J* = 23.8 Hz), 35.7, 30.5, 23.0 (t, *J* = 3.0 Hz). HRMS-EI (m/z): Calcd for $C_{11}H_{13}ClF_2^+$ $[M]^+$ 218.0674, found 218.0669.

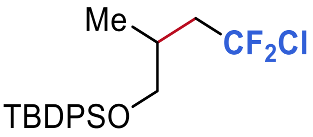

tert-butyl(4-chloro-4,4-difluoro-2-methylbutoxy)diphenylsilane (**1k**)

Following the general procedure **A**, the title compound (72.1 mg, colourless liquid) was obtained in 91% yield (5 mmol scale, 88% yield, 1.746 g). **^{1}H NMR** (400 MHz, $CDCl_3$) δ 7.68 – 7.61 (m, 4H), 7.47 – 7.35 (m, 6H), 3.57 (dd, *J* = 10.4, 4.5 Hz, 1H), 3.46 (dd, *J* = 10.1, 6.2 Hz, 1H), 2.74 – 2.59 (m, 1H), 2.18 – 2.04 (m, 2H), 1.07 (s, 9H), 1.04 (dt, *J* = 6.6, 1.0 Hz, 3H). **^{19}F NMR** (377 MHz, $CDCl_3$) δ -46.83 (ddd, *J* = 159.0, 15.1, 9.3 Hz), (-48.43) – (-49.19) (m). **^{13}C NMR** (126 MHz, $CDCl_3$) δ 135.7, 133.6, 130.4 (t, *J* = 292.5 Hz), 129.9, 127.9, 67.9, 44.9 (t, *J* = 22.9 Hz), 32.1, 27.0, 19.4, 17.1. HRMS-EI (m/z): Calcd for $C_{21}H_{28}ClF_2OSi^+$ $[M+H]^+$ 397.1561, found 397.1557.

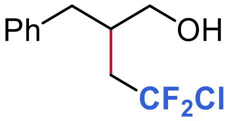

2-benzyl-4-chloro-4,4-difluorobutan-1-ol (**1l**)

Following the general procedure **A**, the title compound (24.3 mg, colourless liquid) was obtained in 52% yield.

^{1}H NMR (400 MHz, $CDCl_3$) δ 7.36 – 7.30 (m, 2H), 7.23 – 7.17 (m, 3H), 3.78 – 3.50 (m, 2H), 2.86 – 2.71 (m, 2H), 2.65 – 2.48 (m, 1H), 2.40 – 2.17 (m, 2H). **^{19}F NMR** (377 MHz, $CDCl_3$) δ -47.31 – -47.95 (m), -48.11 – -48.69 (m). **^{13}C NMR** (126 MHz, $CDCl_3$) δ 139.2, 130.2 (t, *J* = 292.3 Hz), 129.3, 128.7, 126.6, 63.3, 42.4 (t, *J* = 23.2 Hz), 38.4, 37.5. HRMS-EI (m/z): Calcd for $C_{11}H_{13}ClF_2O^+$ $[M]^+$ 234.0618, found 234.0616.

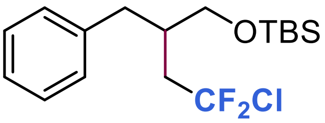

(2-benzyl-4-chloro-4,4-difluorobutoxy)(tert-butyl)dimethylsilane (**1m**)

Following the general procedure **A**, the title compound (64.1 mg, colourless liquid) was obtained in 92% yield.

^{1}**H NMR** (400 MHz, $CDCl_3$) δ 7.35 – 7.26 (m, 2H), 7.26 – 7.16 (m, 3H), 3.71 – 3.36 (m, 2H), 2.84 – 2.67 (m, 2H), 2.64 – 2.47 (m, 1H), 2.34 – 2.17 (m, 2H), 0.93 (d, *J* = 0.9 Hz, 9H), 0.04 (d, *J* = 2.1 Hz, 6H). ^{19}**F NMR** (377 MHz, $CDCl_3$) δ -47.09 – -47.67 (m), -48.17 (dt, *J* = 160.0, 14.1 Hz). ^{13}**C NMR** (126 MHz, $CDCl_3$) δ 139.6, 130.4 (t, *J* = 292.5 Hz), 129.4, 128.5, 126.4, 63.0, 42.5 (t, *J* = 23.0 Hz), 38.6, 37.4, 26.0, 18.4, -5.4, -5.5. HRMS-EI (m/z): Calcd for $C_{17}H_{26}ClF_2OSi^+$ $[M-H]^+$ 347.1404, found 347.1409.

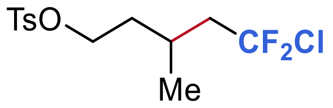

5-chloro-5,5-difluoro-3-methylpentyl 4-methylbenzenesulfonate (**1n**)
Following the general procedure **A**, the title compound (58.0 mg, colourless liquid) was obtained in 89% yield.
^{1}**H NMR** (400 MHz, $CDCl_3$) δ 7.91 – 7.61 (m, 2H), 7.50 – 7.33 (m, 2H), 4.08 (dd, *J* = 6.9, 5.9 Hz, 2H), 2.45 (s, 3H), 2.32 – 1.98 (m, 3H), 1.80 (dtd, *J* = 14.0, 6.8, 5.3 Hz, 1H), 1.62 – 1.49 (m, 1H), 0.97 (d, *J* = 6.7 Hz, 3H). ^{19}**F NMR** (377 MHz, $CDCl_3$) δ -47.39 (ddd, *J* = 160.9, 15.3, 11.0 Hz), -48.78 (dt, *J* = 161.2, 14.7 Hz). ^{13}**C NMR** (126 MHz, $CDCl_3$) δ 145.1, 133.1, 130.1, 129.7 (t, *J* = 292.7 Hz), 128.0, 67.9, 48.1 (t, *J* = 22.9 Hz), 35.5, 26.0 (t, *J* = 1.9 Hz), 21.8, 19.4. HRMS-EI (m/z): Calcd for $C_{13}H_{17}ClF_2O_3S^+$ $[M]^+$ 326.0550, found 326.0550.

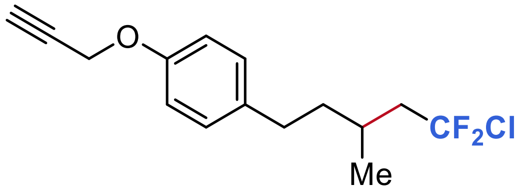

1-(5-Chloro-5,5-difluoro-3-methylpentyl)-4-(prop-2-yn-1-yloxy)benzene (**1o**)
Following the general procedure **A**, the title compound (44.6 mg, colourless liquid) was obtained in 78% yield.
^{1}**H NMR** (500 MHz, $CDCl_3$) δ 7.14 – 7.06 (m, 2H), 6.94 – 6.88 (m, 2H), 4.67 (d, *J* = 2.4 Hz, 2H), 2.63 (ddd, *J* = 13.7, 6.8, 3.5 Hz, 1H), 2.59 – 2.52 (m, 1H), 2.51 (t, *J* = 2.4 Hz, 1H), 2.37 (qd, *J* = 14.9, 4.9 Hz, 1H), 2.17 (tdd, *J* = 15.1, 11.0, 7.8 Hz, 1H), 1.96 (hept, *J* = 6.9, 6.3 Hz, 1H), 1.74 – 1.65 (m, 1H), 1.58 – 1.51 (m, 1H), 1.08 (d, *J* = 6.7 Hz, 3H) ppm. ^{19}**F NMR** (377 MHz, $CDCl_3$) δ -47.03 (ddd, *J* = 160.0, 15.8, 11.3 Hz), -48.56 (dt, *J* = 159.9, 14.9 Hz). ^{13}**C NMR** (126 MHz, $CDCl_3$) δ 156.0, 135.2, 130.2 (t, *J* = 292.6 Hz), 129.4, 115.0, 79.0, 75.5, 56.0, 48.4 (t, *J* = 22.5 Hz), 39.0, 32.2, 28.9, 20.0. HRMS-EI (m/z): Calcd for $C_{15}H_{17}ClF_2O^+$ $[M]^+$ 286.0931, found 286.0934.

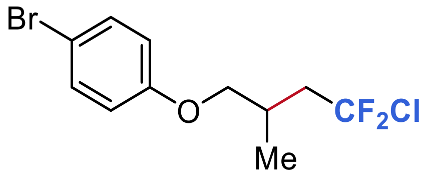

1-bromo-4-(4-chloro-4,4-difluoro-2-methylbutoxy)benzene (**1p**)
Following the general procedure **A**, the title compound (48.0 mg, colourless liquid) was obtained in 77% yield.
^{1}H NMR (400 MHz, $CDCl_3$) δ 7.43 – 7.28 (m, 2H), 6.91 – 6.63 (m, 2H), 3.99 – 3.52 (m, 2H), 2.66 (dtd, *J* = 15.5, 14.6, 5.0 Hz, 1H), 2.48 – 2.36 (m, 1H), 2.26 (tdd, *J* = 15.0, 11.3, 7.6 Hz, 1H), 1.18 (dt, *J* = 6.8, 1.0 Hz, 3H). **^{19}F NMR** (377 MHz, $CDCl_3$) δ -47.51 (ddd, *J* = 160.9, 15.6, 11.6 Hz), -48.87 (dt, *J* = 161.2, 14.8 Hz). **^{13}C NMR** (126 MHz, $CDCl_3$) δ 155.0, 132.4, 128.8 (t, *J* = 292.3 Hz), 116.4, 113.25, 71.9, 45.0 (t, *J* = 23.3 Hz), 29.7 (t, *J* = 2.2 Hz), 17.3. HRMS-EI (m/z): Calcd for $C_{11}H_{12}BrClF_2O^+$ $[M]^+$ 311.9723, found 311.9729.

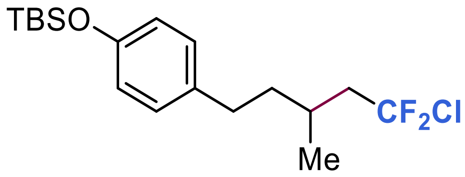

tert-butyl(4-(5-chloro-5,5-difluoro-3-methylpentyl)phenoxy)dimethylsilane (**1q**)
Following the general procedure A, the title compound (57.2mg, colourless liquid) was obtained in 79% yield.
^{1}H NMR (500 MHz, $CDCl_3$) δ 7.04 – 7.00 (m, 2H), 6.78 – 6.73 (m, 2H), 2.60 (ddd, *J* = 13.7, 10.3, 5.8 Hz, 1H), 2.53 (ddd, *J* = 13.8, 10.2, 6.1 Hz, 1H), 2.43 – 2.31 (m, 1H), 2.16 (tdd, *J* = 15.2, 11.0, 7.9 Hz, 1H), 1.96 (h, *J* = 7.7, 7.2 Hz, 1H), 1.69 (ddt, *J* = 13.6, 10.2, 5.9 Hz, 1H), 1.58 – 1.49 (m, 1H), 1.07 (d, *J* = 5.6 Hz, 3H), 0.98 (s, 9H), 0.18 (s, 6H). **^{19}F NMR** (377 MHz, $CDCl_3$) δ -46.96 (ddd, *J* = 159.9, 15.7, 11.0 Hz), -48.57 (dt, *J* = 159.8, 15.0 Hz). **^{13}C NMR** (126 MHz, $CDCl_3$) δ 153.9, 134.7, 130.3 (t, *J* = 292.4 Hz), 129.3, 120.1, 48.4 (t, *J* = 22.4 Hz), 39.0, 32.3, 28.8, 25.9, 20.0, 18.4, -4.3. HRMS-EI (m/z): Calcd for $C_{18}H_{29}ClF_2OSi^+$ $[M]^+$ 362.1639, found 362.1642.

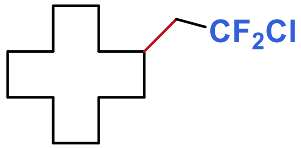

(2-chloro-2,2-difluoroethyl)cyclododecane (**1r**)
Following the general procedure **A**, the title compound (37.3 mg, colourless liquid) was obtained in 70% yield.
^{1}H NMR (400 MHz, $CDCl_3$) δ 2.22 (td, *J* = 14.3, 6.1 Hz, 2H), 1.94 (qt, *J* = 7.2, 3.4 Hz, 1H), 1.52 – 1.18 (m, 22H). **^{19}F NMR** (377 MHz, $CDCl_3$) δ -47.86 (td, *J* = 14.2, 2.7 Hz). **^{13}C NMR** (126 MHz, $CDCl_3$) δ 130.6 (t, *J* = 293.0 Hz), 46.8 (t, *J* = 22.1 Hz), 30.2, 29.5, 24.6, 24.1, 23.5, 23.4, 21.6. HRMS-EI (m/z): Calcd for $C_{14}H_{25}ClF_2^+$ $[M]^+$

266.1607, found 266.1609.

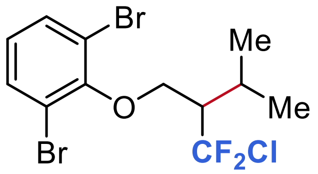

1,3-dibromo-2-(2-(chlorodifluoromethyl)-3-methylbutoxy)benzene (**1s**)

Following the general procedure **A**, the title compound (75.9 mg, colourless liquid) was obtained in 94% yield.

^{1}H NMR (400 MHz, $CDCl_3$) δ 7.52 (d, *J* = 8.0 Hz, 2H), 6.89 (t, *J* = 8.0 Hz, 1H), 4.22 (qd, *J* = 9.5, 5.6 Hz, 2H), 2.89 (tddd, *J* = 12.1, 6.1, 5.0, 3.0 Hz, 1H), 2.48 (heptd, *J* = 7.1, 3.0 Hz, 1H), 1.23 (d, *J* = 7.1 Hz, 3H), 1.14 (d, *J* = 7.0 Hz, 3H). **^{19}F NMR** (377 MHz, $CDCl_3$) δ -49.90 (d, *J* = 12.2 Hz). **^{13}C NMR** (126 MHz, $CDCl_3$) δ 153.3, 133.0, 131.3 (t, *J* = 297.1 Hz), 126.9, 118.6, 69.2 (t, *J* = 3.6 Hz), 55.5 (t, *J* = 20.1 Hz), 27.2 (t, *J* = 1.8 Hz), 21.3, 19.0. HRMS-EI (m/z): Calcd for $C_{12}H_{13}Br_2ClF_2O^+$ $[M]^+$ 403.8984, found 403.8982.

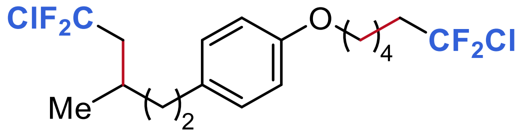

1-(5-chloro-5,5-difluoro-3-methylpentyl)-4-((6-chloro-6,6-difluorohexyl)oxy)benzene (**1t**)

Following the general procedure **A**, the title compound (57.1 mg, colourless liquid) was obtained in 71% yield. **^{1}H NMR** (400 MHz, $CDCl_3$) δ 7.15 – 7.03 (m, 2H), 6.93 – 6.69 (m, 2H), 3.95 (t, *J* = 6.3 Hz, 2H), 2.68 – 2.49 (m, 2H), 2.43 – 2.28 (m, 3H), 2.23 – 2.11 (m, 1H), 2.03 – 1.92 (m, 1H), 1.81 (dq, *J* = 8.2, 6.4 Hz, 2H), 1.69 (dtt, *J* = 9.8, 6.0, 2.8 Hz, 3H), 1.61 – 1.50 (m, 3H), 1.08 (dt, *J* = 6.7, 1.1 Hz, 3H). **^{19}F NMR** (377 MHz, $CDCl_3$) δ -47.00 (dd, *J* = 159.8, 2.4 Hz), -48.11 – -49.12 (m), -50.56 (d, *J* = 2.5 Hz). **^{13}C NMR** (126 MHz, $CDCl_3$) δ 157.3, 134.1, 130.2 (t, *J* = 293.0 Hz), 130.1 (t, *J* = 291.8 Hz), 129.3, 114.6, 67.6, 48.4 (t, *J* = 22.4 Hz), 41.9 (t, *J* = 23.8 Hz), 39.1, 32.2, 29.1, 28.8, 25.4, 23.2 (t, *J* = 3.1 Hz), 20.0. HRMS-EI (m/z): Calcd for $C_{18}H_{24}Cl_2F_4O^+$ $[M]^+$ 402.1135, found 402.1133.

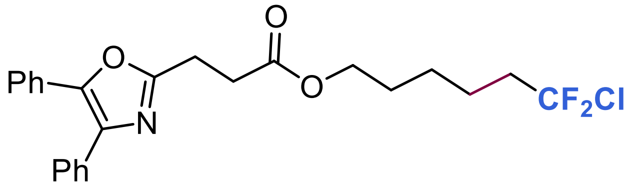

6-chloro-6,6-difluorohexyl 3-(4,5-diphenyloxazol-2-yl)propanoate (**1u**)

Following the general procedure **A**, the title compound (65.4 mg, colourless liquid) was obtained in 73% yield (5 mml scale, 65% yield, 1.456 g).

^{1}H NMR (400 MHz, $CDCl_3$)δ 7.65 – 7.61 (m, 2H), 7.60 – 7.54 (m, 2H), 7.43 – 7.27

(m, 6H), 4.14 (t, J = 6.5 Hz, 2H), 3.19 (dd, J = 8.0, 6.9 Hz, 2H), 2.92 (dd, J = 8.0, 6.9 Hz, 2H), 2.32 – 2.07 (m, 2H), 1.74 – 1.48 (m, 4H), 1.42 (qd, J = 8.5, 7.2, 3.9 Hz, 2H). **^{19}F NMR** (377 MHz, $CDCl_3$) δ -50.61 (td, *J* = 12.9, 3.0 Hz). **^{13}C NMR** (126 MHz, $CDCl_3$) δ 172.1, 161.9, 145.6, 135.2, 132.5, 130.0 (t, *J* = 292.1 Hz), 129.1, 128.8, 128.7, 128.6, 128.2, 128.0, 126.6, 64.5, 41.7 (t, *J* = 24.0 Hz), 31.3, 28.4, 25.1, 23.7, 23.1 (t, *J* = 3.1 Hz). HRMS-EI (m/z): Calcd for $C_{24}H_{25}ClF_2NO_3^+$ $[M+H]^+$ 448.1486, found 448.1483

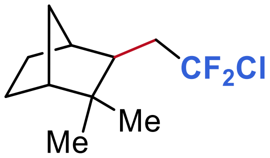

3-(2-chloro-2,2-difluoroethyl)-2,2-dimethylbicyclo[2.2.1]heptane (**1v**)

Following the general procedure **A**, the title compound (31.1 mg, colourless liquid) was obtained in 70% yield.

^{1}H NMR (400 MHz, $CDCl_3$) δ 2.40 – 2.17 (m, 2H), 1.81 – 1.71 (m, 2H), 1.65 (dtd, *J* = 9.9, 3.3, 1.7 Hz, 1H), 1.60 – 1.52 (m, 1H), 1.37 – 1.18 (m, 5H), 0.98 (s, 3H), 0.81 (s, 3H). **^{19}F NMR** (377 MHz, $CDCl_3$) δ -47.60 (dd, *J* = 158.7, 2.7 Hz), -50.15 (dd, *J* = 158.8, 2.8 Hz). **^{13}C NMR** (126 MHz, $CDCl_3$) δ 130.8 (t, *J* = 292.7 Hz), 48.6, 45.3, 41.9, 39.2 (t, *J* = 23.0 Hz), 37.4, 37.1, 31.9, 24.7, 22.2, 20.4. HRMS-EI (m/z): Calcd for $C_{11}H_{17}ClF_2^+$ $[M]^+$ 222.0981, found 222.0977.

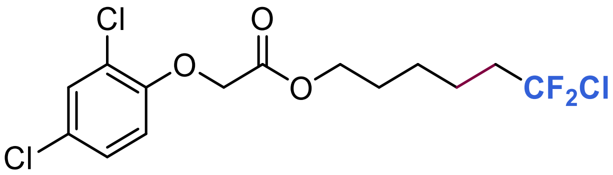

6-chloro-6,6-difluorohexyl 2-(2,4-dichlorophenoxy)acetate (1**w**)

Following the general procedure **A**, the title compound (67.3 mg, colourless liquid) was obtained in 90% yield.

^{1}H NMR (400 MHz, $CDCl_3$) δ 7.33 (d, *J* = 2.5 Hz, 1H), 7.10 (dd, *J* = 8.8, 2.6 Hz, 1H), 6.71 (d, *J* = 8.8 Hz, 1H), 4.62 (s, 2H), 4.14 (t, *J* = 6.5 Hz, 2H), 2.48 – 2.00 (m, 2H), 1.86 – 1.41 (m, 4H), 1.33 (qd, *J* = 8.2, 7.2, 3.5 Hz, 2H). **^{19}F NMR** (377 MHz, $CDCl_3$) δ -50.59 (td, *J* = 13.0, 2.9 Hz). **^{13}C NMR** (126 MHz, $CDCl_3$) δ 168.3, 152.5, 130.5, 129.9 (t, *J* = 291.7 Hz), 127.7, 127.3, 124.4, 114.8, 66.5, 65.2, 41.7 (t, *J* = 23.9 Hz), 28.3, 25.0, 23.0 (t, *J* = 3.2 Hz). HRMS-EI (m/z): Calcd for $C_{14}H_{15}Cl_3F_2O_3^+$ $[M]^+$ 374.0049, found 374.0055.

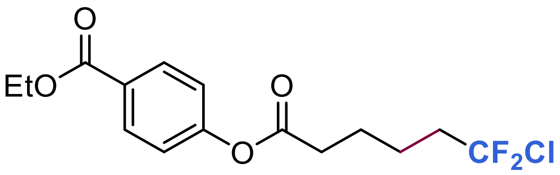

ethyl 4-((6-chloro-6,6-difluorohexanoyl)oxy)benzoate (**1x**)

Following the general procedure **A**, the title compound (58.8 mg, colourless liquid)

was obtained in 88 % yield. ^{1}H NMR (400 MHz, $CDCl_3$) δ 8.10 – 8.05 (m, 2H), 7.18 – 7.13 (m, 2H), 4.37 (q, J = 7.1 Hz, 2H), 2.62 (t, J = 7.2 Hz, 2H), 2.42 – 2.28 (m, 2H), 1.90 – 1.80 (m, 2H), 1.79 – 1.70 (m, 2H), 1.38 (t, J = 7.1 Hz, 3H). **^{19}F NMR** (377 MHz, $CDCl_3$) δ -50.67 (td, J = 12.7, 2.8 Hz). **^{13}C NMR** (126 MHz, $CDCl_3$) δ 171.1, 165.9, 154.2, 131.3, 129.8 (t, J = 292.4 Hz), 128.2, 121.6, 61.2, 41.6 (t, J = 24.2 Hz), 34.0, 23.8, 22.9 (t, J = 3.2 Hz), 14.4. HRMS-EI (m/z): Calcd for $C_{15}H_{17}ClF_2O_4^+$ $[M]^+$ 334.0783, found 334.0786. Calcd for $C_{16}H_{23}ClF_2O^+$ $[M]^+$ 304.1400, found 304.1404.

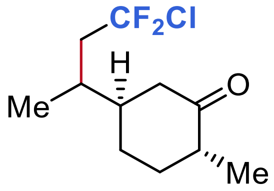

(2R,5R)-5-(4-chloro-4,4-difluorobutan-2-yl)-2-methylcyclohexan-1-one (**1y**)

Following the general procedure **A**, the title compound (36.3 mg, d.r. = 1/1, colourless liquid) was obtained in 76% yield.

^{1}H NMR (400 MHz, $CDCl_3$) δ 2.48 – 2.25 (m, 3H), 2.20 – 2.05 (m, 3H), 2.02 – 1.88 (m, 1H), 1.87 – 1.70 (m, 2H), 1.59 – 1.42 (m, 1H), 1.40 – 1.26 (m, 1H), 1.03 (t, J = 6.4 Hz, 6H). **^{19}F NMR** (377 MHz, $CDCl_3$) δ -47.23 (dd, J = 160.2, 2.8 Hz), -49.49 (ddd, J = 160.7, 63.7, 2.4 Hz). **^{13}C NMR** (126 MHz, $CDCl_3$) δ 212.5, 212.4, 130.2 (t, J = 292.8 Hz), 46.1 (t, J = 22.7 Hz), 45.9 (t, J = 22.7 Hz), 45.5, 45.1, 45.0, 44.9, 44.0, 34.7, 34.7, 33.4, 29.2, 27.5, 16.4, 16.4, 14.4, 14.4. HRMS-EI (m/z): Calcd for $C_{11}H_{18}ClF_2O^+$ $[M+H]^+$ 239.1009, found 239.1011.

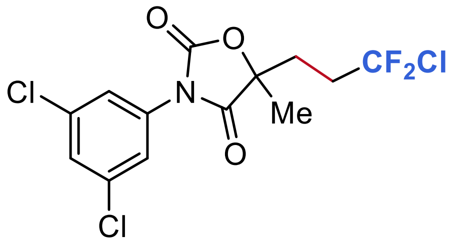

5-(3-chloro-3,3-difluoropropyl)-3-(3,5-dichlorophenyl)-5-methyloxazolidine-2,4-dione (**1z**)

Following the general procedure **A**, the title compound (72.7 mg, white solid, m.p. 114.0-114.7 °C) was obtained in 98% yield.

^{1}H NMR (400 MHz, $CDCl_3$) δ 7.45 (d, J = 1.8 Hz, 2H), 7.44 – 7.42 (m, 1H), 2.62 – 2.48 (m, 1H), 2.45 – 2.33 (m, 1H), 2.29 (ddt, J = 9.4, 5.9, 2.9 Hz, 2H), 1.71 (s, 3H). **^{19}F NMR** (377 MHz, $CDCl_3$) δ -51.44 (tdd, J = 11.9, 5.6, 2.8 Hz). **^{13}C NMR** (126 MHz, $CDCl_3$) δ 172.9, 151.9, 135.8, 132.4, 129.3, 128.6 (t, J = 291.4 Hz), 123.8, 84.2, 36.0 (t, J = 25.8 Hz), 30.7 (t, J = 3.2 Hz), 22.3. HRMS-EI (m/z): Calcd for $C_{13}H_{10}Cl_3F_2NO_3^+$ $[M]^+$ 370.9689, found 370.9691.

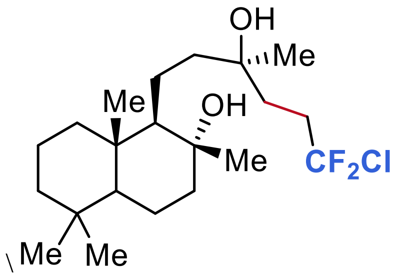

(1R,2R,8aS)-1-((R)-6-chloro-6,6-difluoro-3-hydroxy-3-methylhexyl)-2,5,5,8a-tetramethyldecahydronaphthalen-2-ol (**1aa**)

Following the general procedure **A**, the title compound (51.2 mg, white solid, m.p. 155.7-156.6 °C) was obtained in 65% yield.

^{1}H NMR (400 MHz, $CDCl_3$) δ 2.42 (ttd, *J* = 14.8, 7.6, 5.9 Hz, 3H), 1.84 (dt, *J* = 12.1, 3.2 Hz, 1H), 1.75 – 1.50 (m, 8H), 1.47 – 1.22 (m, 5H), 1.19 – 1.09 (m, 8H), 0.98 – 0.90 (m, 1H), 0.86 (s, 3H), 0.79 (s, 3H), 0.78 (s, 3H). **^{19}F NMR** (377 MHz, $CDCl_3$) δ -50.37 (td, *J* = 13.0, 3.6 Hz). **^{13}C NMR** (126 MHz, $CDCl_3$) δ 130.7 (t, *J* = 291.2 Hz), 75.3, 71.8, 61.9, 56.2, 44.8, 44.5, 42.1, 39.8, 39.4, 37.0 (t, *J* = 24.0 Hz), 36.6, 33.5, 33.4, 25.67, 24.5, 21.6, 20.7, 18.9, 18.5, 15.5. HRMS-EI (m/z): Calcd for $C_{20}H_{35}ClF_2O_2$ $[M-18]^+$ 376.2339, found 376.2345.

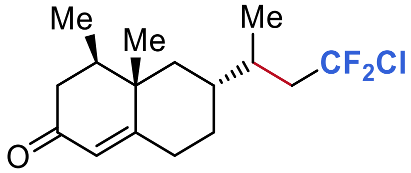

(4R,4aS,6R)-6-((S)-4-chloro-4,4-difluorobutan-2-yl)-4,4a-dimethyl-4,4a,5,6,7,8-hexahydronaphthalen-2(3H)-one (**1ab**)

Following the general procedure **A**, the title compound (36.5 mg, d.r. = 1/1, colourless liquid) was obtained in 60% yield.

^{1}H NMR (400 MHz, $CDCl_3$) δ 5.75 (d, *J* = 1.8 Hz, 1H), 2.51 – 2.31 (m, 3H), 2.29 – 2.23 (m, 1H)., 2.14 (tdd, *J* = 15.1, 8.3, 4.2 Hz, 1H), 2.05 – 1.67 (m, 6H), 1.19 (ddd, *J* = 13.2, 8.6, 4.3 Hz, 1H), 1.08 (d, *J* = 3.4 Hz, 3H), 1.04 – 0.92 (m, 7H). **^{19}F NMR** (377 MHz, $CDCl_3$) δ -47.08 (ddt, *J* = 160.1, 15.7, 11.6 Hz), -49.09 (ddt, *J* = 160.2, 99.6, 15.2 Hz). **^{13}C NMR** (126 MHz, $CDCl_3$) δ 199.6, 170.4, 170.3, 130.4 (t, *J* = 292.7 Hz), 124.8 (d, *J* = 2.3 Hz), 46.3 (t, *J* = 22.4 Hz), 45.9 (t, *J* = 22.5 Hz), 42.5, 42.2, 40.7, 40.7, 40.6, 39.4, 39.3, 37.7, 37.6, 33.4, 33.1, 32.9, 29.9, 28.2, 17.0, 17.0, 16.7, 16.4, 15.1, 15.1. HRMS-EI (m/z): Calcd for $C_{16}H_{23}ClF_2O^+$ $[M]^+$ 304.1400, found 304.1404.

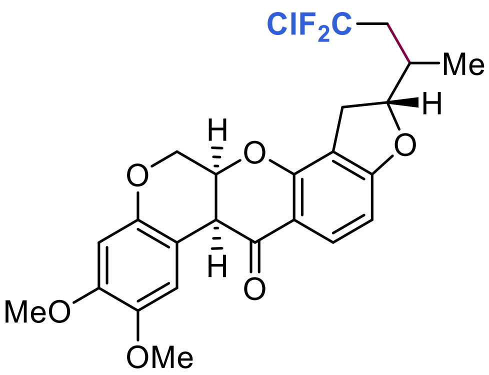

(2R,6aS,12aS)-2-(4-chloro-4,4-difluorobutan-2-yl)-8,9-dimethoxy-1,2,12,12a-tetrahydrochromeno[3,4-b]furo[2,3-h]chromen-6(6aH)-one (**1ac**)

Following the general procedure **A**, the title compound (72.0 mg, d.r = 1/1, colourless liquid) was obtained in 75% yield.

^{1}H NMR (400 MHz, $CDCl_3$) δ 7.94 – 7.76 (m, 1H), 6.79 – 6.73 (m, 1H), 6.53 – 6.42 (m, 2H), 5.23 (t, *J* = 9.0 Hz, 0.5H), 5.06 (dt, *J* = 1.7, 1.0 Hz, 0.5H), 4.92 (qd, *J* = 3.0, 1.9 Hz, 1.5H), 4.87 – 4.64 (m, 0.5H), 4.64 – 4.54 (m, 1H), 4.17 (ddt, *J* = 12.0, 2.0, 1.1 Hz, 1H), 3.85 – 3.81 (m, 1H), 3.80 (d, *J* = 0.8 Hz, 3H), 3.75 (d, *J* = 1.7 Hz, 3H), 3.38 – 3.13 (m, 1H), 2.91 (ddd, *J* = 32.6, 15.8, 8.2 Hz, 1H), 2.78 – 2.44 (m, 0.5H), 2.33 – 2.15 (m, 1H), 1.76 (t, *J* = 1.2 Hz, 1.5H), 1.67 (s, 0.5H), 1.15 – 1.11 (m, 1H), 1.11 – 1.03 (m, 0.5H). **^{19}F NMR** (377 MHz, $CDCl_3$) δ -46.85 (dddd, *J* = 197.4, 161.7, 15.5, 9.1 Hz), -48.59 – -50.00 (m). **^{13}C NMR** (126 MHz, $CDCl_3$) δ 189.1, 189.0, 167.5, 167.1, 158.1, 158.0, 149.6, 149.6, 147.5, 144.0, 143.1, 130.2, 130.1, 129.9 (t, *J* = 292.5 Hz), 128.8 (t, *J* = 292.9 Hz), 113.5, 113.5, 113.4, 113.1, 112.9, 112.8, 112.7, 110.5, 110.4, 105.0, 104.9, 104.8, 101.0, 88.3, 88.0, 72.4, 72.3, 66.4, 66.4, 56.4, 56.0, 44.7, 44.3 (t, *J* = 23.4 Hz), 44.0 (t, *J* = 23.3 Hz), 34.6, 34.0, 31.4, 29.9, 29.2, 17.2, 15.8, 14.3. HRMS-EI (m/z): Calcd for $C_{24}H_{23}ClF_2O_6$ [M] 480.1146, found 480.1136.

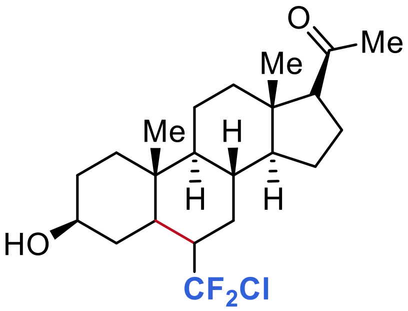

1-((3S,8R,9S,10R,13S,14S,17R)-6-(chlorodifluoromethyl)-3-hydroxy-10,13-dimethylhexadecahydro-1H-cyclopenta[a]phenanthren-17-yl)ethan-1-one (**1ad**)

Following the general procedure **A**, the title compound (72.3 mg, white solid, d.r. = 1/1, m.p. 163.0-164.6 ^oC) was obtained in 90% yield.

^{1}H NMR (400 MHz, $CDCl_3$) δ 3.45 (ddt, *J* = 14.8, 10.9, 5.1 Hz, 1H), 2.55 – 2.34 (m, 2H), 2.17 – 2.07 (m, 2H), 2.05 (s, 3H), 2.00 – 1.90 (m, 1H), 1.82 – 1.69 (m, 4H), 1.67 – 1.46 (m, 6H), 1.39 – 1.27 (m, 3H), 1.24 – 1.14 (m, 2H), 1.04 (dddd, *J* = 12.4, 10.7, 6.9, 1.5 Hz, 1H), 0.90 (td, *J* = 13.7, 12.8, 3.9 Hz, 1H), 0.83 (d, *J* = 3.6 Hz, 3H), 0.64

(dd, J = 11.1, 4.1 Hz, 1H), 0.57 (s, 3H). **^{19}F NMR** (377 MHz, $CDCl_3$) δ -44.02 (d, J = 154.6 Hz), -46.12 (ddt, J = 154.5, 29.7, 3.8 Hz). **^{13}C NMR** (126 MHz, $CDCl_3$) δ 209.6, 133.6 (dd, J = 303.0, 301.4 Hz), 72.1, 63.8, 56.5, 55.0, 49.9, 49.7, 49.7, 49.6, 46.9, 44.3, 40.0, 39.0, 37.5, 37.5, 35.1, 33.1, 33.1, 32.1, 32.1, 31.6, 31.5, 24.5, 22.9, 21.2, 14.9, 14.8, 13.6. HRMS-EI (m/z): Calcd for $C_{22}H_{33}ClF_2O_2^+$ $[M]^+$ 402.2132, found 402.2136.

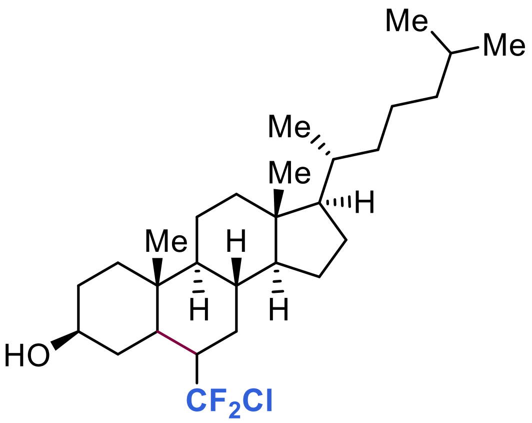

(3S,8S,9S,10R,13R,14S,17R)-6-(chlorodifluoromethyl)-10,13-dimethyl-17-((R)-6-methylheptan-2-yl)hexadecahydro-1H-cyclopenta[a]phenanthren-3-ol (**1ae**)
Following the general procedure **A**, the title compound (66.1 mg, d.r = 1/1, colourless liquid) was obtained in 70% yield.
^{1}H NMR (400 MHz, $CDCl_3$) δ 3.50 (dq, J = 10.6, 6.7, 5.1 Hz, 1H), 2.59 – 2.40 (m, 1H), 2.19 – 2.10 (m, 1H), 1.99 (dt, J = 12.6, 3.5 Hz, 1H), 1.92 – 1.75 (m, 5H), 1.70 – 1.44 (m, 7H), 1.42 – 1.20 (m, 7H), 1.19 – 1.04 (m, 7H), 1.00 – 0.82 (m, 14H), 0.68 (s, 3H). **^{19}F NMR** (377 MHz, $CDCl_3$) δ -43.77 (d, J = 153.9 Hz), -45.96 (ddd, J = 153.4, 30.2, 3.9 Hz). **^{13}C NMR** (126 MHz, $CDCl_3$) δ 133.9 (dd, J = 303.7, 300.6 Hz), 72.3, 72.2, 56.4, 56.4, 55.2, 50.1, 49.9, 49.9, 49.7, 46.9, 42.8, 40.0, 40.0, 39.7, 37.6, 37.6, 36.3, 35.9, 35.1, 33.2, 33.2, 32.1, 32.1, 31.7, 28.3, 28.2, 24.3, 24.0, 23.0, 22.7, 21.3, 18.8, 14.9, 14.8, 12.3. HRMS-EI (m/z): Calcd for $C_{28}H_{47}ClF_2O^+$ $[M]^+$ 472.3278, found 472.3286.

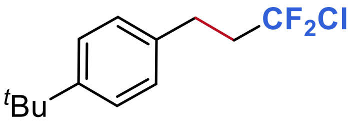

1-(tert-butyl)-4-(3-chloro-3,3-difluoropropyl)benzene (**1af**)
Following the general procedure **B**, the title compound (34.5 mg, colourless liquid) was obtained in 70% yield (5 mmol scale, 65% yield, 0.80 g).
^{1}H NMR (400 MHz, $CDCl_3$) δ 7.39 – 7.34 (m, 2H), 7.19 – 7.14 (m, 2H), 2.95 – 2.89 (m, 2H), 2.67 – 2.55 (m, 2H), 1.34 (s, 9H). **^{19}F NMR** (377 MHz, $CDCl_3$) δ -51.23 (td, J = 12.9, 2.8 Hz). **^{13}C NMR** (126 MHz, Chloroform-*d*) δ 149.6, 135.8, 129.4 (t, J = 291.8 Hz), 128.0, 125.6, 43.6 (t, J = 23.7 Hz), 34.4, 31.3, 28.9 (t, J = 3.3 Hz). **^{13}C NMR** (126 MHz, $CDCl_3$) δ 149.7, 136.0, 129.6 (t, J = 291.8 Hz), 128.1, 125.8, 43.8 (t, J = 23.8 Hz), 34.6, 31.5, 29.1 (t, J = 3.3 Hz). HRMS-EI (m/z): Calcd for $C_{13}H_{11}ClF_2^+$

[M]$^+$ 246.0981, found 246.0977.

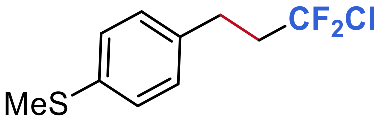

(4-(3-chloro-3,3-difluoropropyl)phenyl)(methyl)sulfane (**1ag**)

Following the general procedure **B**, the title compound (29.2 mg, colourless liquid) was obtained in 62% yield (5 mmol scale, 60% yield, 0.71 g).

^{1}H NMR (400 MHz, $CDCl_3$) δ 7.25 – 7.16 (m, 2H), 7.16 – 7.08 (m, 2H), 2.99 – 2.85 (m, 2H), 2.68 – 2.52 (m, 2H), 2.48 (s, 3H). **^{19}F NMR** (377 MHz, $CDCl_3$) δ -51.17 (td, J = 12.6, 2.7 Hz). **^{13}C NMR** (101 MHz, $CDCl_3$) δ 136.7, 135.9, 129.4 (t, J = 292.2 Hz), 128.9, 127.3, 43.7 (t, J = 23.9 Hz), 29.1 (t, J = 3.3 Hz), 16.2. HRMS-EI (m/z): Calcd for $C_{10}H_{11}ClF_2S^+$ [M]$^+$ 236.0233, found 236.0236.

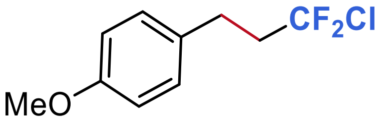

1-(3-chloro-3,3-difluoropropyl)-4-methoxybenzene (**1ah**)

Following the general procedure **B**, the title compound (25.5 mg, colourless liquid) was obtained in 68% yield.

^{1}H NMR (500 MHz, $CDCl_3$) δ 7.15 – 7.11 (m, 2H), 6.90 – 6.83 (m, 2H), 3.80 (s, 3H), 2.91 – 2.85 (m, 2H), 2.62 – 2.52 (m, 2H). **^{19}F NMR** (377 MHz, $CDCl_3$) δ -51.16 (td, J = 12.9, 2.7 Hz). **^{13}C NMR** (126 MHz, $CDCl_3$) δ 158.5, 131.0, 129.5 (t, J = 292.2 Hz), 129.4, 114.3, 55.4, 44.0 (t, J = 23.7 Hz), 28.8 (t, J = 3.5 Hz). HRMS-EI (m/z): Calcd for $C_{10}H_{11}ClF_2O^+$ [M]$^+$ 220.0461, found 220.0460.

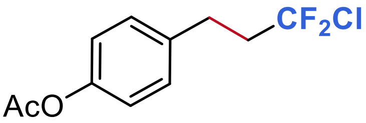

4-(3-chloro-3,3-difluoropropyl)phenyl acetate (**1ai**)

Following the general procedure **B**, the title compound (33.6 mg, colourless liquid) was obtained in 68% yield.

^{1}H NMR (400 MHz, $CDCl_3$) δ 7.25 – 7.17 (m, 2H), 7.07 – 6.99 (m, 2H), 2.96 – 2.88 (m, 2H), 2.68 – 2.51 (m, 2H), 2.30 (s, 3H). **^{19}F NMR** (377 MHz, $CDCl_3$) δ -51.30 (td, J = 12.6, 2.7 Hz). **^{13}C NMR** (126 MHz, $CDCl_3$) δ 169.7, 149.5, 136.6, 129.4, 129.3 (t, J = 294.2 Hz), 122.0, 43.7 (t, J = 23.9 Hz), 29.1 (t, J = 3.2 Hz), 21.3. HRMS-EI (m/z): Calcd for $C_{11}H_{11}ClF_2O_2^+$ [M]$^+$ 248.0140, found 248.0410.

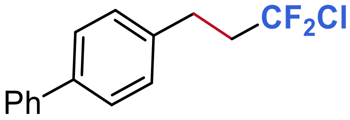

4-(3-chloro-3,3-difluoropropyl)-1,1'-biphenyl (**1aj**)

Following the general procedure **B**, the title compound (31.9 mg, white solid, m.p. 63.5-64.2 ^{o}C) was obtained in 60% yield (5 mmol scale, 57% yield, 0.76 g).

^{1}**H NMR** (400 MHz, $CDCl_3$) δ 7.72 – 7.58 (m, 4H), 7.58 – 7.48 (m, 2H), 7.48 – 7.40 (m, 1H), 7.35 (d, *J* = 8.3 Hz, 2H), 3.11 – 3.02 (m, 2H), 2.81 – 2.63 (m, 2H). ^{19}**F NMR** (377 MHz, $CDCl_3$) δ -51.00 (td, *J* = 12.6, 2.7 Hz). ^{13}**C NMR** (101 MHz, $CDCl_3$) δ 140.9, 139.8, 138.0, 129.5 (t, *J* = 292.2 Hz), 128.9, 128.9, 127.5, 127.4, 127.1, 43.7 (t, *J* = 23.9 Hz), 29.3 (t, *J* = 3.4 Hz). HRMS-EI (m/z): Calcd for $C_{15}H_{13}ClF_2^+$ $[M]^+$ 266.0668, found 266.0670.

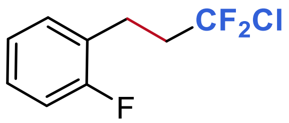

1-(3-chloro-3,3-difluoropropyl)-2-fluorobenzene (**1ak**)

Following the general procedure **B**, the title compound (24.1 mg, colourless liquid) was obtained in 58% yield.

^{1}**H NMR** (400 MHz, $CDCl_3$) δ 7.29 – 7.17 (m, 2H), 7.14 – 7.00 (m, 2H), 3.13 – 2.80 (m, 2H), 2.74 – 2.48 (m, 2H). ^{19}**F NMR** (377 MHz, $CDCl_3$) δ -51.44 (td, *J* = 12.6, 2.7 Hz), -118.49 –118.55 (m). ^{13}**C NMR** (126 MHz, $CDCl_3$) δ 161.3 (d, *J* = 245.7 Hz), 130.7 (d, *J* = 4.6 Hz), 129.4 (t, *J* = 292.1 Hz)， 128.7 (d, *J* = 8.1 Hz), 126.0 (d, *J* = 15.6 Hz), 124.4 (d, *J* = 3.7 Hz), 115.7 (d, *J* = 21.8 Hz), 42.2 (t, *J* = 24.1 Hz), 23.6 – 23.5 (m). HRMS-EI (m/z): Calcd for $C_9H_8ClF_3^+$ $[M]^+$ 208.0261, found 208.0262.

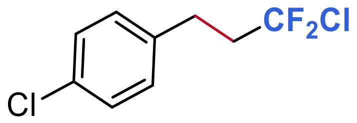

1-chloro-4-(3-chloro-3,3-difluoropropyl)benzene (**1al**)

Following the general procedure **B**, the title compound (22.4 mg, colourless liquid) was obtained in 50% yield.

^{1}**H NMR** (400 MHz, $CDCl_3$) δ 7.31 – 7.27 (m, 2H), 7.16 – 7.12 (m, 2H), 2.94 – 2.88 (m, 2H), 2.64 – 2.51 (m, 2H). ^{19}**F NMR** (377 MHz, $CDCl_3$) δ -51.26 (td, *J* = 12.5, 2.7 Hz). ^{13}**C NMR** (126 MHz, $CDCl_3$) δ 137.4, 132.6, 129.8, 129.3 (t, *J* = 292.4 Hz), 129.0, 43.6 (t, *J* = 24.0 Hz), 29.1 (t, *J* = 3.4 Hz). HRMS-EI (m/z): Calcd for $C_9H_8Cl_2F_2^+$ $[M]^+$ 223.9966, found 223.9967.

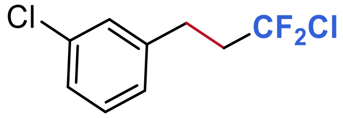

1-chloro-3-(3-chloro-3,3-difluoropropyl)benzene (**1am**)

Following the general procedure **B**, the title compound (24.6 mg, colourless liquid) was obtained in 55% yield.

^{1}**H NMR** (400 MHz, $CDCl_3$) δ 7.29 – 7.18 (m, 3H), 7.10 (dt, *J* = 6.9, 1.8 Hz, 1H), 3.01 – 2.83 (m, 2H), 2.70 – 2.48 (m, 2H). ^{19}**F NMR** (377 MHz, $CDCl_3$) δ -51.34 (td, *J* = 12.6, 2.7 Hz). ^{13}**C NMR** (126 MHz, $CDCl_3$) δ 141.0, 134.6, 130.1, 129.22 (t, *J* = 291.6 Hz), 128.6, 127.1, 126.7, 43.4 (t, *J* = 24.2 Hz), 29.4 (t, *J* = 3.4 Hz). HRMS-EI (m/z): Calcd for $C_9H_8Cl_2F_2^+$ $[M]^+$ 223.9966, found 223.9969.

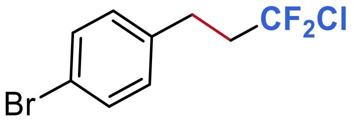

1-bromo-4-(3-chloro-3,3-difluoropropyl)benzene (**1an**)

Following the general procedure **B**, the title compound (24.1 mg, colourless liquid) was obtained in 55% yield.

^{1}H NMR (400 MHz, $CDCl_3$) δ 7.74 – 7.35 (m, 2H), 7.16 – 6.95 (m, 2H), 3.26 – 2.72 (m, 2H), 2.76 – 2.33 (m, 2H). **^{19}F NMR** (377 MHz, $CDCl_3$) δ -51.27 (td, *J* = 12.6, 2.7 Hz). **^{13}C NMR** (126 MHz, $CDCl_3$) δ 138.0, 132.0, 130.2, 129.3 (t, *J* = 292.1 Hz), 120.7, 43.5 (t, *J* = 24.1 Hz), 29.1 (t, *J* = 3.6 Hz). HRMS-EI (m/z): Calcd for $C_9H_8BrClF_2^+$ $[M]^+$ 267.9460, found 267.9462.

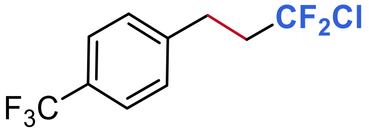

1-(3-chloro-3,3-difluoropropyl)-4-(trifluoromethyl)benzene (**1ao**)

Following the general procedure **B**, the title compound (27.9 mg, colourless liquid) was obtained in 54% yield.

^{1}H NMR (400 MHz, $CDCl_3$) δ 7.59 (d, *J* = 8.1 Hz, 2H), 7.43 – 7.30 (m, 2H), 3.17 – 2.89 (m, 2H), 2.77 – 2.50 (m, 2H). **^{19}F NMR** (377 MHz, $CDCl_3$) δ -51.39 (td, *J* = 12.6, 2.9 Hz), -62.54. **^{13}C NMR** (126 MHz, $CDCl_3$) δ 143.1, 129.3 (q, *J* = 32.6 Hz), 129.2 (t, *J* = 292.0 Hz), 128.9, 125.8 (q, *J* = 3.4, 2.9 Hz), 124.3 (q, *J* = 272.0 Hz), 43.3 (t, *J* = 24.3 Hz), 29.5 (t, *J* = 3.4 Hz). HRMS-EI (m/z): Calcd for $C_{10}H_8ClF_5^+$ $[M]^+$ 258.0229, found 258.0235.

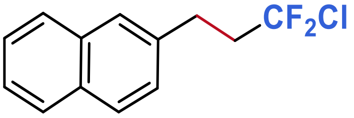

2-(3-chloro-3,3-difluoropropyl)naphthalene (**1ap**)

Following the general procedure **B**, the title compound (19.2 mg, colourless liquid) was obtained in 40% yield.

^{1}H NMR (400 MHz, $CDCl_3$) δ 7.86 – 7.79 (m, 3H), 7.67 (s, 1H), 7.57 – 7.43 (m, 2H), 7.34 (dd, *J* = 8.5, 1.8 Hz, 1H), 3.20 – 3.04 (m, 2H), 2.82 – 2.60 (m, 2H). **^{19}F NMR** (377 MHz, $CDCl_3$) δ -51.10 (td, *J* = 12.6, 2.7 Hz). **^{13}C NMR** (126 MHz, $CDCl_3$) δ 136.5, 133.7, 132.4, 129.5 (t, *J* = 292.0 Hz), 128.6, 127.8, 127.6, 126.8, 126.8, 126.4, 125.8, 43.7 (t, *J* = 23.9 Hz), 29.8 (t, *J* = 3.5 Hz). HRMS-EI (m/z): Calcd for $C_{13}H_{11}ClF_2^+$ $[M]^+$ 240.0512, found 240.0515.

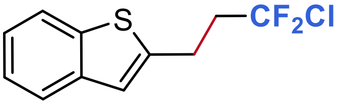

2-(3-chloro-3,3-difluoropropyl)benzo[b]thiophene (**1aq**)

Following the general procedure **B**, the title compound (19.7 mg, white solid, m.p.

71.1-72.0 °C) was obtained in 40% yield.

^{1}H NMR (400 MHz, $CDCl_3$) δ 7.93 – 7.54 (m, 2H), 7.42 – 7.24 (m, 2H), 7.08 (q, *J* = 1.0 Hz, 1H), 3.32 – 3.17 (m, 2H), 2.84 – 2.69 (m, 2H). **^{19}F NMR** (377 MHz, $CDCl_3$) δ -51.37 (td, *J* = 12.6, 2.7 Hz). **^{13}C NMR** (126 MHz, $CDCl_3$) δ 142.2, 140.0, 139.5, 129.0 (t, *J* = 292.1 Hz), 124.5, 124.2, 123.2, 122.3, 121.7, 43.2 (t, *J* = 24.4 Hz), 24.9 (t, *J* = 3.7 Hz). HRMS-EI (m/z): Calcd for $C_{11}H_9ClF_2S^+$ $[M]^+$ 246.0076, found 246.0079.

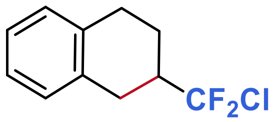

2-(chlorodifluoromethyl)-1,2,3,4-tetrahydronaphthalene (**1ar**)

Following the general procedure **B**, the title compound (20.7 mg, colourless liquid) was obtained in 48% yield.

^{1}H NMR (400 MHz, $CDCl_3$) δ 7.17 – 7.04 (m, 4H), 3.05 (ddd, *J* = 15.8, 5.1, 1.9 Hz, 1H), 2.99 – 2.79 (m, 3H), 2.67 – 2.50 (m, 1H), 2.24 (ddt, *J* = 10.3, 5.2, 2.6 Hz, 1H), 1.72 (dtd, *J* = 13.0, 11.9, 5.8 Hz, 1H). **^{19}F NMR** (377 MHz, $CDCl_3$) δ -56.39 (ddd, *J* = 162.3, 9.2, 2.7 Hz), -56.86 – -57.48 (m). **^{13}C NMR** (126 MHz, $CDCl_3$) δ 135.6, 133.9, 129.3, 128.9, 126.4, 126.2, 46.0 (t, *J* = 22.8 Hz), 29.6 (t, *J* = 3.2 Hz), 28.6, 23.6 (t, *J* = 3.0 Hz). HRMS-EI (m/z): Calcd for $C_{11}H_{11}ClF_2^+$ $[M]^+$ 216.0512, found 216.0511.

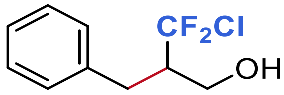

2-benzyl-3-chloro-3,3-difluoropropan-1-ol (**1as**)

Following the general procedure **B**, the title compound (20.2 mg, colourless liquid) was obtained in 46% yield.

^{1}H NMR (400 MHz, $CDCl_3$) δ 7.32 – 7.26 (m, 2H), 7.24 – 7.18 (m, 3H), 3.81 (dd, *J* = 12.2, 3.5 Hz, 1H), 3.69 (dd, *J* = 12.2, 5.2 Hz, 1H), 3.06 (dd, *J* = 13.9, 3.9 Hz, 1H), 2.80 (dd, *J* = 13.9, 10.3 Hz, 1H), 2.59 (qdt, *J* = 10.4, 5.2, 3.8 Hz, 1H). **^{19}F NMR** (377 MHz, $CDCl_3$) δ -52.39 (ddd, *J* = 19.3, 10.3, 2.8 Hz). **^{13}C NMR** (126 MHz, $CDCl_3$) δ 137.9, 131.6 (t, *J* = 295.5 Hz), 129.3, 128.9, 127.0, 60.0 (t, *J* = 3.1 Hz), 53.8 (t, *J* = 19.6 Hz), 32.1 (t, *J* = 3.0 Hz). HRMS-EI (m/z): Calcd for $C_{10}H_{11}ClF_2O^+$ $[M]^+$ 220.0461, found 220.0466.

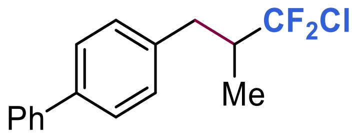

4-(3-chloro-3,3-difluoro-2-methylpropyl)-1,1'-biphenyl (**1at**)

Following the general procedure **B**, the title compound (39.0 mg, white solid, m.p. 60.0-60.8 °C) was obtained in 70% yield.

^{1}H NMR (400 MHz, $CDCl_3$) δ 7.53 – 7.42 (m, 4H), 7.38 – 7.31 (m, 2H), 7.31 – 7.22 (m, 1H), 7.20 – 7.15 (m, 2H), 3.14 (dd, *J* = 13.1, 2.9 Hz, 1H), 2.56 – 2.45 (m, 1H), 2.41 (dd, *J* = 13.0, 10.7 Hz, 1H), 1.02 (d, *J* = 6.6 Hz, 3H). **^{19}F NMR** (377 MHz,

$CDCl_3$) δ -55.17 – -56.55 (m). **^{13}C NMR** (126 MHz, $CDCl_3$) δ 140.9, 139.7, 137.4, 132.8 (t, *J* = 294.5 Hz), 129.7, 128.9, 127.4, 127.4, 127.2, 46.7 (t, *J* = 21.6 Hz), 36.6 (t, *J* = 3.1 Hz), 13.6 (t, *J* = 3.3 Hz). HRMS-EI (m/z): Calcd for $C_{16}H_{15}ClF_2^+$ $[M]^+$ 280.0825, found 280.0830.

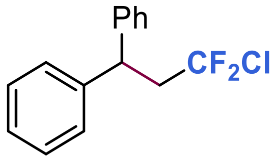

(3-Chloro-3,3-difluoropropane-1,1-diyl)dibenzene (**1au**)

Following the general procedure **B**, the title compound (23.4 mg, colourless liquid) was obtained in 44% yield.

^{1}H NMR (400 MHz, $CDCl_3$) δ 7.35 – 7.26 (m, 8H), 7.25 – 7.20 (m, 2H), 4.43 (t, *J* = 7.0 Hz, 1H), 3.16 (td, *J* = 12.9, 7.0 Hz, 2H) ppm. **^{19}F NMR** (377 MHz, $CDCl_3$) δ -48.50 (td, *J* = 13.0, 2.7 Hz). **^{13}C NMR** (126 MHz, $CDCl_3$) δ 143.0, 129.4 (t, *J* = 294.0 Hz), 128.9, 127.7, 126.9, 47.5 (t, *J* = 22.4 Hz), 46.5 (t, *J* = 2.0 Hz). HRMS-EI (m/z): Calcd for $C_{15}H_{13}ClF_2^+$ $[M]^+$ 266.0668, found 266.0670.

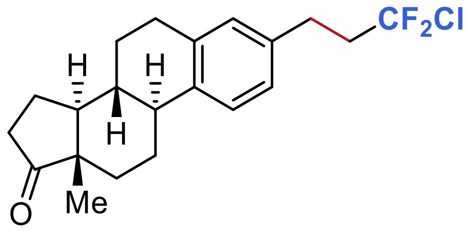

(8S,9R,13R,14R)-3-(3-chloro-3,3-difluoropropyl)-13-methyl-6,7,8,9,11,12,13,14,15,16-decahydro-17H-cyclopenta[a]phenanthren-17-one (**1av**)

Following the general procedure **B**, the title compound (40.3 mg, white solid, m.p. = 109.5-111.0 °C) was obtained in 55% yield. **^{1}H NMR** (400 MHz, $CDCl_3$) δ 7.29 – 7.21 (m, 1H), 7.00 (dd, *J* = 8.1, 2.0 Hz, 1H), 6.95 (d, *J* = 2.0 Hz, 1H), 2.88 (ddd, *J* = 17.7, 8.8, 4.3 Hz, 4H), 2.65 – 2.36 (m, 4H), 2.30 (dt, *J* = 13.1, 6.5 Hz, 1H), 2.21 – 1.89 (m, 4H), 1.68 – 1.43 (m, 6H), 0.91 (s, 3H). **^{19}F NMR** (376 MHz, CDCl3) δ -51.23 (td, J = 12.8, 2.8 Hz). **^{13}C NMR** (151 MHz, $CDCl_3$) δ 220.9, 138.2, 137.0, 136.4, 129.5 (t, *J* = 292.3 Hz), 129.1, 125.8, 125.8, 50.6, 48.1, 44.4, 43.7 (t, *J* = 23.7 Hz), 38.3, 35.9, 31.7, 29.5, 29.1 (t, *J* = 3.4 Hz), 26.6, 25.8, 21.7, 13.9. $C_{21}H_{26}OF_2Cl^+$ $[M]^+$ 367.1635, found 367.1639.

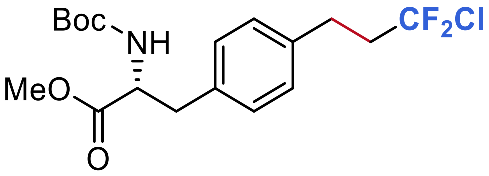

methyl (R)-2-((tert-butoxycarbonyl)amino)-3-(4-(3-chloro-3,3-difluoropropyl)phenyl)propanoate (**1aw**)

Following the general procedure **B**, the title compound (31.3 mg, colorless liquid) was obtained in 40% yield. **^{1}H NMR** (400 MHz, $CDCl_3$) δ 7.18 – 6.94 (m, 4H), 5.01 (d, *J* = 8.4 Hz, 1H), 4.56 (dt, *J* = 8.4, 5.9 Hz, 1H), 3.70 (s, 3H), 3.18 – 2.95 (m, 2H), 2.94 – 2.82 (m, 2H), 2.66 – 2.40 (m, 2H), 1.41 (s, 9H). **^{19}F NMR** (376 MHz, $CDCl_3$) δ -51.24 (td, *J* = 12.8, 2.8 Hz). **^{13}C NMR** (101 MHz, $CDCl_3$) δ 172.4, 155.1, 137.6, 134.5, 129.7, 129.4 (t, *J* = 292.1 Hz), 128.5, 80.0, 54.5, 52.3, 43.6 (t, *J* = 23.8 Hz), 38.0, 29.2 (t, *J* = 3.3 Hz), 28.4. HRMS-EI (m/z): Calcd for $C_{18}H_{25}NO_4F_2Cl^+$ $[M]^+$ 392.1440, found 392.1451.

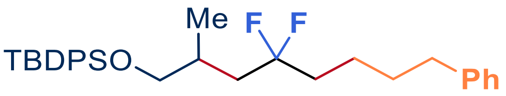

tert-butyl((4,4-difluoro-2-methyl-8-phenyloctyl)oxy)diphenylsilane (**2a**)
Following the general procedure **C**, the title compound (44.5 mg, colourless liquid) was obtained in 90% yield.
^{1}H NMR (400 MHz, $CDCl_3$) δ 7.70 – 7.63 (m, 4H), 7.48 – 7.34 (m, 6H), 7.33 – 7.24 (m, 2H), 7.24 – 7.15 (m, 3H), 3.57 – 3.41 (m, 2H), 2.65 – 2.61 (m, 2H), 2.20 – 2.08 (m, 1H), 2.00 (ddd, *J* = 10.9, 8.6, 5.4 Hz, 1H), 1.90 – 1.75 (m, 2H), 1.71 – 1.49 (m, 5H), 1.07 (s, 9H), 1.01 (d, *J* = 6.7 Hz, 3H). **^{19}F NMR** (377 MHz, $CDCl_3$) δ -94.30 (ddtd, *J* = 240.2, 22.3, 17.0, 11.4 Hz), -96.07 (ddq, *J* = 240.2, 21.9, 15.9 Hz). **^{13}C NMR** (126 MHz, $CDCl_3$) δ 142.3, 135.7, 133.9, 129.8, 128.5, 128.5, 127.8, 125.9, 125.8 (t, *J* = 240.8 Hz), 68.8, 39.3 (t, *J* = 24.5 Hz), 37.0 (t, *J* = 25.5 Hz), 35.9, 31.4, 31.0 (t, *J* = 2.7 Hz), 27.0, 22.3 (t, *J* = 4.6 Hz), 19.5, 17.8. HRMS-EI (m/z): Calcd for $C_{31}H_{40}F_2OSi^+$ $[M]^+$ 494.2811, found 494.2816.

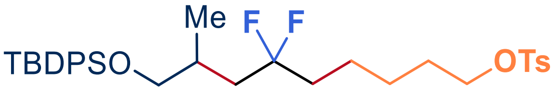

9-((tert-butyldiphenylsilyl)oxy)-6,6-difluoro-8-methylnonyl 4-methylbenzenesulfonate (**2b**)
Following the general procedure **C**, the title compound (30.7 mg, colourless liquid) was obtained in 51% yield.
^{1}H NMR (400 MHz, $CDCl_3$) δ 7.83 – 7.74 (m, 2H), 7.69 – 7.59 (m, 4H), 7.46 – 7.29 (m, 8H), 4.02 (t, *J* = 6.4 Hz, 2H), 3.56 – 3.40 (m, 2H), 2.44 (s, 3H), 2.16 – 2.05 (m, 1H), 1.96 (td, *J* = 13.7, 12.4, 7.4 Hz, 1H), 1.81 – 1.59 (m, 4H), 1.47 – 1.28 (m, 5H), 1.05 (s, 9H), 0.99 (d, *J* = 6.7 Hz, 3H). **^{19}F NMR** (377 MHz, $CDCl_3$) δ -94.75 (ddtd, *J* = 240.7, 22.2, 17.3, 11.5 Hz), -96.50 (ddq, *J* = 240.1, 22.3, 16.1 Hz). **^{13}C NMR** (126 MHz, $CDCl_3$) δ 144.9, 135.7, 133.9, 133.3, 130.0, 129.8, 128.0, 127.8, 125.6 (t, *J* = 240.6 Hz), 70.4, 68.7, 39.4 (t, *J* = 24.5 Hz), 36.9 (t, *J* = 25.6 Hz), 31.0 (t, *J* = 3.0 Hz), 28.8, 27.0, 25.3, 21.9 (t, *J* = 4.7 Hz), 21.8, 19.4, 17.7. HRMS-ESI (m/z): Calcd for $C_{33}H_{44}F_2O_4SSiNa^+$ $[M+Na]^+$ 625.2590, found 625.2592.

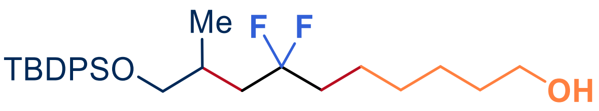

10-((tert-butyldiphenylsilyl)oxy)-7,7-difluoro-9-methyldecan-1-ol (**2c**)

Following the general procedure **C**, the title compound (38.3 mg, colourless liquid) was obtained in 83% yield.

^{1}H NMR (400 MHz, $CDCl_3$) δ 7.70 – 7.65 (m, 4H), 7.50 – 7.35 (m, 6H), 3.65 (t, *J* = 6.6 Hz, 2H), 3.57 – 3.40 (m, 2H), 2.22 – 2.09 (m, 1H), 2.00 (tdd, *J* = 10.9, 5.4, 3.0 Hz, 1H), 1.89 – 1.73 (m, 2H), 1.65 – 1.54 (m, 3H), 1.54 – 1.45 (m, 2H), 1.41 – 1.34 (m, 4H), 1.07 (s, 9H), 1.01 (d, *J* = 6.7 Hz, 3H). **^{19}F NMR** (377 MHz, $CDCl_3$) δ -94.32 (ddtd, *J* = 240.0, 22.2, 17.1, 11.4 Hz), -96.02 (ddq, *J* = 240.2, 21.9, 16.0 Hz). **^{13}C NMR** (126 MHz, $CDCl_3$) δ 135.7, 133.9, 129.7, 127.8, 125.8 (t, *J* = 240.8 Hz), 68.8, 63.0, 39.3 (t, *J* = 24.6 Hz), 37.1 (t, *J* = 25.4 Hz), 32.7, 31.0 (t, *J* = 3.2 Hz), 29.3, 27.0, 25.7, 22.5 (t, *J* = 4.7 Hz), 19.4, 17.7. HRMS-EI (m/z): Calcd for $C_{27}H_{40}F_2O_2SiNa^+$ $[M+Na]^+$ 485.2658, found 485.2661.

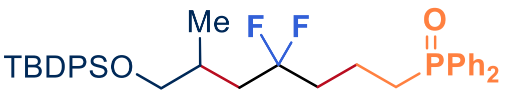

(7-((tert-butyldiphenylsilyl)oxy)-4,4-difluoro-6-methylheptyl)diphenylphosphine oxide (**2d**)

Following the general procedure **C**, the title compound (46.6 mg, colourless liquid) was obtained in 77% yield.

^{1}H NMR (400 MHz, $CDCl_3$) δ 7.74 (ddq, *J* = 11.4, 6.6, 1.3 Hz, 4H), 7.67 – 7.60 (m, 4H), 7.55 – 7.34 (m, 12H), 3.51 – 3.42 (m, 2H), 2.37 – 2.23 (m, 2H), 2.05 – 1.76 (m, 6H), 1.67 – 1.50 (m, 1H), 1.04 (s, 9H), 0.97 (d, *J* = 6.7 Hz, 3H). **^{19}F NMR** (377 MHz, $CDCl_3$) δ -95.00 – -96.07 (m), -96.28 – -97.50 (m). **^{31}P NMR** (162 MHz, $CDCl_3$) δ 31.92 (dp, *J* = 21.3, 10.1 Hz). **^{13}C NMR** (126 MHz, $CDCl_3$) δ 135.7, 133.8 (d, *J* = 3.3 Hz), 132.9 (dd, *J* = 98.3, 1.7 Hz), 131.9 (d, *J* = 2.8 Hz), 130.9 (d, *J* = 9.2 Hz), 129.7, 128.8 (d, *J* = 11.6 Hz), 127.7, 125.2 (t, *J* = 241.8 Hz), 68.62, 39.52 (t, *J* = 24.3 Hz), 37.92 (td, *J* = 25.7, 13.7 Hz), 30.79 (t, *J* = 2.7 Hz), 29.54 (d, *J* = 72.0 Hz), 26.97, 19.39, 17.8, 15.1 (td, *J* = 4.5, 3.7 Hz). HRMS-ESI (m/z): Calcd for $C_{36}H_{44}F_2O_2PSi^+$ $[M+H]^+$ 605.2811, found 605.2812.

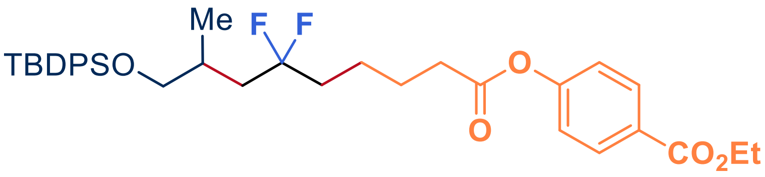

ethyl 4-((9-((tert-butyldiphenylsilyl)oxy)-6,6-difluoro-8-methylnonanoyl)oxy)benzoate (**2e**)

Following the general procedure **C**, the title compound (40.3 mg, colourless liquid) was obtained in 66% yield.

^{1}H NMR (400 MHz, $CDCl_3$) δ 8.11 – 8.03 (m, 2H), 7.69 – 7.62 (m, 4H), 7.47 – 7.34

(m, 6H), 7.20 – 7.11 (m, 2H), 4.38 (q, J = 7.1 Hz, 2H), 3.57 – 3.42 (m, 2H), 2.59 (t, J = 7.4 Hz, 2H), 2.13 – 1.95 (m, 2H), 1.92 – 1.73 (m, 4H), 1.66 – 1.57 (m, 3H), 1.39 (t, J = 7.1 Hz, 3H), 1.06 (s, 9H), 1.01 (d, J = 6.7 Hz, 3H). **^{19}F NMR** (377 MHz, $CDCl_3$) δ -94.27 – -95.39 (m), -96.59 (ddq, J = 240.7, 22.0, 15.8 Hz). **^{13}C NMR** (126 MHz, $CDCl_3$) δ 171.4, 166.0, 154.4, 135.7, 134.9, 133.9, 131.3, 129.8, 127.9 (t, J = 252.8 Hz), 127.8, 121.7, 68.8, 61.2, 39.5 (t, J = 24.5 Hz), 36.8 (t, J = 25.4 Hz), 34.3, 31.0 (t, J = 3.0 Hz), 27.0, 24.7, 22.0 (t, J = 4.2 Hz), 19.5, 17.8, 14.5. HRMS-ESI (m/z): Calcd for $C_{35}H_{44}F_2O_5SiNa^+$ $[M+Na]^+$ 633.2818, found 633.2818.

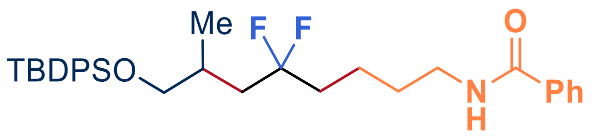

N-(8-((tert-butyldiphenylsilyl)oxy)-5,5-difluoro-7-methyloctyl)benzamide (**2f**)
Following the general procedure **C**, the title compound (46.2 mg, colourless liquid) was obtained in 86% yield.
^{1}H NMR (400 MHz, $CDCl_3$) δ 7.65 (ddd, J = 7.9, 1.6, 0.6 Hz, 4H), 7.53 – 7.27 (m, 9H), 7.14 – 7.05 (m, 2H), 3.60 – 3.38 (m, 2H), 2.35 (t, J = 7.5 Hz, 2H), 2.23 – 2.07 (m, 1H), 1.98 (dt, J = 12.4, 6.2 Hz, 1H), 1.90 – 1.70 (m, 4H), 1.66 – 1.60 (m, 1H), 1.57 – 1.51 (m, 2H), 1.05 (s, 9H), 0.99 (d, J = 6.7 Hz, 3H). **^{19}F NMR** (377 MHz, $CDCl_3$) δ -94.71 (ddtd, J = 240.3, 22.2, 17.0, 11.4 Hz), -96.45 (ddq, J = 240.5, 22.3, 16.1 Hz). **^{13}C NMR** (126 MHz, $CDCl_3$) δ 135.7, 133.9, 129.8, 129.2, 127.8, 125.6 (t, J = 241.1 Hz), 124.4, 119.9, 68.8, 39.4 (t, J = 24.6 Hz), 37.6, 36.9 (t, J = 25.6 Hz), 31.0 (t, J = 3.6 Hz), 27.0, 25.3, 22.2 (t, J = 4.7 Hz), 19.5, 17.8. HRMS-ESI (m/z): Calcd for $C_{32}H_{41}F_2NO_2SiNa^+$ $[M+Na]^+$ 560.2767, found 560.2767.

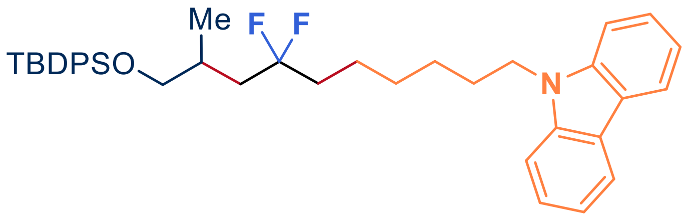

9-(10-((tert-butyldiphenylsilyl)oxy)-7,7-difluoro-9-methyldecyl)-9H-carbazole (**2g**)
Following the general procedure **C**, the title compound (36.7 mg, colourless liquid) was obtained in 60% yield.
^{1}H NMR (400 MHz, $CDCl_3$) δ 8.13 (dt, J = 7.8, 1.0 Hz, 2H), 7.71 – 7.60 (m, 4H), 7.53 – 7.35 (m, 10H), 7.25 (ddd, J = 8.0, 7.0, 1.1 Hz, 2H), 4.32 (t, J = 7.2 Hz, 2H), 3.65 – 3.30 (m, 2H), 2.19 – 1.96 (m, 2H), 1.90 (p, J = 7.2 Hz, 2H), 1.82 – 1.68 (m, 2H), 1.64 – 1.51 (m, 1H), 1.49 – 1.32 (m, 6H), 1.07 (s, 9H), 1.01 (d, J = 6.7 Hz, 3H). **^{19}F NMR** (377 MHz, $CDCl_3$) δ -94.31 (ddtd, J = 240.3, 22.2, 17.1, 11.2 Hz), -96.01 (ddq, J = 240.3, 21.8, 16.0 Hz). **^{13}C NMR** (126 MHz, $CDCl_3$) δ 140.5, 135.7, 133.9, 129.8, 127.8, 125.7, 123.9 (t, J = 242.0 Hz), 123.0, 120.5, 118.9, 108.7, 68.8, 43.1, 39.3 (t, J = 24.5 Hz), 37.0 (t, J = 25.5 Hz), 31.0 (t, J = 2.9 Hz), 29.4, 29.0, 27.3, 27.0, 22.4 (t, J = 4.6 Hz), 19.4, 17.7. HRMS-ESI (m/z): Calcd for $C_{39}H_{47}F_2NOSi^+$ $[M]^+$

611.3389, found 611.3388.

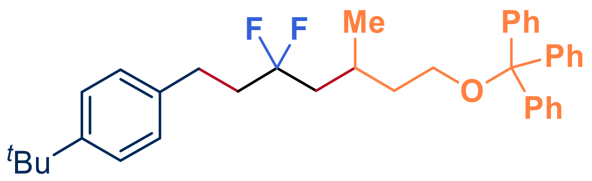

(((7-(4-(tert-butyl)phenyl)-5,5-difluoro-3-methylheptyl)oxy)methanetriyl)tribenzene (**2h**)

Following the general procedure **C**, the title compound (44.8 mg, colourless liquid) was obtained in 83% yield.

^{1}**H NMR** (400 MHz, $CDCl_3$) δ 7.47 – 7.41 (m, 6H), 7.34 – 7.28 (m, 6H), 7.27 – 7.19 (m, 5H), 7.14 – 7.09 (m, 2H), 3.18 – 3.03 (m, 2H), 2.83 – 2.71 (m, 2H), 2.20 – 1.97 (m, 3H), 1.95 – 1.59 (m, 3H), 1.55 – 1.43 (m, 1H), 1.33 (s, 9H), 0.92 (d, *J* = 6.7 Hz, 3H). ^{19}**F NMR** (377 MHz, $CDCl_3$) δ -94.73 (dtt, *J* = 241.1, 20.4, 13.3 Hz), -95.94 – -97.16 (m). ^{13}**C NMR** (126 MHz, $CDCl_3$) δ 149.1, 144.5, 137.8, 128.8, 128.1, 127.9, 127.0, 125.6, 125.2 (t, *J* = 241.0 Hz), 61.4, 43.2 (t, *J* = 23.9 Hz), 39.0 (t, *J* = 25.3 Hz), 37.7, 34.5, 31.5, 28.1 (t, *J* = 4.8 Hz), 25.4 (t, *J* = 3.4 Hz), 20.8. HRMS-EI (m/z): Calcd for $C_{37}H_{42}F_2O^+$ $[M]^+$ 540.3198, found 540.3196.

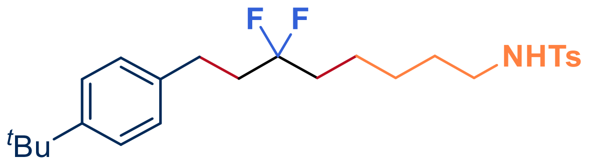

N-(9-(4-(tert-butyl)phenyl)-6,6-difluorononyl)-4-methylbenzenesulfonamide (**2i**)

Following the general procedure **C**, the title compound (22.6 mg, colourless liquid) was obtained in 50% yield.

^{1}**H NMR** (400 MHz, $CDCl_3$) δ 7.93 – 7.68 (m, 2H), 7.42 – 7.26 (m, 4H), 7.19 – 6.96 (m, 2H), 4.59 (t, *J* = 5.9 Hz, 1H), 2.93 (q, *J* = 6.9 Hz, 2H), 2.80 – 2.71 (m, 2H), 2.42 (s, 3H), 2.18 – 2.00 (m, 2H), 1.87 – 1.70 (m, 2H), 1.51 – 1.37 (m, 4H), 1.34 – 1.26 (m, 11H). ^{19}**F NMR** (377 MHz, $CDCl_3$) δ -98.80 (p, *J* = 16.5 Hz). ^{13}**C NMR** (126 MHz, $CDCl_3$) δ 149.2, 143.6, 137.7, 137.1, 129.9, 128.1, 127.2, 125.6, 124.7 (t, *J* = 240.8 Hz), 43.1, 38.4 (t, *J* = 25.4 Hz), 36.4 (t, *J* = 25.4 Hz), 31.5, 29.5, 28.0 (t, *J* = 4.7 Hz), 26.4, 22.0 (t, *J* = 4.4 Hz), 21.6. HRMS-EI (m/z): Calcd for $C_{25}H_{35}F_2NO_2S^+$ $[M]^+$ 451.2351, found 451.2358.

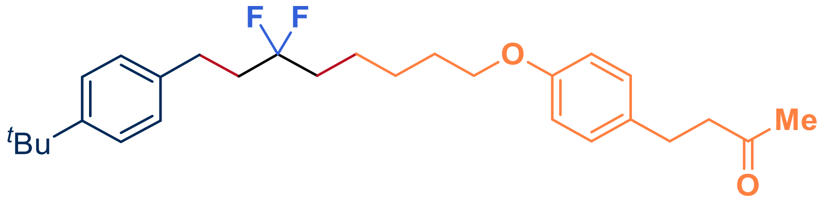

4-(4-((8-(4-(tert-butyl)phenyl)-6,6-difluorooctyl)oxy)phenyl)butan-2-one (**2j**)

Following the general procedure **C**, the title compound (32.4 mg, colourless liquid)

was obtained in 73% yield.

^{1}H NMR (400 MHz, $CDCl_3$) δ 7.37 – 7.29 (m, 2H), 7.18 – 7.04 (m, 4H), 6.88 – 6.75 (m, 2H), 3.93 (t, *J* = 6.4 Hz, 2H), 2.89 – 2.65 (m, 6H), 2.19 – 2.07 (m, 5H), 1.85 – 1.72 (m, 4H), 1.59 – 1.46 (m, 4H), 1.32 (s, 9H). **^{19}F NMR** (377 MHz, $CDCl_3$) δ -98.62 (p, *J* = 16.6 Hz). **^{13}C NMR** (126 MHz, $CDCl_3$) δ 208.3, 157.5, 149.2, 137.8, 133.1, 129.3, 128.1, 125.6, 124.85 (t, *J* = 240.6 Hz), 114.6, 67.8, 53.6, 45.6, 38.4 (t, *J* = 25.4 Hz), 36.59 (t, *J* = 25.4 Hz), 31.5, 30.2, 29.2, 29.0, 28.0 (t, *J* = 4.8 Hz), 26.1, 22.3 (t, *J* = 4.5 Hz). HRMS-EI (m/z): Calcd for $C_{28}H_{38}F_2O_2^+$ $[M]^+$ 444.2834, found 444.2829.

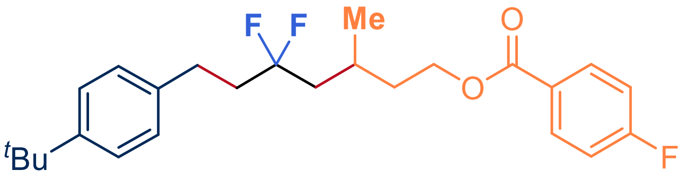

7-(4-(tert-butyl)phenyl)-5,5-difluoro-3-methylheptyl 4-fluorobenzoate (**2k**)

Following the general procedure **C**, the title compound (29.4 mg, colourless liquid) was obtained in 70% yield.

^{1}H NMR (400 MHz, $CDCl_3$) δ 8.19 – 7.90 (m, 2H), 7.35 – 7.28 (m, 2H), 7.14 – 7.04 (m, 4H), 4.36 (td, *J* = 7.2, 6.8, 1.4 Hz, 2H), 2.82 – 2.72 (m, 2H), 2.21 – 2.01 (m, 3H), 1.99 – 1.86 (m, 2H), 1.83 – 1.62 (m, 2H), 1.31 (s, 9H), 1.09 (d, *J* = 6.6 Hz, 3H). **^{19}F NMR** (377 MHz, $CDCl_3$) δ -96.39 (ddq, *J* = 48.7, 20.2, 15.9 Hz), -105.75 – -105.84 (m). **^{13}C NMR** (126 MHz, $CDCl_3$) δ 166.9, 165.3 (d, *J* = 113.9 Hz), 149.3, 137.6, 132.2 (d, *J* = 9.3 Hz), 128.1, 126.9 (t, *J* = 241.9 Hz), 125.6, 115.7 (d, *J* = 22.0 Hz), 63.1, 43.2 (t, *J* = 24.3 Hz), 39.1 (t, *J* = 25.2 Hz), 36.1, 31.5, 31.1, 28.1 (t, *J* = 4.9 Hz), 25.3 (t, *J* = 3.0 Hz), 20.7. HRMS-EI (m/z): Calcd for $C_{25}H_{31}F_3O_2^+$ $[M]^+$ 420.2271, found 420.2262.

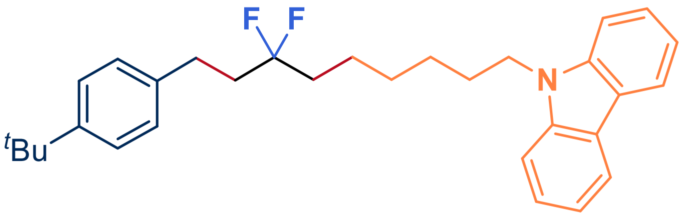

9-(9-(4-(tert-butyl)phenyl)-7,7-difluorononyl)-9H-carbazole (**2l**)

Following the general procedure **C**, the title compound (25.4 mg, colourless liquid) was obtained in 55% yield.

^{1}H NMR (400 MHz, $CDCl_3$) δ 8.10 (dt, *J* = 7.8, 1.0 Hz, 2H), 7.45 (ddd, *J* = 8.2, 7.0, 1.2 Hz, 2H), 7.39 (dt, *J* = 8.2, 1.0 Hz, 2H), 7.33 – 7.27 (m, 2H), 7.26 – 7.19 (m, 2H), 7.14 – 7.05 (m, 2H), 4.30 (t, *J* = 7.2 Hz, 2H), 2.77 – 2.68 (m, 2H), 2.14 – 1.96 (m, 2H), 1.95 – 1.69 (m, 4H), 1.48 – 1.32 (m, 6H), 1.30 (s, 9H). **^{19}F NMR** (377 MHz, $CDCl_3$) δ -98.60 (p, *J* = 16.7 Hz). **^{13}C NMR** (126 MHz, $CDCl_3$) δ 149.2, 140.5, 137.8, 128.1, 125.7, 125.6, 124.9 (t, *J* = 240.7 Hz), 123.0, 120.5, 118.9, 108.7, 43.1, 38.3 (t, *J* = 25.5 Hz), 36.5 (t, *J* = 25.4 Hz), 34.5, 31.5, 29.3, 29.0, 28.0 (t, *J* = 4.6 Hz), 27.2, 22.4 (t,

J = 4.5 Hz). HRMS-EI (m/z): Calcd for $C_{31}H_{38}F_2N^+$ $[M]^+$ 462.2967, found 462.2966.

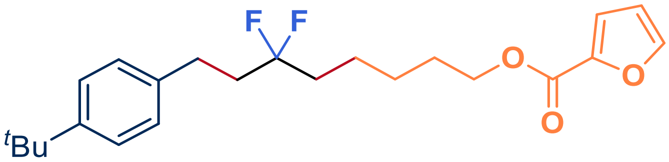

9-(4-(tert-butyl)phenyl)-6,6-difluorononyl furan-2-carboxylate (**2m**)

Following the general procedure **C**, the title compound (23.5 mg, colourless liquid) was obtained in 60% yield.

^{1}H NMR (400 MHz, $CDCl_3$) δ 7.57 (dd, J = 1.8, 0.9 Hz, 1H), 7.36 – 7.29 (m, 2H), 7.17 (dd, J = 3.5, 0.9 Hz, 1H), 7.16 – 7.11 (m, 2H), 6.50 (dd, J = 3.5, 1.7 Hz, 1H), 4.31 (t, J = 6.6 Hz, 2H), 2.90 – 2.68 (m, 2H), 2.21 – 2.02 (m, 2H), 1.93 – 1.82 (m, 2H), 1.77 (dt, J = 14.0, 6.8 Hz, 2H), 1.57 – 1.42 (m, 4H), 1.31 (s, 9H). **^{19}F NMR** (377 MHz, $CDCl_3$) δ -98.74 (p, J = 16.6 Hz). **^{13}C NMR** (126 MHz, $CDCl_3$) δ 158.9, 149.2, 146.4, 137.8, 128.1, 125.6, 124.79 (t, J = 240.5 Hz), 118.0, 112.0, 64.9, 38.4 (t, J = 25.5 Hz), 36.6 (t, J = 25.3 Hz), 34.5, 31.5, 28.7, 28.0 (t, J = 4.8 Hz), 25.9, 22.2 (t, J = 4.7 Hz). HRMS-EI (m/z): Calcd for $C_{23}H_{30}F_2O_3^+$ $[M]^+$ 392.2158, found 392.2166.

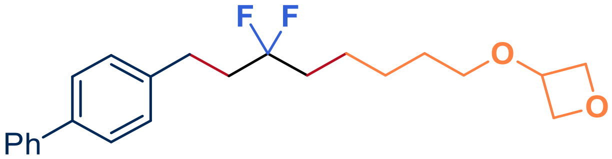

3-((8-([1,1'-biphenyl]-4-yl)-6,6-difluorooctyl)oxy)oxetane (**2n**)

Following the general procedure **C**, the title compound (15.0 mg, white solid, m.p. 49.3-50.5 °C) was obtained in 40% yield.

^{1}H NMR (400 MHz, $CDCl_3$) δ 7.62 – 7.54 (m, 2H), 7.58 – 7.50 (m, 2H), 7.48 – 7.39 (m, 2H), 7.38 – 7.29 (m, 1H), 7.32 – 7.25 (m, 2H), 4.84 – 4.69 (m, 2H), 4.64 – 4.57 (m, 2H), 4.52 (qd, J = 6.0, 4.8 Hz, 2H), 3.35 (t, J = 6.5 Hz, 1H), 2.93 – 2.77 (m, 2H), 2.29 – 2.08 (m, 2H), 1.98 – 1.79 (m, 2H), 1.63 – 1.50 (m, 4H), 1.46 – 1.37 (m, 2H). **^{19}F NMR** (377 MHz, $CDCl_3$) δ -98.76 (p, J = 16.5 Hz). **^{13}C NMR** (126 MHz, $CDCl_3$) δ 141.0, 134.0, 139.4, 128.9, 128.8, 127.4, 127.3, 127.1, 124.8 (t, J = 240.4 Hz), 79.0, 72.3, 68.7, 38.4 (t, J = 25.5 Hz), 36.7 (t, J = 25.3 Hz), 29.6, 28.2 (t, J = 4.8 Hz), 26.1, 22.3 (t, J = 4.4 Hz). HRMS-EI (m/z): Calcd for $C_{23}H_{28}F_2O_2^+$ $[M]^+$ 374.2052, found 374.2053.

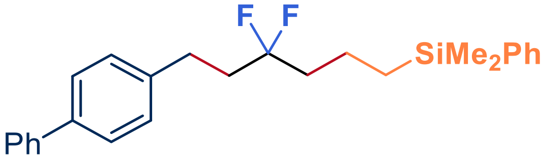

(6-([1,1'-biphenyl]-4-yl)-4,4-difluorohexyl)dimethyl(phenyl)silane (**2o**)

Following the general procedure **C**, the title compound (36.3 mg, colourless liquid) was obtained in 89% yield.

^{1}H NMR (400 MHz, $CDCl_3$) δ 7.60 – 7.55 (m, 2H), 7.54 – 7.48 (m, 4H), 7.46 – 7.39

(m, 2H), 7.38 – 7.30 (m, 4H), 7.26 – 7.21 (m, 2H), 2.87 – 2.73 (m, 2H), 2.19 – 2.01 (m, 2H), 1.96 – 1.79 (m, 2H), 1.60 – 1.45 (m, 2H), 0.81 – 0.74 (m, 2H), 0.28 (s, 6H). ^{19}**F NMR** (377 MHz, $CDCl_3$) δ -98.49 (p, J = 16.7 Hz). ^{13}**C NMR** (126 MHz, $CDCl_3$) δ 141.1, 140.0, 139.3, 139.1, 133.7, 129.11, 128.9, 128.9, 128.0, 127.4, 127.3, 127.2, 124.7 (t, J = 240.8 Hz), 40.4 (t, J = 24.8 Hz), 38.4 (t, J = 25.5 Hz), 28.3 (t, J = 4.8 Hz), 17.1 (t, J = 4.6 Hz), 15.9, -3.0. HRMS-EI (m/z): Calcd for $C_{26}H_{30}F_2Si^+$ $[M]^+$ 408.2079, found 408.2080.

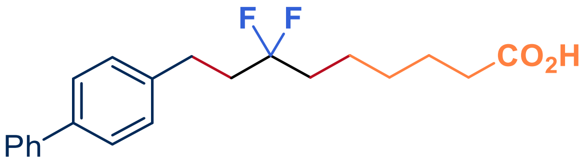

9-([1,1'-biphenyl]-4-yl)-7,7-difluorononanoic acid (**2p**)

Following the general procedure **C**, the title compound (30.5 mg, white solid, m.p. 112.0-113.0 °C) was obtained in 88% yield.

^{1}**H NMR** (400 MHz, $CDCl_3$) δ 7.60 – 7.55 (m, 2H), 7.55 – 7.51 (m, 2H), 7.46 – 7.40 (m, 2H), 7.37 – 7.30 (m, 1H), 7.30 – 7.25 (m, 2H), 2.88 – 2.79 (m, 2H), 2.37 (t, J = 7.4 Hz, 2H), 2.25 – 2.06 (m, 2H), 1.95 – 1.78 (m, 2H), 1.66 (p, J = 7.4 Hz, 2H), 1.58 – 1.48 (m, 2H), 1.44 – 1.35 (m, 2H). ^{19}**F NMR** (377 MHz, $CDCl_3$) δ -98.83 (p, J = 16.7 Hz). ^{13}**C NMR** (126 MHz, $CDCl_3$) δ 179.0, 141.1, 140.0, 139.4, 128.9, 128.9, 127.4, 127.3, 127.2, 124.7 (t, J = 240.9 Hz), 38.4 (t, J = 25.4 Hz), 36.5 (t, J = 25.1 Hz), 33.8, 28.9, 28.3 (t, J = 4.7 Hz), 24.6, 22.2 (t, J = 4.3 Hz). HRMS-EI (m/z): Calcd for $C_{21}H_{24}F_2O_2^+$ $[M]^+$ 346.1739, found 346.1742.

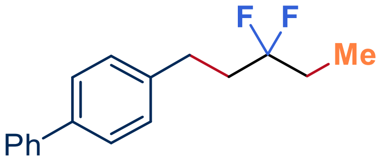

4-(3,3-difluoropentyl)-1,1'-biphenyl (**2q**)

Following the general procedure **C**, the title compound (12.0 mg, colourless liquid) was obtained in 46% yield.

^{1}**H NMR** (400 MHz, $CDCl_3$) δ 7.61 – 7.52 (m, 4H), 7.47 – 7.41 (m, 2H), 7.37 – 7.32 (m, 1H), 7.31 – 7.27 (m, 2H), 2.90 – 2.82 (m, 2H), 2.25 – 2.08 (m, 2H), 1.92 (ddt, J = 23.9, 16.6, 7.5 Hz, 2H), 1.06 (t, J = 7.5 Hz, 3H). ^{19}**F NMR** (377 MHz, $CDCl_3$) δ -100.84 (p, J = 16.4 Hz). ^{13}**C NMR** (126 MHz, $CDCl_3$) δ 141.1, 140.1, 139.3, 128.9, 128.9, 127.4, 127.3, 127.2, 125.1 (t, J = 240.7 Hz)., 38.0 (t, J = 25.5 Hz), 29.9 (t, J = 26.2 Hz), 28.2 (t, J = 5.1 Hz), 6.8 (t, J = 5.7 Hz). HRMS-EI (m/z): Calcd for $C_{17}H_{18}F_2^+$ $[M]^+$ 260.1371, found 260.1375.

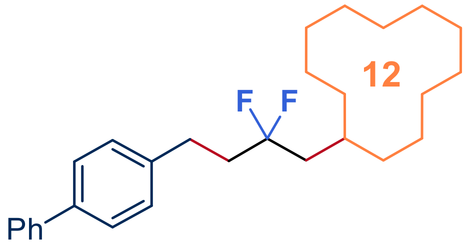

4-(4-cyclododecyl-3,3-difluorobutyl)-1,1'-biphenyl (**2r**)

Following the general procedure **C**, the title compound (26.8 mg, white solid, m.p. 78.1-79.6 °C) was obtained in 65% yield.

^{1}H NMR (400 MHz, $CDCl_3$) δ 7.60 – 7.52 (m, 4H), 7.43 (dd, J = 8.3, 6.9 Hz, 2H), 7.36 – 7.31 (m, 1H), 7.30 – 7.27 (m, 2H), 2.90 – 2.81 (m, 2H), 2.26 – 2.09 (m, 2H), 1.88 – 1.73 (m, 3H), 1.40 – 1.31 (m, 22H). **^{19}F NMR** (377 MHz, $CDCl_3$) δ -96.12 (p, J = 17.1 Hz). **^{13}C NMR** (126 MHz, $CDCl_3$) δ 141.1, 140.1, 139.3, 128.9, 127.4, 127.3, 127.2, 125.3 (t, J = 241.8 Hz), 41.5 (t, J = 23.9 Hz), 39.1 (t, J = 25.7 Hz), 30.0, 29.0, 28.4 (t, J = 5.2 Hz), 24.7, 24.3, 23.5, 23.4, 21.7. HRMS-EI (m/z): Calcd for $C_{28}H_{38}F_2^+$ $[M]^+$ 412.2936, found 412.2922.

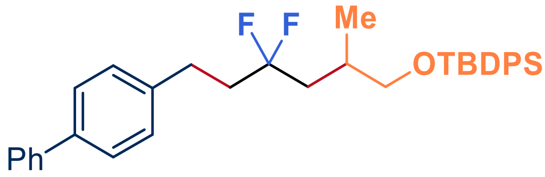

((6-([1,1'-biphenyl]-4-yl)-4,4-difluoro-2-methylhexyl)oxy)(tert-butyl)diphenylsilane (**2s**)

Following the general procedure **C**, the title compound (42.3 mg, colourless liquid) was obtained in 78% yield.

^{1}H NMR (400 MHz, $CDCl_3$) δ 7.70 – 7.66 (m, 4H), 7.61 – 7.57 (m, 2H), 7.57 – 7.51 (m, 2H), 7.49 – 7.32 (m, 9H), 7.30 – 7.26 (m, 2H), 3.64 – 3.43 (m, 2H), 2.86 (t, J = 8.6 Hz, 2H), 2.30 – 1.94 (m, 4H), 1.69 (dddd, J = 22.4, 14.7, 11.4, 8.2 Hz, 1H), 1.08 (s, 9H), 1.05 (d, J = 6.4 Hz, 3H). **^{19}F NMR** (377 MHz, $CDCl_3$) δ -94.12 – -95.86 (m), -96.16 – -98.48 (m). **^{13}C NMR** (126 MHz, $CDCl_3$) δ 141.1, 140.0, 139.3, 135.7, 133.9, 129.8, 128.9, 128.9, 127.8, 127.4, 127.3, 127.2, 124.3 (d, J = 241.4 Hz), 68.8, 39.6 (t, J = 24.3 Hz), 39.1 (t, J = 25.6 Hz), 31.1 (t, J = 2.9 Hz), 28.3 (t, J = 4.8 Hz), 27.0, 19.5, 17.8. HRMS-ESI (m/z): Calcd for $C_{35}H_{41}F_2OSi^+$ $[M+H]^+$ 543.2889, found 543.2893.

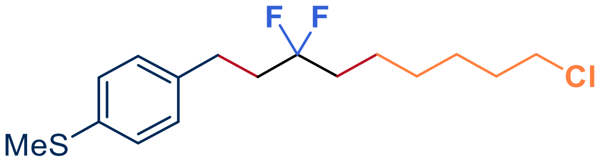

(4-(9-chloro-3,3-difluorononyl)phenyl)(methyl)sulfane (**2t**)

Following the general procedure **C**, the title compound (9.6 mg, colourless liquid) was obtained in 30% yield.

^{1}H NMR (400 MHz, $CDCl_3$) δ 7.24 – 7.18 (m, 2H), 7.15 – 7.09 (m, 2H), 3.54 (t, J = 6.7 Hz, 2H), 2.83 – 2.72 (m, 2H), 2.47 (s, 3H), 2.19 – 1.99 (m, 2H), 1.94 – 1.73 (m,

4H), 1.53 – 1.40 (m, 4H), 1.40 – 1.29 (m, 2H). **^{19}F NMR** (377 MHz, $CDCl_3$) δ -98.76 (p, *J* = 16.5 Hz). **^{13}C NMR** (126 MHz, $CDCl_3$) δ 137.9, 136.1, 129.0, 127.4, 124.7 (t, *J* = 241.0 Hz), 45.1, 38.4 (t, *J* = 25.5 Hz), 36.6 (t, *J* = 25.3 Hz), 32.5, 28.8, 28.1 (t, *J* = 5.1 Hz), 26.8, 22.3 (t, *J* = 4.6 Hz), 16.4. HRMS-EI (m/z): Calcd for $C_{16}H_{23}ClF_2S^+$ $[M]^+$ 320.1172, found 320.1169.

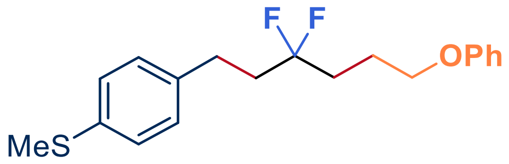

(4-(3,3-difluoro-6-phenoxyhexyl)phenyl)(methyl)sulfane (**2u**)

Following the general procedure **C**, the title compound (14.5 mg, white solid, m.p. 39.1-39.5 °C) was obtained in 43% yield.

^{1}H NMR (400 MHz, $CDCl_3$) δ 7.31 – 7.26 (m, 2H), 7.23 – 7.19 (m, 2H), 7.15 – 7.11 (m, 2H), 6.95 (tt, *J* = 7.4, 1.1 Hz, 1H), 6.91 – 6.87 (m, 2H), 4.01 (t, *J* = 5.9 Hz, 2H), 2.84 – 2.76 (m, 2H), 2.47 (s, 3H), 2.23 – 1.97 (m, 6H). **^{19}F NMR** (377 MHz, $CDCl_3$) δ -99.26 (p, *J* = 16.4 Hz). **^{13}C NMR** (126 MHz, $CDCl_3$) δ 158.9, 137.8, 136.1, 129.6, 129.0, 127.4, 124.6 (t, *J* = 241.2 Hz), 121.0, 114.6, 67.0, 38.6 (t, *J* = 25.4 Hz), 33.5 (t, *J* = 25.6 Hz), 28.1 (t, *J* = 5.0 Hz), 22.6 (t, *J* = 4.7 Hz), 16.4. HRMS-EI (m/z): Calcd for $C_{19}H_{22}F_2OS^+$ $[M]^+$ 336.1359, found 336.1363.

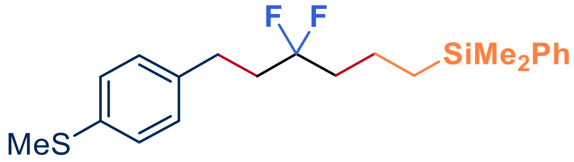

(4,4-difluoro-6-(4-(methylthio)phenyl)hexyl)dimethyl(phenyl)silane (**2v**)

Following the general procedure **C**, the title compound (20.8 mg, white solid, m.p. 40.0-40.5 °C) was obtained in 55% yield.

^{1}H NMR (400 MHz, $CDCl_3$) δ 7.53 – 7.48 (m, 2H), 7.39 – 7.33 (m, 3H), 7.23 – 7.17 (m, 2H), 7.12 – 7.06 (m, 2H), 2.75 – 2.68 (m, 2H), 2.47 (s, 3H), 2.13 – 1.93 (m, 2H), 1.93 – 1.78 (m, 2H), 1.58 – 1.45 (m, 2H), 0.82 – 0.72 (m, 2H), 0.28 (s, 6H). **^{19}F NMR** (377 MHz, $CDCl_3$) δ -98.55 (p, *J* = 16.7 Hz). **^{13}C NMR** (126 MHz, $CDCl_3$) δ 139.1, 138.0, 136.0, 133.7, 129.1, 129.0, 128.0, 127.4, 124.6 (t, *J* = 241.7 Hz), 40.4 (t, *J* = 24.7 Hz), 38.4 (t, *J* = 25.7 Hz), 28.1 (t, *J* = 5.0 Hz), 17.1 (t, *J* = 4.9 Hz), 16.4, 15.9, -3.0. HRMS-EI (m/z): Calcd for $C_{21}H_{28}F_2SSi^+$ $[M]^+$ 378.1644, found 378.1649.

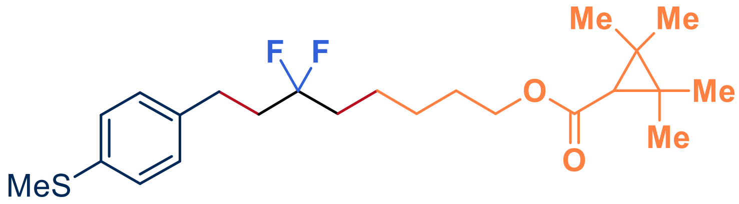

6,6-difluoro-8-(4-(methylthio)phenyl)octyl 2,2,3,3-tetramethylcyclopropane-1-carboxylate (**2w**)

Following the general procedure **C**, the title compound (26.4 mg, colourless liquid)

was obtained in 64% yield.
^{1}H NMR (400 MHz, $CDCl_3$) δ 7.23 – 7.18 (m, 2H), 7.14 – 7.11 (m, 2H), 4.02 (t, *J* = 6.7 Hz, 2H), 2.82 – 2.70 (m, 2H), 2.47 (s, 3H), 2.18 – 2.01 (m, 2H), 1.92 – 1.77 (m, 2H), 1.70 – 1.57 (m, 2H), 1.57 – 1.47 (m, 2H), 1.45 – 1.38 (m, 2H), 1.24 (s, 6H), 1.18 (s, 6H). **^{19}F NMR** (377 MHz, $CDCl_3$) δ -98.78 (p, *J* = 16.5 Hz). **^{13}C NMR** (126 MHz, $CDCl_3$) δ 172.4, 137.9, 136.0, 129.0, 127.4, 124.7 (t, *J* = 240.9 Hz), 63.6, 38.4 (t, *J* = 25.4 Hz), 36.6 (t, *J* = 25.3 Hz), 35.9, 30.1, 28.7, 28.1 (t, *J* = 4.9 Hz), 26.0, 23.7, 22.2 (t, *J* = 4.4 Hz), 16.7, 16.4. HRMS-EI (m/z): Calcd for $C_{23}H_{34}F_2O_2S^+$ $[M]^+$ 412.2242, found 412.2246.

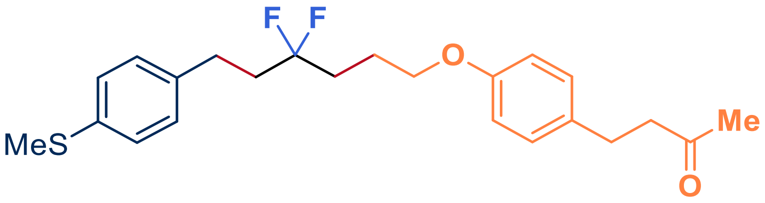

4-(4-((6,6-difluoro-8-(4-(methylthio)phenyl)octyl)oxy)phenyl)butan-2-one (**2x**)
Following the general procedure **C**, the title compound (15.2 mg, colourless liquid) was obtained in 35% yield.
^{1}H NMR (400 MHz, $CDCl_3$) δ 7.24 – 7.19 (m, 2H), 7.14 – 7.05 (m, 4H), 6.87 – 6.75 (m, 2H), 3.93 (t, *J* = 6.4 Hz, 2H), 2.88 – 2.80 (m, 2H), 2.79 – 2.67 (m, 4H), 2.47 (s, 3H), 2.19 – 2.03 (m, 5H), 1.94 – 1.71 (m, 4H), 1.63 – 1.43 (m, 4H). **^{19}F NMR** (377 MHz, $CDCl_3$) δ -98.74 (p, *J* = 16.7 Hz). **^{13}C NMR** (126 MHz, $CDCl_3$) δ 208.3, 157.5, 137.9, 136.0, 133.1, 129.3, 129.0, 127.3, 124.7 (t, *J* = 240.8 Hz), 114.6, 67.8, 45.6, 38.4 (t, *J* = 25.5 Hz), 36.6 (t, *J* = 25.3 Hz), 30.2, 29.2, 29.0, 28.1 (t, *J* = 5.1 Hz), 26.1, 22.3 (t, *J* = 4.5 Hz), 16.4. HRMS-EI (m/z): Calcd for $C_{25}H_{32}F_2O_2S^+$ $[M]^+$ 434.2086, found 434.2078.

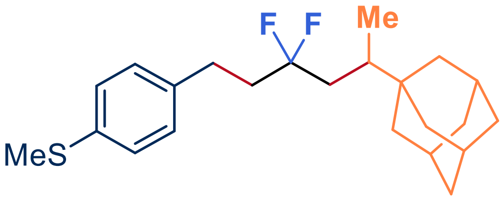

(4-(5-((3r,5r,7r)-adamantan-1-yl)-3,3-difluorohexyl)phenyl)(methyl)sulfane (**2y**)
Following the general procedure **C**, the title compound (15.1 mg, colourless liquid) was obtained in 40% yield.
^{1}H NMR (400 MHz, $CDCl_3$) δ 7.25 – 7.17 (m, 2H), 7.17 – 7.09 (m, 2H), 2.77 (t, *J* = 8.5 Hz, 2H), 2.47 (s, 3H), 2.06 – 1.92 (m, 4H), 1.73 – 1.39 (m, 16H), 0.94 (d, *J* = 6.7 Hz, 3H). **^{19}F NMR** (377 MHz, $CDCl_3$) δ -95.56 (dtdd, *J* = 239.5, 21.2, 14.1, 10.6 Hz), -97.70 (dqd, *J* = 239.4, 19.6, 11.8 Hz). **^{13}C NMR** (126 MHz, $CDCl_3$) δ 138.1, 136.0, 129.0, 127.4, 125.7 (t, *J* = 241.6 Hz), 39.2, 39.0, 38.8, 37.7, 37.6, 37.5, 37.4, 34.8, 28.8, 28.1 (t, *J* = 5.1 Hz), 16.4, 14.6. HRMS-EI (m/z): Calcd for $C_{23}H_{32}F_2S^+$ $[M]^+$ 378.2187, found 378.2200.

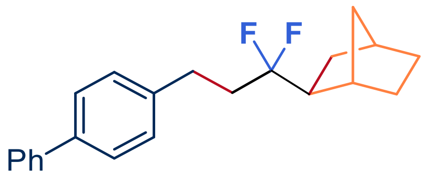

2-(3-([1,1'-biphenyl]-4-yl)-1,1-difluoropropyl)bicyclo[2.2.1]heptane (**2z**)
Following the general procedure **C** (adding 50 equiv. of H_2O), the title compound (20.6 mg, colourless liquid) was obtained in 63% yield.
^{1}H NMR (400 MHz, $CDCl_3$) δ 7.62 – 7.57 (m, 2H), 7.57 – 7.52 (m, 2H), 7.48 – 7.41 (m, 2H), 7.38 – 7.34 (m, 1H), 7.31 – 7.27 (m, 2H), 2.96 – 2.80 (m, 2H), 2.51 – 2.42 (m, 1H), 2.31 (q, *J* = 2.8 Hz, 1H), 2.25 – 2.05 (m, 2H), 1.85 (dddd, *J* = 18.5, 15.5, 9.1, 6.4 Hz, 1H), 1.61 – 1.50 (m, 4H), 1.44 (ddd, *J* = 11.7, 8.9, 2.4 Hz, 1H), 1.16 (ddt, *J* = 19.2, 9.7, 1.8 Hz, 3H). **^{19}F NMR** (377 MHz, $CDCl_3$) δ -99.20 – -104.70 (m), -105.19 – -108.96 (m). **^{13}C NMR** (126 MHz, $CDCl_3$) δ 141.1, 140.3, 139.3, 128.9, 127.4, 127.3, 127.2, 125.7 (t, *J* = 243.2 Hz), 47.9 (t, *J* = 23.9 Hz), 37.7 (t, *J* = 26.0 Hz), 37.6 (m), 36.9, 36.0, 32.6 (m), 30.8, 28.3, 28.1 (t, *J* = 4.9 Hz). HRMS-EI (m/z): Calcd for $C_{22}H_{24}F_2^+$ $[M]^+$ 326.1841, found 326.1842.

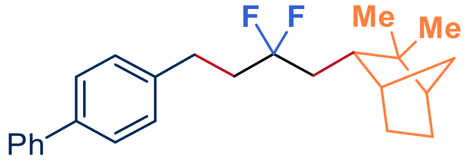

3-(4-([1,1'-biphenyl]-4-yl)-2,2-difluorobutyl)-2,2-dimethylbicyclo[2.2.1]heptane (**2aa**)
Following the general procedure **C** (adding 50 equiv. of H_2O), the title compound (24.7 mg, white solid, m.p. 44.0-44.8 ^{o}C) was obtained in 67% yield.
^{1}H NMR (400 MHz, $CDCl_3$) δ 7.62 – 7.57 (m, 2H), 7.56 – 7.52 (m, 2H), 7.47 – 7.41 (m, 2H), 7.38 – 7.32 (m, 1H), 7.31 – 7.27 (m, 2H), 2.92 – 2.80 (m, 2H), 2.27 – 2.09 (m, 3H), 1.96 – 1.74 (m, 3H), 1.72 – 1.62 (m, 2H), 1.61 – 1.51 (m, 1H), 1.40 – 1.23 (m, 3H), 1.20 (dt, *J* = 9.8, 1.7 Hz, 1H), 0.97 (s, 3H), 0.81 (s, 3H). **^{19}F NMR** (377 MHz, $CDCl_3$) δ -95.59 – -96.68 (m), -97.38 – -98.48 (m). **^{13}C NMR** (126 MHz, $CDCl_3$) δ 141.1, 140.1, 139.3, 128.9, 127.4, 127.3, 127.2, 125.3 (t, *J* = 241.8 Hz), 48.7, 44.4, 42.4, 39.0 (t, *J* = 25.7 Hz), 37.2, 33.3 (t, *J* = 24.6 Hz), 32.1, 28.4 (t, *J* = 5.1 Hz), 24.8, 22.2, 20.4. HRMS-EI (m/z): Calcd for $C_{25}H_{30}F_2^+$ $[M]^+$ 368.2310, found 368.2306.

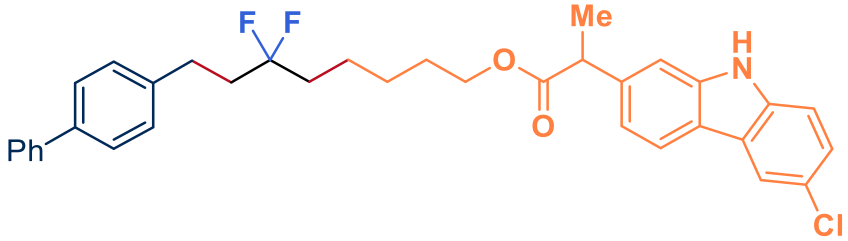

8-([1,1'-biphenyl]-4-yl)-6,6-difluorooctyl 2-(6-chloro-9H-carbazol-2-yl)propanoate (**2ab**)

Following the general procedure **C** (adding 50 equiv. of H_2O), the title compound (25.8 mg, white solid, m.p. 67.1-69.0 °C) was obtained in 45% yield.

^{1}H NMR (400 MHz, $CDCl_3$) δ 8.13 (br, 1H), 8.03 – 7.83 (m, 2H), 7.64 – 7.57 (m, 2H), 7.57 – 7.52 (m, 2H), 7.48 – 7.40 (m, 2H), 7.37 – 7.27 (m, 4H), 7.26 – 7.22 (m, 2H), 7.19 (dd, *J* = 8.1, 1.5 Hz, 1H), 4.25 – 3.99 (m, 2H), 3.88 (q, *J* = 7.1 Hz, 1H), 2.87 – 2.66 (m, 2H), 2.16 – 1.95 (m, 2H), 1.80 – 1.64 (m, 3H), 1.59 (dd, *J* = 7.0, 2.8 Hz, 5H), 1.49 – 1.37 (m, 2H), 1.30 – 1.22 (m, 1H). **^{19}F NMR** (377 MHz, $CDCl_3$) δ -98.95 (p, *J* = 16.6 Hz). **^{13}C NMR** (126 MHz, $CDCl_3$) δ 174.9, 141.0, 140.4, 139.9, 139.5, 139.3, 138.1, 128.9, 128.8, 127.4, 127.3, 127.1, 126.0, 125.1, 124.7 (t, *J* = 240.8 Hz), 124.4, 121.7, 120.7, 120.1, 119.8, 111.7, 109.7, 64.7, 46.1, 38.3 (t, *J* = 25.5 Hz), 36.5 (t, *J* = 25.3 Hz), 28.5, 28.2 (t, *J* = 5.0 Hz), 25.7, 22.0 (t, *J* = 4.5 Hz), 18.9. HRMS-APCI(m/z): Calcd for $C_{35}H_{35}ClF_2NO_2^+$ $[M]^+$ 574.2319, found 574.2320.

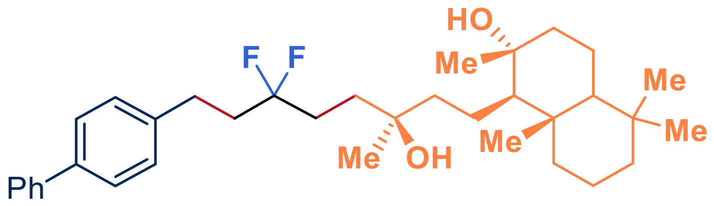

(1R,2R,8aS)-1-((S)-9-([1,1'-biphenyl]-4-yl)-7,7-difluoro-3-hydroxy-3-methylnonyl)-2,5,5,8a-tetramethyldecahydronaphthalen-2-ol (**2ac**)

Following the general procedure **C** (adding 50 equiv. of H_2O), the title compound (38.8 mg, colourless liquid) was obtained in 70% yield.

^{1}H NMR (400 MHz, $CDCl_3$) δ 7.61 – 7.55 (m, 2H), 7.56 – 7.51 (m, 2H), 7.43 (dd, *J* = 8.5, 6.9 Hz, 2H), 7.36 – 7.30 (m, 1H), 7.30 – 7.26 (m, 2H), 2.92 – 2.77 (m, 2H), 2.27 – 2.10 (m, 3H), 2.10 – 1.91 (m, 2H), 1.84 (dt, *J* = 12.1, 3.1 Hz, 1H), 1.71 – 1.48 (m, 8H), 1.47 – 1.31 (m, 5H), 1.22 – 1.11 (m, 8H), 0.99 – 0.89 (m, 2H), 0.87 (s, 3H), 0.79 (d, *J* = 2.3 Hz, 6H). **^{19}F NMR** (377 MHz, $CDCl_3$) δ -98.73 (p, *J* = 16.4 Hz). **^{13}C NMR** (126 MHz, $CDCl_3$) δ 141.1, 140.0, 139.3, 128.9, 127.4, 127.3, 127.1, 125.2 (t, *J* = 240.8 Hz), 75.1, 72.3, 62.0, 56.2, 44.7, 44.4, 42.1, 39.8, 39.3, 38.5 (t, *J* = 25.4 Hz), 35.5, 33.5, 33.3, 31.3 (t, *J* = 25.2 Hz), 28.3 (t, *J* = 5.0 Hz), 25.9, 24.4, 21.6, 20.6, 19.0, 18.5, 15.5. HRMS-EI (m/z): Calcd for $C_{36}H_{50}F_2O^+$ $[M-H_2O]^+$ 536.3830, found 536.3830.

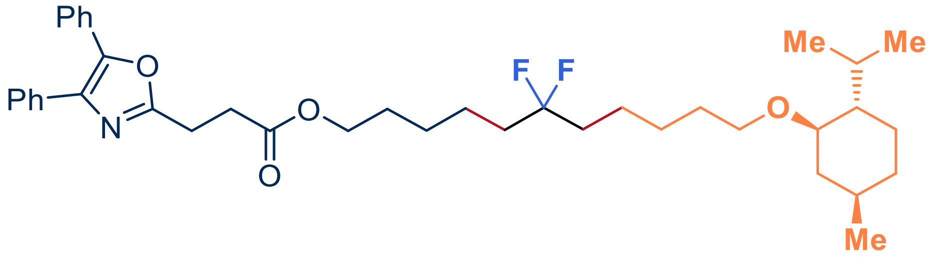

6,6-difluoro-11-(((1R,2S,5R)-2-isopropyl-5-methylcyclohexyl)oxy)undecyl 3-(4,5-diphenyloxazol-2-yl)propanoate (**2ad**)

Following the general procedure **C** (adding 50 equiv. of H_2O), the title compound (35.1 mg, colourless liquid) was obtained in 55% yield.

^{1}H NMR (400 MHz, $CDCl_3$) δ 7.65 – 7.60 (m, 2H), 7.59 – 7.55 (m, 2H), 7.40 – 7.28 (m, 6H), 4.13 (t, *J* = 6.6 Hz, 2H), 3.61 (dt, *J* = 9.1, 6.2 Hz, 1H), 3.25 (dt, *J* = 9.2, 6.6 Hz, 1H), 3.19 (dd, *J* = 8.1, 6.9 Hz, 2H), 2.99 (td, *J* = 10.5, 4.1 Hz, 1H), 2.91 (dd, *J* = 8.2, 6.9 Hz, 2H), 2.21 (qd, *J* = 7.0, 2.7 Hz, 1H), 2.10 – 2.03 (m, 1H), 1.87 – 1.69 (m, 4H), 1.69 – 1.53 (m, 6H), 1.50 – 1.28 (m, 9H), 1.23 – 1.13 (m, 1H), 0.96 (dd, *J* = 12.7, 3.2 Hz, 1H), 0.90 (dd, *J* = 9.0, 6.8 Hz, 6H), 0.86 – 0.80 (m, 2H), 0.76 (d, *J* = 6.9 Hz, 3H). **^{19}F NMR** (377 MHz, $CDCl_3$) δ -98.11 (p, *J* = 16.6 Hz). **^{13}C NMR** (126 MHz, $CDCl_3$) δ 172.2, 161.9, 145.6, 135.2, 132.5, 129.1, 128.8, 128.7, 128.6, 128.2, 128.0, 126.6, 125.3 (t, *J* = 240.1 Hz), 79.4, 68.4, 64.8, 48.5, 40.7, 36.5 (t, *J* = 25.4 Hz), 36.3 (t, *J* = 25.9 Hz), 34.8, 31.7, 31.3, 30.2, 28.6, 26.3, 25.9, 25.8, 23.7, 23.5, 22.5, 22.3 (t, *J* = 4.7 Hz), 22.1 (t, *J* = 4.4 Hz), 21.1, 16.4. HRMS-EI (m/z): Calcd for $C_{39}H_{54}F_2NO_4^+$ $[M+H]^+$ 638.4015, found 638.4011.

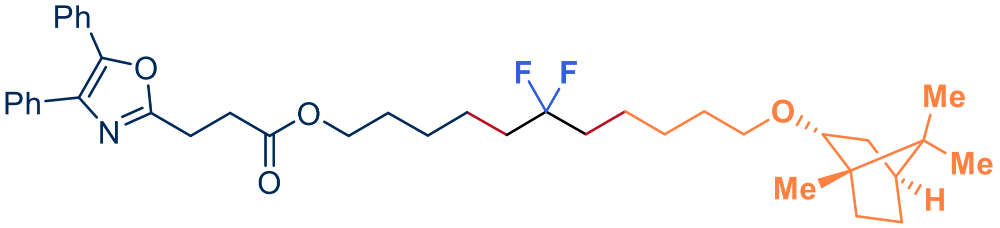

6,6-difluoro-11-(((1S,2R,4S)-1,7,7-trimethylbicyclo[2.2.1]heptan-2-yl)oxy)undecyl 3-(4,5-diphenyloxazol-2-yl)propanoate (**2ae**)

Following the general procedure **C** (adding 50 equiv. of H_2O), the title compound (33.1 mg, colourless liquid) was obtained in 52% yield.

^{1}H NMR (400 MHz, $CDCl_3$) δ 7.59 – 7.52 (m, 2H), 7.51 – 7.46 (m, 2H), 7.33 – 7.21 (m, 6H), 4.05 (t, *J* = 6.6 Hz, 2H), 3.45 (ddd, *J* = 9.4, 3.4, 1.9 Hz, 1H), 3.35 (dt, *J* = 9.3, 6.4 Hz, 2H), 3.11 (dd, *J* = 8.1, 6.9 Hz, 2H), 2.84 (dd, *J* = 8.1, 6.9 Hz, 2H), 2.02 (dddd, *J* = 12.8, 9.5, 4.8, 3.2 Hz, 1H), 1.89 (ddd, *J* = 12.0, 9.7, 4.6 Hz, 1H), 1.78 – 1.63 (m, 4H), 1.62 – 1.51 (m, 4H), 1.51 – 1.44 (m, 2H), 1.44 – 1.34 (m, 4H), 1.34 – 1.24 (m, 4H), 1.18 – 1.07 (m, 2H), 0.91 (dd, *J* = 12.9, 3.2 Hz, 1H), 0.78 (s, 3H), 0.76 (s, 6H). **^{19}F NMR** (377 MHz, $CDCl_3$) δ -98.02 (p, *J* = 16.6 Hz). **^{13}C NMR** (126 MHz, $CDCl_3$) δ 172.2, 161.9, 145.5, 135.2, 132.5, 129.1, 128.8, 128.7, 128.6, 128.2, 128.0, 126.6, 125.3 (t, *J* = 240.6 Hz), 84.8, 69.8, 64.8, 49.3, 47.9, 45.1, 36.5 (d, *J* = 25.5 Hz), 36.5, 36.3 (d, *J* = 25.5 Hz), 31.3, 30.0, 28.6, 28.4, 26.8, 26.4, 25.9, 23.7, 22.3 (t, *J* = 4.0 Hz), 22.1 (t, *J* = 4.3 Hz), 19.9, 19.0, 14.2. HRMS-EI (m/z): Calcd for $C_{39}H_{52}F_2NO_4^+$ $[M+H]^+$ 636.3859, found 636.3851.

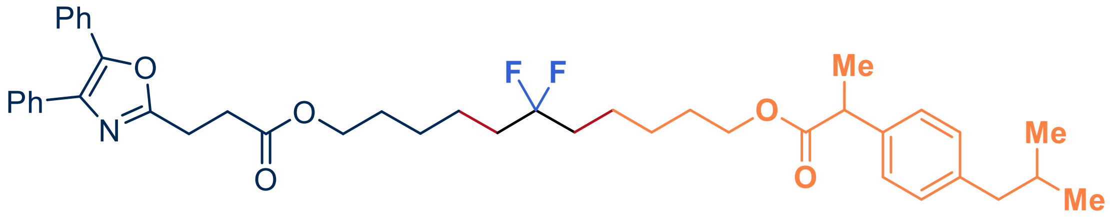

6,6-difluoro-11-((2-(4-isobutylphenyl)propanoyl)oxy)undecyl 3-(4,5-diphenyloxazol-2-yl)propanoate (**2af**)

Following the general procedure **C** (adding 50 equiv. of H_2O), the title compound (28.9 mg, colourless liquid) was obtained in 42% yield.

^{1}H NMR (500 MHz, $CDCl_3$) δ 7.66 – 7.61 (m, 2H), 7.59 – 7.55 (m, 2H), 7.38 – 7.30 (m, 6H), 7.20 (d, *J* = 8.1 Hz, 2H), 7.13 – 7.06 (m, 2H), 4.14 (t, *J* = 6.6 Hz, 2H), 4.06 (td, *J* = 6.6, 2.3 Hz, 2H), 3.68 (q, *J* = 7.2 Hz, 1H), 3.19 (dd, *J* = 8.1, 6.9 Hz, 2H), 2.92 (dd, *J* = 8.1, 6.9 Hz, 2H), 2.44 (d, *J* = 7.2 Hz, 2H), 1.85 (dq, *J* = 13.5, 6.8 Hz, 1H), 1.78 – 1.62 (m, 6H), 1.61 – 1.55 (m, 2H), 1.49 (d, *J* = 7.2 Hz, 3H), 1.48 – 1.34 (m, 6H), 1.27 (d, *J* = 8.2 Hz, 2H), 0.89 (d, *J* = 6.6 Hz, 6H). **^{19}F NMR** (377 MHz, $CDCl_3$) δ -98.28 (p, *J* = 16.4 Hz). **^{13}C NMR** (126 MHz, $CDCl_3$) δ 174.9, 172.1, 161.9, 145.5, 140.6, 138.0, 135.2, 132.5, 129.4, 128.8, 128.7, 128.6, 128.2, 128.0, 127.2, 126.6, 125.1 (t, *J* = 240.4 Hz), 64.7, 64.5, 45.3, 45.1, 36.3 (t, *J* = 25.6), 36.3 (t, *J* = 25.5), 31.3, 30.3, 29.8, 28.6, 28.5, 25.8, 25.7, 23.7, 22.5, 22.1 (t, *J* = 4.8 Hz), 22.0 (t, *J* = 4.6 Hz), 18.5. HRMS-EI (m/z): Calcd for $C_{42}H_{52}F_2NO_5^+$ $[M+H]^+$ 688.3808, found 688.3804.

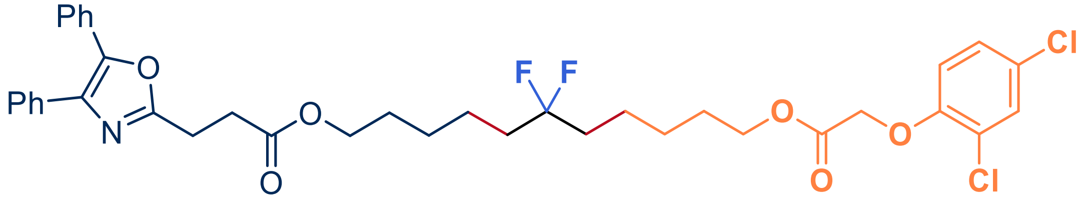

11-(2-(2,4-dichlorophenoxy)acetoxy)-6,6-difluoroundecyl 3-(4,5-diphenyloxazol-2-yl)propanoate (**2ag**)

Following the general procedure **C** (adding 50 equiv. of H_2O), the title compound (31.6 mg, colourless liquid) was obtained in 45% yield.

^{1}H NMR (400 MHz, $CDCl_3$) δ 7.66 – 7.60 (m, 2H), 7.59 – 7.54 (m, 2H), 7.41 – 7.30 (m, 6H), 7.26 (s, 1H), 7.16 (dd, *J* = 8.8, 2.5 Hz, 1H), 6.77 (d, *J* = 8.8 Hz, 1H), 4.68 (s, 2H), 4.20 (t, *J* = 6.6 Hz, 2H), 4.13 (t, *J* = 6.6 Hz, 2H), 3.19 (dd, *J* = 8.2, 6.8 Hz, 2H), 2.91 (dd, *J* = 8.2, 6.8 Hz, 2H), 1.81 – 1.61 (m, 8H), 1.52 – 1.29 (m, 8H). **^{19}F NMR** (377 MHz, $CDCl_3$) δ -98.43 (p, *J* = 16.6 Hz). **^{13}C NMR** (126 MHz, $CDCl_3$) δ 172.2, 168.3, 161.9, 152.6, 133.8, 130.5, 129.6, 129.0, 128.8, 128.7, 128.6, 128.2, 128.0, 127.7, 127.2, 126.6, 125.1 (t, *J* = 240.7 Hz), 124.4, 114.8, 66.5, 65.5, 64.8, 36.4 (t, *J* = 25.7 Hz), 36.3 (t, *J* = 25.5 Hz), 31.3, 28.6, 28.4, 25.9, 25.7, 23.7, 22.1 (t, *J* = 4.4 Hz), 22.0 (t, *J* = 4.6 Hz). HRMS-APCI (m/z): Calcd for $C_{37}H_{40}Cl_2F_2NO_6$ [M] 702.2195,

found 702.2202.

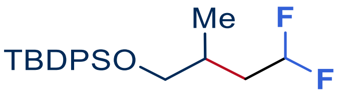

tert-butyl(4,4-difluoro-2-methylbutoxy)diphenylsilane (**3a**)

This compound is a known compound tha has been reported in a previous study. [5] Following the general procedure **D**, the title compound (44.9 mg, colourless liquid) was obtained in 62% yield.

^{1}H NMR (400 MHz, $CDCl_3$) δ 7.58 – 7.51 (m, 4H), 7.40 – 7.26 (m, 6H), 5.82 (tt, *J* = 57.0, 4.9 Hz, 1H), 3.53 – 3.32 (m, 2H), 2.03 – 1.78 (m, 2H), 1.60 (dtdd, *J* = 20.4, 13.8, 7.4, 4.9 Hz, 1H), 0.96 (s, 9H), 0.87 (d, *J* = 6.7 Hz, 3H). **^{19}F NMR** (377 MHz, $CDCl_3$) δ -113.70 (dddd, *J* = 281.2, 56.9, 20.7, 14.4 Hz), -115.12 (dddd, *J* = 281.3, 57.0, 21.5, 14.3 Hz).

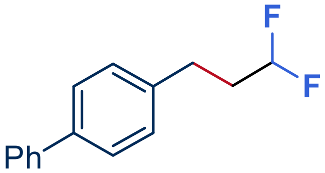

4-(3,3-difluoropropyl)-1,1'-biphenyl (**3b**)

Following the general procedure **D**, the title compound (41.8 mg, colourless liquid) was obtained in 90% yield.

^{1}H NMR (400 MHz, $CDCl_3$) δ 7.65 – 7.52 (m, 4H), 7.48 – 7.41 (m, 2H), 7.39 – 7.32 (m, 1H), 7.31 – 7.27 (m, 2H), 5.85 (tt, *J* = 56.7, 4.5 Hz, 1H), 3.16 – 2.44 (m, 2H), 2.35 – 1.99 (m, 2H). **^{19}F NMR** (377 MHz, $CDCl_3$) δ -117.07 (dt, *J* = 56.5, 17.0 Hz). **^{13}C NMR** (126 MHz, $CDCl_3$) δ 141.0, 139.6, 139.1, 128.9, 128.9, 127.5, 127.4, 127.2, 116.8 (t, *J* = 239.1 Hz), 35.8 (t, *J* = 21.1 Hz), 28.2 (t, *J* = 6.0 Hz). HRMS-EI (m/z): Calcd for $C_{15}H_{14}F_2^+$ $[M]^+$ 232.1058, found 232.1058.

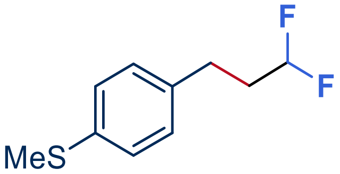

(4-(3,3-difluoropropyl)phenyl)(methyl)sulfane (**3c**)

Following the general procedure **D**, the title compound (32.3 mg, colourless liquid) was obtained in 80% yield.

^{1}H NMR (400 MHz, $CDCl_3$) δ 7.25 – 7.19 (m, 2H), 7.17 – 7.09 (m, 2H), 5.80 (tt, *J* = 56.6, 4.5 Hz, 1H), 2.84 – 2.67 (m, 2H), 2.48 (s, 3H), 2.31 – 1.89 (m, 2H). **^{19}F NMR** (377 MHz, $CDCl_3$) δ -117.12 (dt, *J* = 56.6, 17.0 Hz). **^{13}C NMR** (126 MHz, $CDCl_3$) δ 137.0, 136.4, 129.0, 127.3, 116.8 (t, *J* = 239.1 Hz), 35.8 (t, *J* = 21.0 Hz), 28.0 (t, *J* = 6.0 Hz), 16.3. HRMS-EI (m/z): Calcd for $C_{10}H_{12}F_2S^+$ $[M]^+$ 202.0622, found 202.0622.

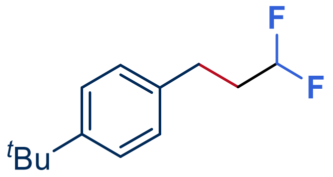

1-(tert-butyl)-4-(3,3-difluoropropyl)benzene (**3d**)

Following the general procedure **D**, the title compound (38.2 mg, colourless liquid) was obtained in 90% yield.

^{1}H NMR (400 MHz, $CDCl_3$) δ 7.28 – 7.24 (m, 2H), 7.12 – 7.02 (m, 2H), 5.73 (tt, *J* = 56.7, 4.5 Hz, 1H), 2.71 – 2.63 (m, 2H), 2.21 – 1.99 (m, 2H), 1.24 (s, 9H). **^{19}F NMR** (377 MHz, $CDCl_3$) δ -117.09 (dt, *J* = 56.5, 17.0 Hz). **^{13}C NMR** (126 MHz, $CDCl_3$) δ 149.4, 136.9, 128.1, 125.7, 116.9 (t, *J* = 238.9 Hz), 35.8 (t, *J* = 21.0 Hz), 31.5, 28.0 (t, *J* = 5.9 Hz). HRMS-EI (m/z): Calcd for $C_{13}H_{18}F_2^+$ $[M]^+$ 212.1371, found 212.1371.

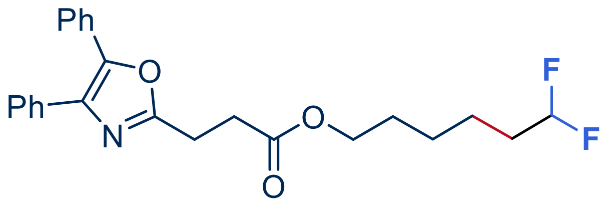

6,6-difluorohexyl 3-(4,5-diphenyloxazol-2-yl)propanoate (**3e**)

Following the general procedure **D**, the title compound (38.2 mg, colourless liquid) was obtained in 65% yield.

^{1}H NMR (400 MHz, $CDCl_3$) δ 7.68 – 7.60 (m, 2H), 7.60 – 7.51 (m, 2H), 7.40 – 7.31 (m, 6H), 5.76 (tt, *J* = 56.9, 4.5 Hz, 1H), 4.13 (t, *J* = 6.5 Hz, 2H), 3.19 (t, *J* = 7.4 Hz, 2H), 2.92 (t, *J* = 7.4 Hz, 2H), 1.78 (qdd, *J* = 13.1, 7.4, 3.1 Hz, 2H), 1.70 – 1.58 (m, 2H), 1.51 – 1.34 (m, 2H). **^{19}F NMR** (377 MHz, $CDCl_3$) δ -115.88 (dt, *J* = 56.8, 17.6 Hz). **^{13}C NMR** (126 MHz, $CDCl_3$) δ 172.12, 161.9, 145.6, 135.2, 132.5, 128.8, 128.7, 128.6, 128.3, 128.0, 126.6, 117.3 (t, *J* = 238.8 Hz), 64.7, 34.0 (t, *J* = 20.6 Hz), 31.3, 28.6, 25.6, 23.7, 21.9 (t, *J* = 5.6 Hz). HRMS-EI (m/z): Calcd for $C_{24}H_{25}F_2NO_3^+$ $[M]^+$ 413.1797, found 413.1805.

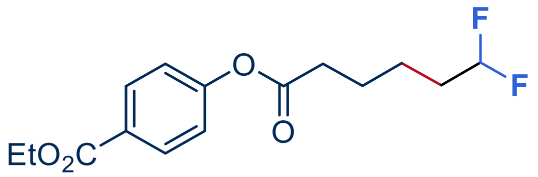

ethyl 4-((6,6-difluorohexanoyl)oxy)benzoate (**3f**)

This compound is a known compound tha has been reported in a previous study. [4] Following the general procedure **D**, the title compound (36.0 mg, colourless liquid) was obtained in 60% yield.

^{1}H NMR (400 MHz, $CDCl_3$) δ 8.22 – 7.93 (m, 2H), 7.18 – 7.13 (m, 2H), 5.84 (tt, *J* =

56.7, 4.4 Hz, 1H), 4.38 (q, *J* = 7.1 Hz, 2H), 2.62 (t, *J* = 7.4 Hz, 2H), 1.99 – 1.74 (m, 4H), 1.67 – 1.51 (m, 3H), 1.39 (t, *J* = 7.1 Hz, 3H). **^{19}F NMR** (377 MHz, $CDCl_3$) δ -116.03 (dt, *J* = 57.2, 17.6 Hz).

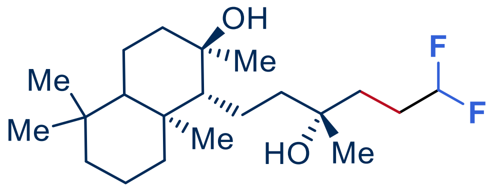

(1R,2R,8aS)-1-((R)-6,6-difluoro-3-hydroxy-3-methylhexyl)-2,5,5,8a-tetramethyldecahydronaphthalen-2-ol (**3g**)

This compound is a known compound tha has been reported in a previous study. [4] Following the general procedure **D**, the title compound (72.0 mg, colourless liquid) was obtained in 60% yield.

^{1}H NMR (400 MHz, $CDCl_3$) δ 5.84 (tt, *J* = 57.2, 4.5 Hz, 1H), 2.08 – 1.77 (m, 5H), 1.69 – 1.51 (m, 8H), 1.46 – 1.22 (m, 7H), 1.20 – 1.16 (m, 6H), 0.98 – 0.89 (m, 2H), 0.86 (s, 3H), 0.78 (d, *J* = 2.3 Hz, 6H). **^{19}F NMR** (377 MHz, $CDCl_3$) δ -115.55 (ddt, *J* = 56.6, 19.4, 17.7 Hz).

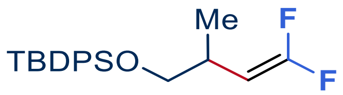

tert-butyl((4,4-difluoro-2-methylbut-3-en-1-yl)oxy)diphenylsilane (**3h**)

This compound is a known compound tha has been reported in a previous study. [6] Following the general procedure **E**, the title compound (26.6 mg, colourless liquid) was obtained in 37% yield.

^{1}H NMR (400 MHz, $CDCl_3$) δ 7.70 – 7.62 (m, 4H), 7.45 – 7.34 (m, 6H), 4.06 (ddd, *J* = 25.8, 9.7, 2.9 Hz, 1H), 3.50 (td, *J* = 6.4, 1.0 Hz, 2H), 2.57 (dddd, *J* = 14.1, 7.3, 4.3, 1.2 Hz, 1H), 1.06 (s, 9H), 1.04 (dt, *J* = 6.9, 0.8 Hz, 3H). **^{19}F NMR** (377 MHz, $CDCl_3$) δ -88.98 (d, *J* = 47.7 Hz), -90.19 (dd, *J* = 47.8, 25.8 Hz). **^{13}C NMR** (126 MHz, $CDCl_3$) δ 157.5 (d, *J* = 286.7 Hz), 135.7 (d, *J* = 2.6 Hz), 133.8 (d, *J* = 5.4 Hz), 129.8, 127.8, 81.2 (t, *J* = 20.4 Hz), 68.2 (t, *J* = 2.6 Hz), 31.4 (d, *J* = 4.2 Hz), 26.9, 19.4, 17.4 (t, *J* = 2.3 Hz).

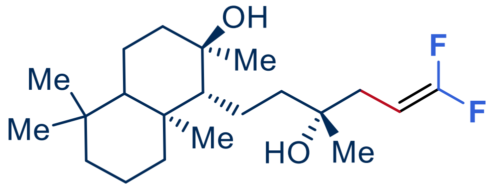

(1R,2R,8aS)-1-((R)-6,6-difluoro-3-hydroxy-3-methylhex-5-en-1-yl)-2,5,5,8a-tetramethyldecahydronaphthalen-2-ol (**3i**)

This compound is a known compound tha has been reported in a previous study. [5]

Following the general procedure **E**, the title compound (64.4 mg, white solid, m.p. 120.0-122.1 °C) was obtained in 90% yield.
^{1}H NMR (400 MHz, $CDCl_3$) δ 4.21 (dtd, *J* = 25.5, 8.1, 2.7 Hz, 1H), 2.18 – 2.09 (m, 2H), 2.04 (dt, *J* = 8.1, 1.8 Hz, 2H), 1.77 (dt, *J* = 12.1, 3.2 Hz, 1H), 1.61 – 1.42 (m, 6H), 1.37 – 1.14 (m, 7H), 1.10 (d, *J* = 1.4 Hz, 7H), 0.91 – 0.84 (m, 1H), 0.79 (s, 3H), 0.72 (d, *J* = 2.1 Hz, 6H). **^{19}F NMR** (377 MHz, $CDCl_3$) δ -87.38 (dd, *J* = 45.5, 2.4 Hz), -90.86 (dd, *J* = 45.9, 25.4 Hz). **^{13}C NMR** (126 MHz, $CDCl_3$) δ 158.2 (dd, *J* = 287.4, 285.4 Hz), 75.1, 74.4 (dd, *J* = 22.8, 21.2 Hz), 73.0, 61.9, 56.2, 44.5, 44.3, 42.1, 39.7, 39.4, 36.3 (d, *J* = 3.9 Hz), 33.5, 33.4, 25.7, 24.5, 21.6, 20.6, 18.9, 18.5, 15.5.

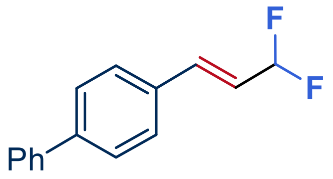

(*Z*)-4-(3,3-difluoroprop-1-en-1-yl)-1,1'-biphenyl (**3j**)
Following the general procedure **E**, the title compound (41.4 mg, white solid, m.p. 121.5-122.6 °C) was obtained in 95% yield.
^{1}H NMR (400 MHz, $CDCl_3$) δ 7.63 (dd, *J* = 7.6, 1.5 Hz, 4H), 7.57 – 7.45 (m, 4H), 7.44 – 7.36 (m, 1H), 6.99 – 6.87 (m, 1H), 6.46 – 6.09 (m, 2H). **^{19}F NMR** (377 MHz, $CDCl_3$) δ -109.33 – -109.49 (m), -109.51 – -109.61 (m). **^{13}C NMR** (126 MHz, $CDCl_3$) δ 142.3, 140.4, 136.8 (t, *J* = 12.2 Hz), 133.5, 129.0, 127.9, 127.6, 127.1, 121.0 (t, *J* = 23.9 Hz), 115.6 (t, *J* = 233.6 Hz). HRMS-EI (m/z): Calcd for $C_{13}H_{18}F_2^+$ $[M]^+$ 230.0902, found 230.0896.

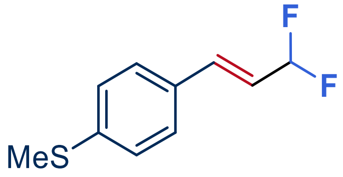

(*E*)-(4-(3,3-difluoroprop-1-en-1-yl)phenyl)(methyl)sulfane (**3k**)
Following the general procedure **E**, the title compound (36.8 mg, white solid, m.p. 50.5-51.3 °C) was obtained in 92% yield.
^{1}H NMR (400 MHz, $CDCl_3$) δ 7.64 – 7.30 (m, 2H), 7.29 – 7.14 (m, 2H), 6.95 – 6.65 (m, 1H), 6.50 – 5.91 (m, 2H), 2.50 (s, 3H). **^{19}F NMR** (377 MHz, $CDCl_3$) δ -109.22 – -109.34 (m), -109.41 – -109.46 (m). **^{13}C NMR** (126 MHz, $CDCl_3$) δ 140.7, 136.6 (t, *J* = 12.2 Hz), 131.2, 127.7, 126.4, 120.2 (t, *J* = 23.9 Hz), 115.6 (t, *J* = 233.6 Hz), 15.5. HRMS-EI (m/z): Calcd for $C_{15}H_{12}F_2^+$ $[M]^+$ 200.0466, found 200.0463.

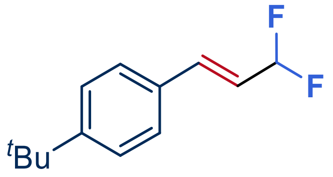

(*E*)-1-(tert-butyl)-4-(3,3-difluoroprop-1-en-1-yl)benzene (**3l**)

Following the general procedure **E**, the title compound (39.9 mg, colourless liquid) was obtained in 95% yield.

^{1}H NMR (400 MHz, $CDCl_3$) δ 7.47 – 7.30 (m, 4H), 6.95 – 6.80 (m, 1H), 6.44 – 6.06 (m, 2H), 1.35 (s, 9H). **^{19}F NMR** (377 MHz, $CDCl_3$) δ -109.07 – -109.17 (m), -109.23 – -109.30 (m).**^{13}C NMR** (126 MHz, $CDCl_3$) δ 152.9, 137.1 (t, *J* = 12.2 Hz), 131.8, 127.2, 125.9, 120.3 (t, *J* = 23.9 Hz), 115.8 (t, *J* = 233.3 Hz), 34.9, 31.3. HRMS-EI (m/z): Calcd for $C_{13}H_{16}F_2^+$ $[M]^+$ 210.1215, found 210.1215.

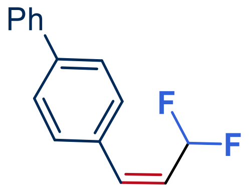

(*Z*)-4-(3,3-difluoroprop-1-en-1-yl)-1,1'-biphenyl (**3m**)

Following the general procedure **F**, the title compound (37.3 mg, Z/E = 1.7/1) was obtained in 81% yield.

^{1}H NMR (400 MHz, $CDCl_3$) δ 7.73 – 7.55 (m, 4H), 7.53 – 7.44 (m, 2H), 7.44 – 7.37 (m, 3H), 7.03 – 6.92 (m, 1H), 6.43 (tdd, *J* = 55.4, 7.4, 0.9 Hz, 1H), 5.91 (dtd, *J* = 11.8, 8.5, 7.4 Hz, 1H). **^{19}F NMR** (471 MHz, $CDCl_3$) δ -107.91 (d, *J* = 8.4 Hz), -108.02 (d, *J* = 8.7 Hz). **^{13}C NMR** (126 MHz, $CDCl_3$) δ 141.7, 140.3, 137.7 (t, *J* = 12.7 Hz), 133.5 (t, *J* = 2.1 Hz), 129.4, 129.0, 127.9, 127.5, 127.2, 123.9 (t, *J* = 26.2 Hz), 112.5 (t, *J* = 230.6 Hz). HRMS-EI (m/z): Calcd for $C_{15}H_{12}F_2^+$ $[M]^+$ 230.0902, found 230.0906.

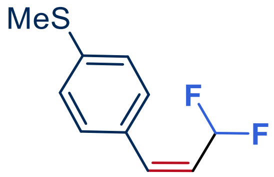

(*Z*)-(4-(3,3-difluoroprop-1-en-1-yl)phenyl)(methyl)sulfane (**3n**)

Following the general procedure **F**, the title compound (28.0 mg, Z/E = 2.6/1) was obtained in 70% yield.

^{1}H NMR (400 MHz, $CDCl_3$) δ 7.37 – 7.02 (m, 4H), 6.89 – 6.72 (m, 1H), 6.26 (tdd, *J* = 55.4, 7.3, 0.9 Hz, 1H), 5.75 (dtd, *J* = 11.8, 8.6, 7.3 Hz, 1H), 2.43 (s, 3H). **^{19}F NMR** (377 MHz, $CDCl_3$) δ -107.75 (d, *J* = 8.7 Hz), -107.90 (d, *J* = 8.8 Hz). ^{13}C NMR (126 MHz, $CDCl_3$) δ 140.0, 137.5 (t, *J* = 12.6 Hz), 131.1, 129.3, 126.3, 123.4 (t, *J* = 26.1 Hz), 112.5 (t, *J* = 230.6 Hz), 15.5. HRMS-EI (m/z): Calcd for $C_{10}H_{10}F_2S^+$ $[M]^+$ 200.0466, found 200.0466.

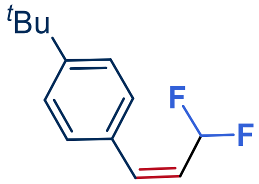

(*Z*)-1-(tert-butyl)-4-(3,3-difluoroprop-1-en-1-yl)benzene (**3o**)

Following the general procedure **F**, the title compound (31.5 mg, Z/E = 4.0/1) was obtained in 75% yield.

^{1}H NMR (400 MHz, $CDCl_3$) δ 7.51 – 7.27 (m, 2H), 7.24 – 7.05 (m, 2H), 6.84 (dd, *J* = 11.7, 1.0 Hz, 1H), 6.30 (tdd, *J* = 55.5, 7.4, 0.9 Hz, 1H), 5.74 (dtd, *J* = 11.8, 8.5, 7.4 Hz, 1H), 1.26 (s, 9H). **^{19}F NMR** (377 MHz, $CDCl_3$) δ -107.85 (d, *J* = 8.3 Hz), -108.00 (d, *J* = 8.7 Hz). **^{13}C NMR** (101 MHz, $CDCl_3$) δ 152.1, 138.0 (t, *J* = 12.6 Hz), 131.8 (t, *J* = 2.2 Hz), 128.8 (d, *J* = 1.8 Hz), 125.7, 123.3 (t, *J* = 26.1 Hz), 112.6 (t, *J* = 230.3 Hz), 34.8, 31.4. HRMS-EI (m/z): Calcd for $C_{13}H_{16}F_2^+$ $[M]^+$ 210.1220, found 210.1223.

IX. Supplementary references

[1] E. Falk, S. Makai, T. Delcaillau, L. Guertler, B. Morandi, Design and scalable synthesis of N-alkylhydroxylamine reagents for the direct Iron-catalyzed installation of medicinally relevant amines. *Angew. Chem., Int. Ed.* **2020**, *59*, 21064.

[2] D. P. Wood, W. Guan, S. Lin, Titanium and cobalt bimetallic radical redox relay for the isomerization of N-Bz aziridines to allylic amides. *Synthesis* **2021**, *53*, 4213.

[3] H. Huang, C. Yu, Y. Zhang, Y. Zhang, P. S. Mariano, W. Wang, Chemo- and Regioselective Organo-Photoredox catalyzed hydroformylation of styrenes via a radical pathway. *J. Am. Chem. Soc.* **2017**, *139*, 9799.

[4] D. Meng, L. Li, A. Brown, J.-N. Desrosiers, S. Duan, C. M. Hayward, Z. He, J. Hu, T. Makowski, M. Maloney, S. Monfette, H. Perfect, J. L. Piper, M. D. Zhou, W. Widlicka, A radical chlorodifluoromethylation protocol for late-stage difluoromethylation and its application to an oncology candidate. *Cell Rep. Phys. Sci.* **2021**, *2*, 10349.

[5] Z.-Q. Zhang, Y.-Q. Sang, C.-Q. Wang, P. Dai, X.-S. Xue, J. L. Piper, Z.-H. Peng, J.-A. Ma, F.-G. Zhang, J. Wu, Difluoromethylation of Unactivated Alkenes Using Freon-22 through Tertiary Amine-Borane-Triggered Halogen Atom Transfer. *J. Am. Chem. Soc*. **2022**, *144*, 14288–14296.

[6] P. Salomon, S. Z. Zard, A Practical Source of Chlorodifluoromethyl Radicals. Convergent Routes to gem-Difluoroalkenes and -dienes and (2,2-Difluoroethyl)-indoles, -azaindoles, and -naphthols. *Org. Lett*. **2014**, *16*, 2926–2929.

[7] Corcoran, E. B., McMullen, J. P., Lévesque, F., Wismer, M. K. & Naber, J. R. Photon equivalents as a parameter for scaling photoredox reactions in flow: translation of S239 photocatalytic C−N cross-coupling from lab scale to multikilogram scale. *Angew. Chem. Int. Ed.* **2022**, *59*, 11964−11968.

[8] Demas, J. N., Bowman, W. D., Zalewski, E. F. & Velapoldi, R. A. Determination of the quantum yield of the ferrioxalate actinometer with electrically calibrated radiometers. *J. Phys. Chem.* **1981**, *85*, 2766−2771.

[9] Hatchard, C. G., Parker, C. A. & Bowen, E. J. A new sensitive chemical actinometer - II. Potassium ferrioxalate as a standard chemical actinometer. *Proc. R. Soc. Lond. A* **1956**, *235*, 518−536.

X. NMR spectra for product characterization

[1]**H NMR** (400 MHz, $CDCl_3$) spectra for compound **1a**

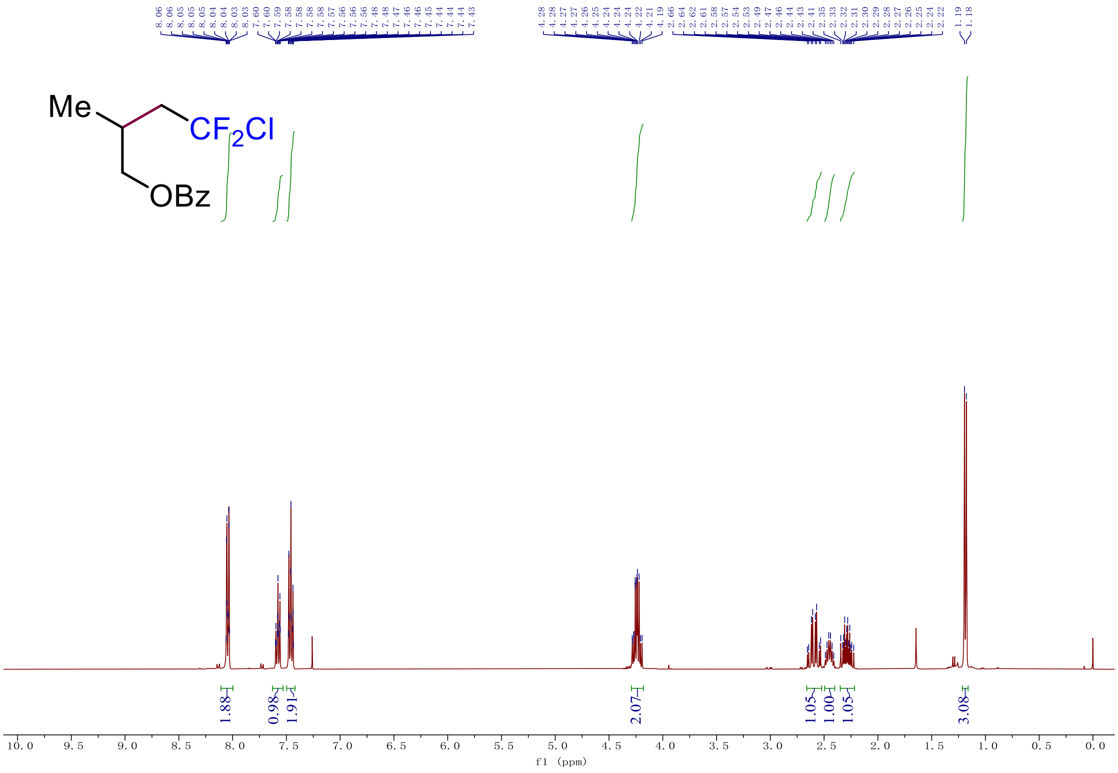

[19]**F NMR** (377 MHz, $CDCl_3$) spectra for compound **1a**

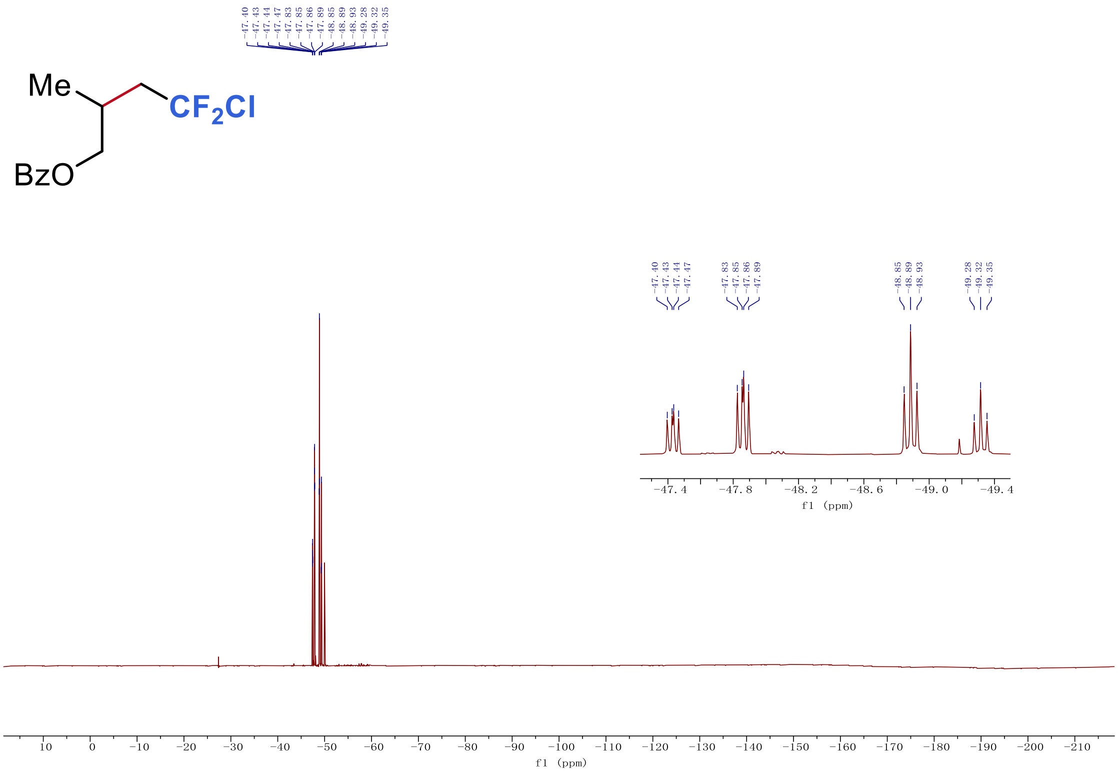

^{13}C NMR (126 MHz, $CDCl_3$) spectra for compound **1a**

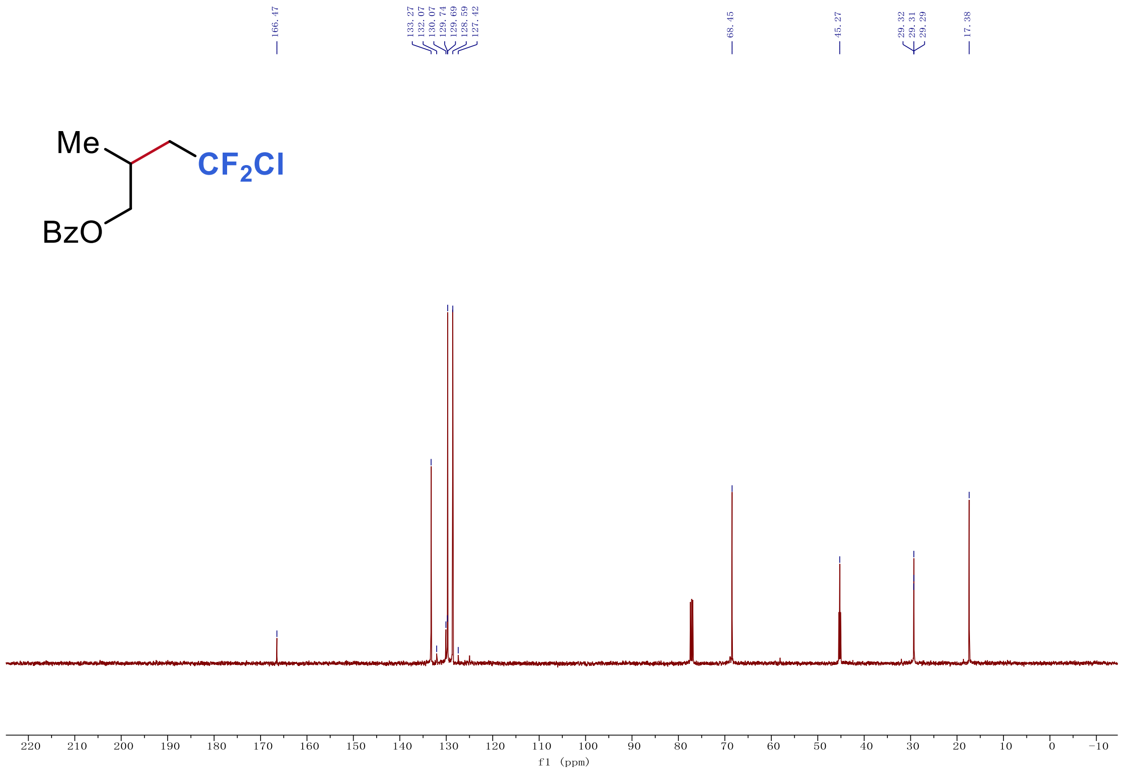

^{1}H NMR (400 MHz, $CDCl_3$) spectra for compound **1b**

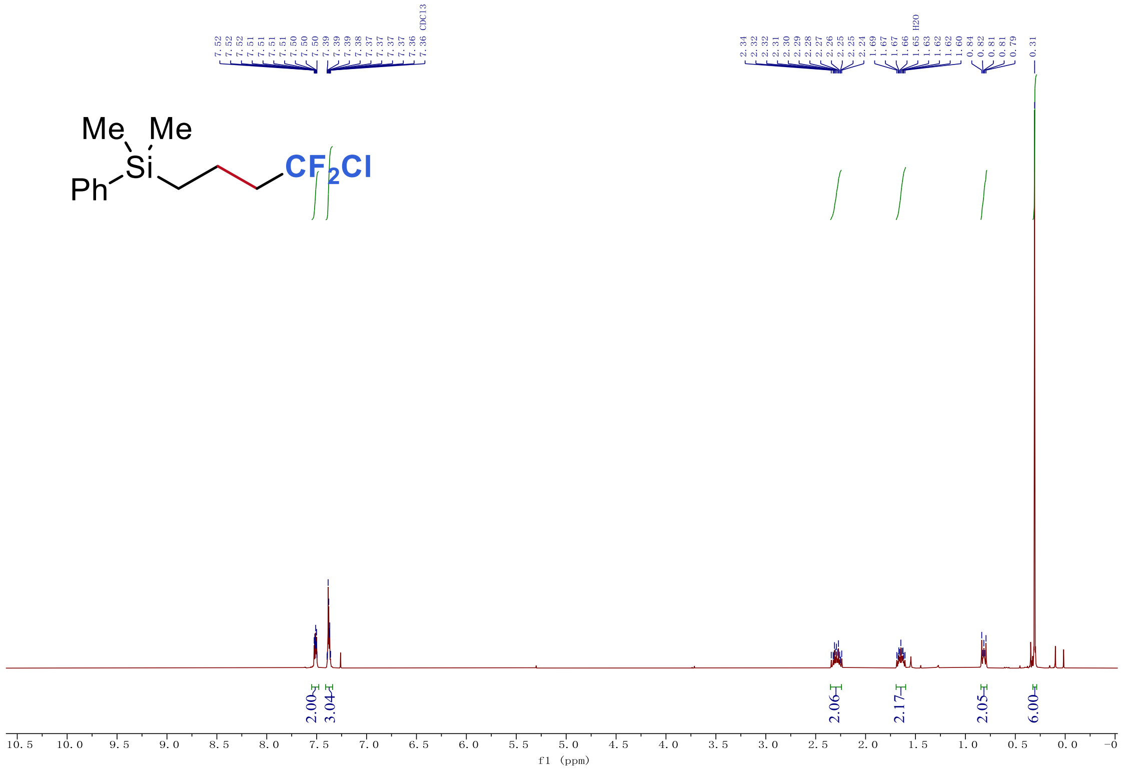

^{19}F NMR (377 MHz, $CDCl_3$) spectra for compound **1b**

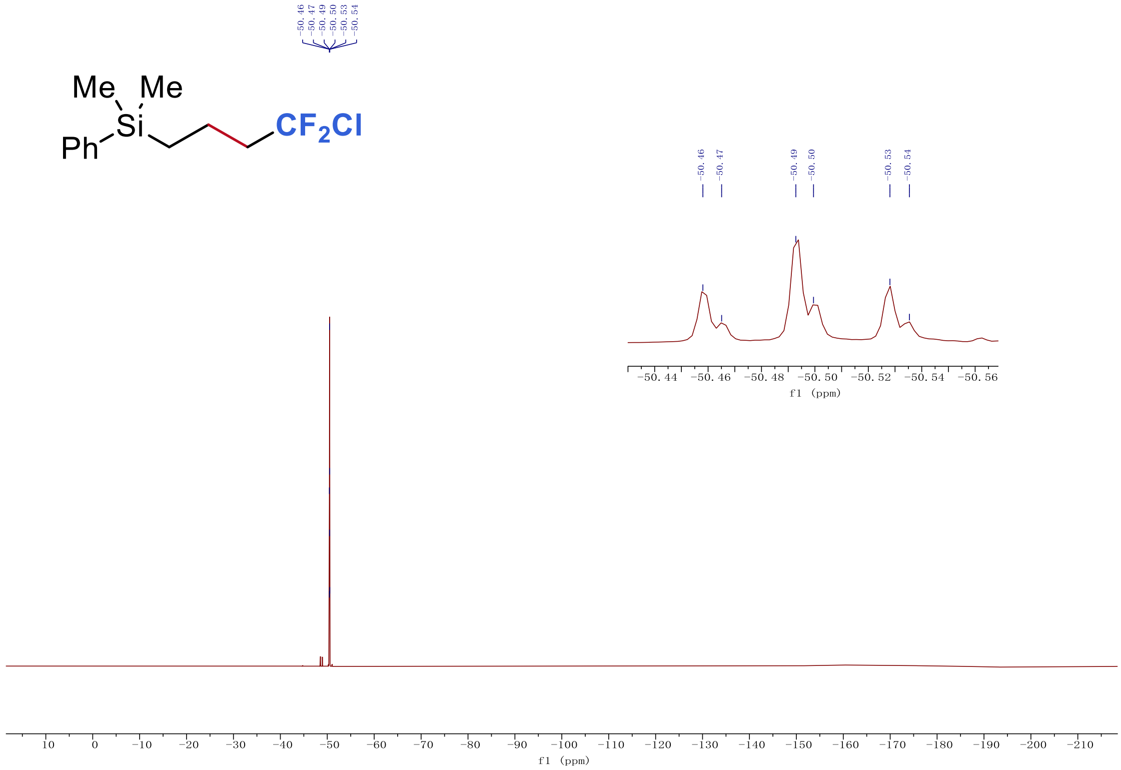

^{13}C NMR (126 MHz, $CDCl_3$) spectra for compound **1b**

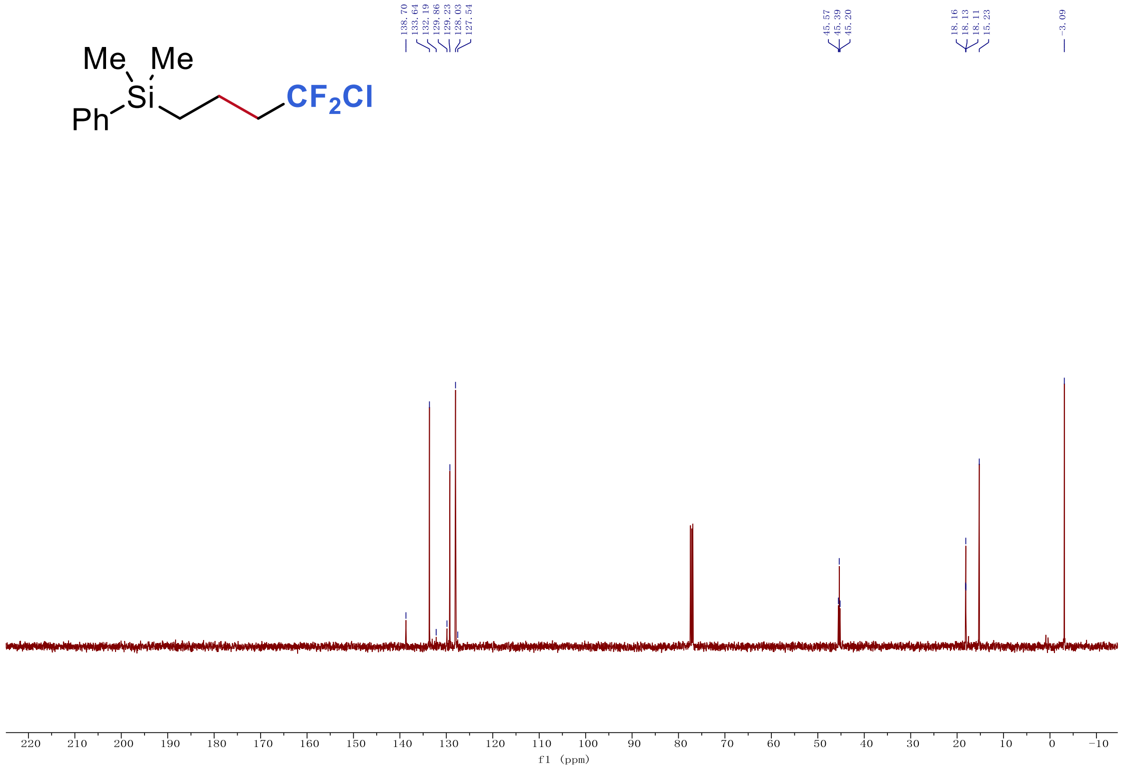

^{1}H NMR (400 MHz, $CDCl_3$) spectra for compound **1c**

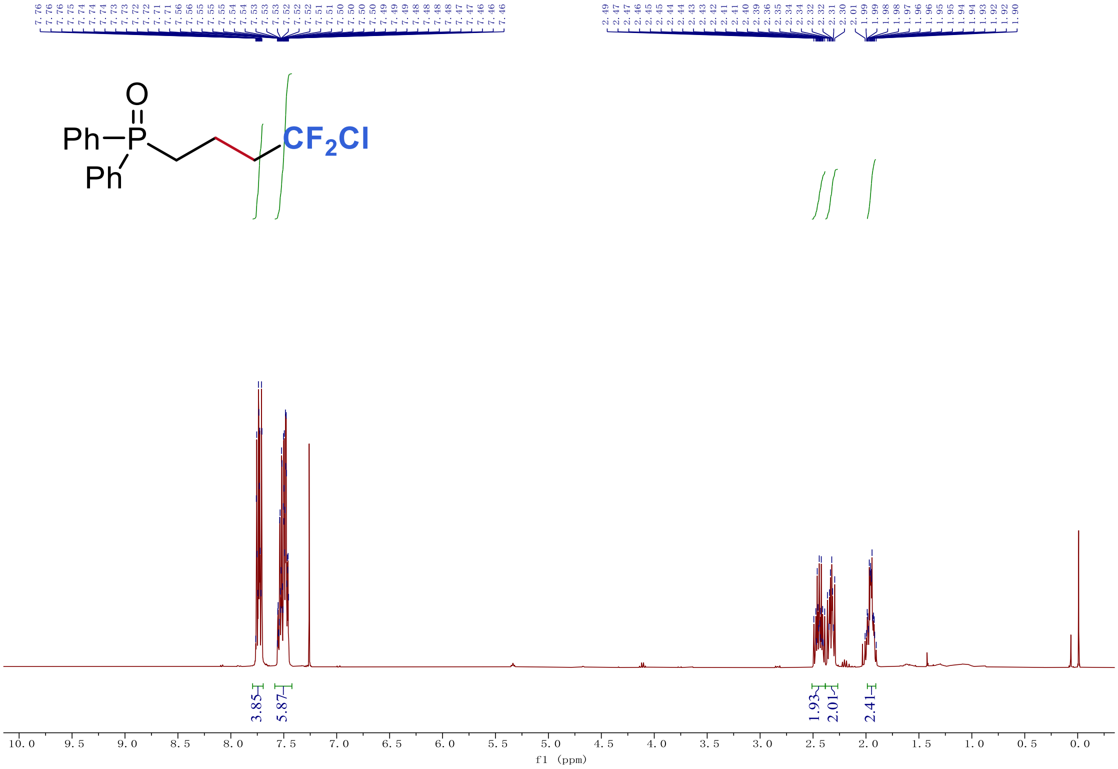

^{19}F NMR (377 MHz, $CDCl_3$) spectra for compound **1c**

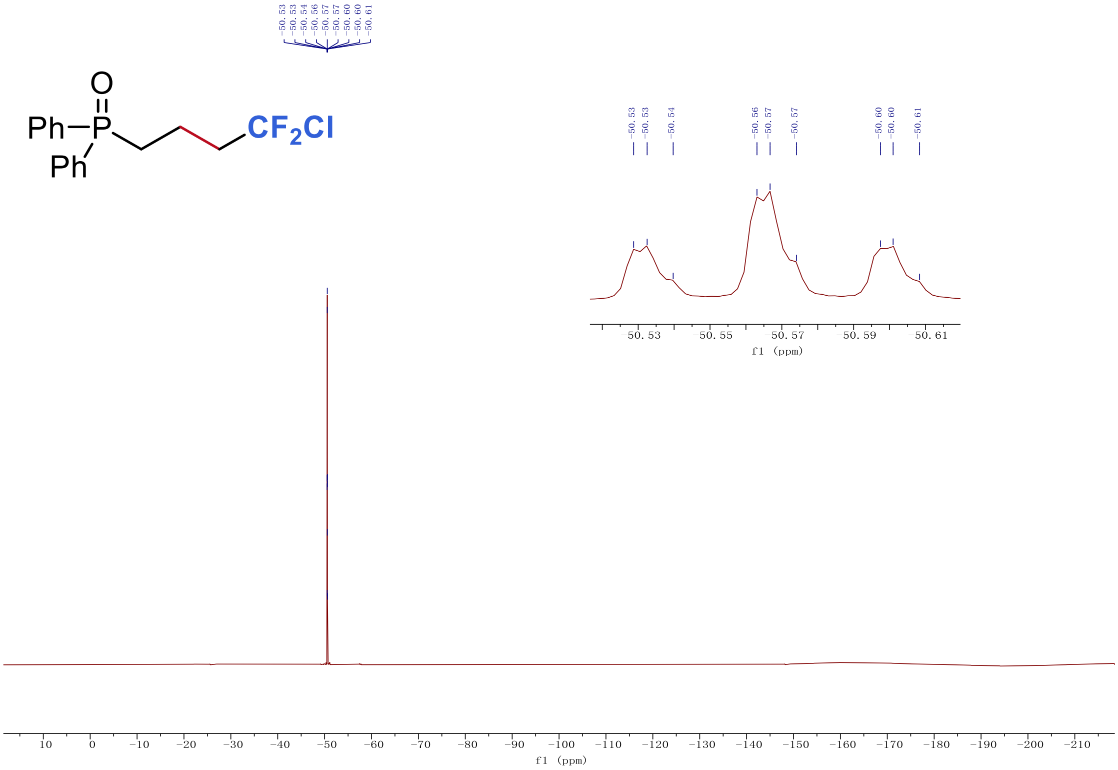

^{31}P NMR (162 MHz, $CDCl_3$) spectra for compound **1c**

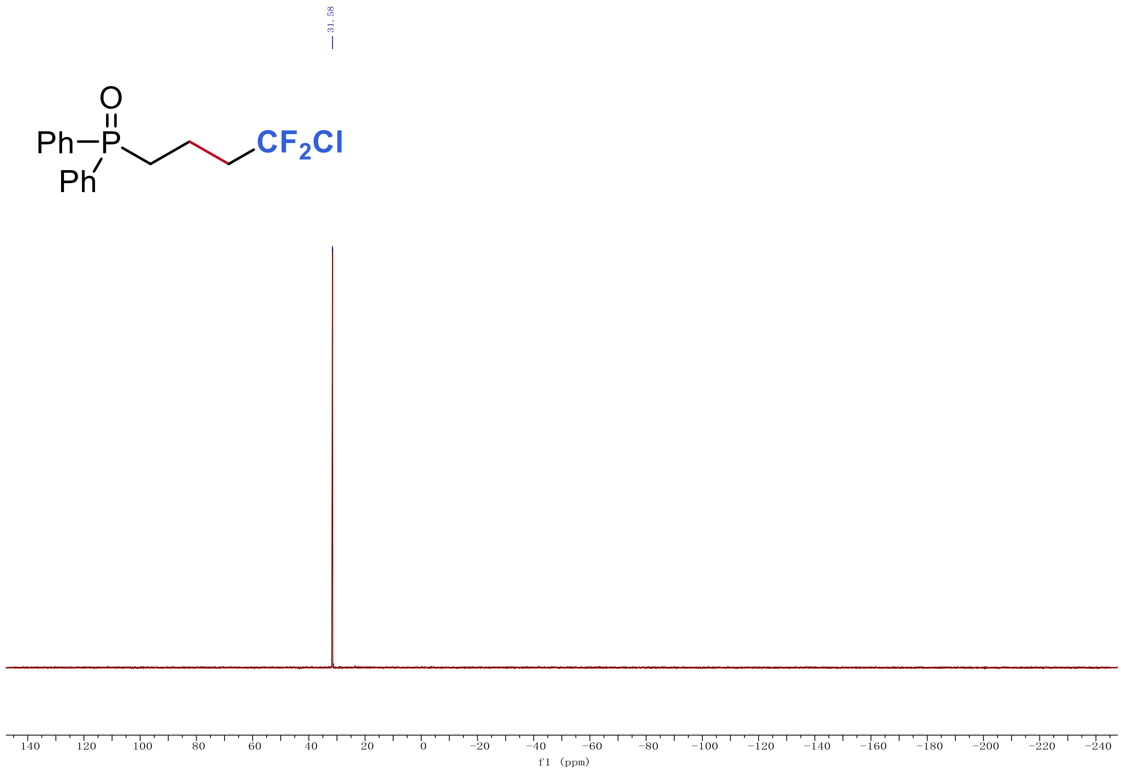

^{13}C NMR (126 MHz, $CDCl_3$) spectra for compound **1c**

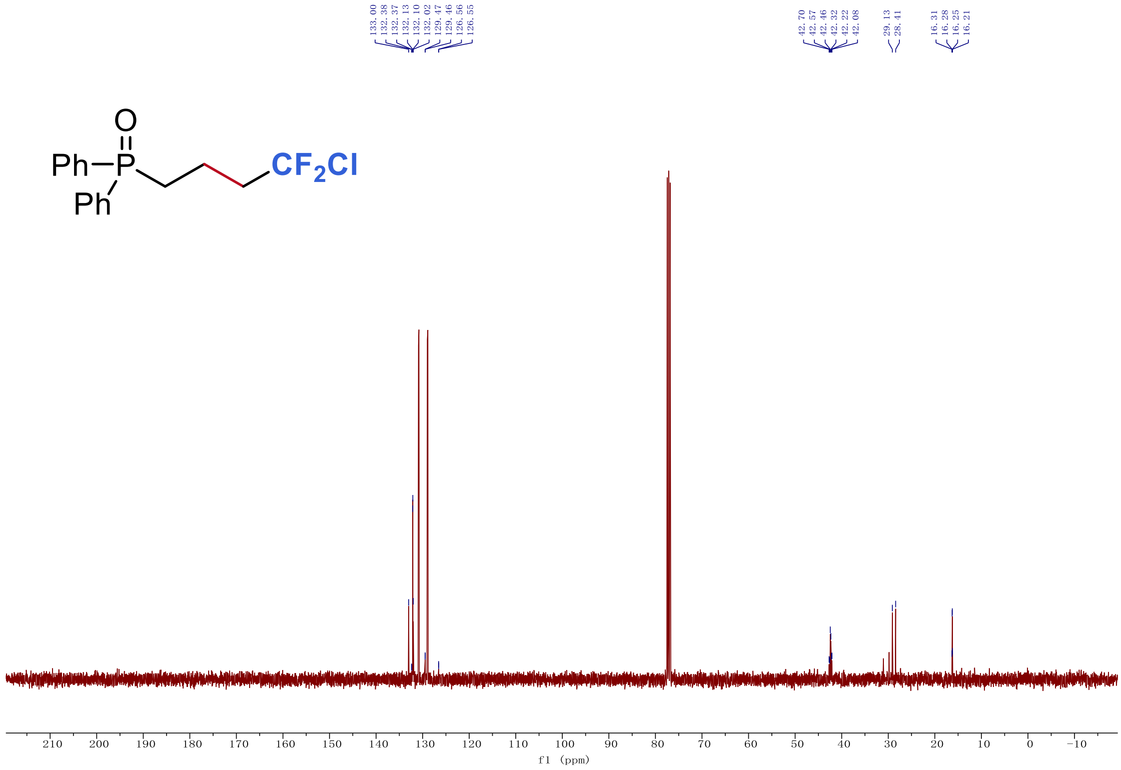

^{1}H NMR (400 MHz, $CDCl_3$) spectra for compound **1d**

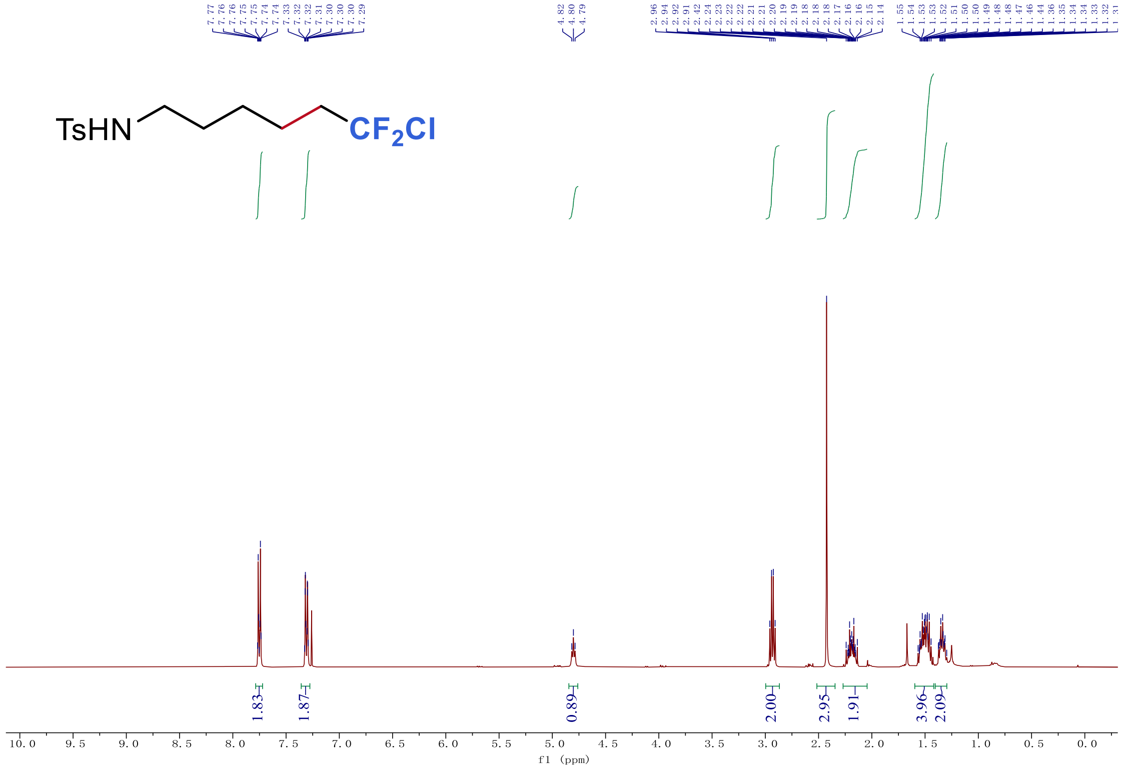

^{19}F NMR (377 MHz, $CDCl_3$) spectra for compound **1d**

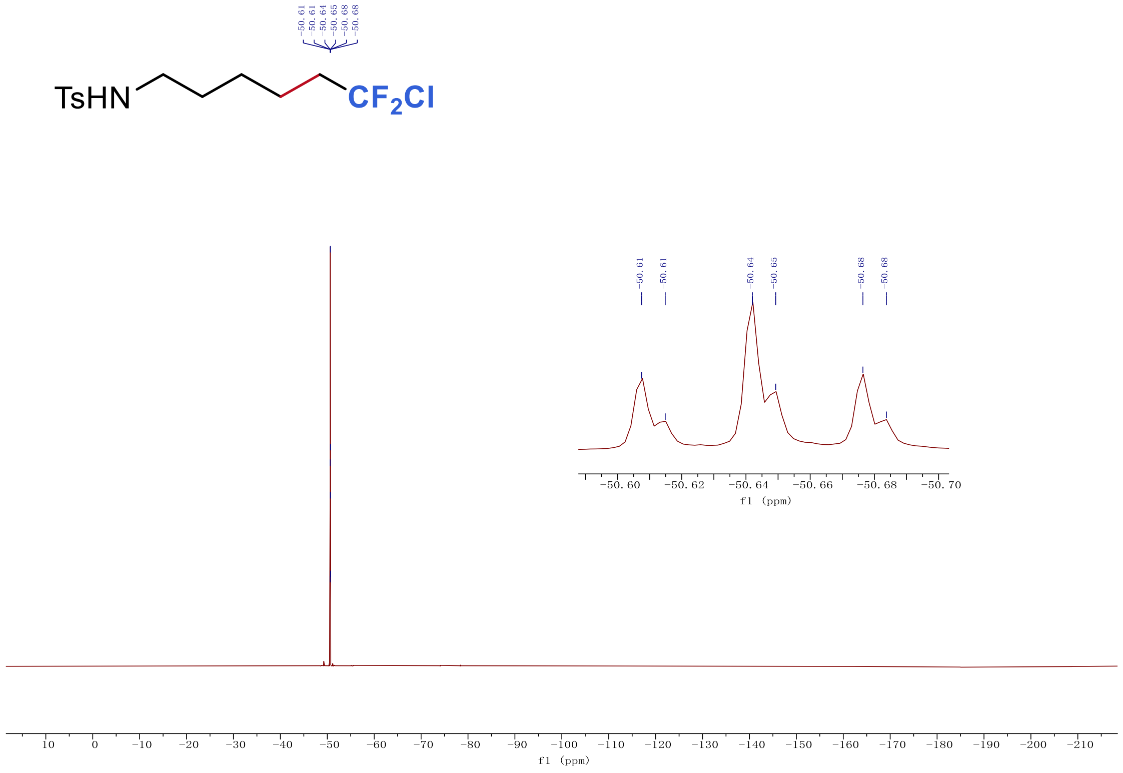

^{13}**C NMR** (126 MHz, $CDCl_3$) spectra for compound **1d**

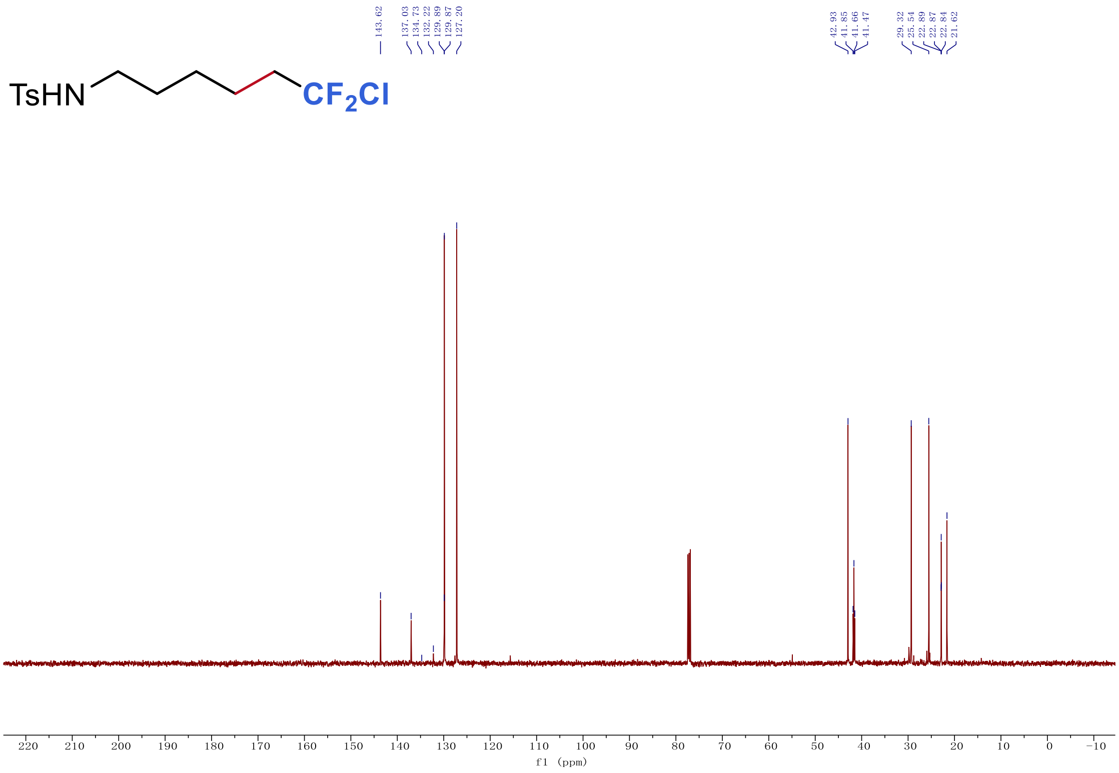

^{1}**H NMR** (400 MHz, $CDCl_3$) spectra for compound **1e**

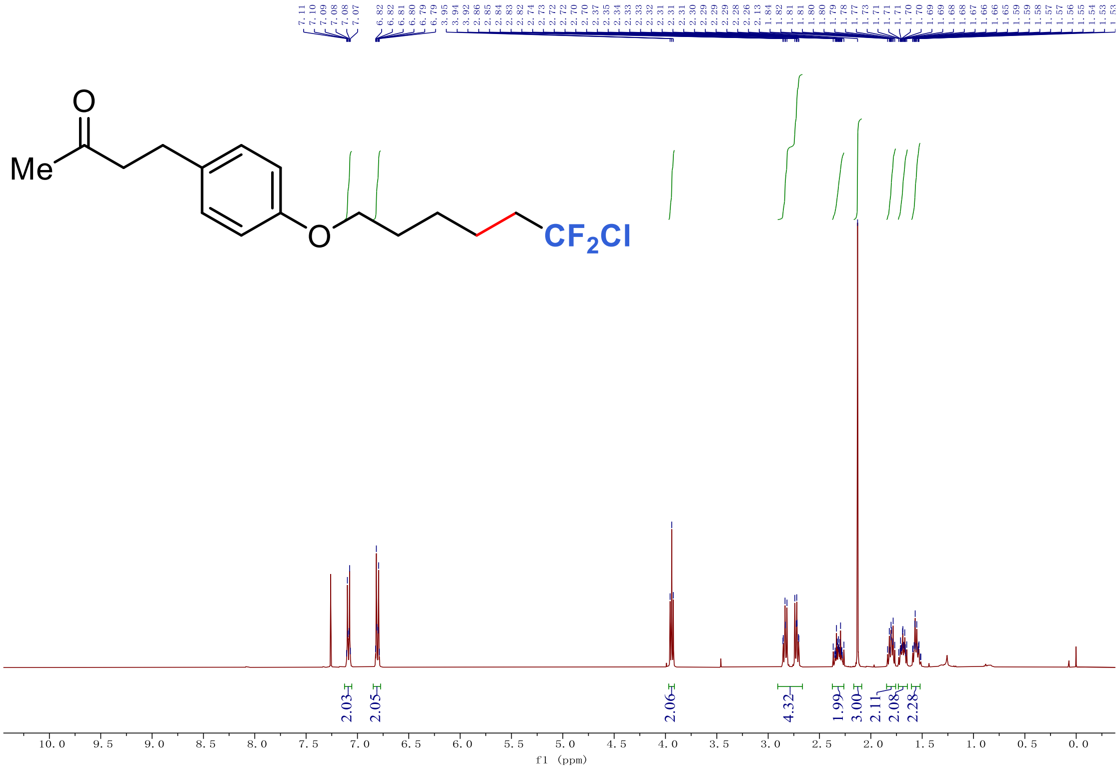

^{19}F NMR (377 MHz, $CDCl_3$) spectra for compound **1e**

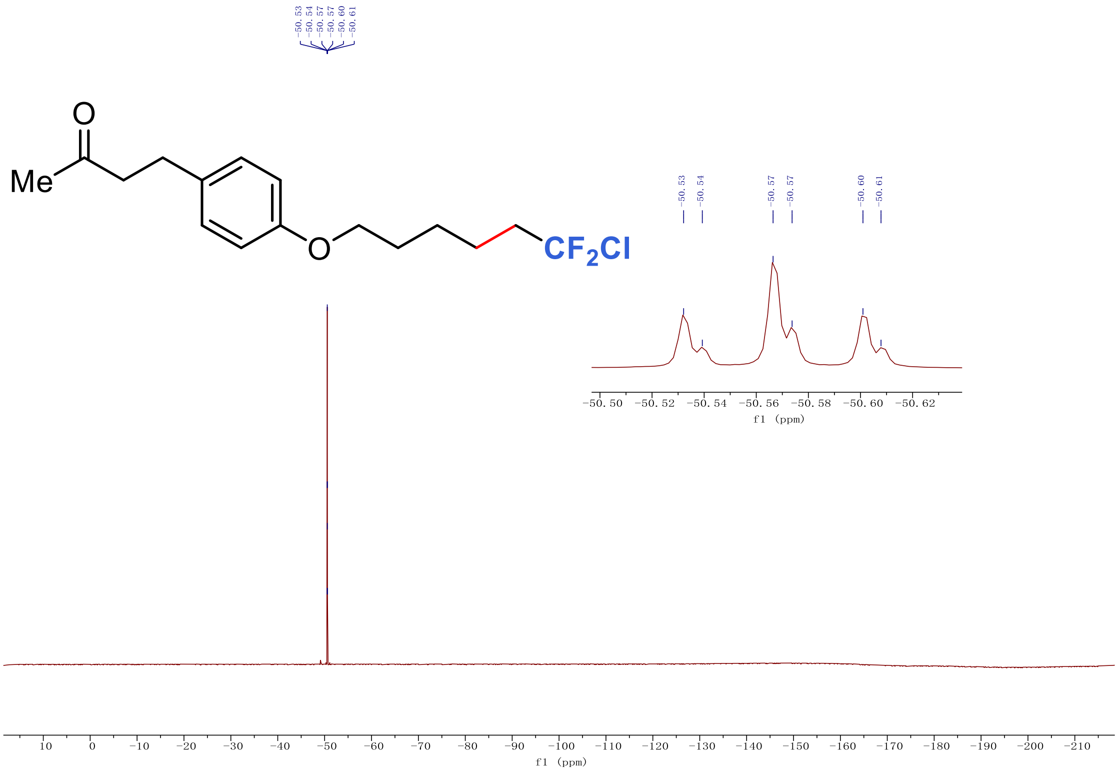

^{13}C NMR (126 MHz, $CDCl_3$) spectra for compound **1e**

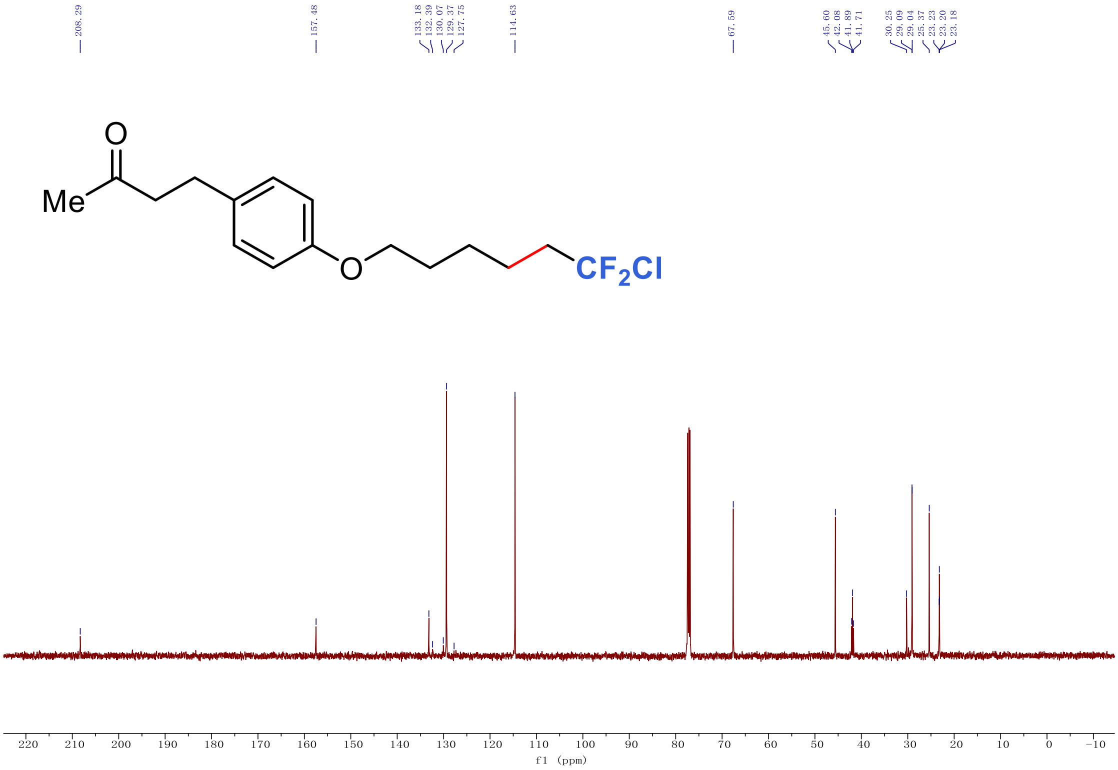

^{1}H NMR (400 MHz, $CDCl_3$) spectra for compound **1f**

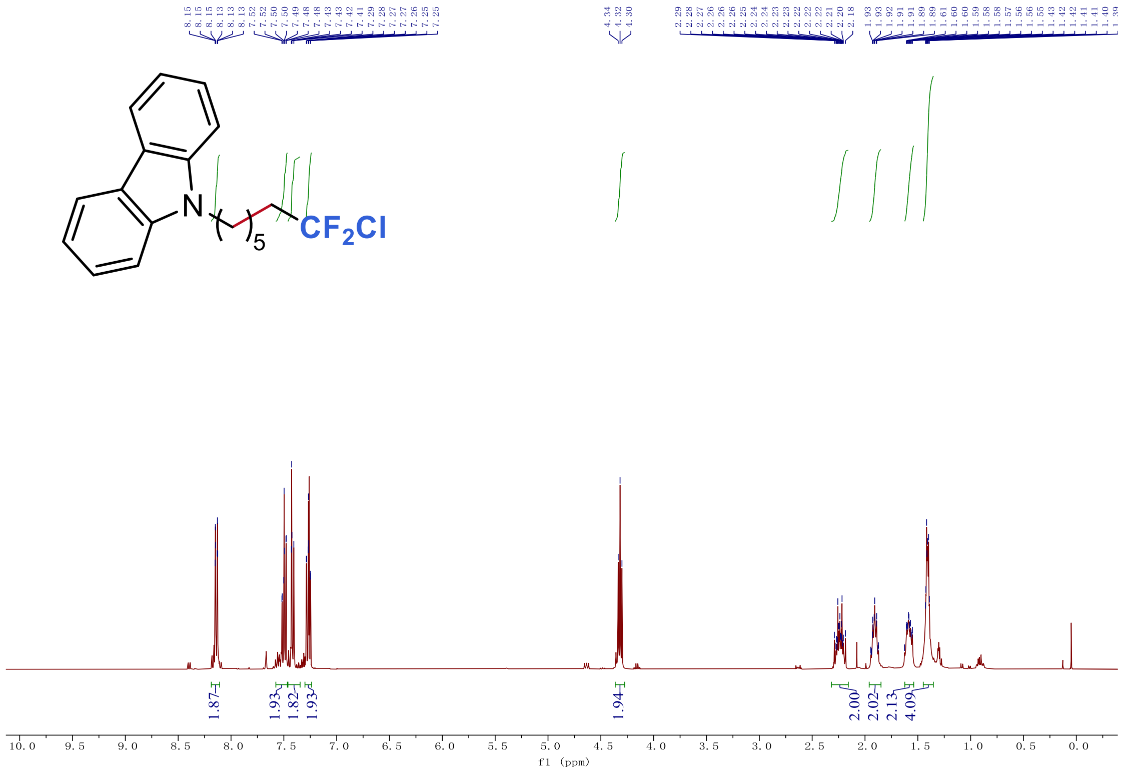

^{19}F NMR (377 MHz, $CDCl_3$) spectra for compound **1f**

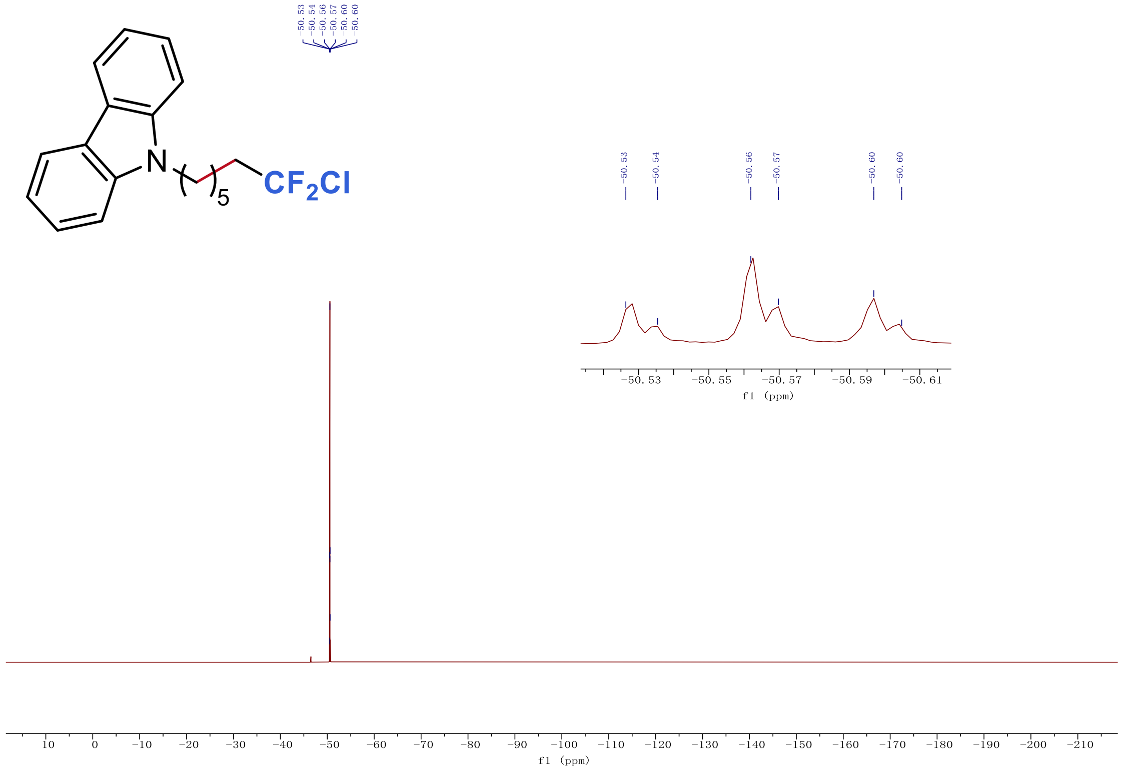

^{13}C NMR (126 MHz, $CDCl_3$) spectra for compound 1f

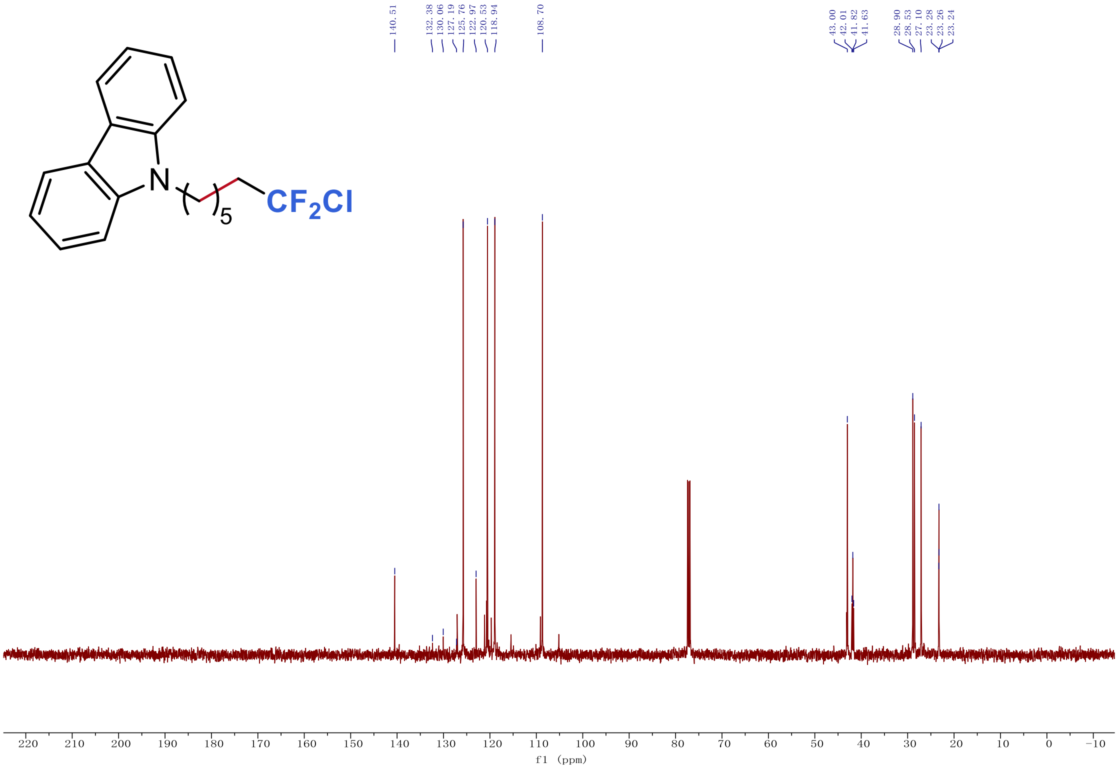

^{1}H NMR (400 MHz, $CDCl_3$) spectra for compound 1g

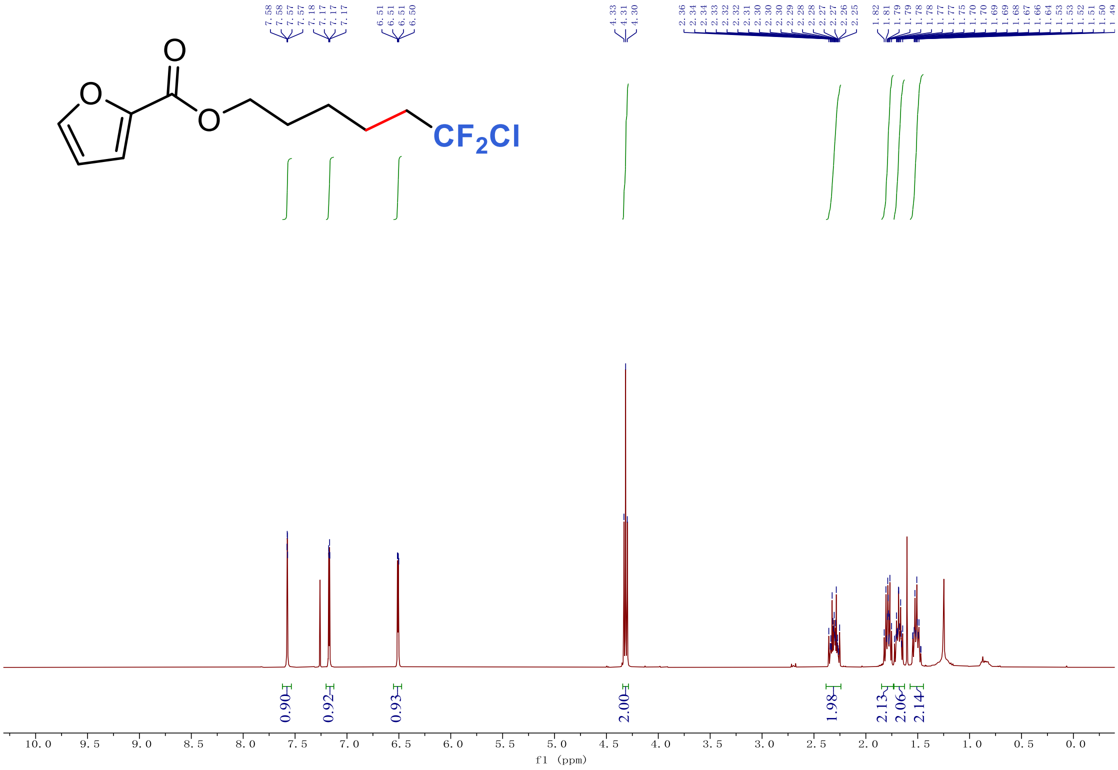

^{19}F NMR (377 MHz, $CDCl_3$) spectra for compound **1g**

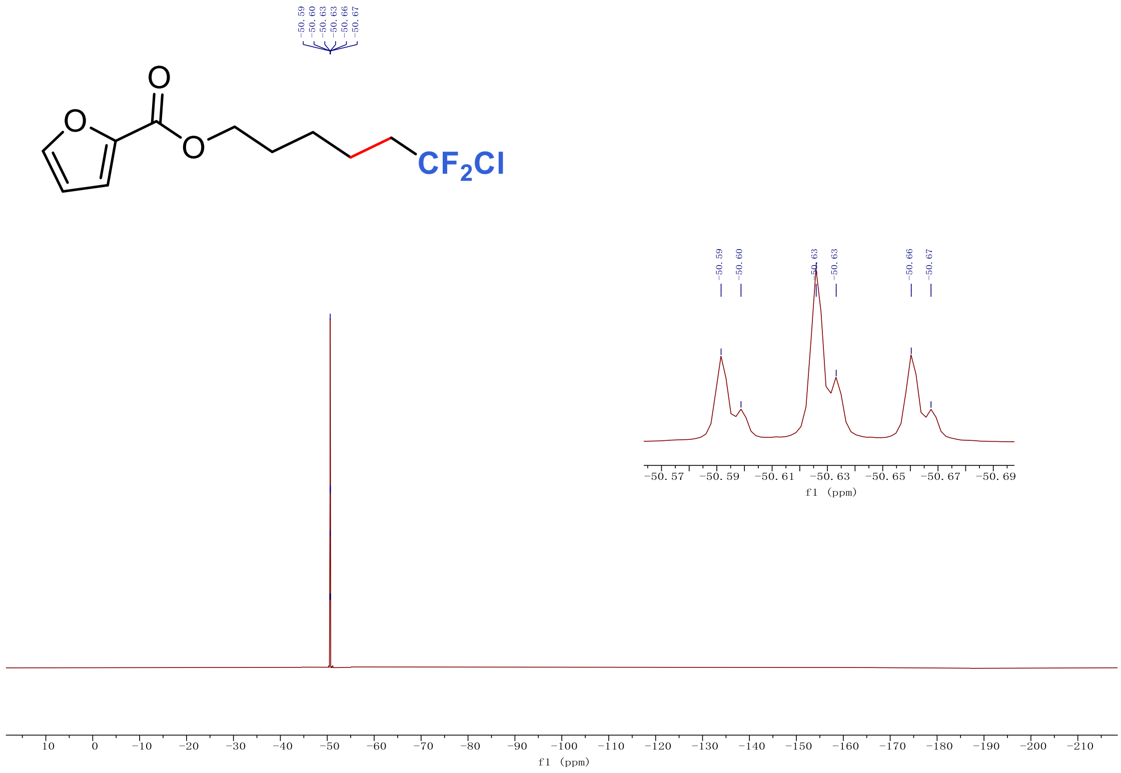

^{13}C NMR (126 MHz, $CDCl_3$) spectra for compound **1g**

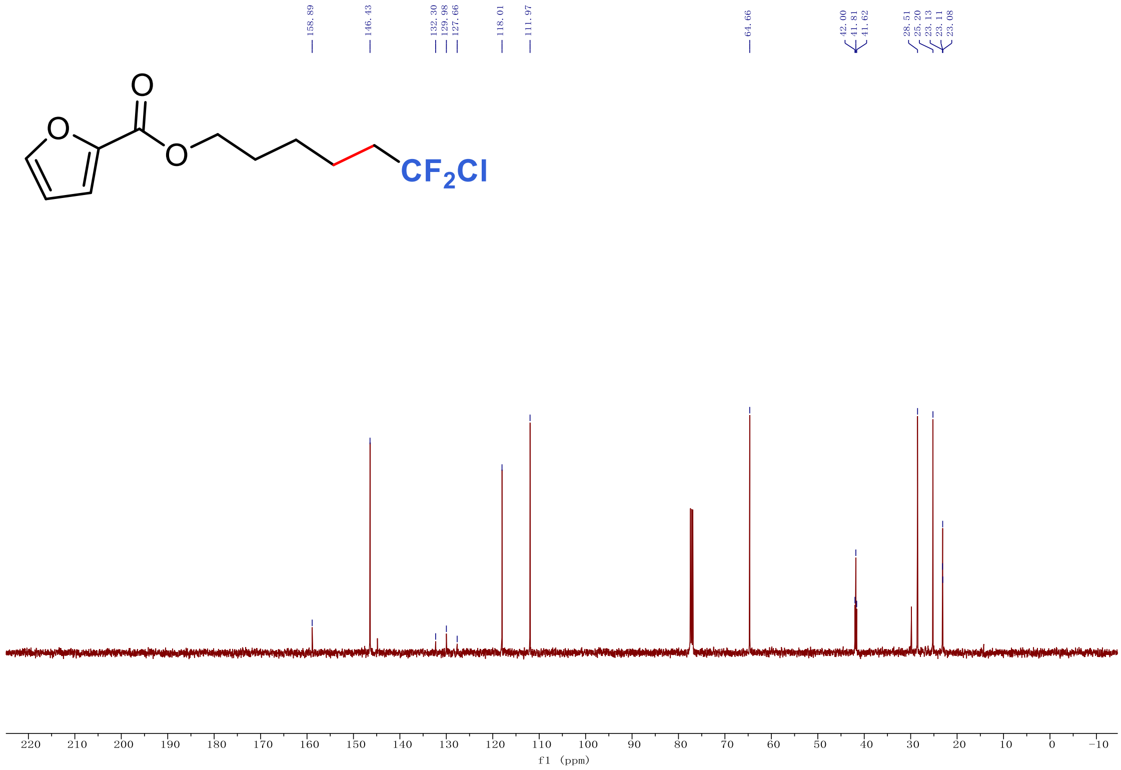

^{1}H NMR (400 MHz, $CDCl_3$) spectra for compound **1an**

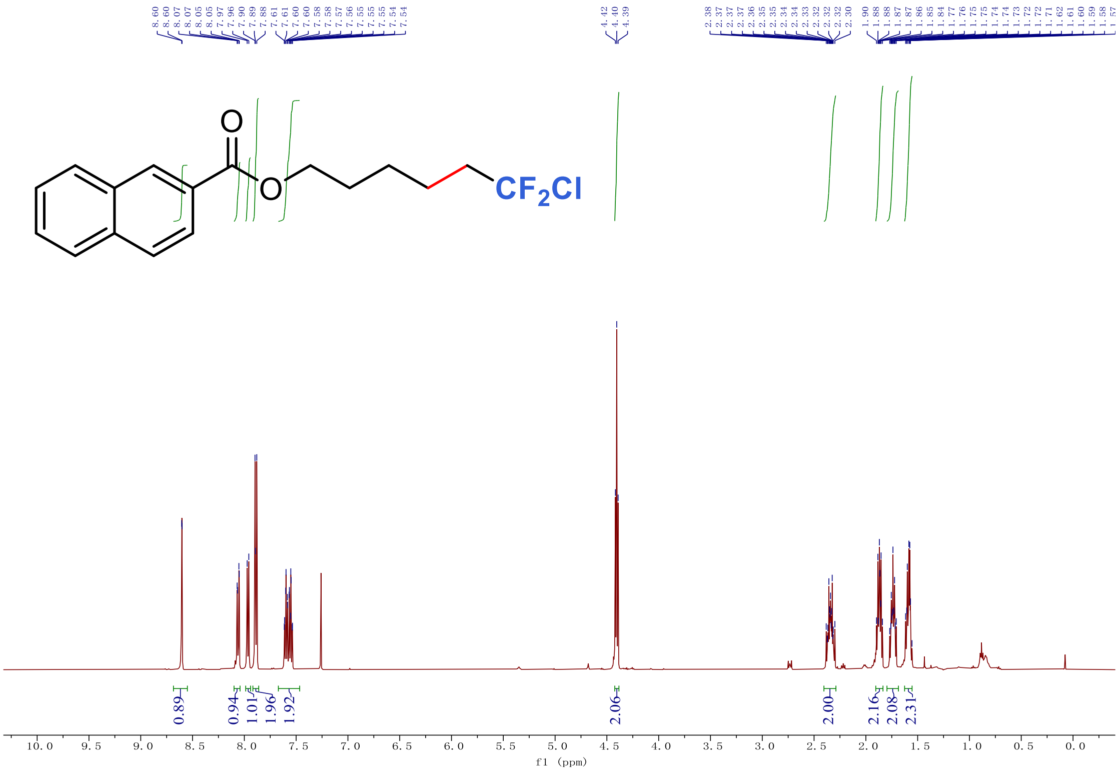

^{19}F NMR (377 MHz, $CDCl_3$) spectra for compound **1an**

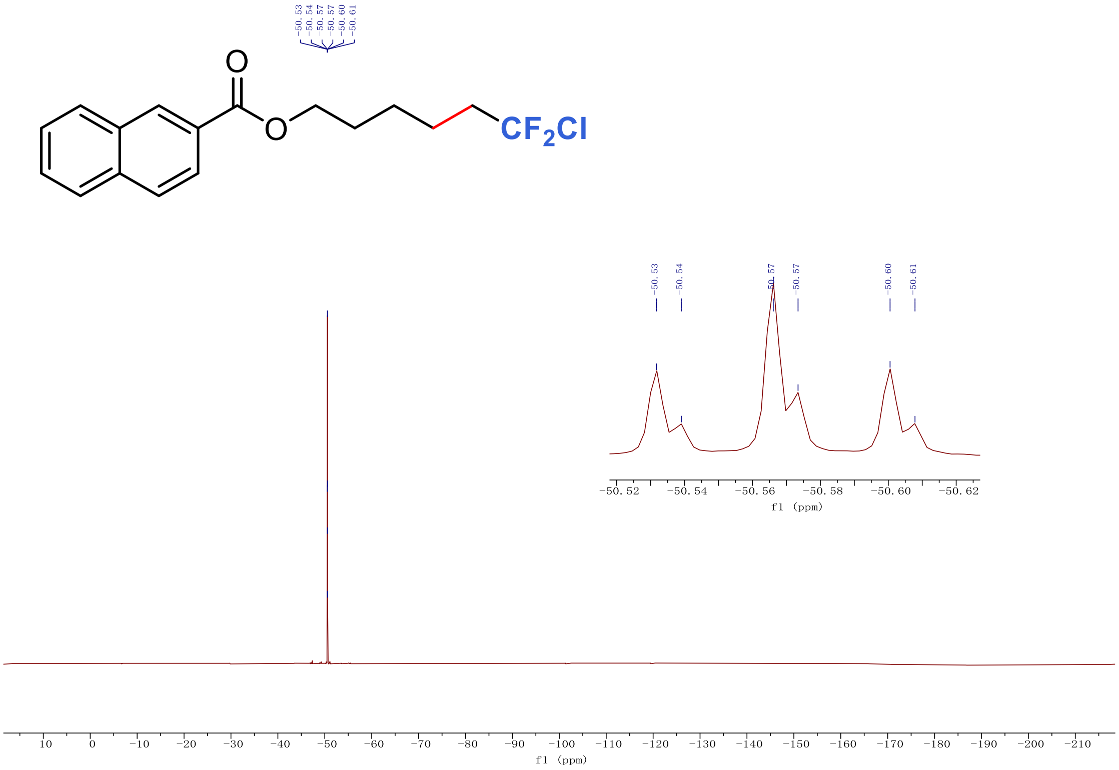

13C NMR (126 MHz, CDCl3) spectra for compound **1an**

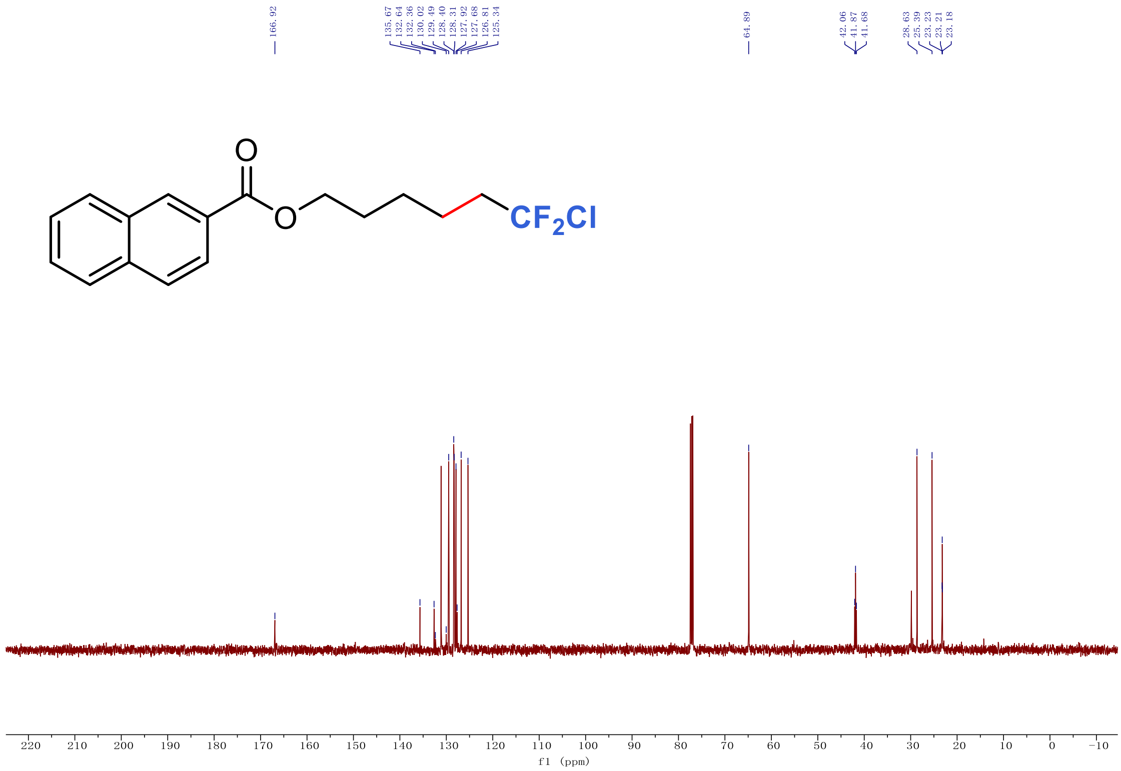

1H NMR (400 MHz, CDCl3) spectra for compound **1i**

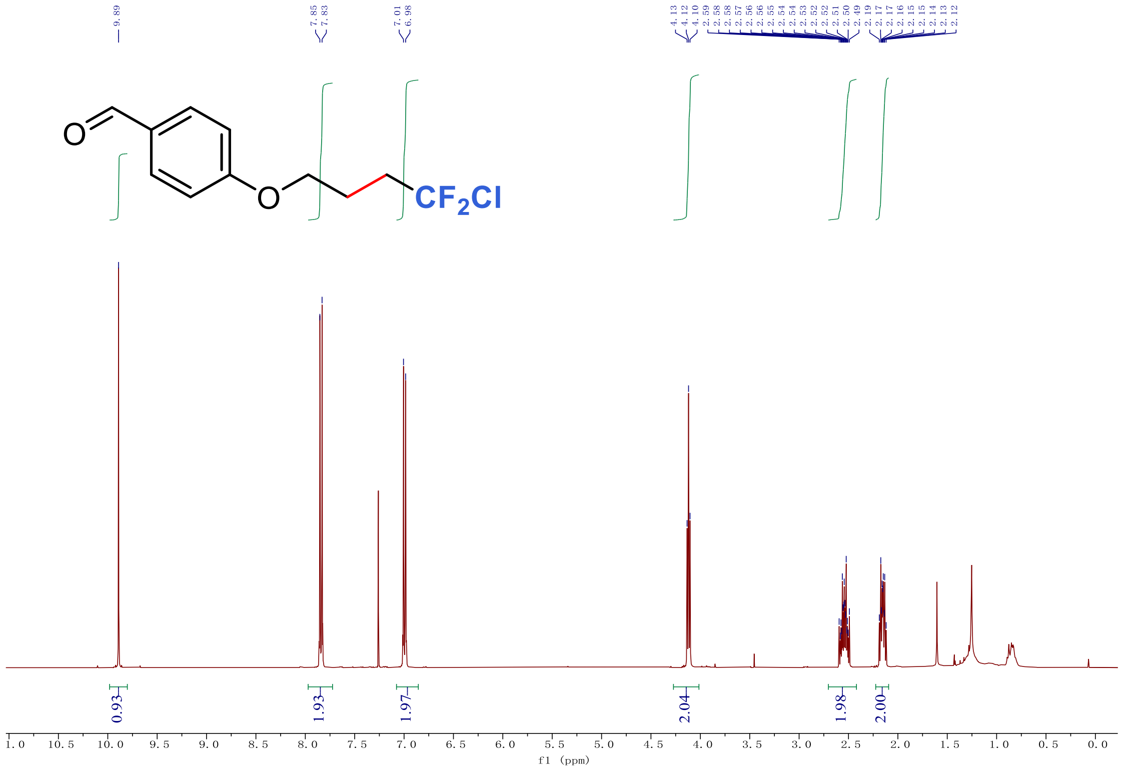

^{19}F NMR (377 MHz, $CDCl_3$) spectra for compound **1i**

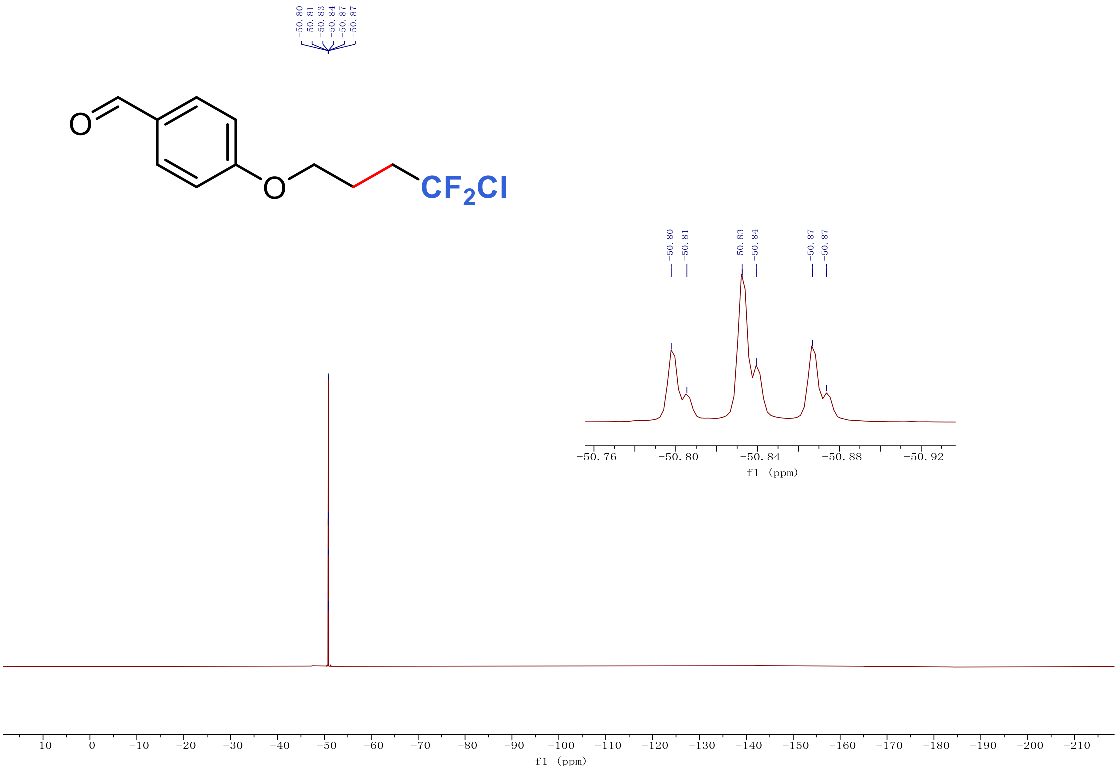

^{13}C NMR (126 MHz, $CDCl_3$) spectra for compound **1i**

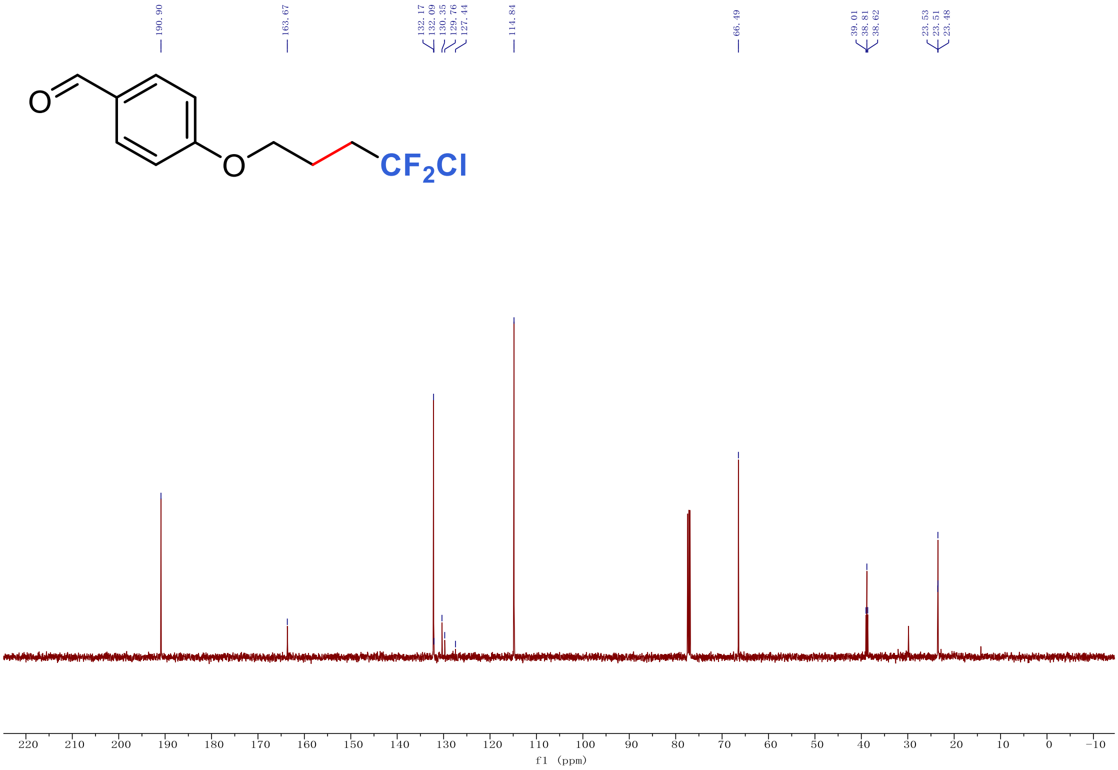

^{1}H NMR (400 MHz, $CDCl_3$) spectra for compound **1j**

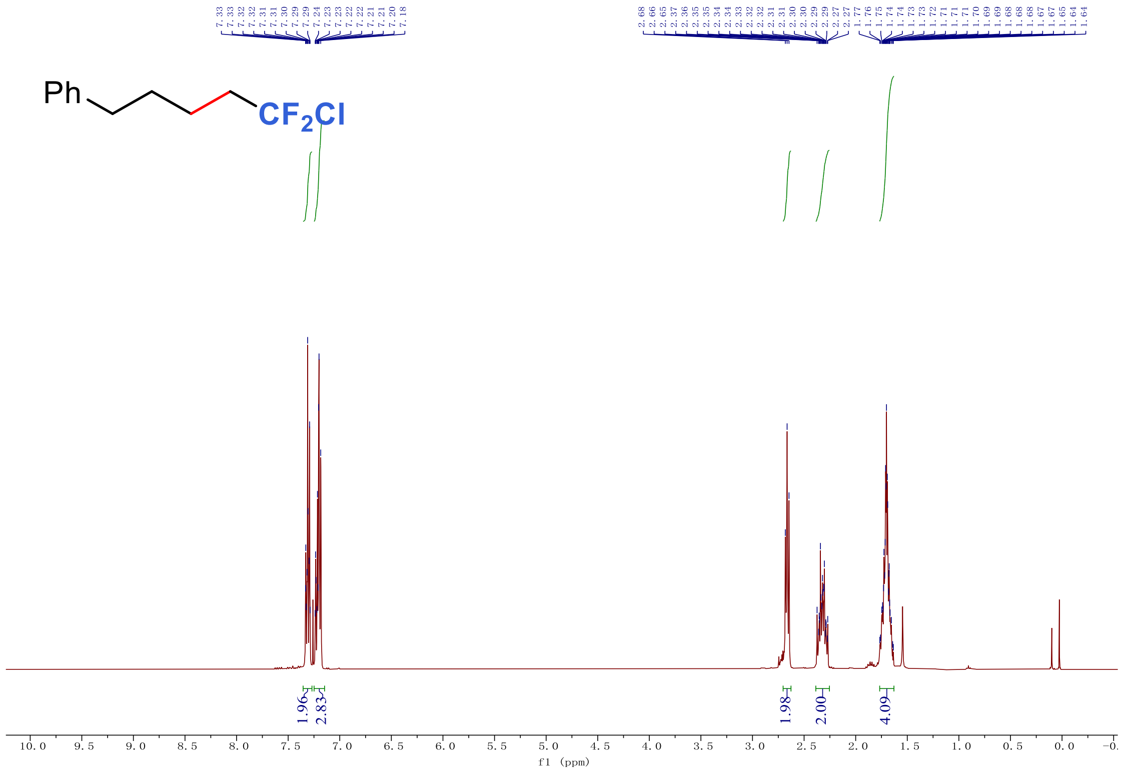

^{19}F NMR (377 MHz, $CDCl_3$) spectra for compound **1j**

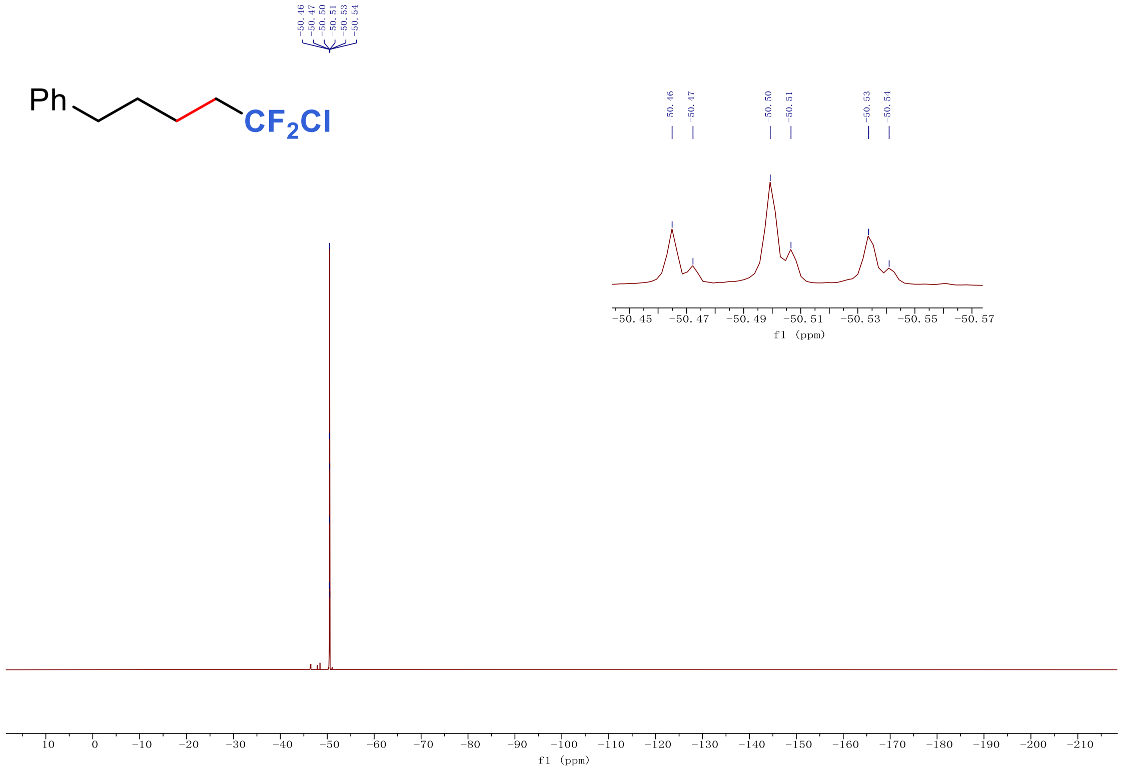

^{13}C NMR (126 MHz, $CDCl_3$) spectra for compound **1j**

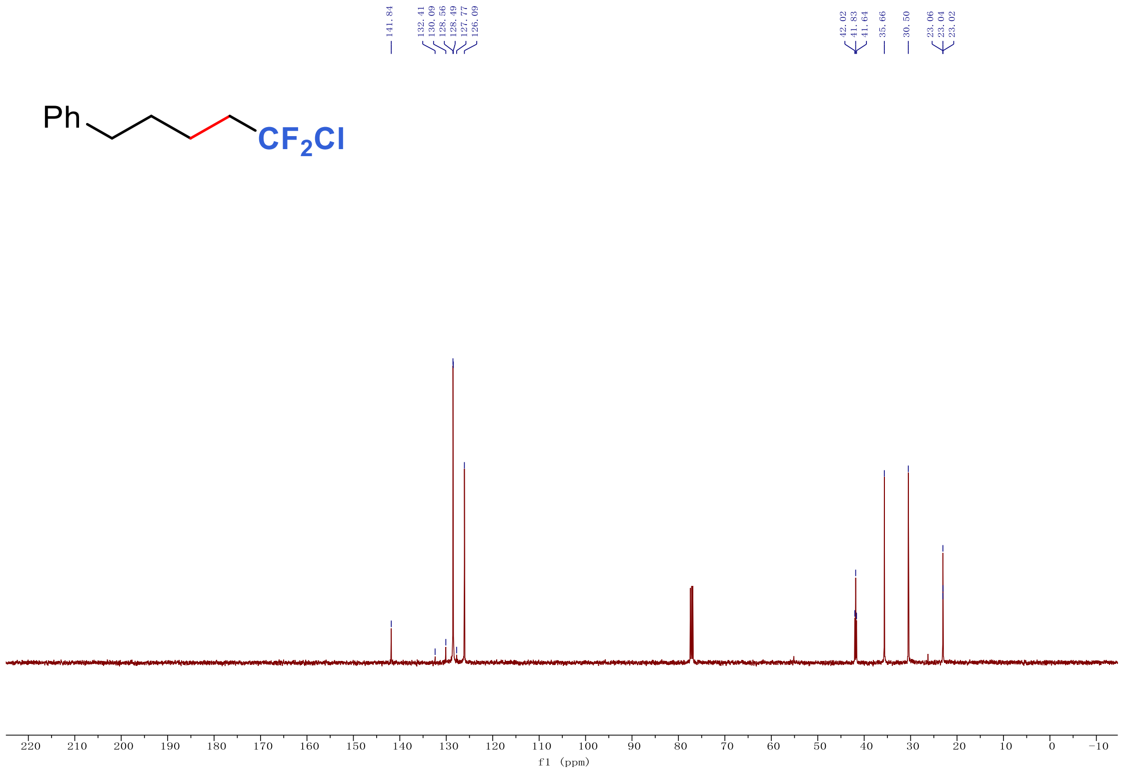

^{1}H NMR (400 MHz, $CDCl_3$) spectra for compound **1k**

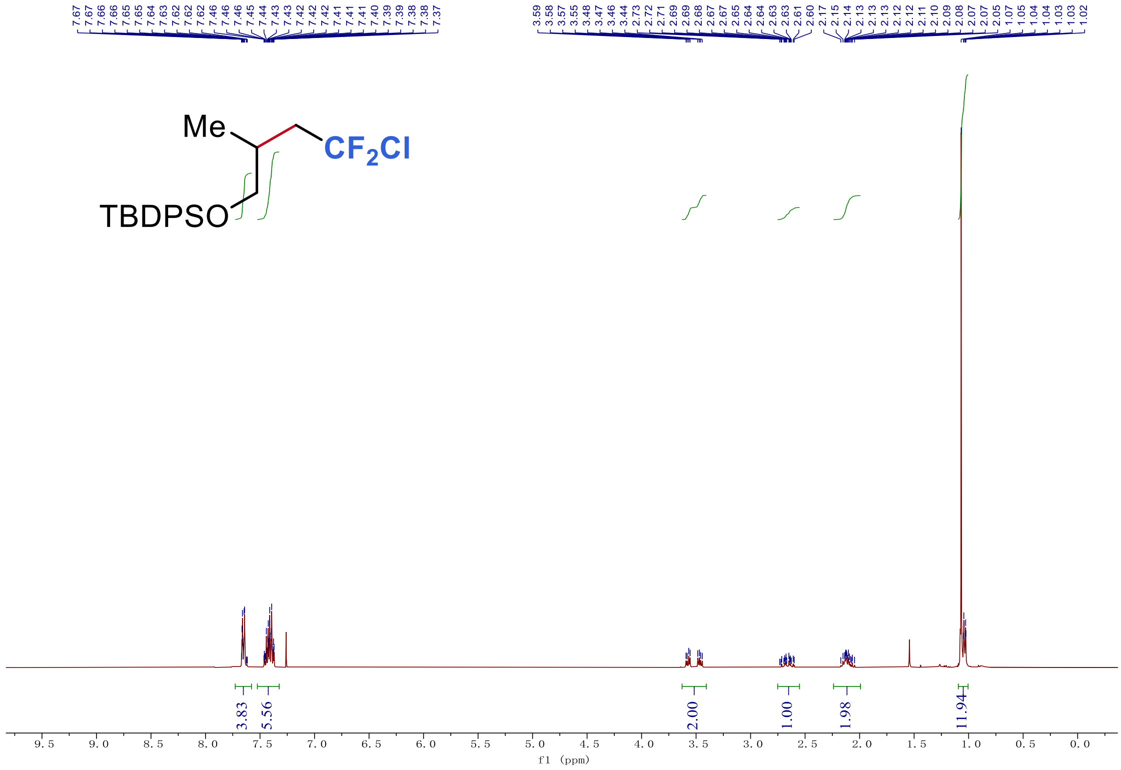

^{19}F NMR (377 MHz, $CDCl_3$) spectra for compound **1k**

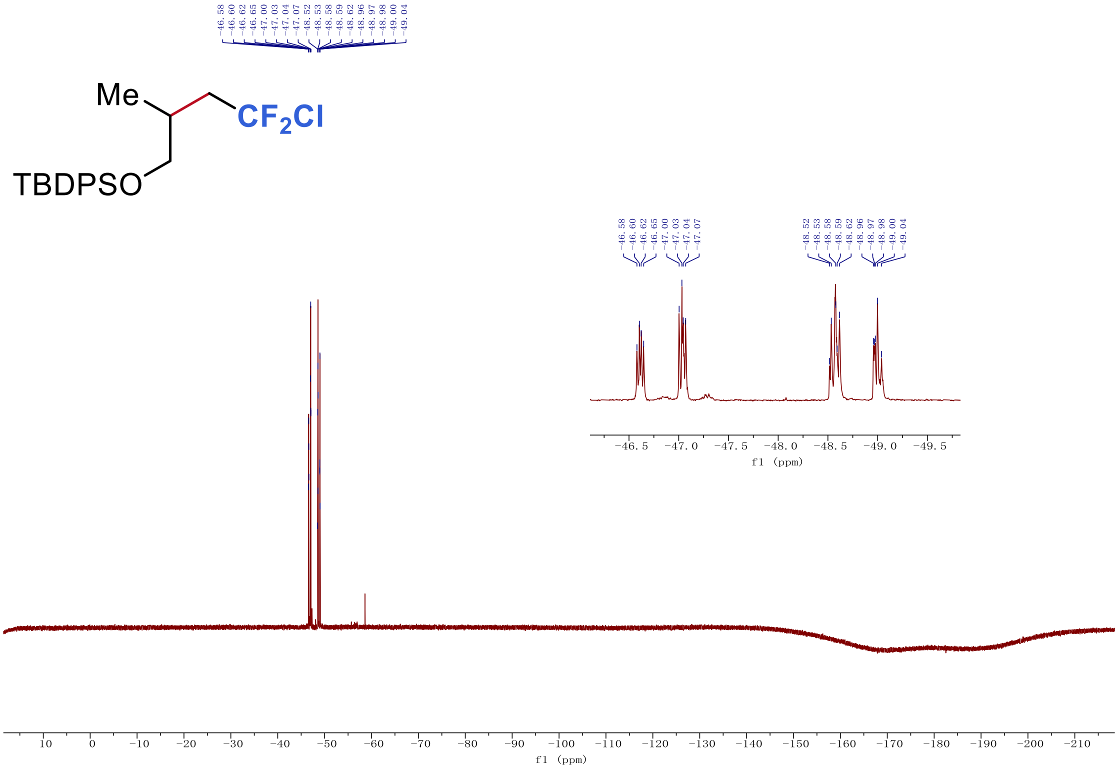

^{13}C NMR (126 MHz, $CDCl_3$) spectra for compound **1k**

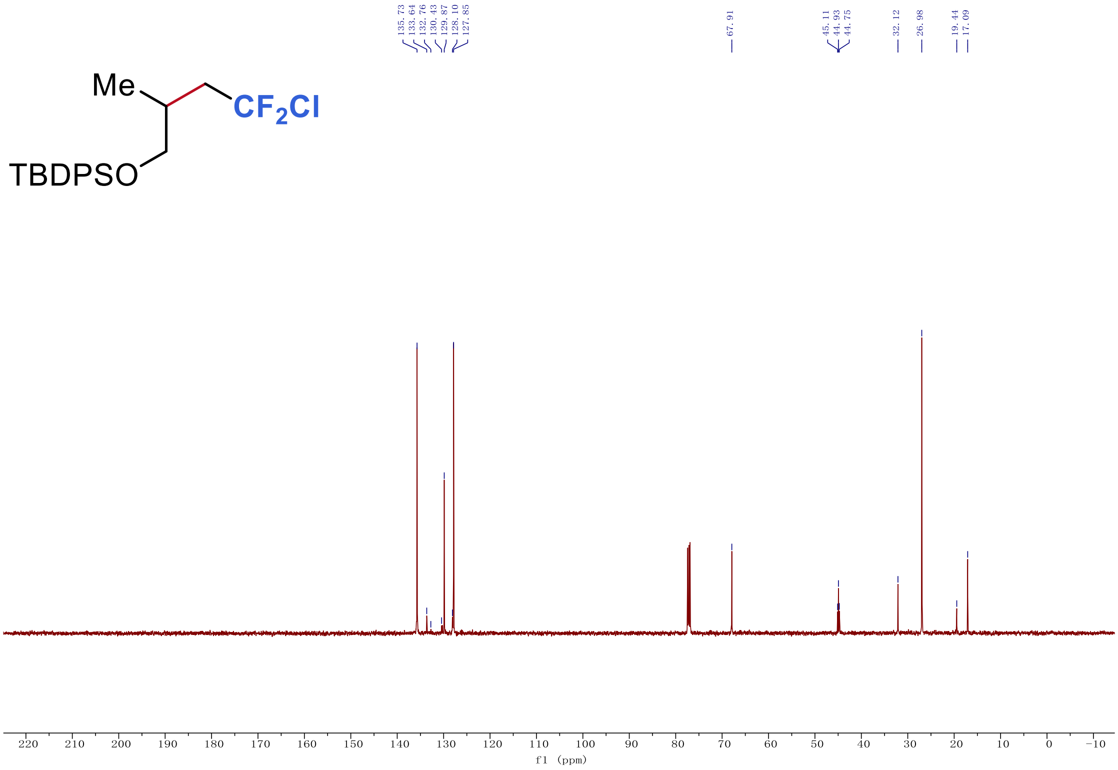

^{1}H NMR (400 MHz, $CDCl_3$) spectra for compound **1l**

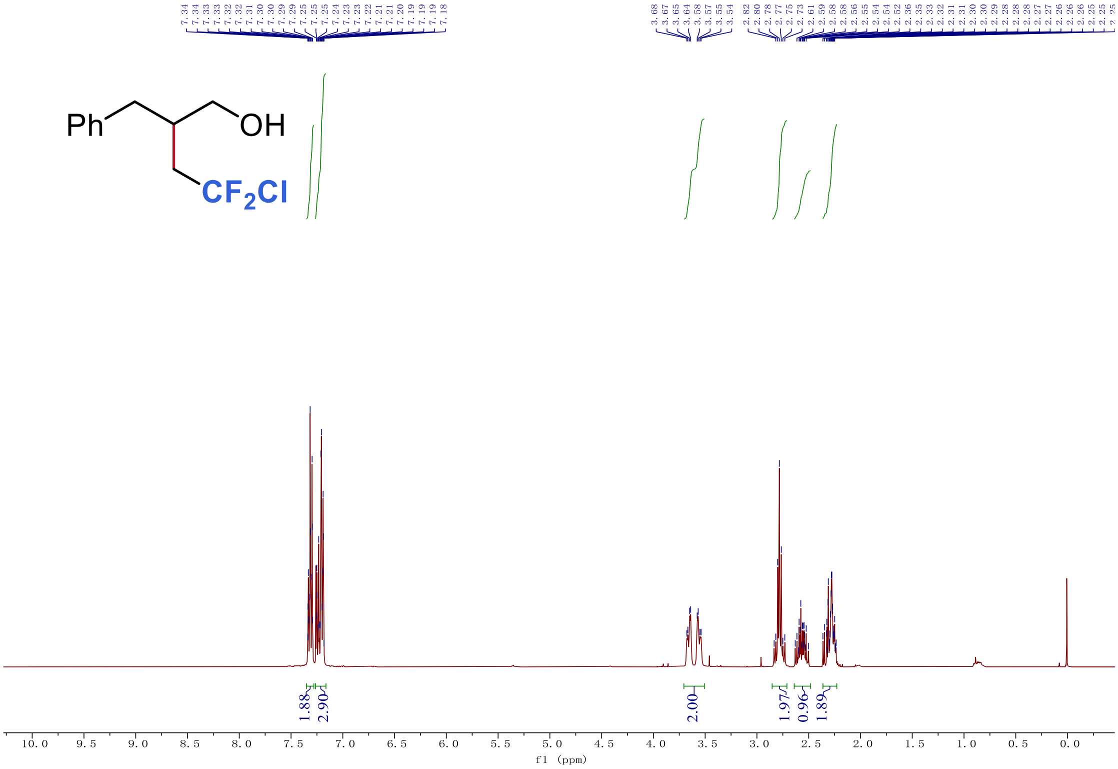

^{19}F NMR (377 MHz, $CDCl_3$) spectra for compound **1l**

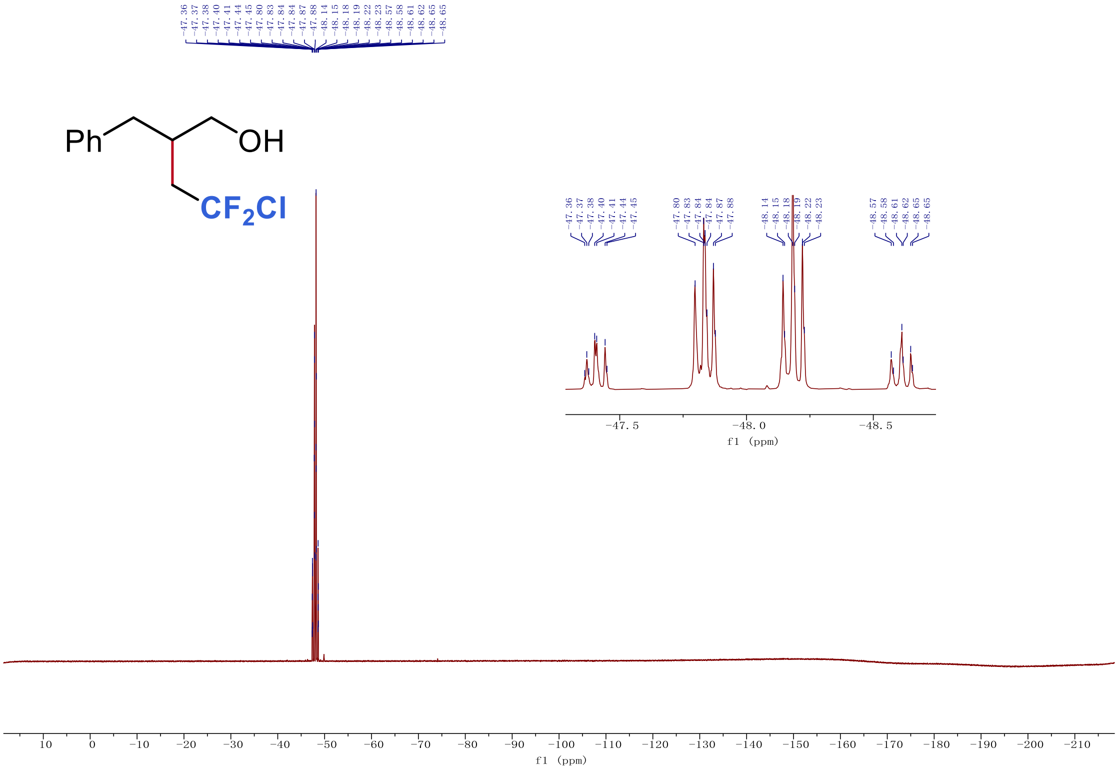

^{13}C NMR (126 MHz, $CDCl_3$) spectra for compound **1l**

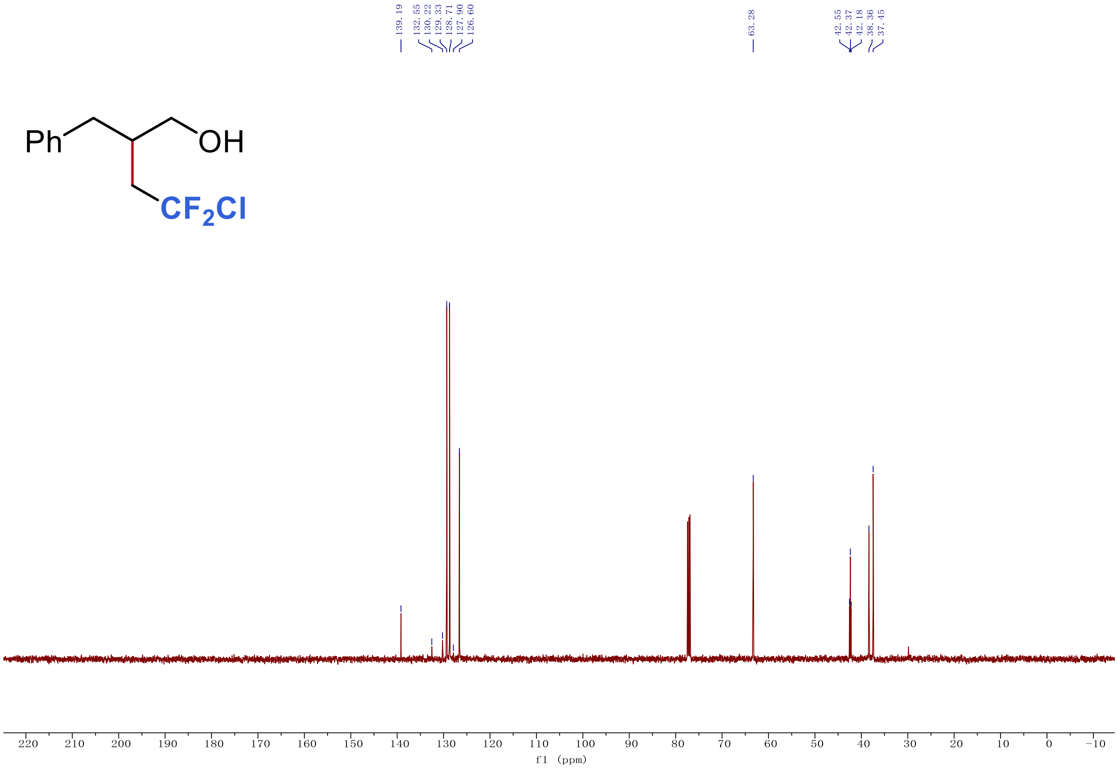

^{1}H NMR (400 MHz, $CDCl_3$) spectra for compound **1m**

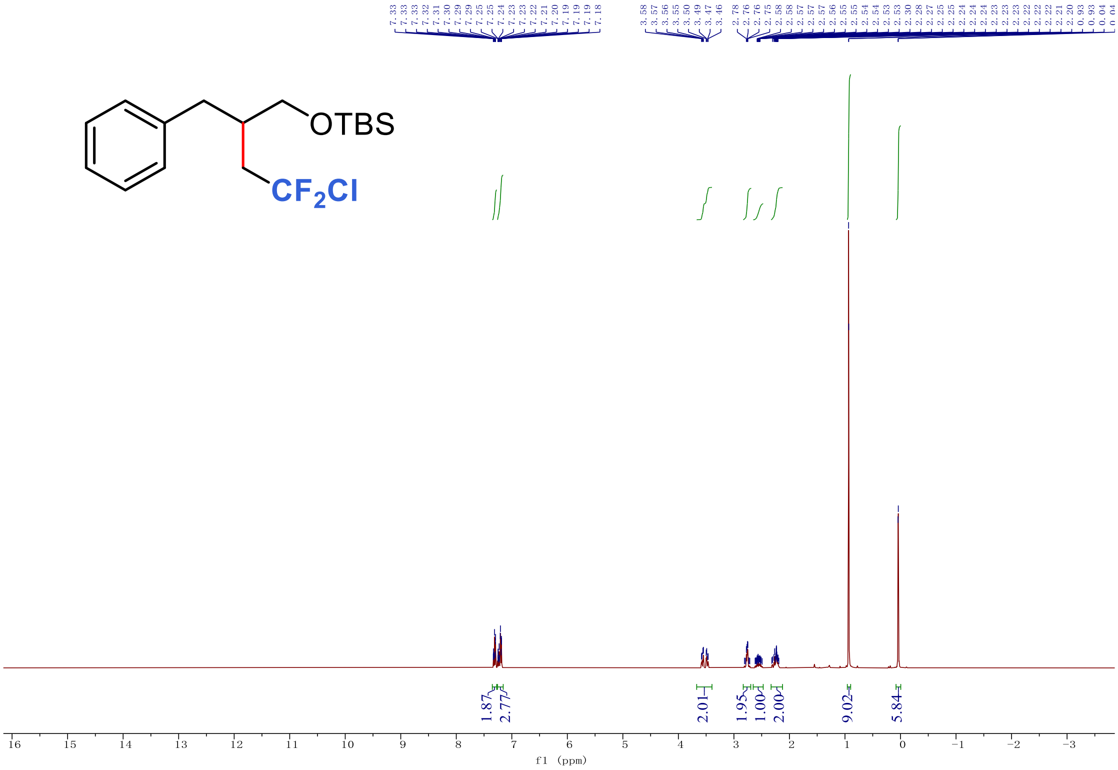

^{19}F NMR (377 MHz, $CDCl_3$) spectra for compound **1m**

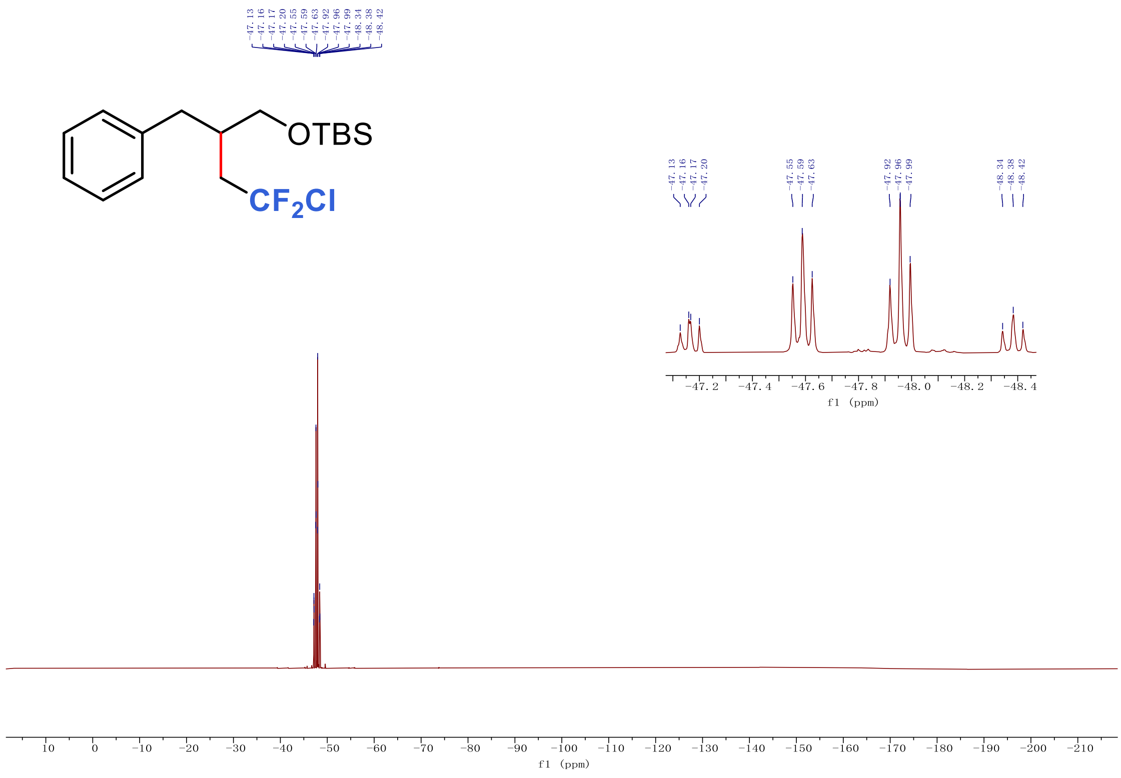

^{13}C NMR (126 MHz, $CDCl_3$) spectra for compound **1m**

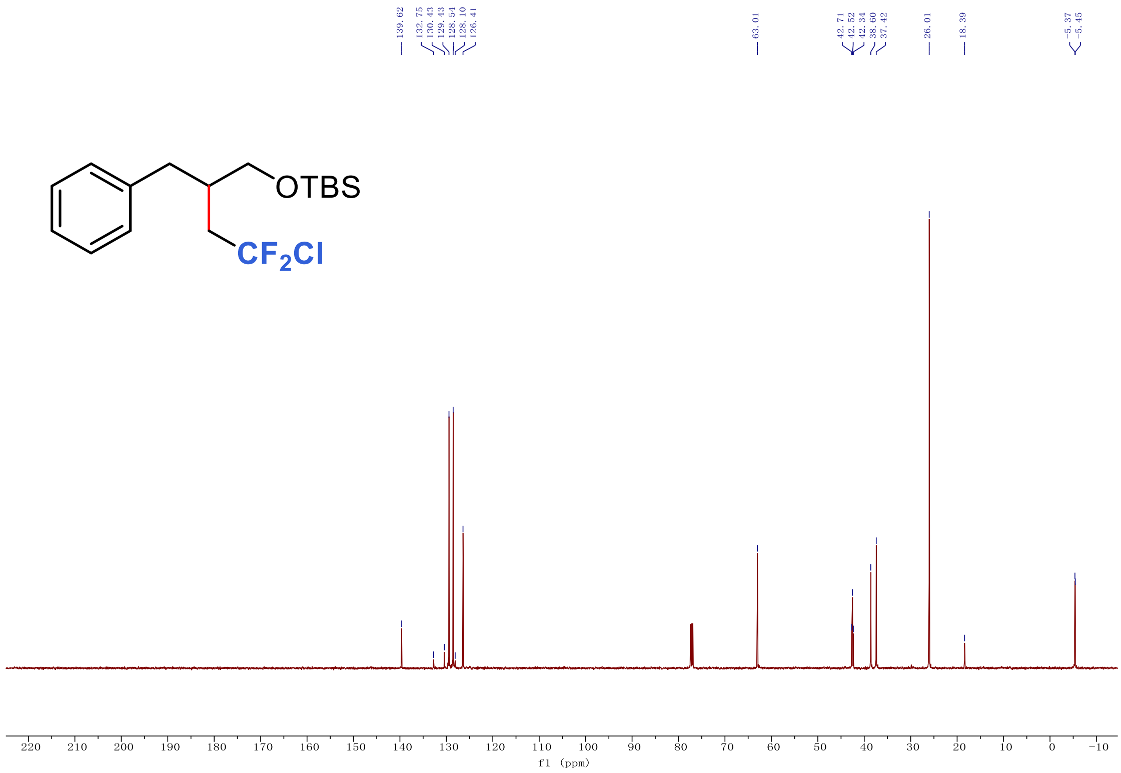

^{1}H NMR (400 MHz, $CDCl_3$) spectra for compound **1n**

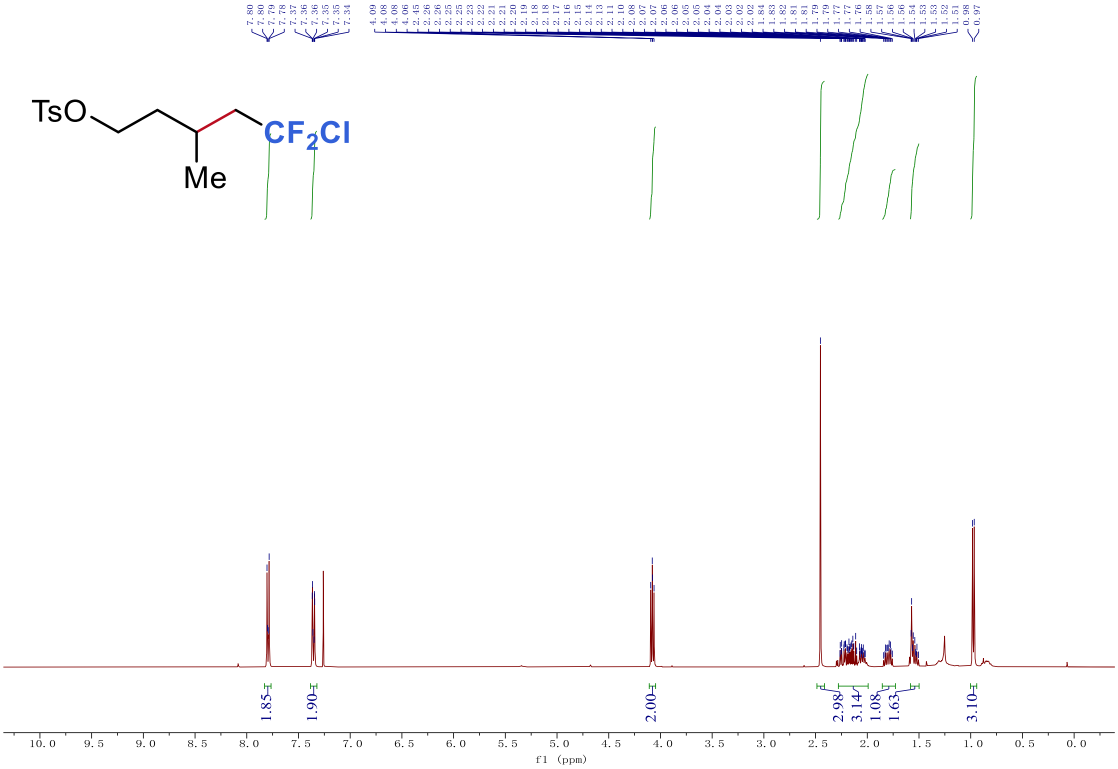

^{19}F NMR (377 MHz, $CDCl_3$) spectra for compound **1n**

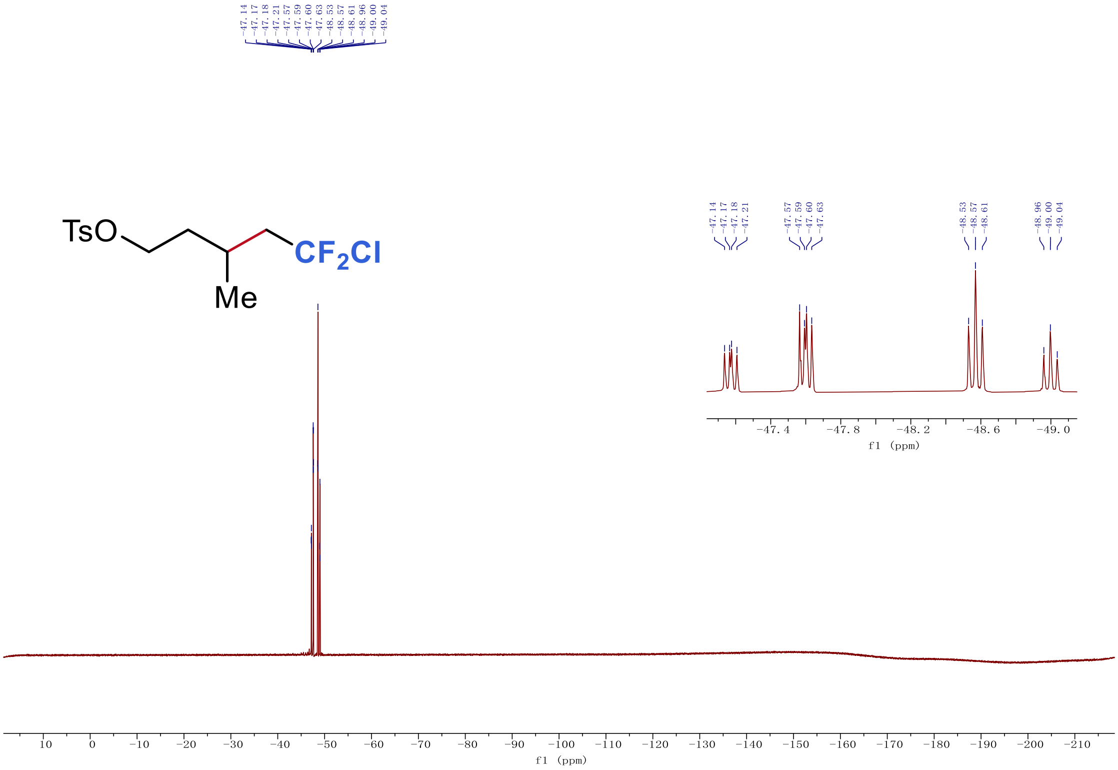

^{13}C NMR (126 MHz, $CDCl_3$) spectra for compound **1n**

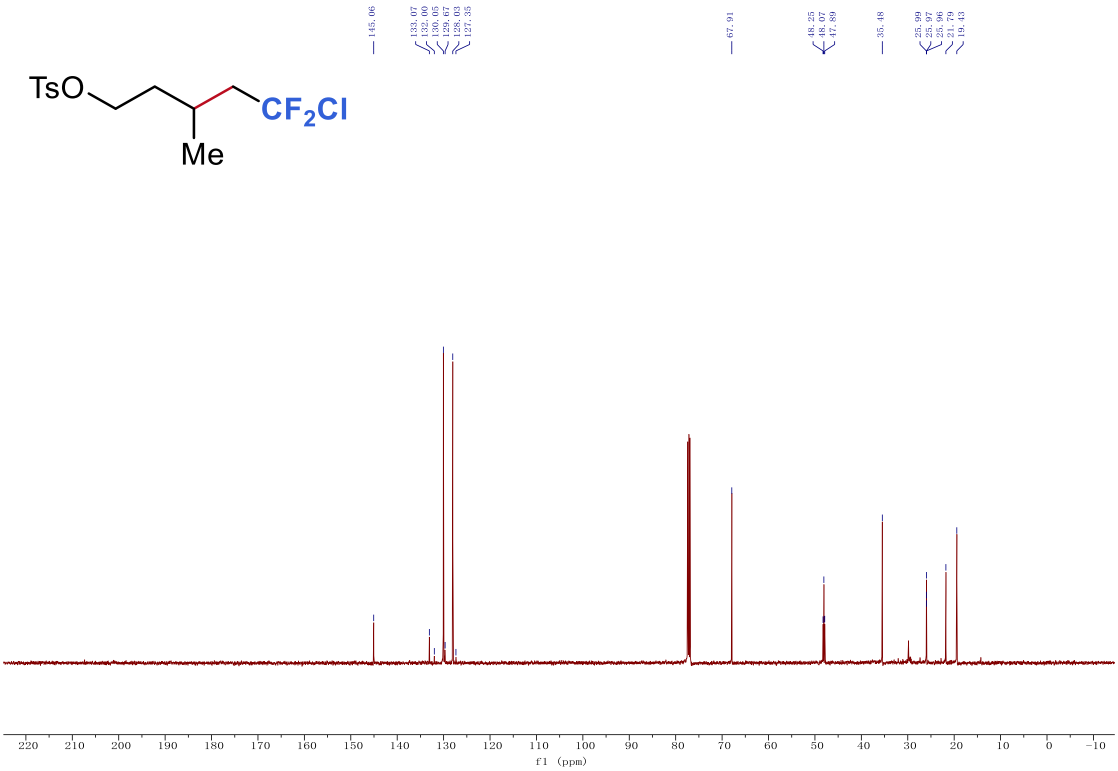

^{1}H NMR (400 MHz, $CDCl_3$) spectra for compound **1o**

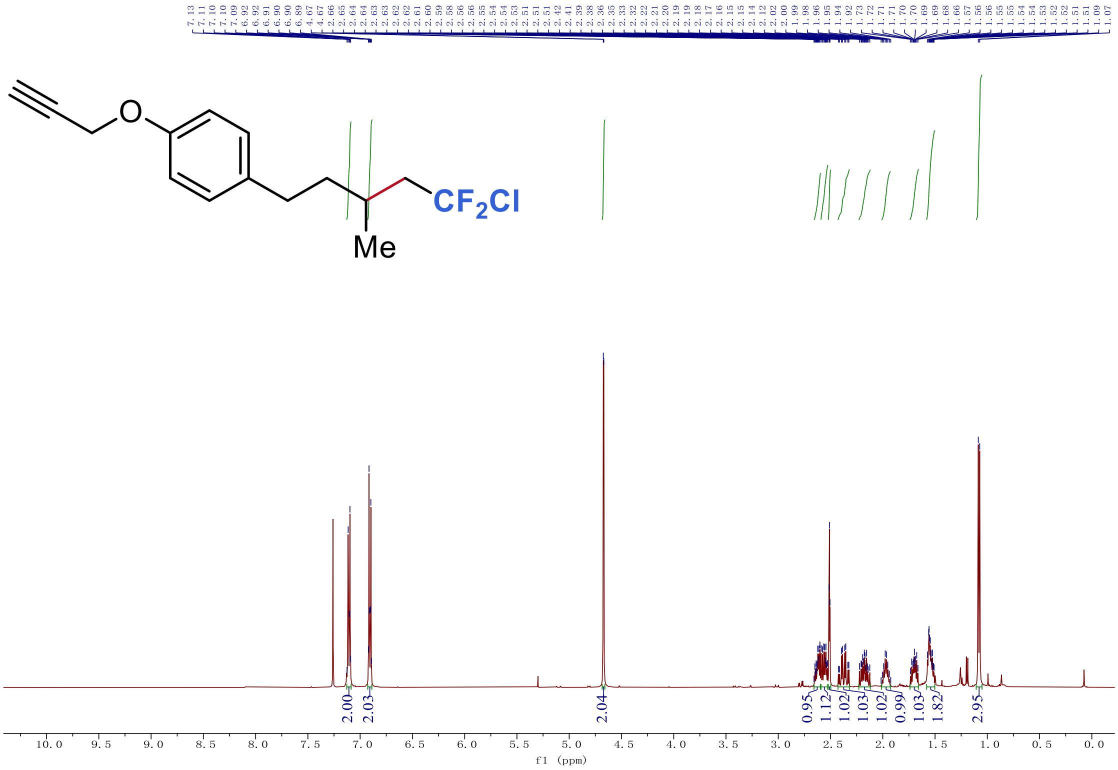

^{19}F NMR (377 MHz, $CDCl_3$) spectra for compound **1o**

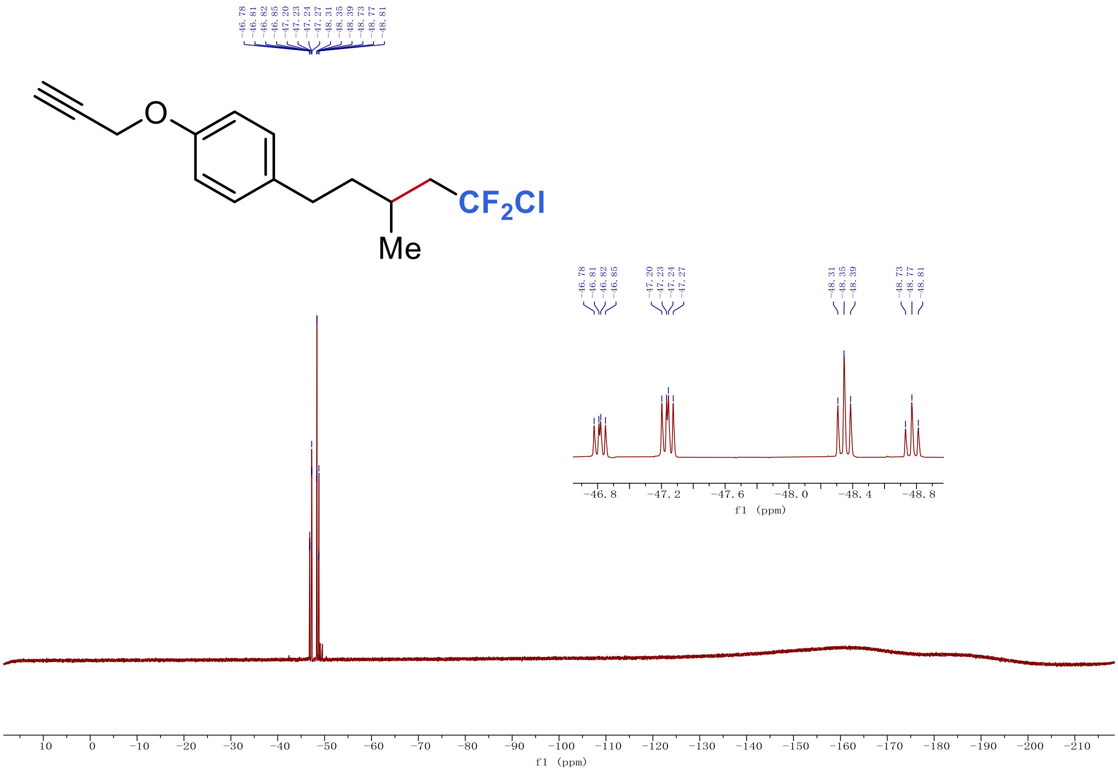

^{13}C NMR (126 MHz, $CDCl_3$) spectra for compound **1o**

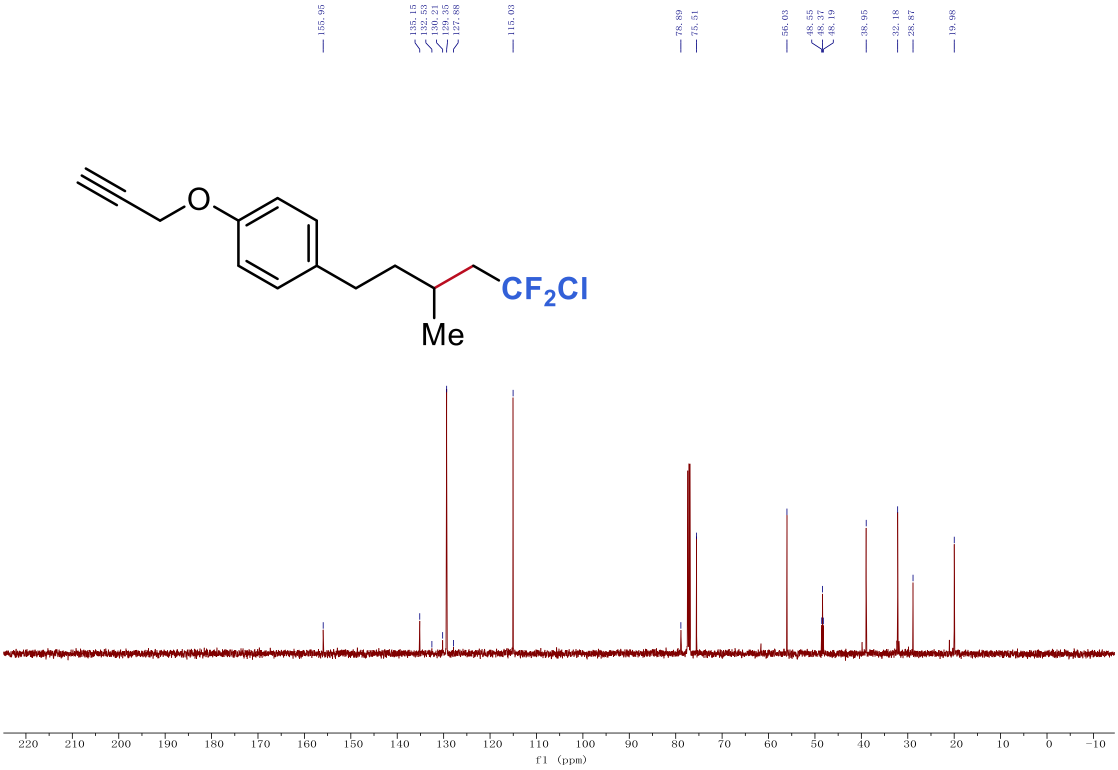

^{1}H NMR (400 MHz, $CDCl_3$) spectra for compound **1p**

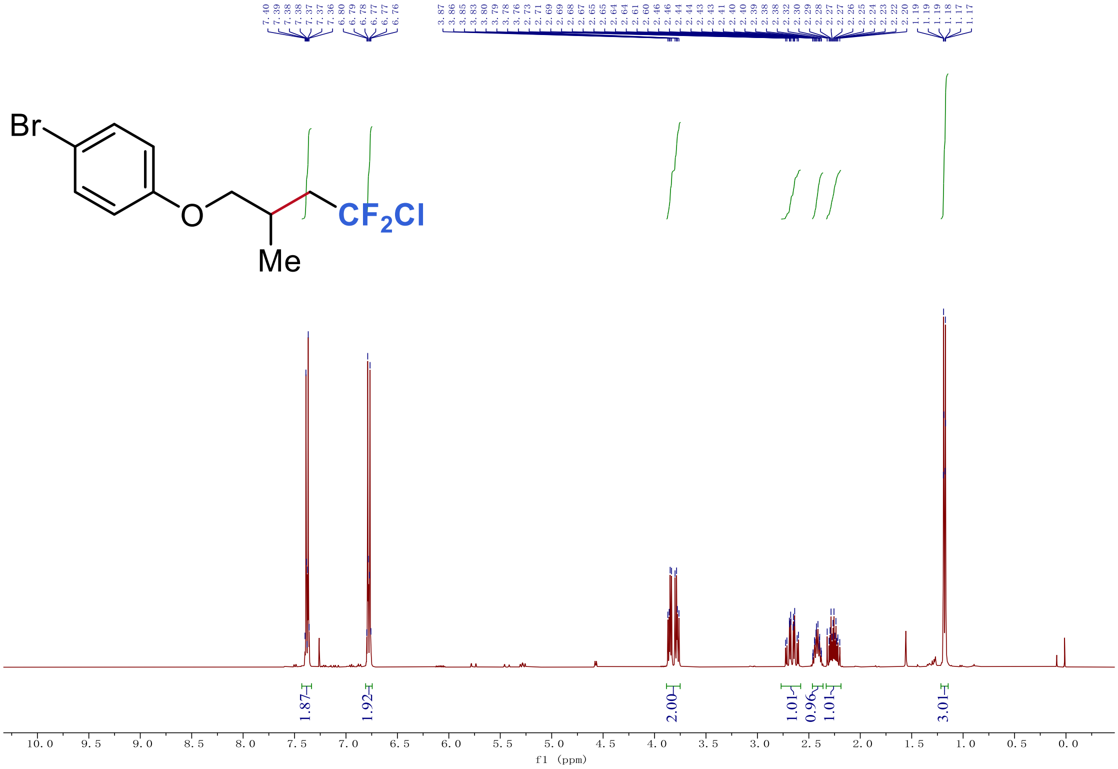

^{19}F NMR (377 MHz, $CDCl_3$) spectra for compound **1p**

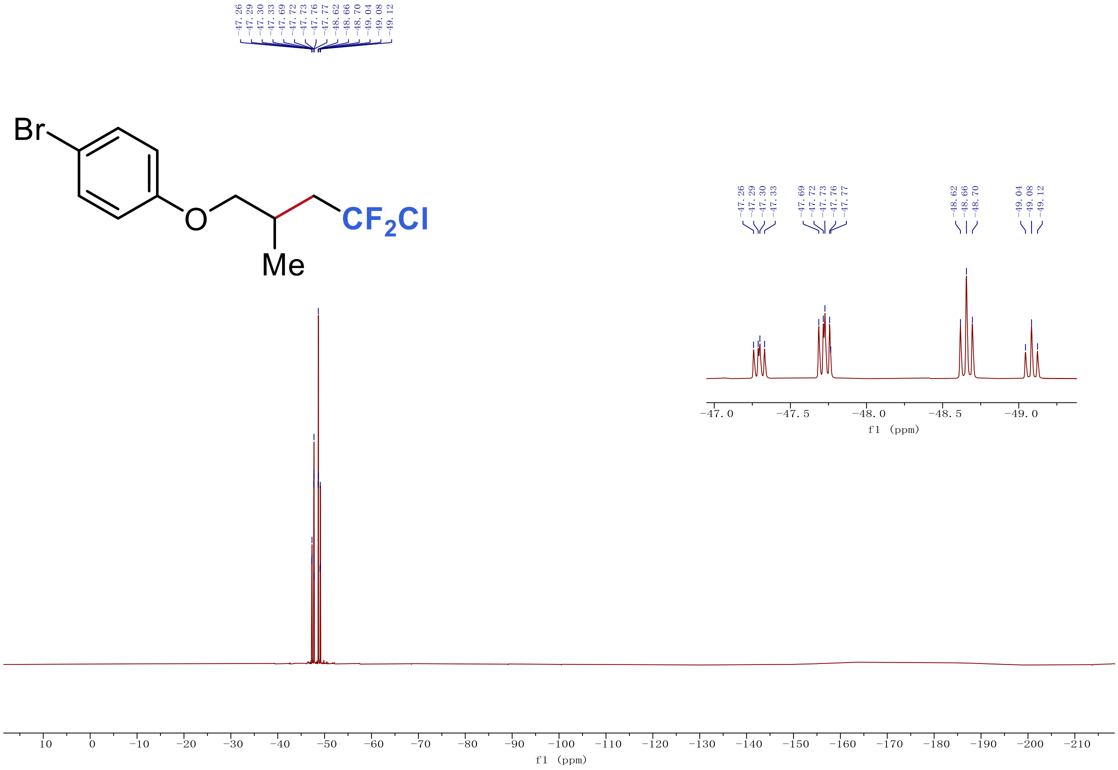

^{13}C NMR (126 MHz, $CDCl_3$) spectra for compound **1p**

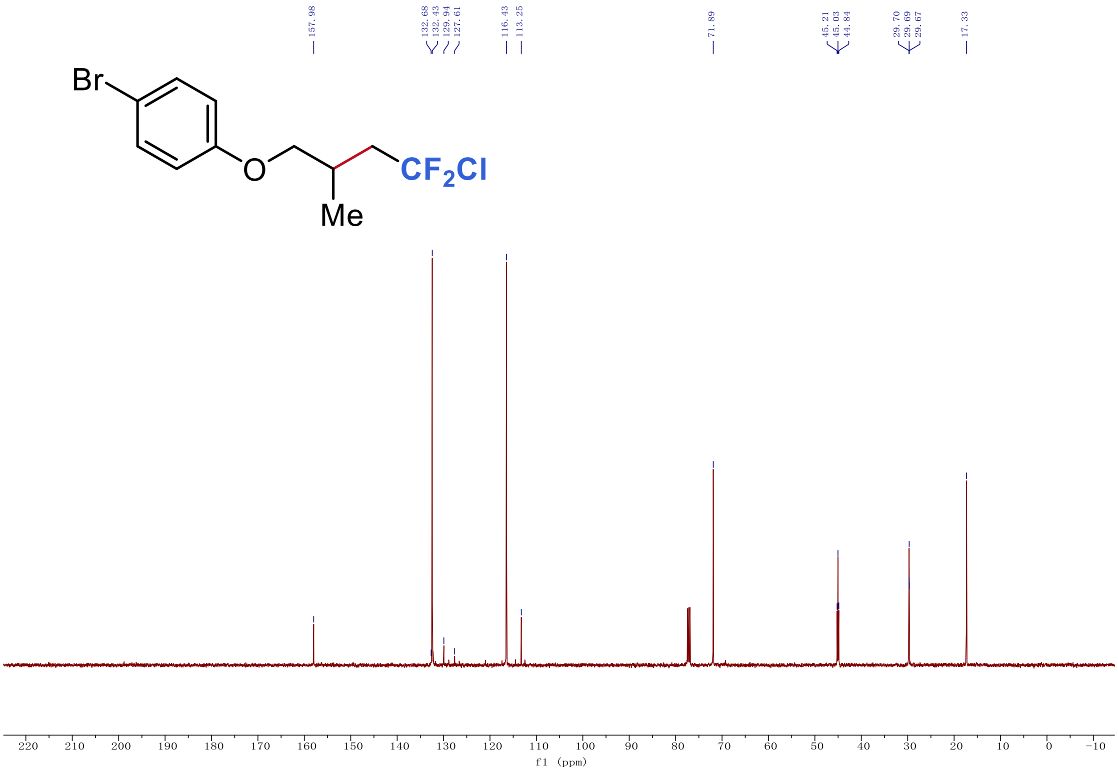

^{1}H NMR (400 MHz, $CDCl_3$) spectra for compound **1q**

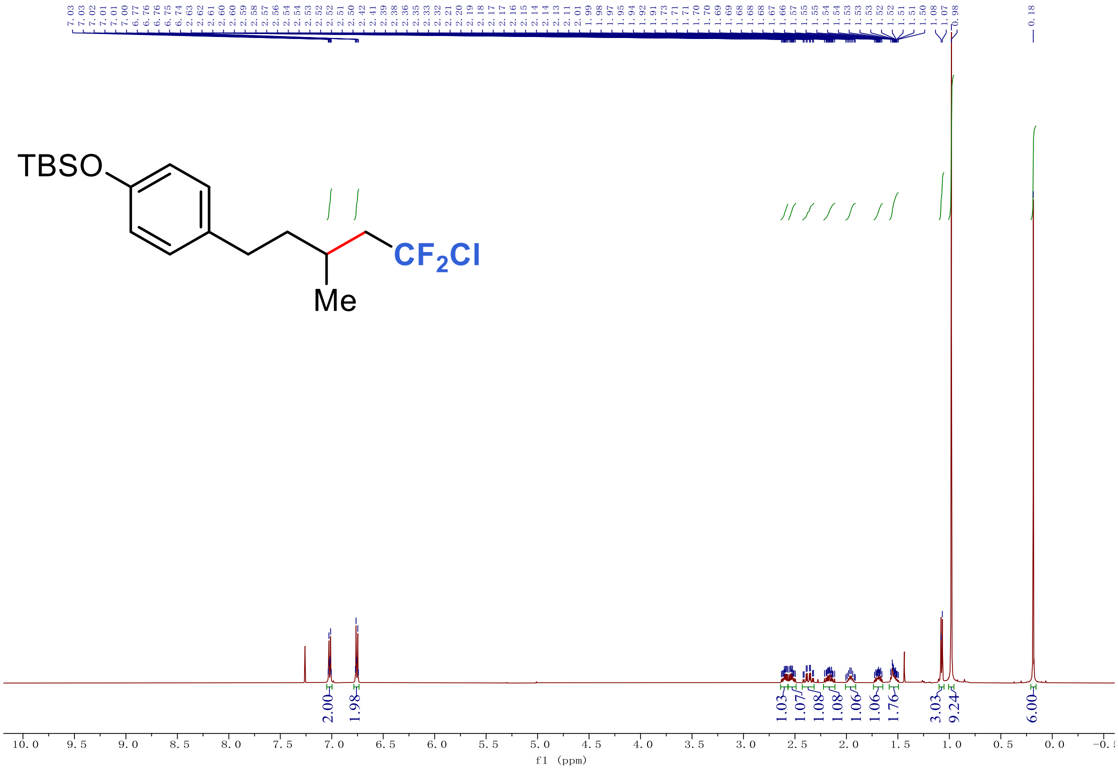

^{19}F NMR (377 MHz, $CDCl_3$) spectra for compound **1q**

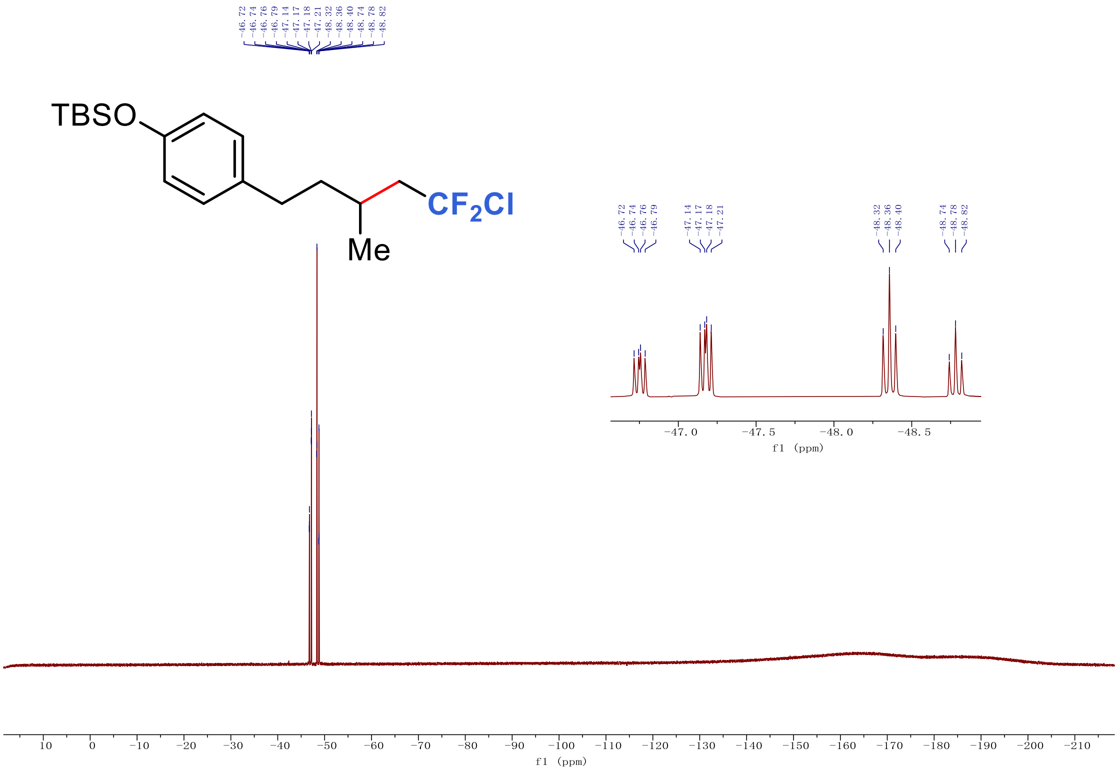

^{13}C NMR (126 MHz, $CDCl_3$) spectra for compound **1q**

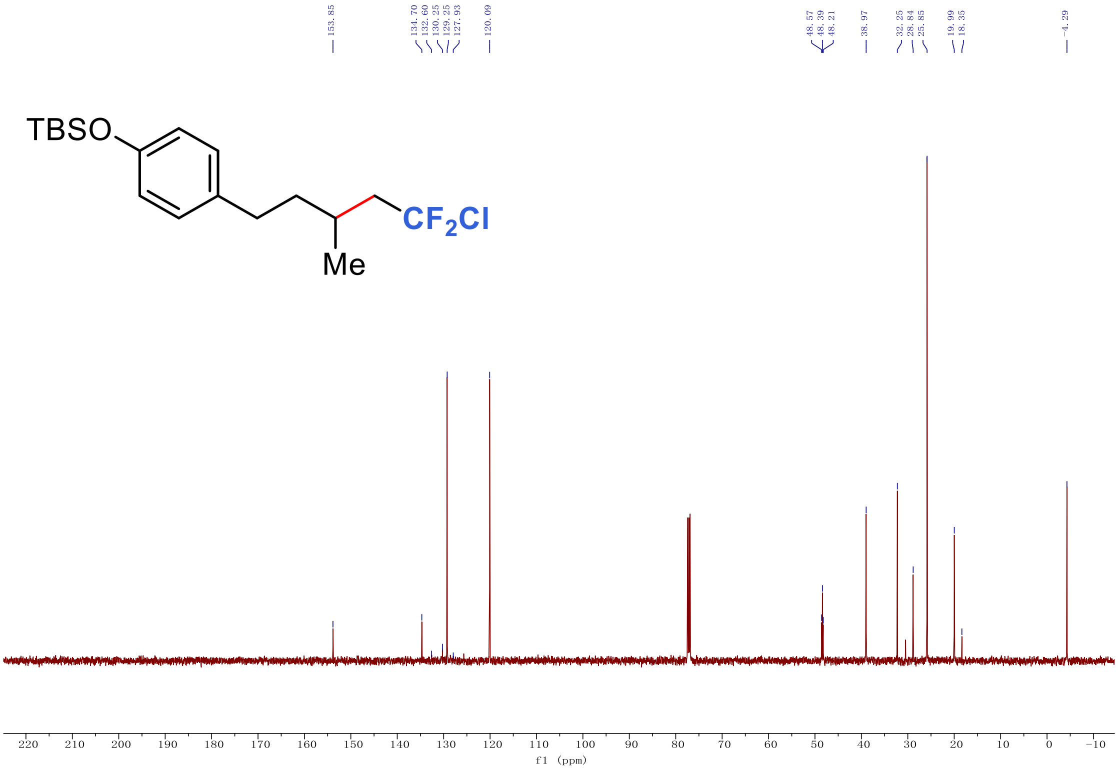

¹H NMR (400 MHz, $CDCl_3$) spectra for compound **1r**

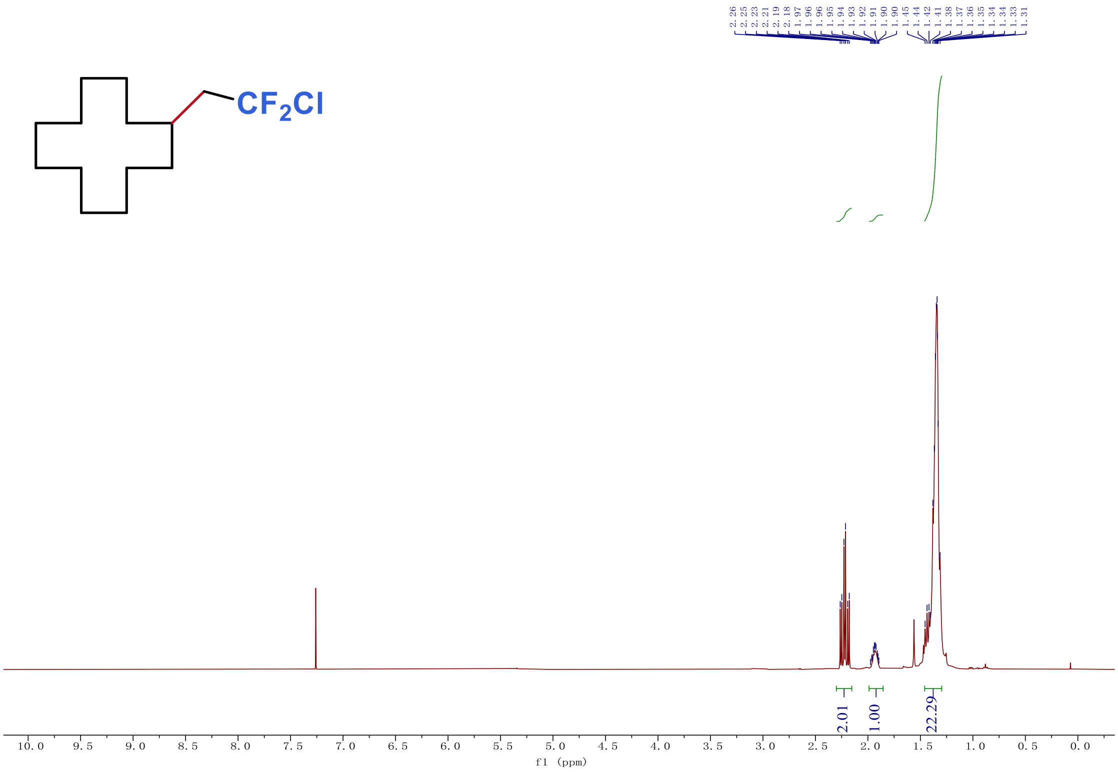

¹⁹F NMR (377 MHz, $CDCl_3$) spectra for compound **1r**

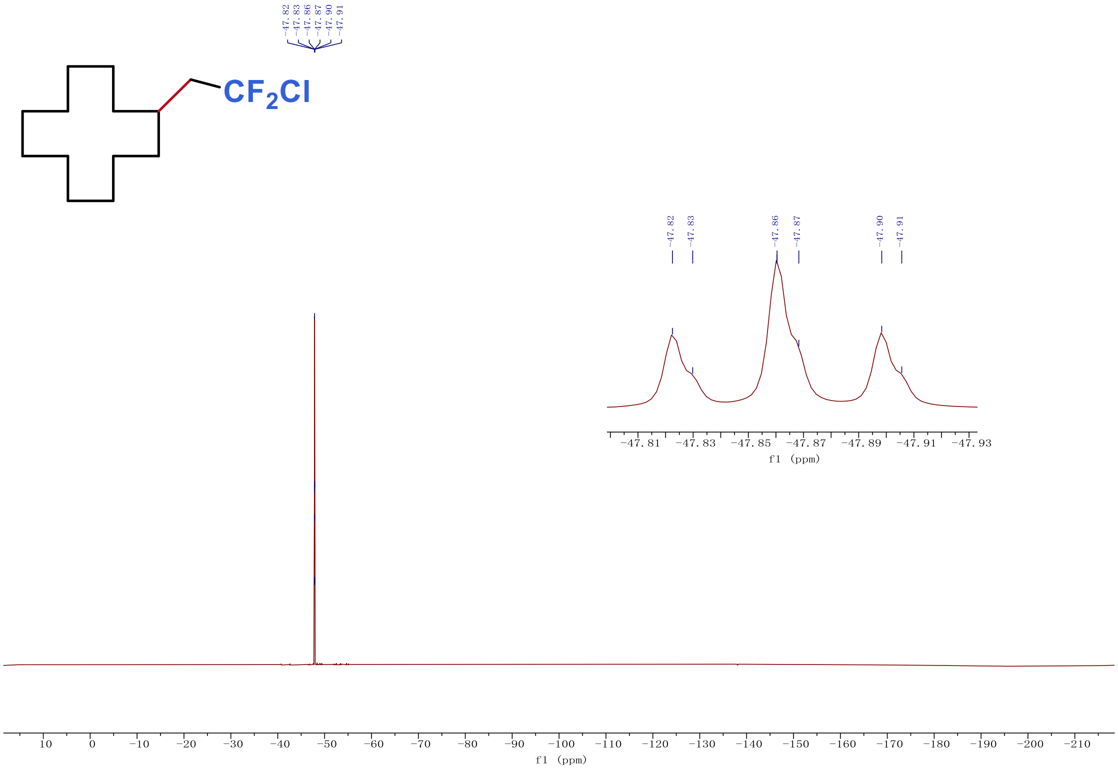

^{13}**C NMR** (126 MHz, $CDCl_3$) spectra for compound **1r**

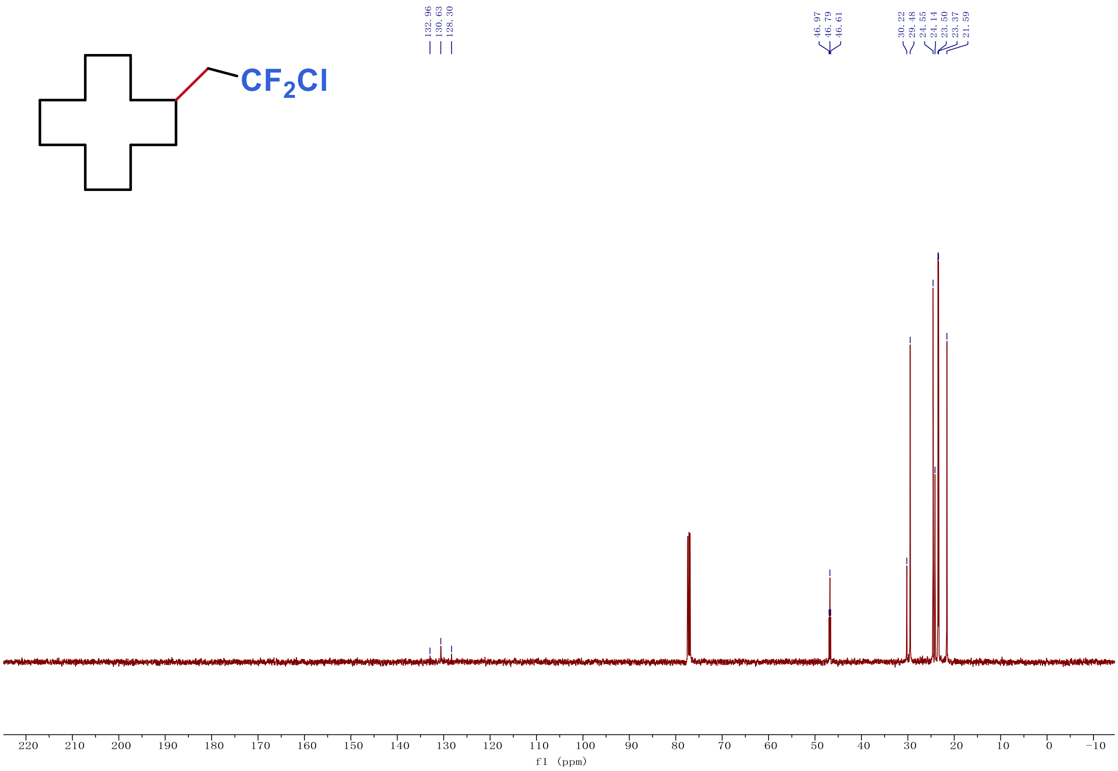

^{1}**H NMR** (400 MHz, $CDCl_3$) spectra for compound **1s**

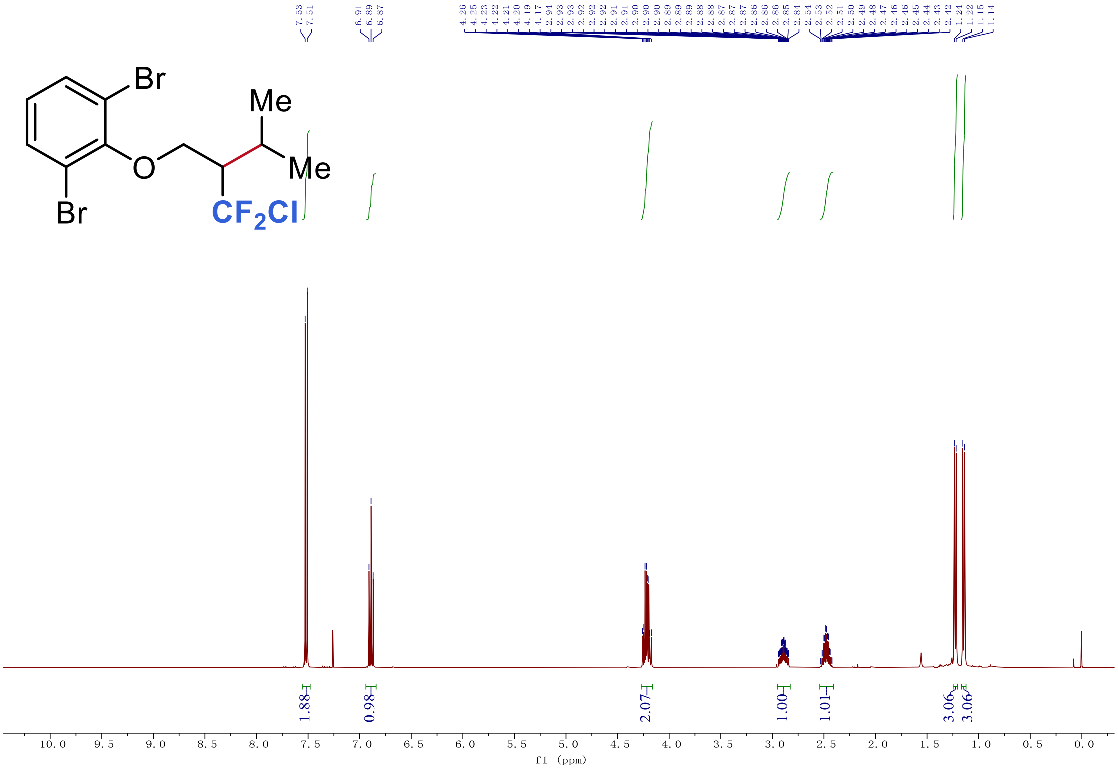

^{19}F NMR (377 MHz, $CDCl_3$) spectra for compound **1s**

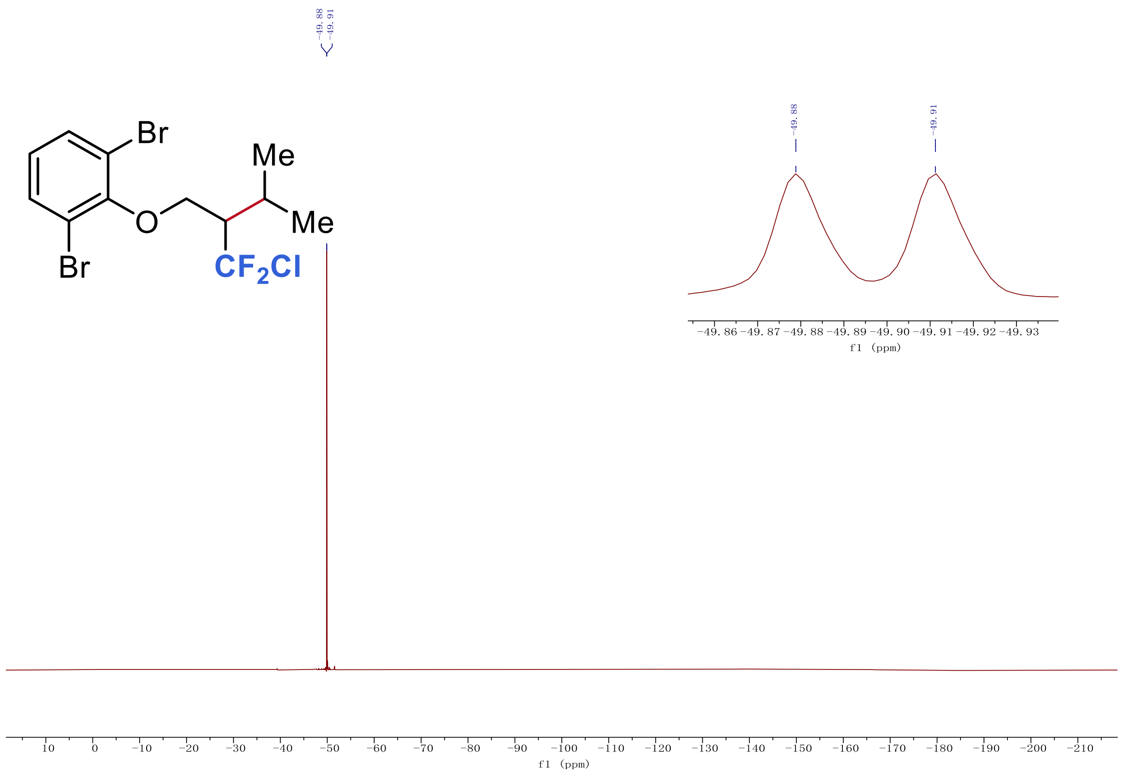

^{13}C NMR (126 MHz, $CDCl_3$) spectra for compound **1s**

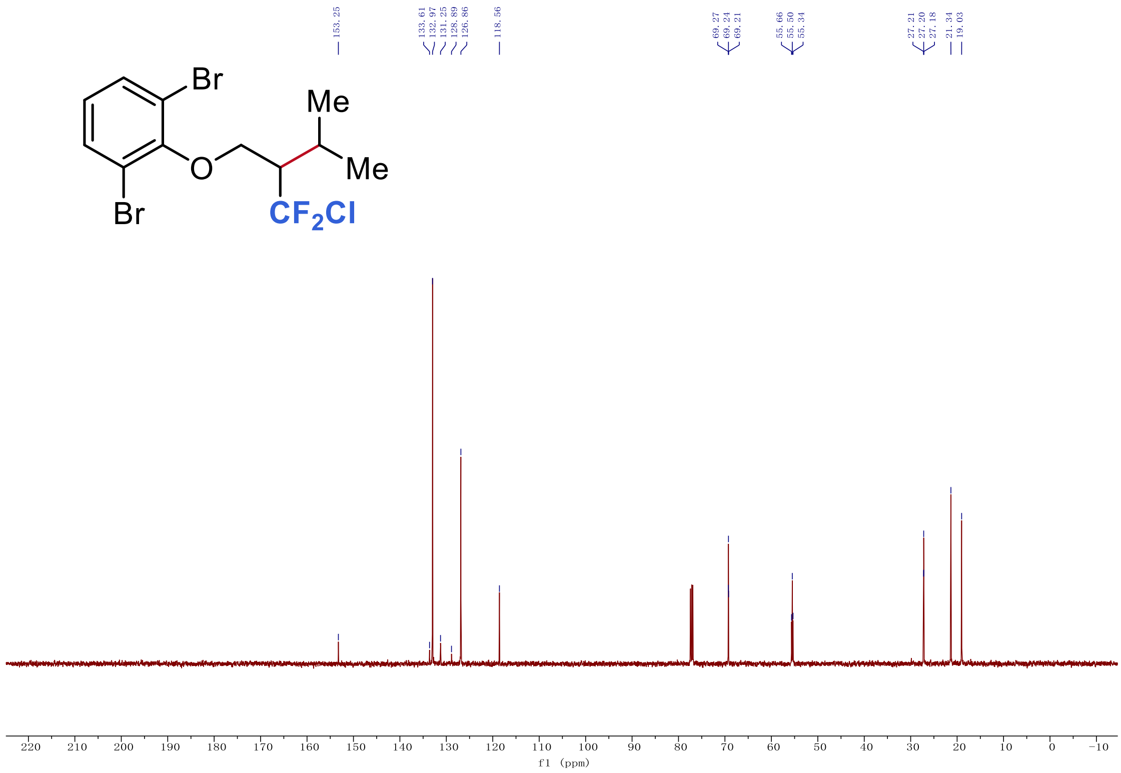

^{1}H NMR (400 MHz, $CDCl_3$) spectra for compound **1t**

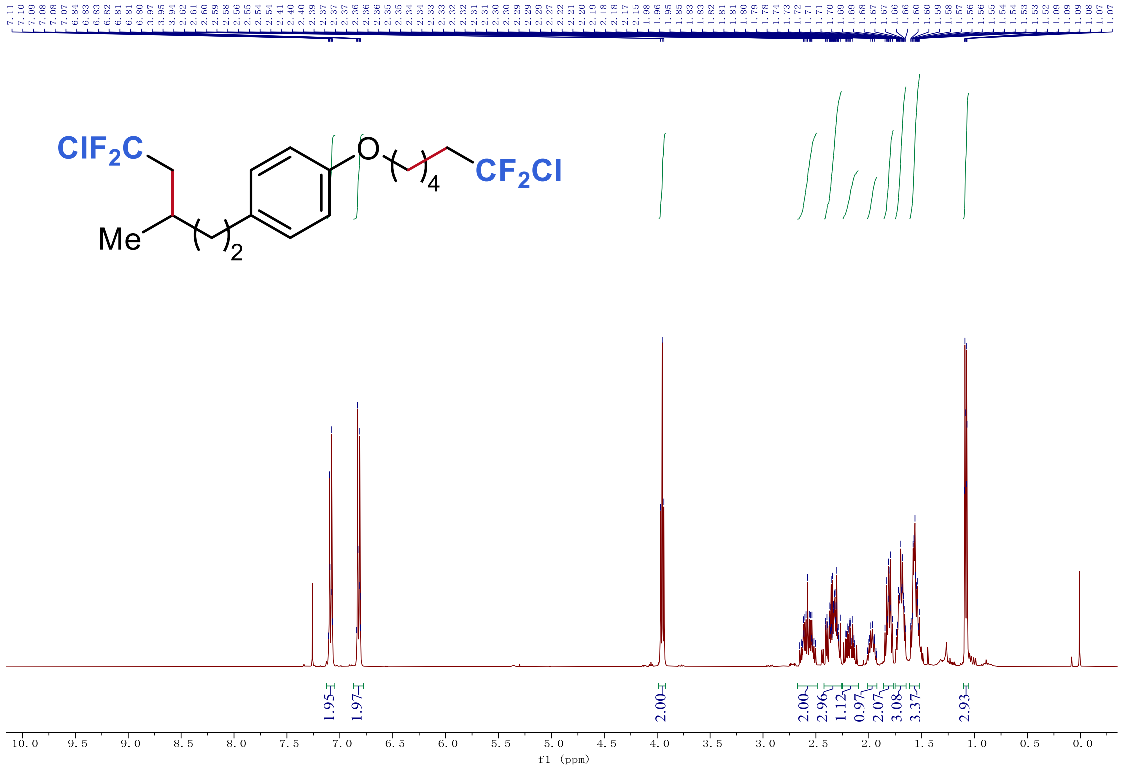

^{19}F NMR (377 MHz, $CDCl_3$) spectra for compound **1t**

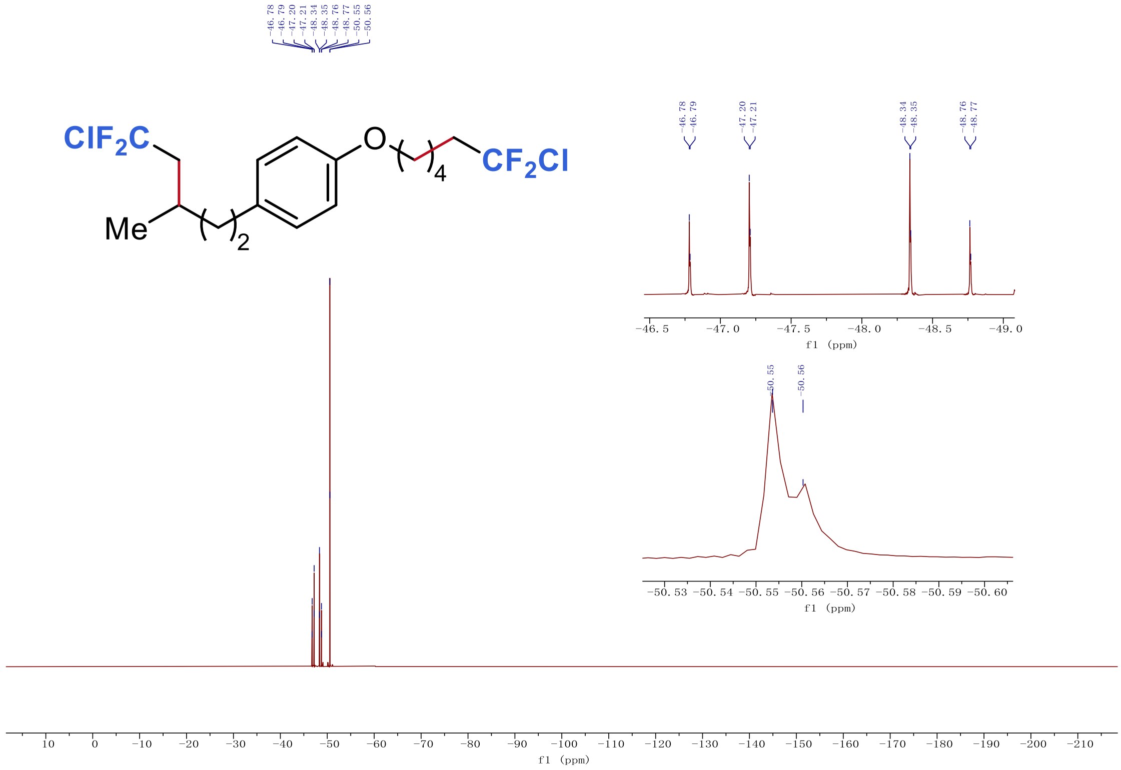

[13]**C NMR** (126 MHz, $CDCl_3$) spectra for compound **1t**

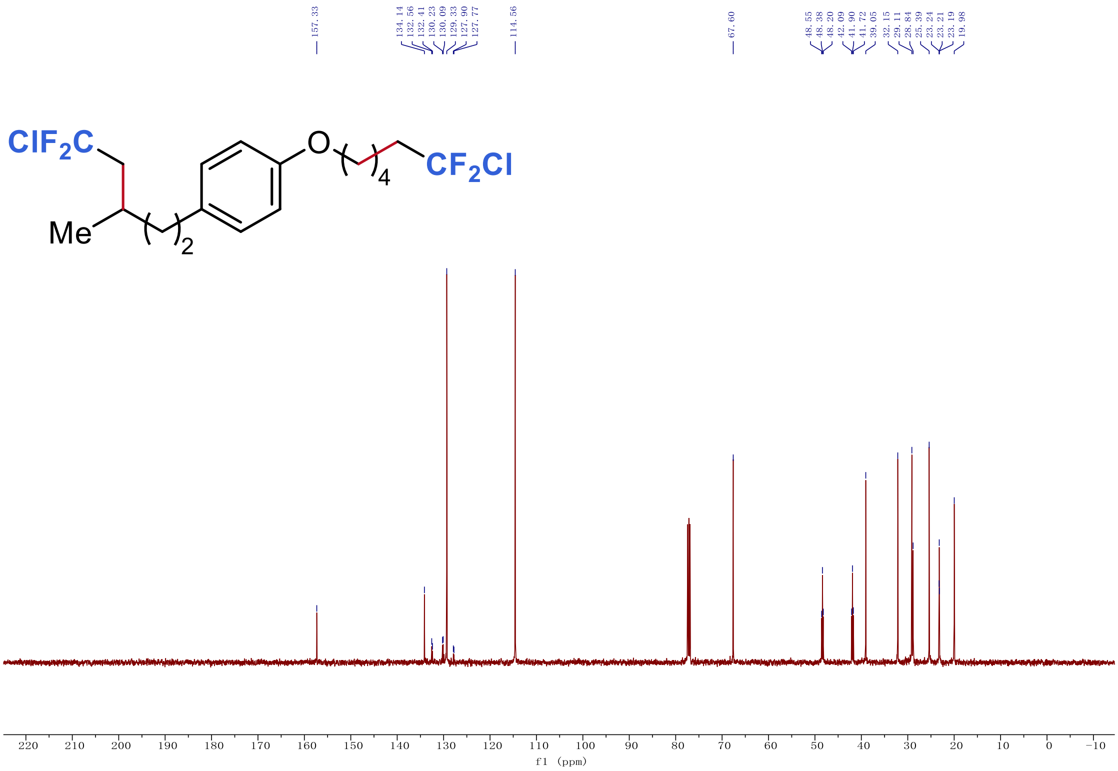

[1]**H NMR** (400 MHz, $CDCl_3$) spectra for compound **1u**

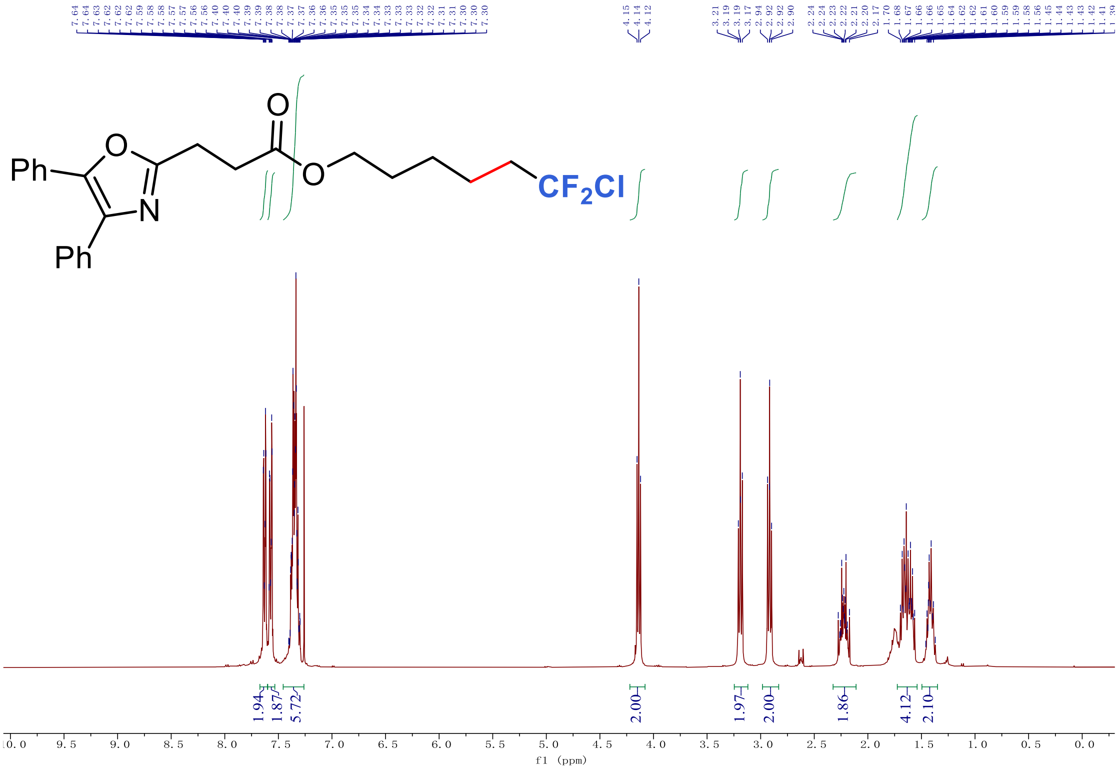

^{19}F NMR (377 MHz, $CDCl_3$) spectra for compound **1u**

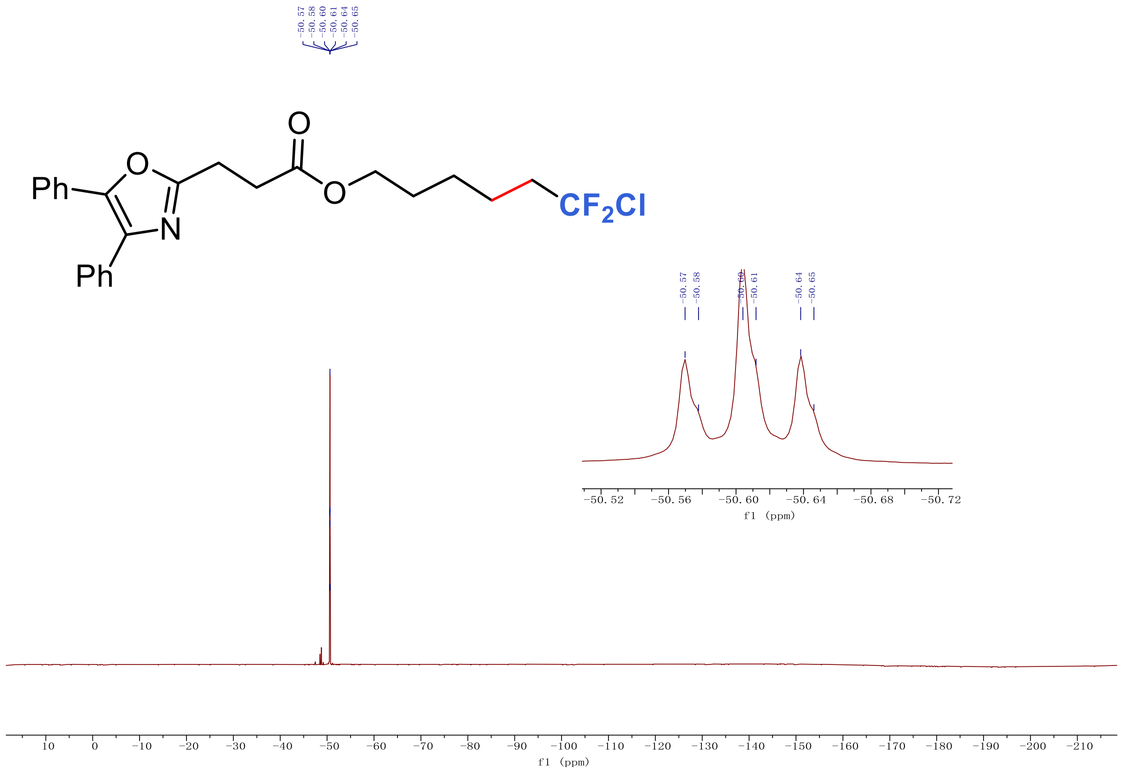

^{13}C NMR (126 MHz, $CDCl_3$) spectra for compound **1u**

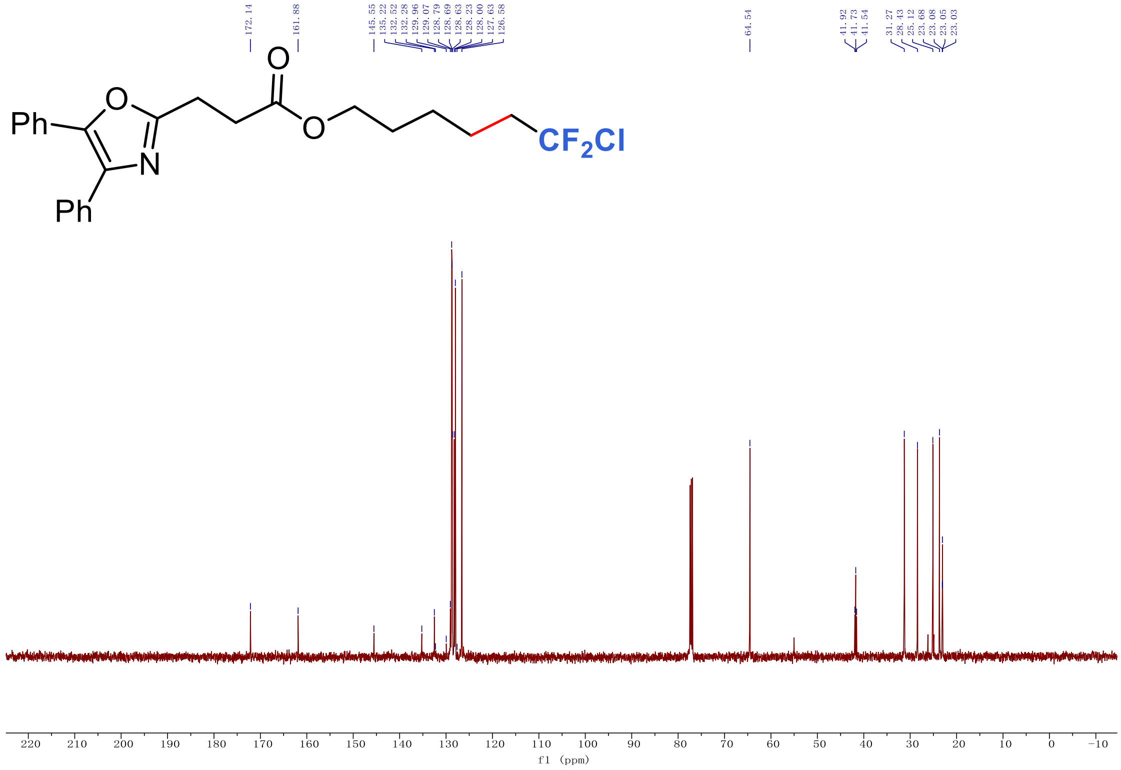

^{1}H NMR (400 MHz, $CDCl_3$) spectra for compound **1v**

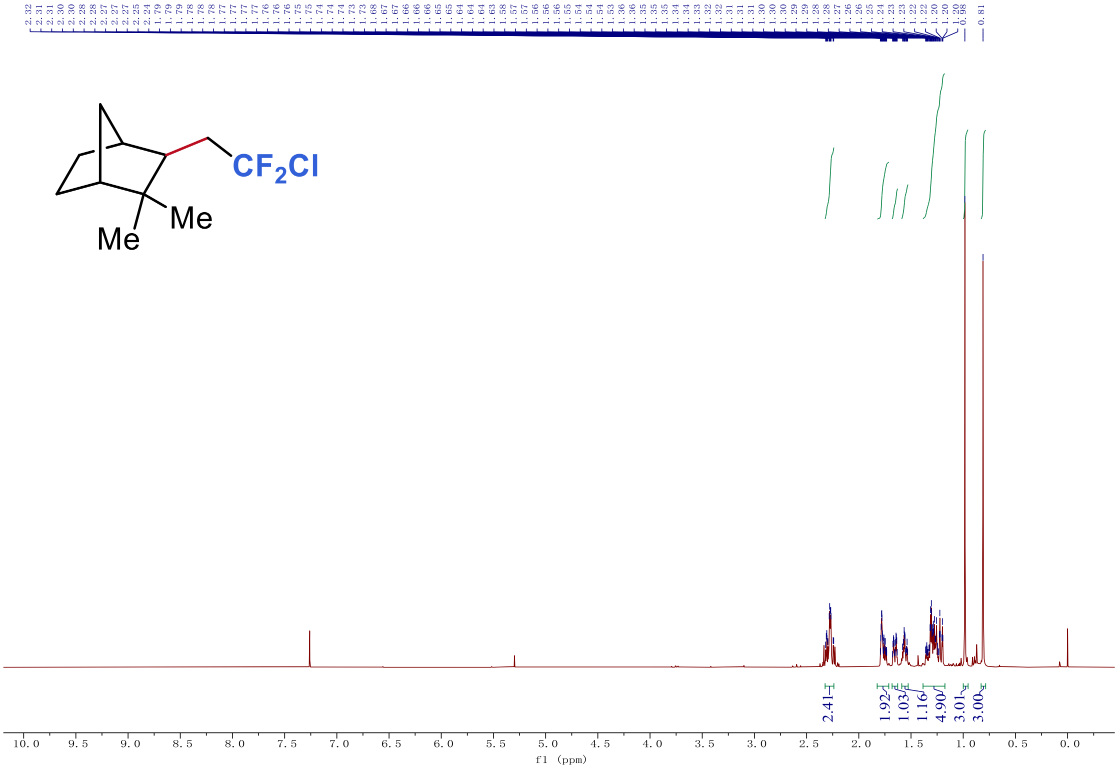

^{19}F NMR (377 MHz, $CDCl_3$) spectra for compound **1v**

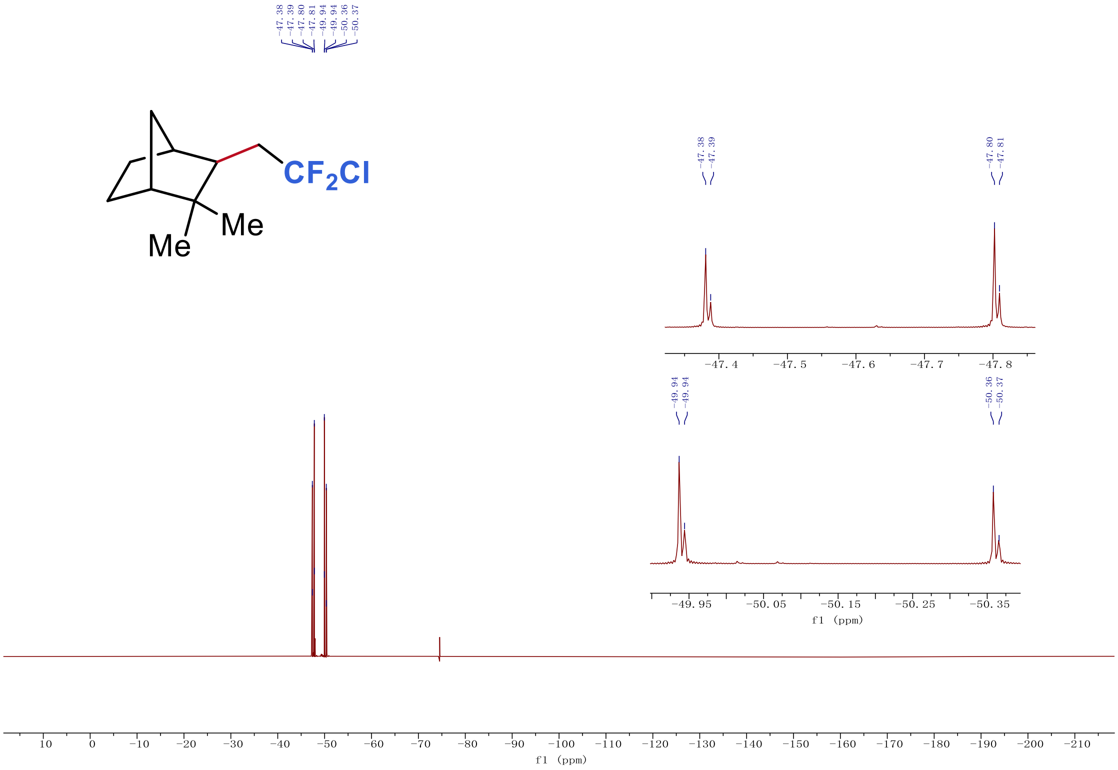

^{13}C NMR (126 MHz, $CDCl_3$) spectra for compound **1v**

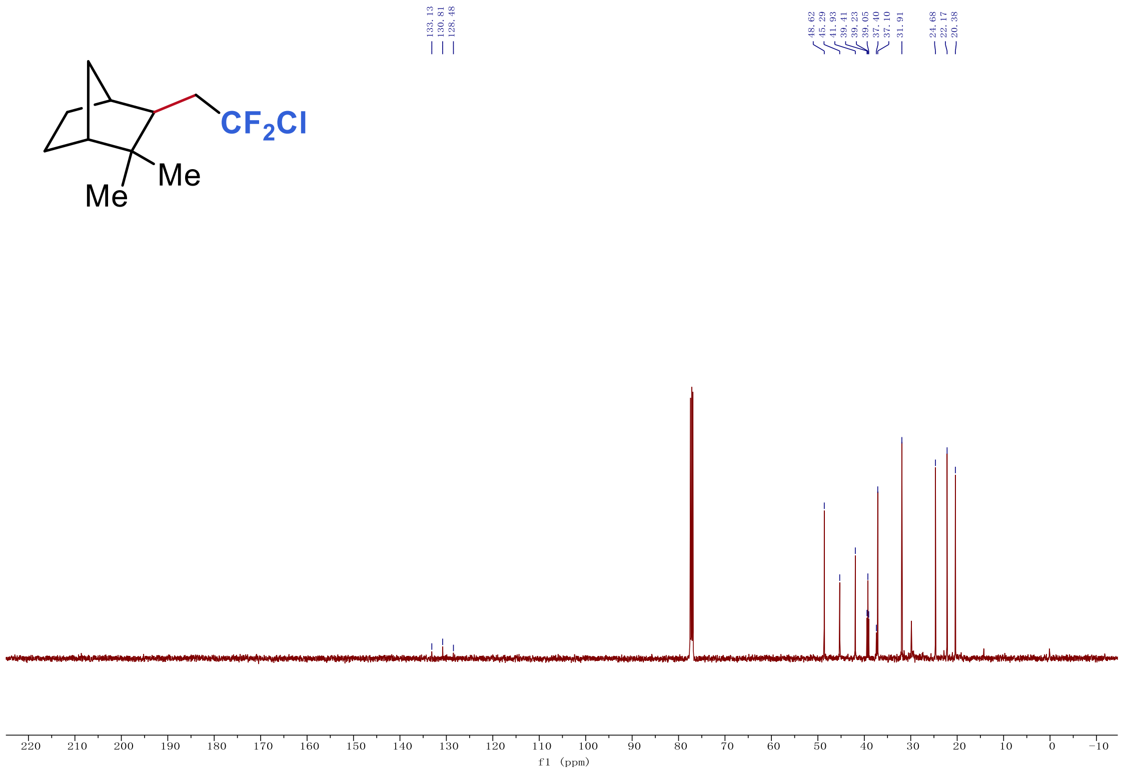

^{1}H NMR (400 MHz, $CDCl_3$) spectra for compound **1w**

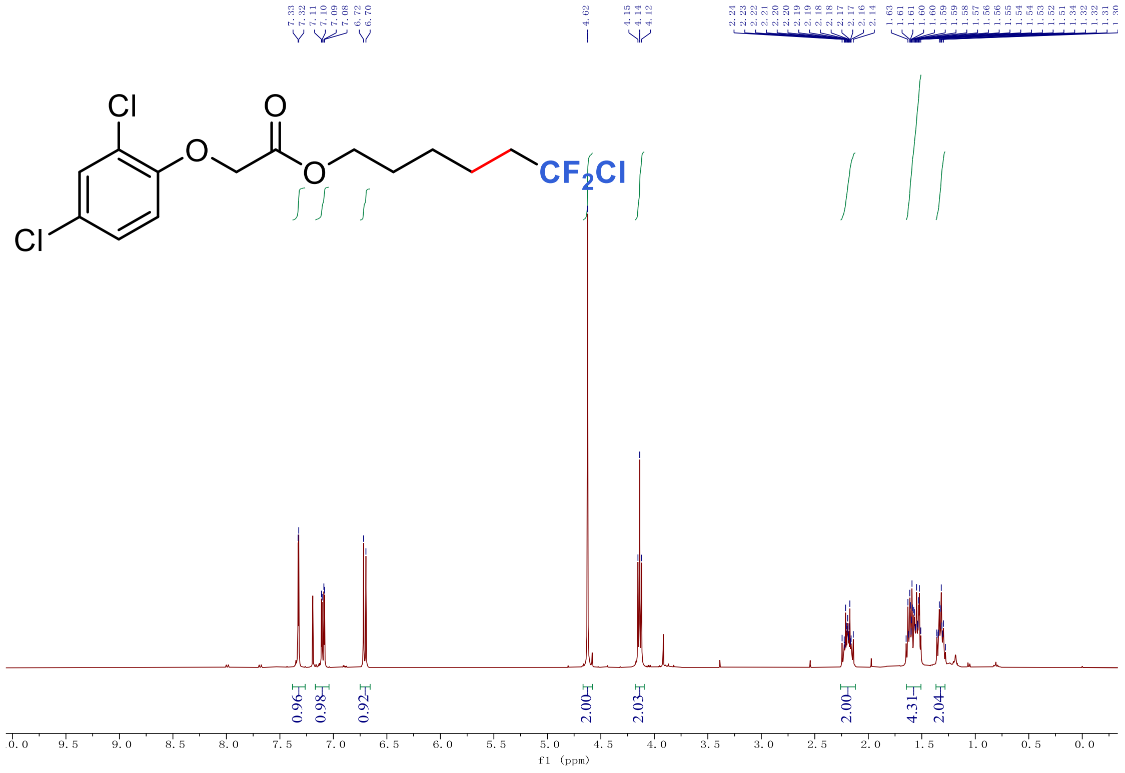

^{19}**F NMR** (377 MHz, $CDCl_3$) spectra for compound **1w**

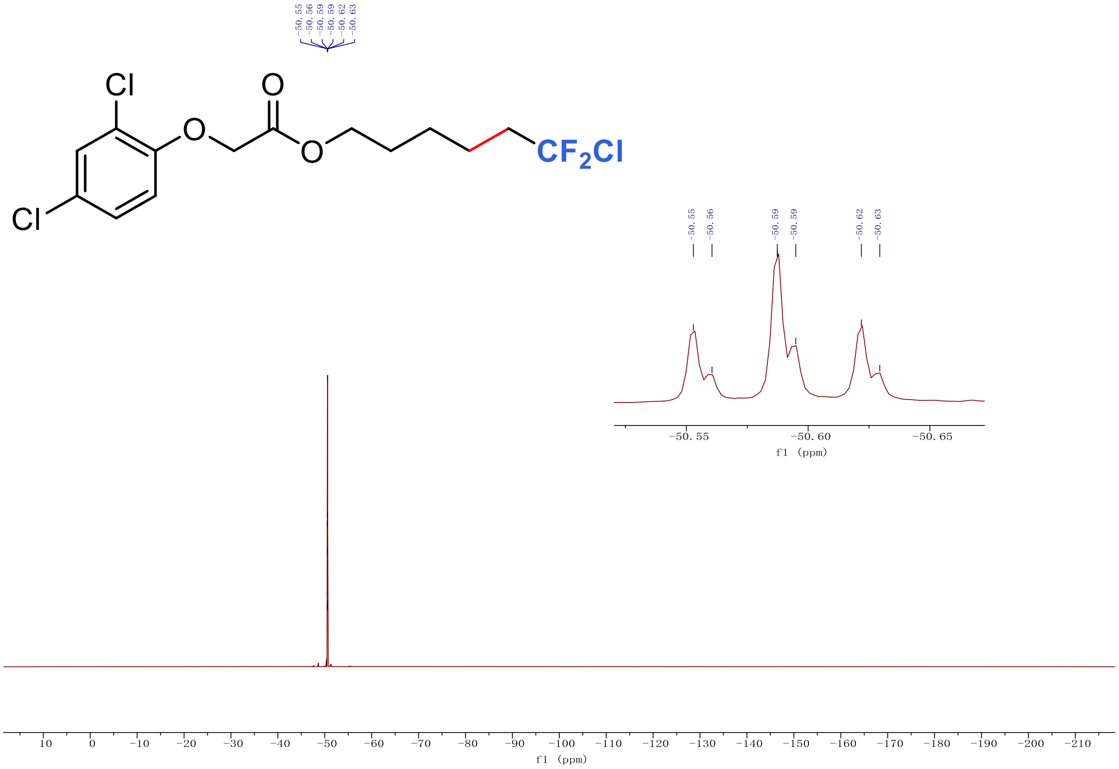

^{13}**C NMR** (126 MHz, $CDCl_3$) spectra for compound **1w**

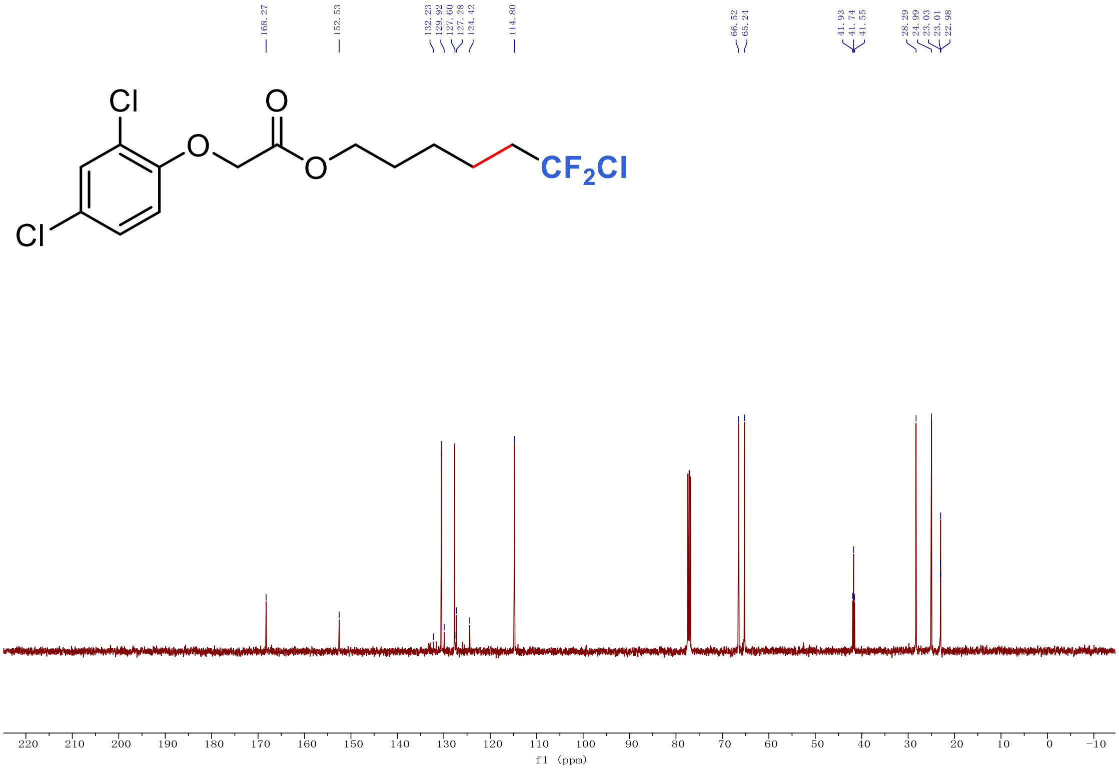

^{1}H NMR (400 MHz, $CDCl_3$) spectra for compound **1x**

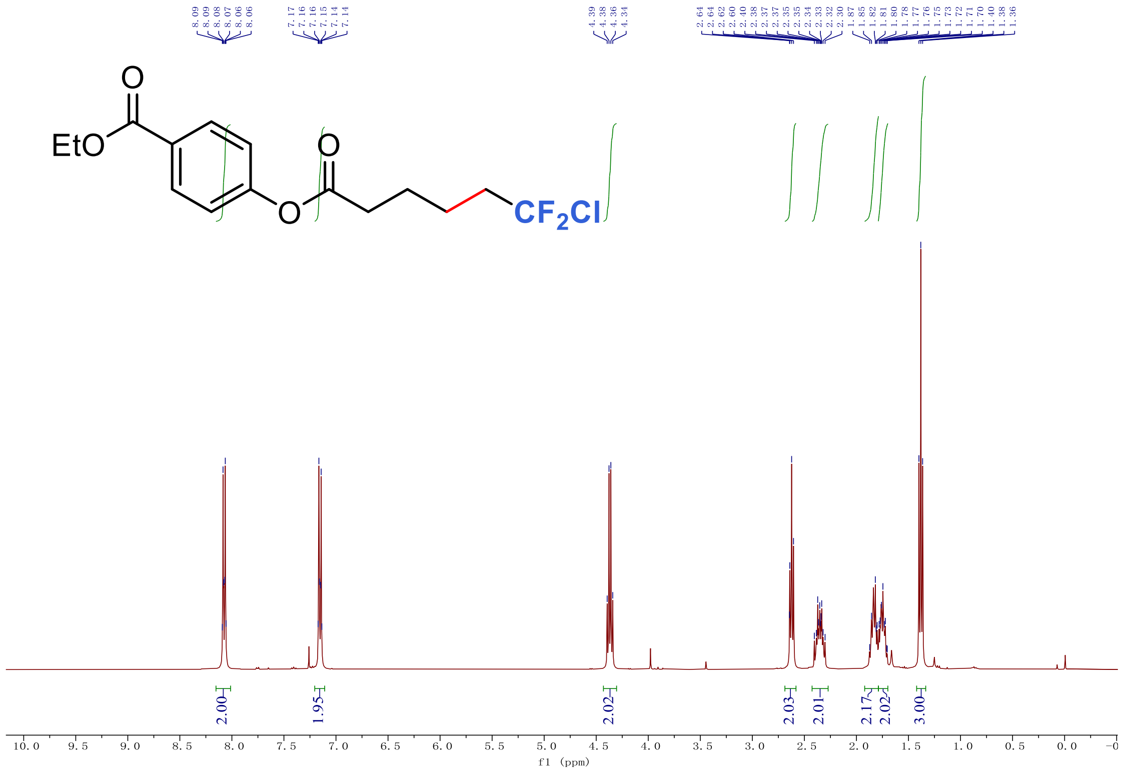

^{19}F NMR (377 MHz, $CDCl_3$) spectra for compound **1x**

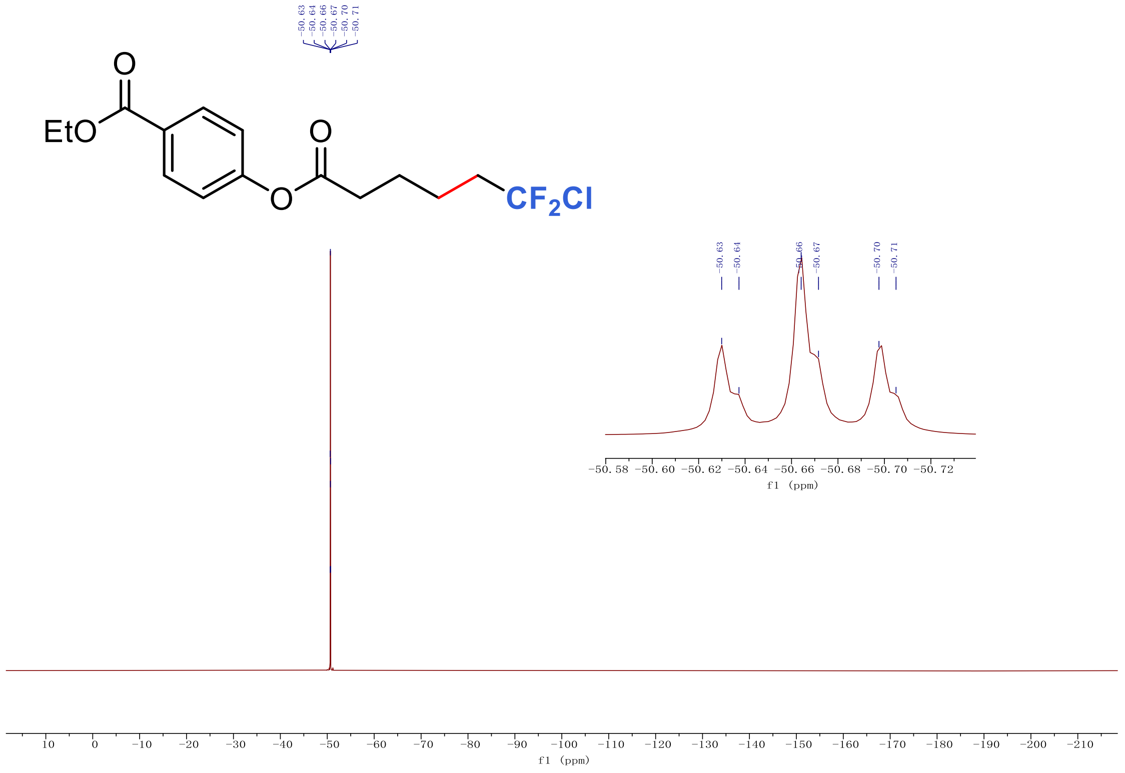

^{13}C NMR (126 MHz, $CDCl_3$) spectra for compound **1x**

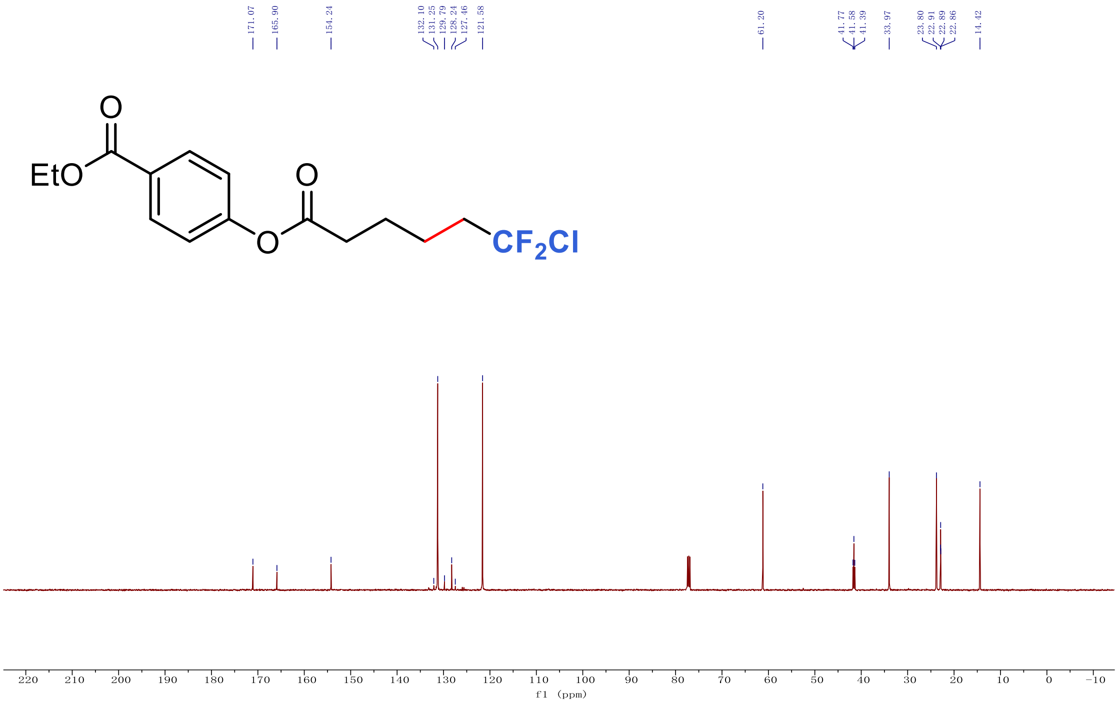

^{1}H NMR (400 MHz, $CDCl_3$) spectra for compound **1y**

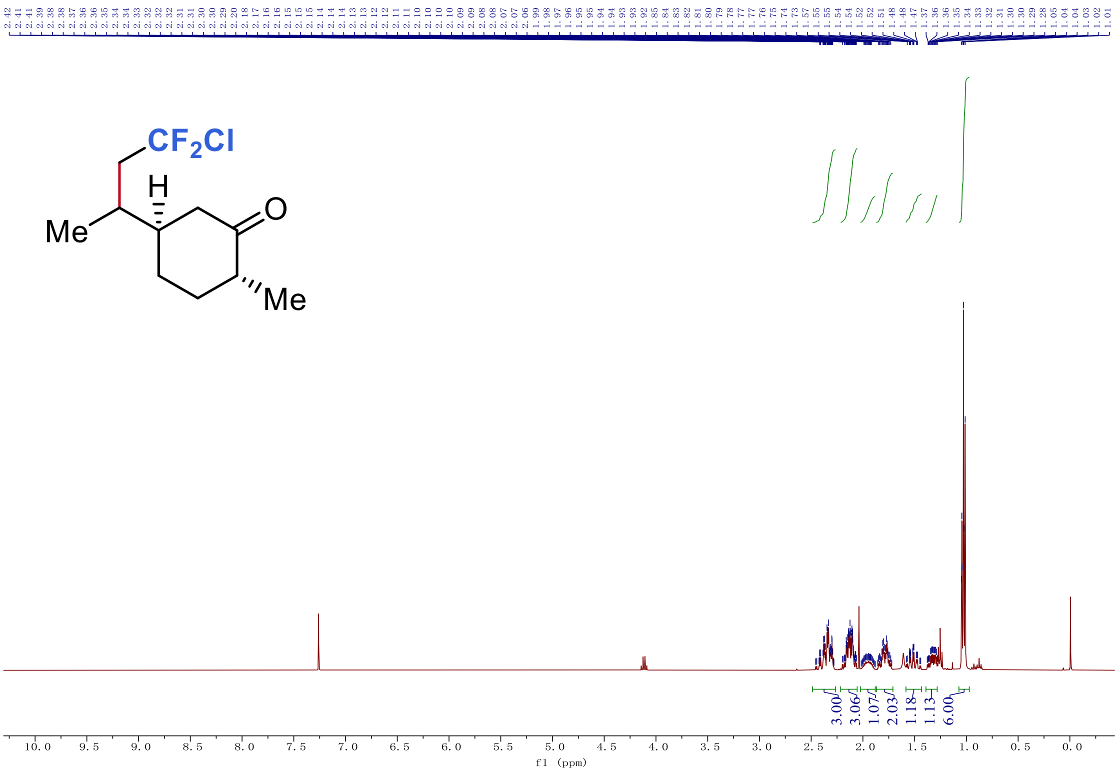

^{19}F NMR (377 MHz, $CDCl_3$) spectra for compound **1y**

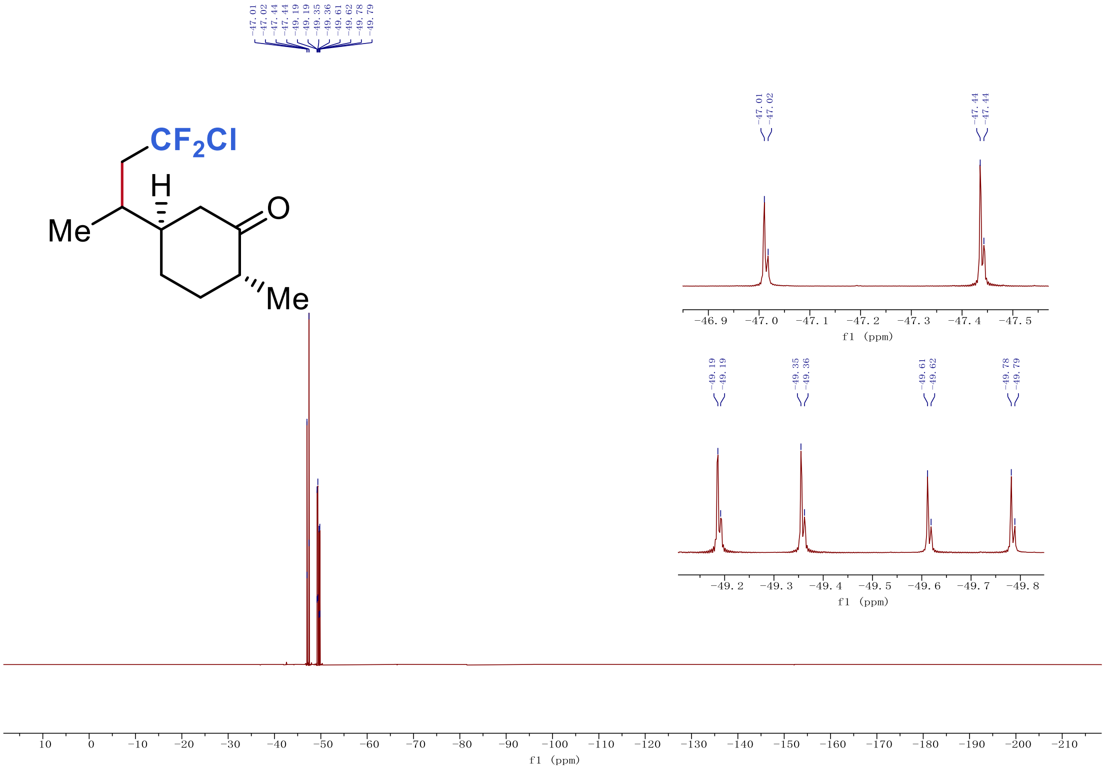

^{13}C NMR (126 MHz, $CDCl_3$) spectra for compound **1y**

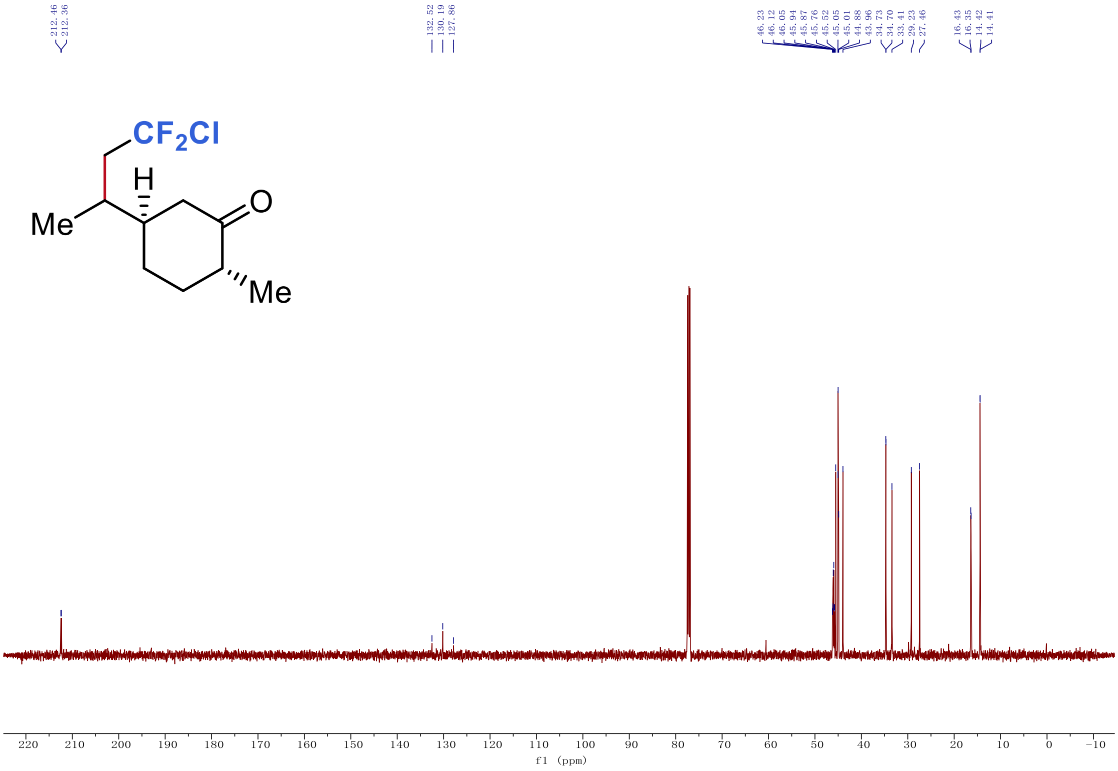

^{1}H NMR (400 MHz, $CDCl_3$) spectra for compound **1z**

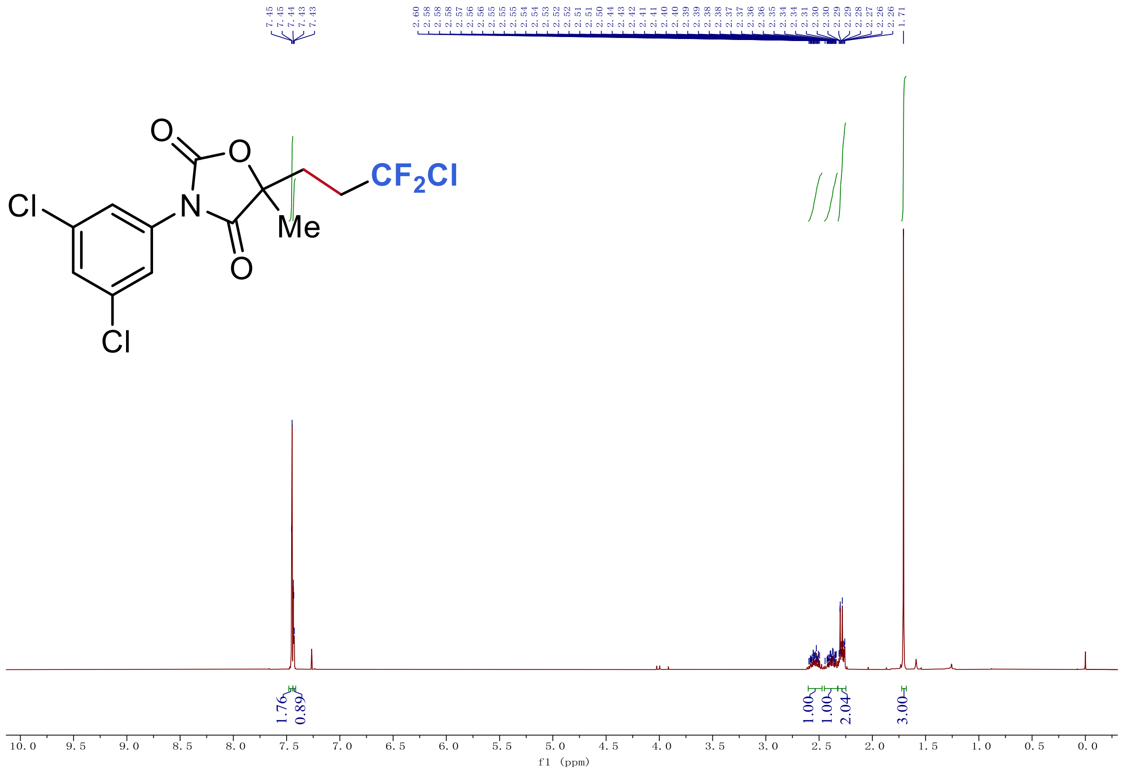

^{19}F NMR (377 MHz, $CDCl_3$) spectra for compound **1z**

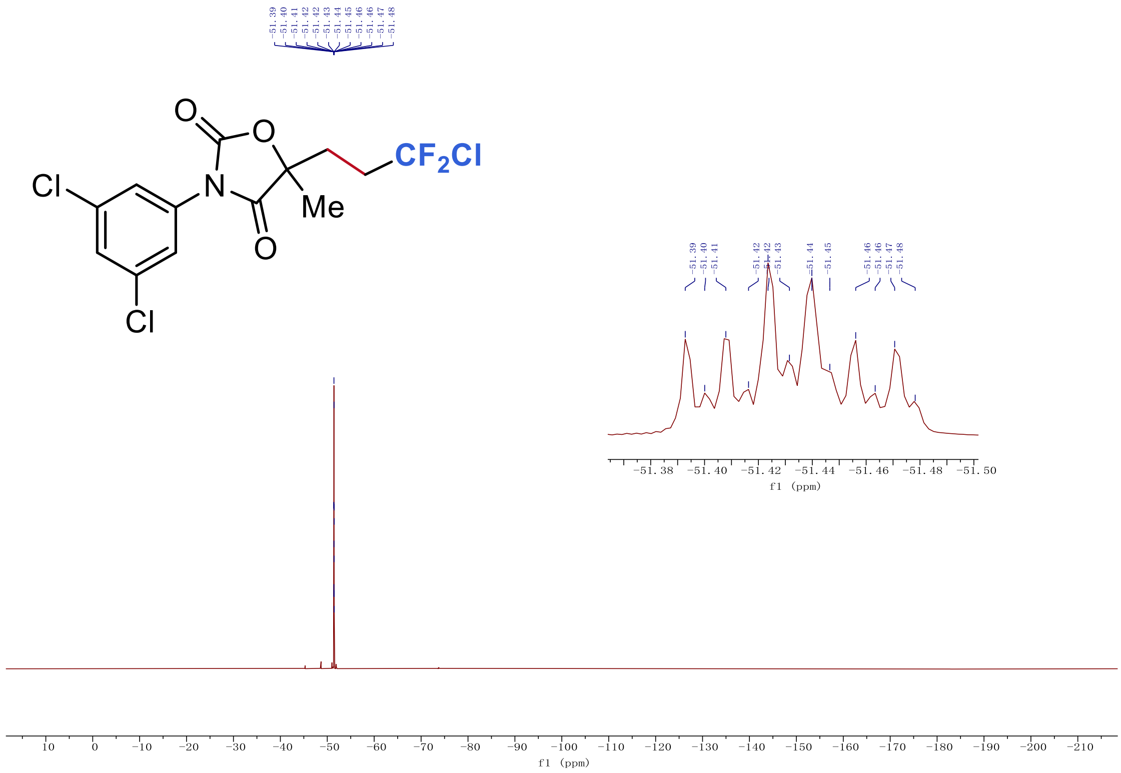

^{13}C NMR (126 MHz, $CDCl_3$) spectra for compound **1z**

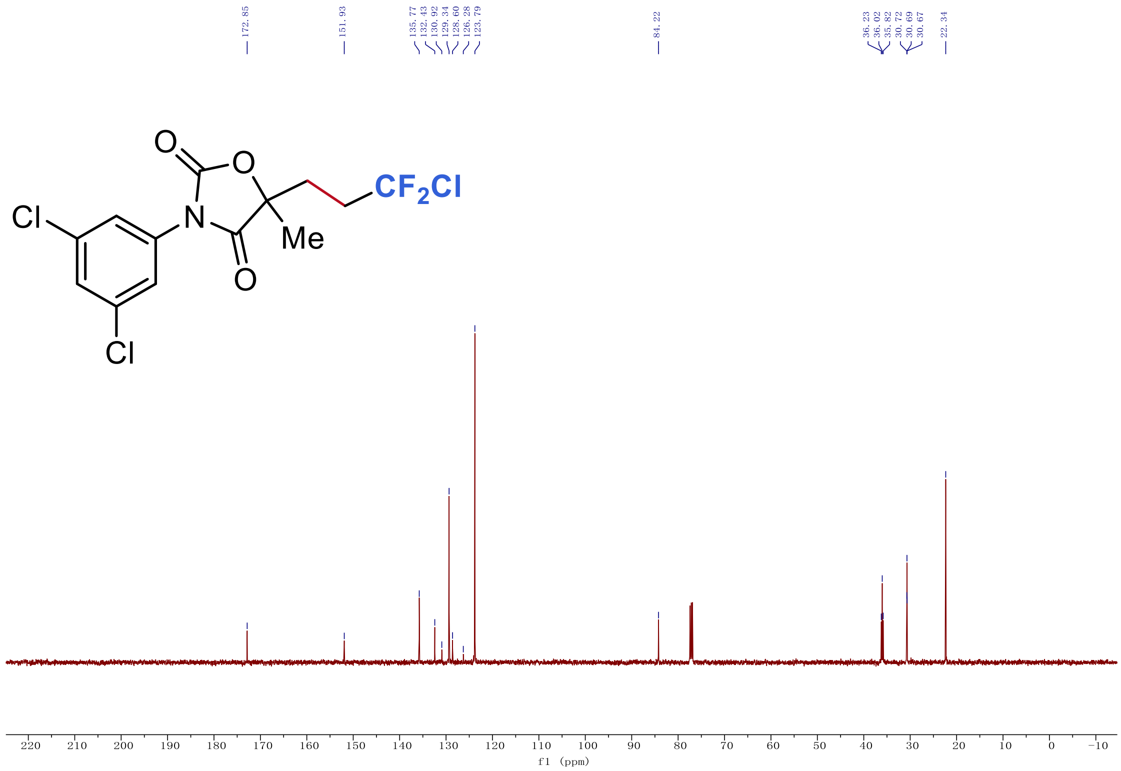

^{1}H NMR (400 MHz, $CDCl_3$) spectra for compound **1aa**

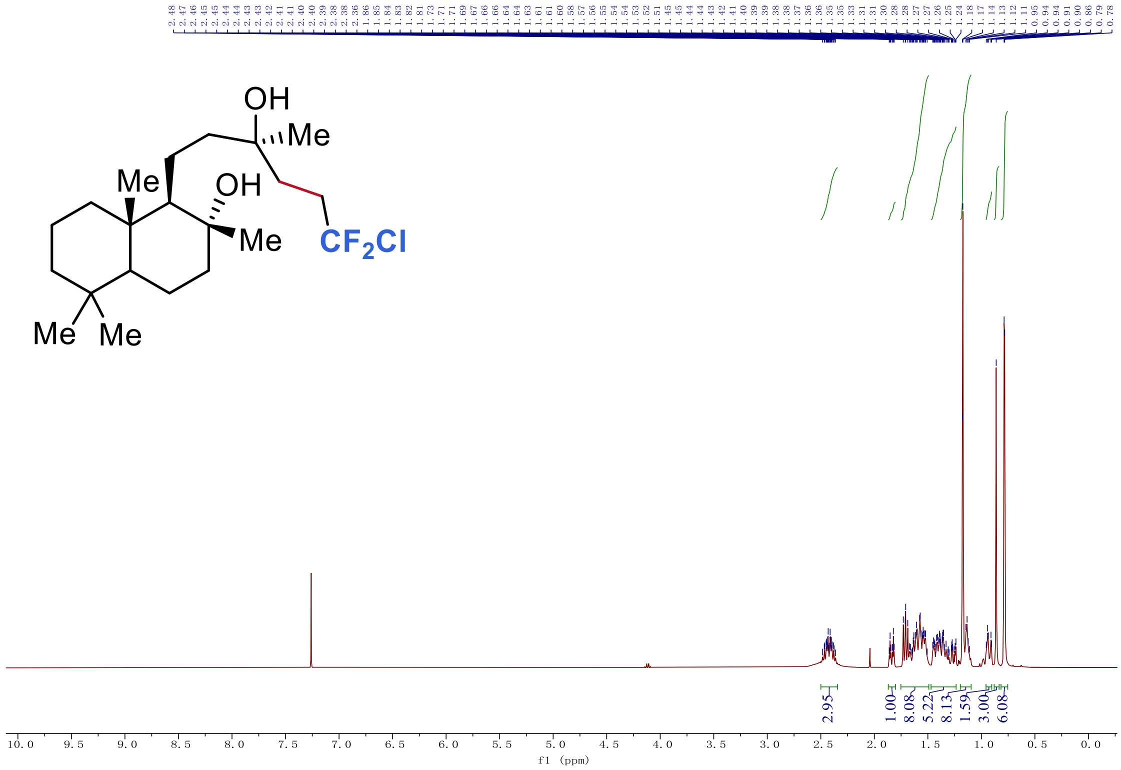

^{19}F NMR (377 MHz, $CDCl_3$) spectra for compound **1aa**

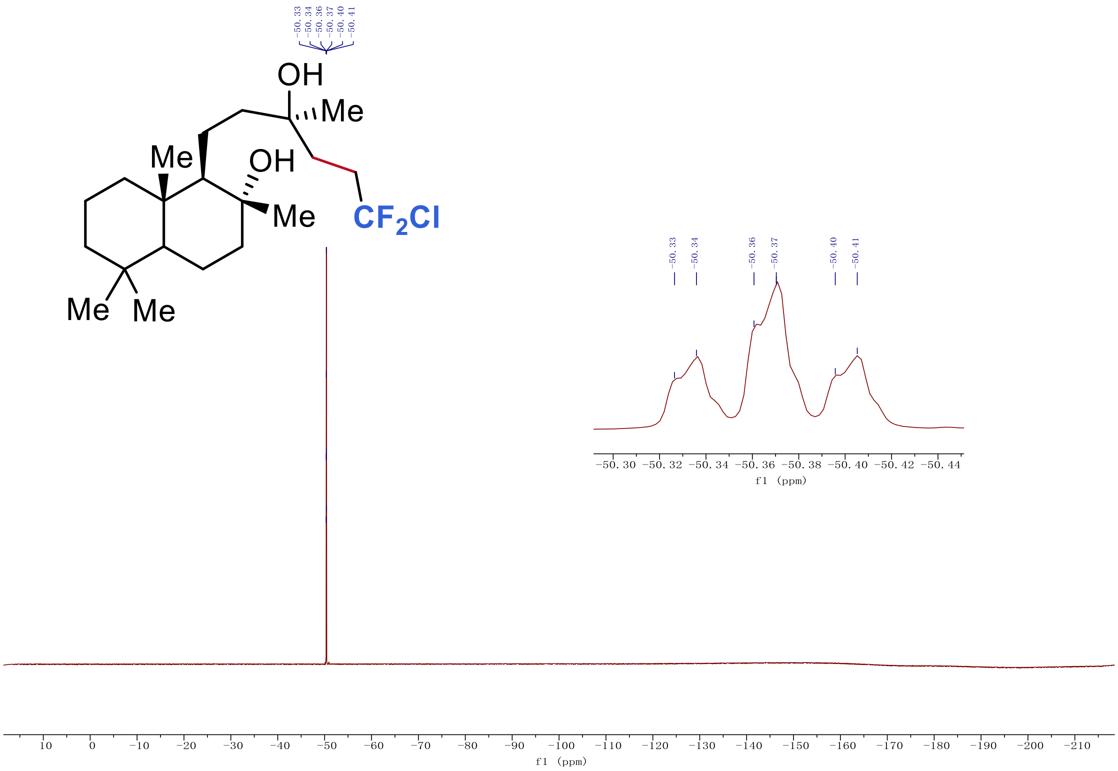

^{13}C NMR (126 MHz, $CDCl_3$) spectra for compound **1aa**

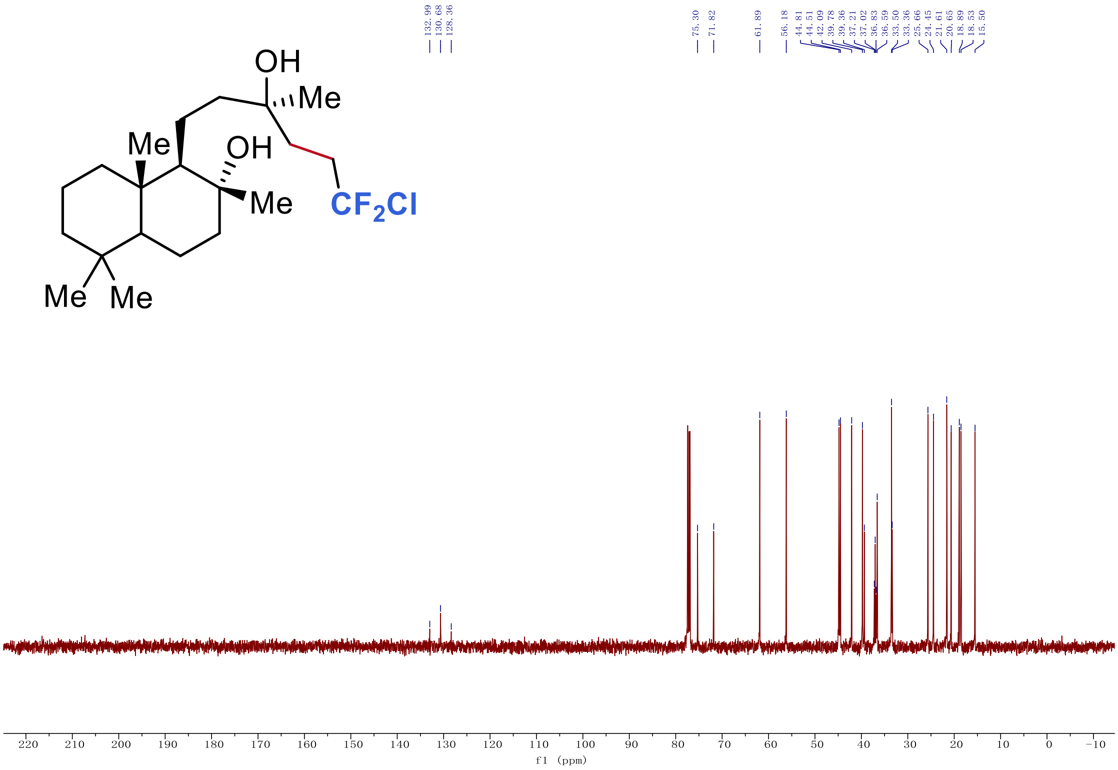

^{1}H NMR (400 MHz, $CDCl_3$) spectra for compound **1ab**

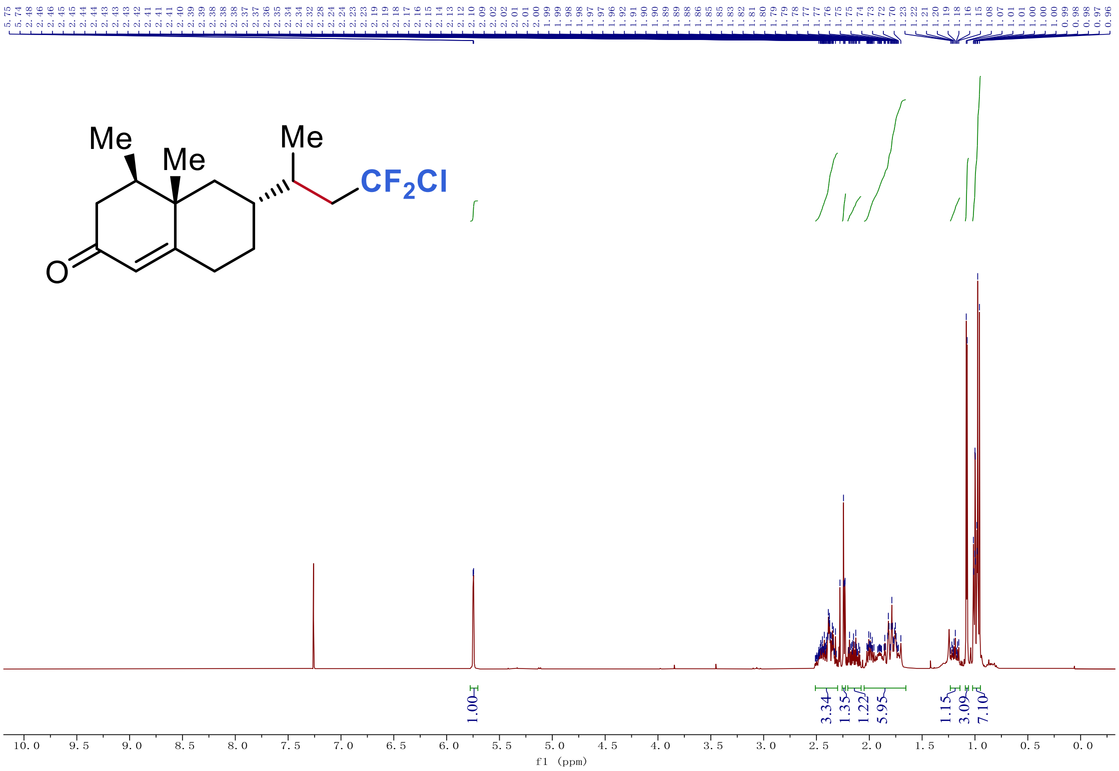

^{19}F NMR (377 MHz, $CDCl_3$) spectra for compound **1ab**

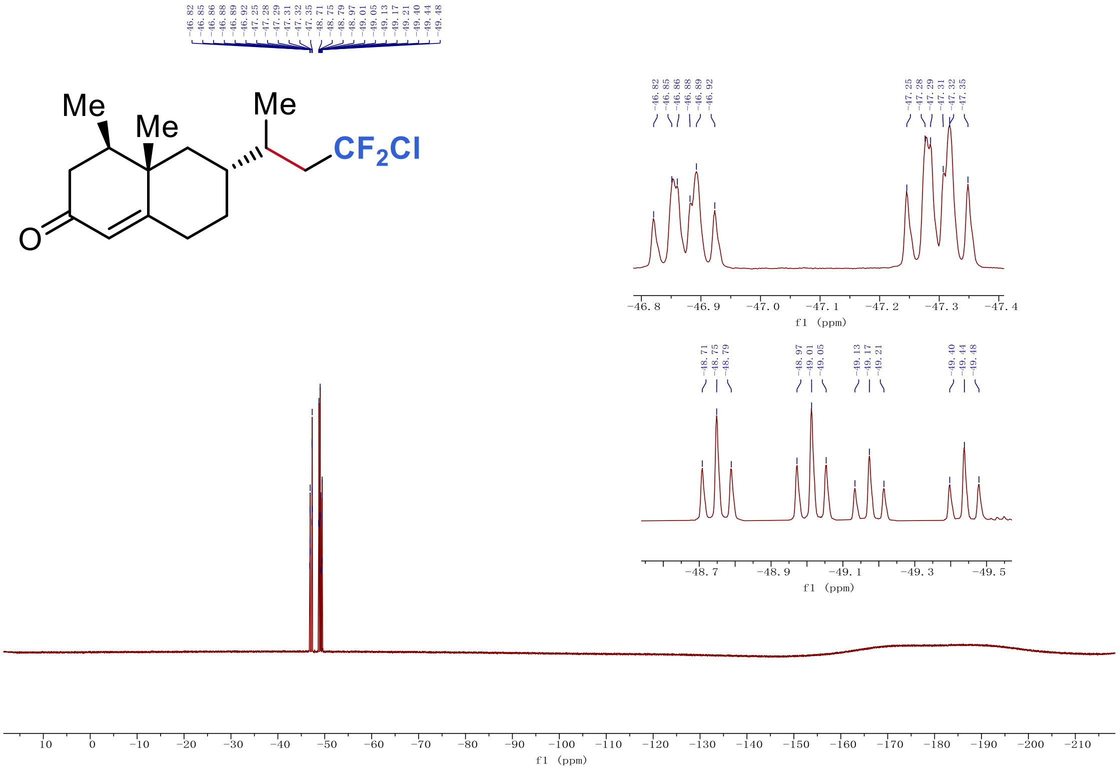

^{13}C NMR (126 MHz, $CDCl_3$) spectra for compound **1ab**

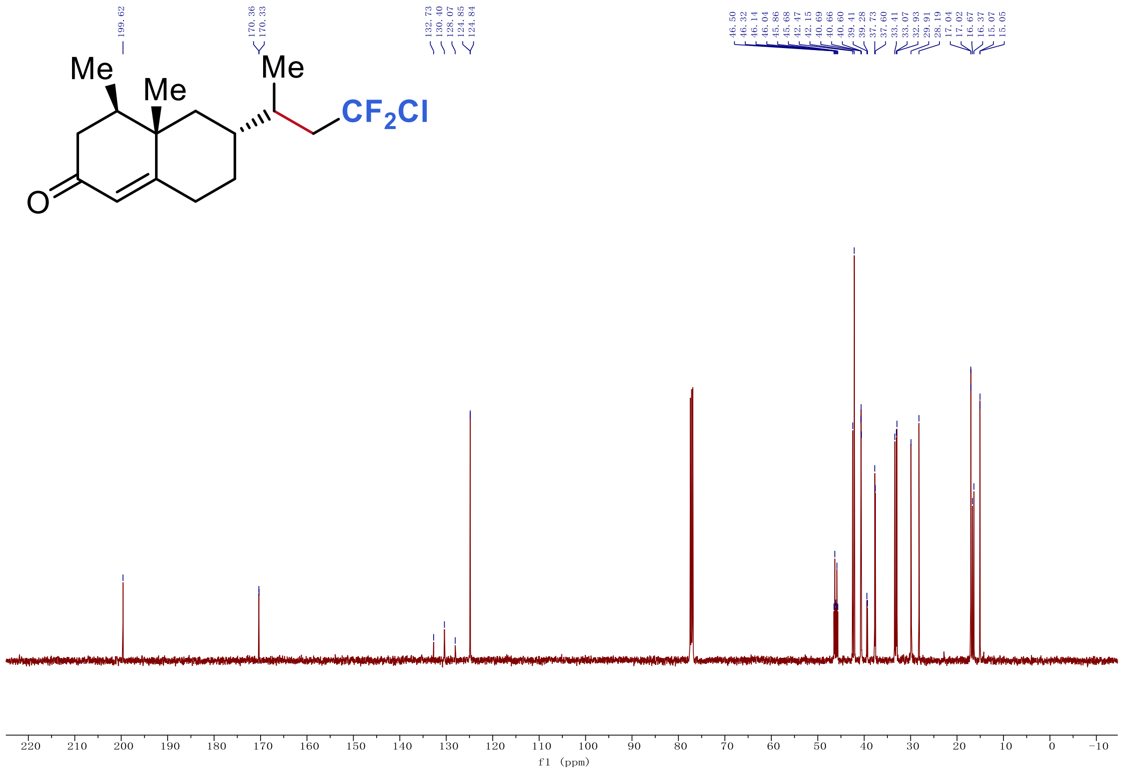

^{1}H NMR (400 MHz, $CDCl_3$) spectra for compound **1ac**

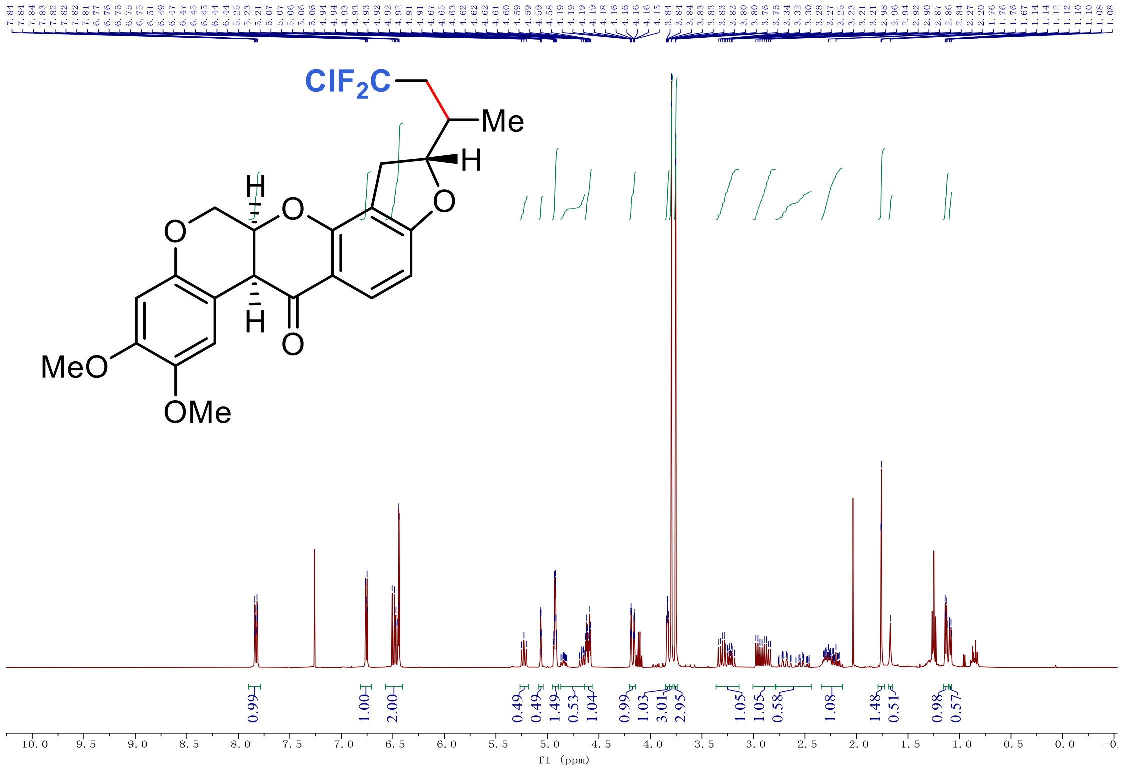

^{19}**F NMR** (377 MHz, $CDCl_3$) spectra for compound **1ac**

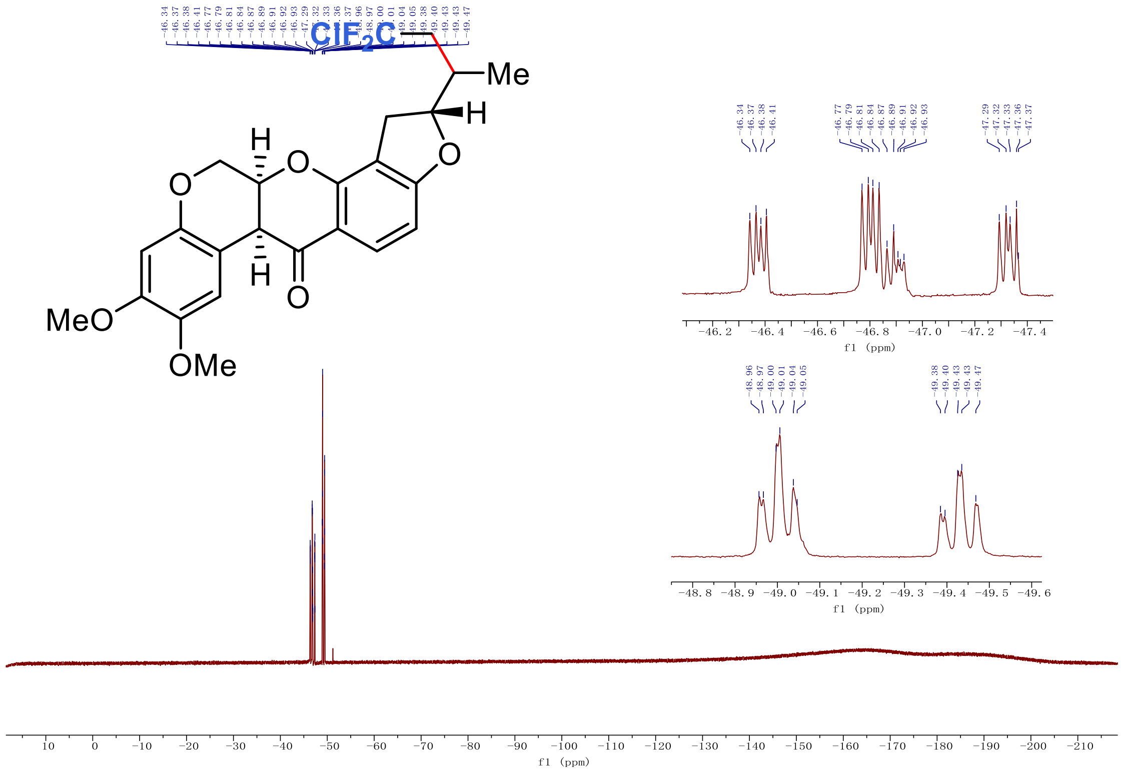

^{13}**C NMR** (126 MHz, $CDCl_3$) spectra for compound **1ac**

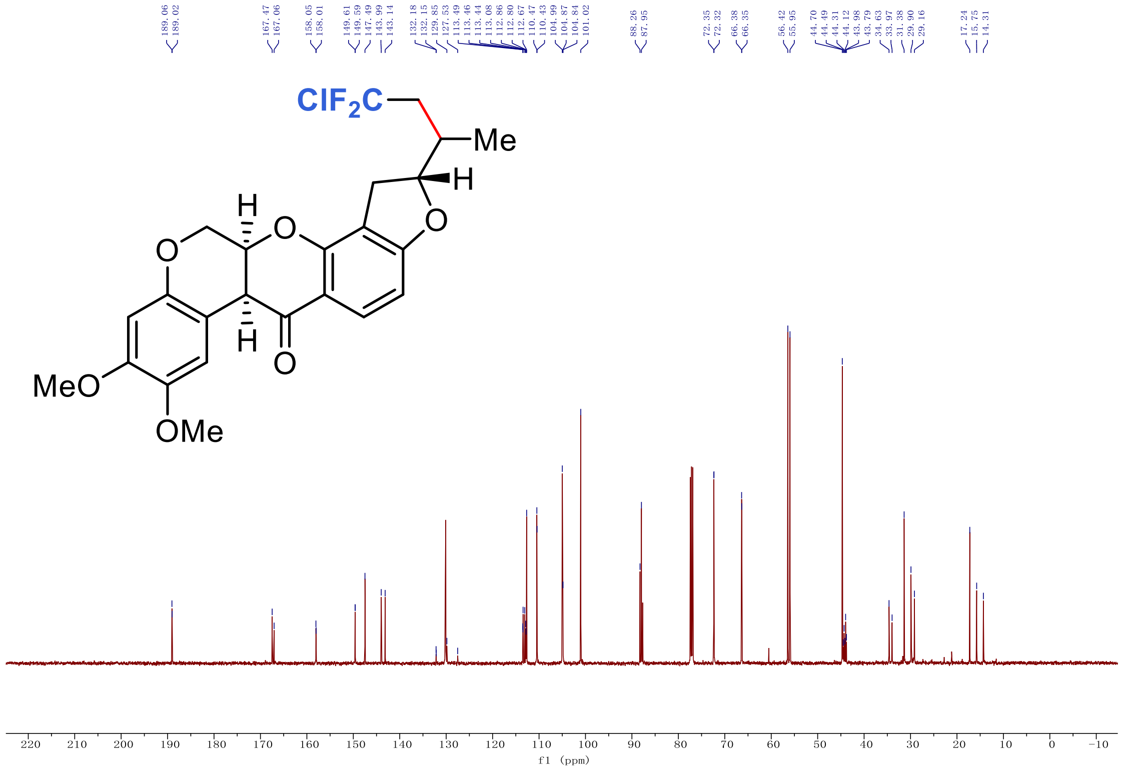

^{1}H NMR (400 MHz, $CDCl_3$) spectra for compound **1ad**

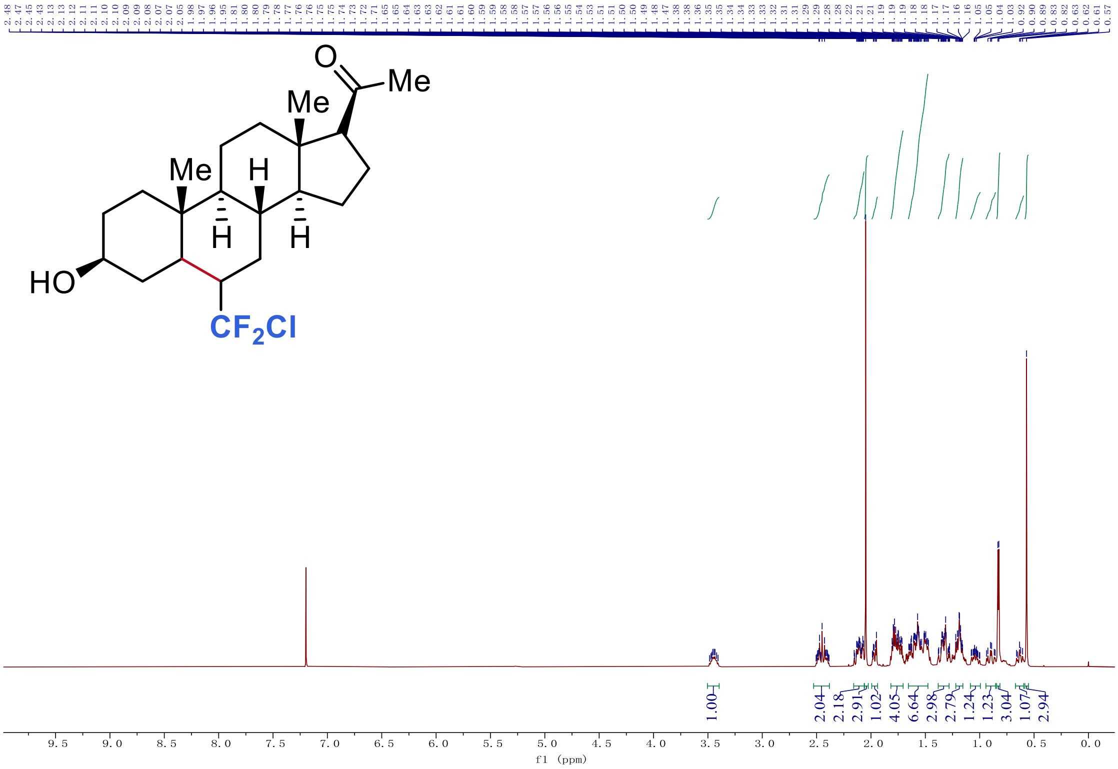

^{19}F NMR (377 MHz, $CDCl_3$) spectra for compound **1ad**

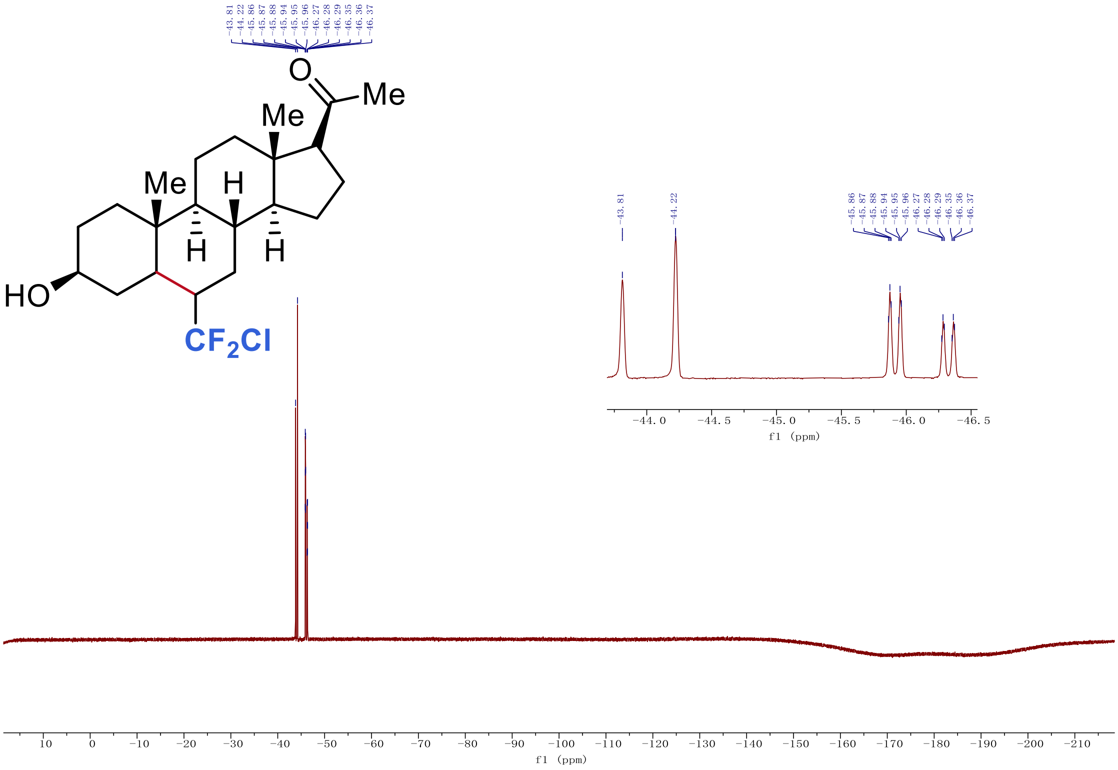

^{13}C NMR (126 MHz, $CDCl_3$) spectra for compound **1ad**

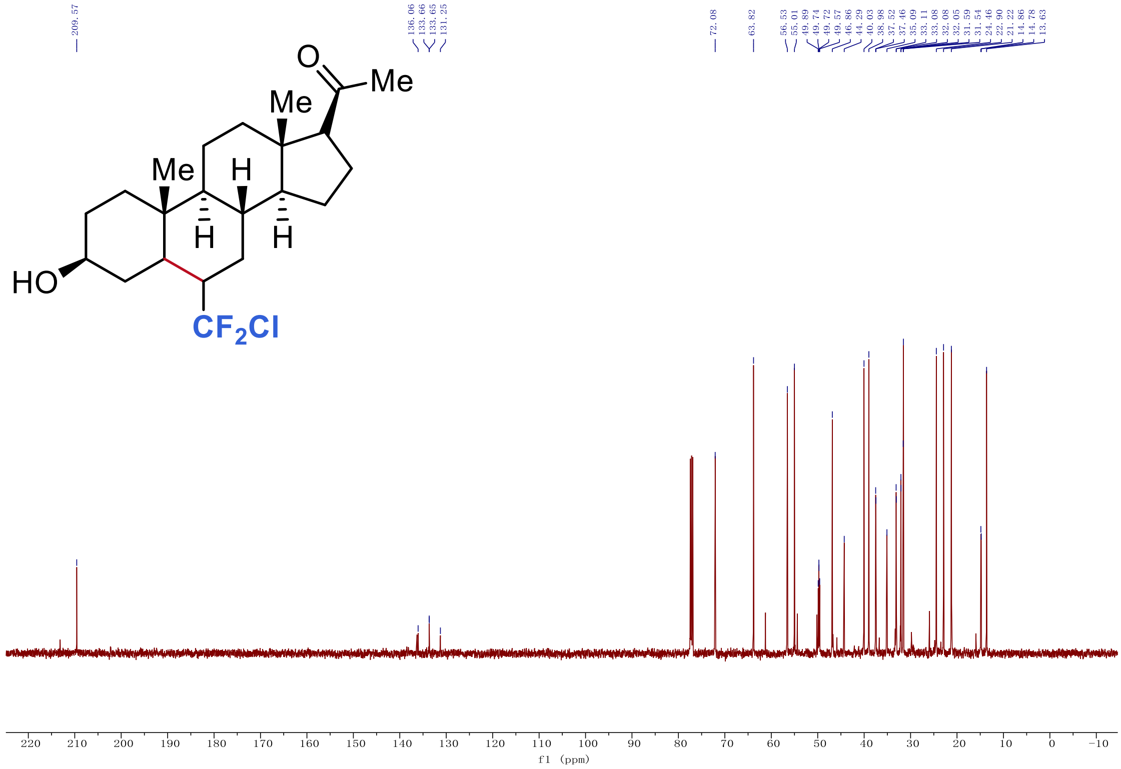

^{1}H NMR (400 MHz, $CDCl_3$) spectra for compound **1ae**

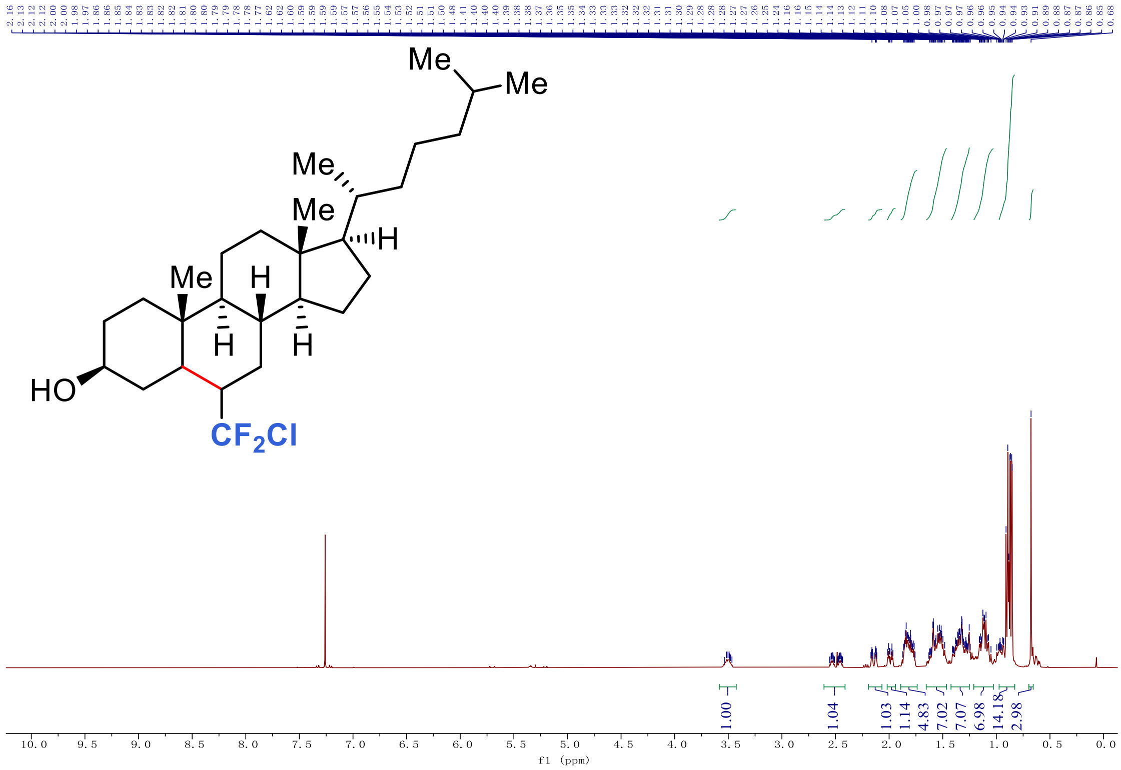

^{19}F NMR (377 MHz, $CDCl_3$) spectra for compound **1ae**

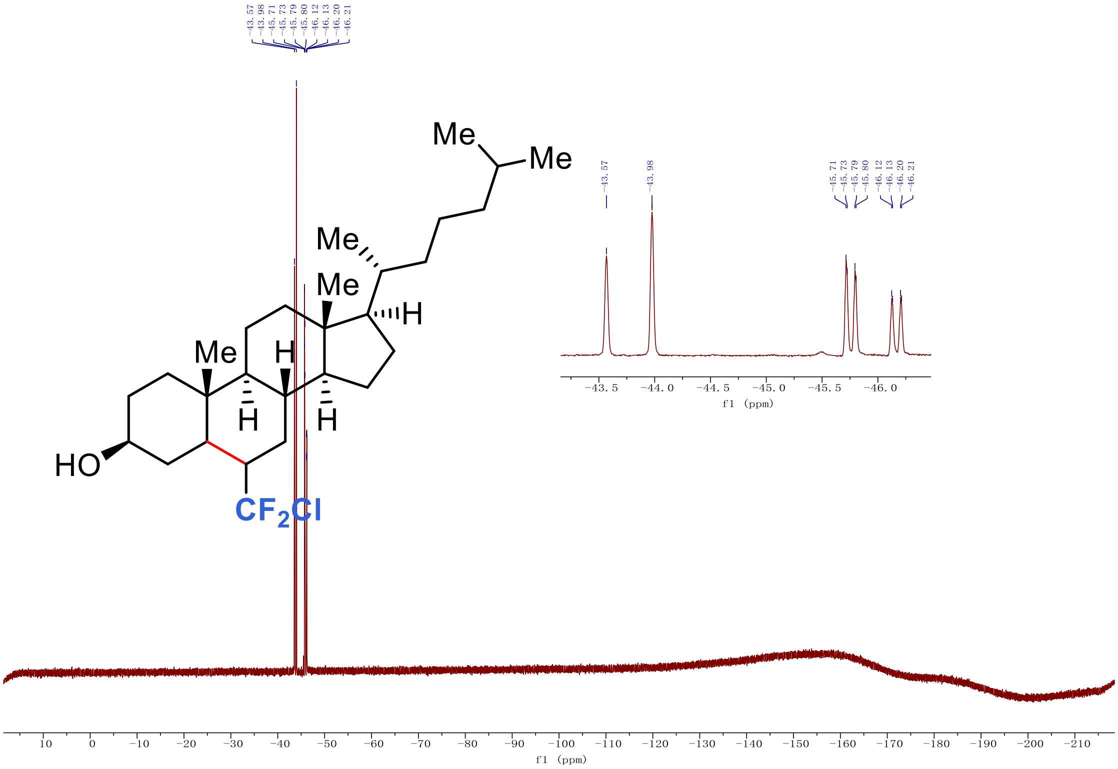

^{13}C NMR (126 MHz, $CDCl_3$) spectra for compound **1ae**

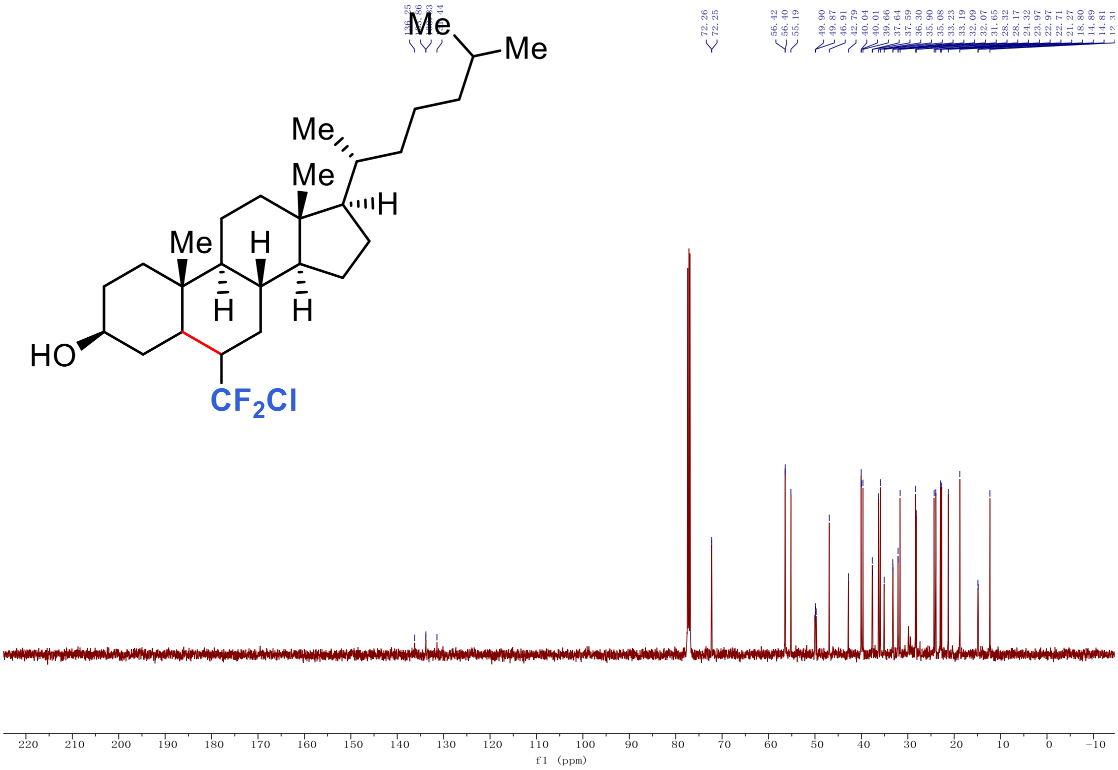

^{1}H NMR (400 MHz, $CDCl_3$) spectra for compound **1af**

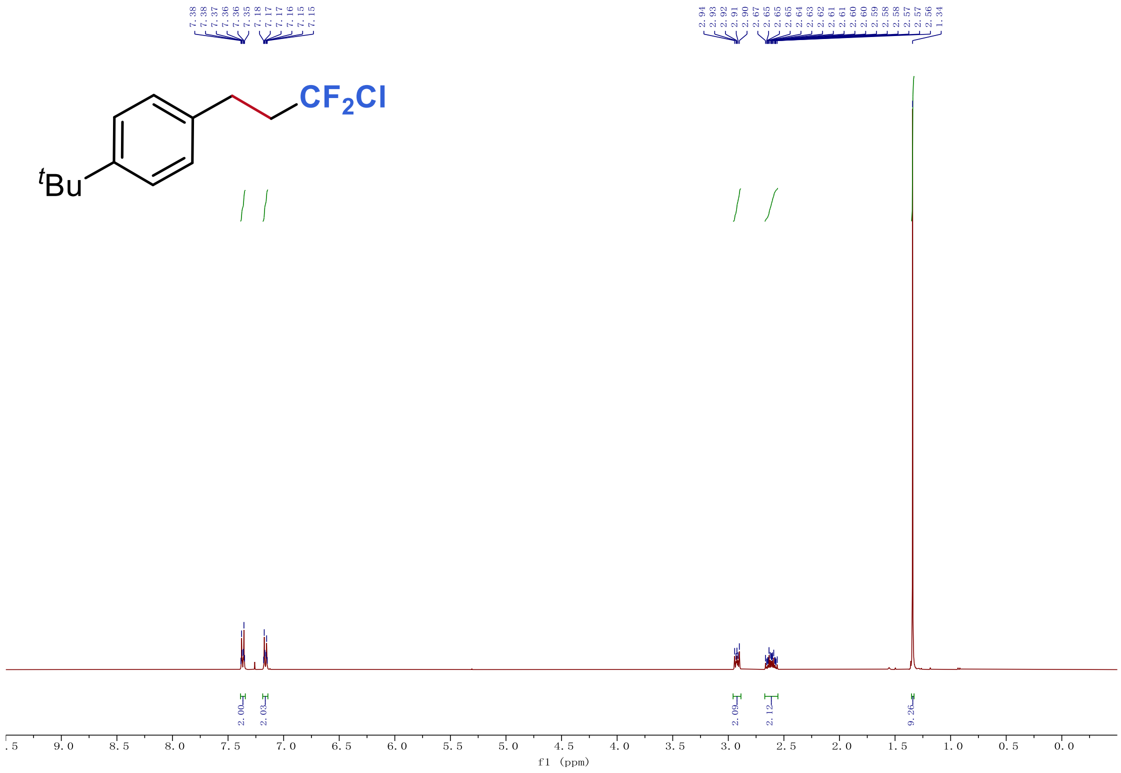

^{19}F NMR (377 MHz, $CDCl_3$) spectra for compound **1af**

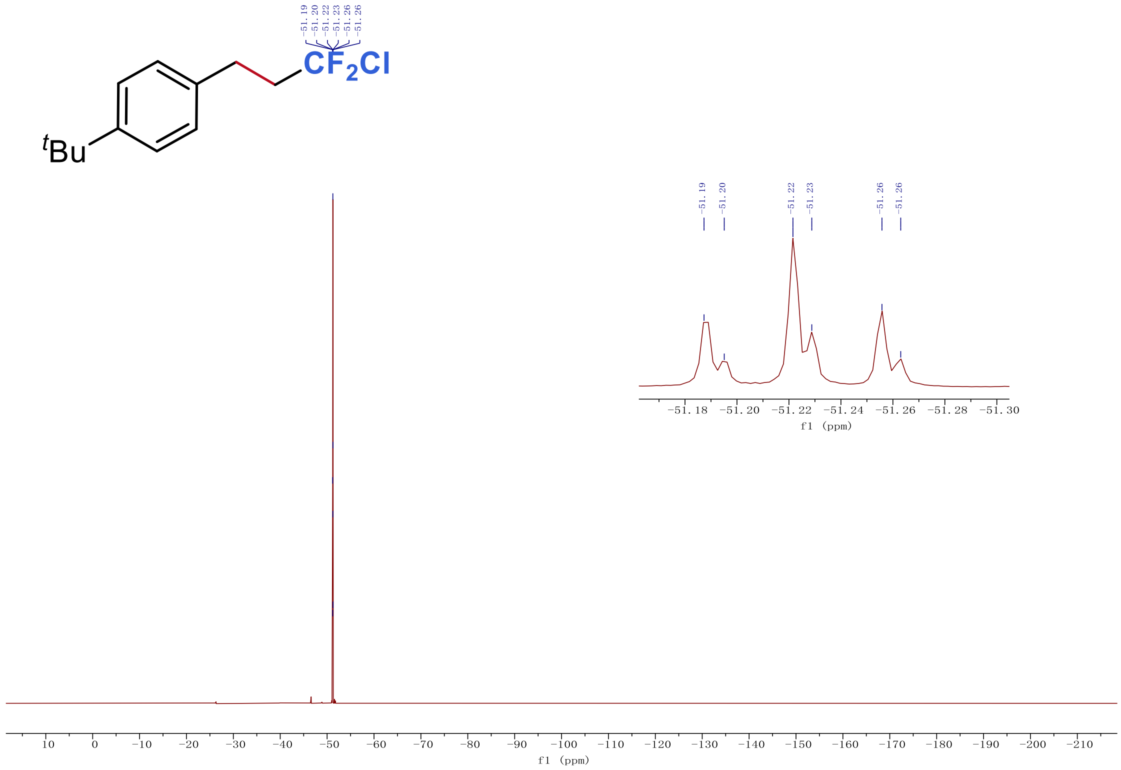

^{13}C NMR (126 MHz, $CDCl_3$) spectra for compound **1af**

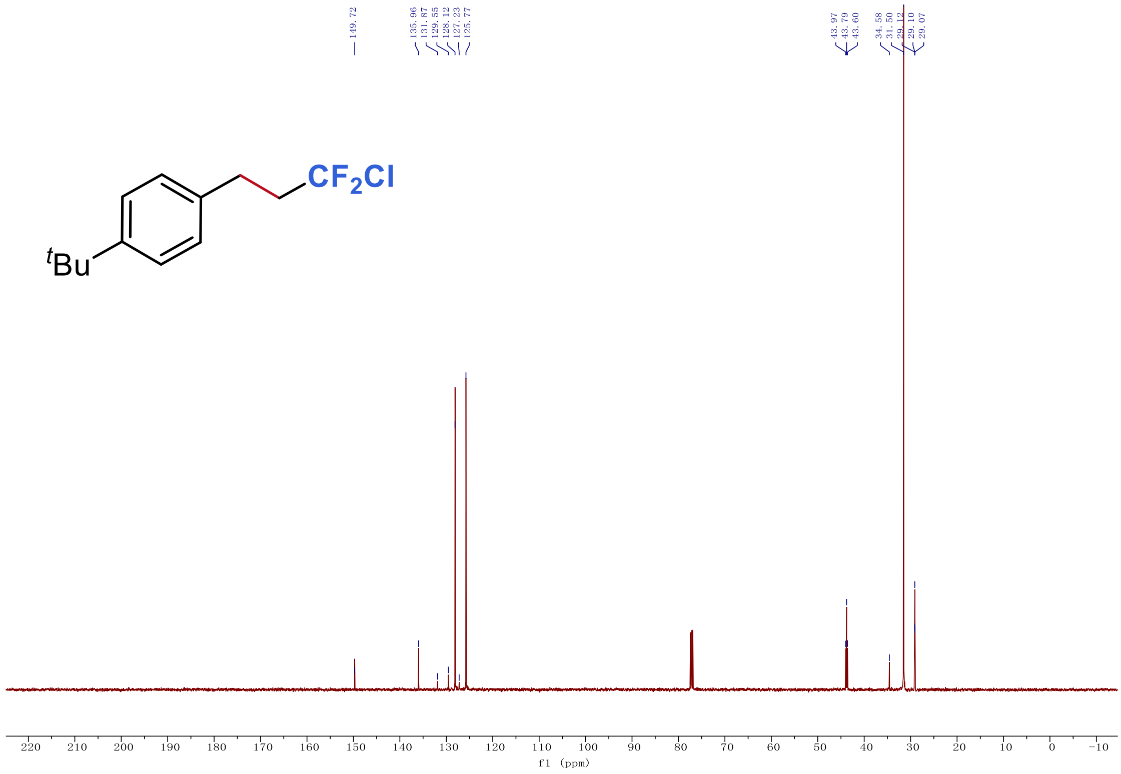

^{1}H NMR (400 MHz, $CDCl_3$) spectra for compound **1ag**

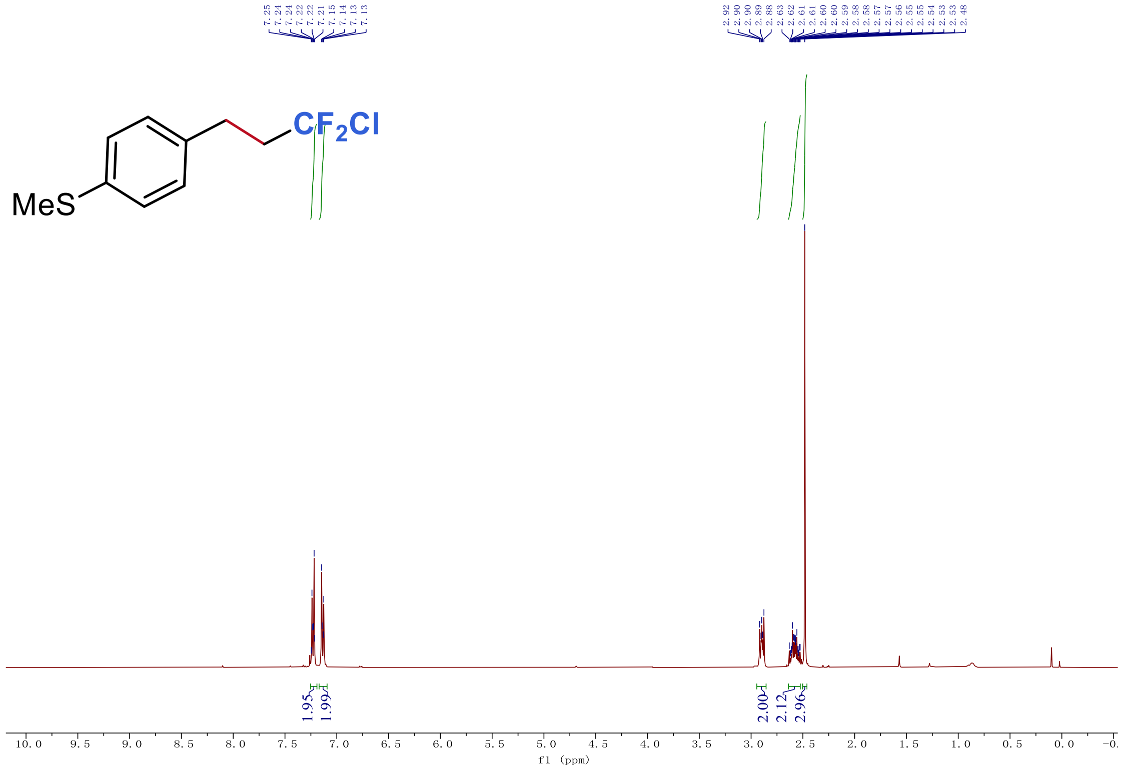

^{19}F **NMR** (377 MHz, $CDCl_3$) spectra for compound **1ag**

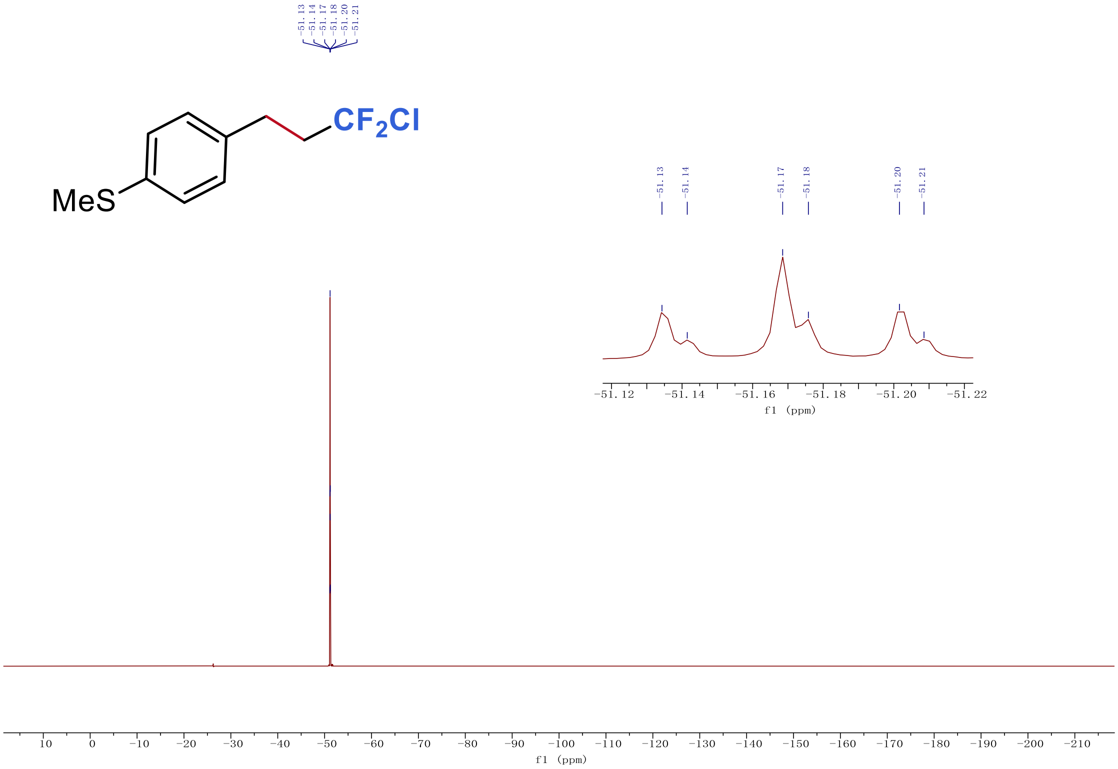

^{13}C **NMR** (126 MHz, $CDCl_3$) spectra for compound **1ag**

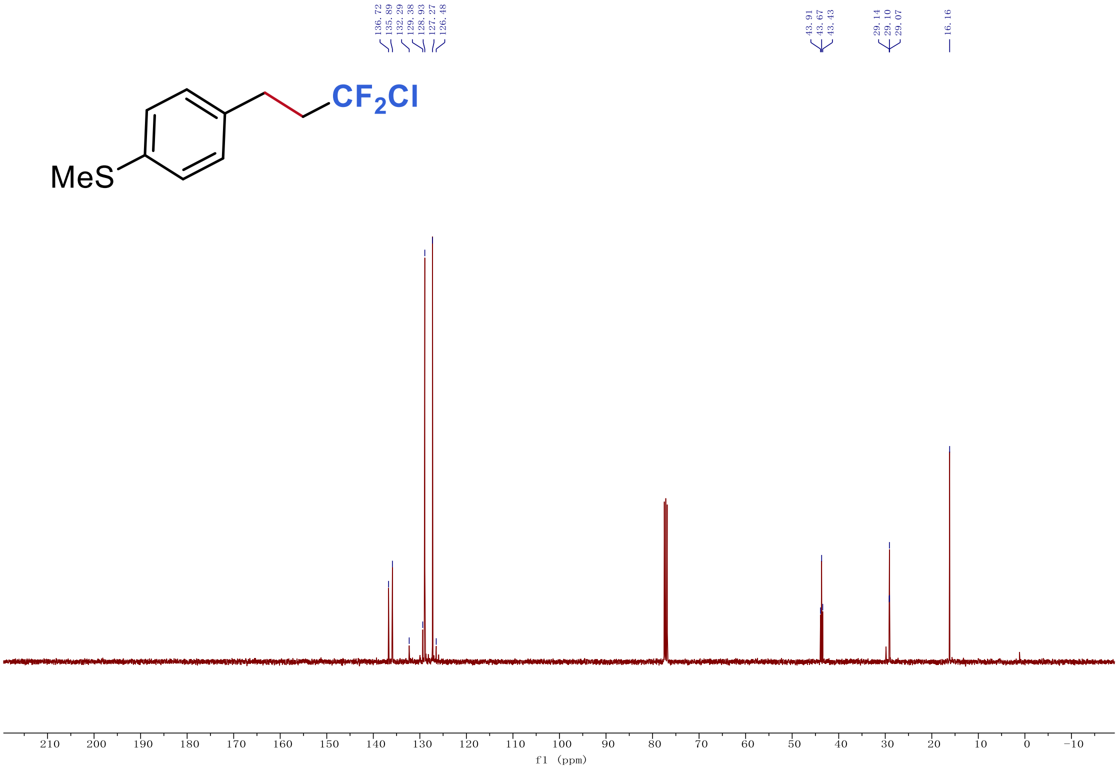

^{1}H NMR (400 MHz, $CDCl_3$) spectra for compound **1ah**

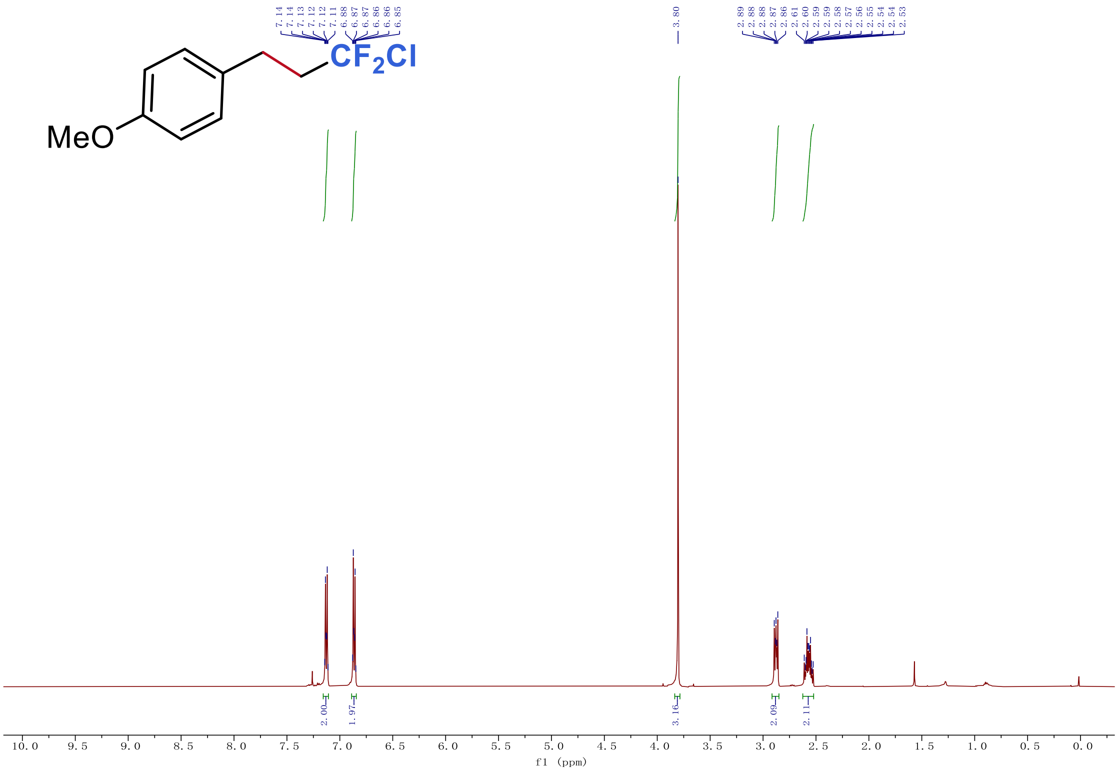

^{19}F NMR (377 MHz, $CDCl_3$) spectra for compound **1ah**

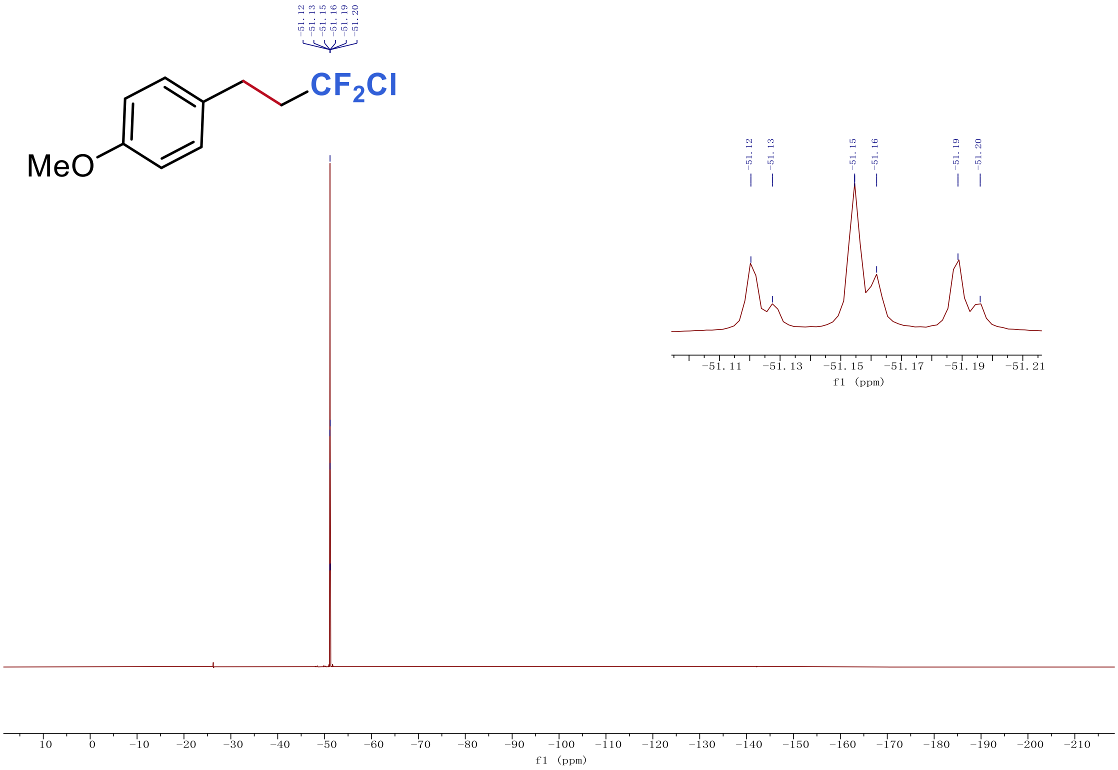

[13] **C NMR** (126 MHz, $CDCl_3$) spectra for compound **1ah**

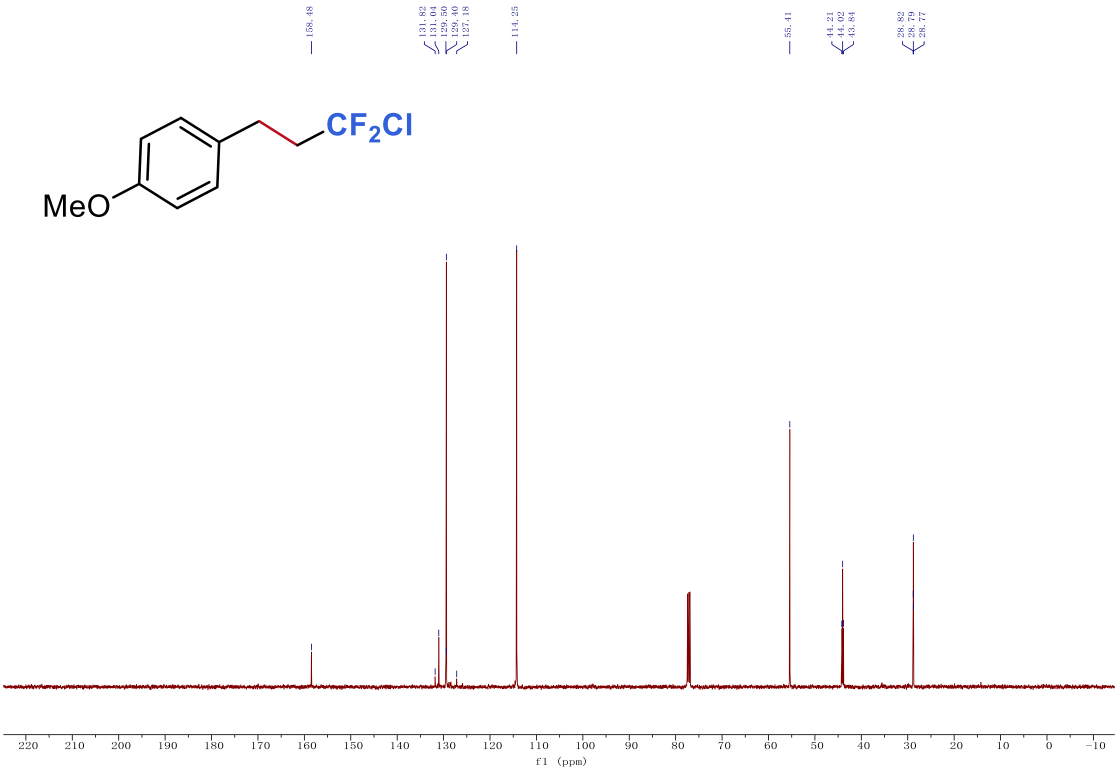

[1] **H NMR** (400 MHz, $CDCl_3$) spectra for compound **1ai**

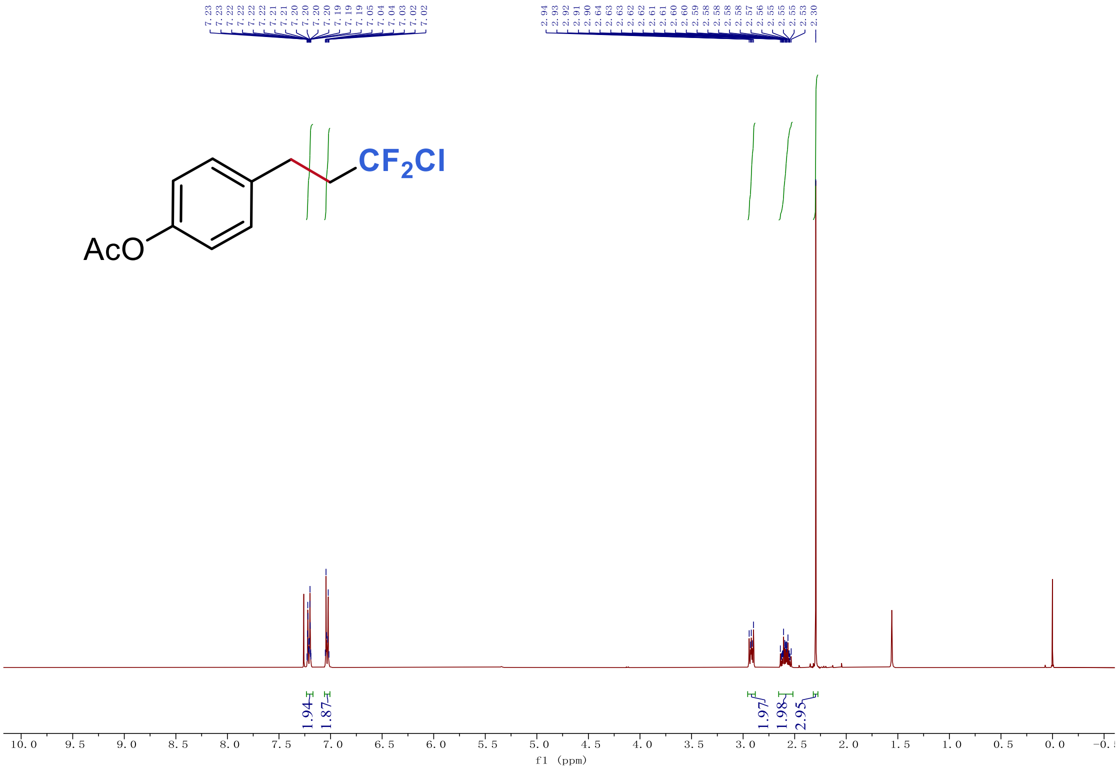

^{19}F NMR (377 MHz, $CDCl_3$) spectra for compound **1ai**

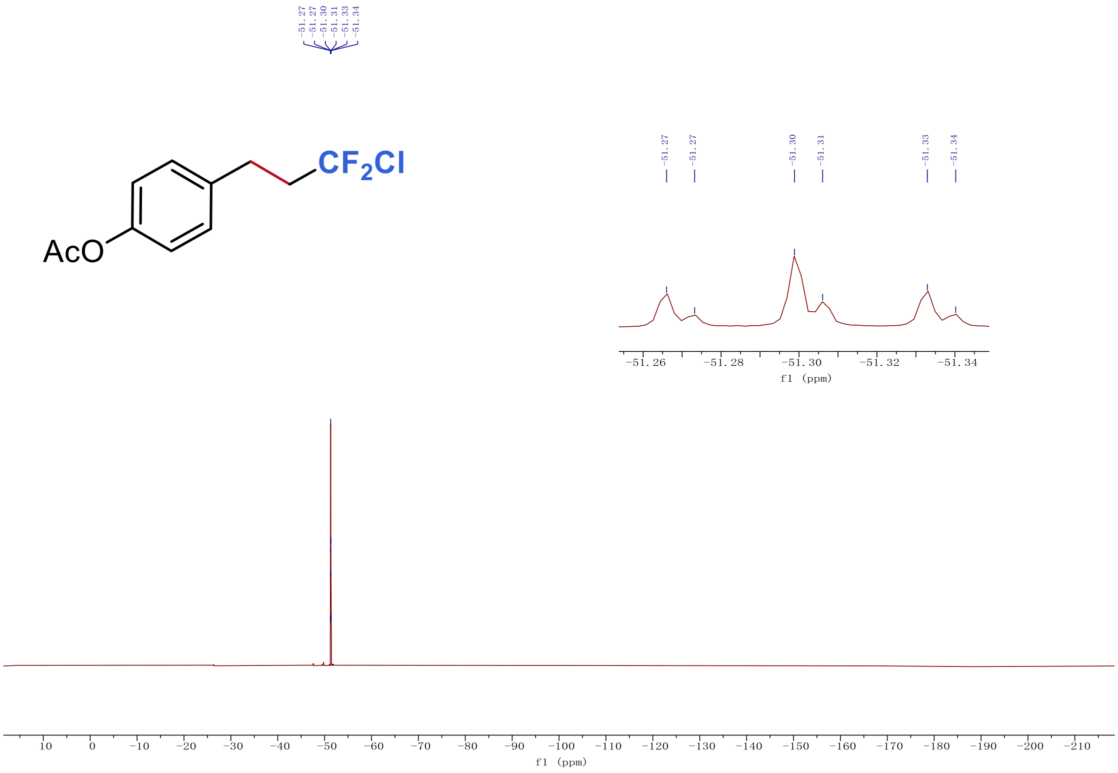

^{13}C NMR (126 MHz, $CDCl_3$) spectra for compound **1ai**

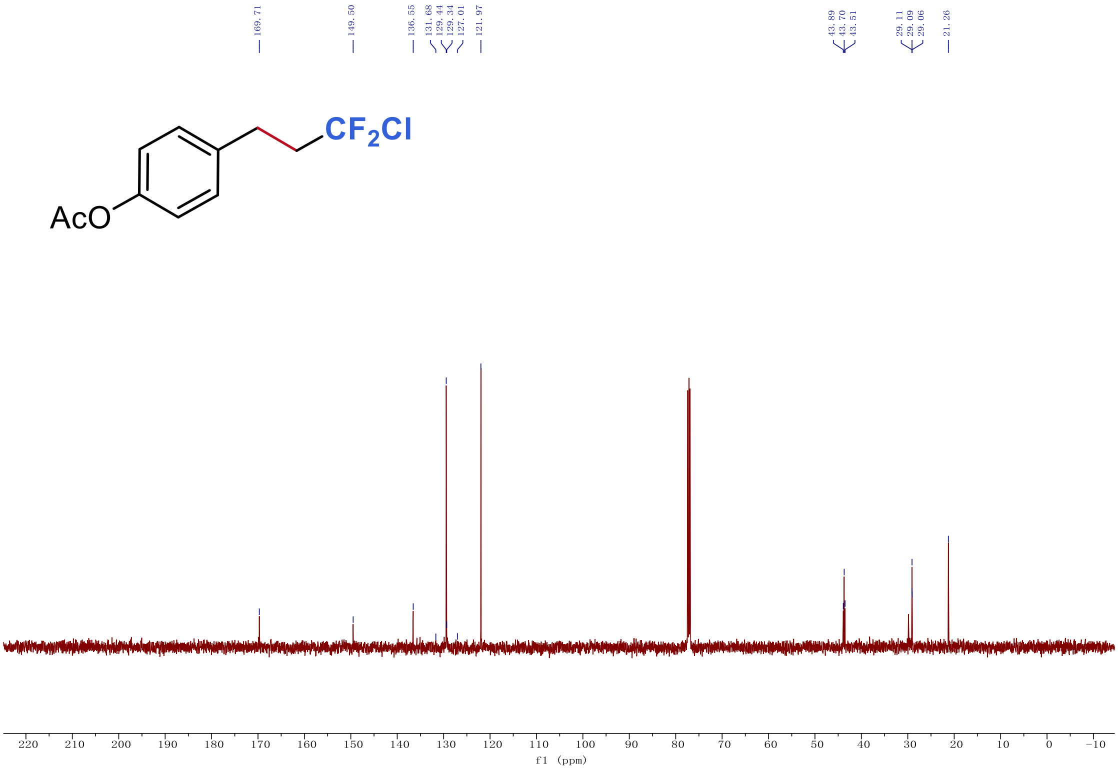

^{1}H NMR (400 MHz, $CDCl_3$) spectra for compound **1aj**

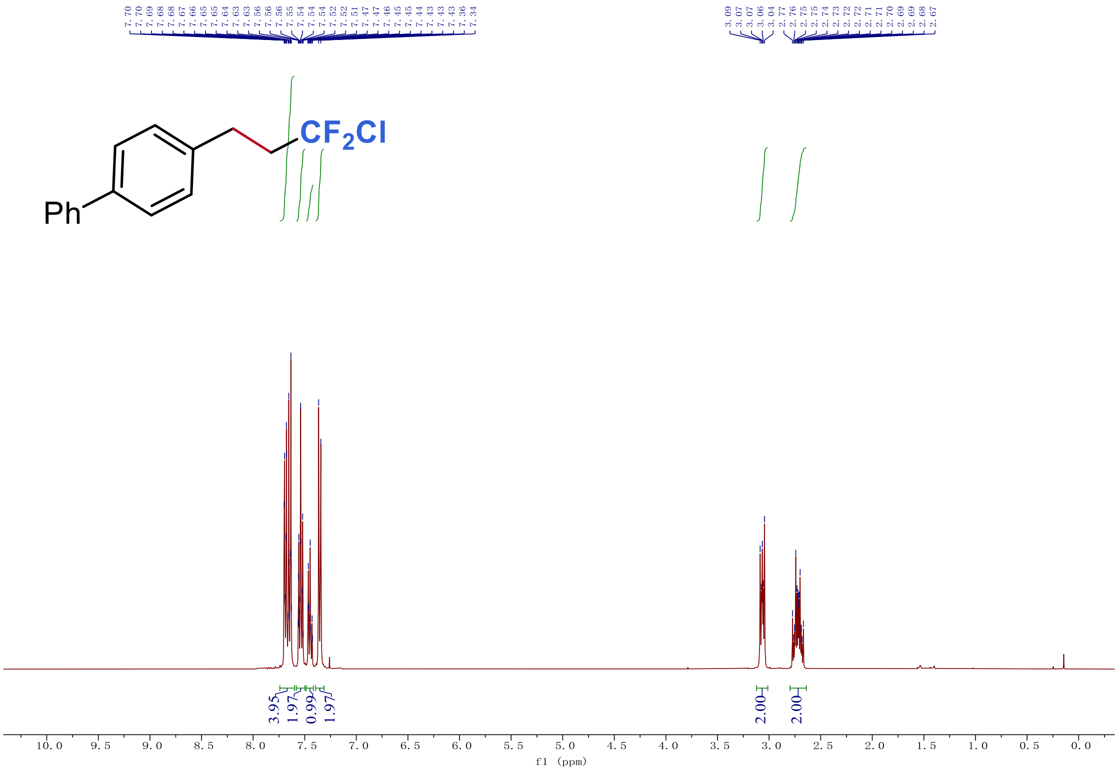

^{19}F NMR (377 MHz, $CDCl_3$) spectra for compound **1aj**

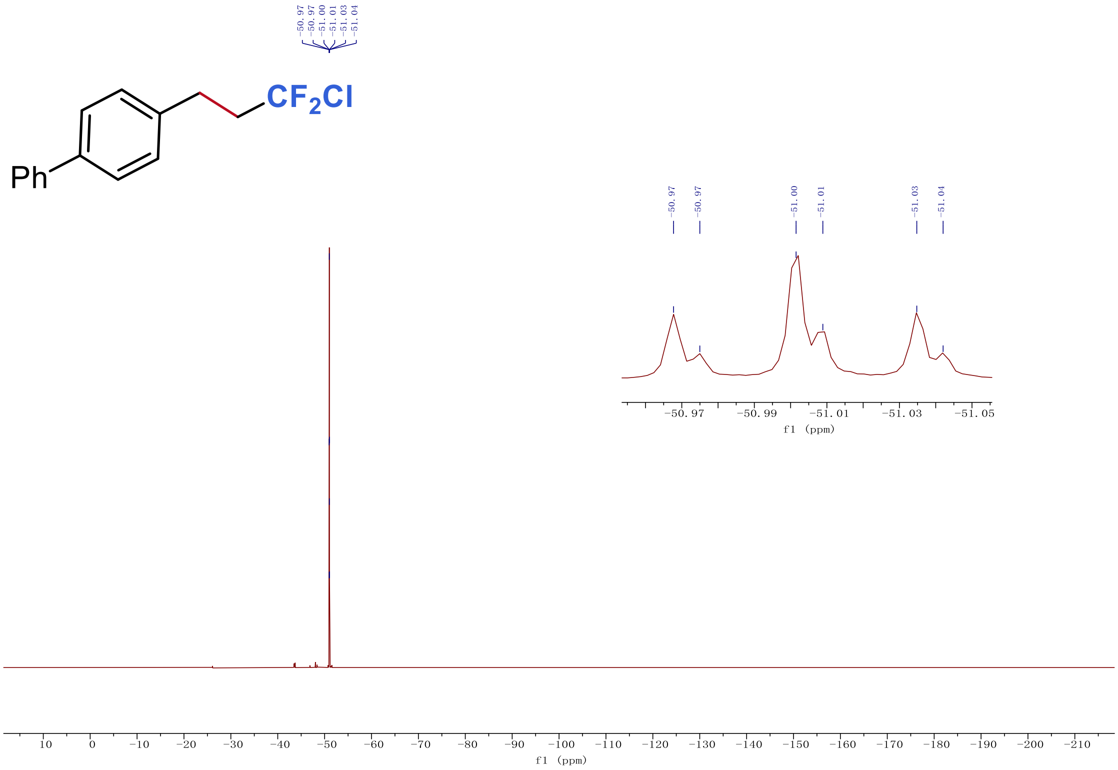

^{13}C NMR (126 MHz, $CDCl_3$) spectra for compound **1aj**

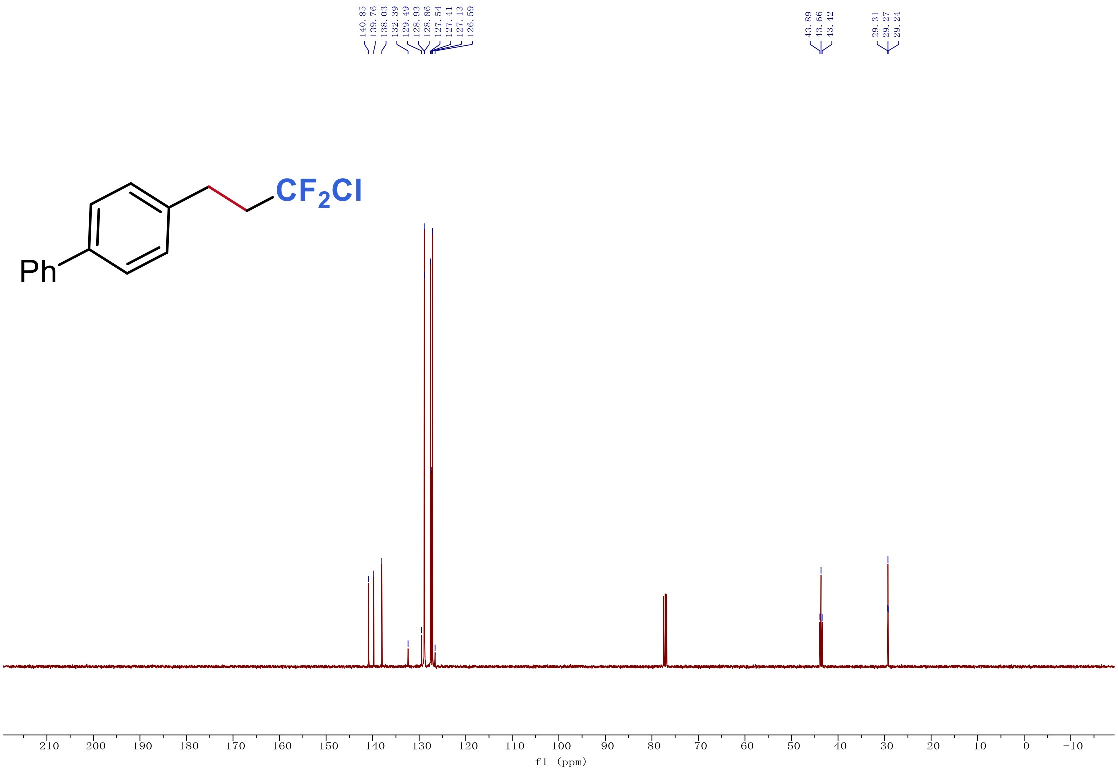

^{1}H NMR (400 MHz, $CDCl_3$) spectra for compound **1ak**

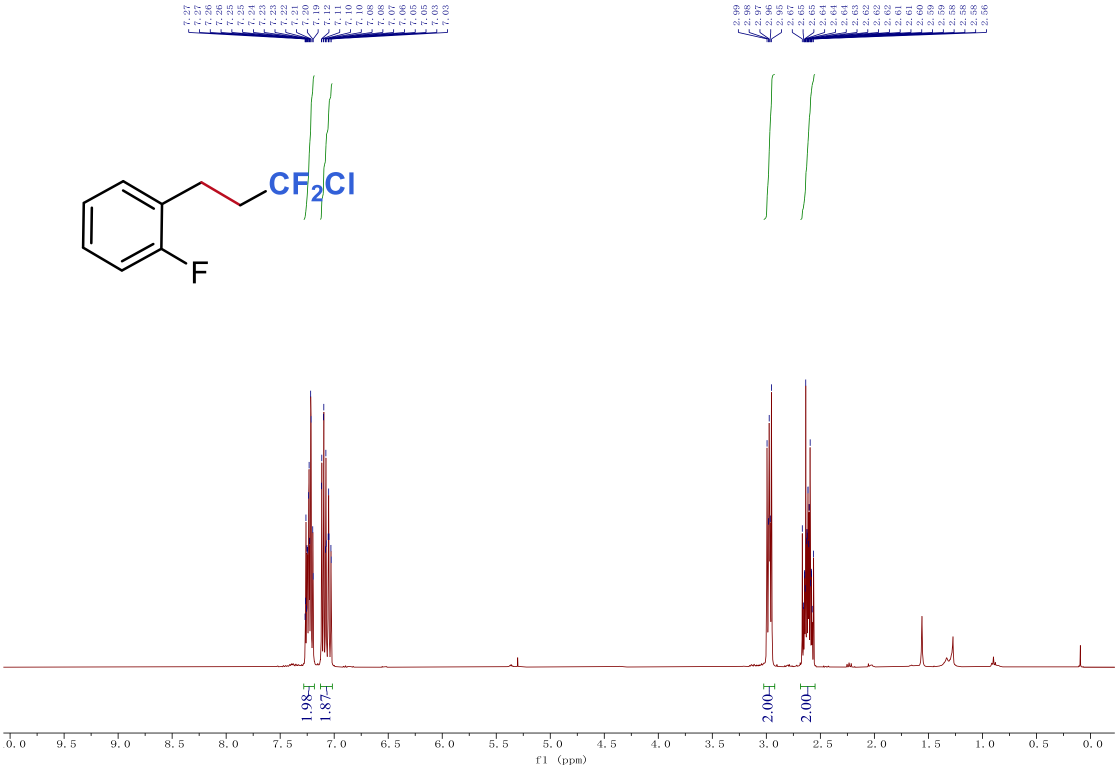

^{19}F NMR (377 MHz, $CDCl_3$) spectra for compound **1ak**

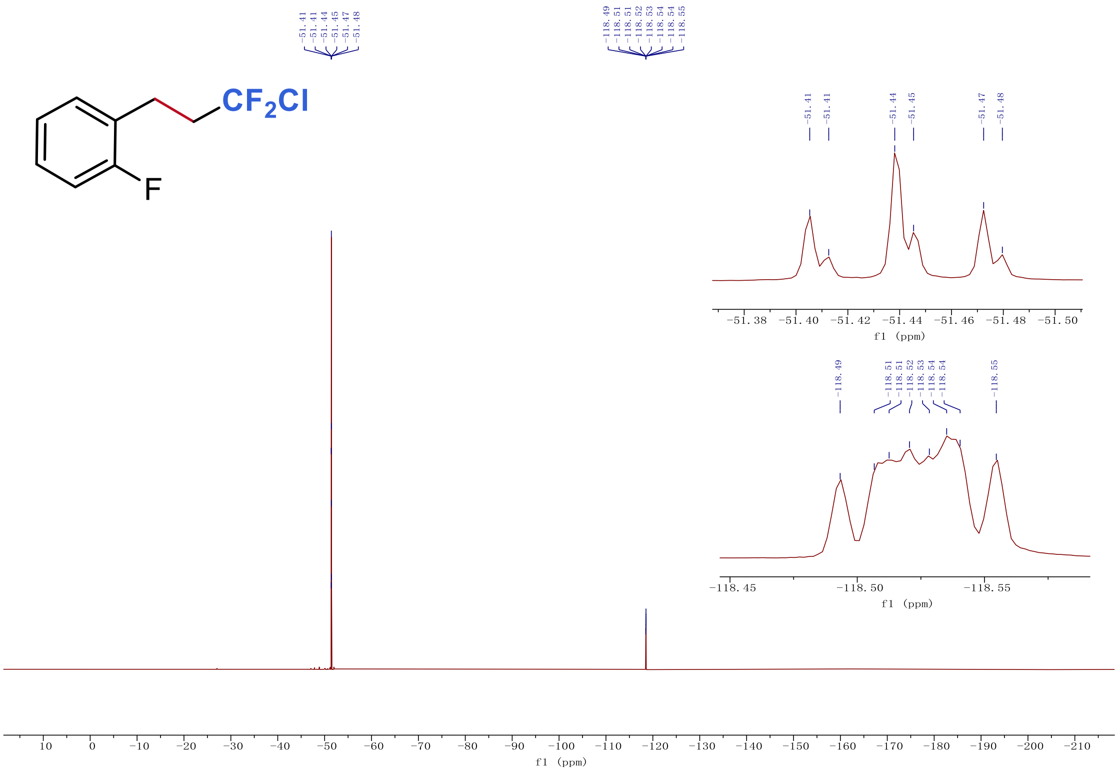

^{13}C NMR (126 MHz, $CDCl_3$) spectra for compound **1ak**

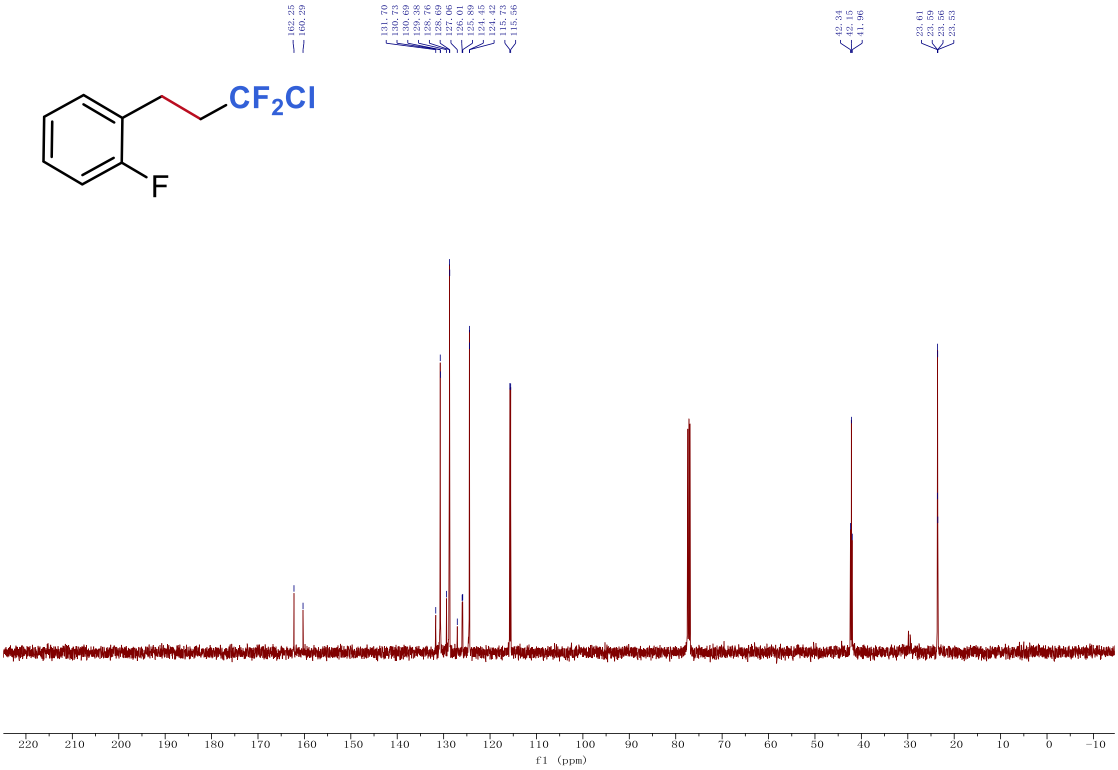

^{1}H NMR (400 MHz, $CDCl_3$) spectra for compound **1al**

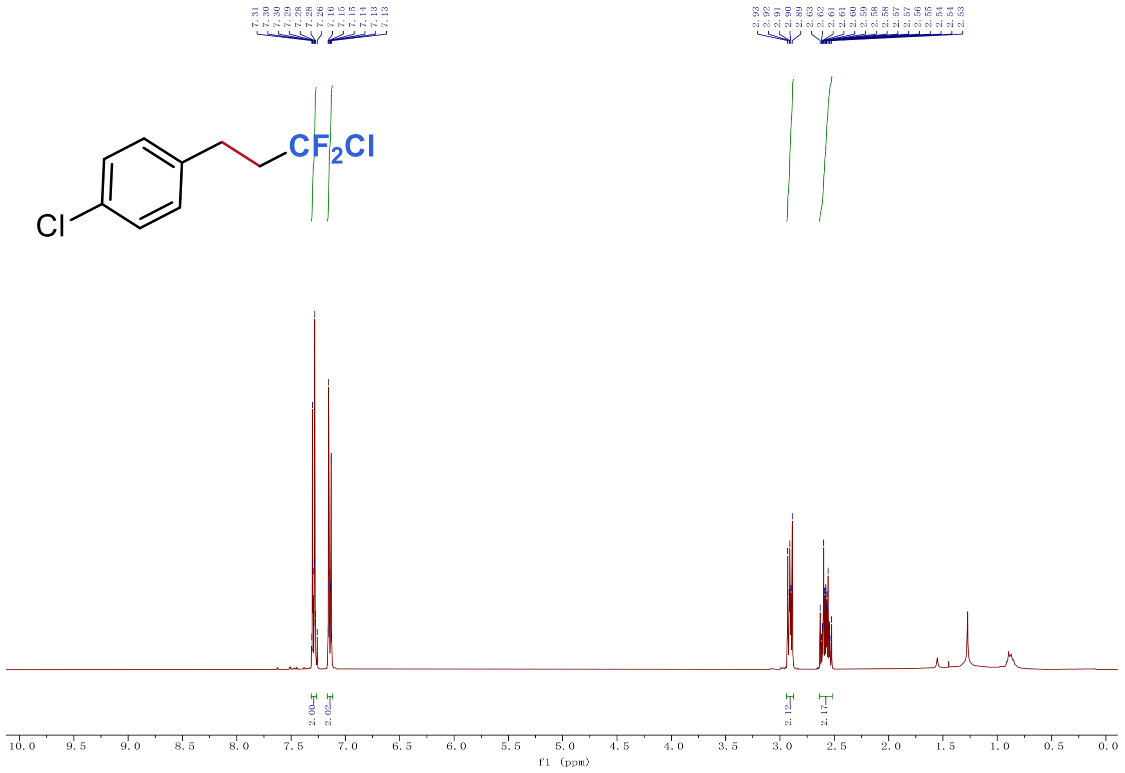

^{19}F NMR (377 MHz, $CDCl_3$) spectra for compound **1al**

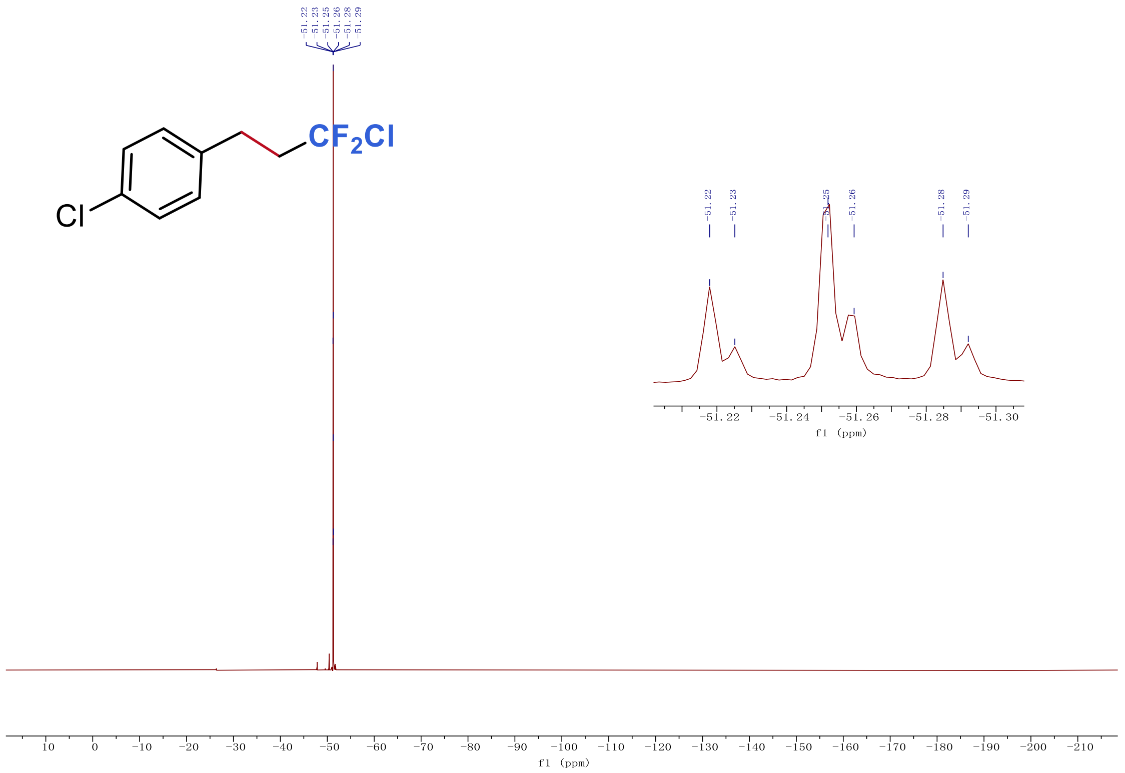

^{13}C NMR (126 MHz, $CDCl_3$) spectra for compound **1al**

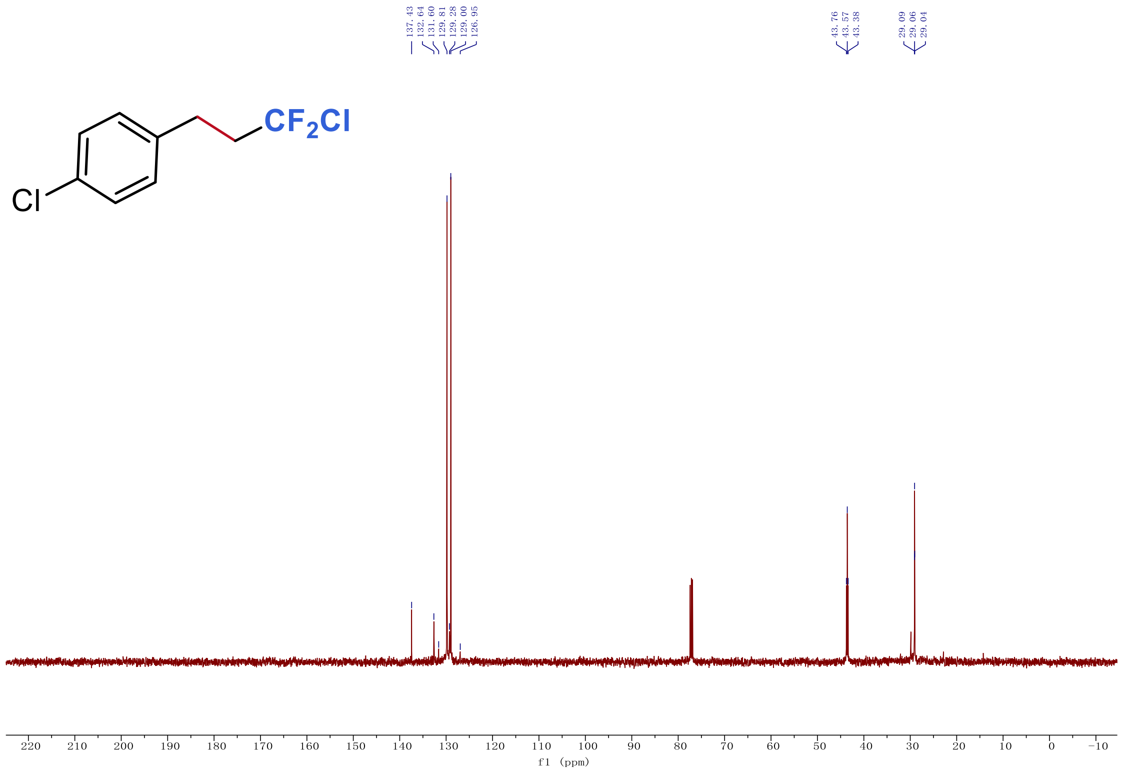

^{1}H NMR (400 MHz, $CDCl_3$) spectra for compound **1am**

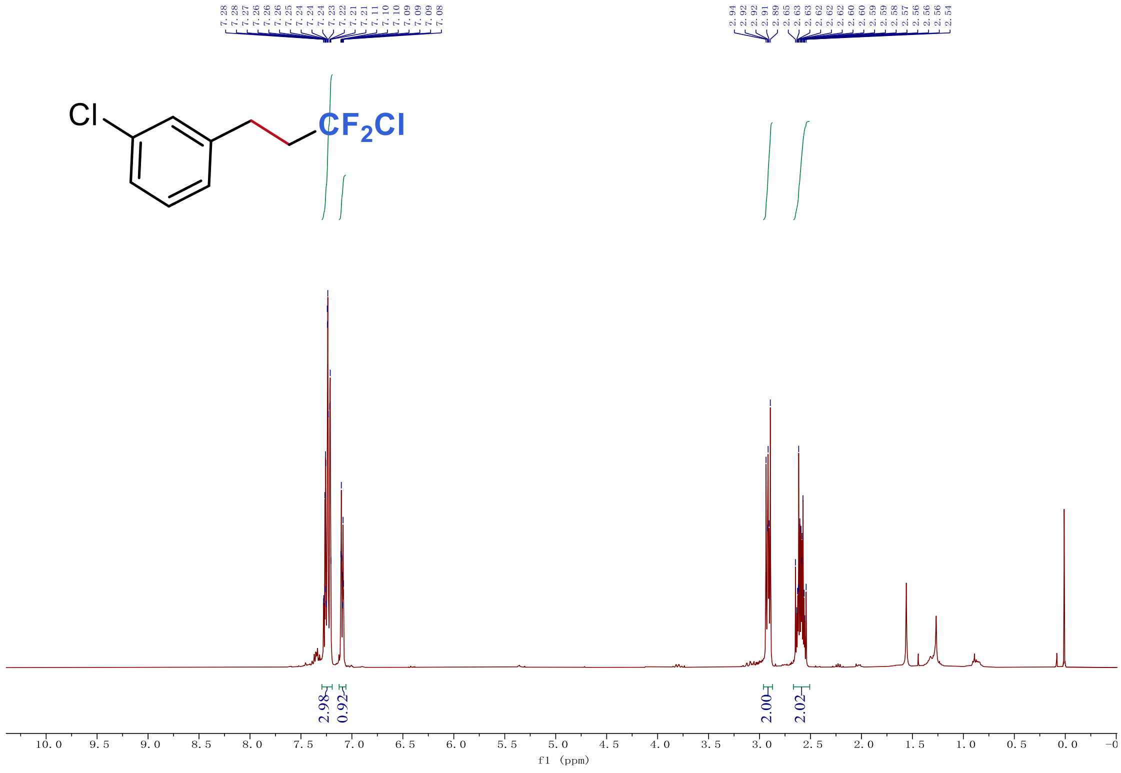

^{19}F NMR (377 MHz, $CDCl_3$) spectra for compound **1am**

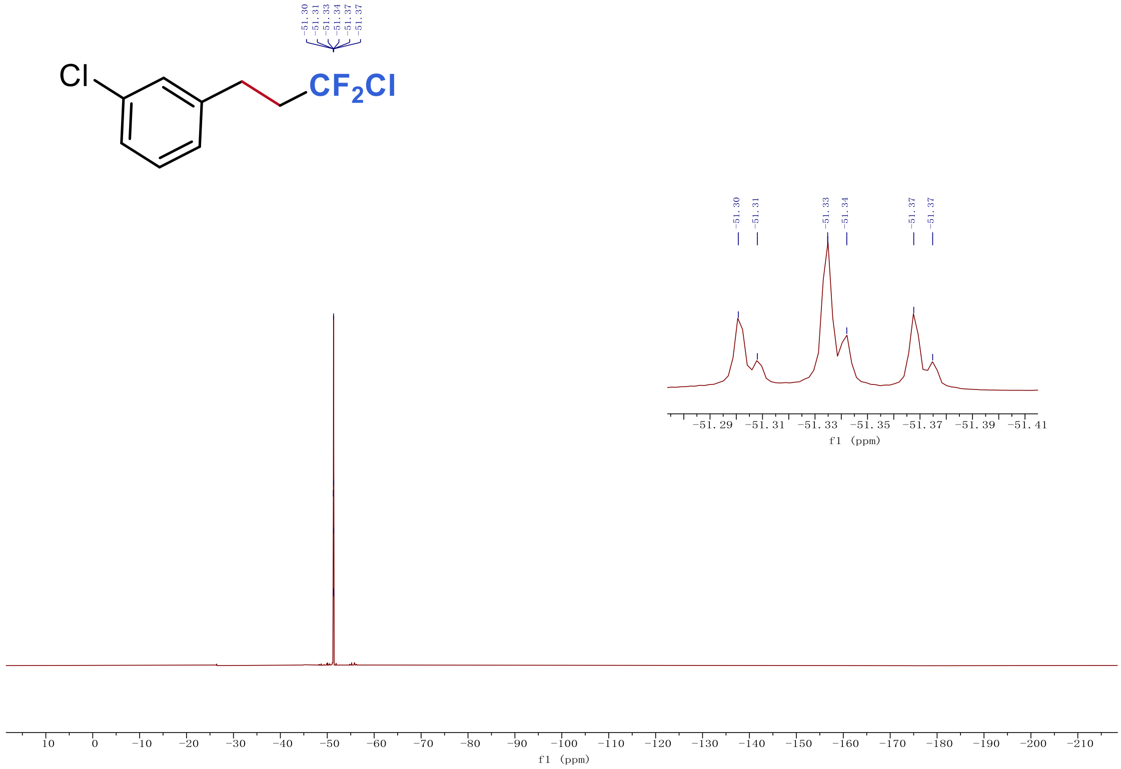

^{13}C NMR (126 MHz, $CDCl_3$) spectra for compound **1am**

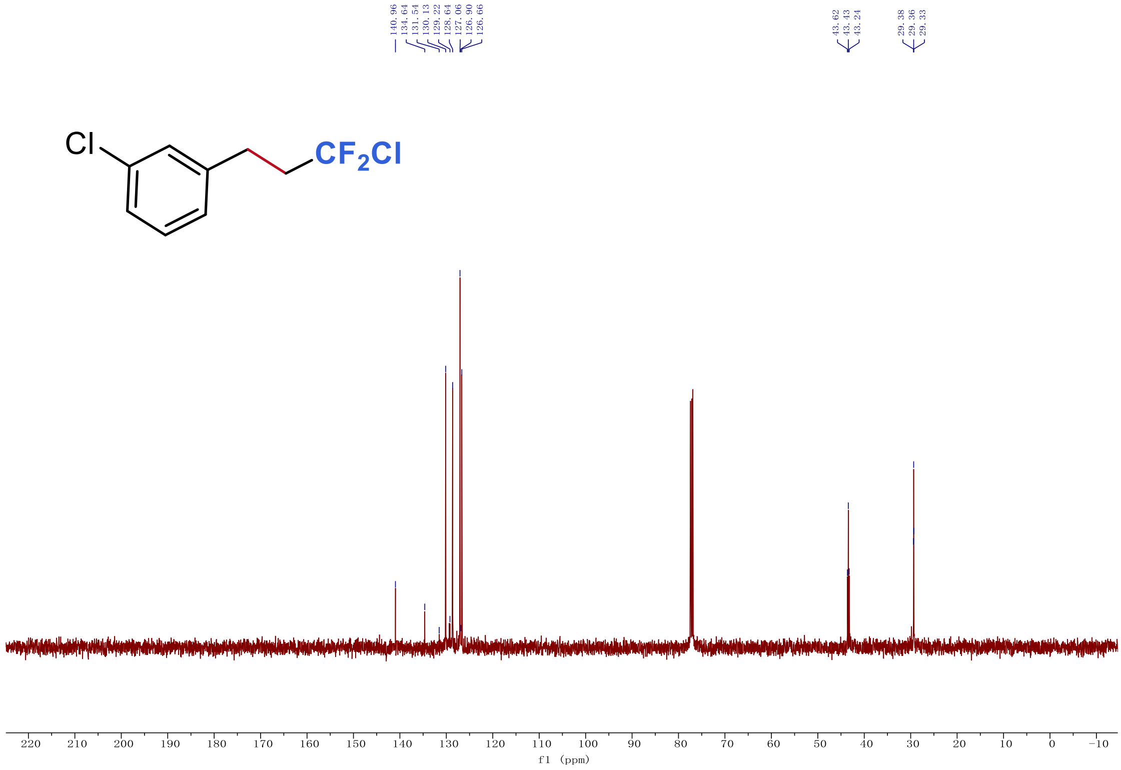

^{1}**H NMR** (400 MHz, $CDCl_3$) spectra for compound **1an**

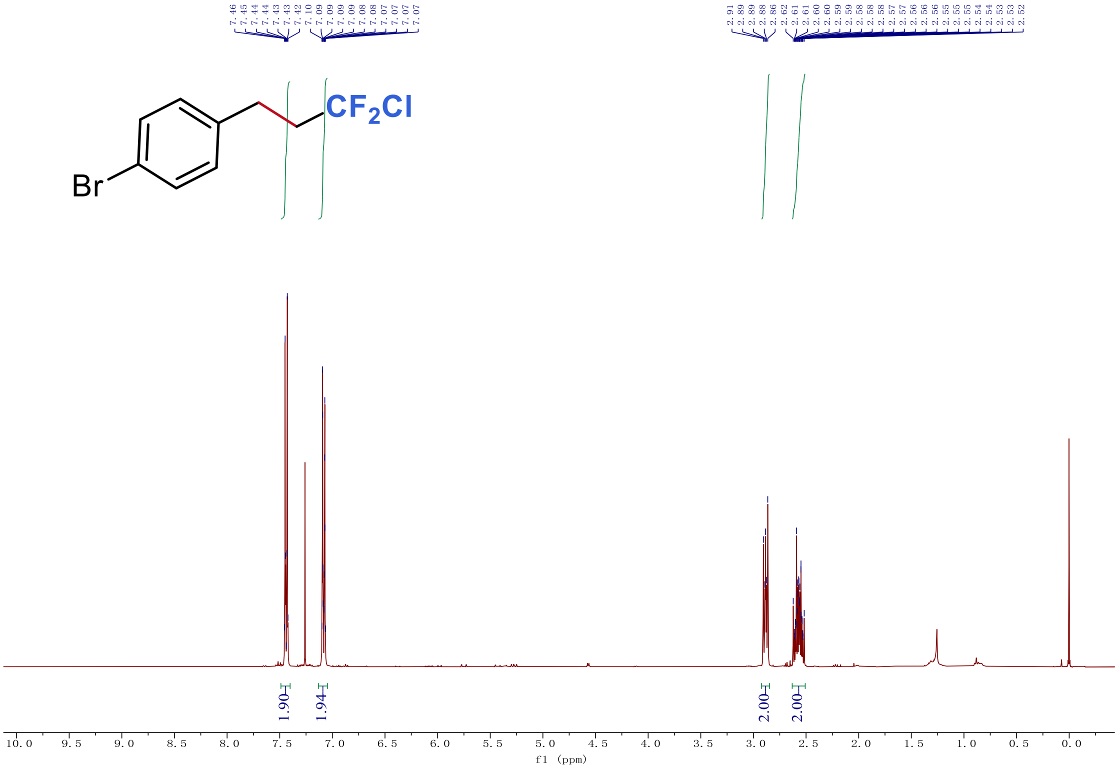

^{19}**F NMR** (377 MHz, $CDCl_3$) spectra for compound **1an**

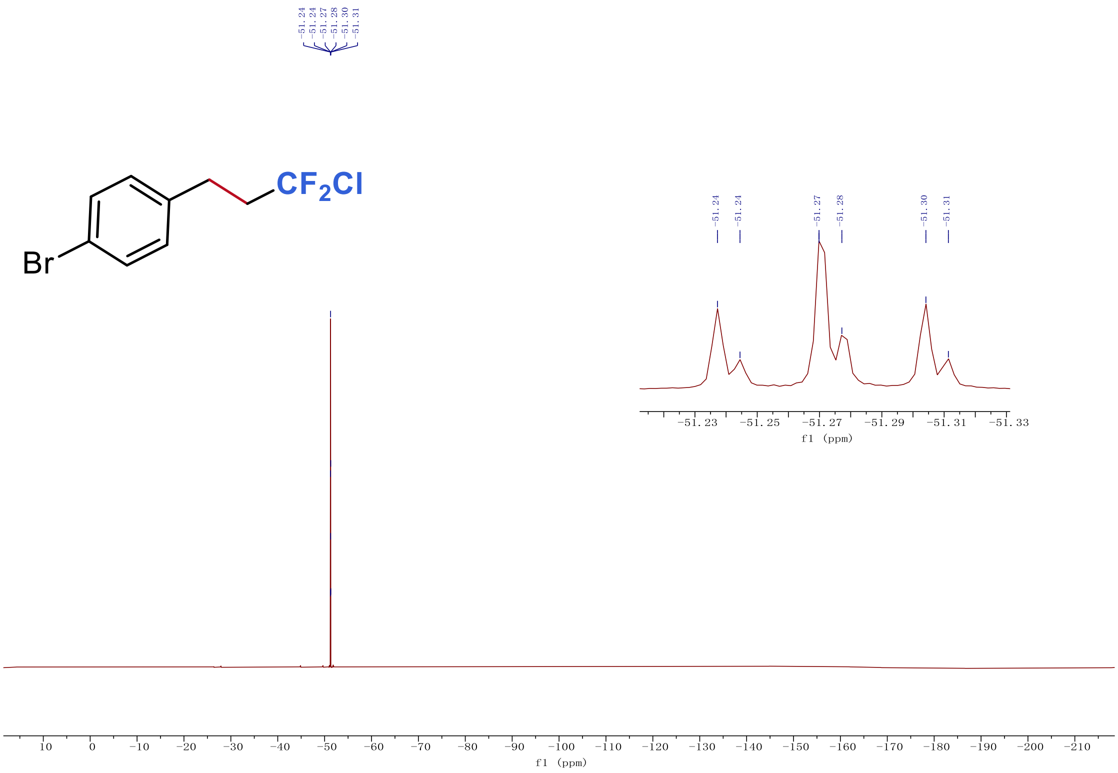

^{13}C NMR (126 MHz, $CDCl_3$) spectra for compound **1an**

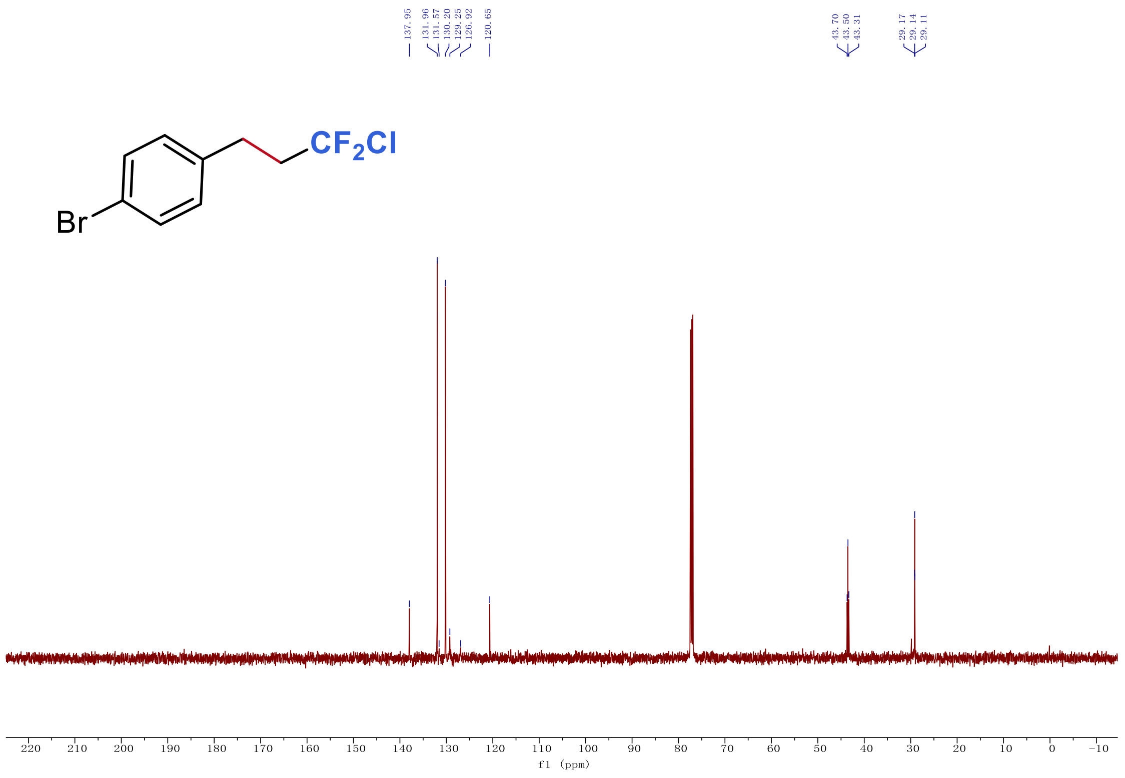

^{1}H NMR (400 MHz, $CDCl_3$) spectra for compound **1ao**

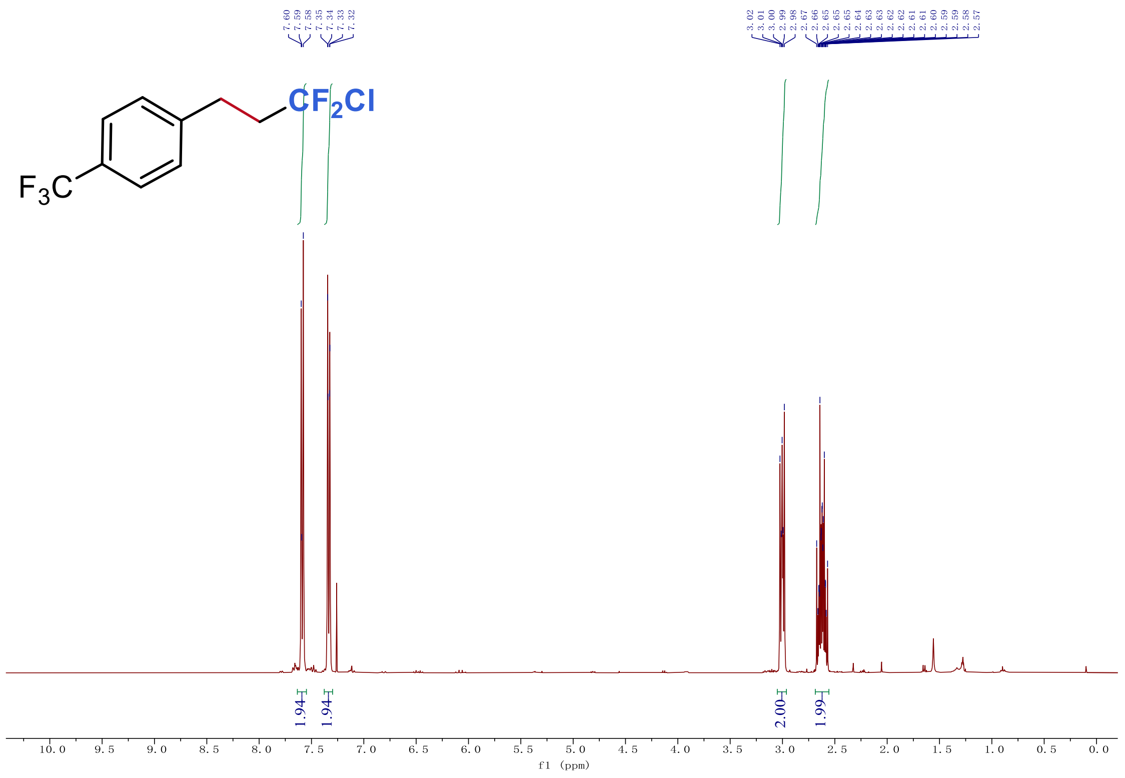

^{19}F NMR (377 MHz, $CDCl_3$) spectra for compound **1ao**

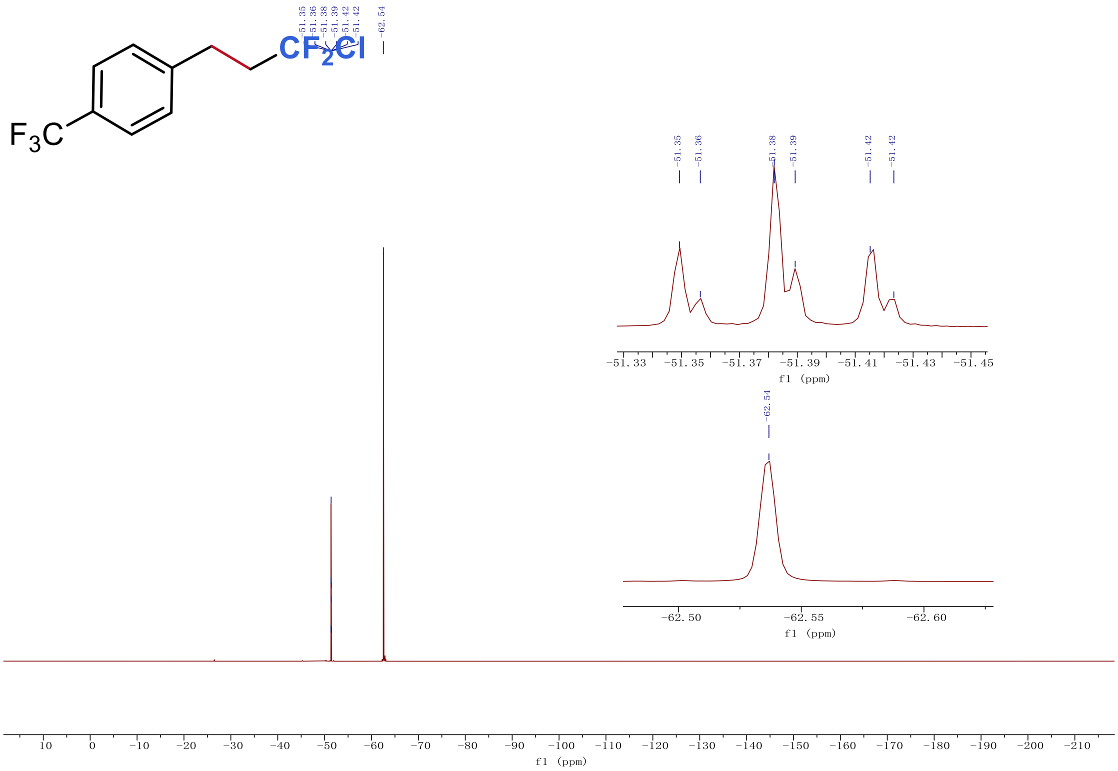

^{13}C NMR (126 MHz, $CDCl_3$) spectra for compound **1ao**

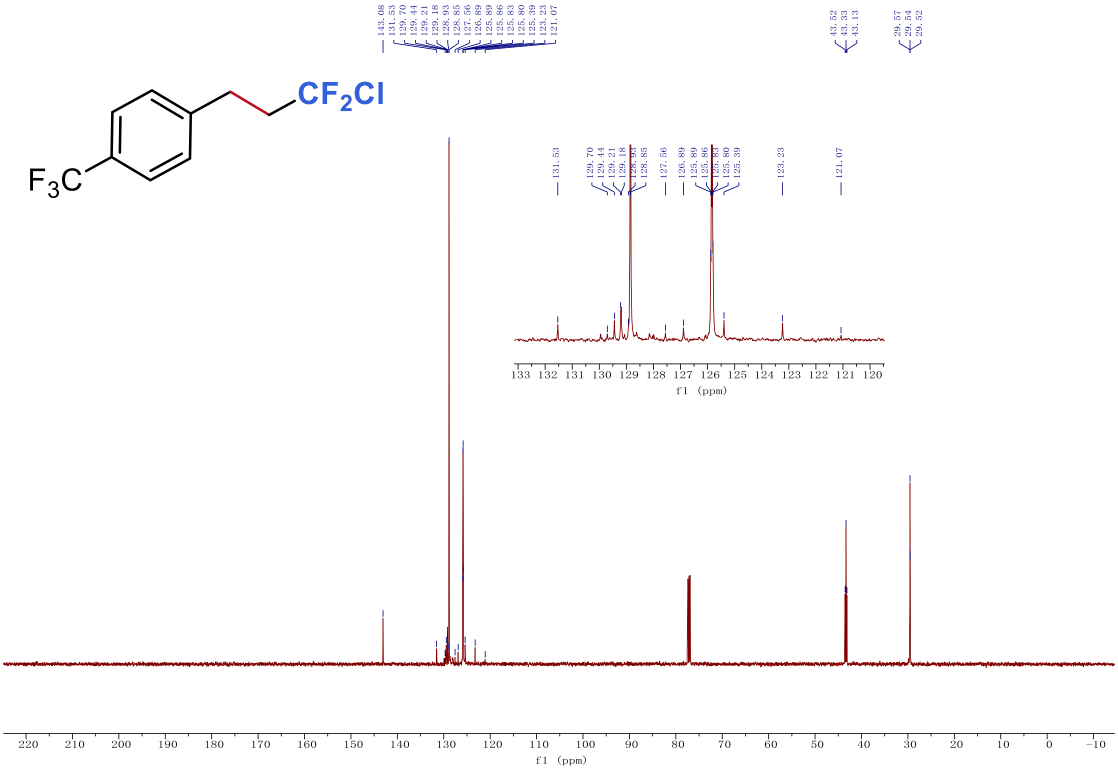

^{1}H NMR (400 MHz, $CDCl_3$) spectra for compound **1ap**

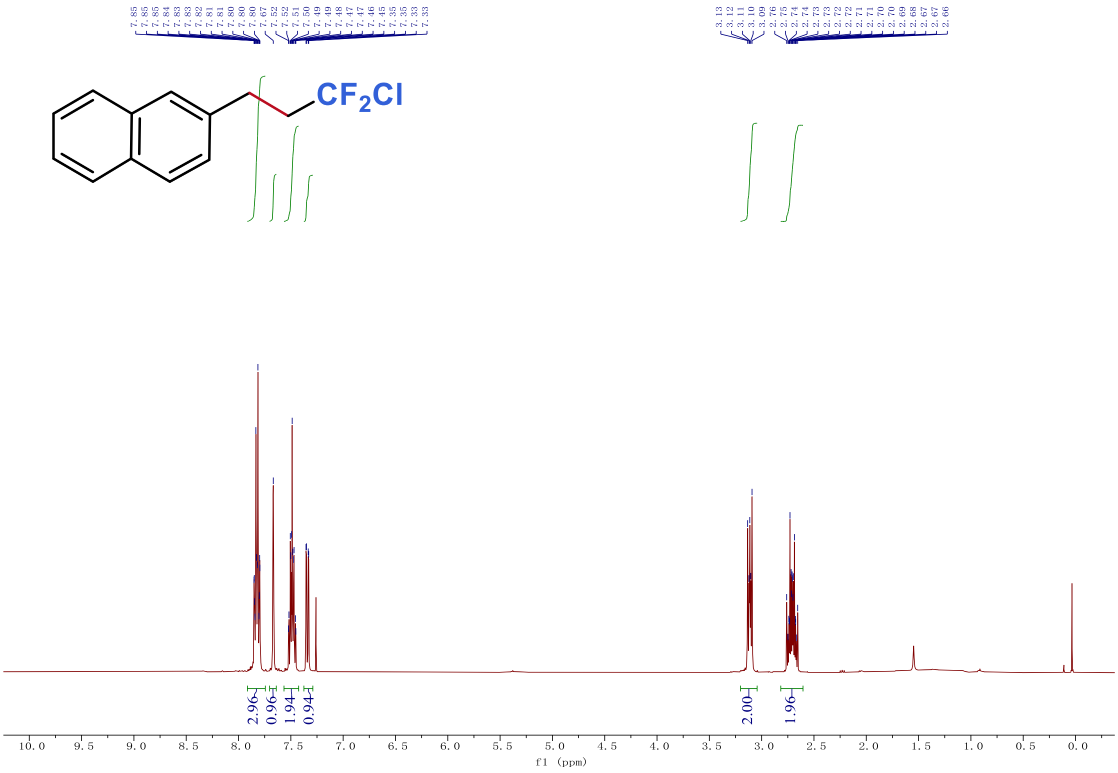

^{19}F NMR (377 MHz, $CDCl_3$) spectra for compound **1ap**

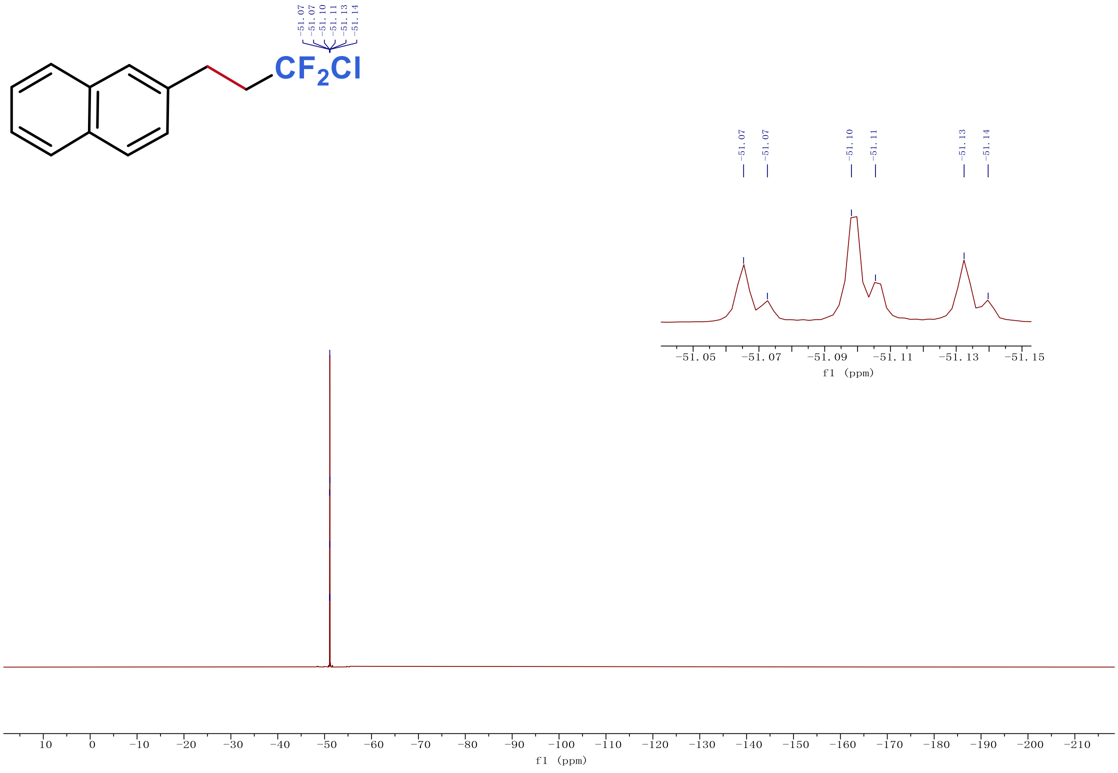

^{13}C NMR (126 MHz, $CDCl_3$) spectra for compound **1ap**

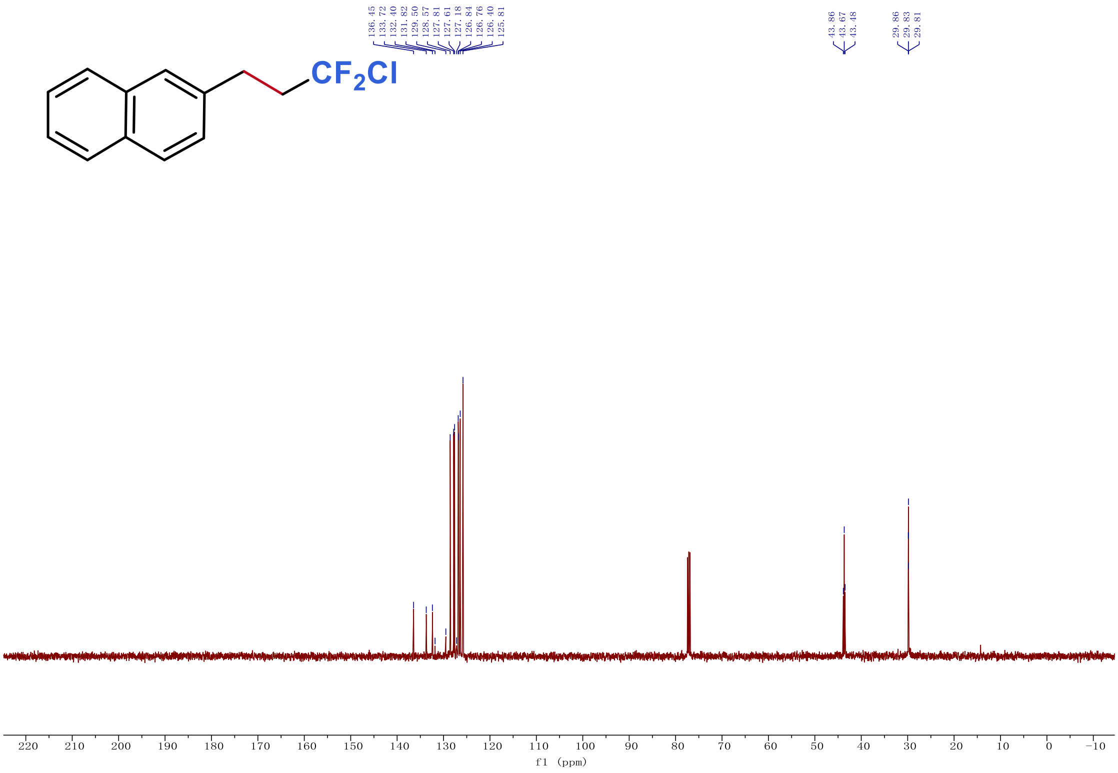

^{1}H NMR (400 MHz, $CDCl_3$) spectra for compound **1aq**

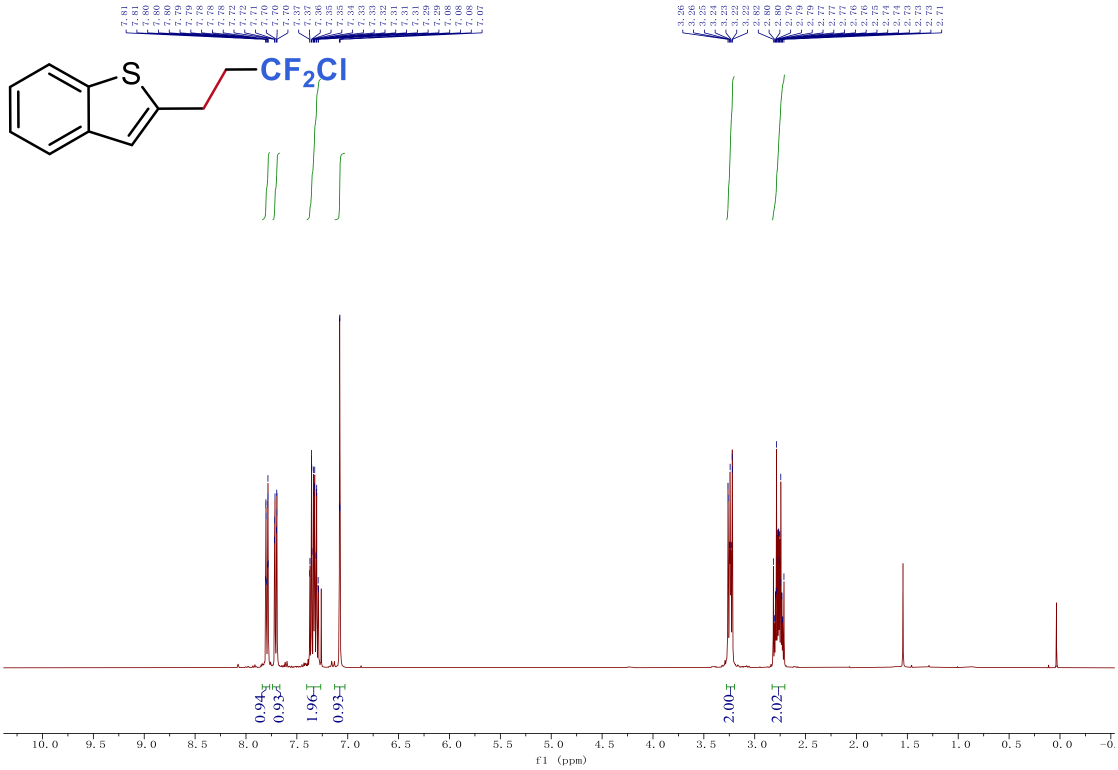

^{19}F NMR (377 MHz, $CDCl_3$) spectra for compound **1aq**

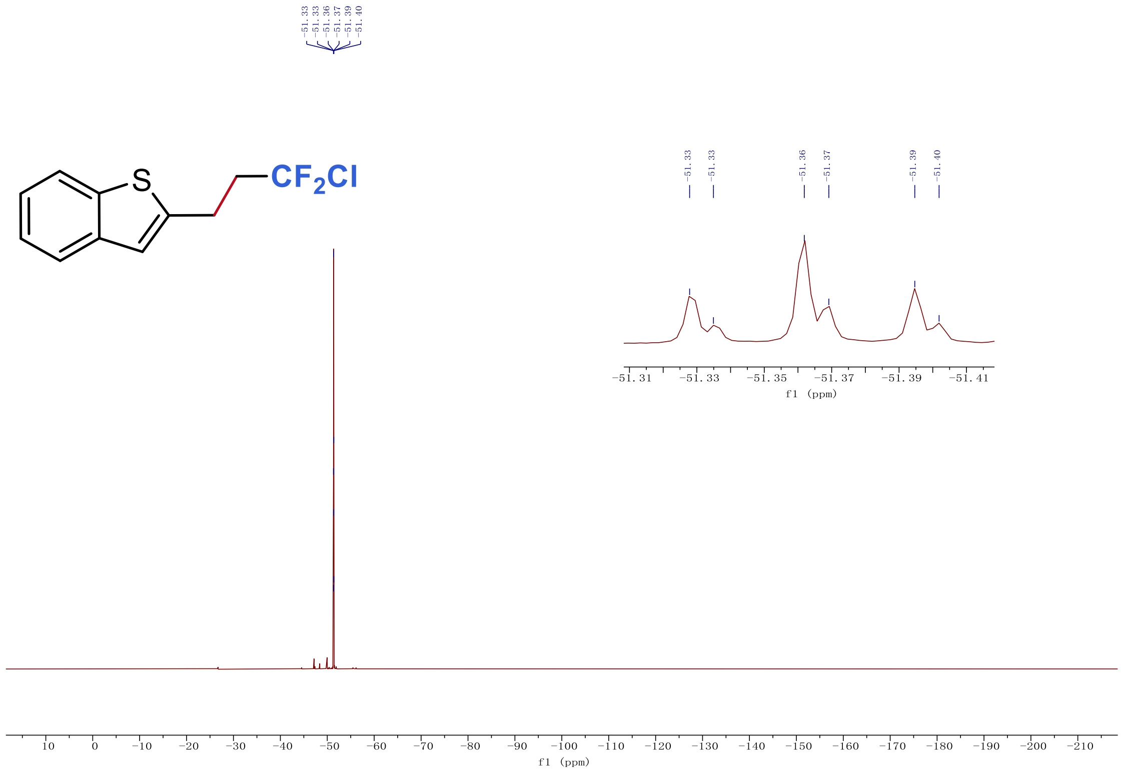

^{13}C NMR (126 MHz, $CDCl_3$) spectra for compound **1aq**

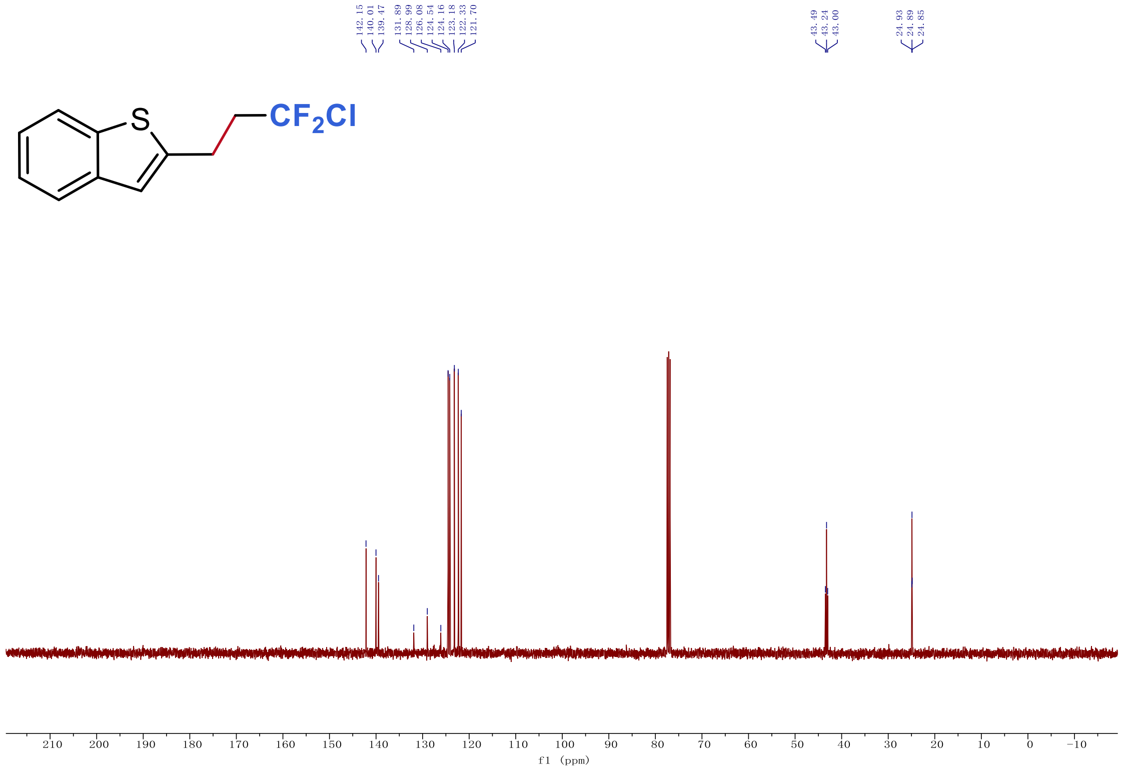

^{1}H NMR (400 MHz, $CDCl_3$) spectra for compound **1ar**

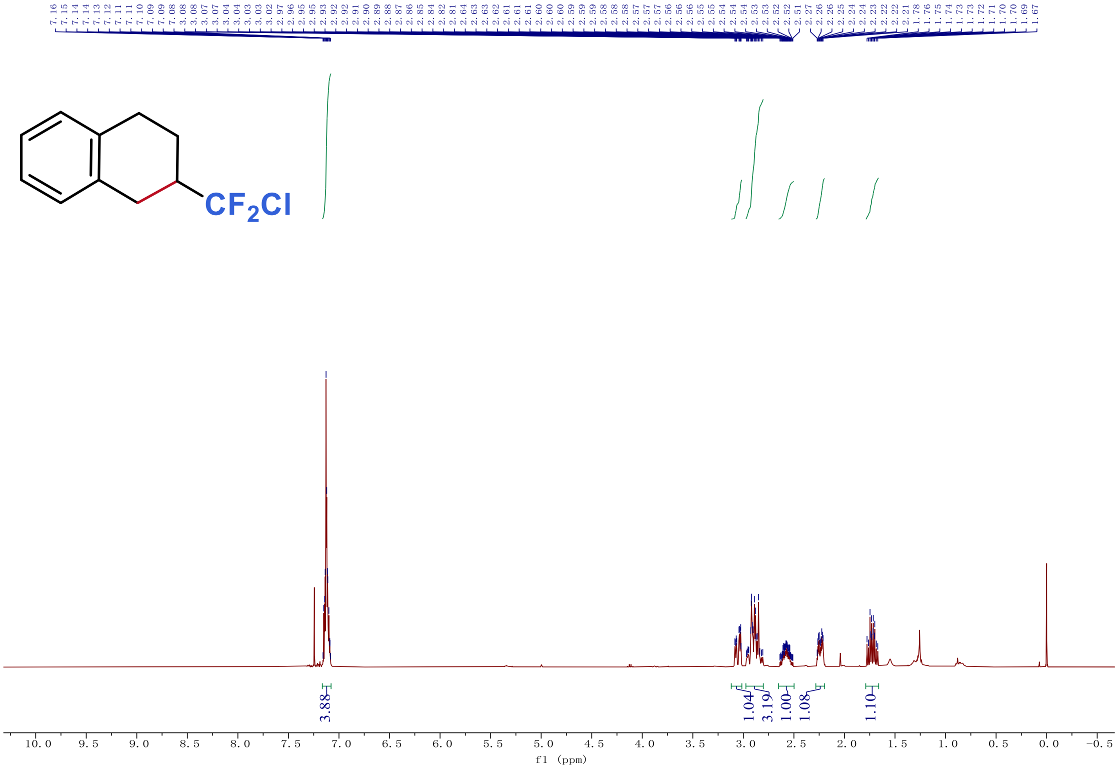

^{19}F NMR (377 MHz, $CDCl_3$) spectra for compound **1ar**

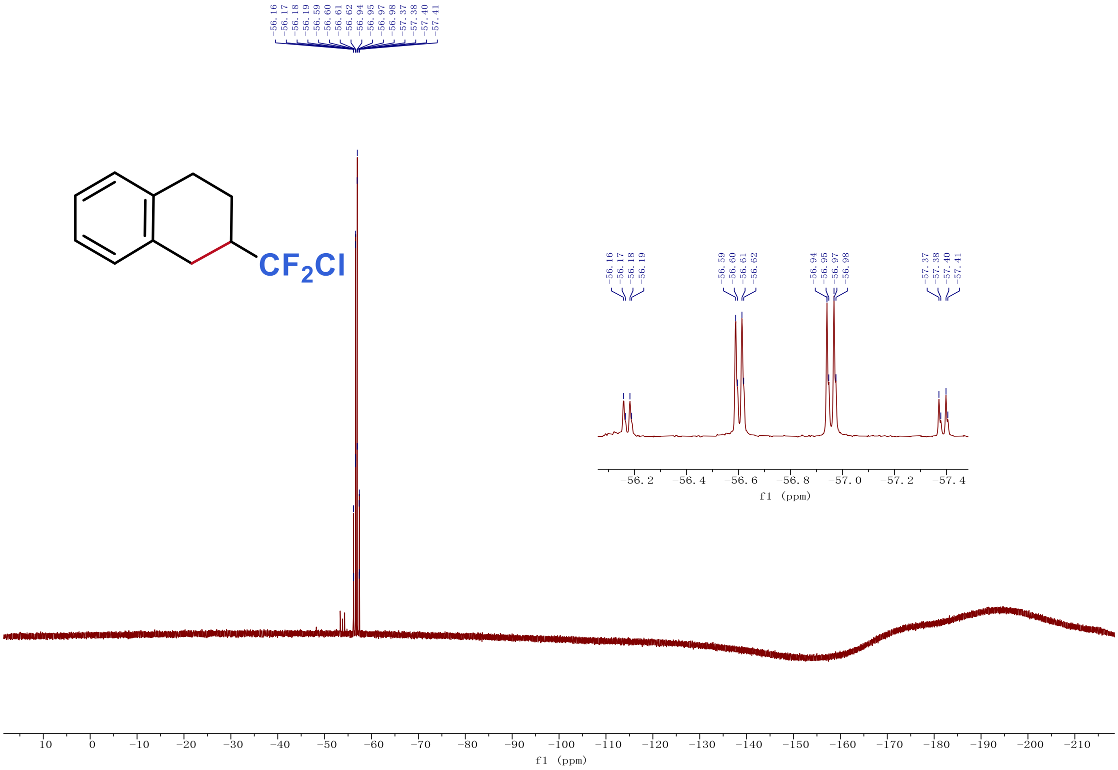

^{13}C NMR (126 MHz, $CDCl_3$) spectra for compound **1ar**

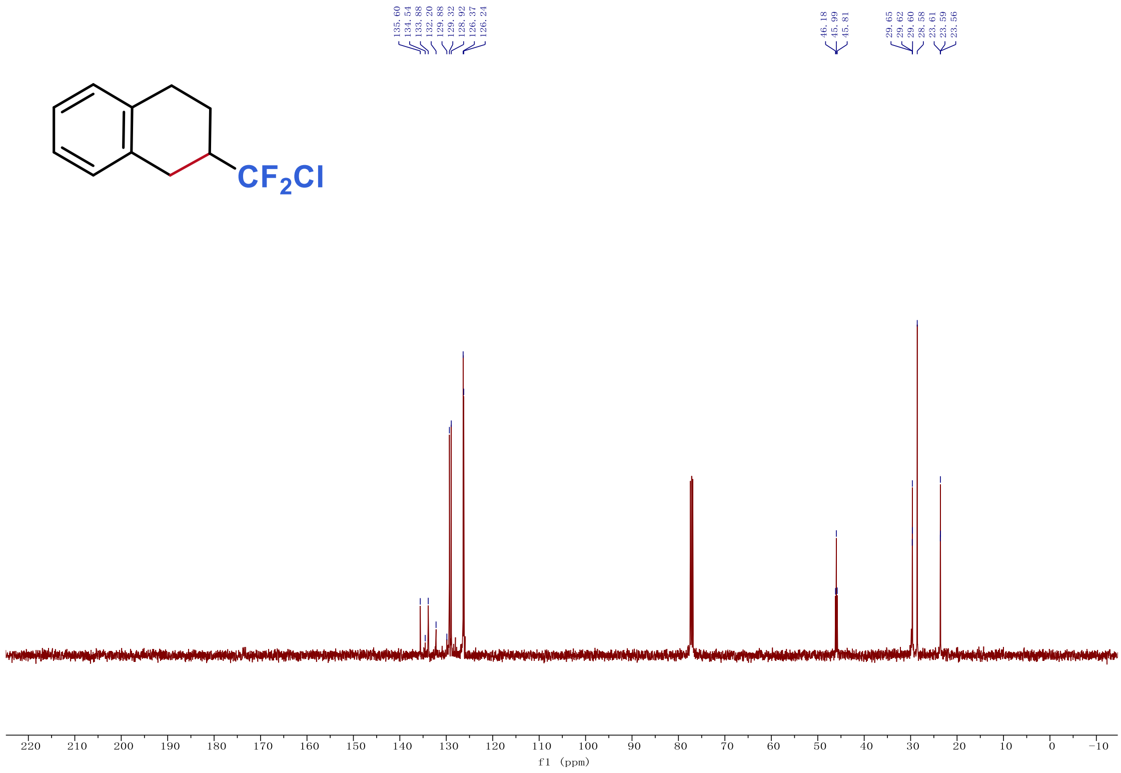

^{1}H NMR (400 MHz, $CDCl_3$) spectra for compound **1as**

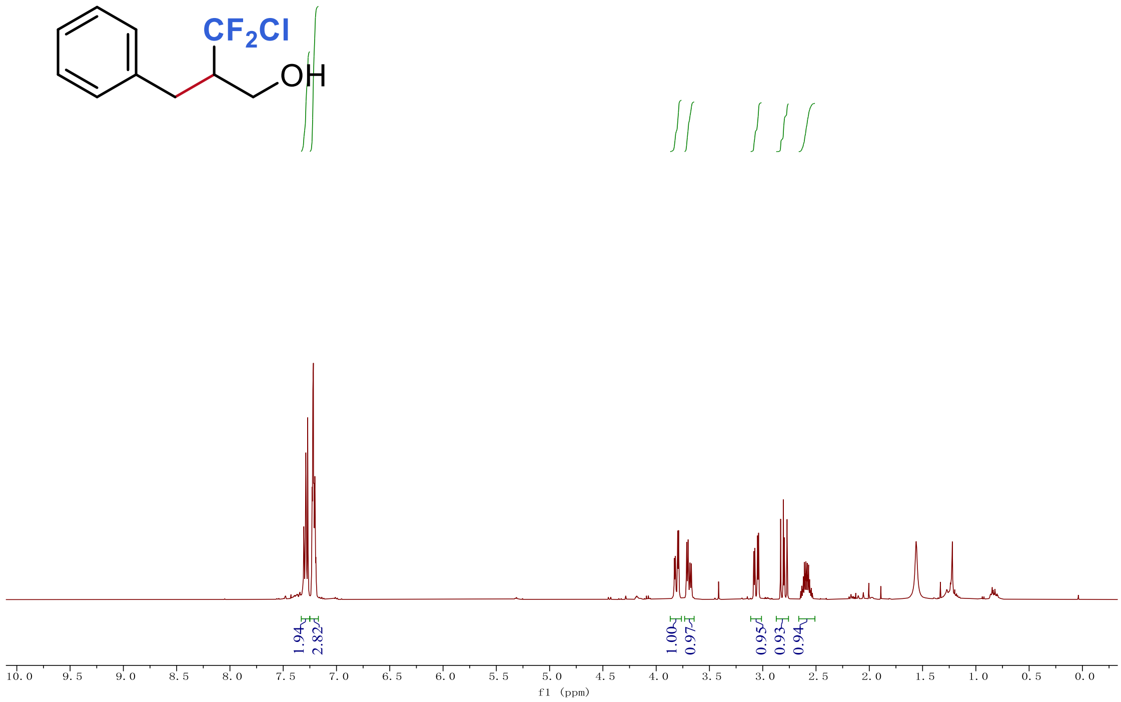

^{19}F NMR (377 MHz, $CDCl_3$) spectra for compound **1as**

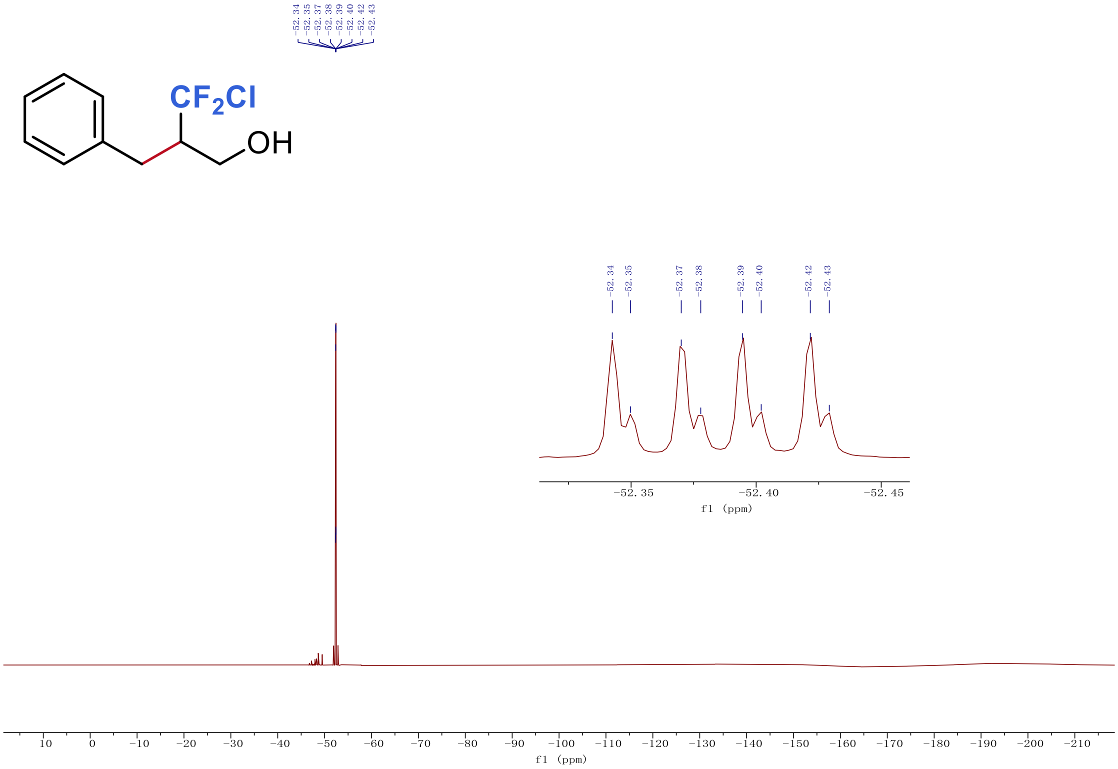

^{13}C NMR (126 MHz, $CDCl_3$) spectra for compound **1as**

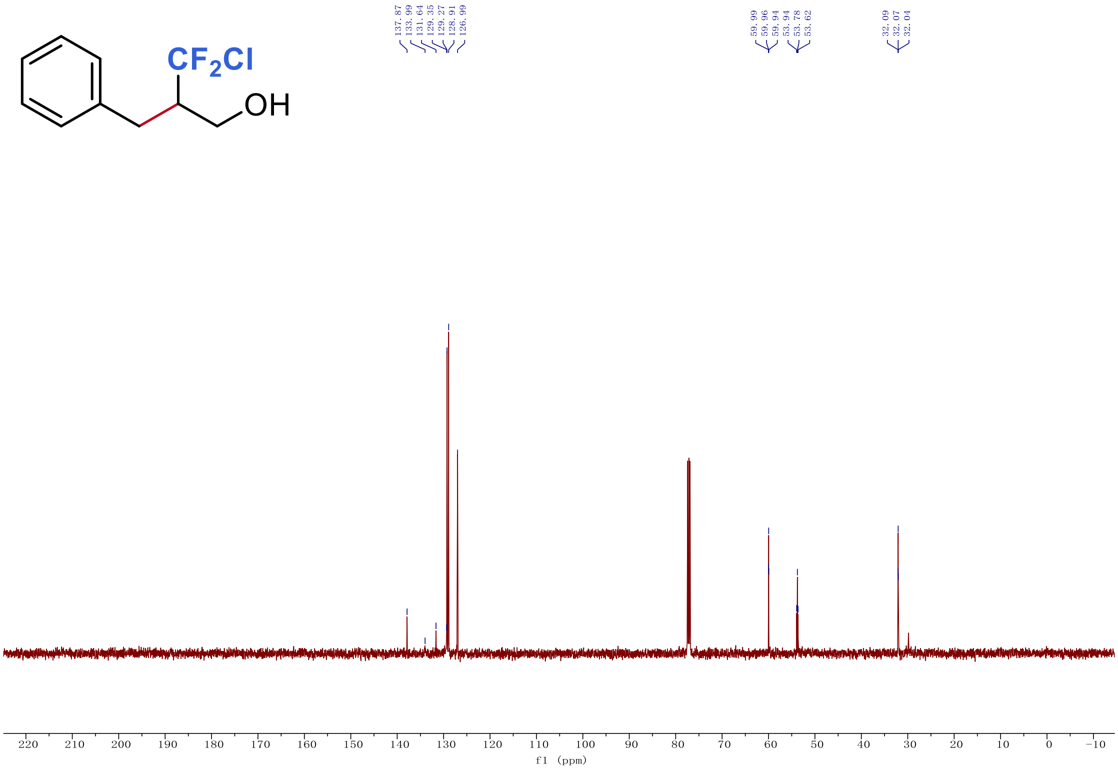

^{1}H NMR (400 MHz, $CDCl_3$) spectra for compound **1at**

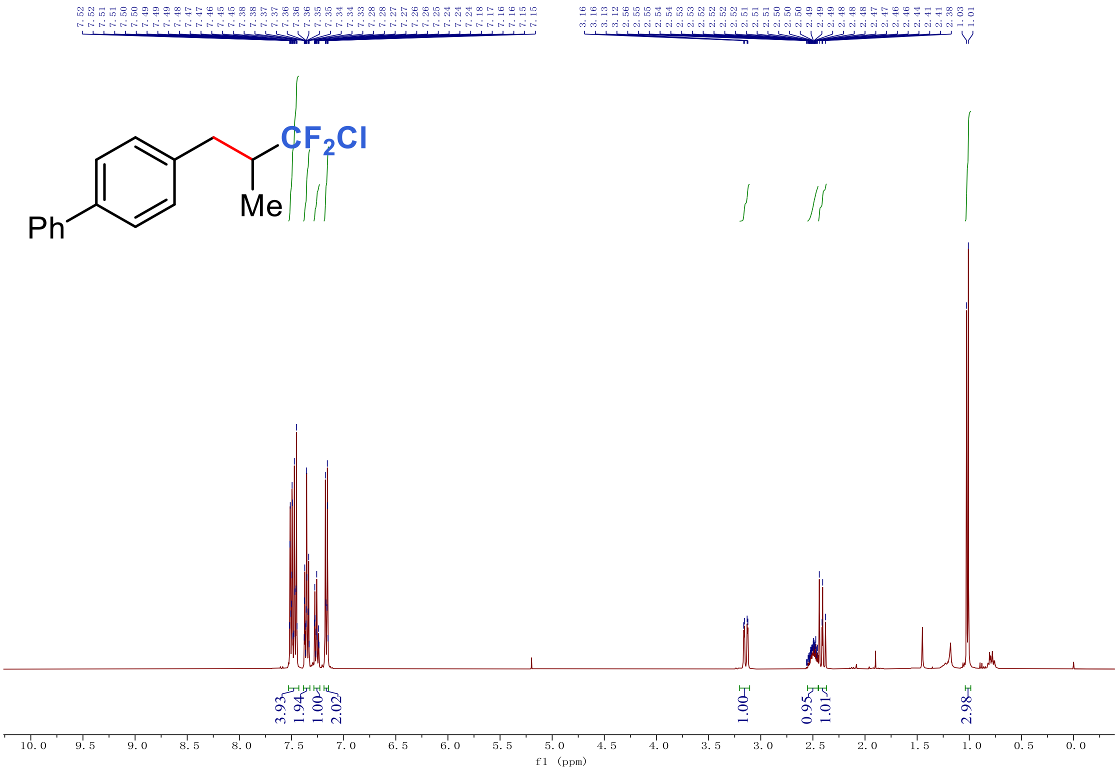

^{19}F NMR (377 MHz, $CDCl_3$) spectra for compound **1at**

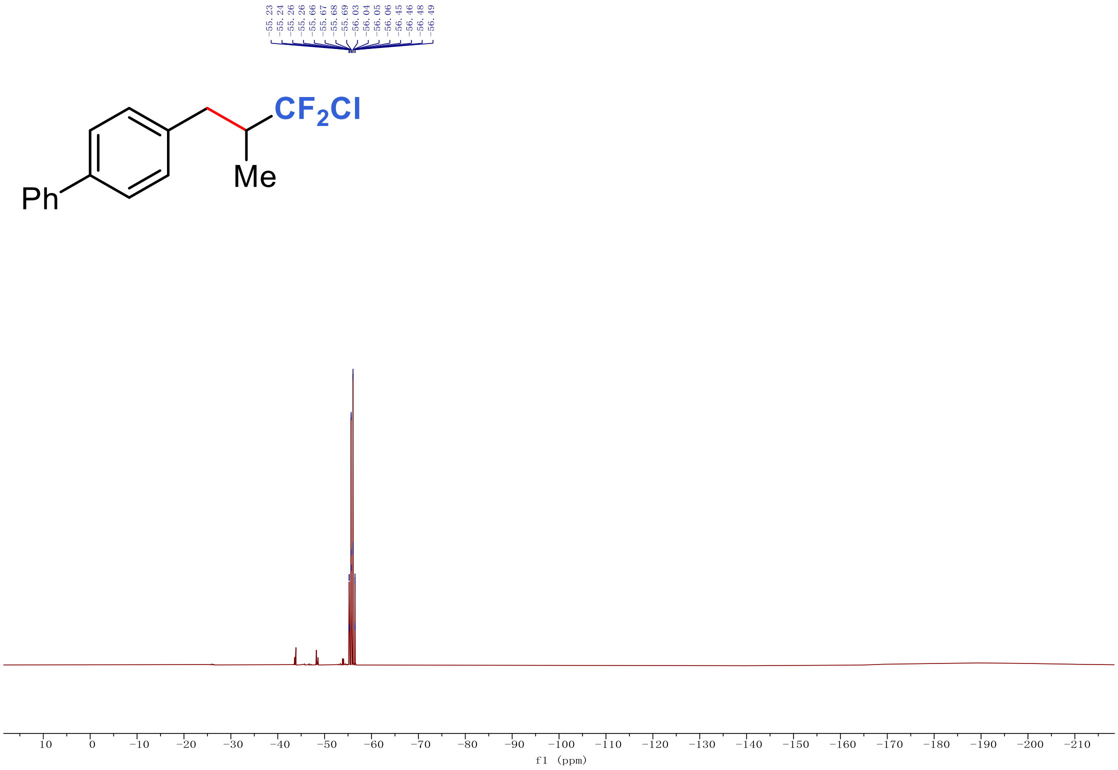

^{13}C NMR (126 MHz, $CDCl_3$) spectra for compound **1at**

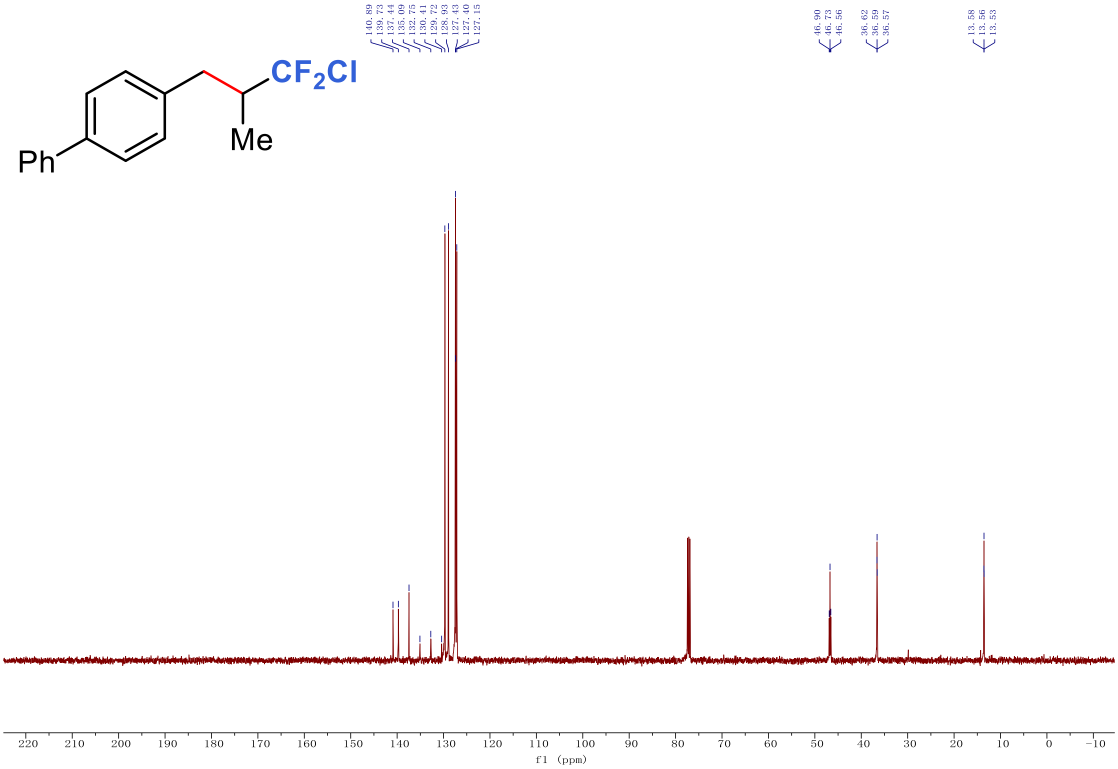

^{1}H NMR (400 MHz, $CDCl_3$) spectra for compound **1au**

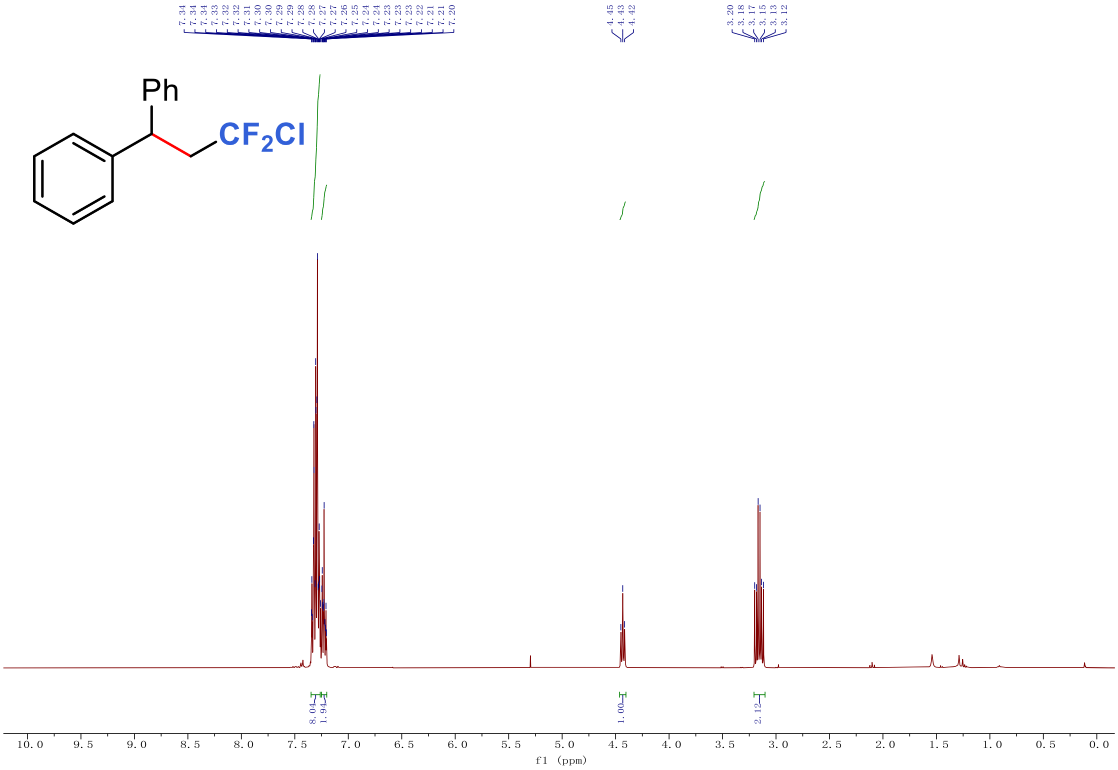

^{19}F NMR (377 MHz, CDCl3) spectra for compound **1au**

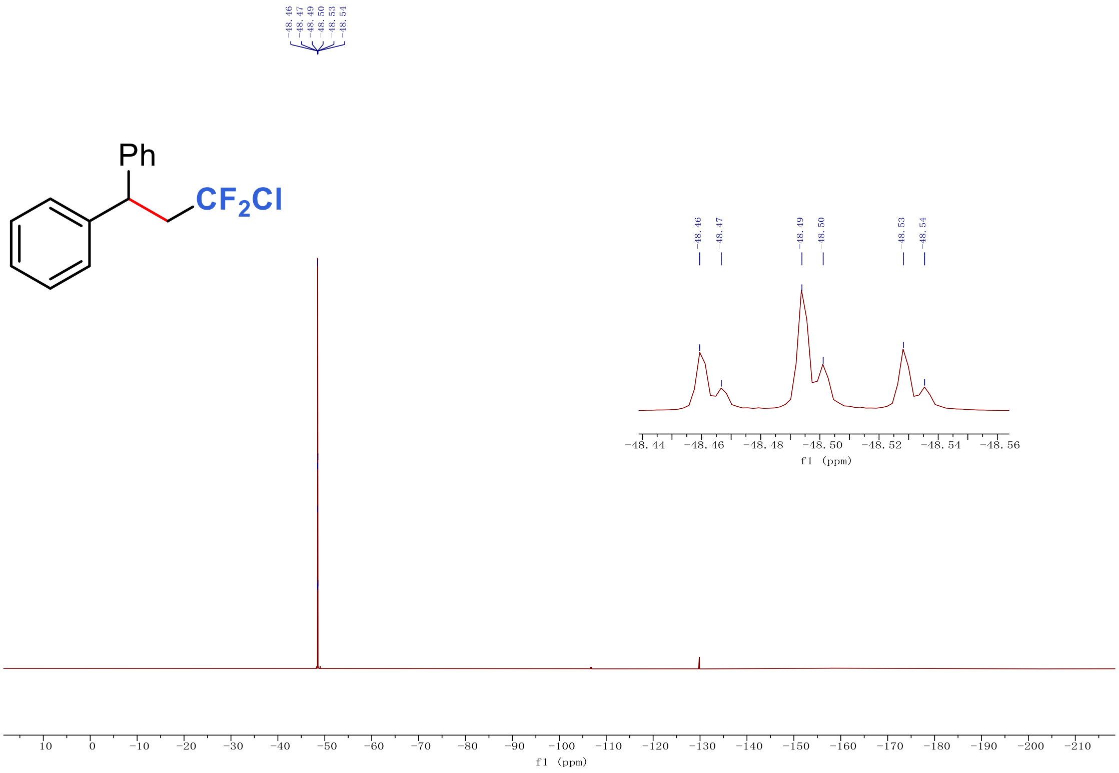

^{13}C NMR (126 MHz, CDCl3) spectra for compound **1au**

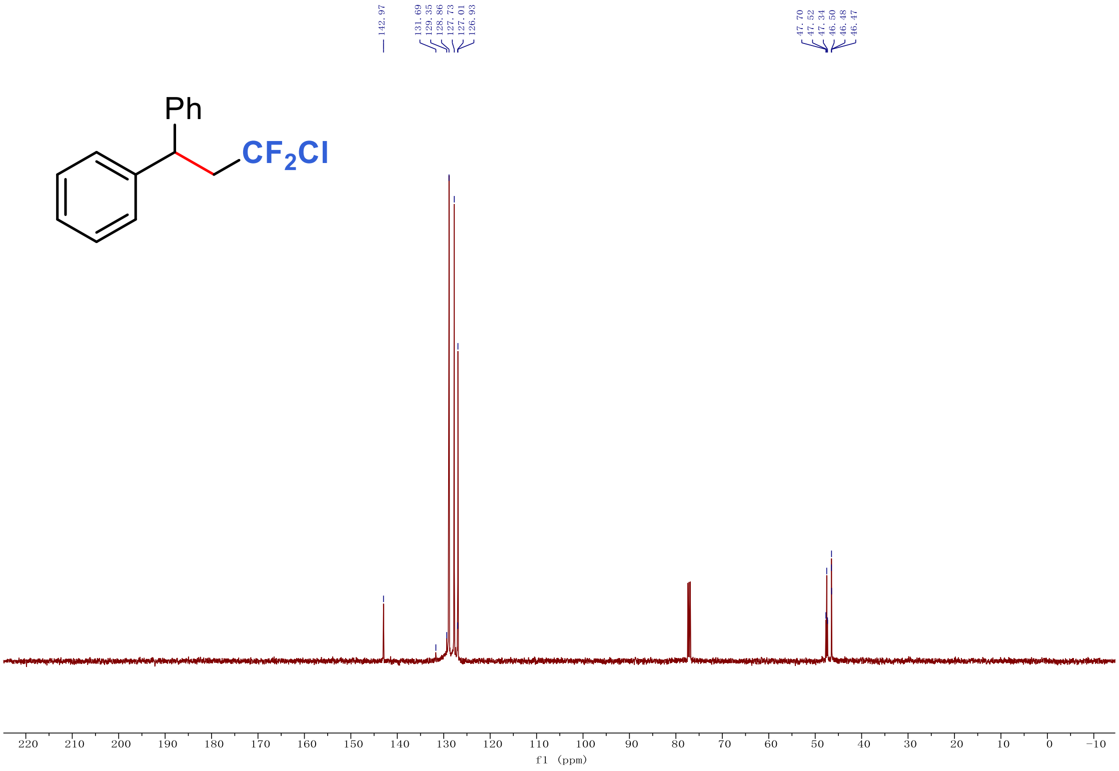

^{1}H NMR (400 MHz, $CDCl_3$) spectra for compound **1av**

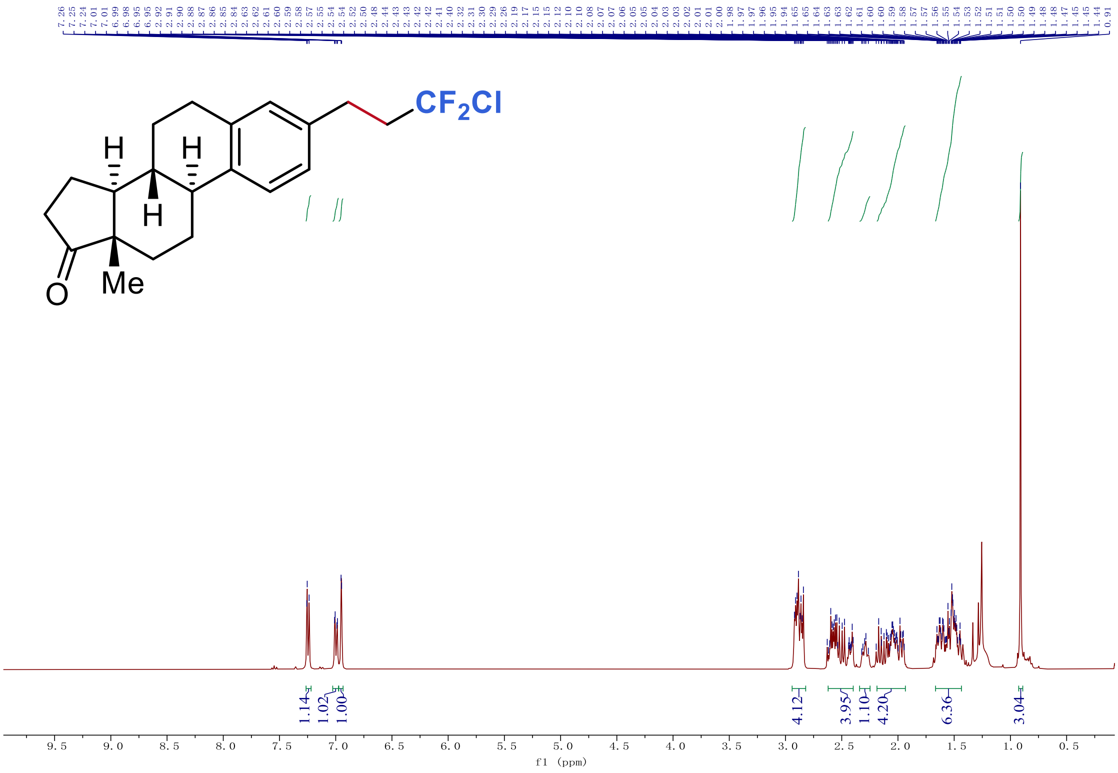

^{19}F NMR (377 MHz, $CDCl_3$) spectra for compound **1av**

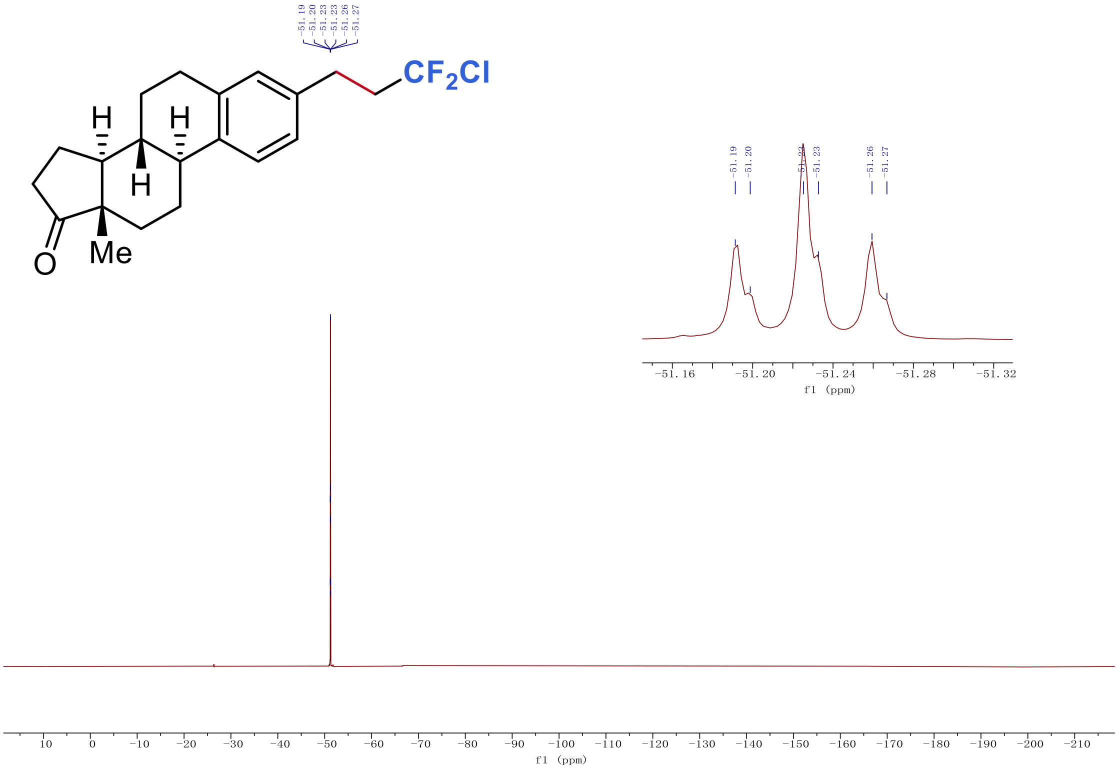

^{13}C NMR (151 MHz, $CDCl_3$) spectra for compound **1av**

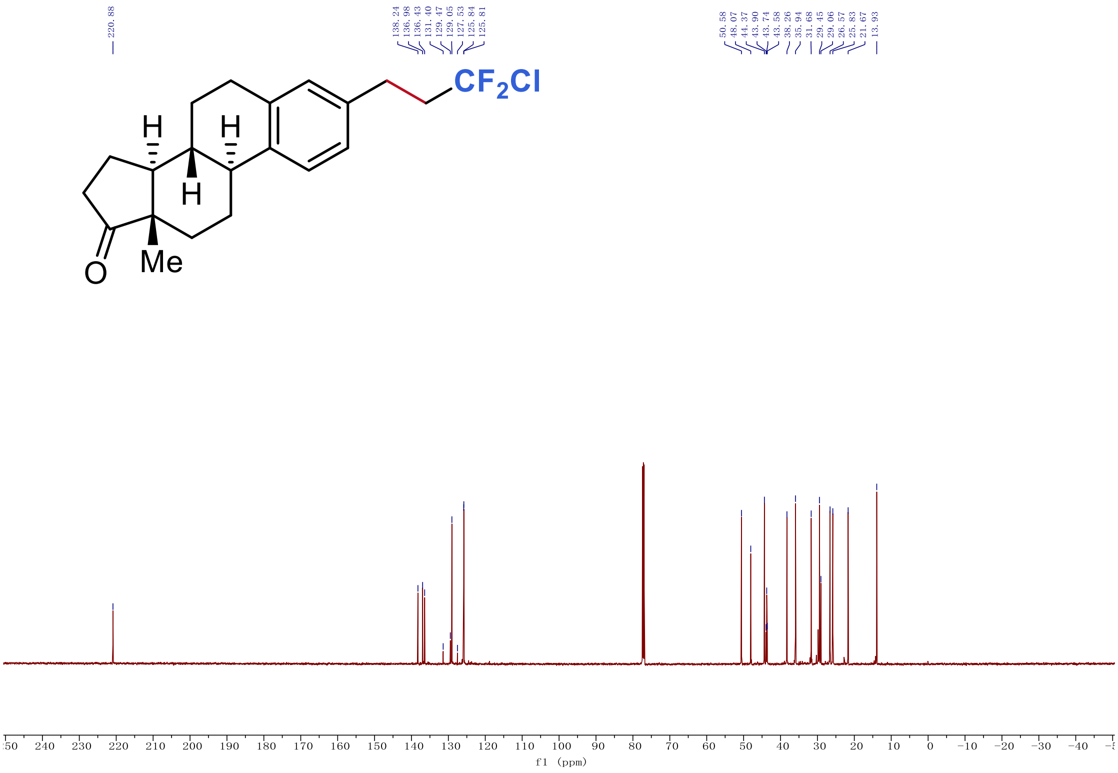

^{1}H NMR (400 MHz, $CDCl_3$) spectra for compound **1aw**

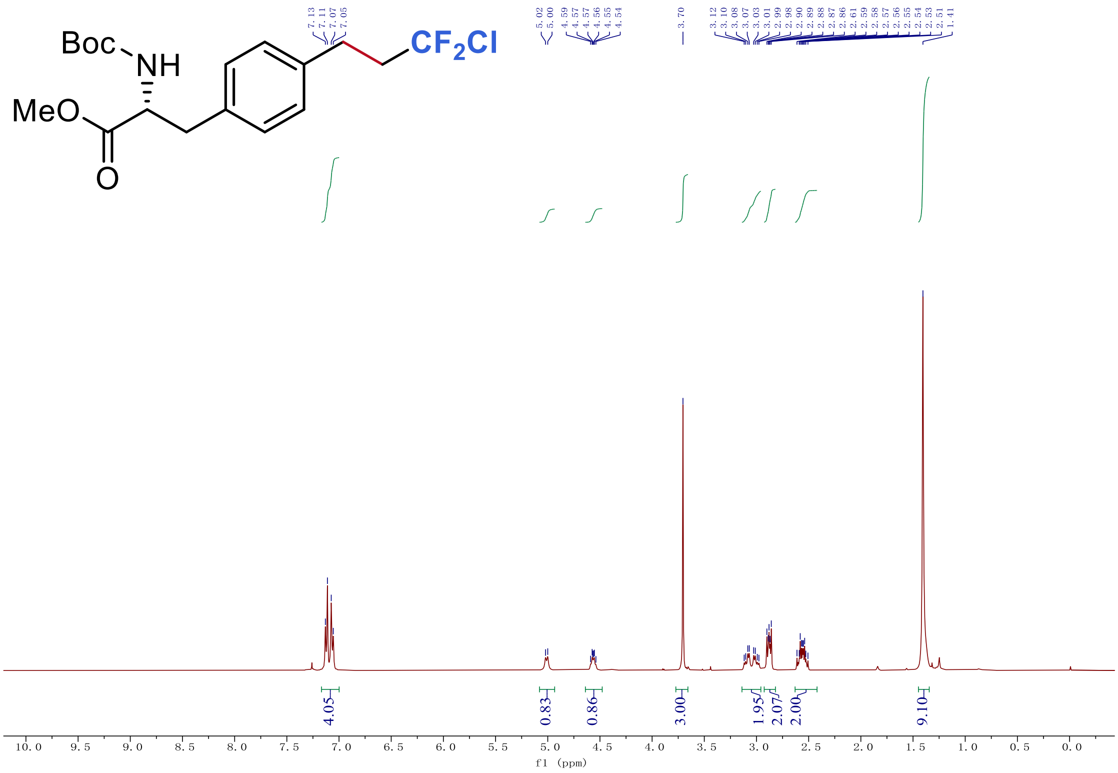

^{19}F NMR (377 MHz, $CDCl_3$) spectra for compound **1aw**

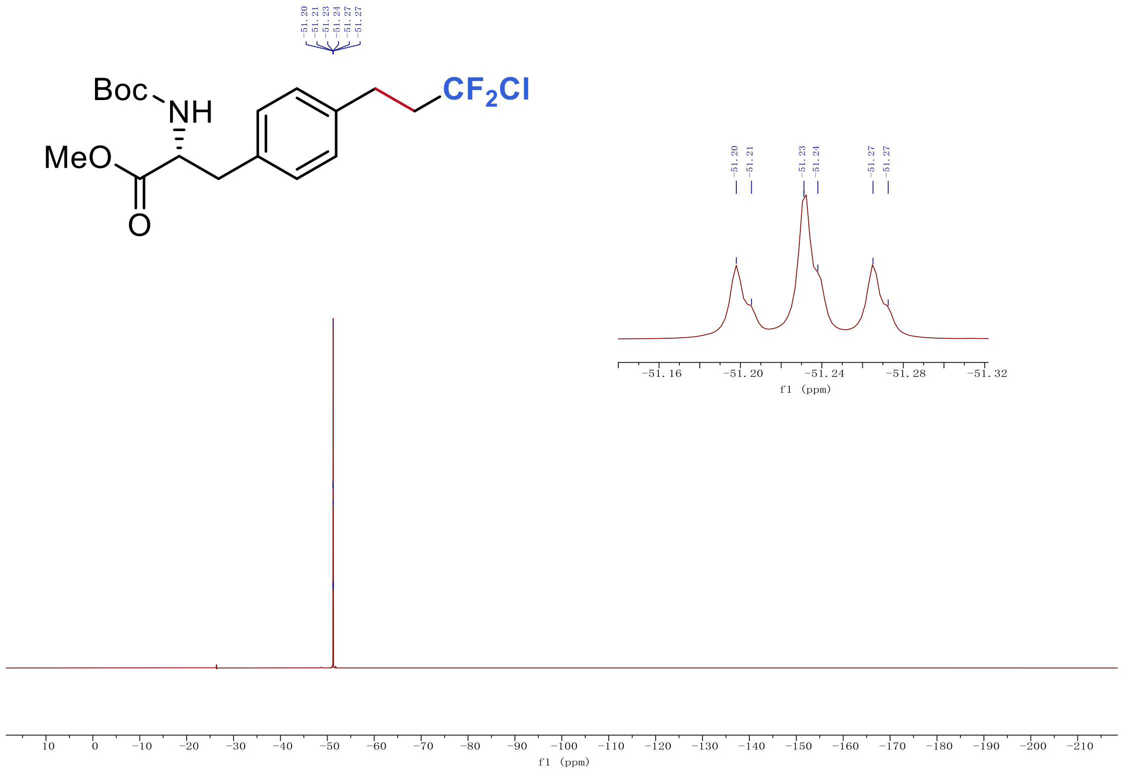

^{13}C NMR (126 MHz, $CDCl_3$) spectra for compound **1aw**

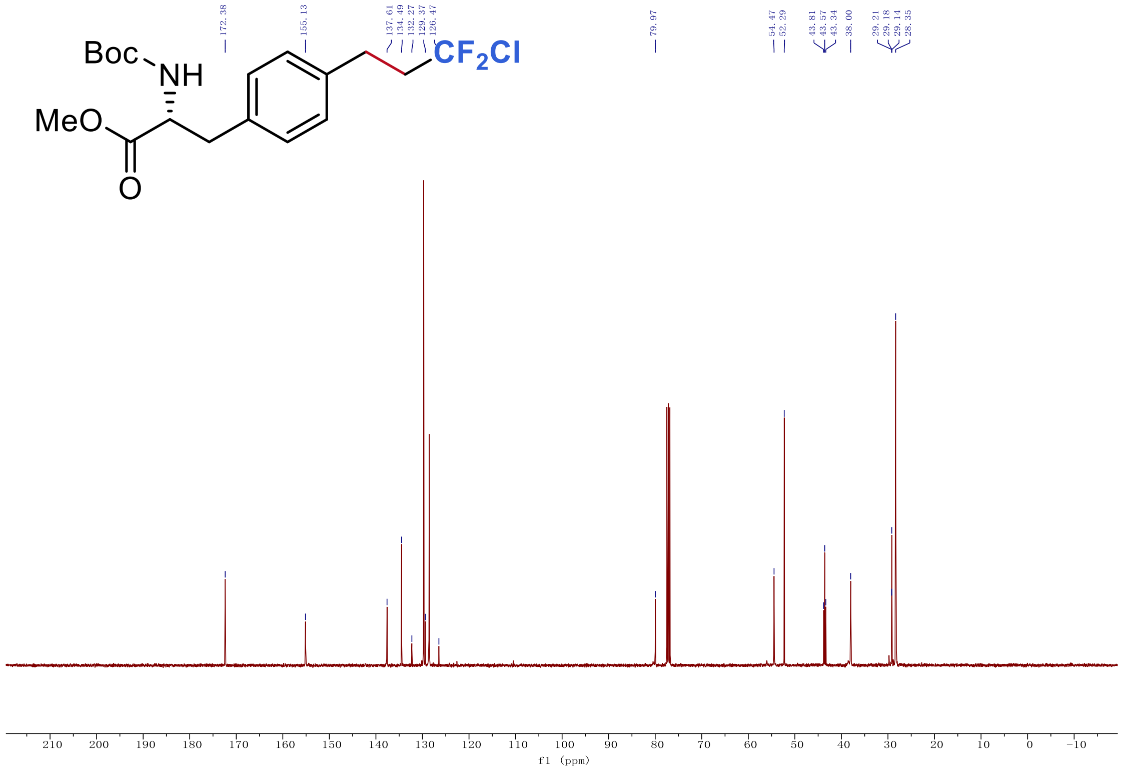

^{1}H NMR (400 MHz, $CDCl_3$) spectra for compound **2a**

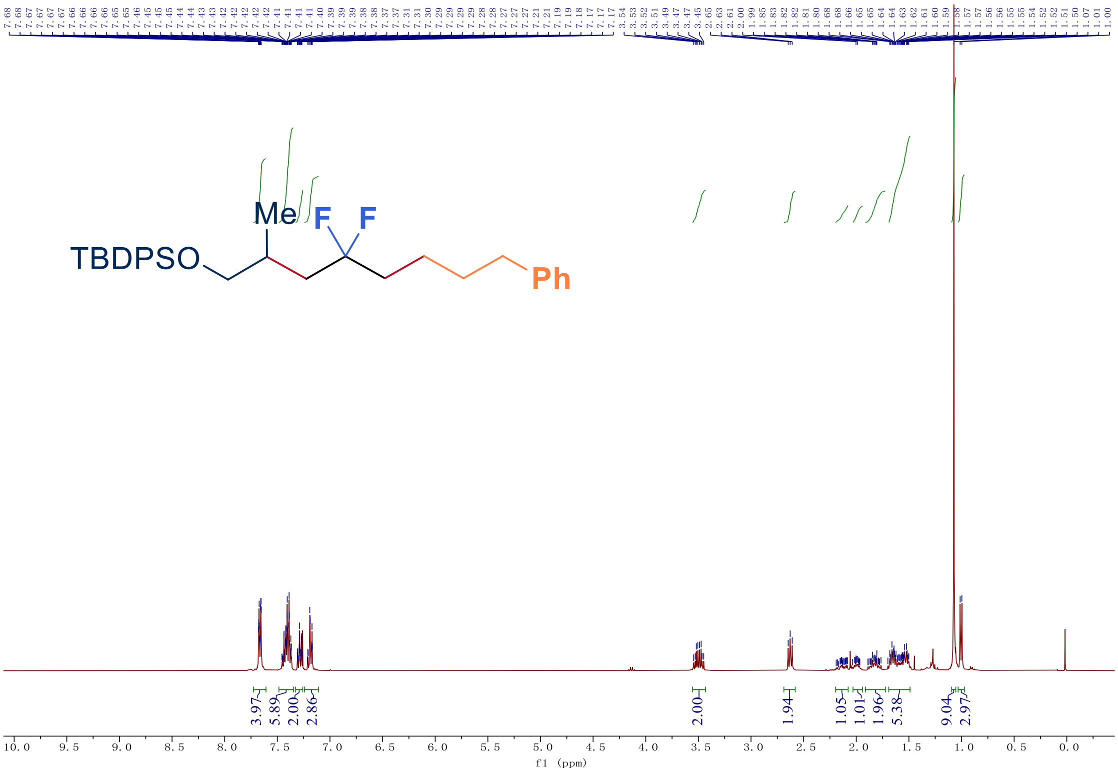

^{19}F NMR (377 MHz, $CDCl_3$) spectra for compound **2a**

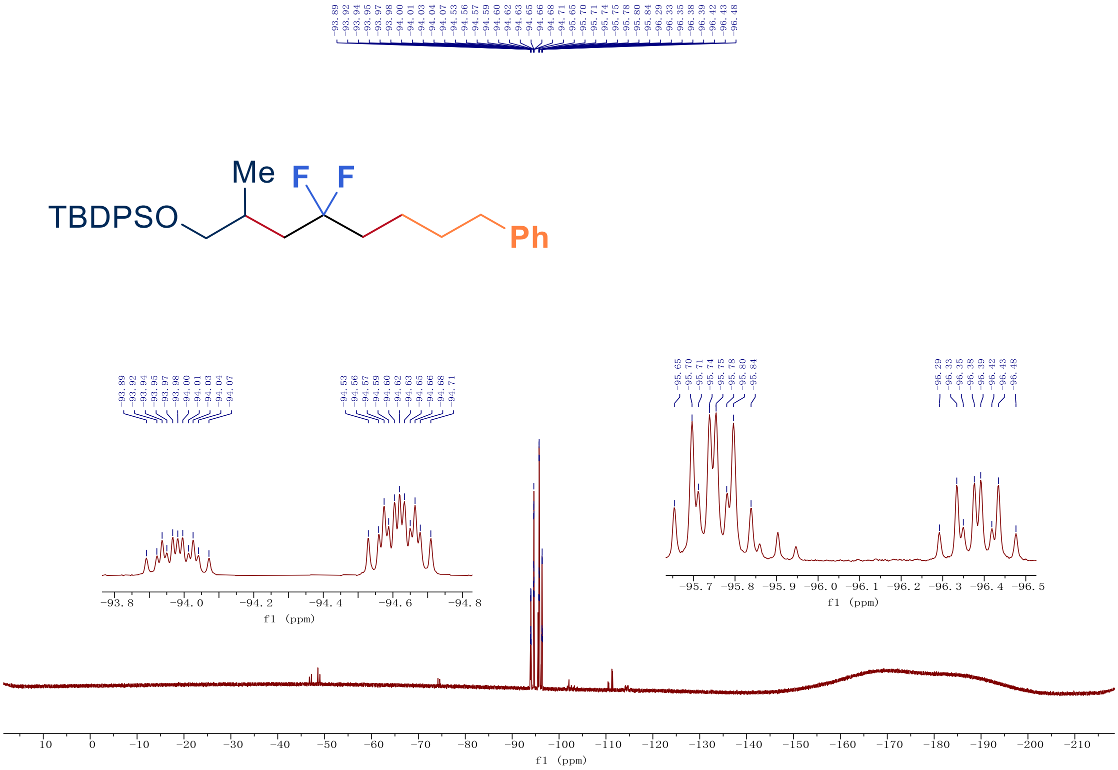

^{13}C NMR (126 MHz, $CDCl_3$) spectra for compound **2a**

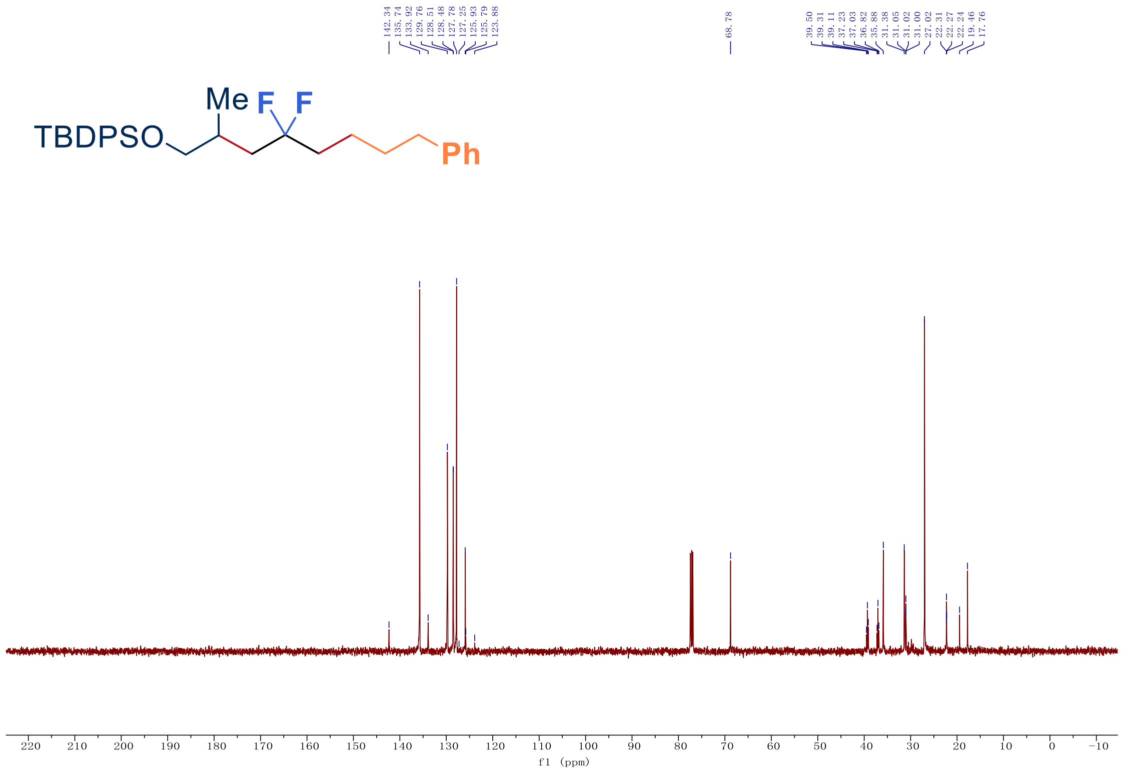

^{1}H NMR (400 MHz, $CDCl_3$) spectra for compound **2b**

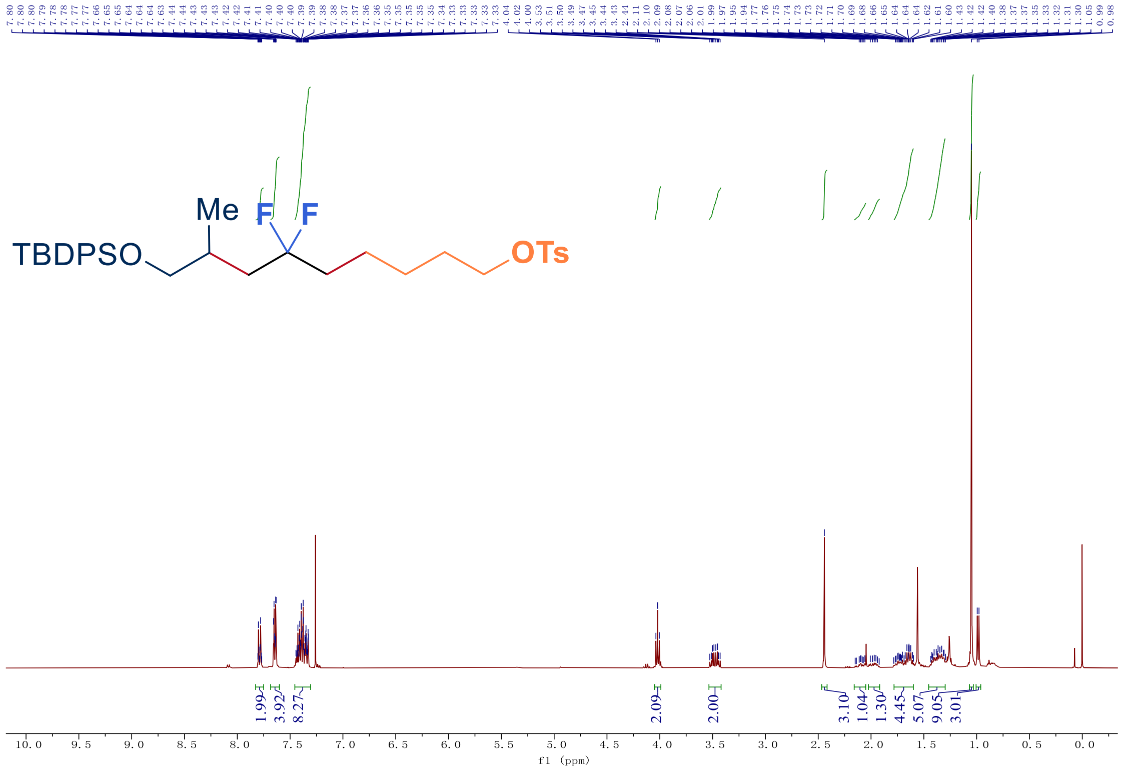

^{19}F NMR (377 MHz, $CDCl_3$) spectra for compound **2b**

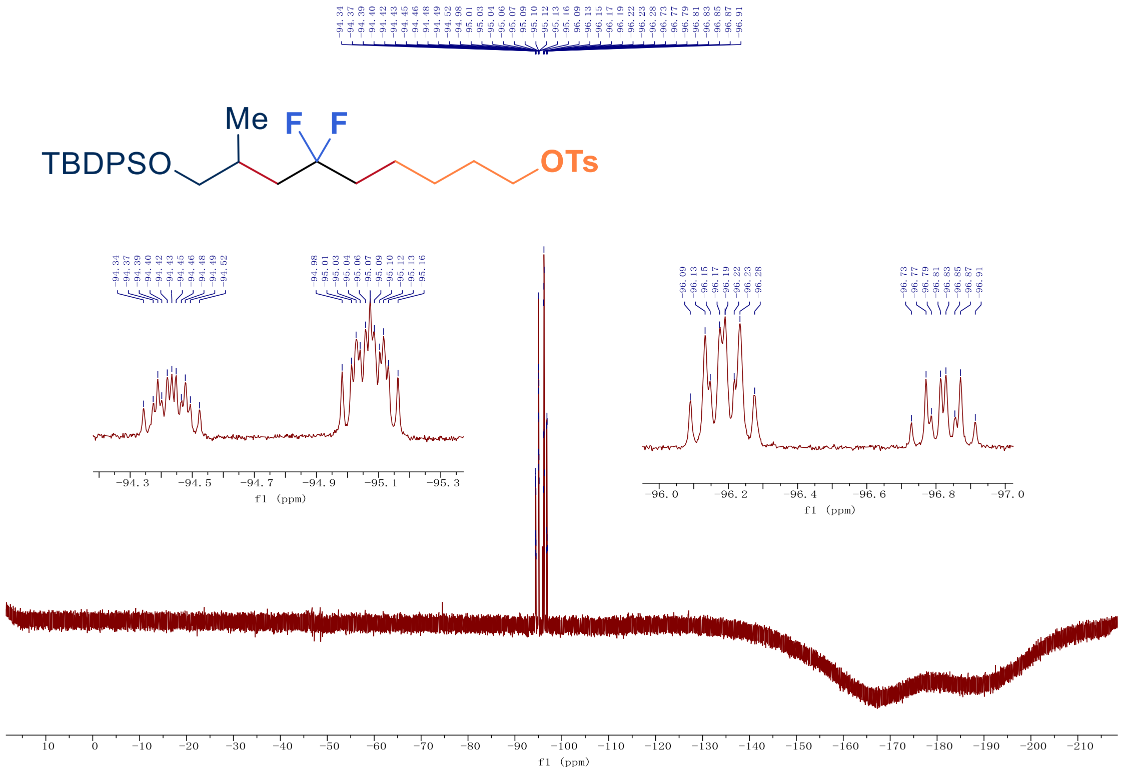

^{13}C NMR (126 MHz, $CDCl_3$) spectra for compound **2b**

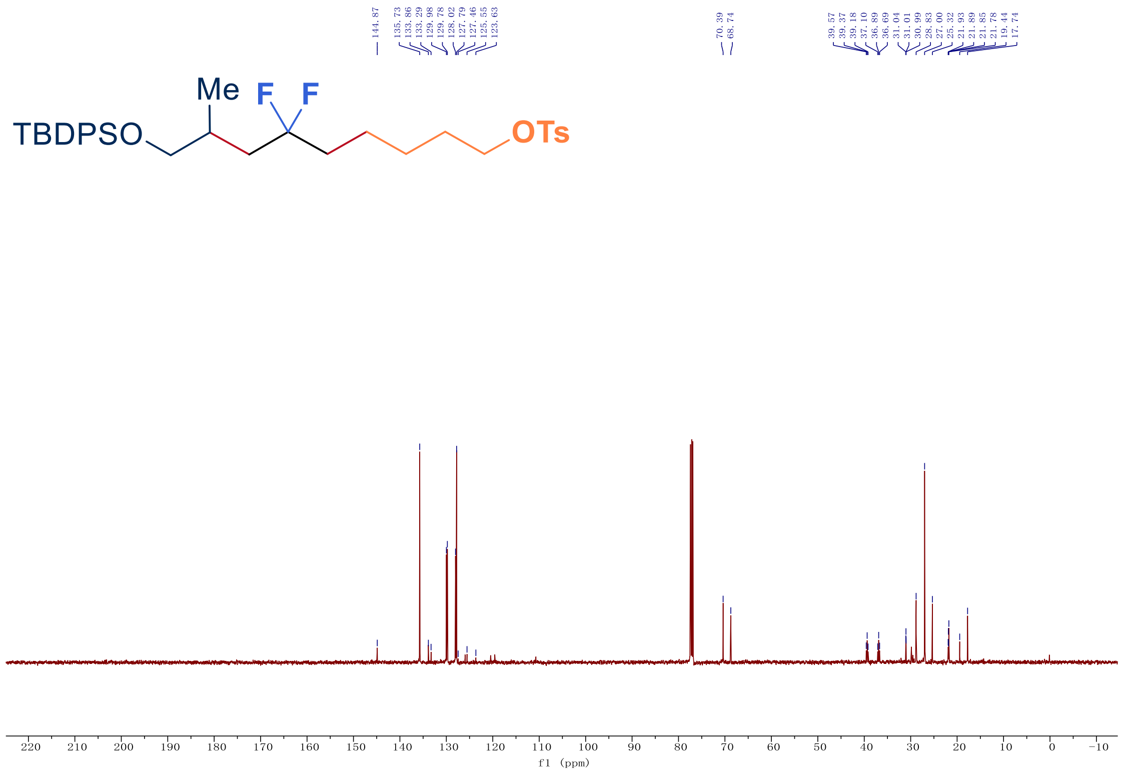

^{1}H NMR (400 MHz, $CDCl_3$) spectra for compound **2c**

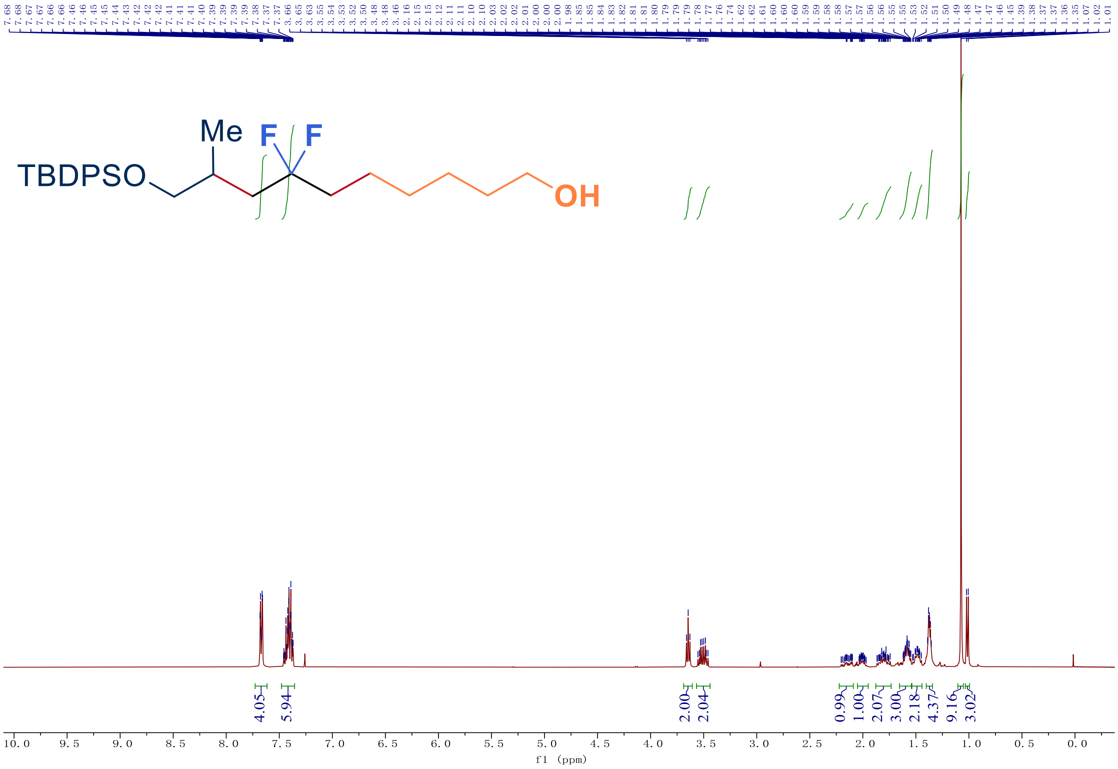

^{19}F NMR (377 MHz, $CDCl_3$) spectra for compound **2c**

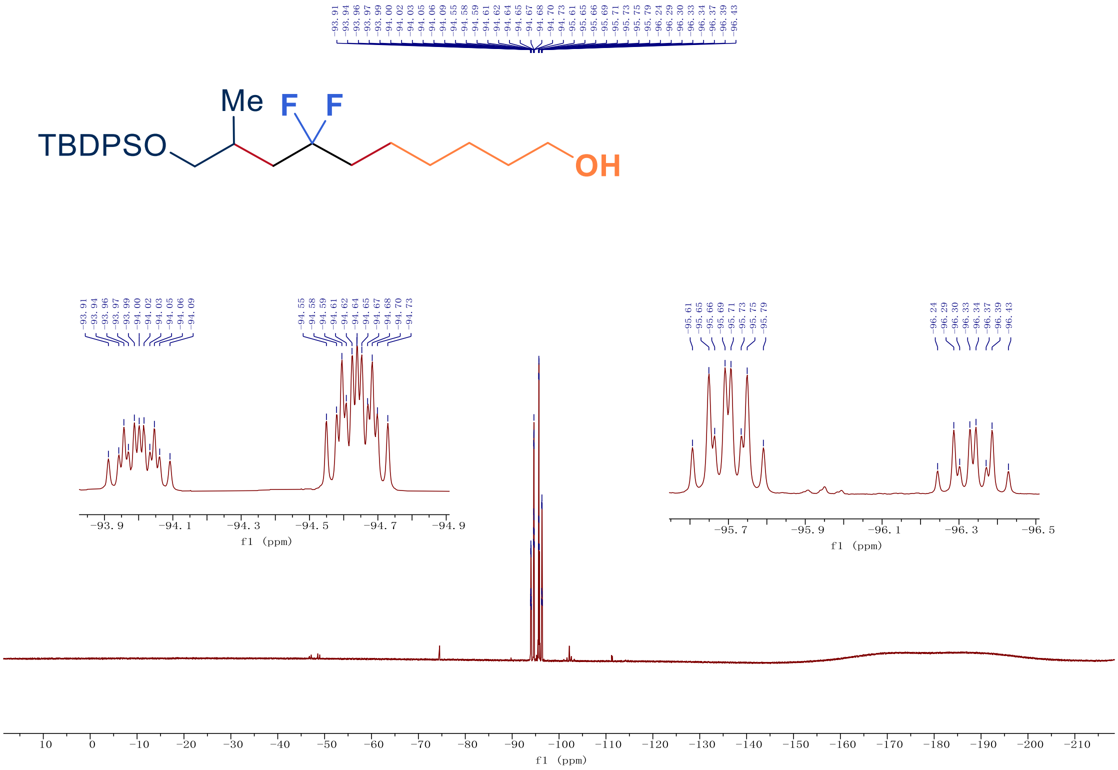

^{13}C NMR (126 MHz, $CDCl_3$) spectra for compound **2c**

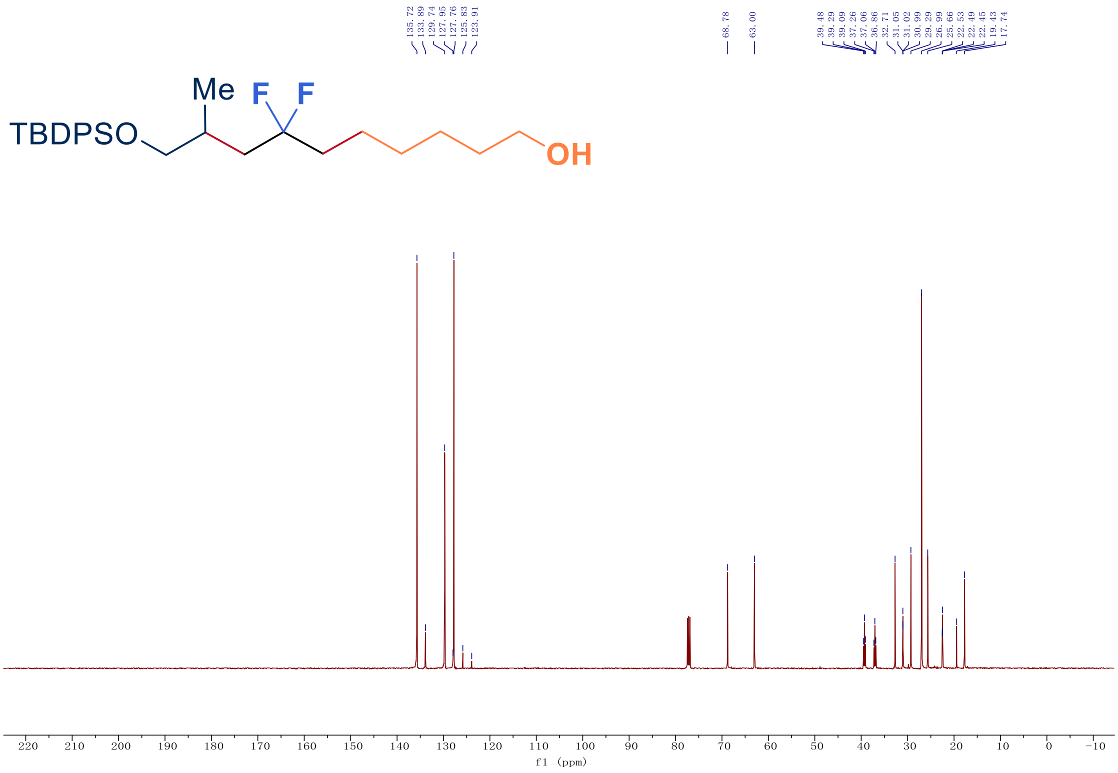

^{1}H NMR (400 MHz, $CDCl_3$) spectra for compound **2d**

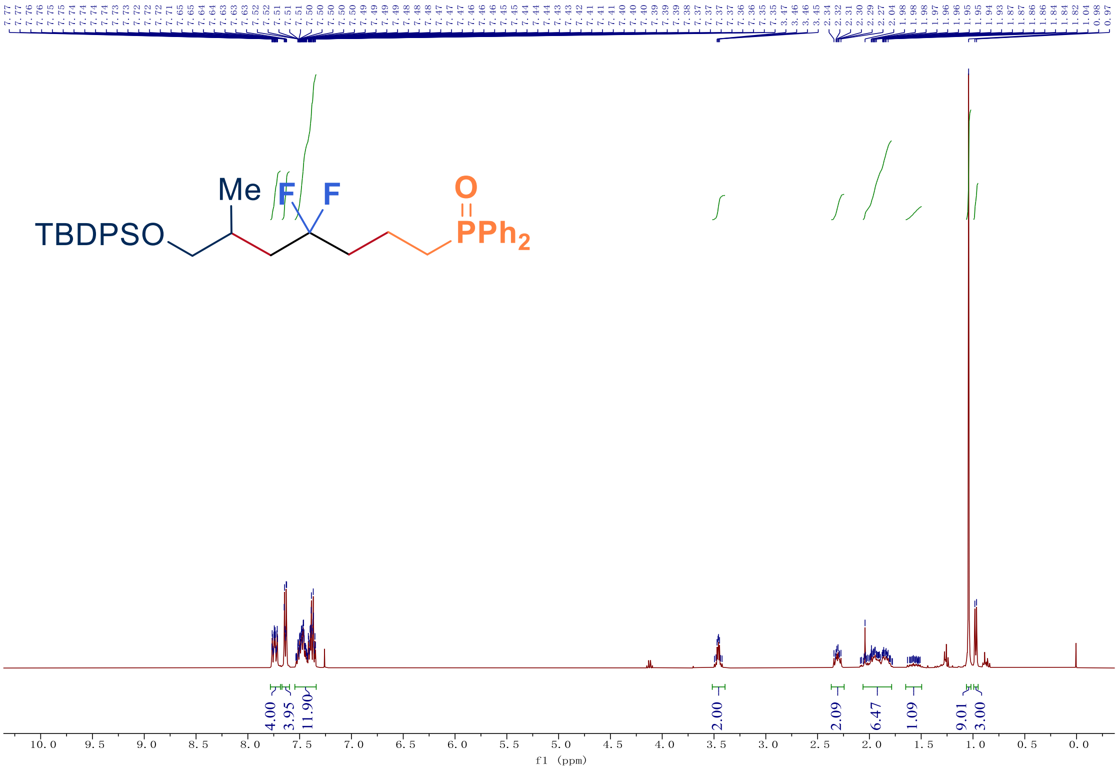

^{19}F NMR (377 MHz, $CDCl_3$) spectra for compound **2d**

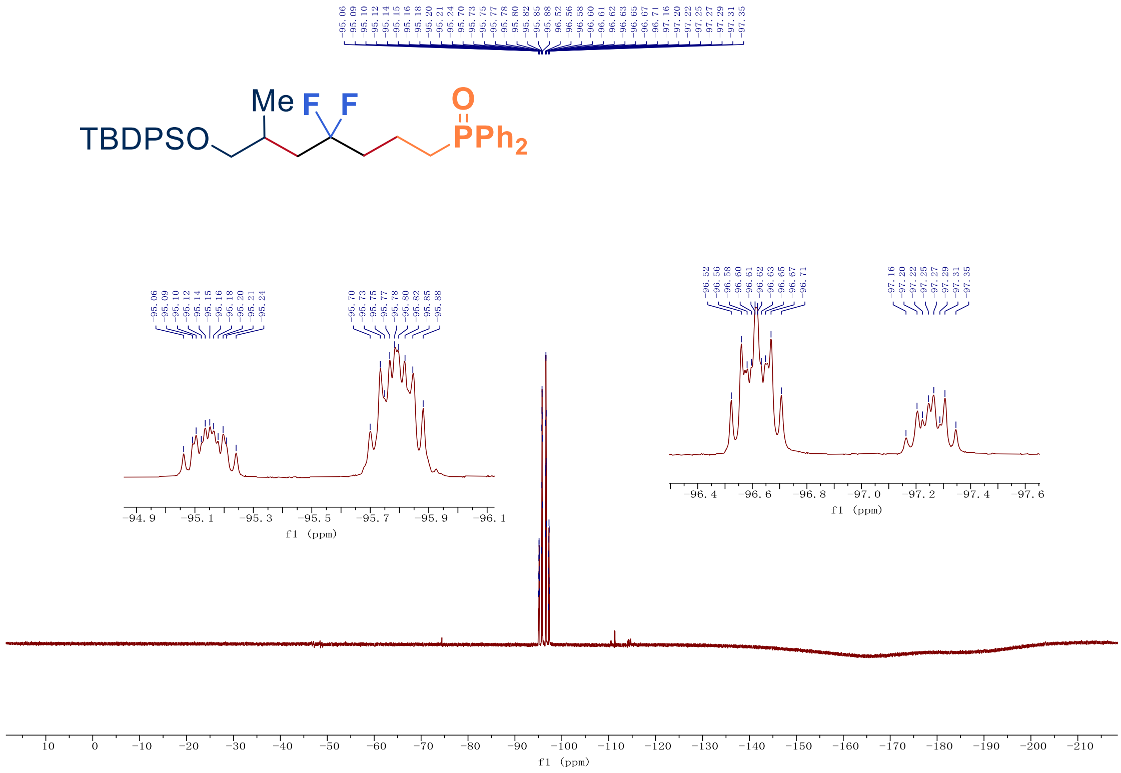

^{31}P NMR (162 MHz, $CDCl_3$) spectra for compound **2d**

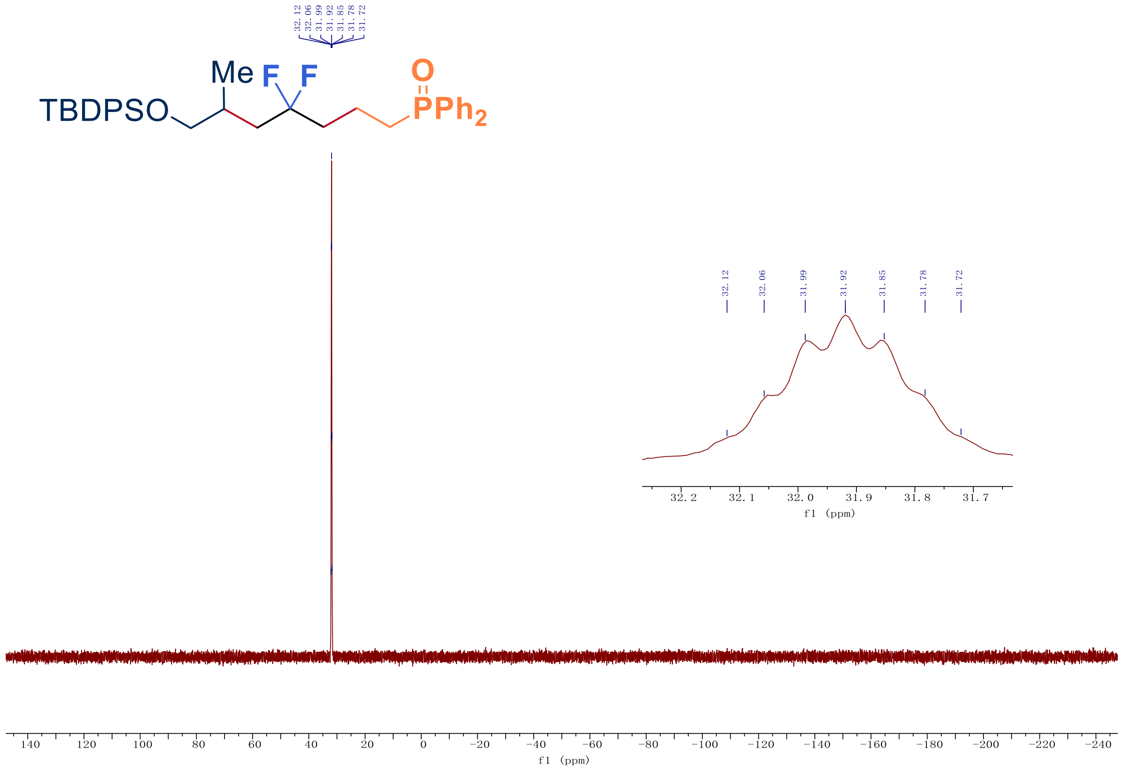

^{13}C NMR (126 MHz, $CDCl_3$) spectra for compound **2d**

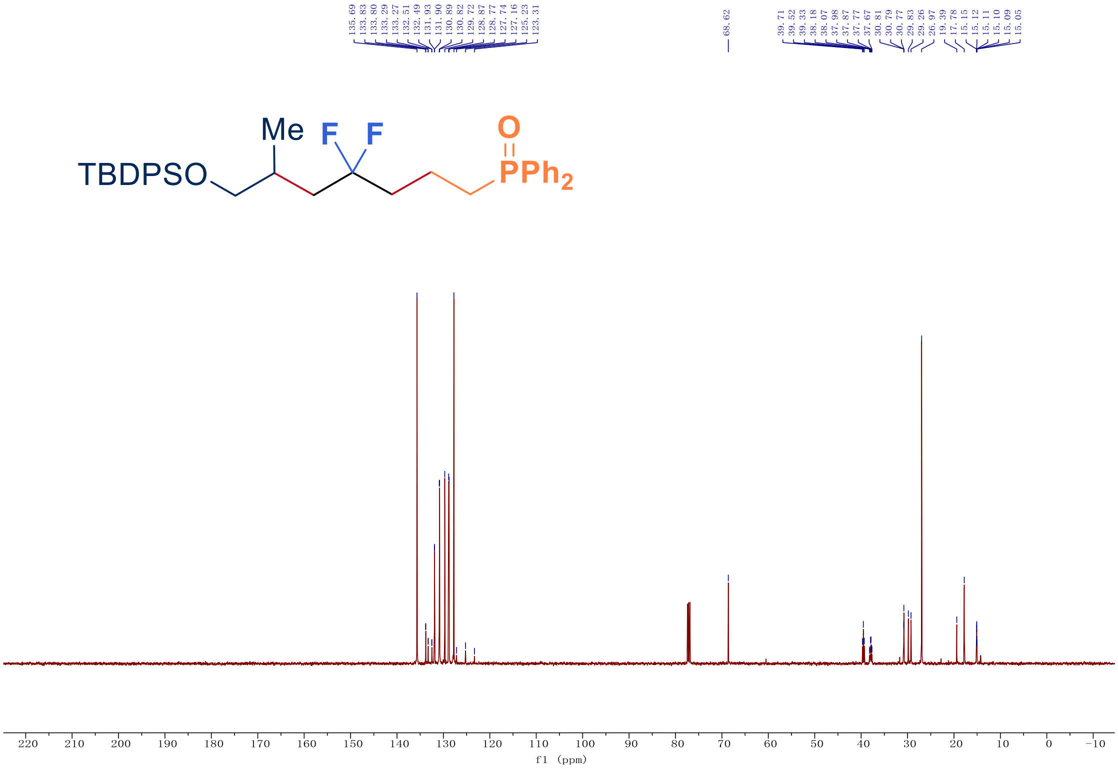

^{1}H NMR (400 MHz, $CDCl_3$) spectra for compound **2e**

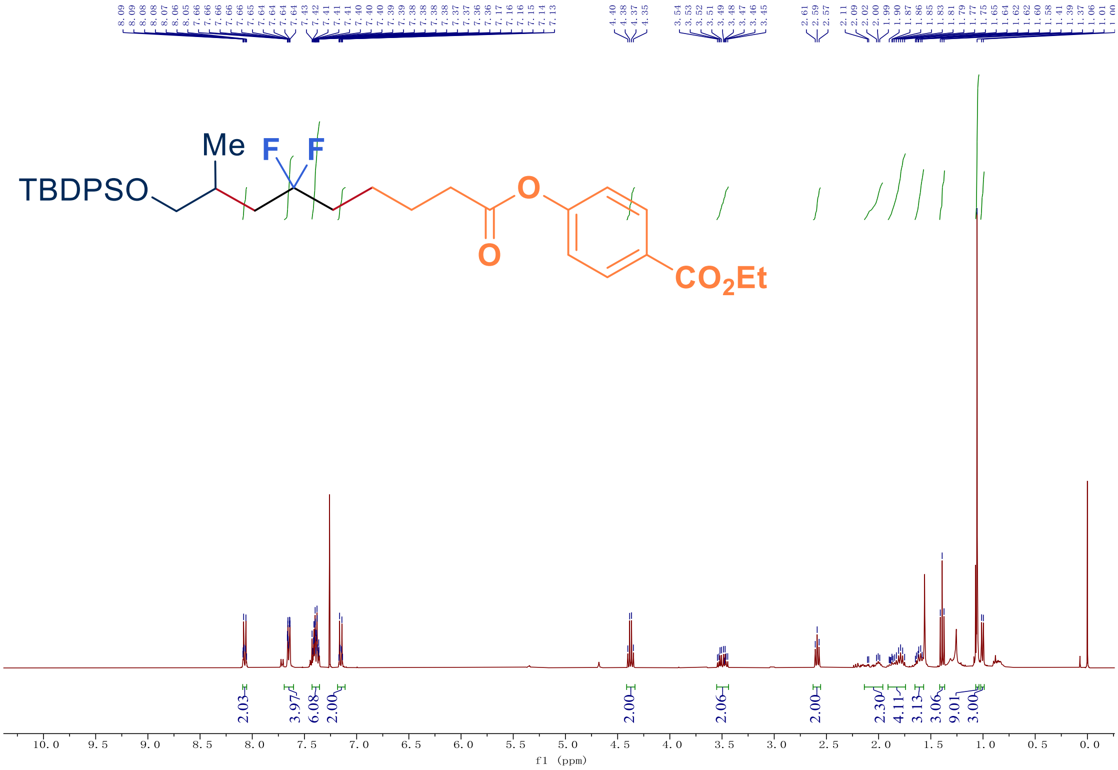

^{19}F NMR (377 MHz, $CDCl_3$) spectra for compound **2e**

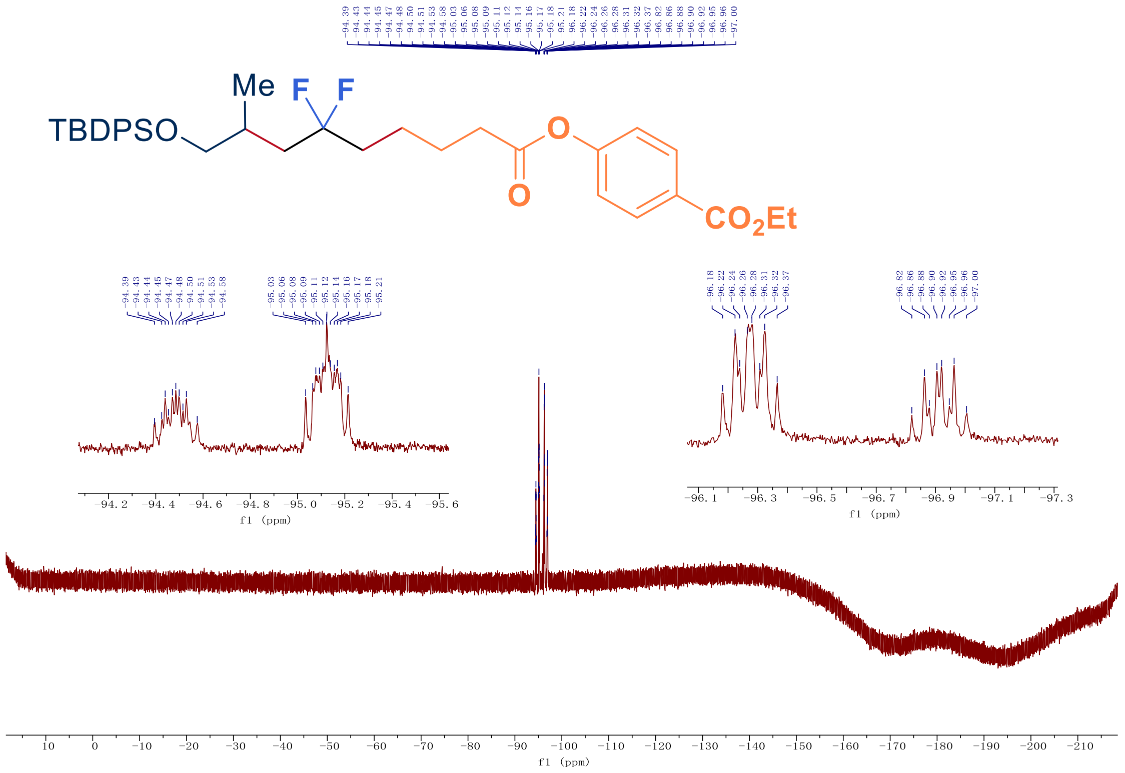

^{13}C NMR (126 MHz, $CDCl_3$) spectra for compound **2e**

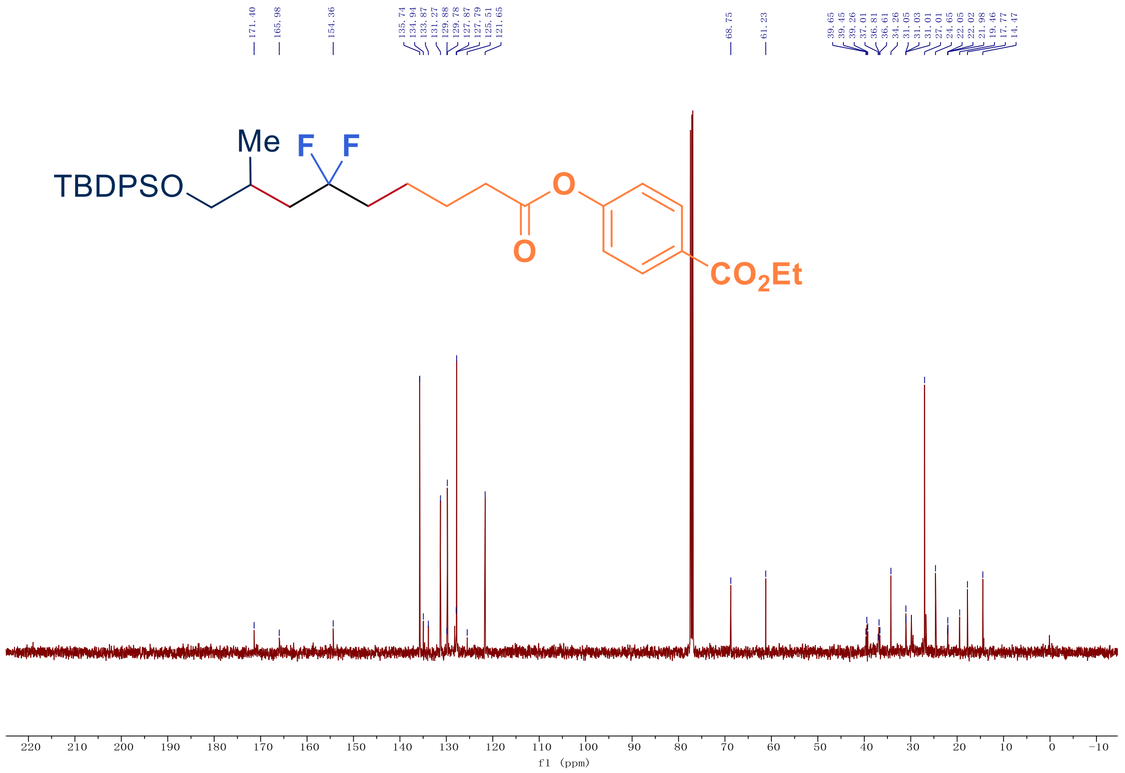

^{1}H NMR (400 MHz, $CDCl_3$) spectra for compound **2f**

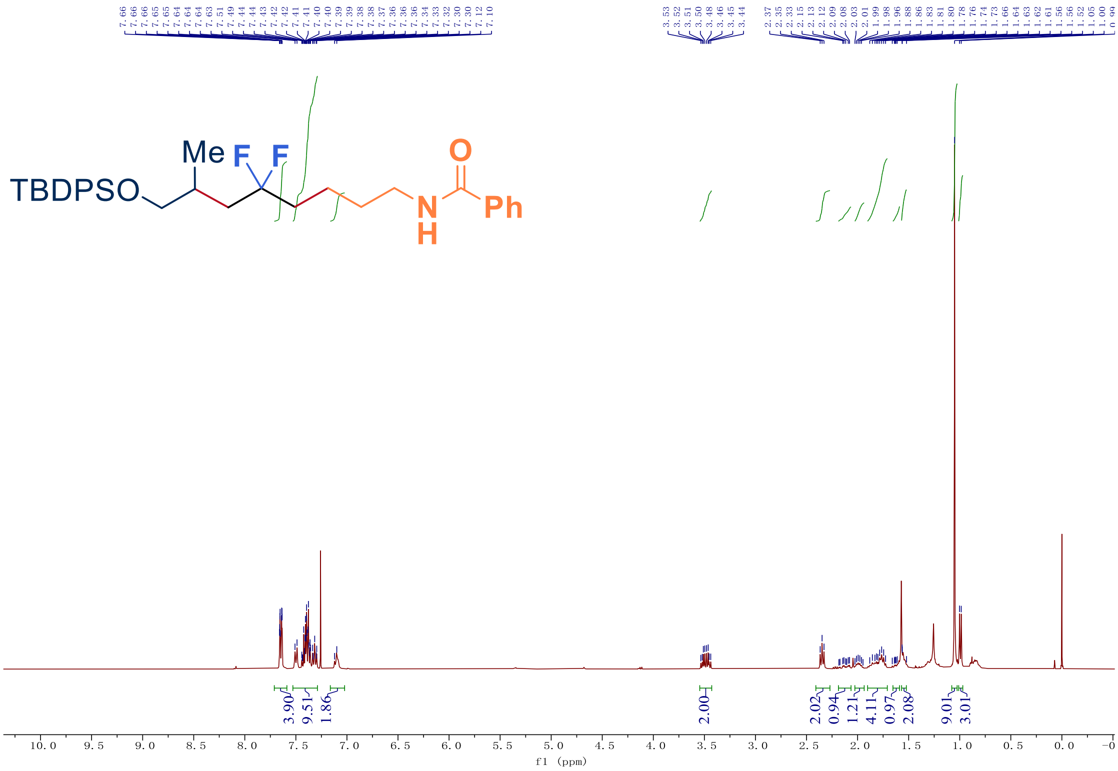

^{19}F NMR (377 MHz, $CDCl_3$) spectra for compound **2f**

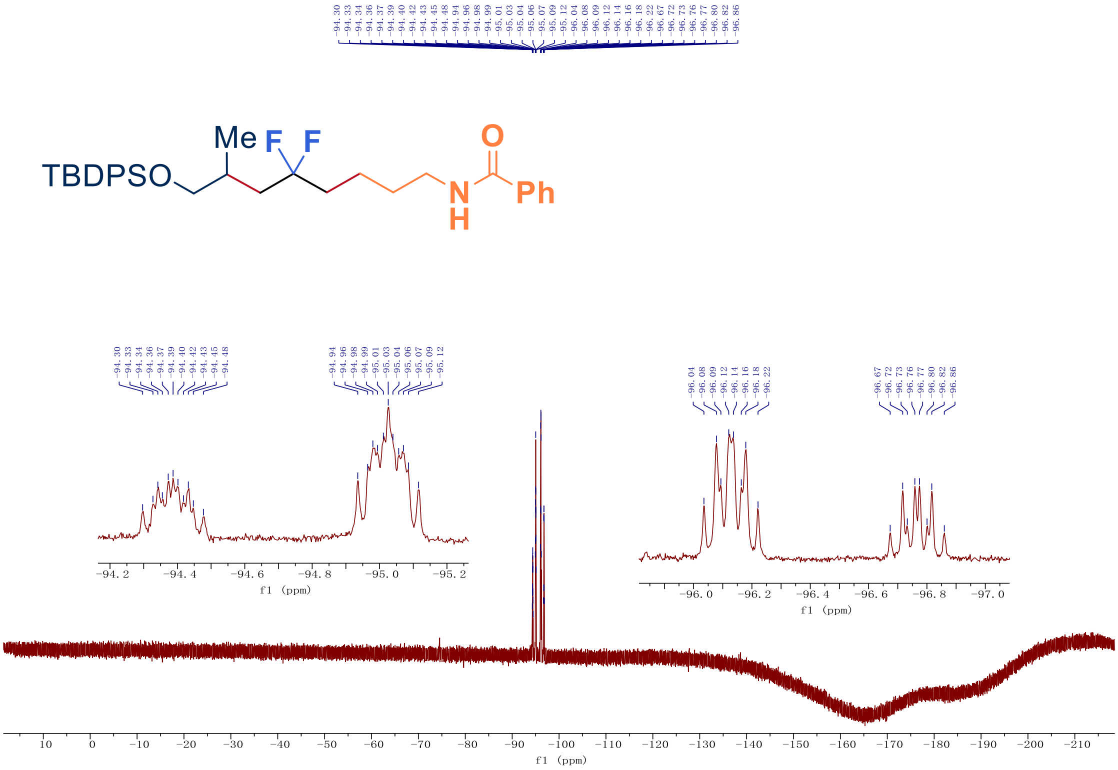

13C NMR (126 MHz, $CDCl_3$) spectra for compound **2f**

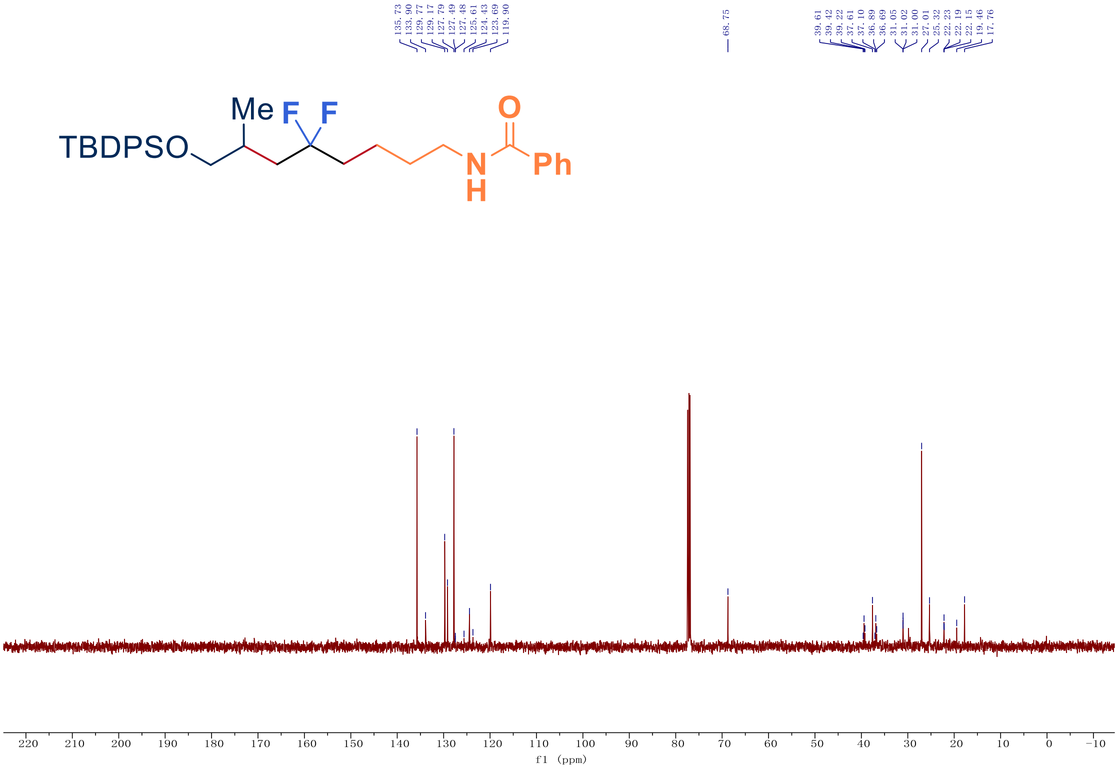

1H NMR (400 MHz, $CDCl_3$) spectra for compound **2g**

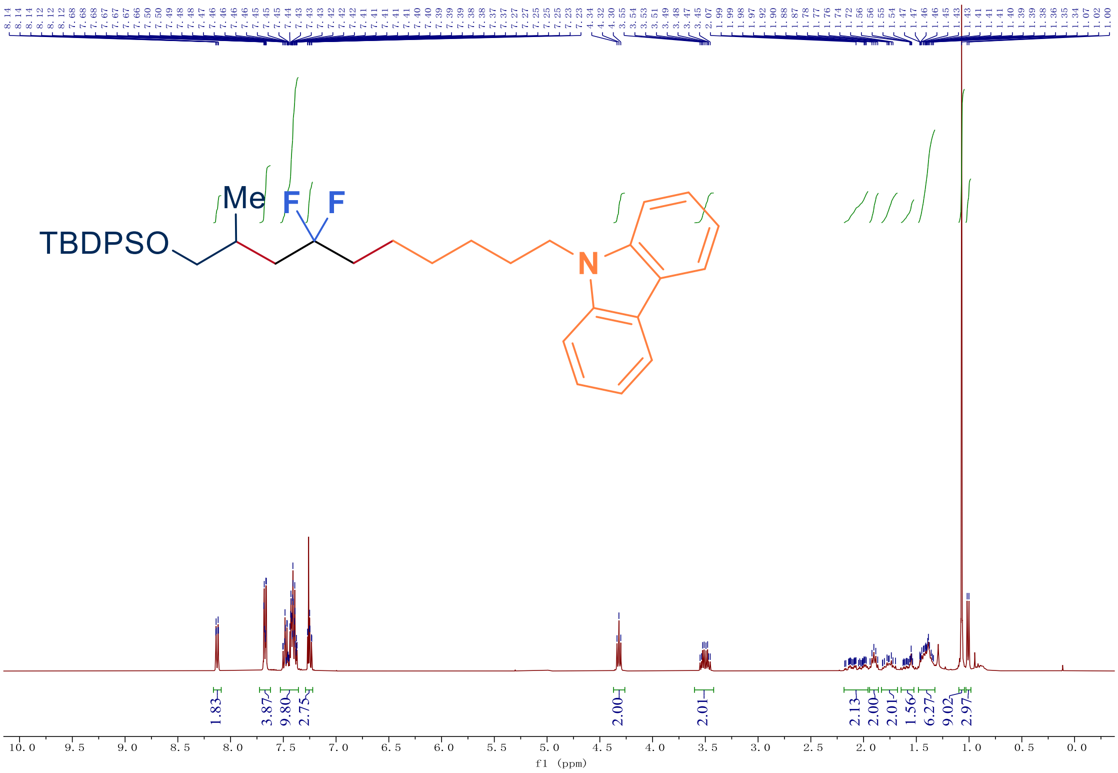

^{19}F NMR (377 MHz, $CDCl_3$) spectra for compound **2g**

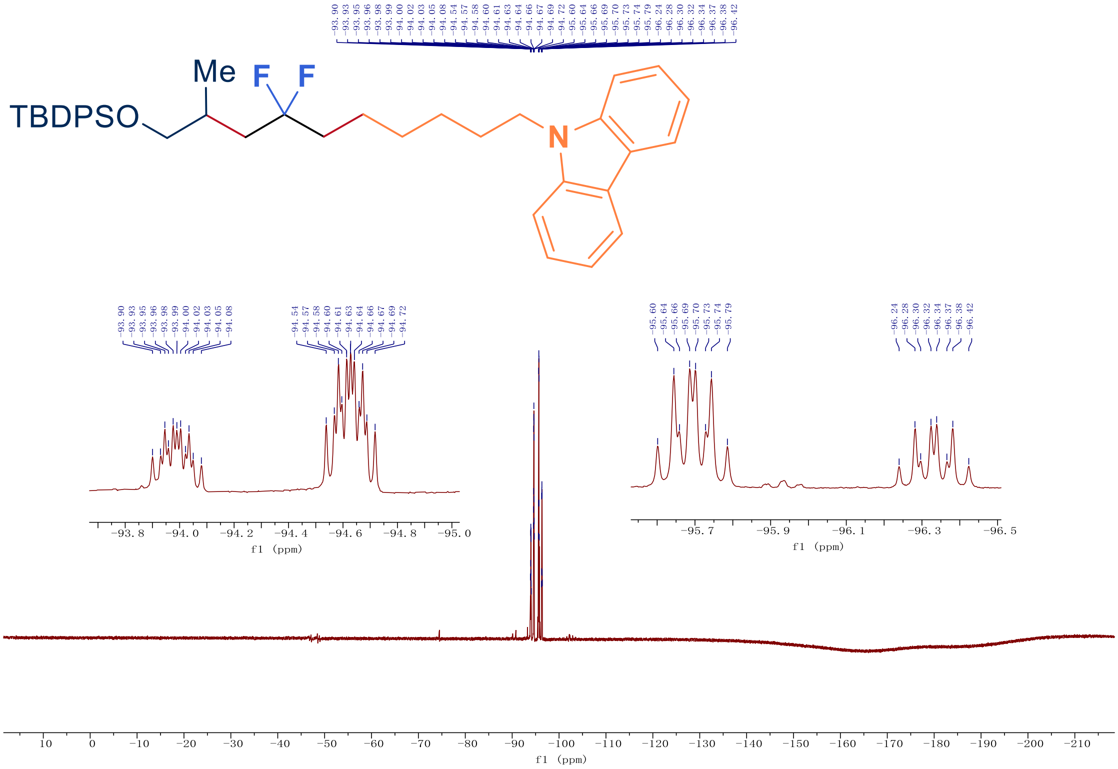

^{13}C NMR (126 MHz, $CDCl_3$) spectra for compound **2g**

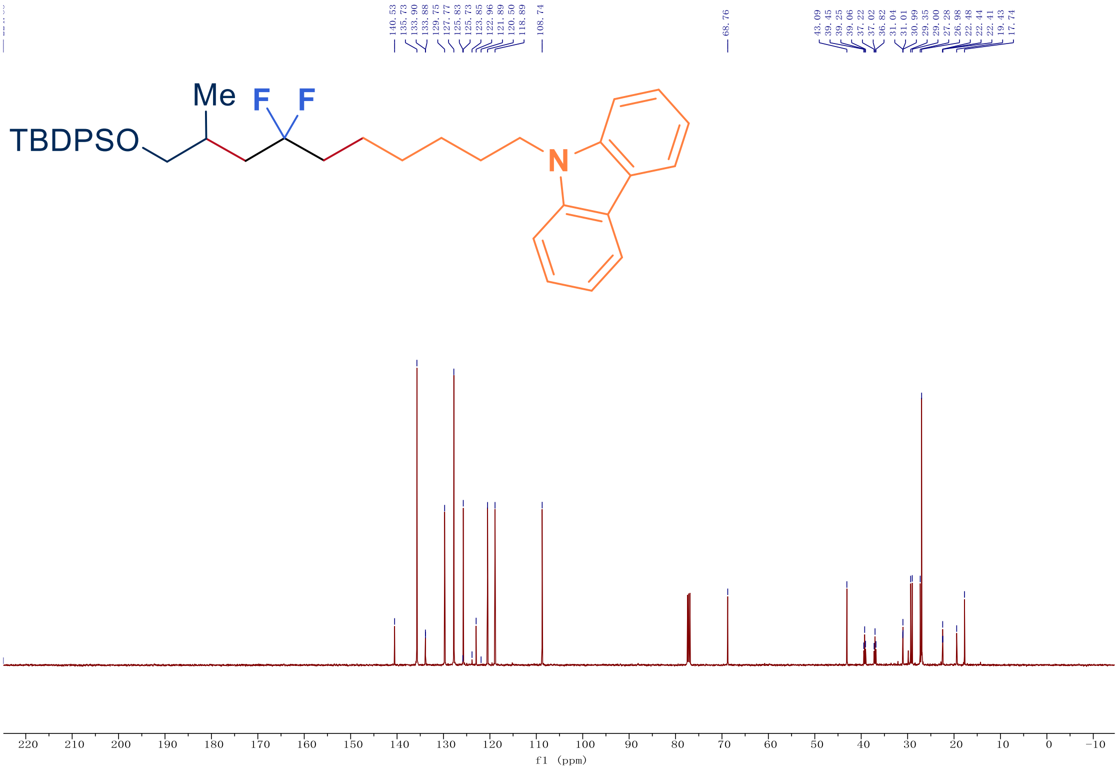

^{1}H NMR (400 MHz, $CDCl_3$) spectra for compound **2h**

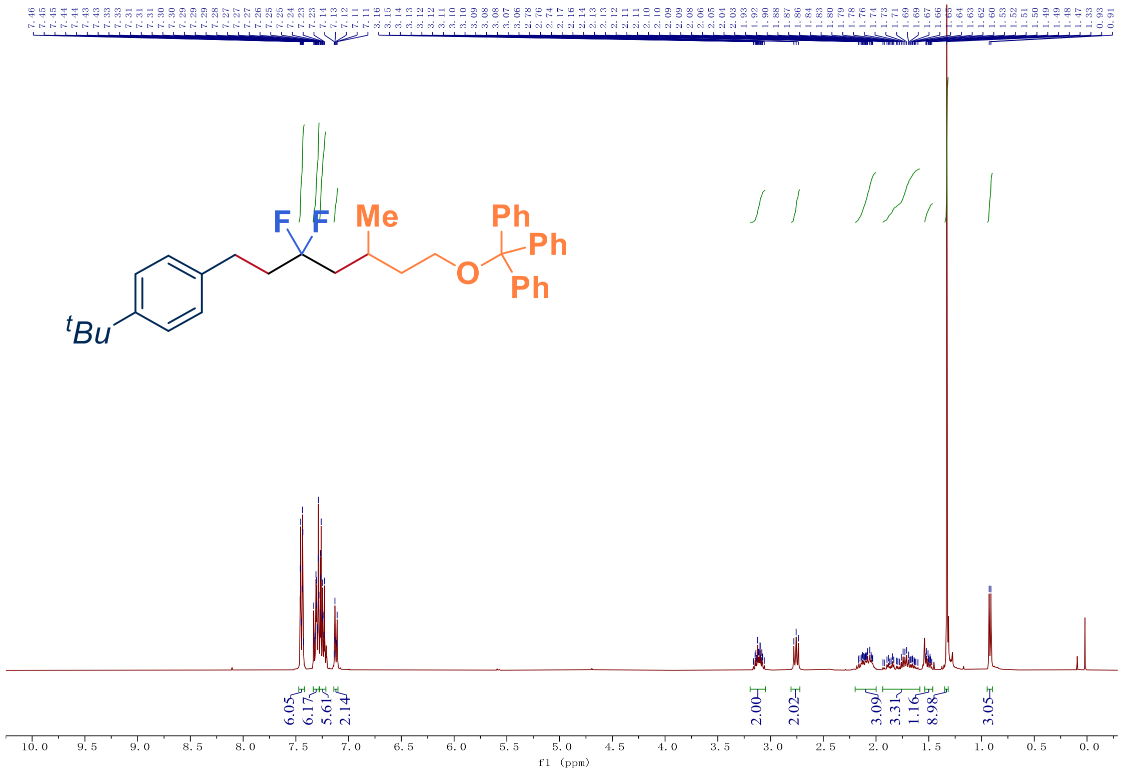

^{19}F NMR (377 MHz, $CDCl_3$) spectra for compound **2h**

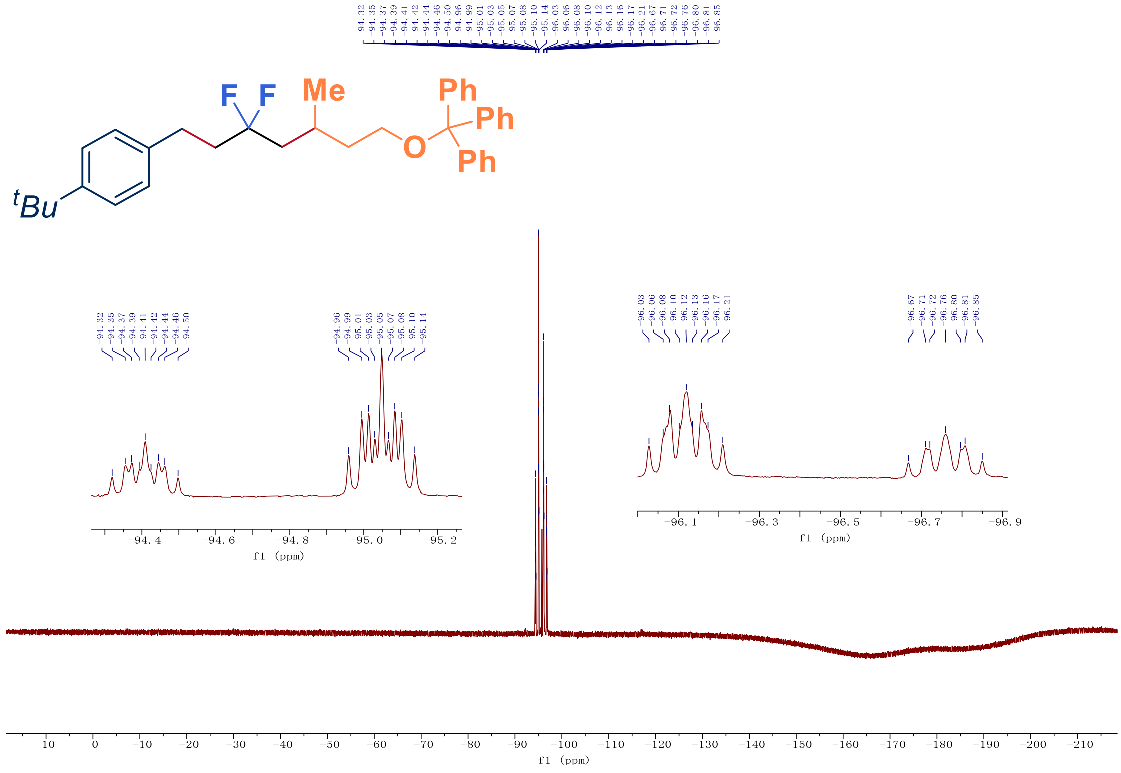

^{13}C NMR (126 MHz, $CDCl_3$) spectra for compound **2h**

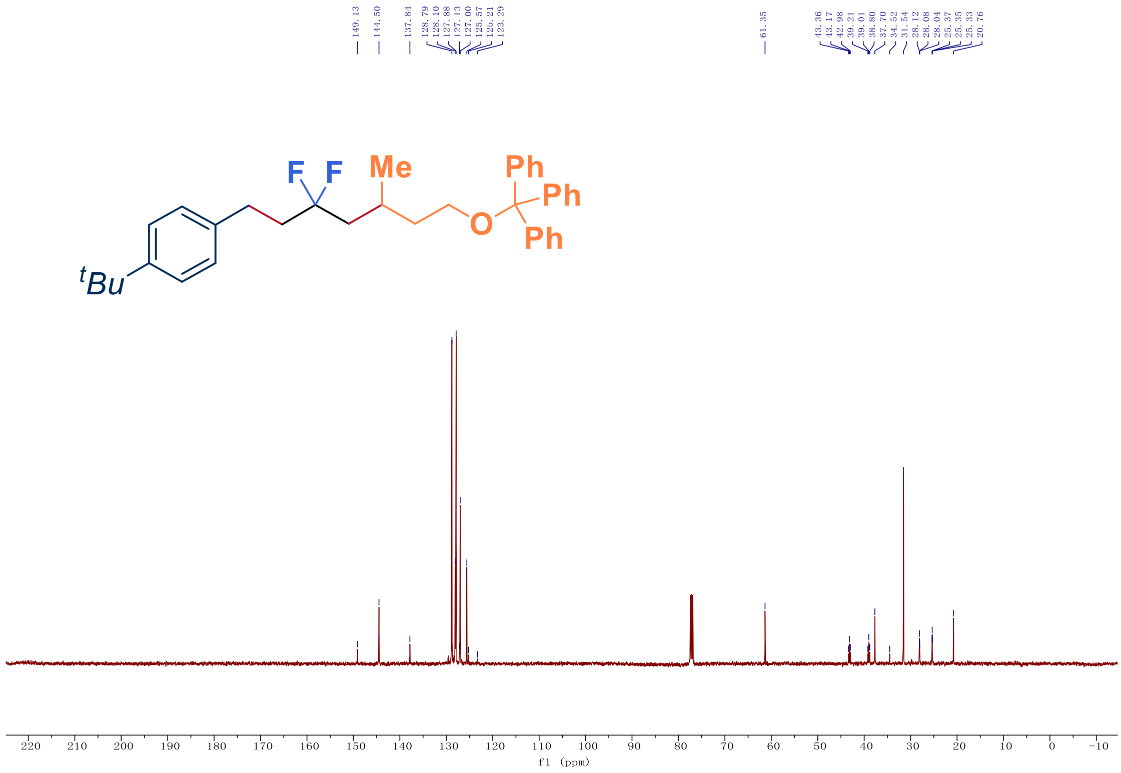

^{1}H NMR (400 MHz, $CDCl_3$) spectra for compound **2i**

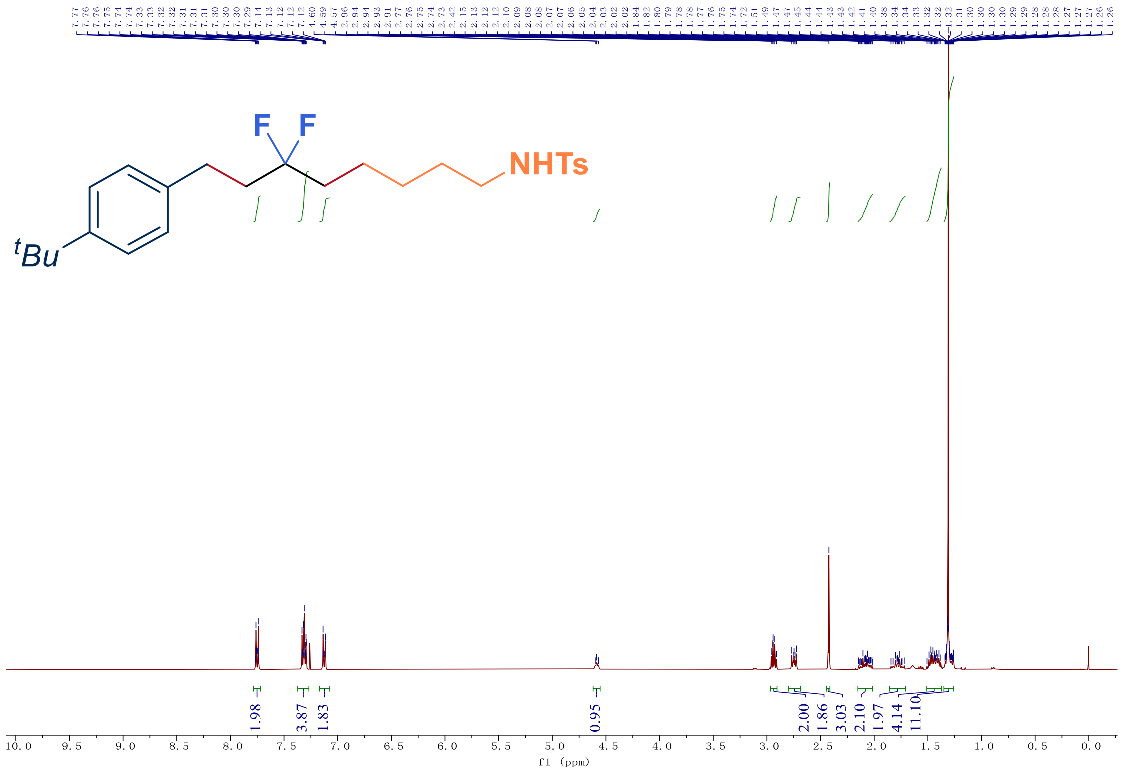

^{19}F NMR (377 MHz, $CDCl_3$) spectra for compound **2i**

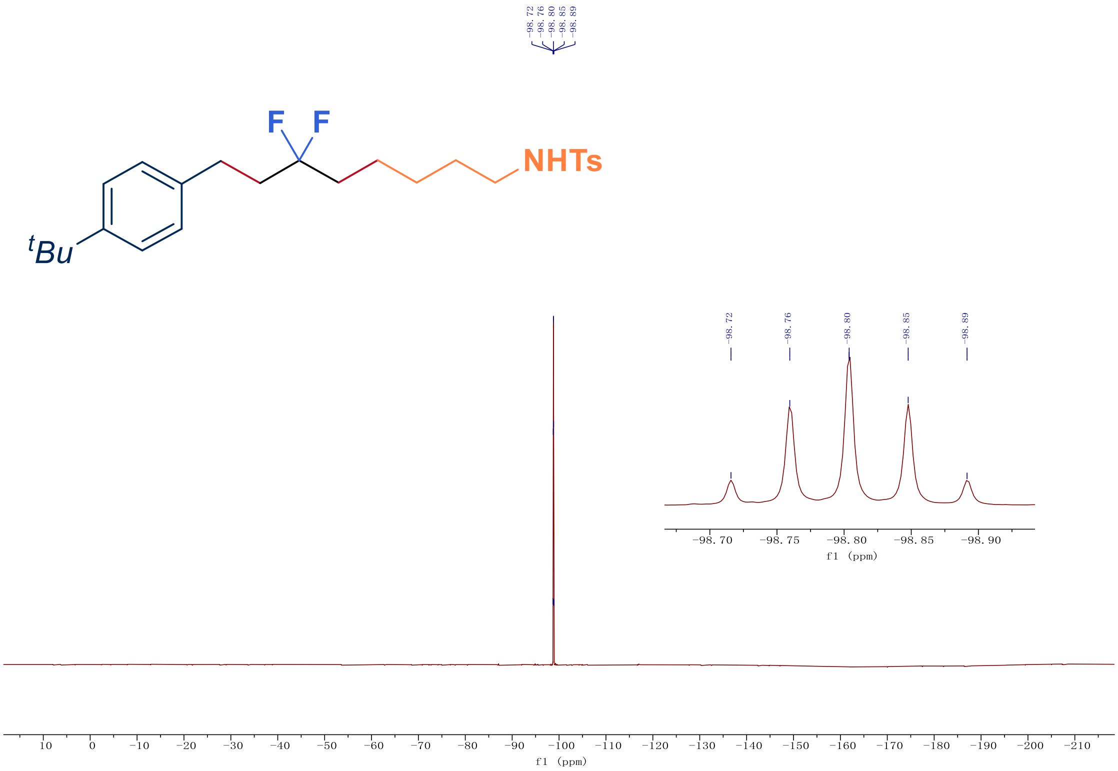

^{13}C NMR (126 MHz, $CDCl_3$) spectra for compound **2i**

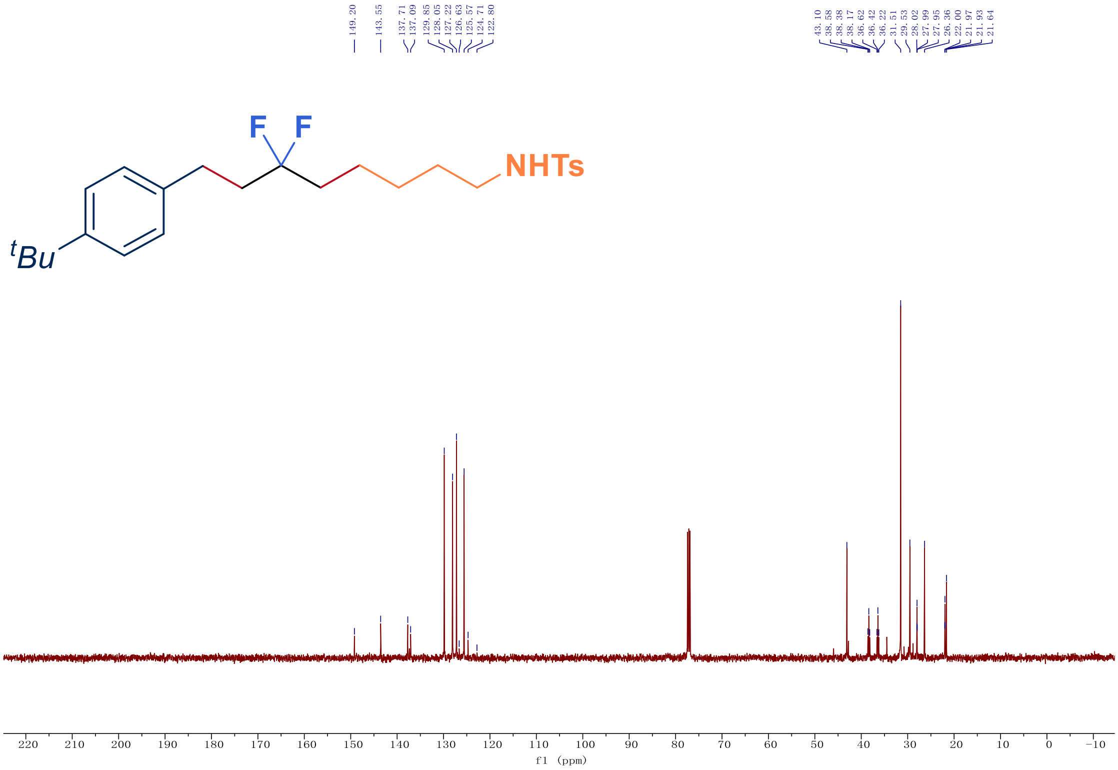

^{1}**H NMR** (400 MHz, $CDCl_3$) spectra for compound **2j**

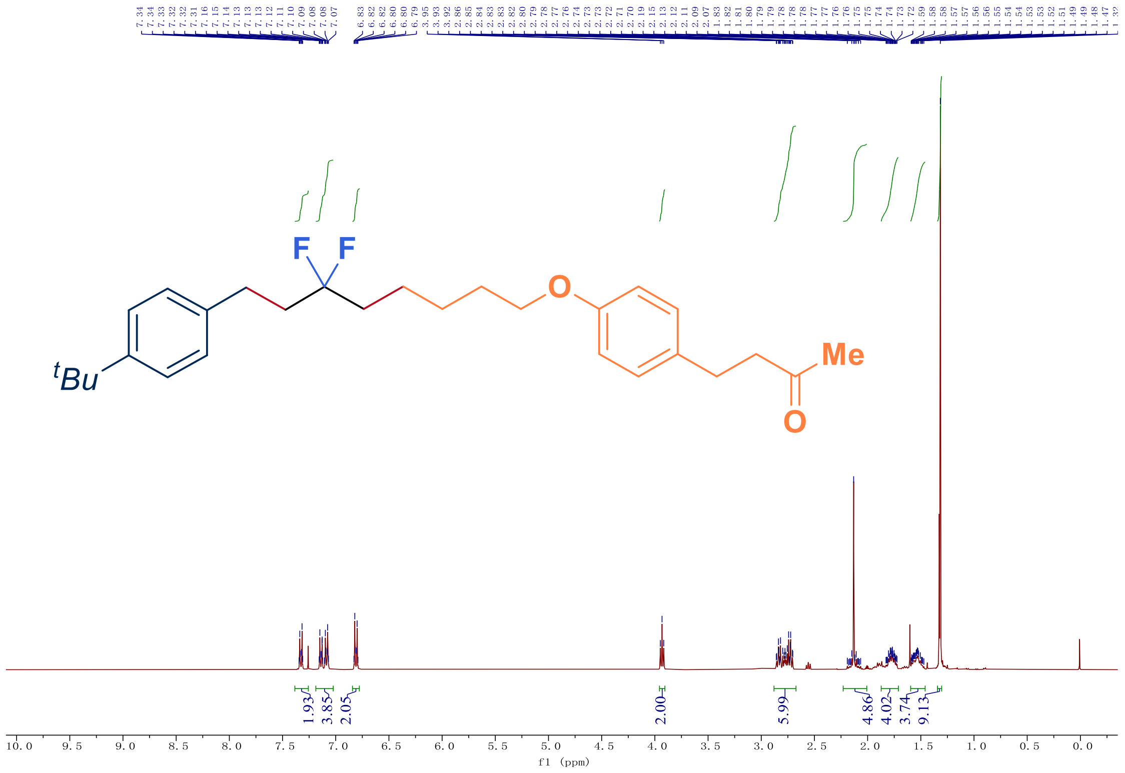

^{19}**F NMR** (377 MHz, $CDCl_3$) spectra for compound **2j**

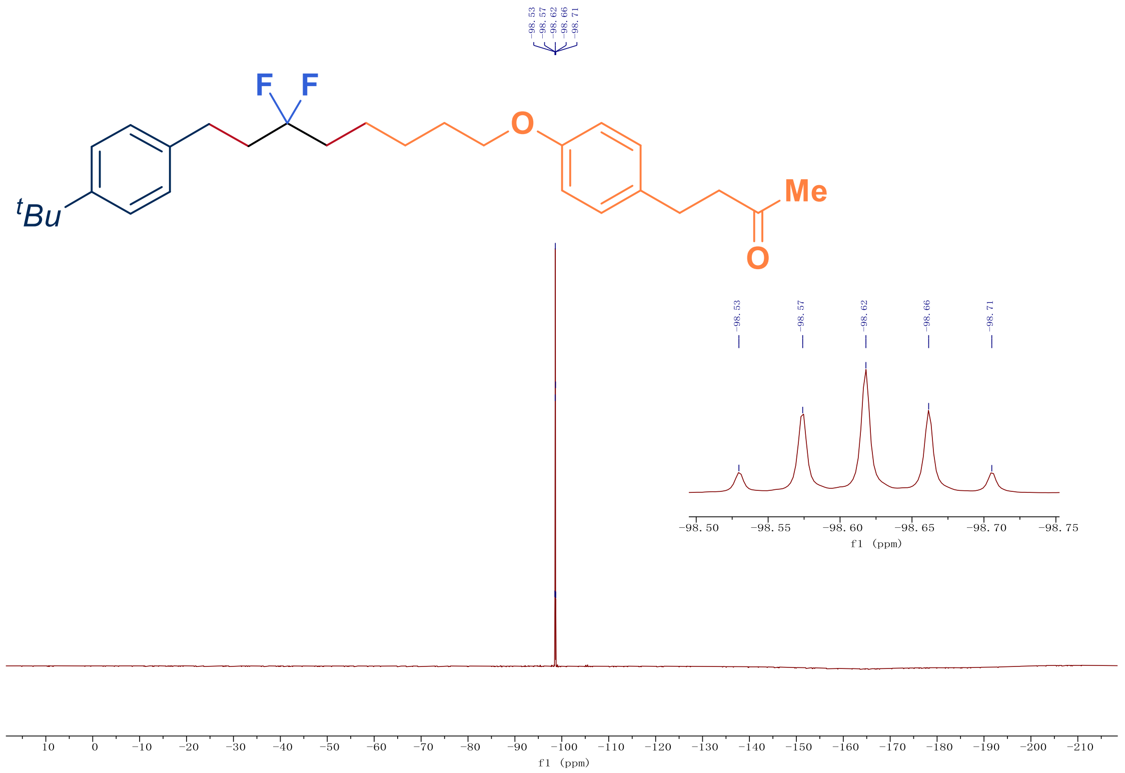

^{13}C NMR (126 MHz, $CDCl_3$) spectra for compound **2j**

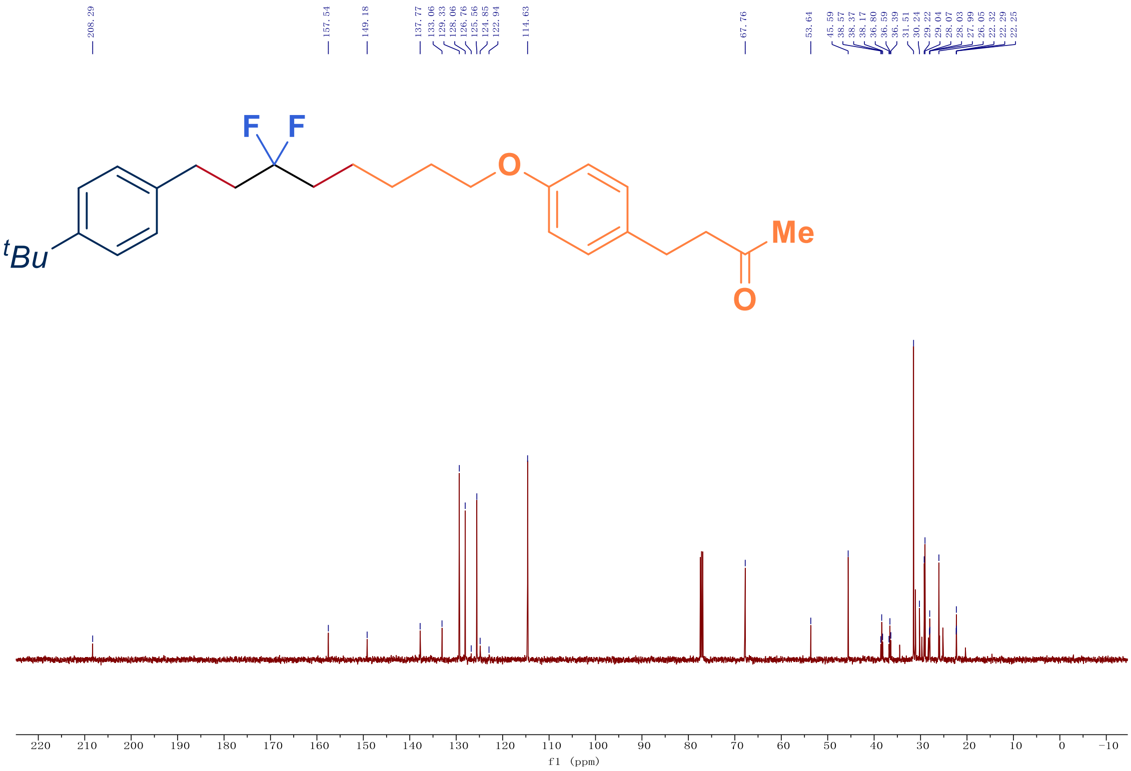

^{1}H NMR (400 MHz, $CDCl_3$) spectra for compound **2k**

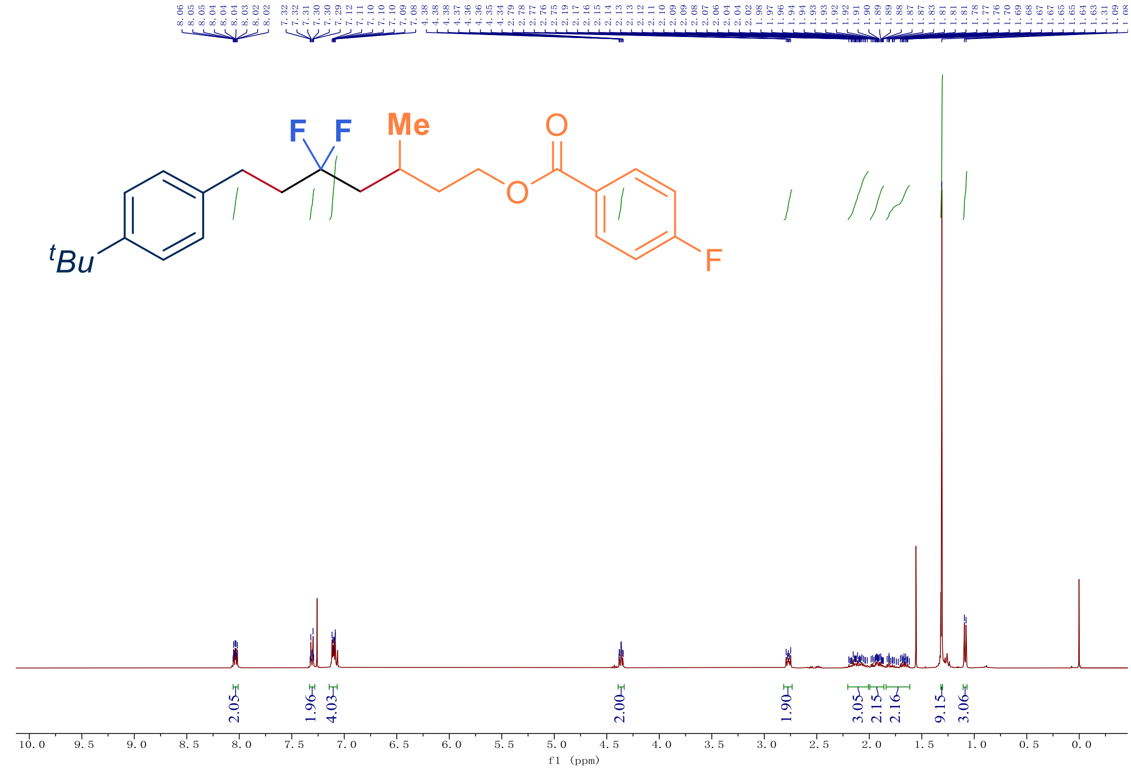

^{19}F NMR (377 MHz, $CDCl_3$) spectra for compound **2k**

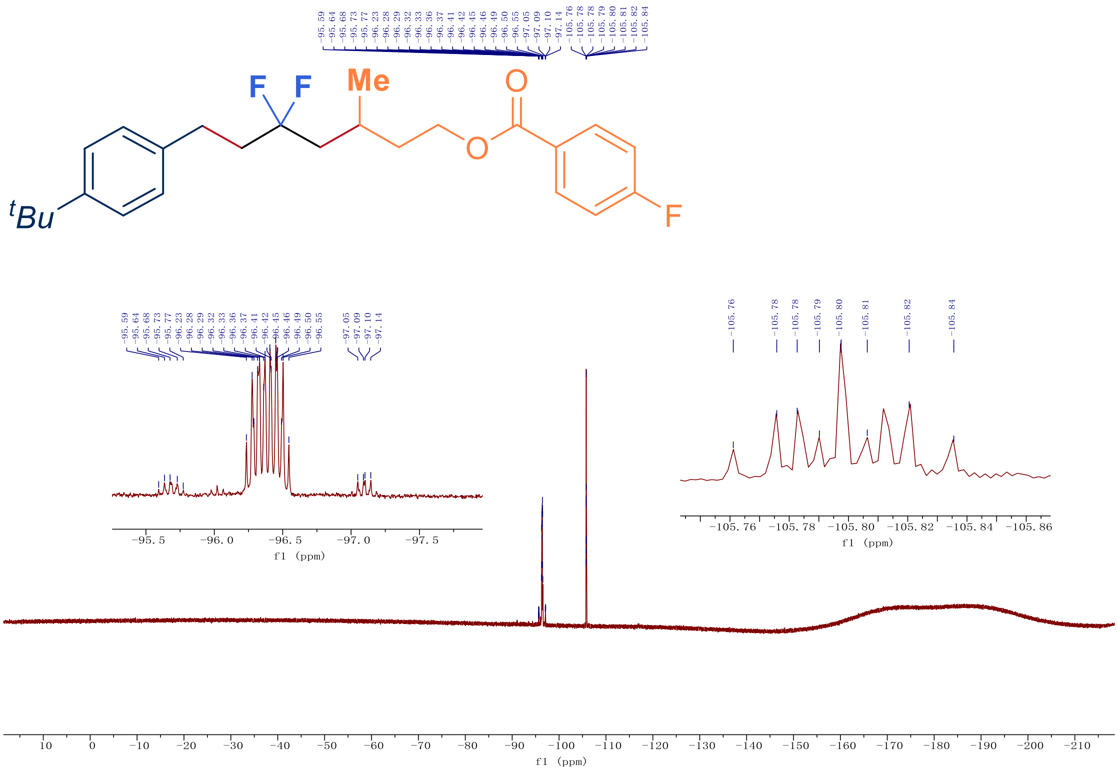

^{13}C NMR (126 MHz, $CDCl_3$) spectra for compound **2k**

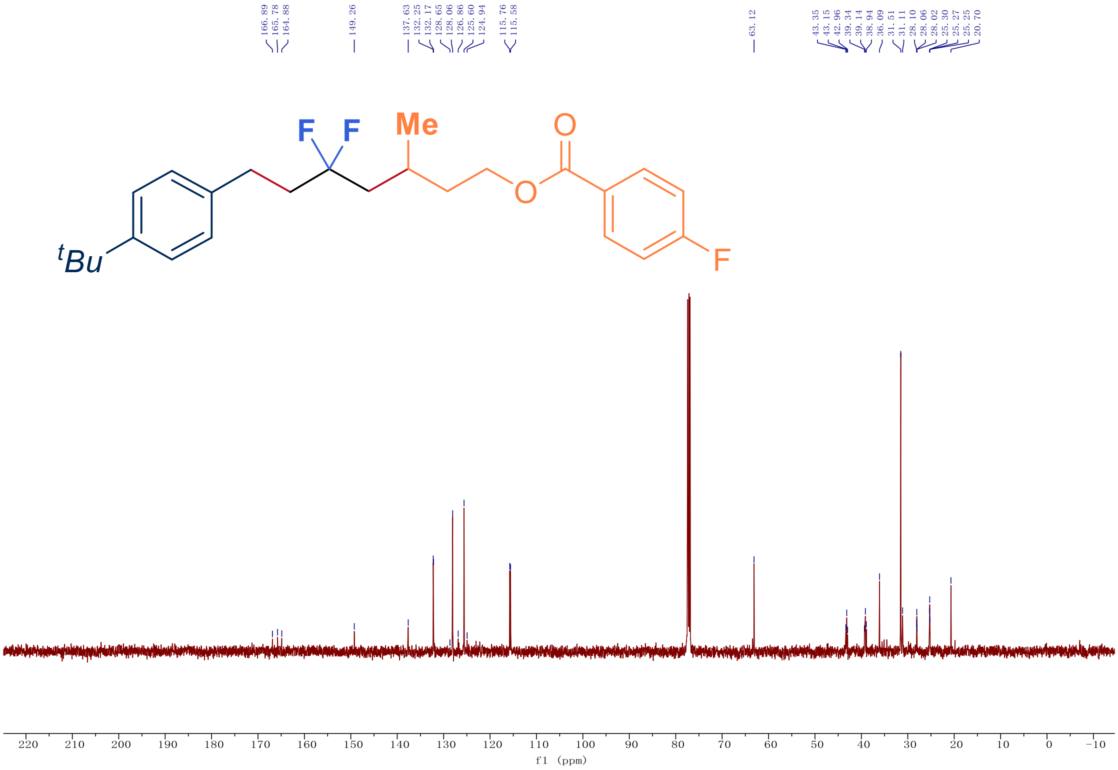

^{1}H NMR (400 MHz, $CDCl_3$) spectra for compound **2l**

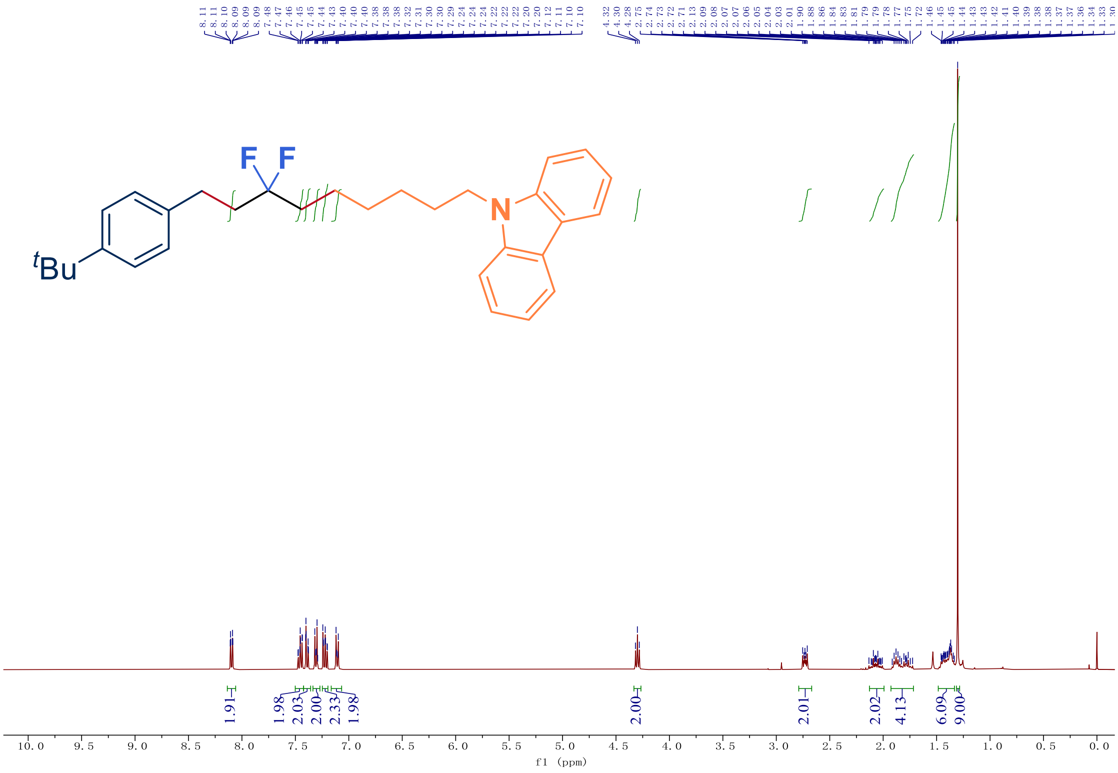

^{19}F NMR (377 MHz, $CDCl_3$) spectra for compound **2l**

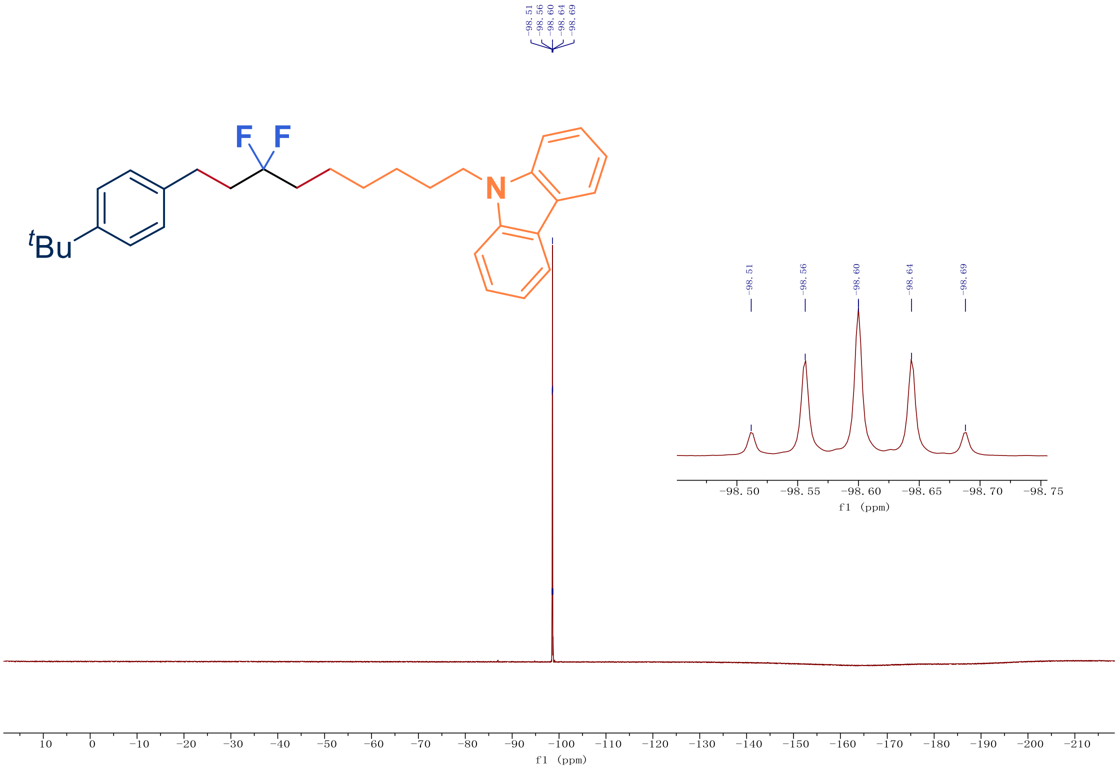

^{13}C NMR (126 MHz, $CDCl_3$) spectra for compound **2l**

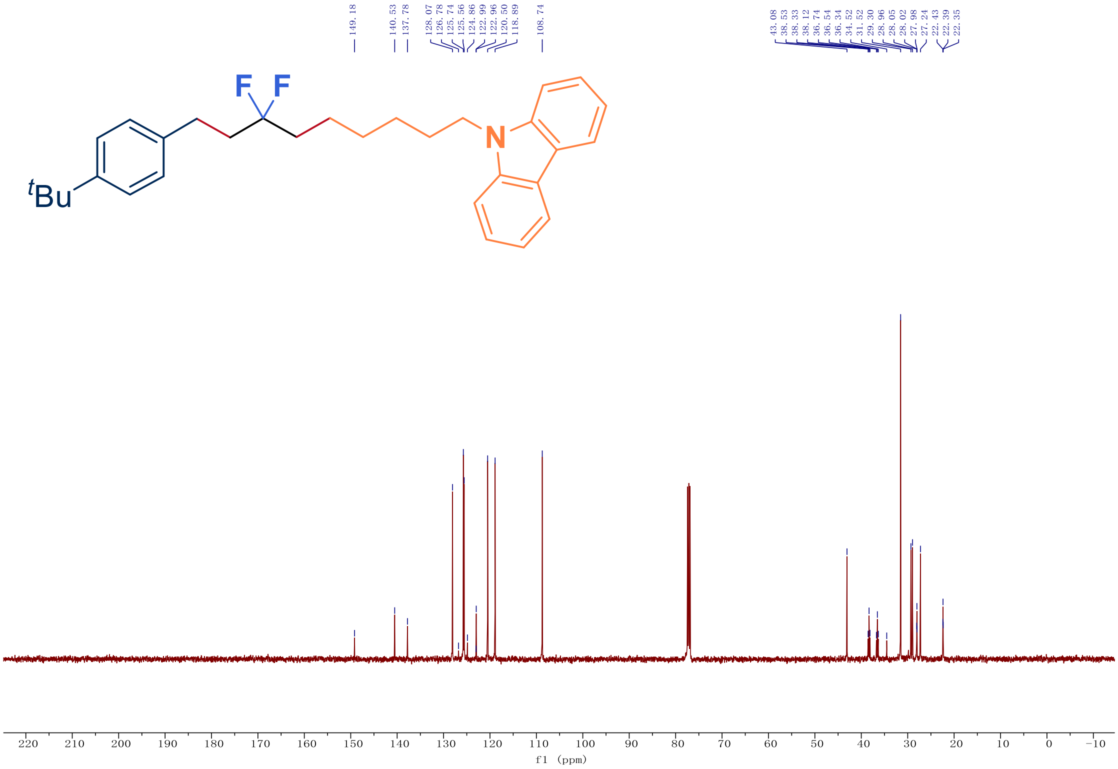

^{1}H NMR (400 MHz, $CDCl_3$) spectra for compound **2m**

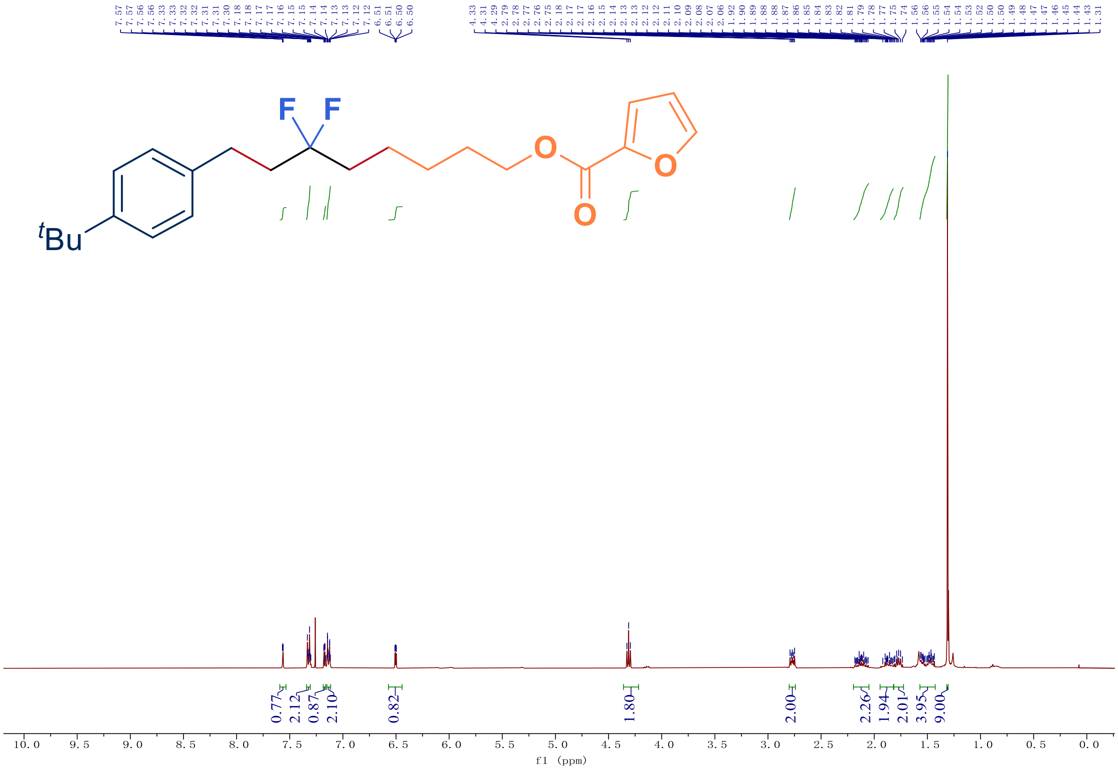

^{19}F NMR (377 MHz, $CDCl_3$) spectra for compound **2m**

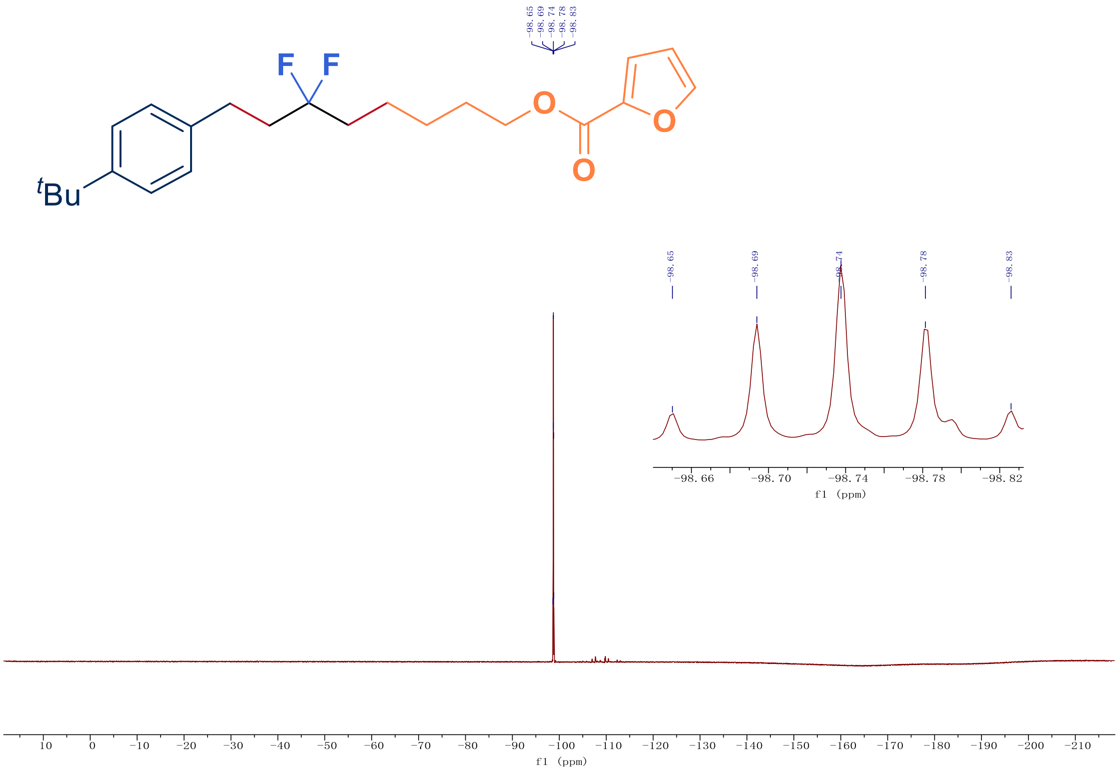

^{13}C NMR (126 MHz, $CDCl_3$) spectra for compound **2m**

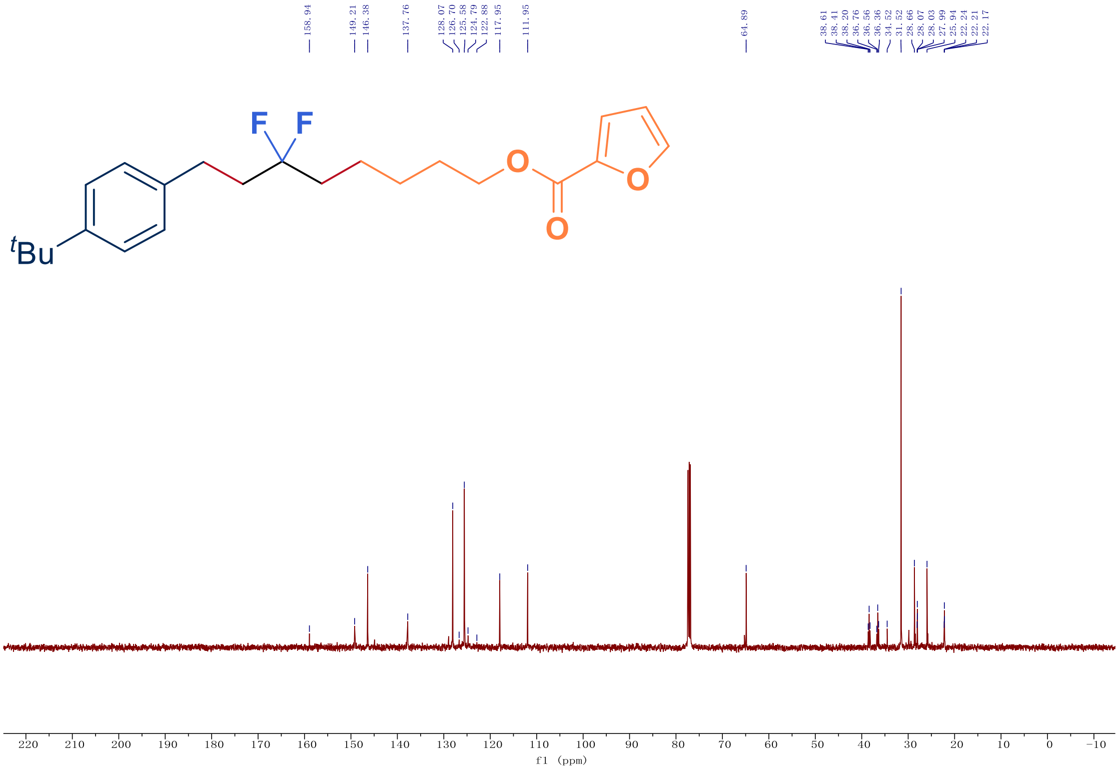

^{1}H NMR (400 MHz, $CDCl_3$) spectra for compound **2n**

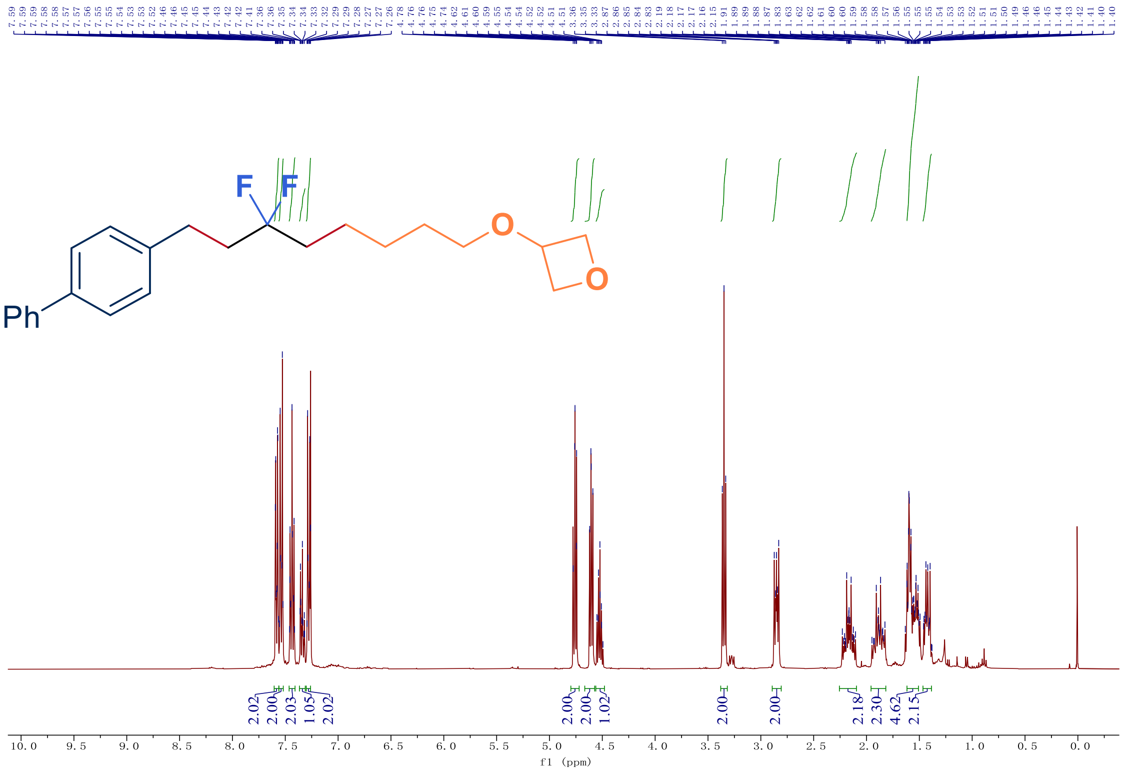

^{19}F NMR (377 MHz, $CDCl_3$) spectra for compound **2n**

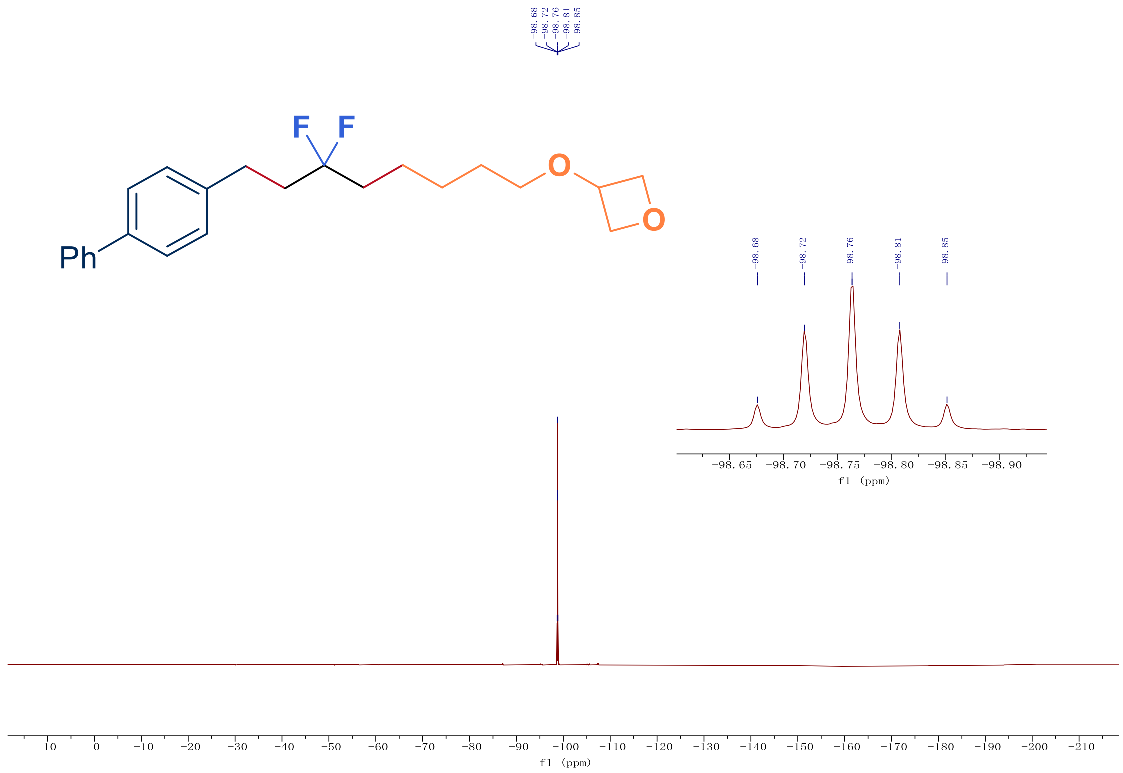

^{13}C NMR (126 MHz, $CDCl_3$) spectra for compound **2n**

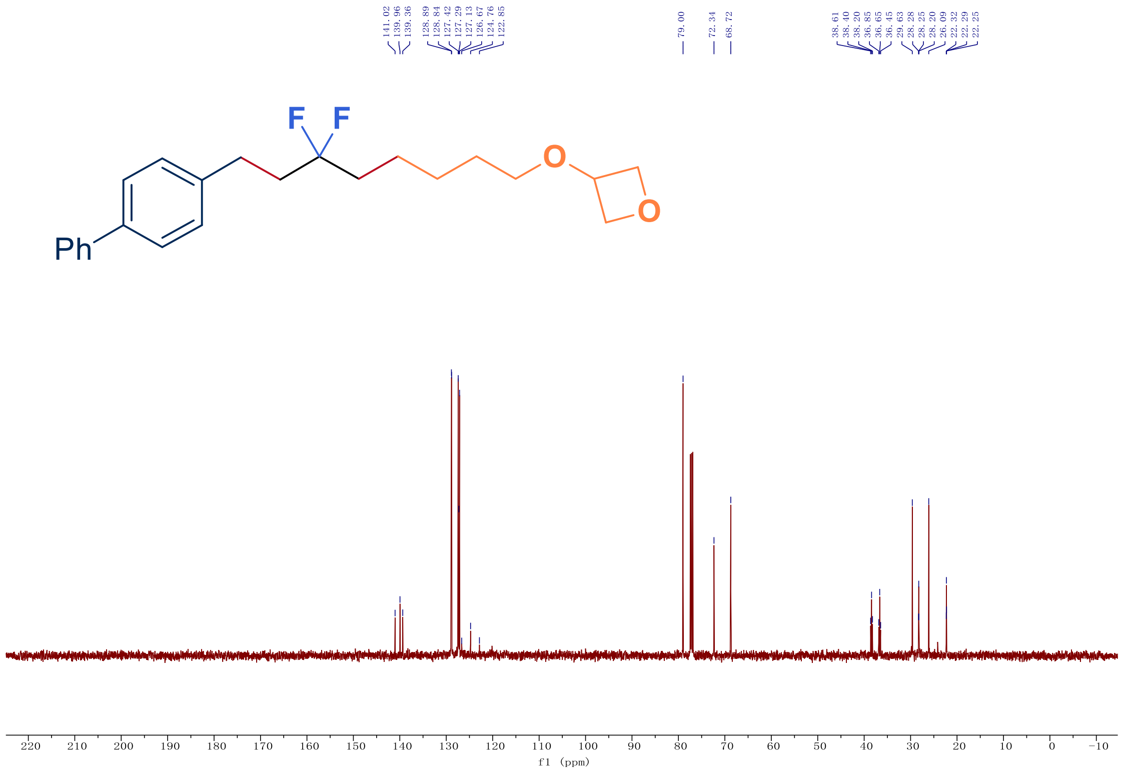

^{1}H NMR (400 MHz, $CDCl_3$) spectra for compound **2o**

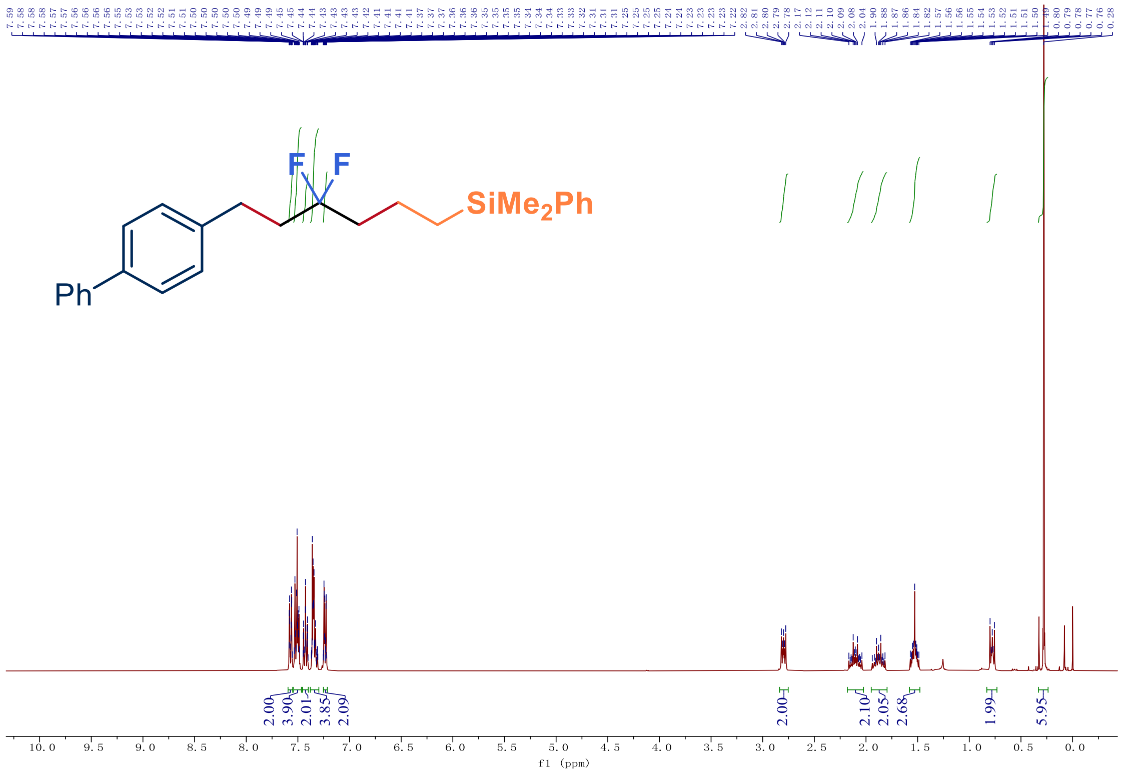

^{19}F NMR (377 MHz, $CDCl_3$) spectra for compound **2o**

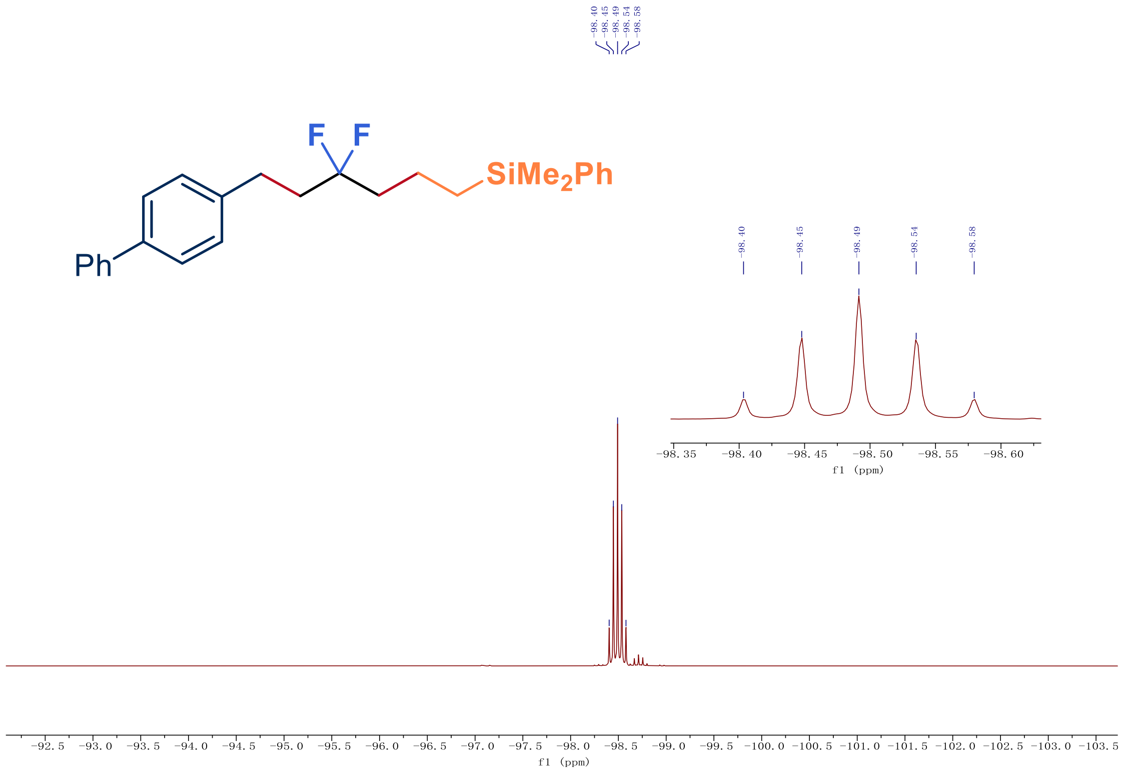

^{13}C NMR (126 MHz, $CDCl_3$) spectra for compound **2o**

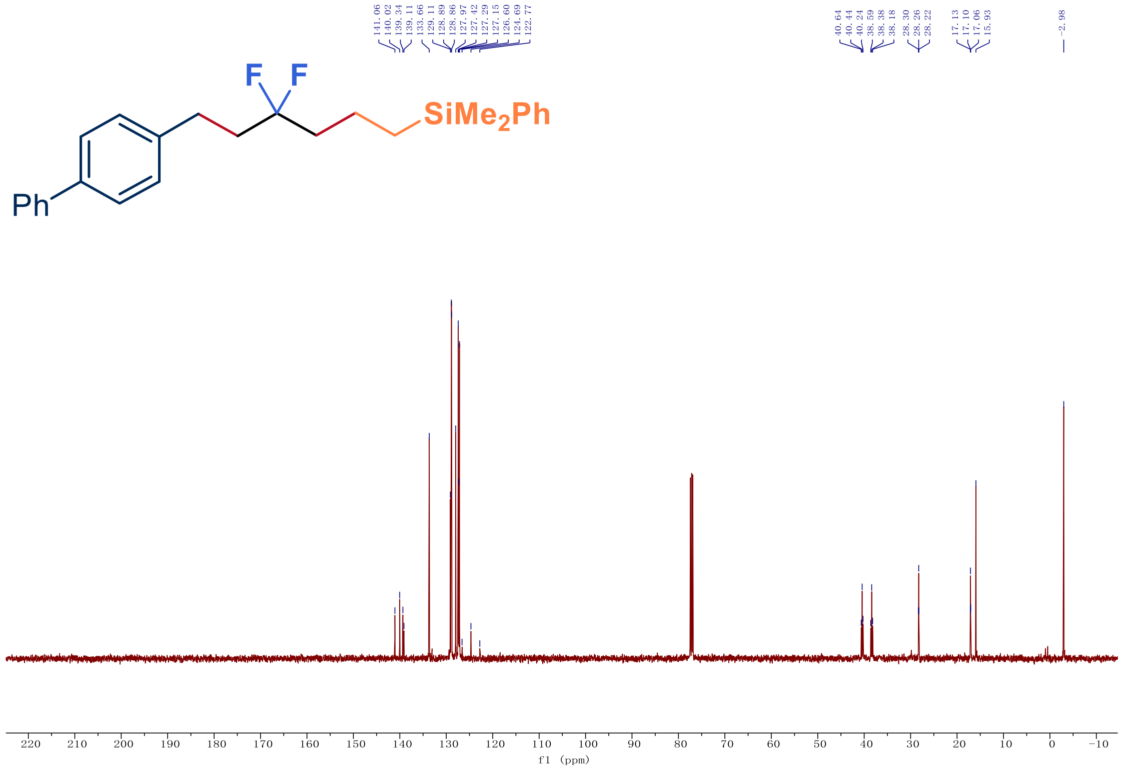

^{1}H NMR (400 MHz, $CDCl_3$) spectra for compound **2p**

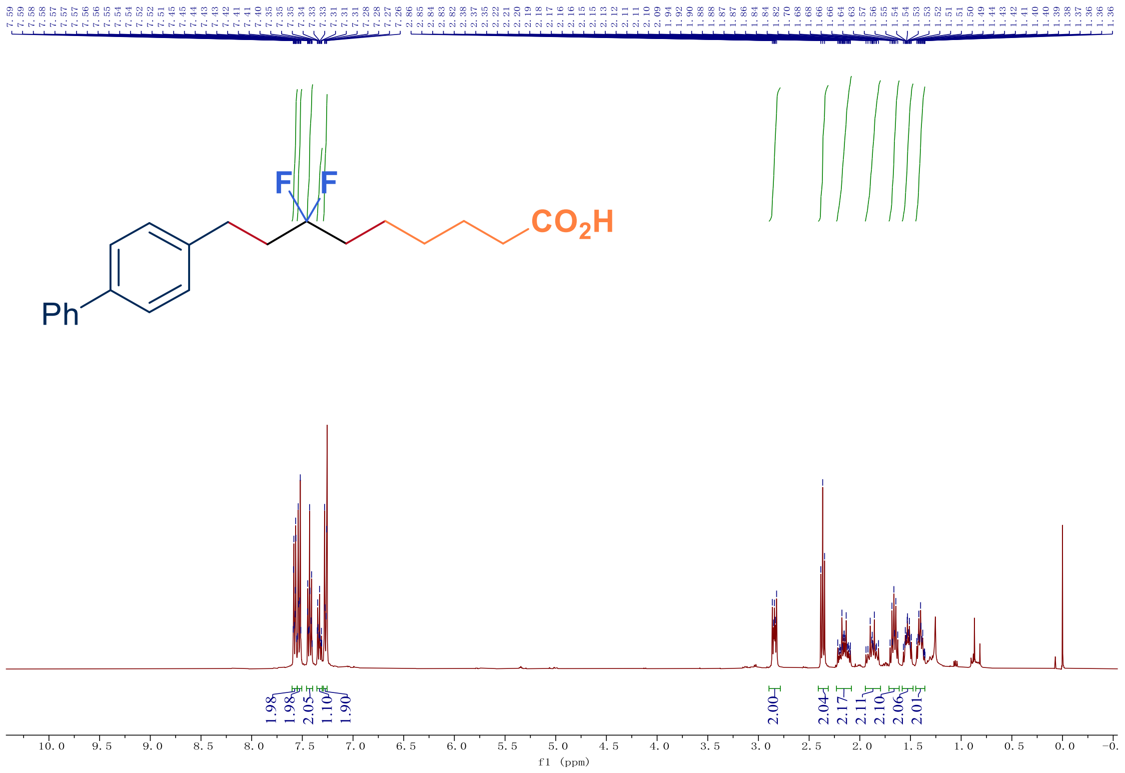

^{19}F NMR (377 MHz, $CDCl_3$) spectra for compound **2p**

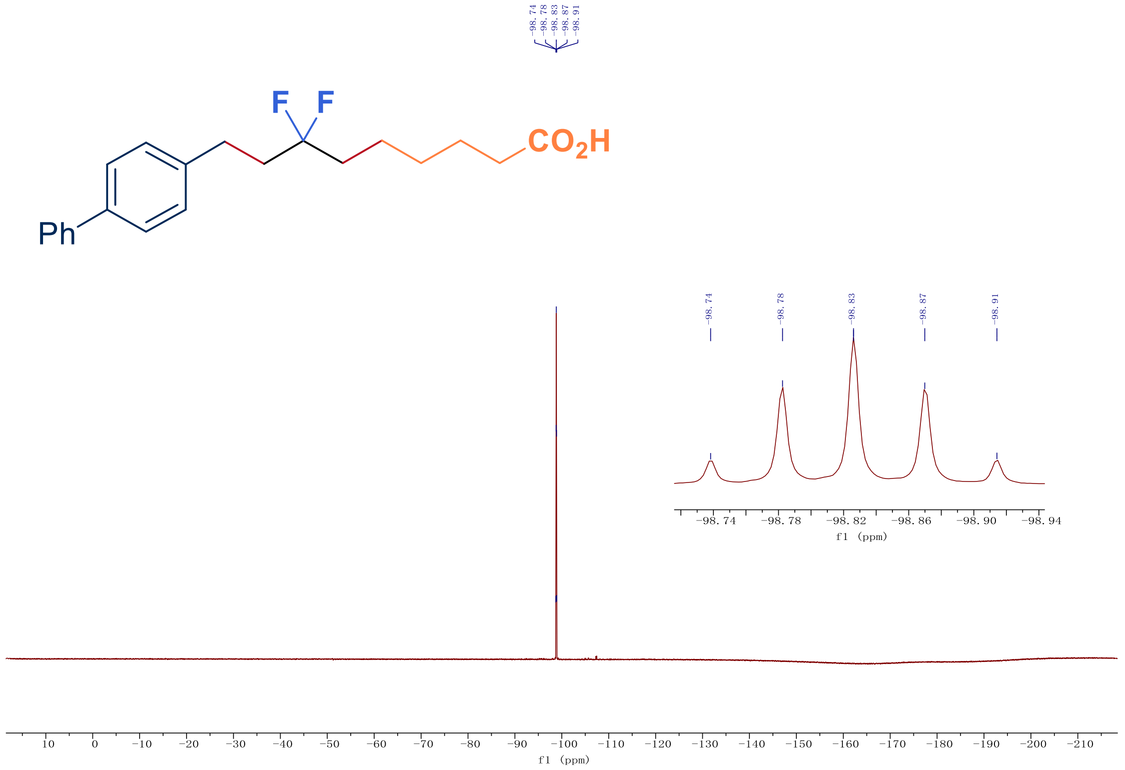

^{13}C NMR (126 MHz, $CDCl_3$) spectra for compound **2p**

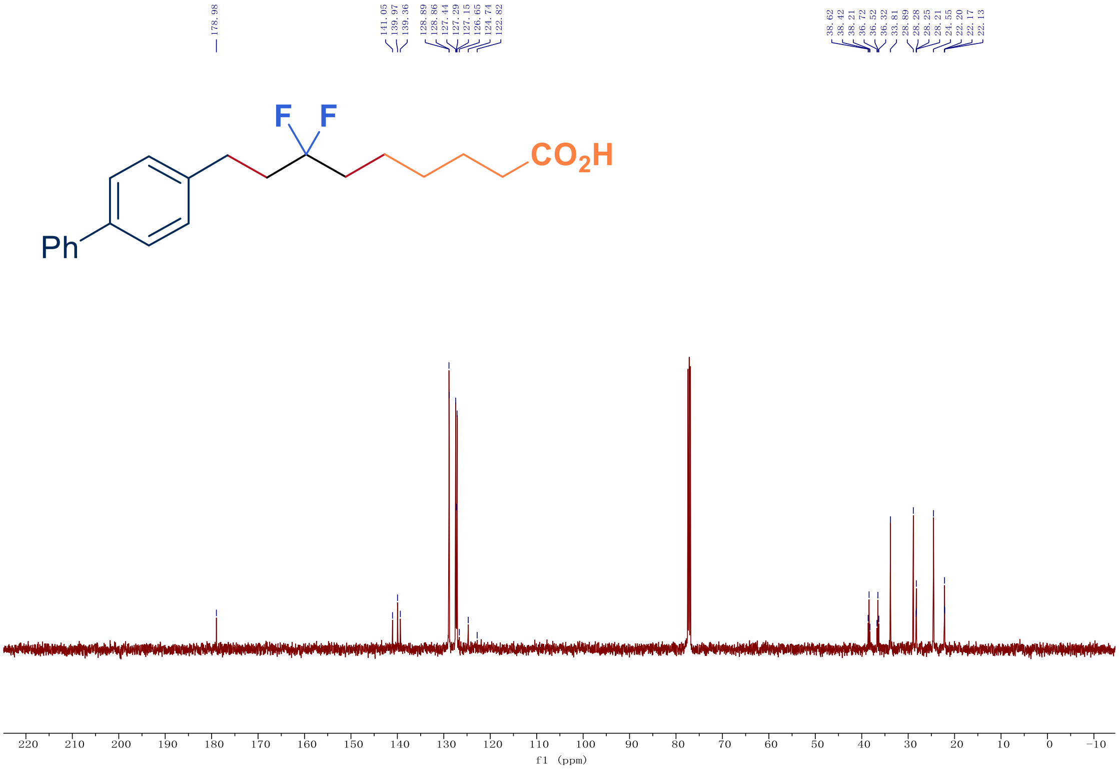

^{1}H NMR (400 MHz, $CDCl_3$) spectra for compound **2q**

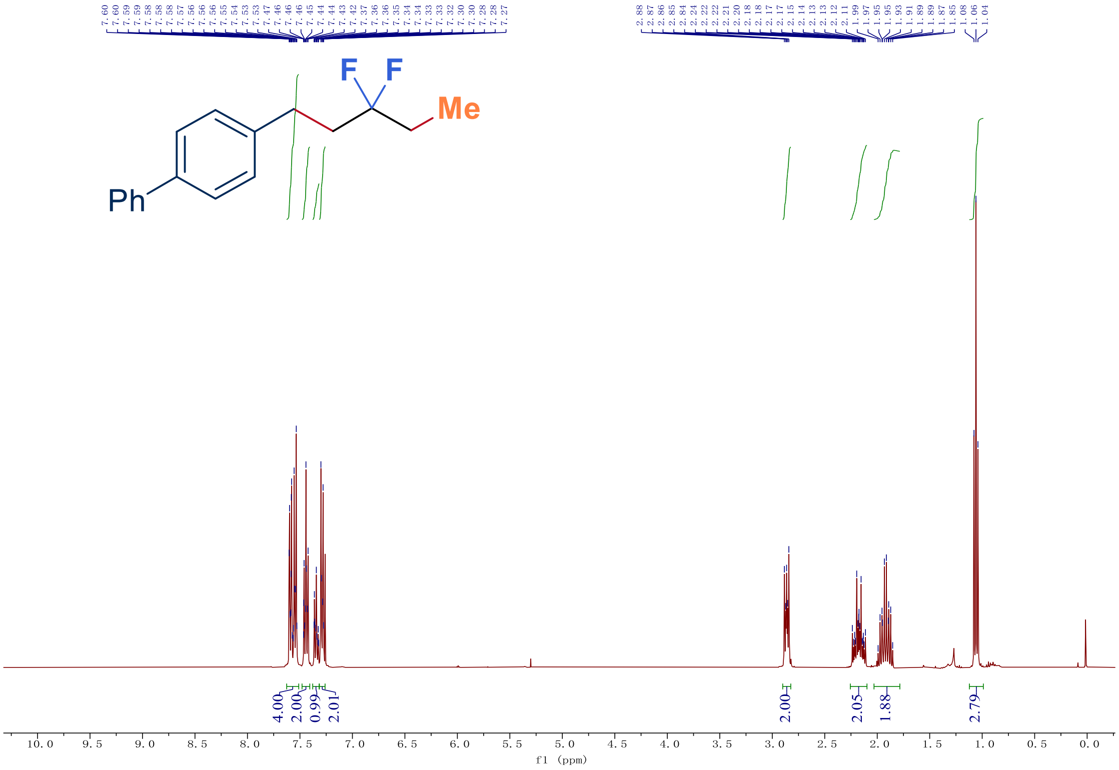

^{19}F NMR (377 MHz, $CDCl_3$) spectra for compound **2q**

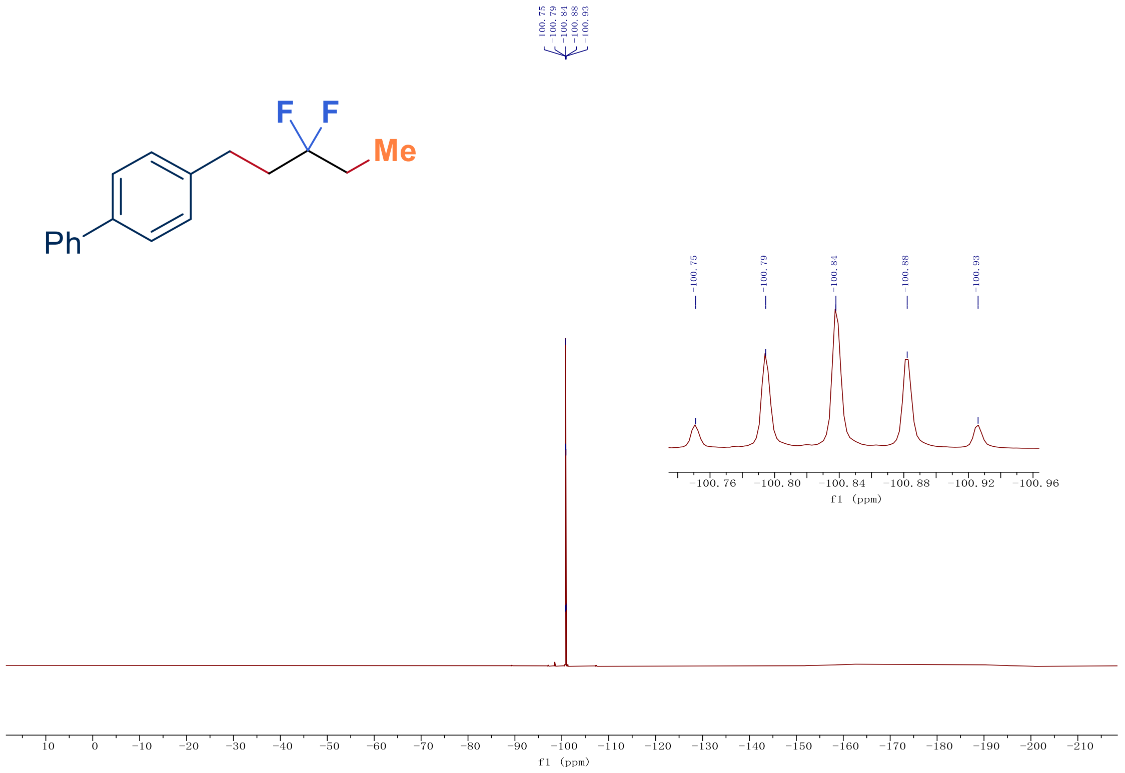

^{13}C NMR (126 MHz, $CDCl_3$) spectra for compound **2q**

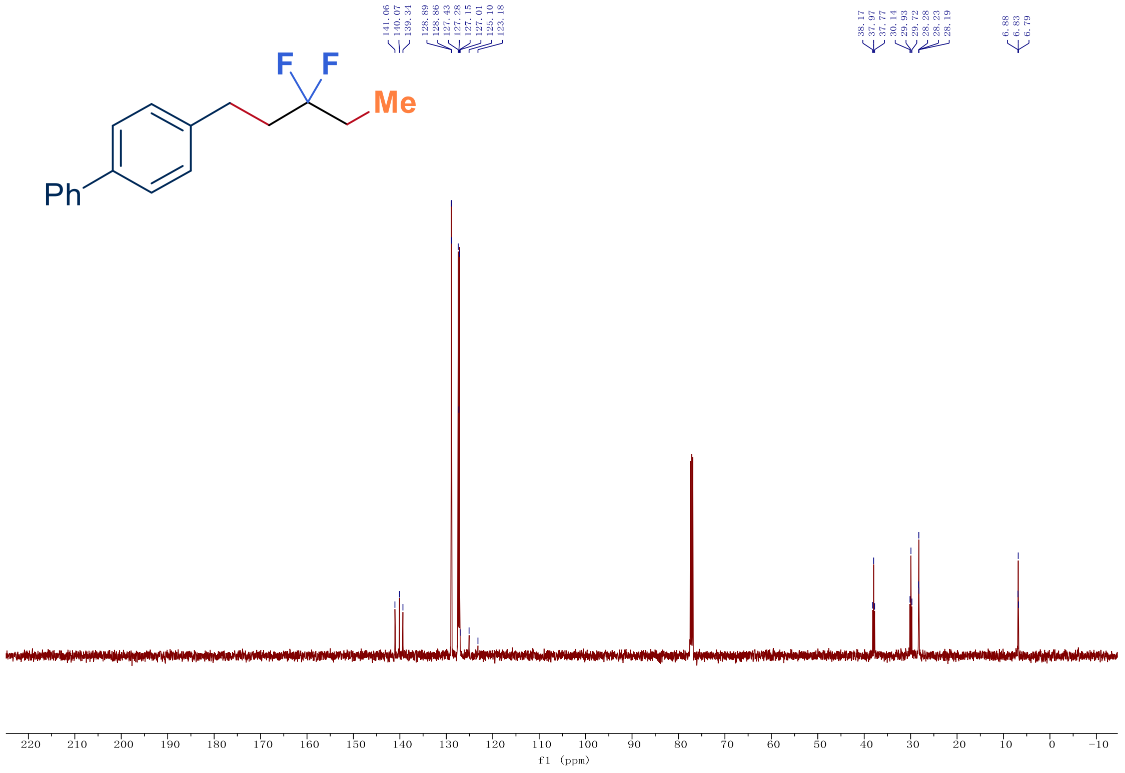

^{1}H NMR (400 MHz, $CDCl_3$) spectra for compound **2r**

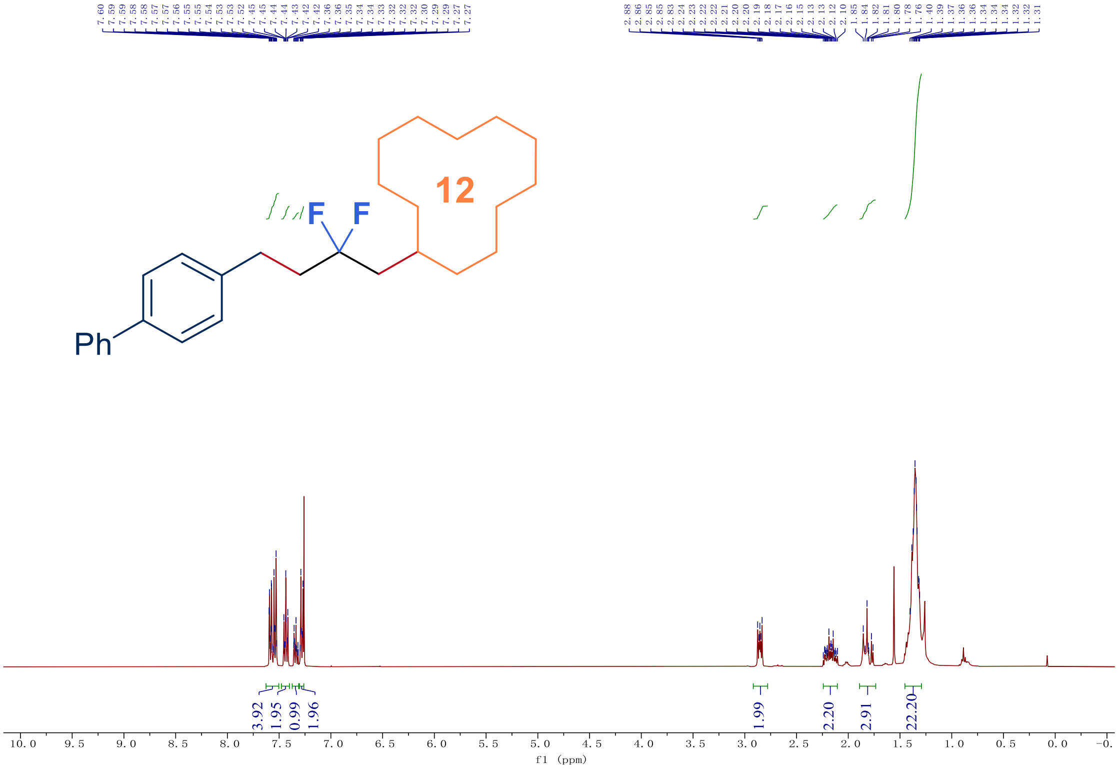

^{19}F NMR (377 MHz, $CDCl_3$) spectra for compound **2r**

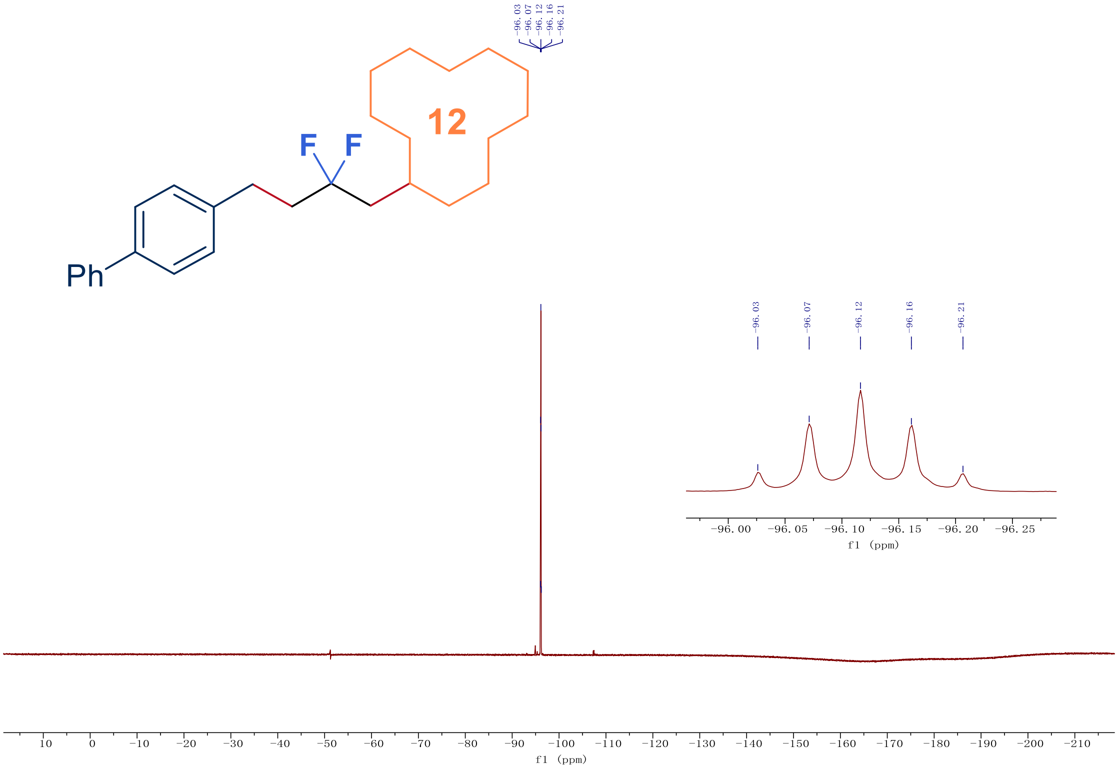

^{13}C NMR (126 MHz, $CDCl_3$) spectra for compound **2r**

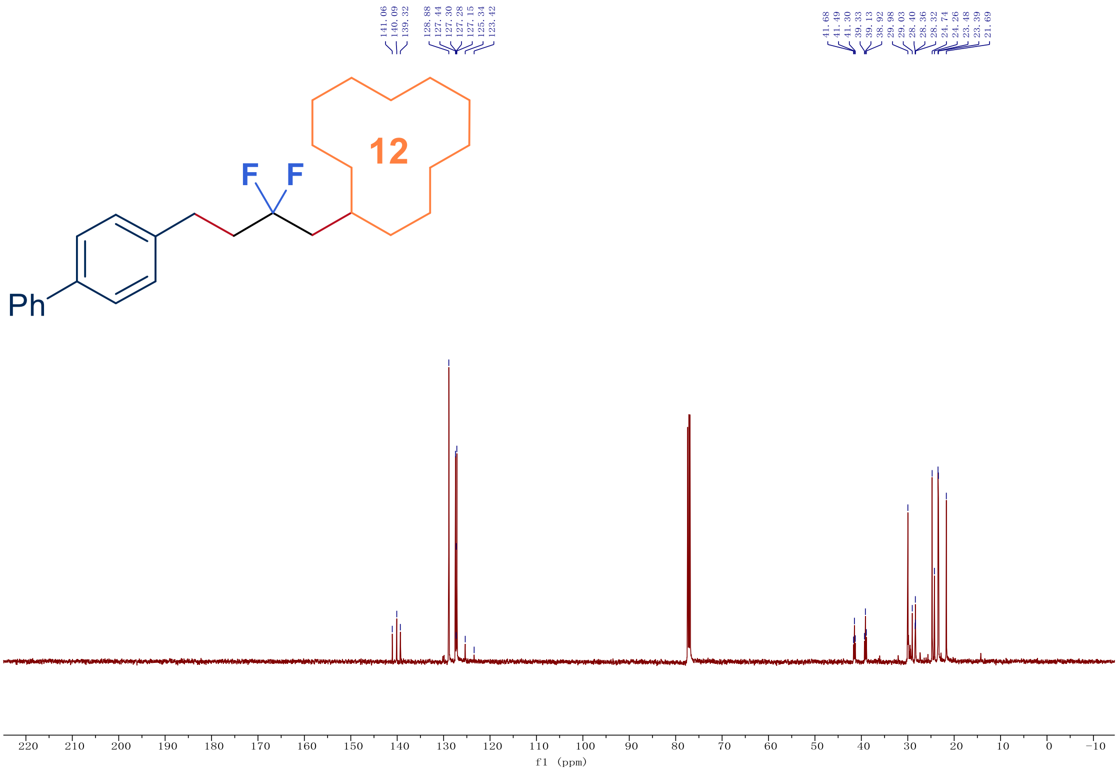

^{1}H NMR (400 MHz, $CDCl_3$) spectra for compound **2s**

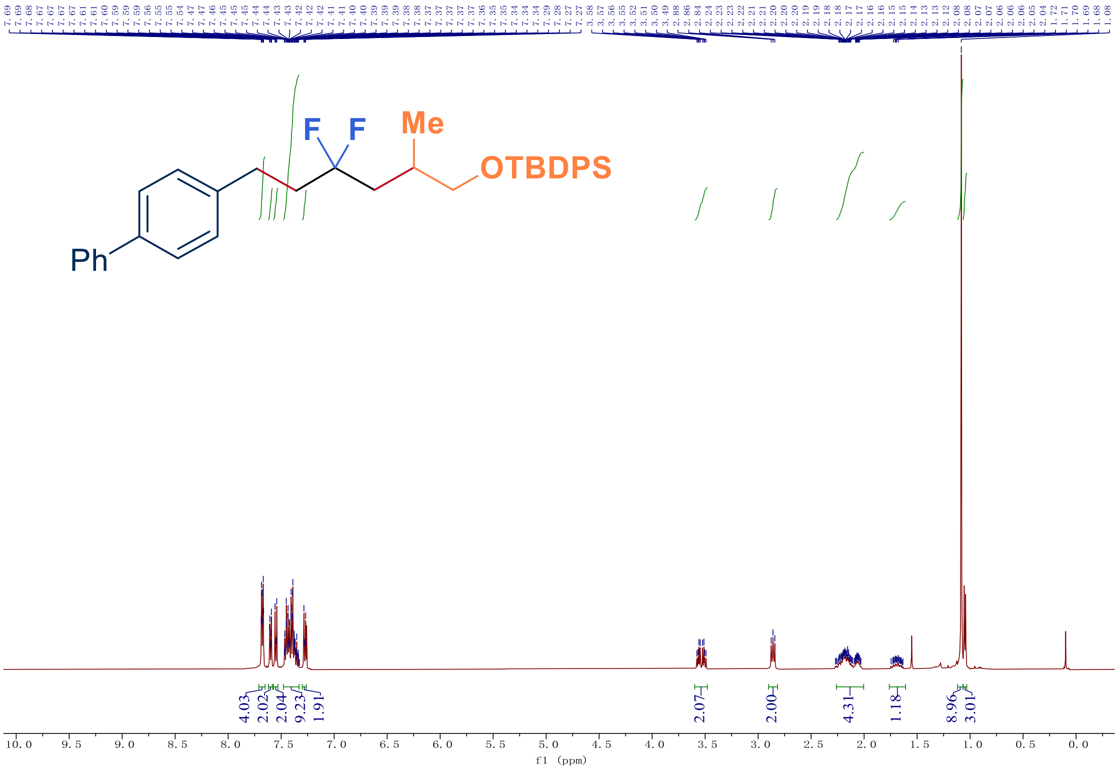

^{19}F NMR (377 MHz, $CDCl_3$) spectra for compound **2s**

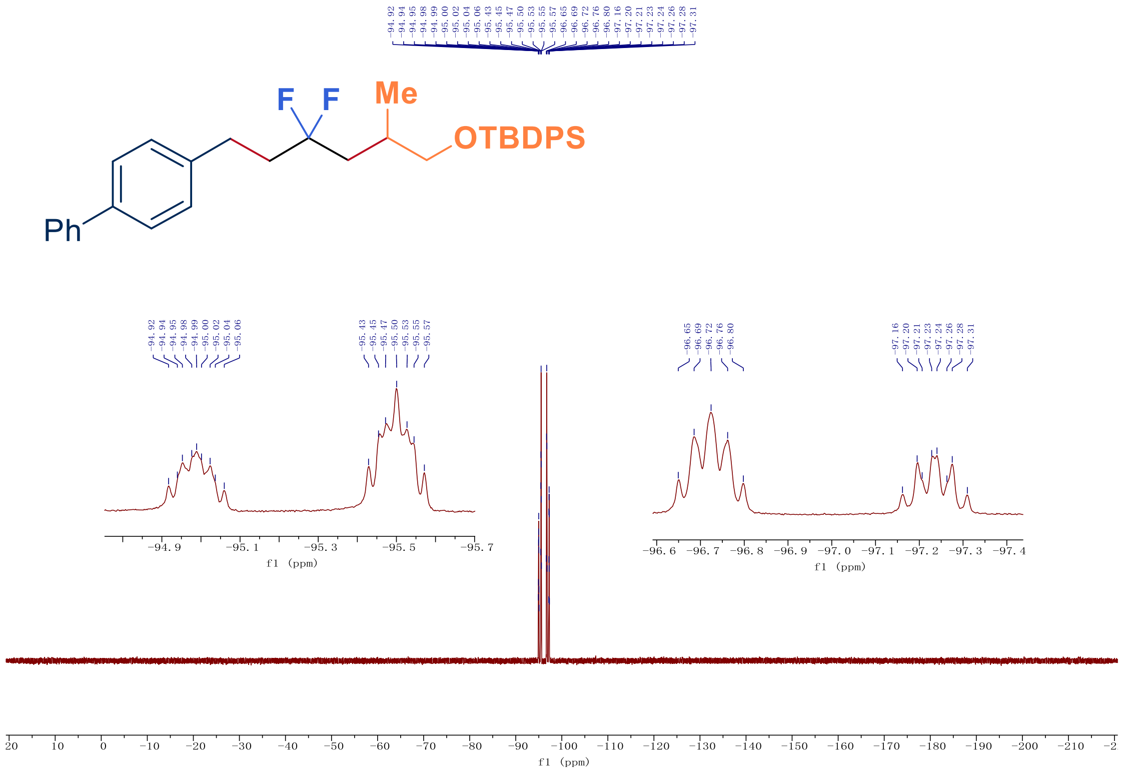

^{13}C NMR (126 MHz, $CDCl_3$) spectra for compound **2s**

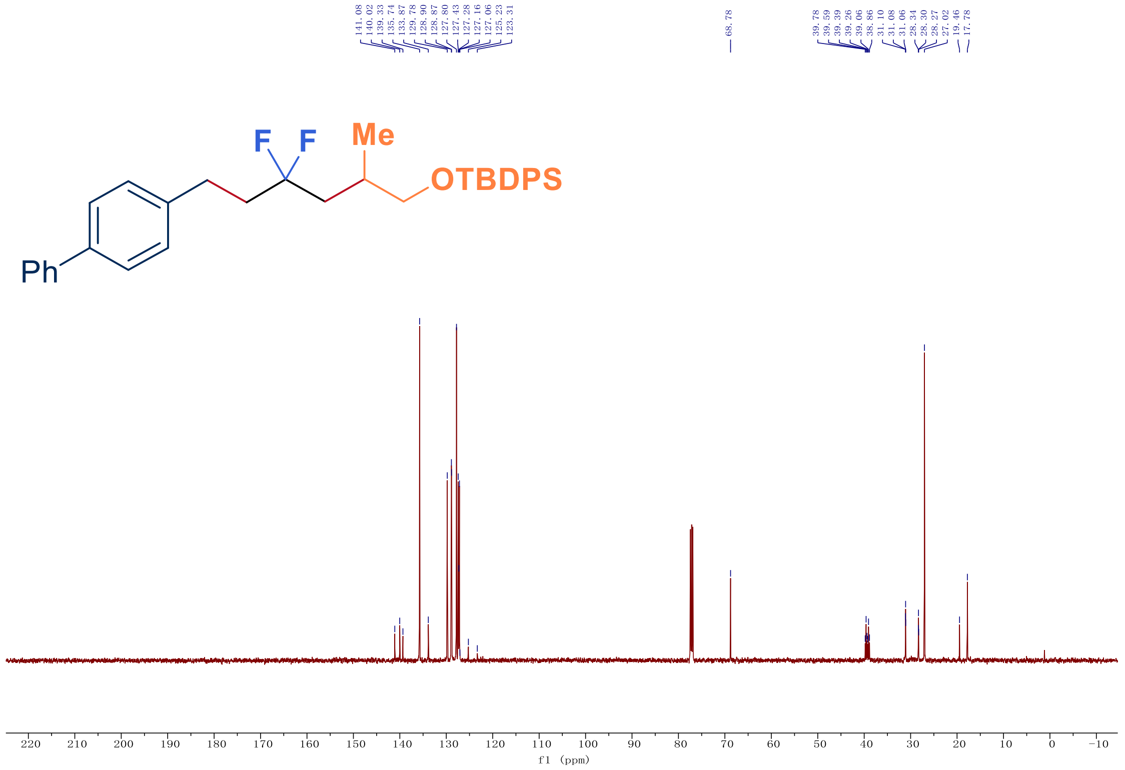

^{1}H NMR (400 MHz, $CDCl_3$) spectra for compound **2t**

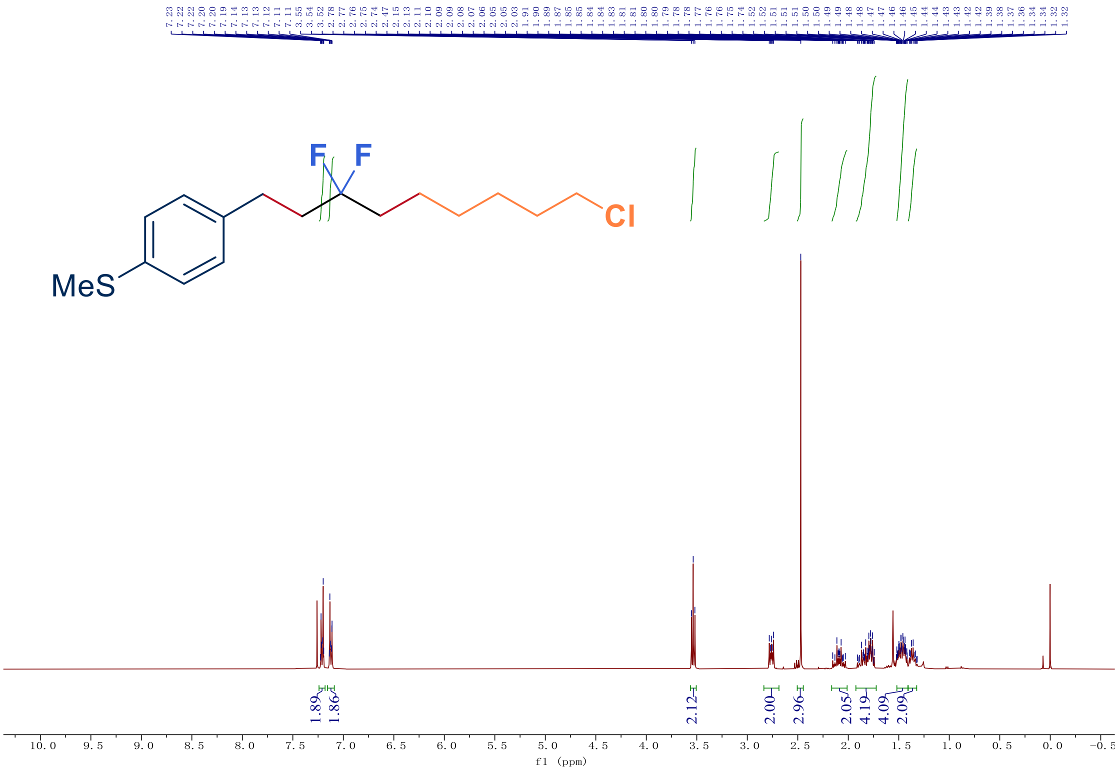

^{19}F NMR (377 MHz, $CDCl_3$) spectra for compound **2t**

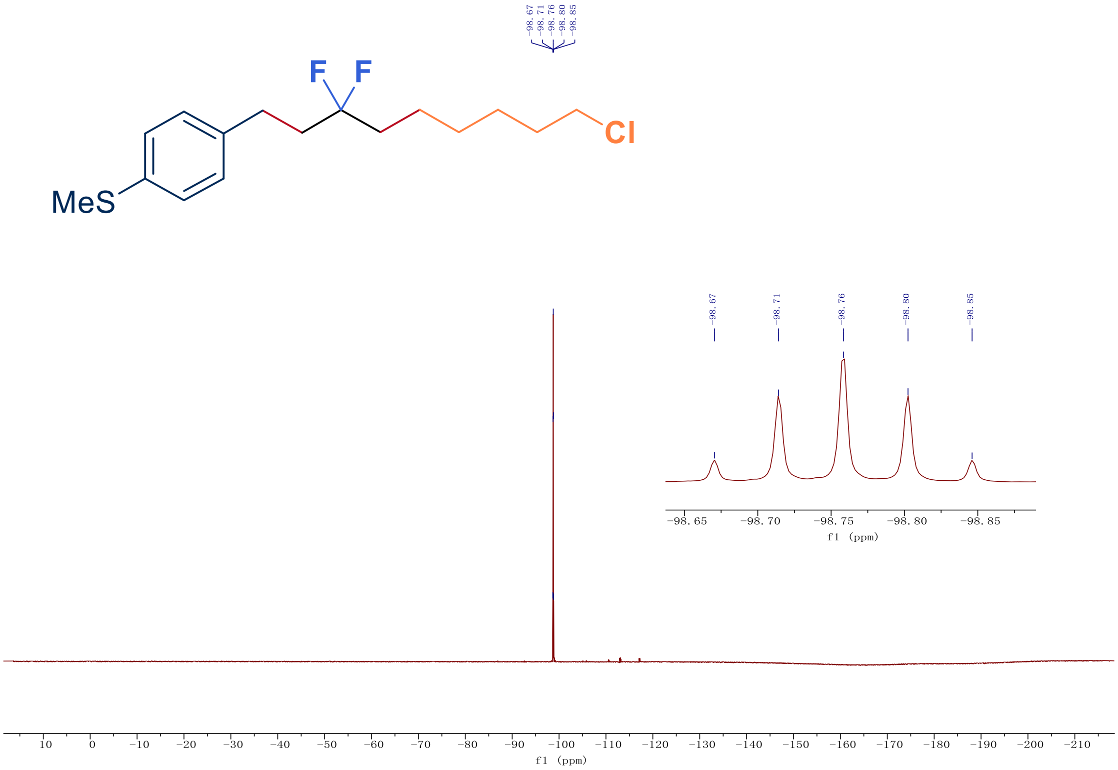

^{13}C NMR (126 MHz, $CDCl_3$) spectra for compound **2t**

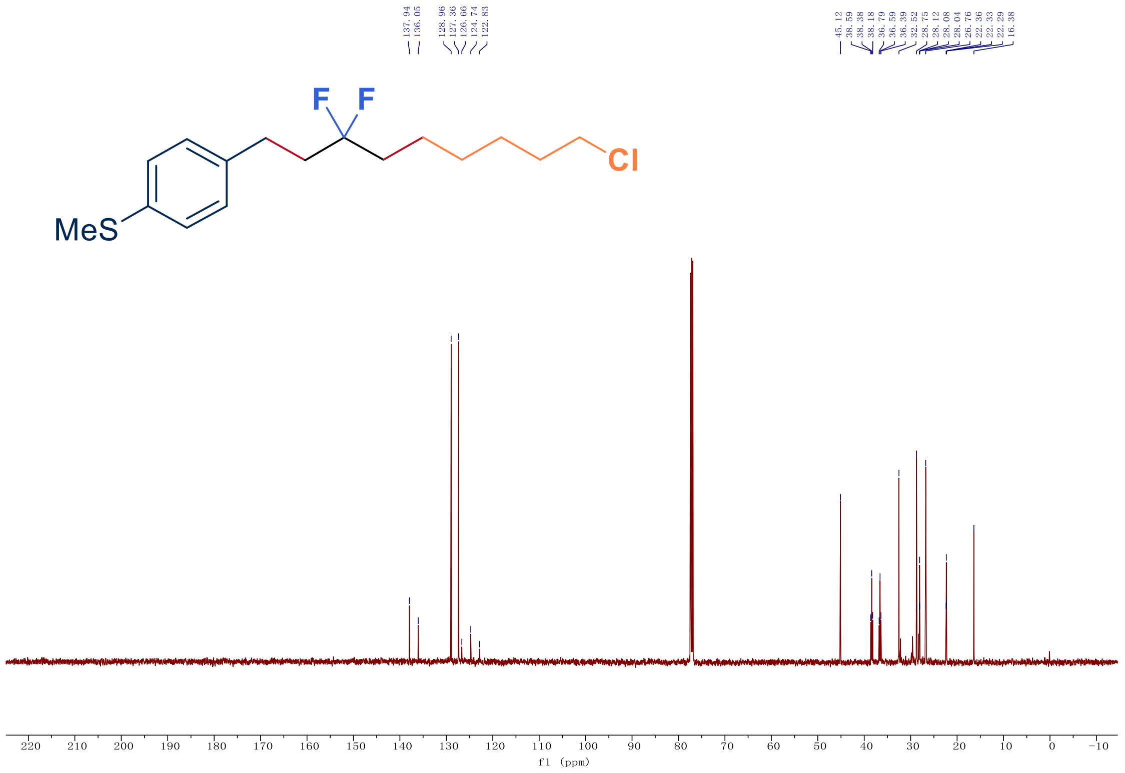

^{1}H NMR (400 MHz, $CDCl_3$) spectra for compound **2u**

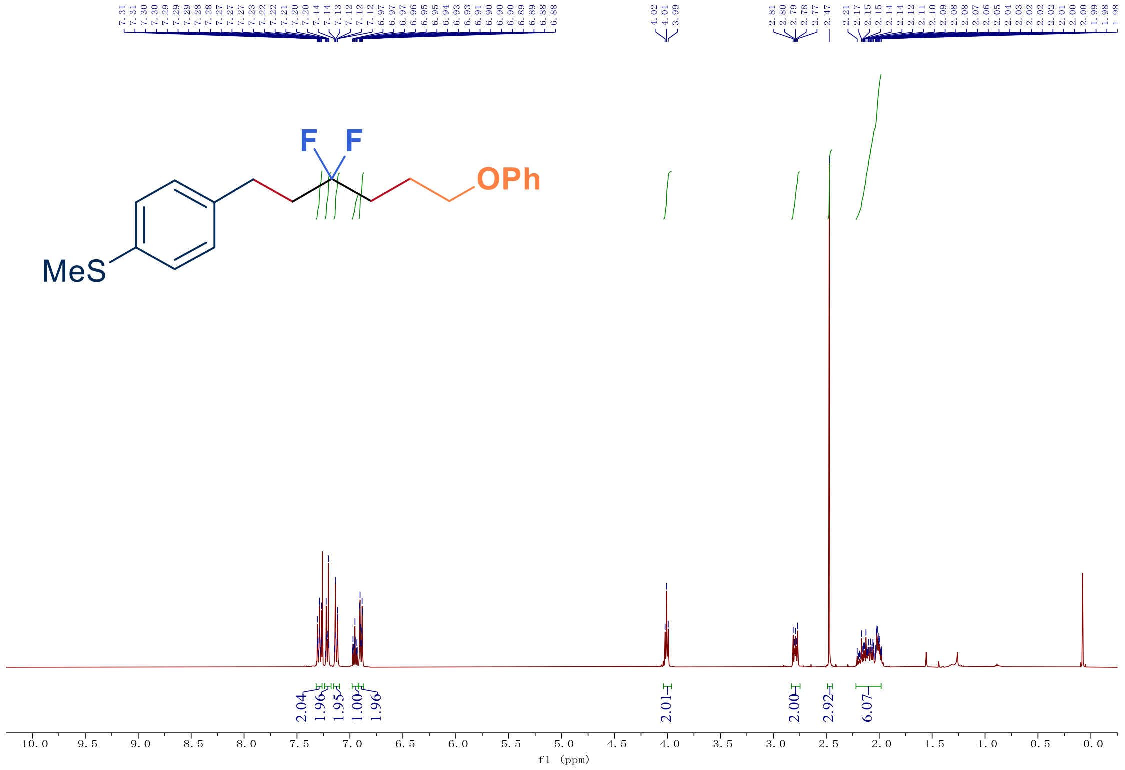

^{19}F NMR (377 MHz, $CDCl_3$) spectra for compound **2u**

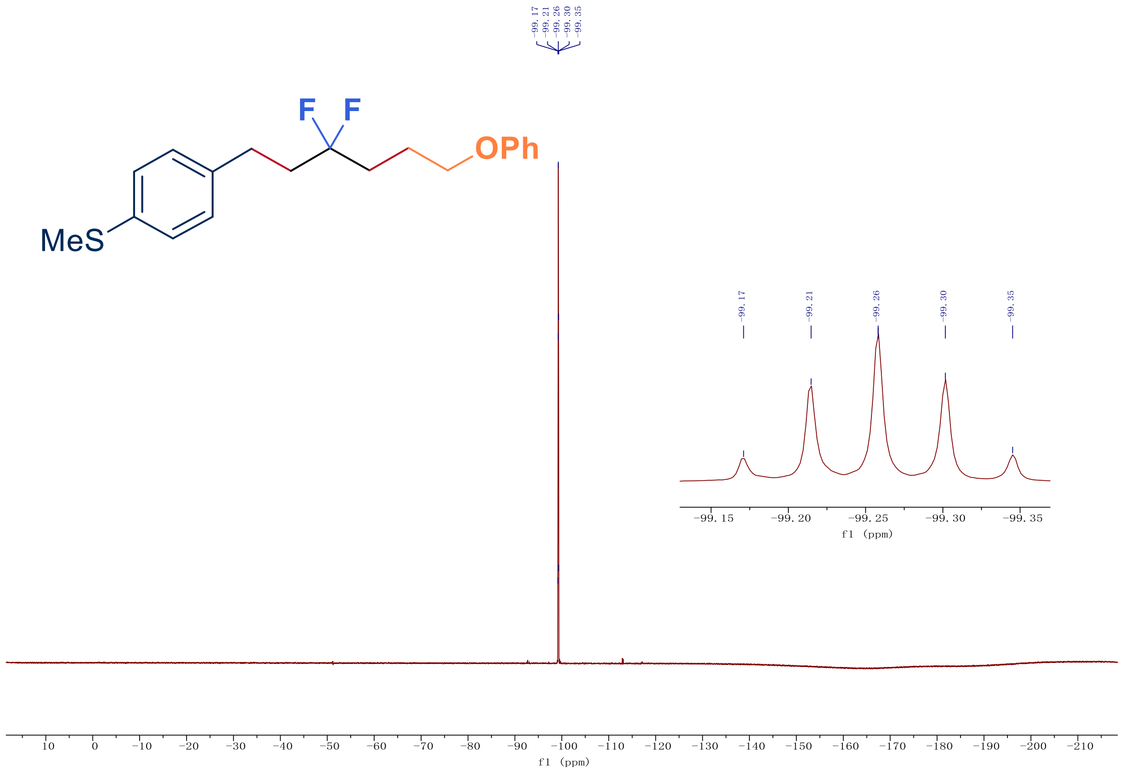

^{13}C NMR (126 MHz, $CDCl_3$) spectra for compound **2u**

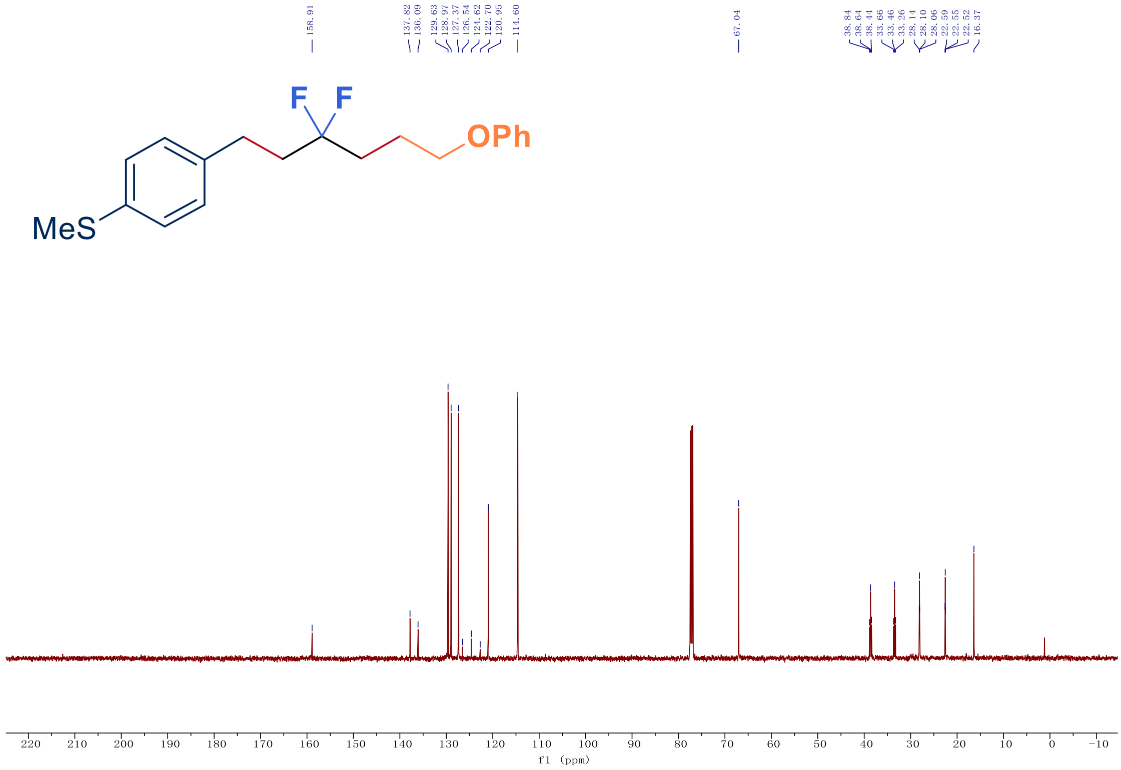

^{1}H NMR (400 MHz, $CDCl_3$) spectra for compound **2v**

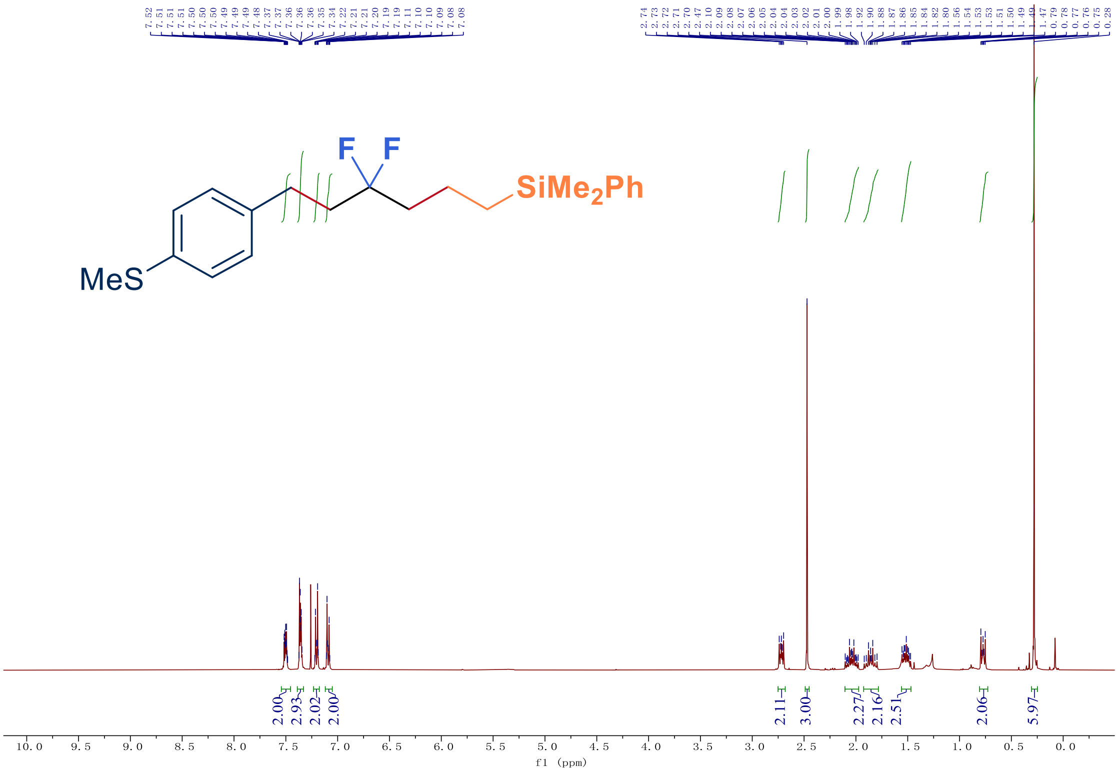

^{19}F NMR (377 MHz, $CDCl_3$) spectra for compound **2v**

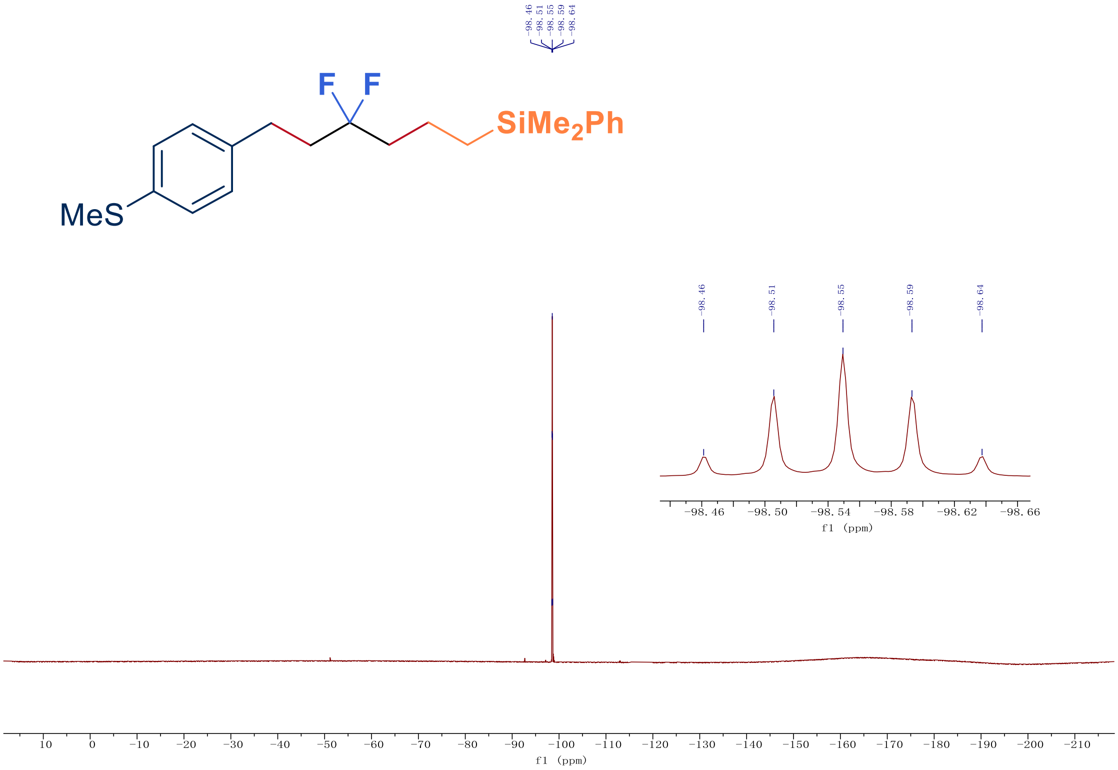

^{13}C NMR (126 MHz, $CDCl_3$) spectra for compound **2v**

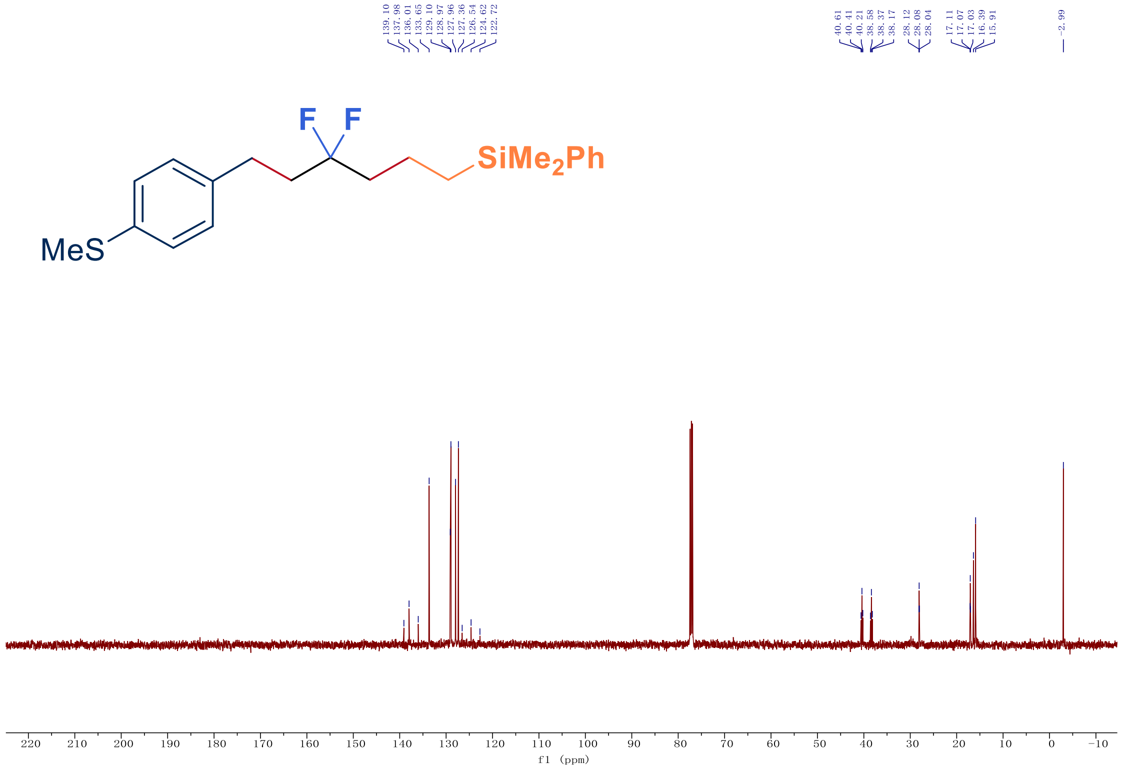

^{1}H NMR (400 MHz, $CDCl_3$) spectra for compound **2w**

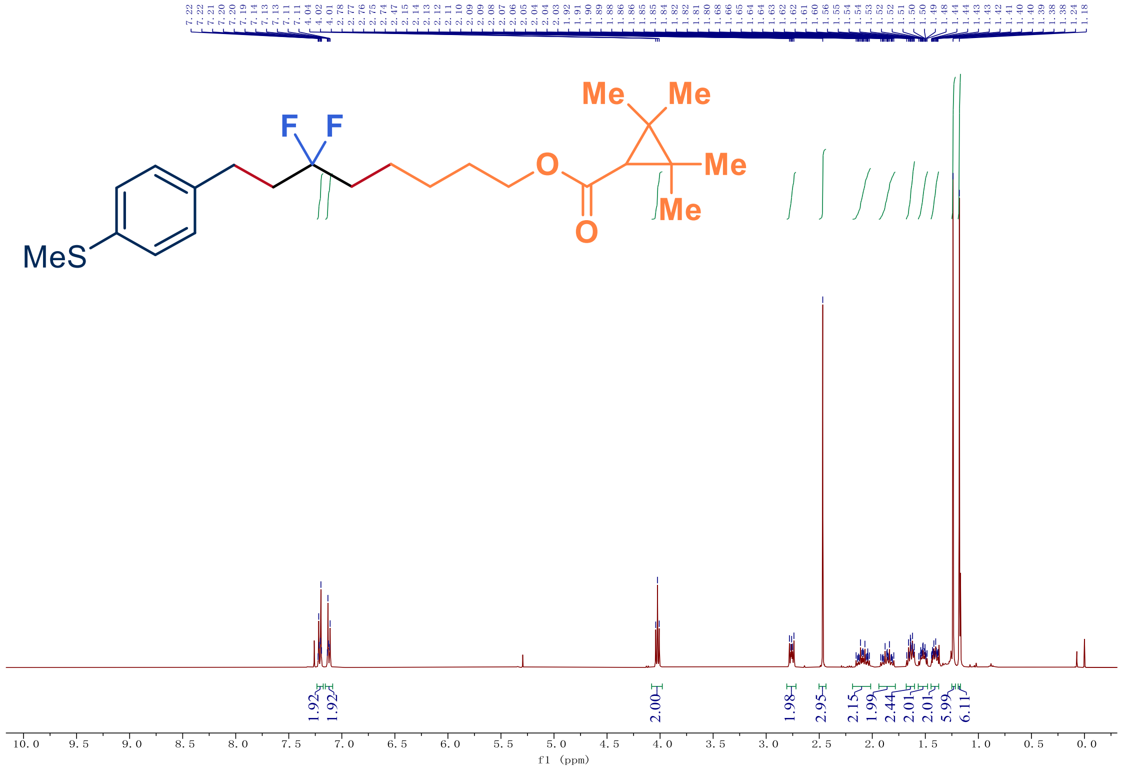

^{19}F NMR (377 MHz, $CDCl_3$) spectra for compound **2w**

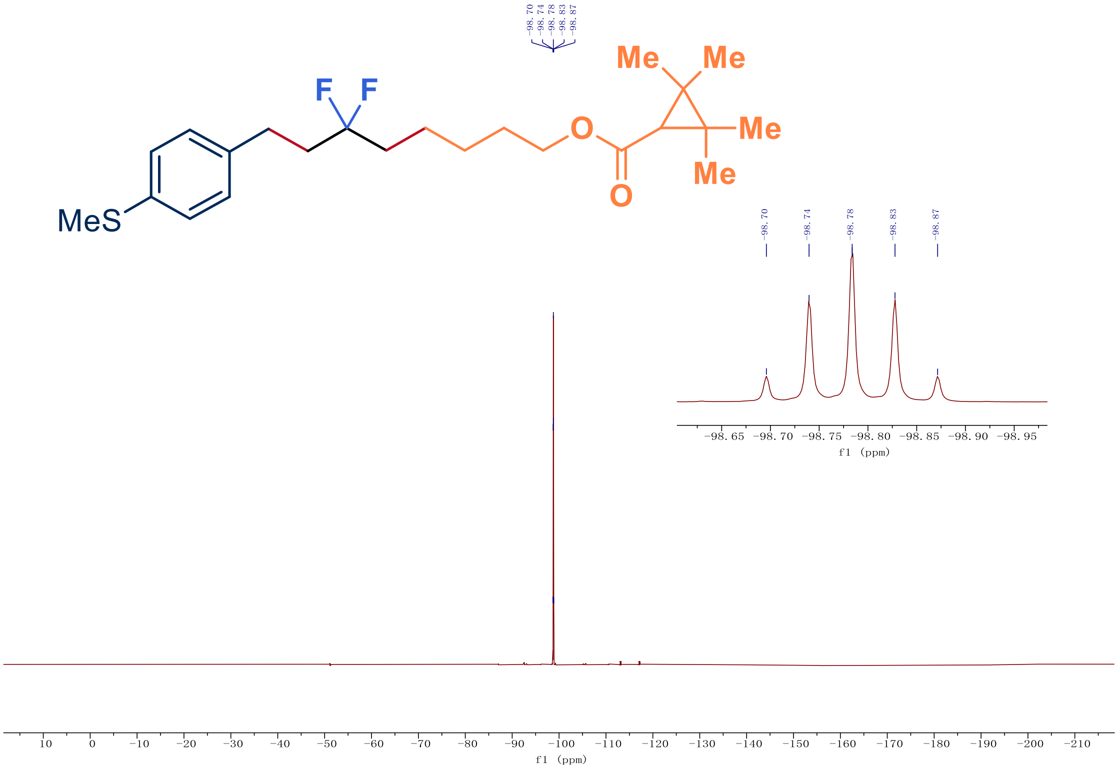

^{13}C NMR (126 MHz, $CDCl_3$) spectra for compound **2w**

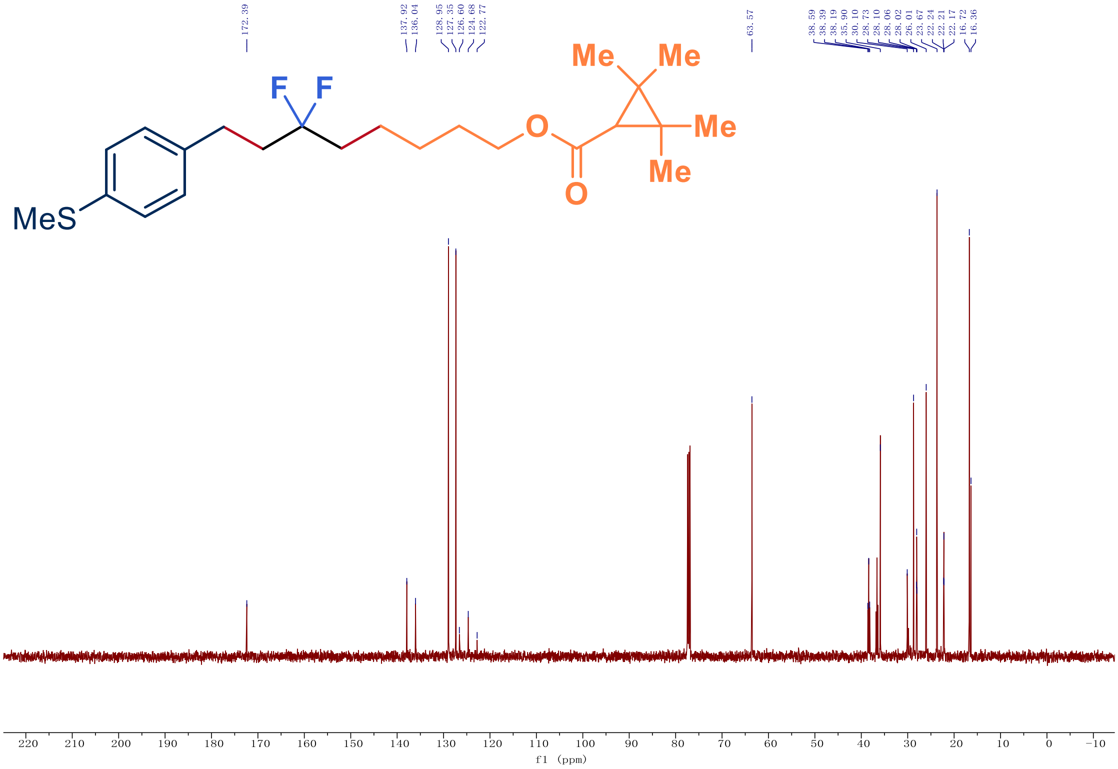

¹H NMR (400 MHz, $CDCl_3$) spectra for compound **2x**

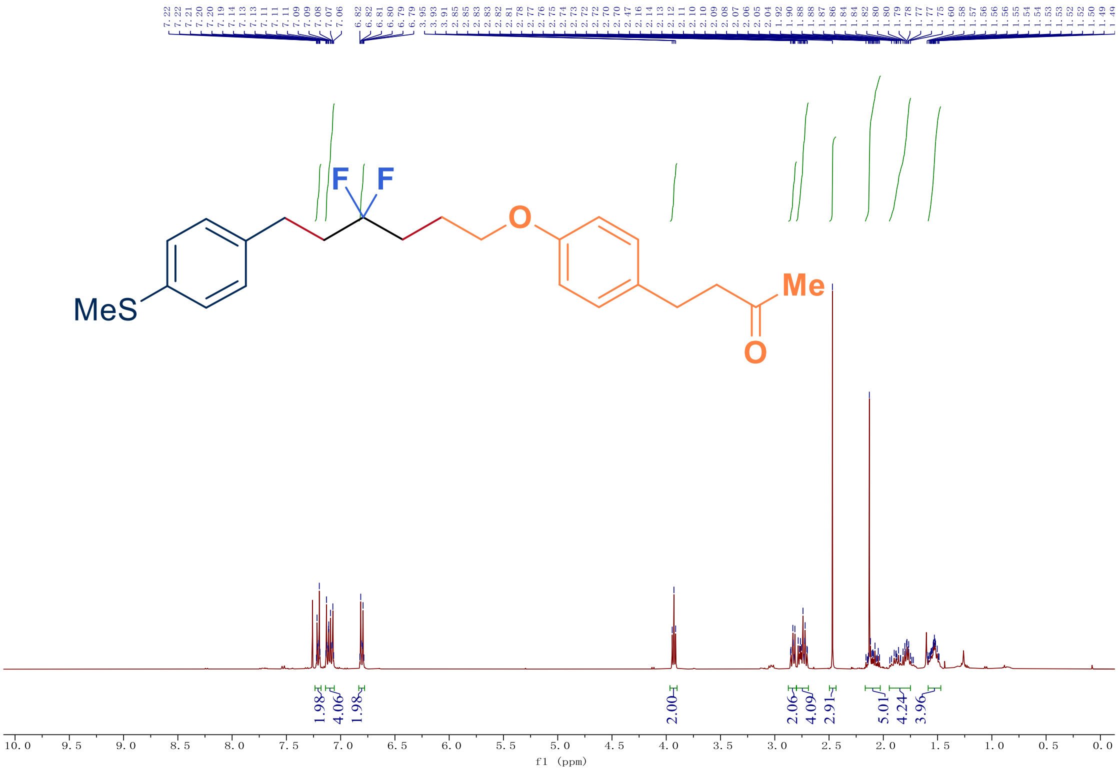

¹⁹F NMR (377 MHz, $CDCl_3$) spectra for compound **2x**

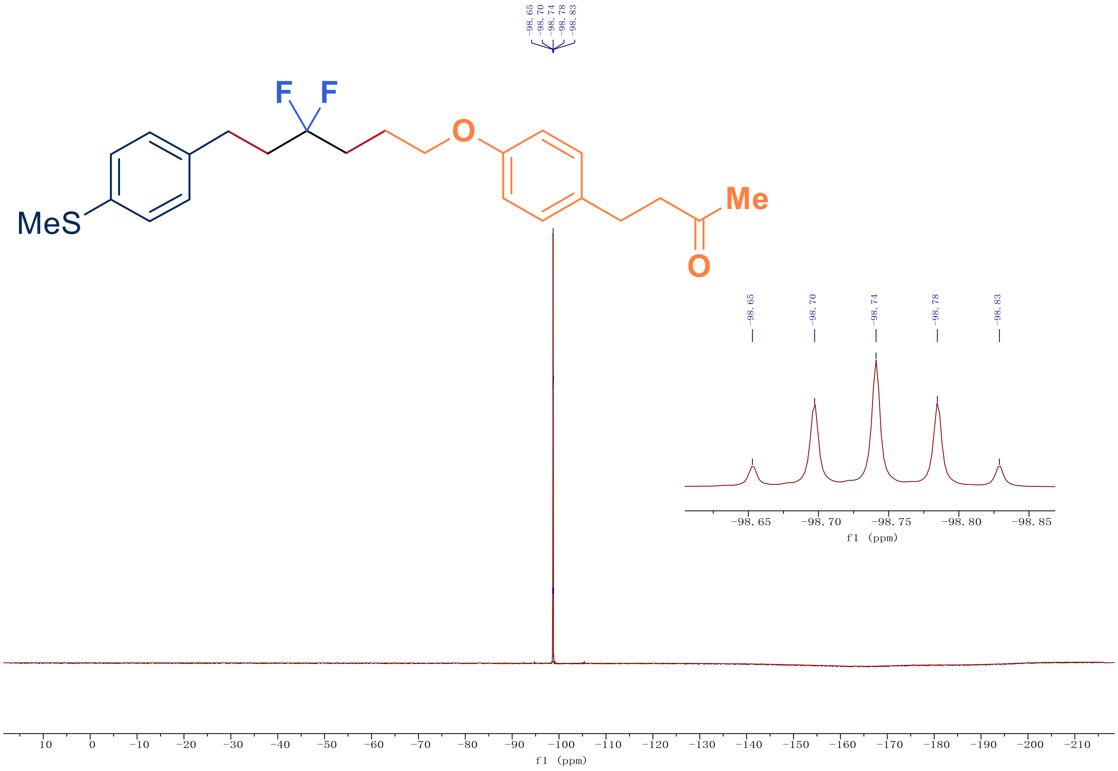

^{13}C NMR (126 MHz, $CDCl_3$) spectra for compound **2x**

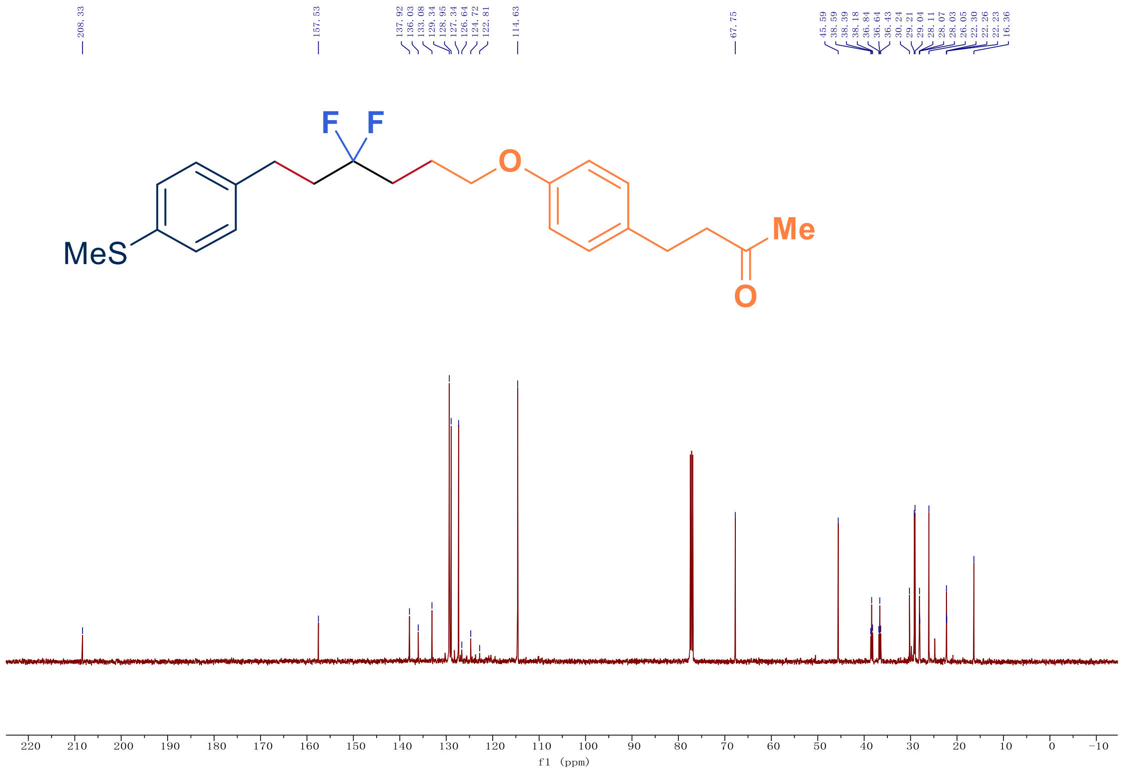

^{1}H NMR (400 MHz, $CDCl_3$) spectra for compound **2y**

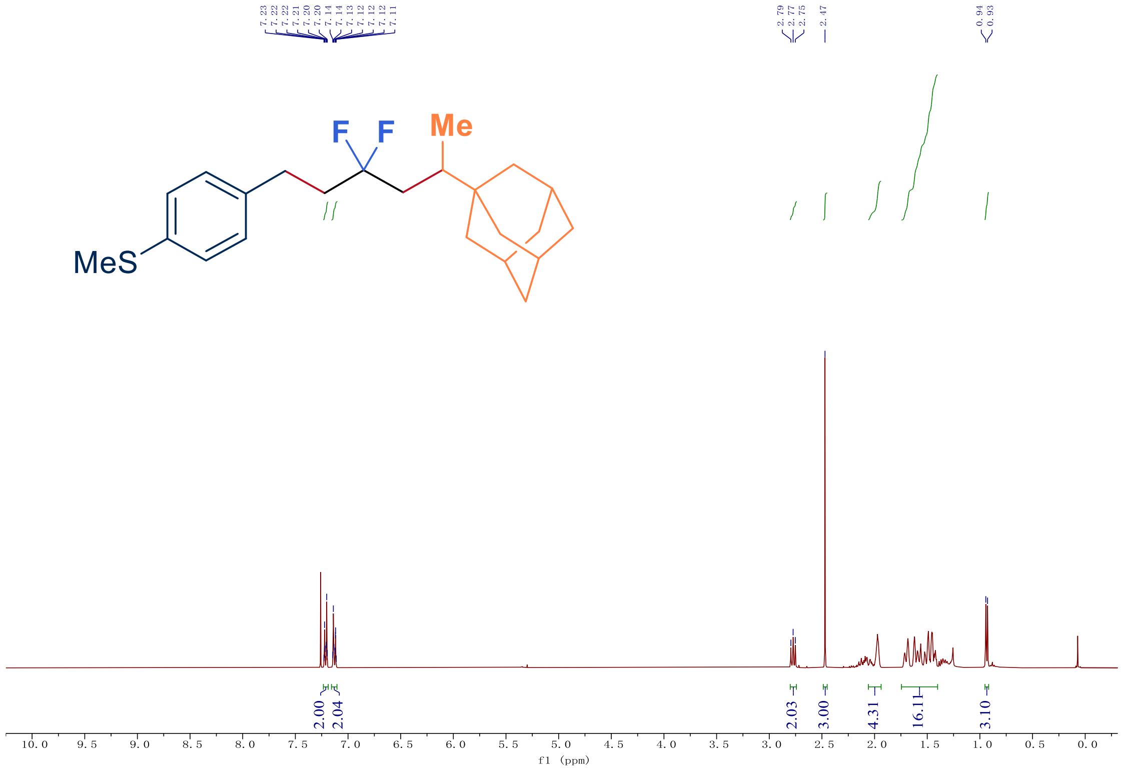

^{19}F NMR (377 MHz, CDCl$_3$) spectra for compound **2y**

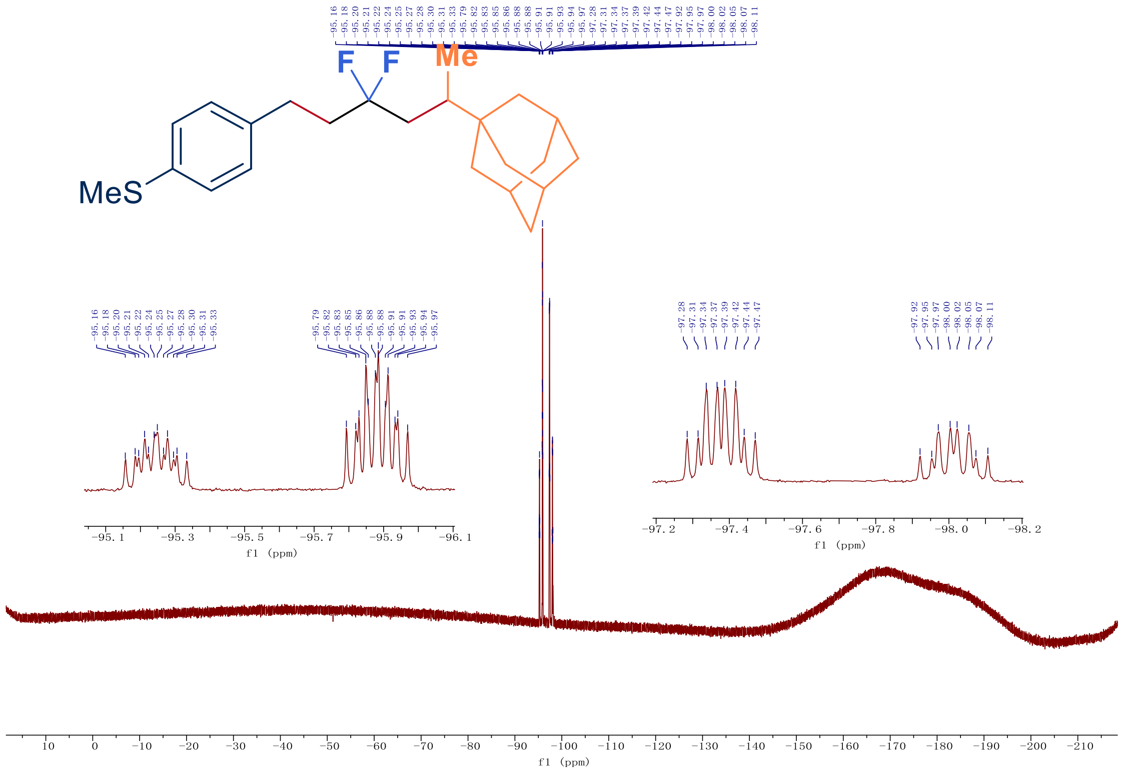

^{13}C NMR (126 MHz, CDCl$_3$) spectra for compound **2y**

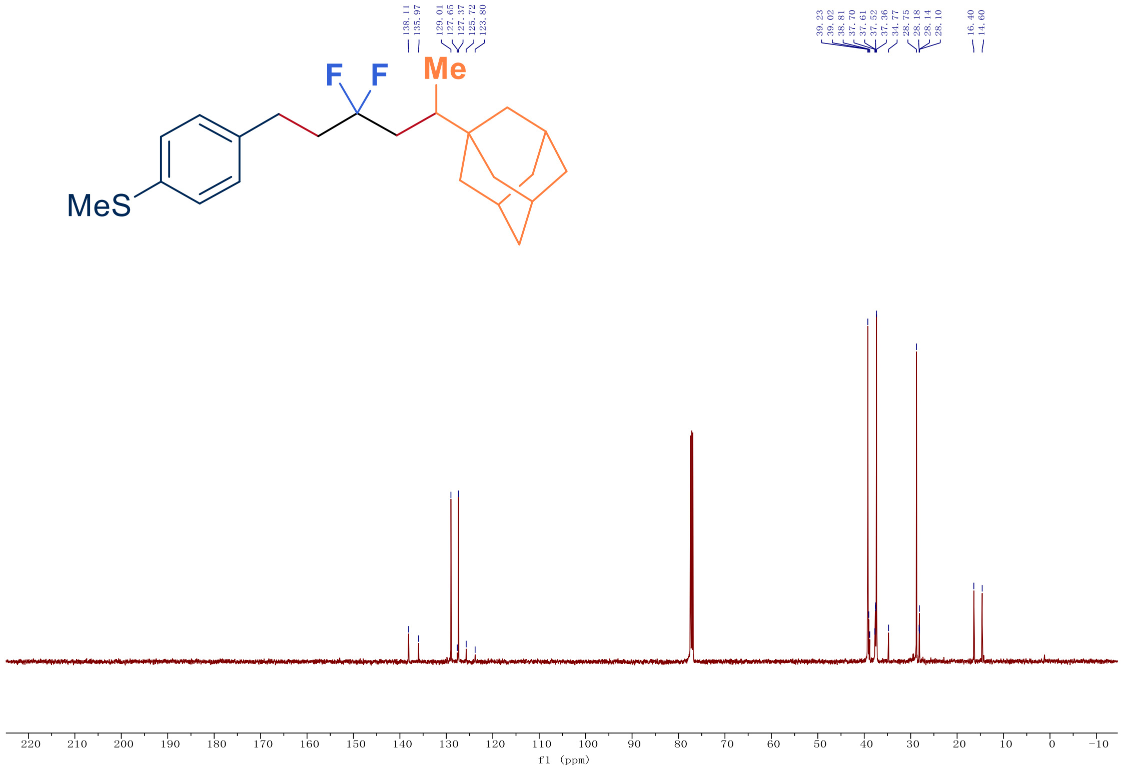

^{1}**H NMR** (400 MHz, $CDCl_3$) spectra for compound **2z**

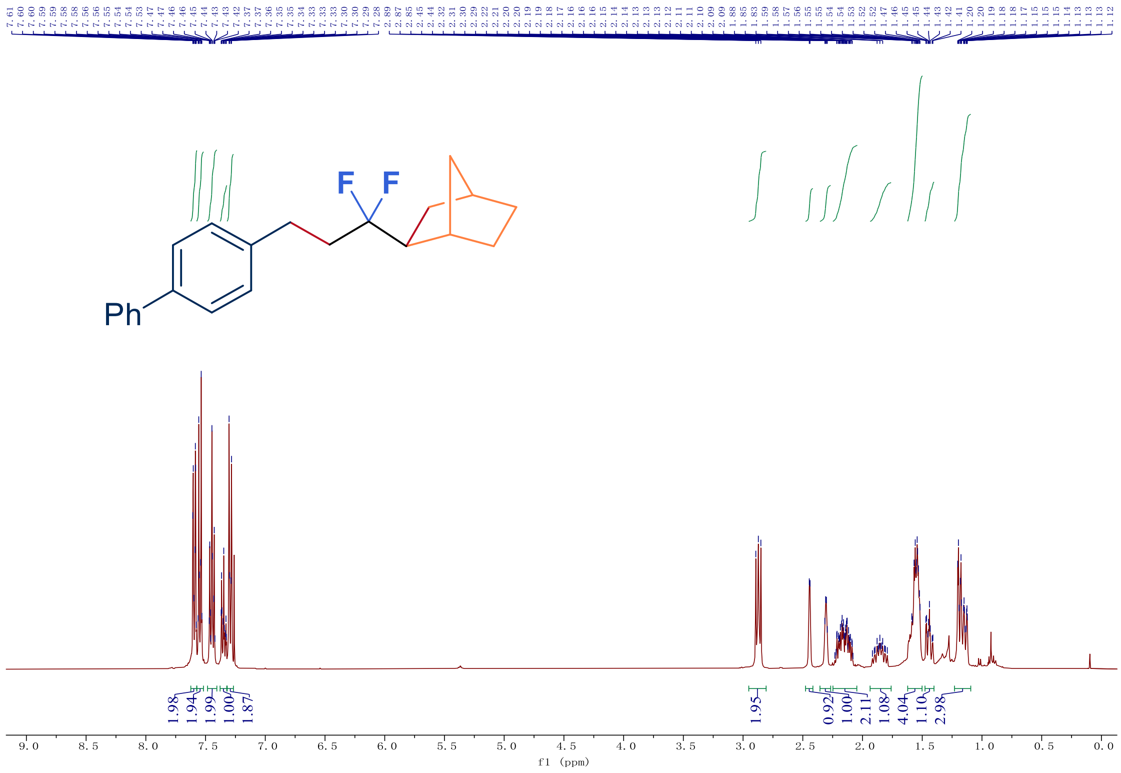

^{19}**F NMR** (377 MHz, $CDCl_3$) spectra for compound **2z**

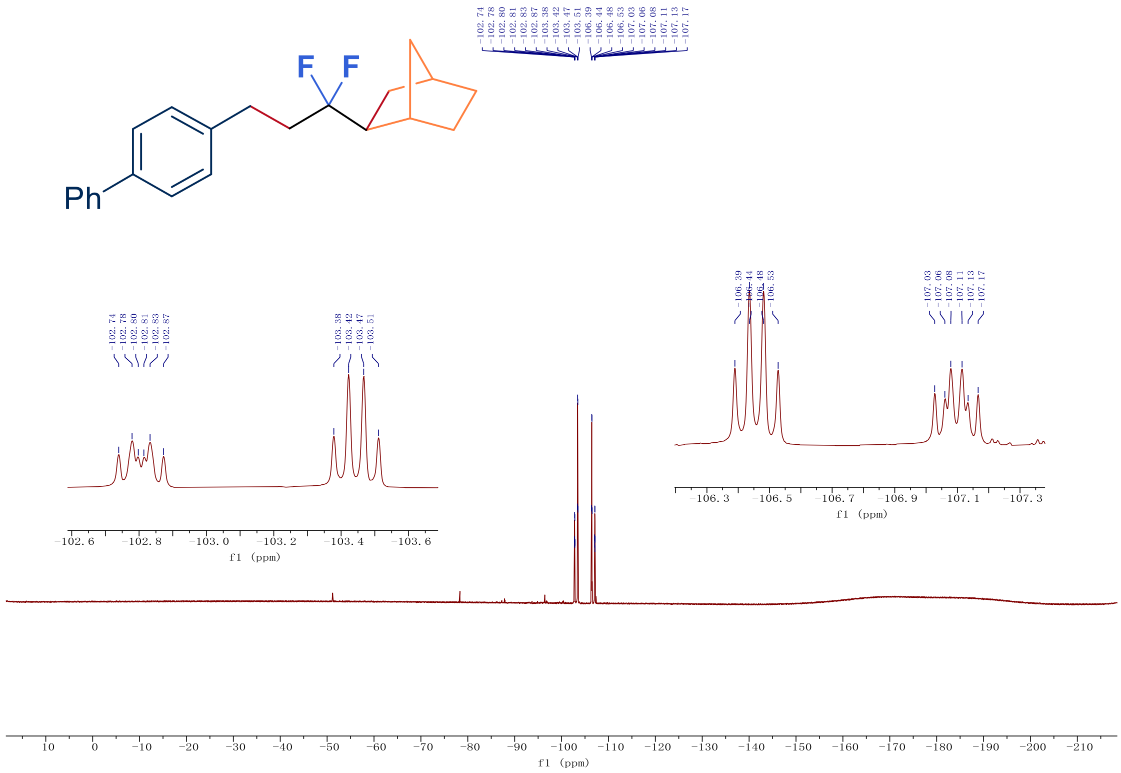

^{13}C NMR (126 MHz, $CDCl_3$) spectra for compound **2z**

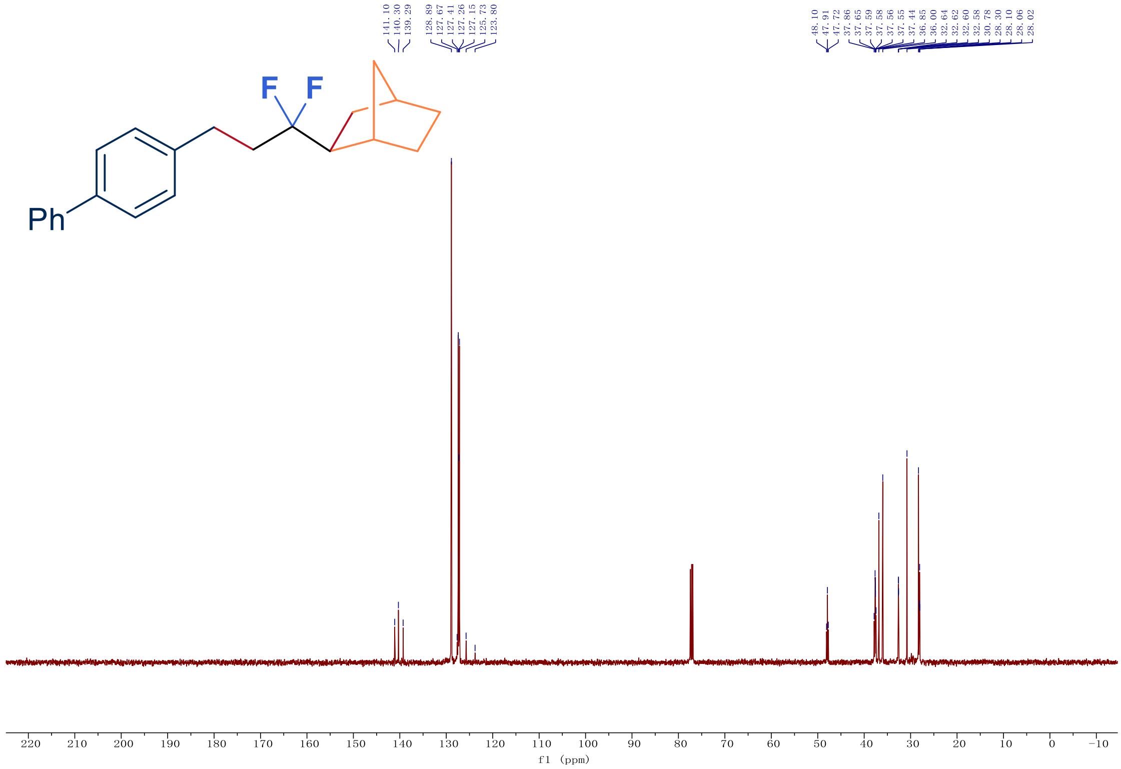

^{1}H NMR (400 MHz, $CDCl_3$) spectra for compound **2aa**

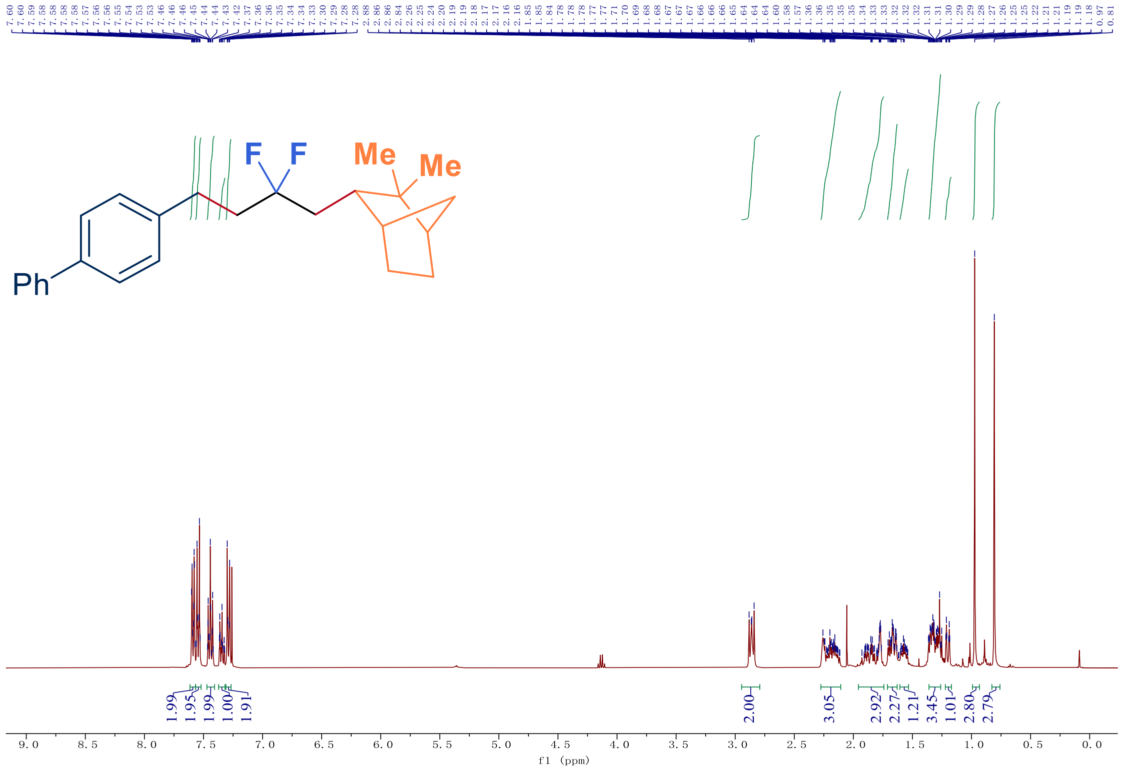

^{19}F NMR (377 MHz, $CDCl_3$) spectra for compound **2aa**

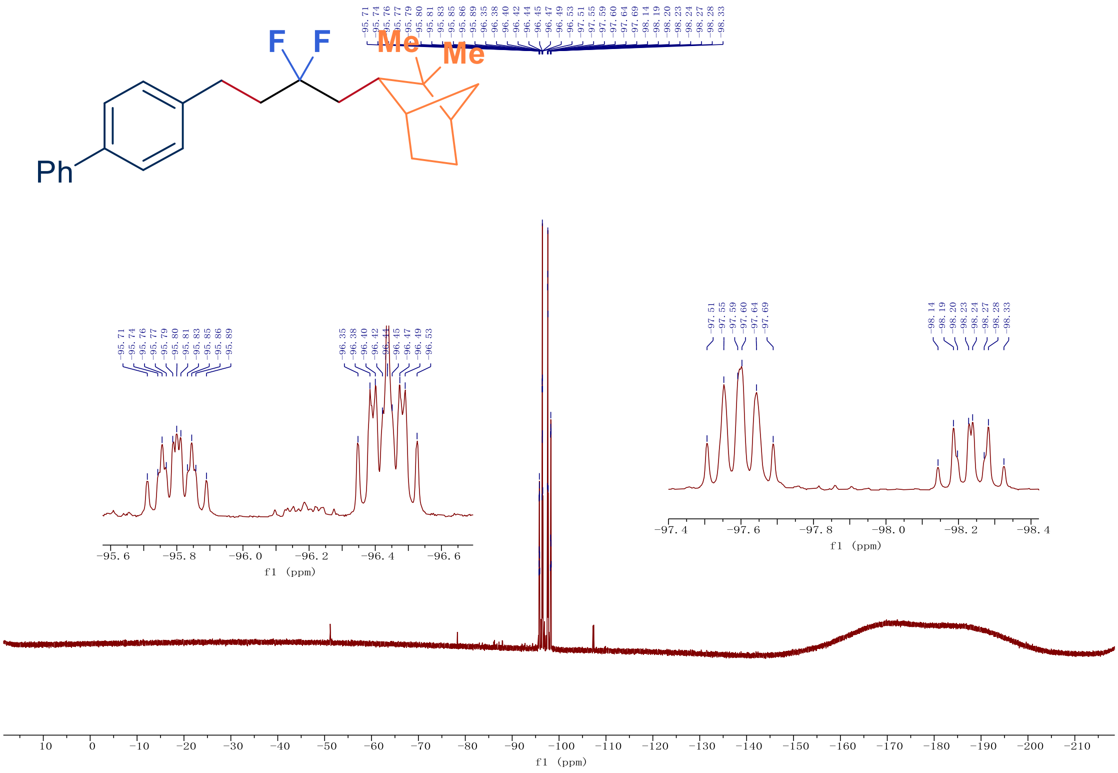

^{13}C NMR (126 MHz, $CDCl_3$) spectra for compound **2aa**

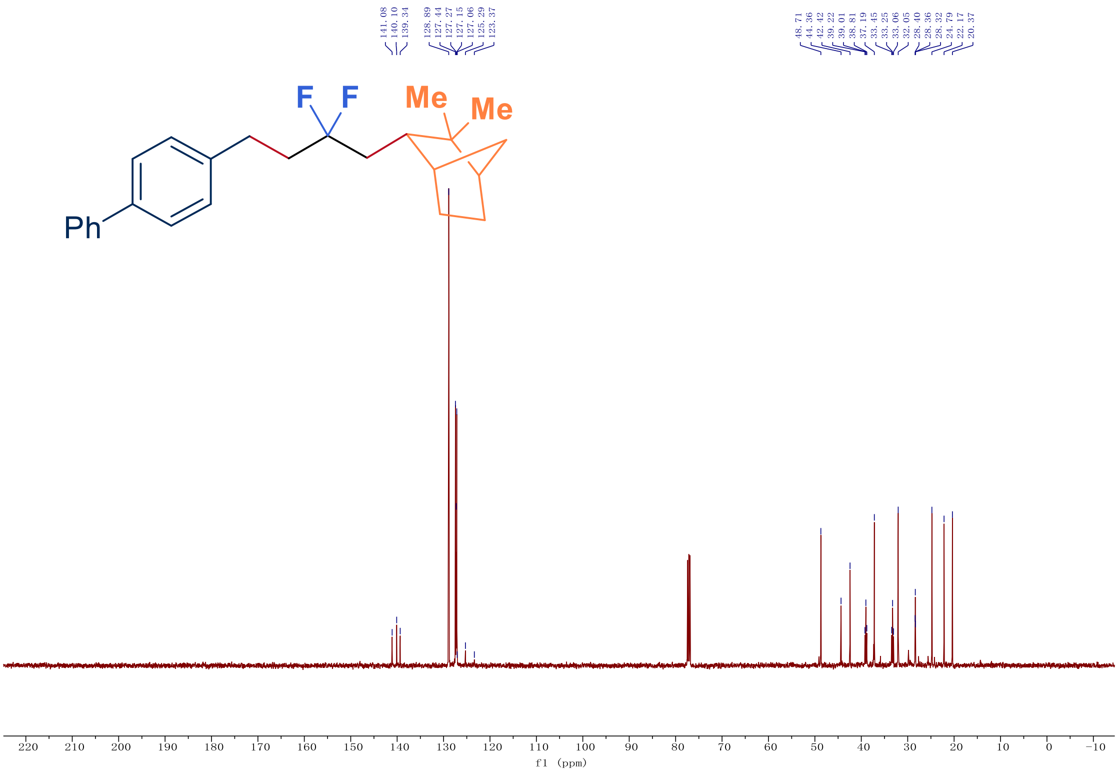

^{1}H NMR (400 MHz, $CDCl_3$) spectra for compound **2ab**

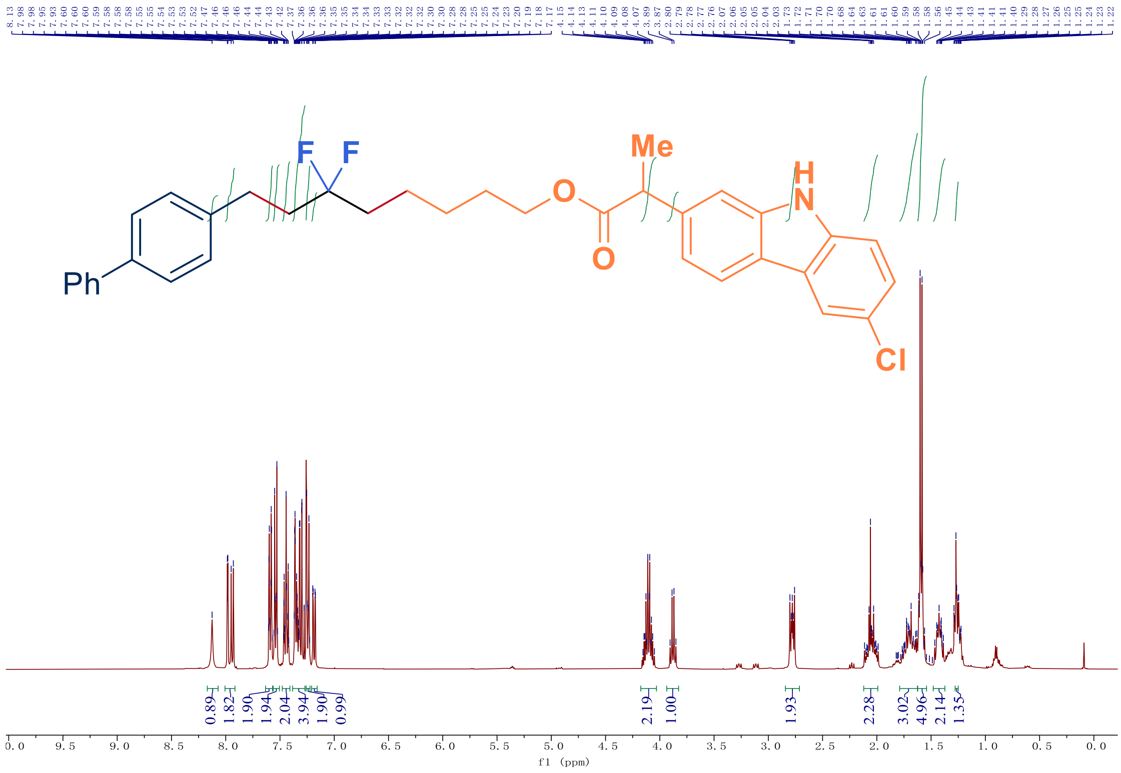

^{19}F NMR (377 MHz, $CDCl_3$) spectra for compound **2ab**

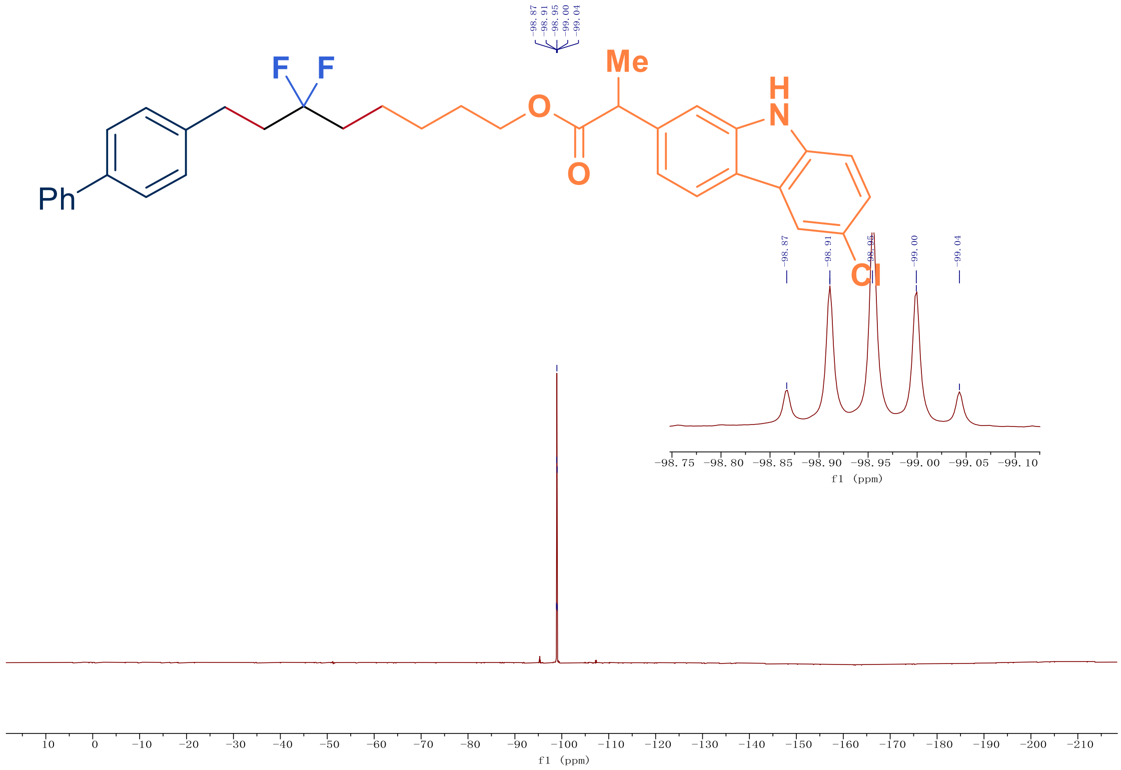

^{13}C NMR (126 MHz, $CDCl_3$) spectra for compound **2ab**

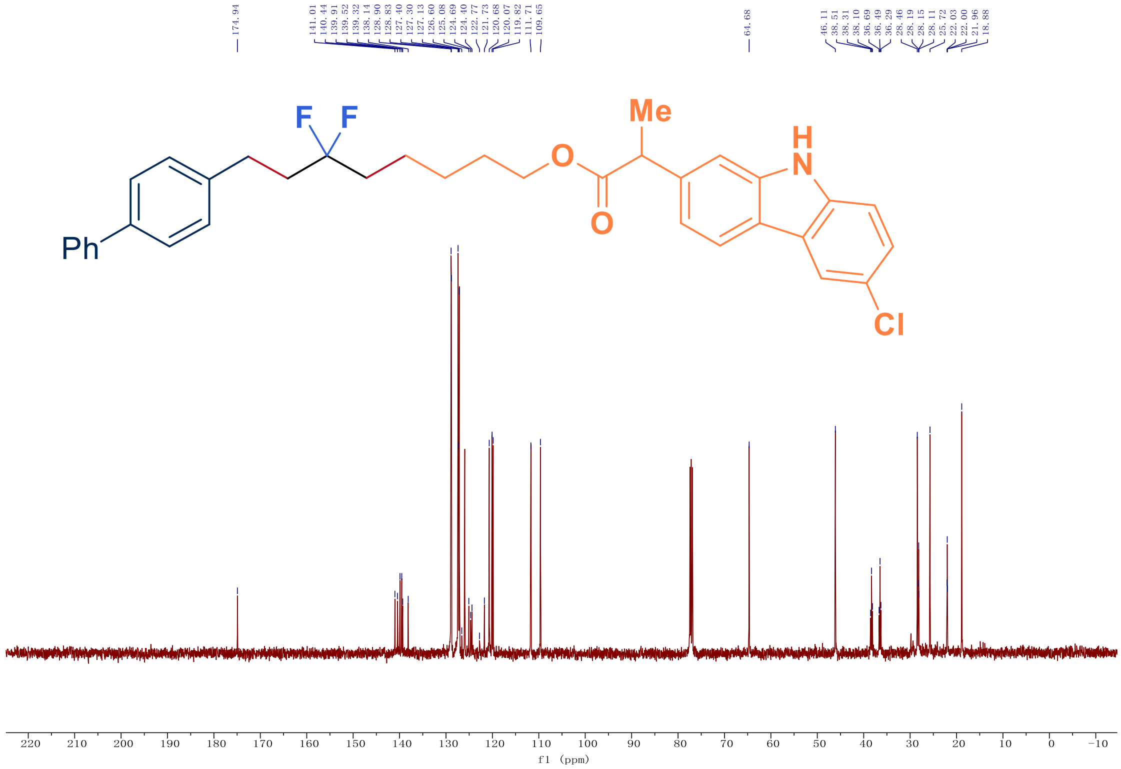

^{1}H NMR (400 MHz, $CDCl_3$) spectra for compound **2ac**

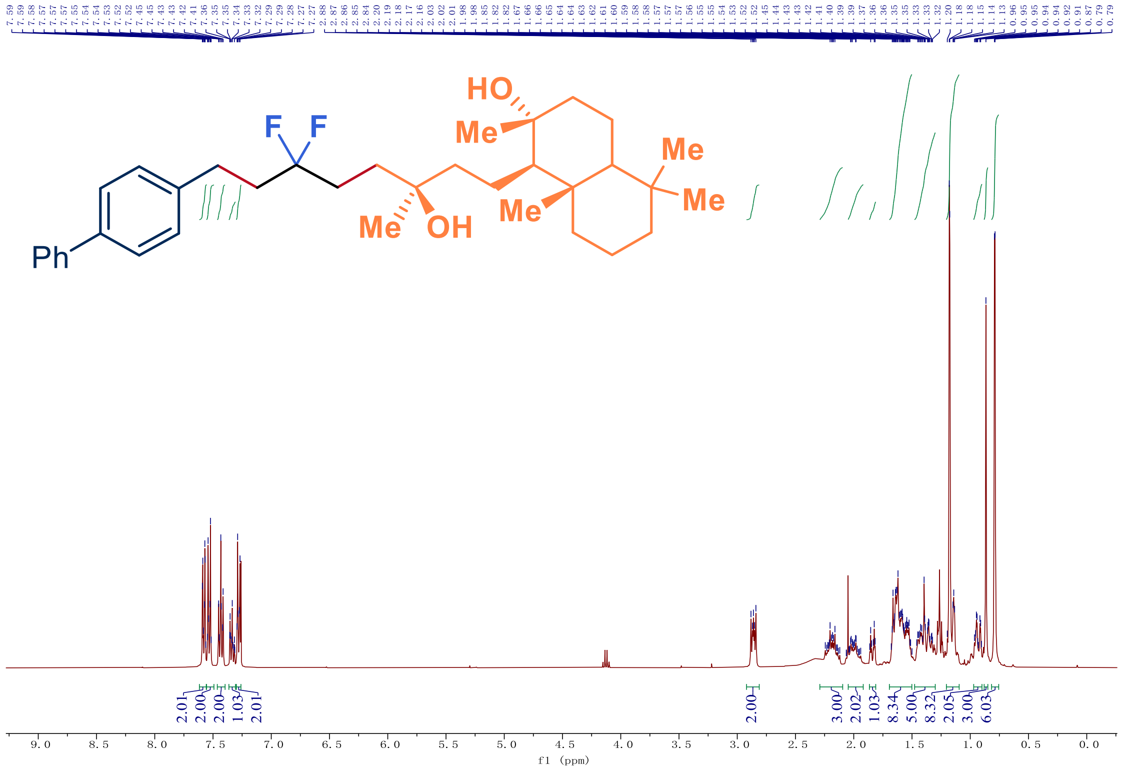

^{19}F NMR (377 MHz, $CDCl_3$) spectra for compound **2ac**

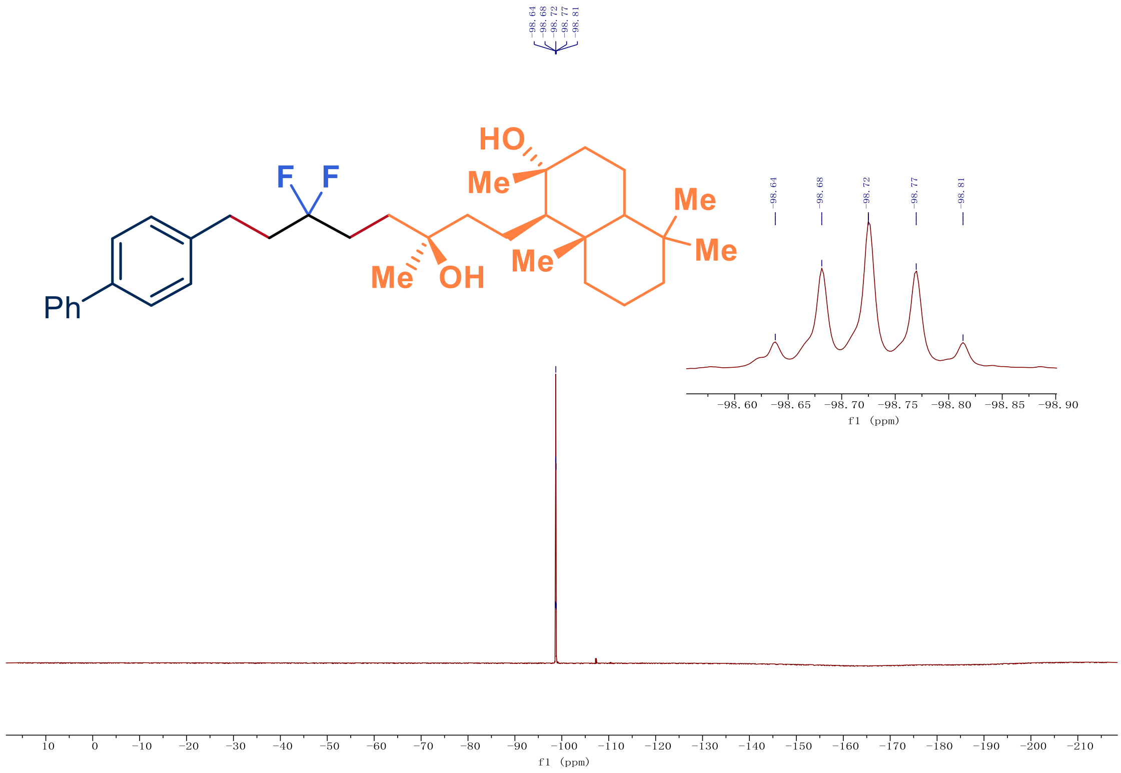

^{13}C NMR (126 MHz, $CDCl_3$) spectra for compound **2ac**

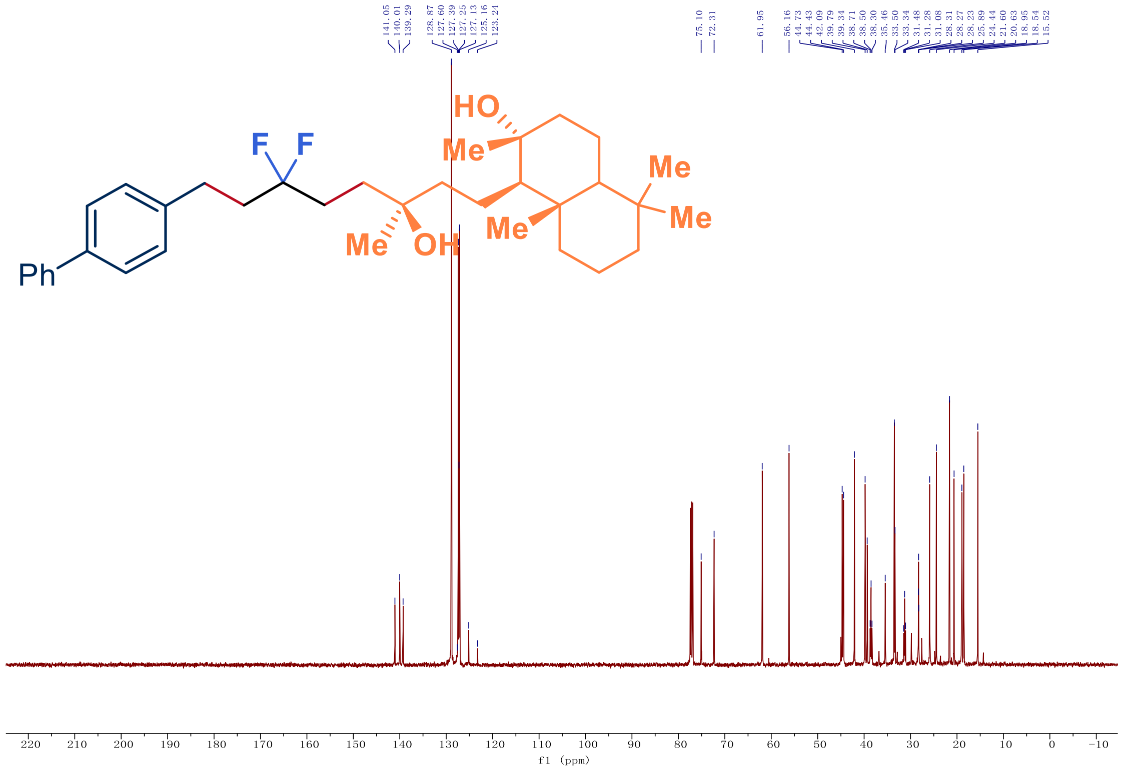

^{1}H NMR (400 MHz, CDCl$_3$) spectra for compound **2ad**

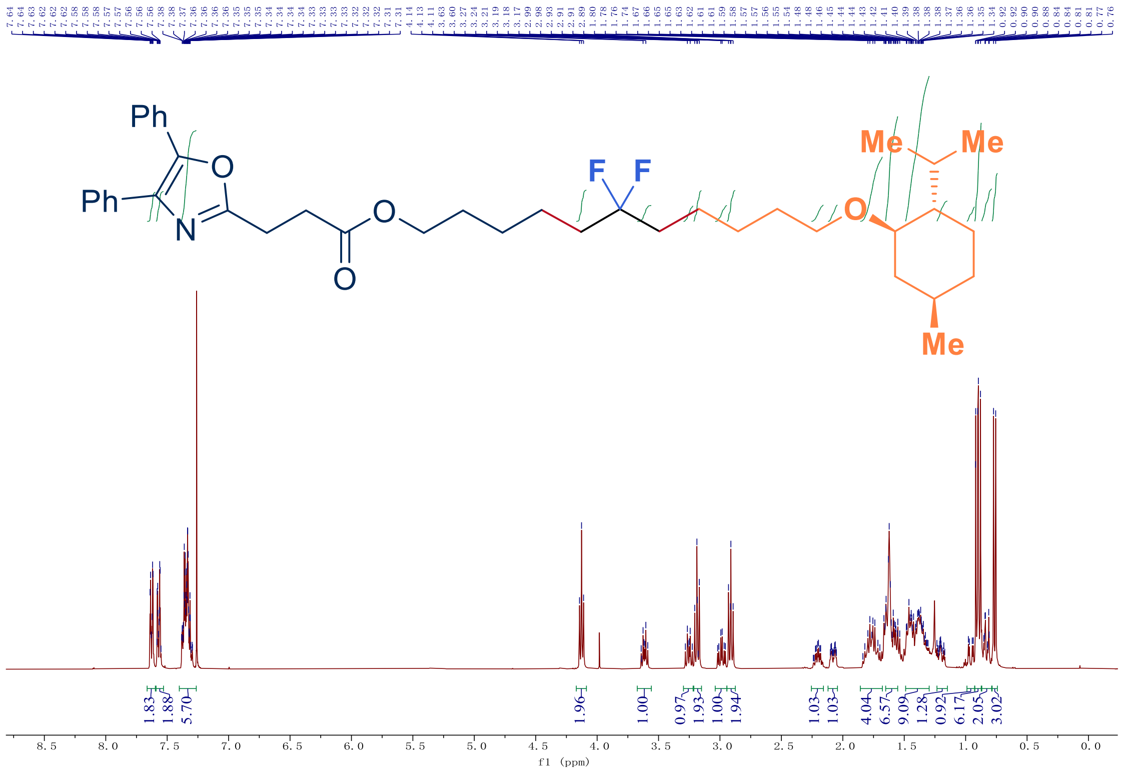

^{19}F NMR (377 MHz, CDCl$_3$) spectra for compound **2ad**

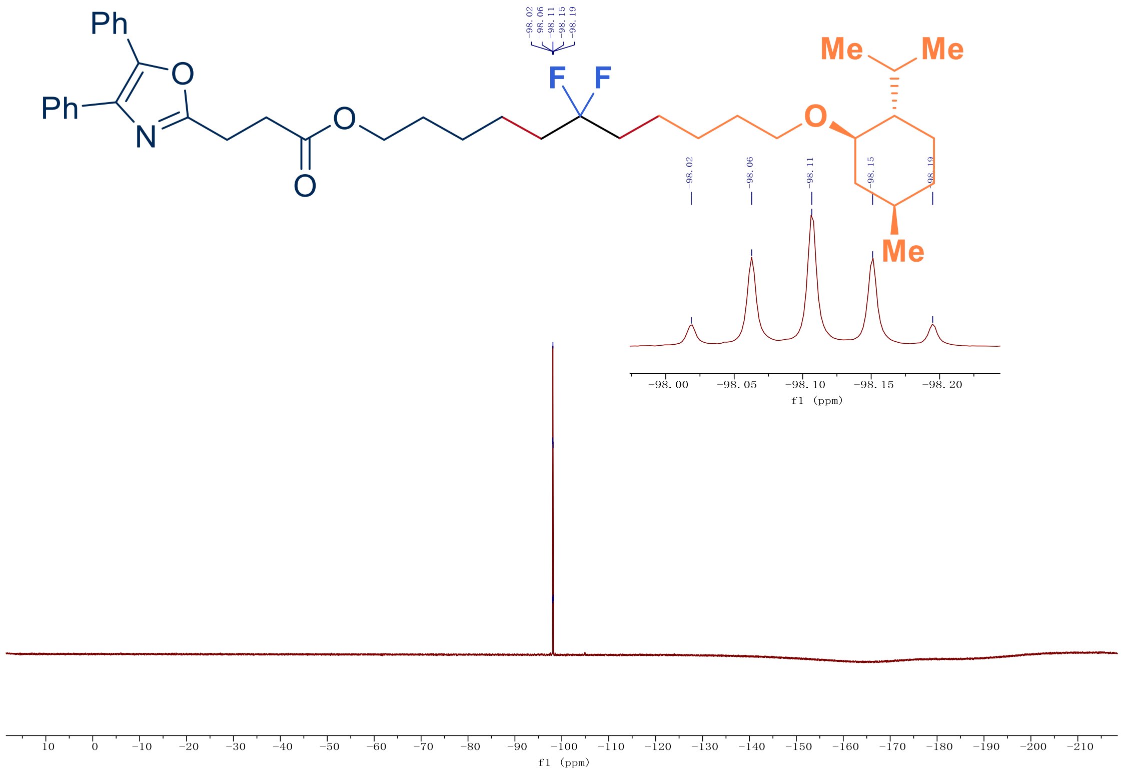

^{13}C NMR (126 MHz, $CDCl_3$) spectra for compound **2ad**

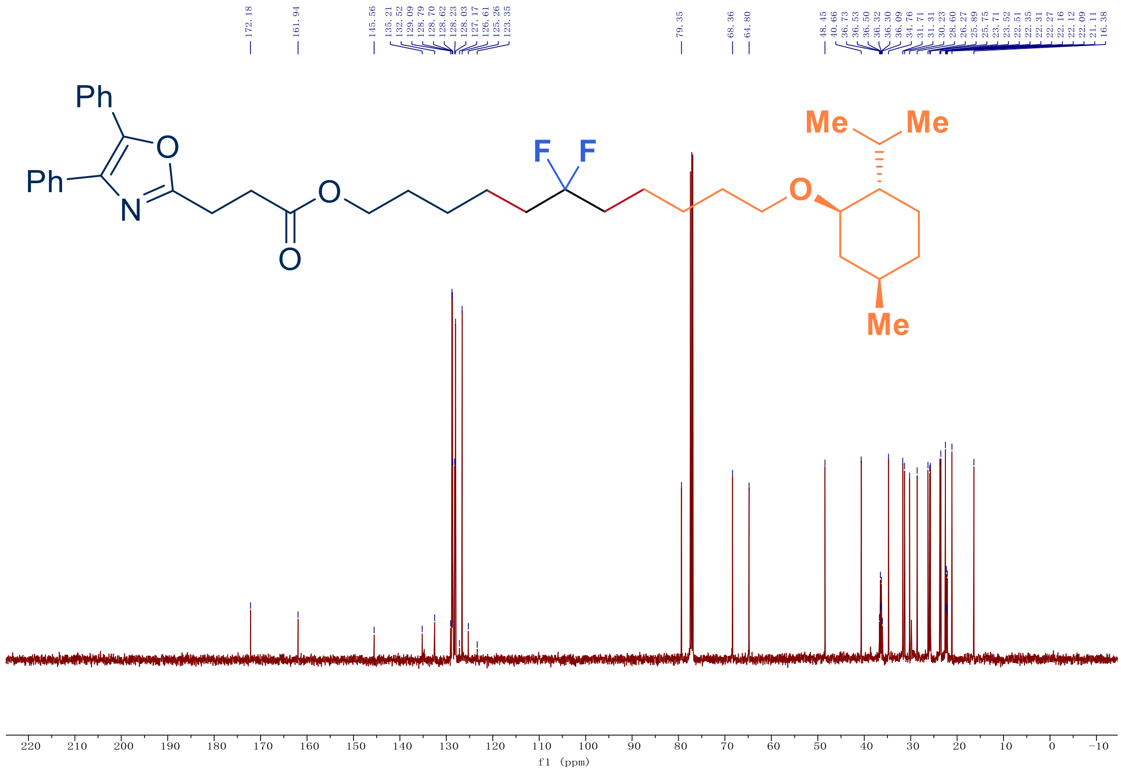

^{1}H NMR (400 MHz, $CDCl_3$) spectra for compound **2ae**

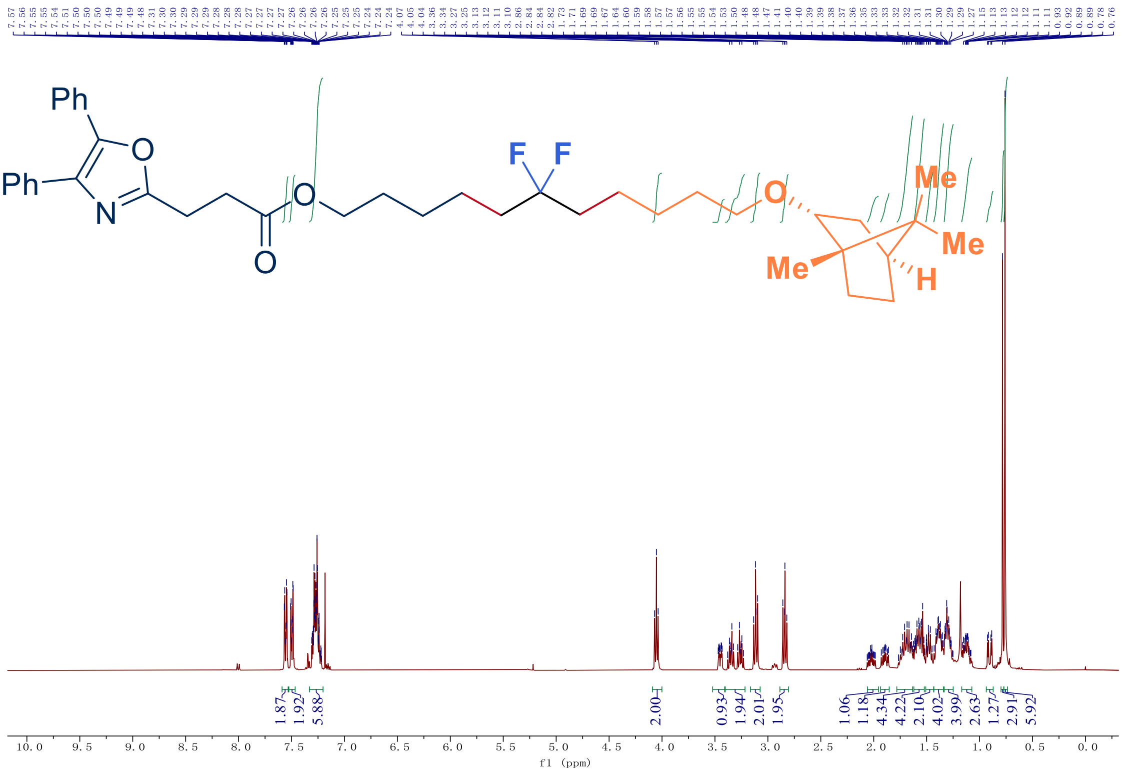

^{19}F NMR (377 MHz, $CDCl_3$) spectra for compound **2ae**

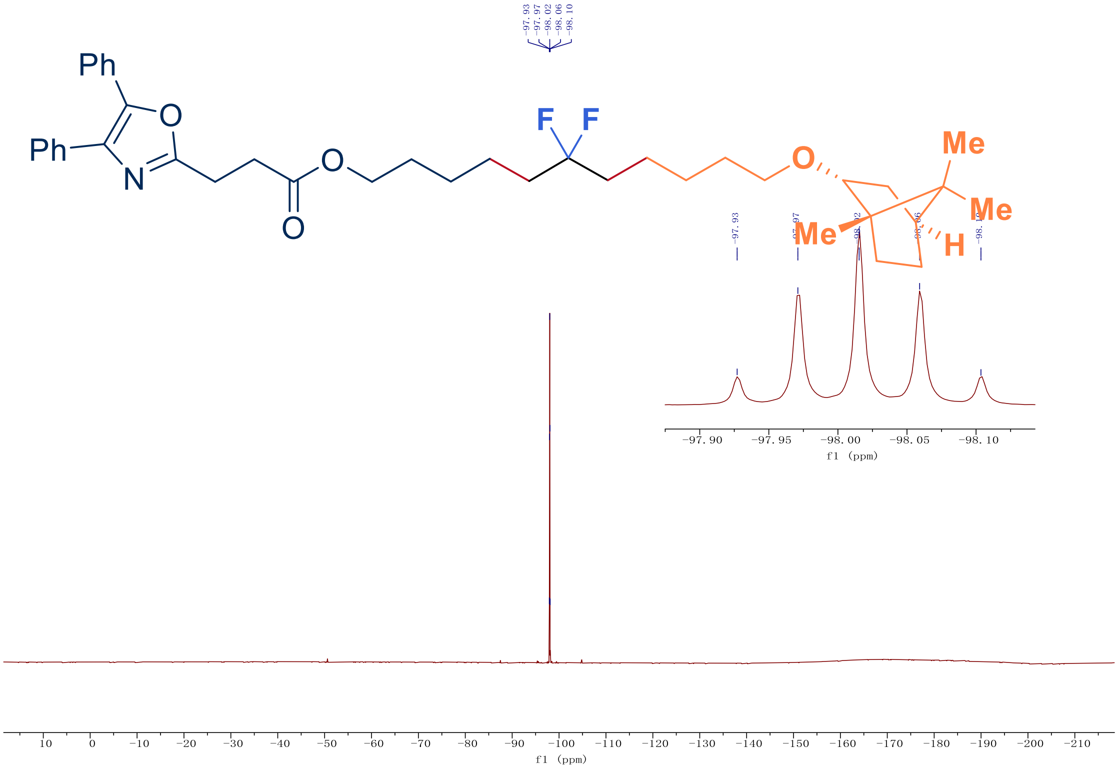

^{13}C NMR (126 MHz, $CDCl_3$) spectra for compound **2ae**

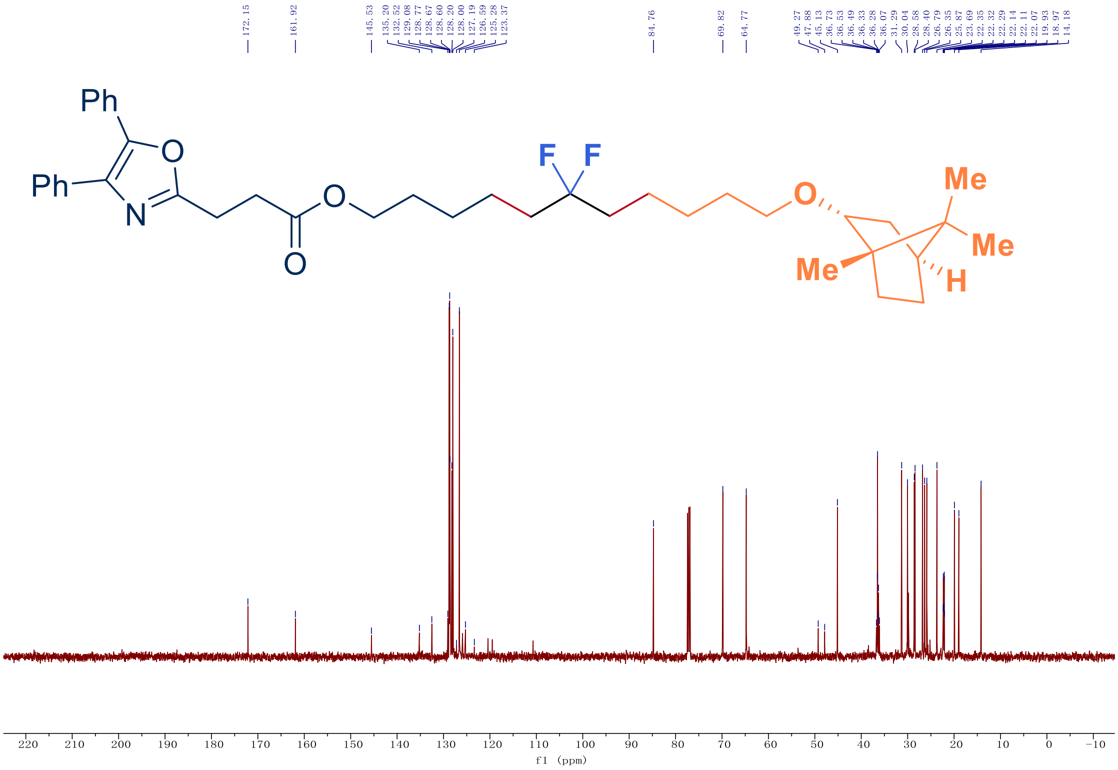

^{1}H NMR (400 MHz, $CDCl_3$) spectra for compound **2af**

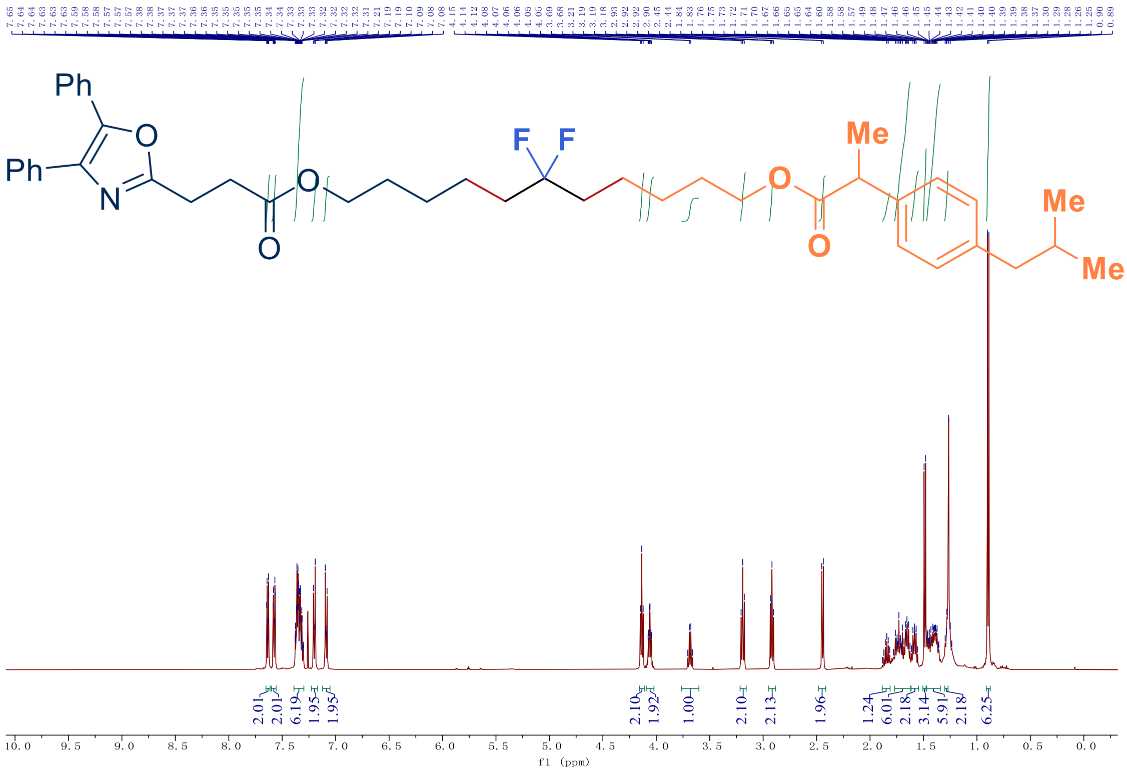

^{19}F NMR (377 MHz, $CDCl_3$) spectra for compound **2af**

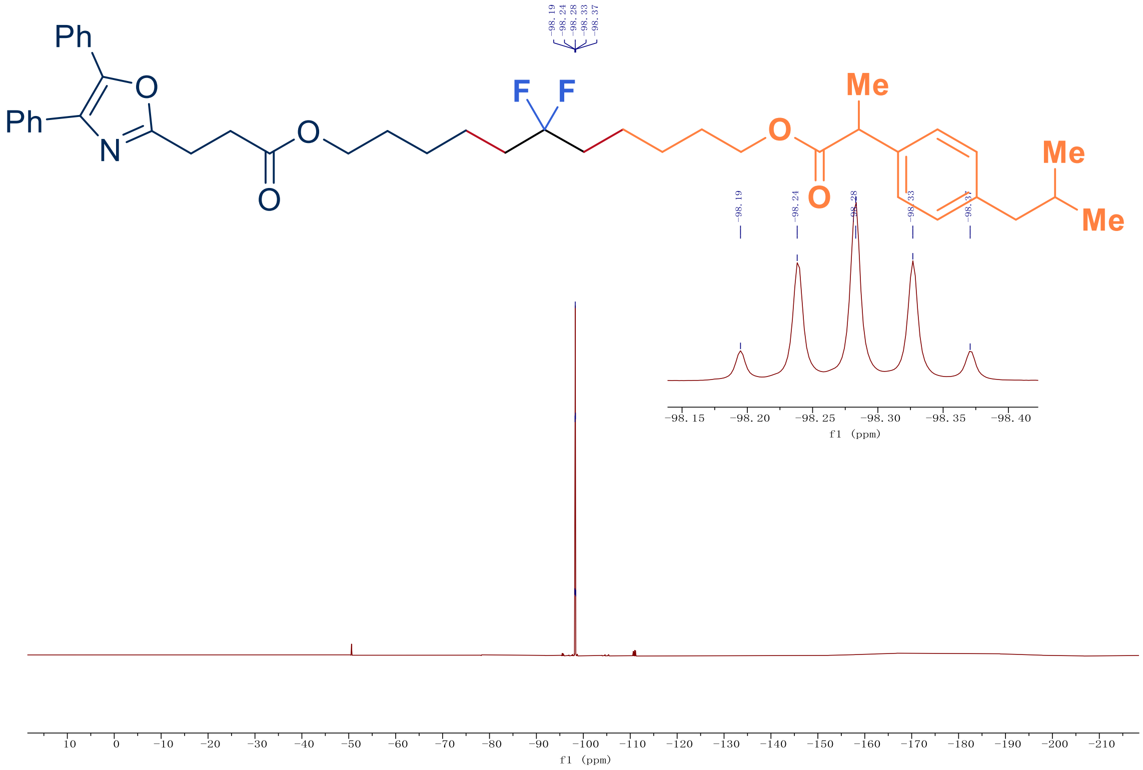

^{13}C NMR (126 MHz, $CDCl_3$) spectra for compound **2af**

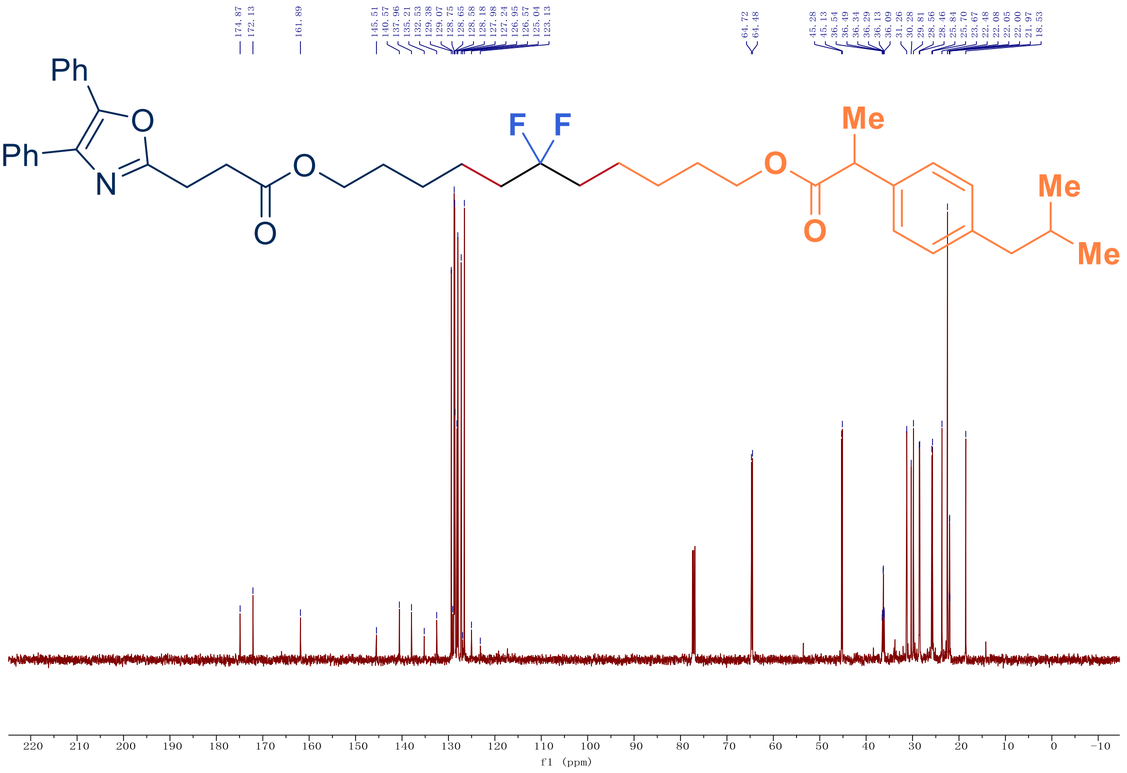

^{1}H NMR (400 MHz, $CDCl_3$) spectra for compound **2ag**

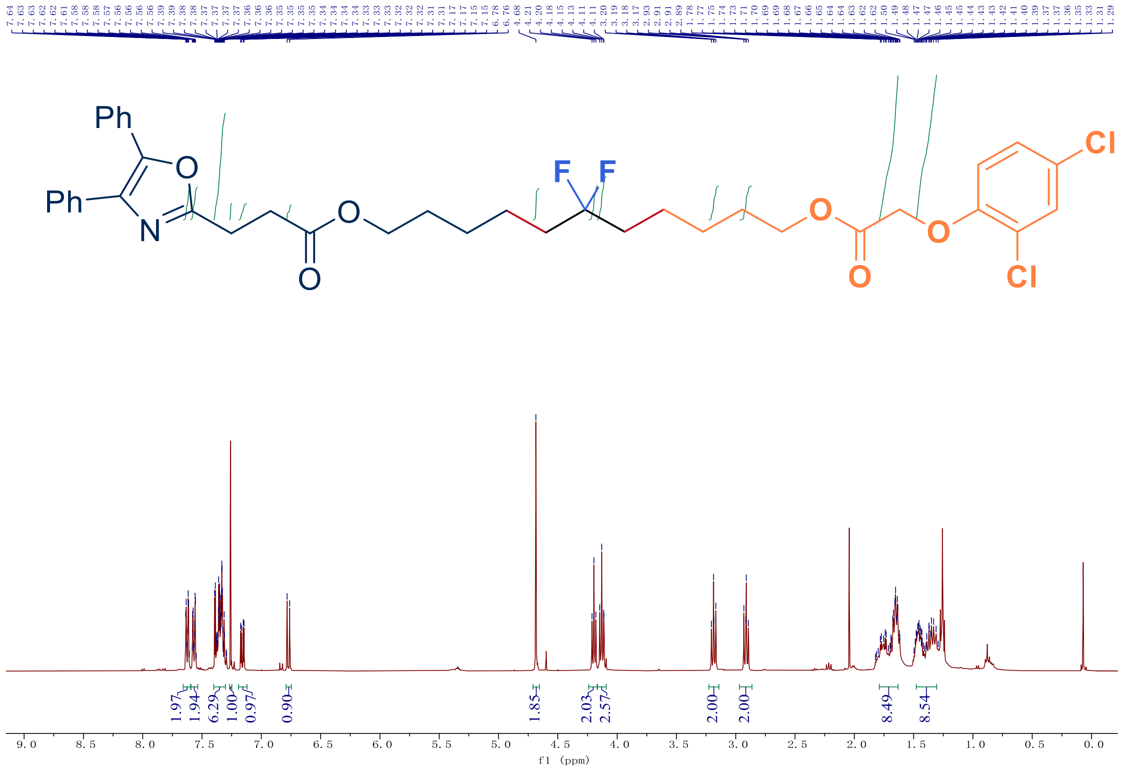

^{19}F NMR (377 MHz, $CDCl_3$) spectra for compound **2ah**

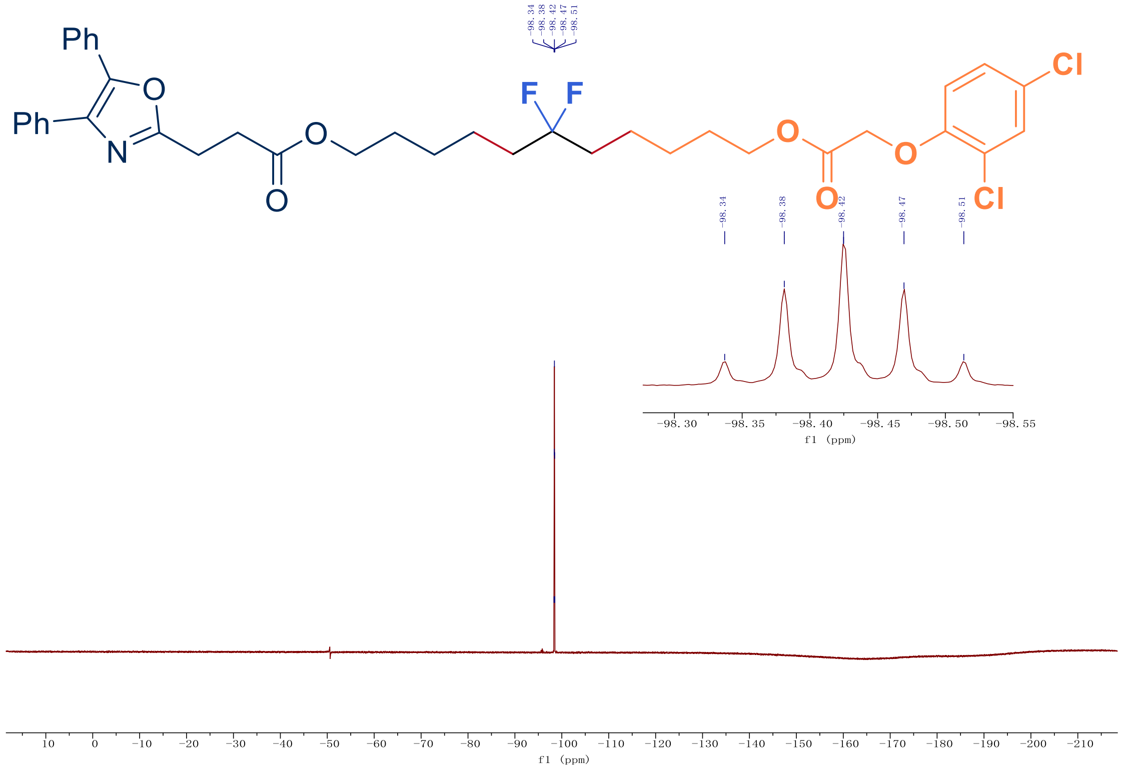

^{13}C NMR (126 MHz, $CDCl_3$) spectra for compound **2ah**

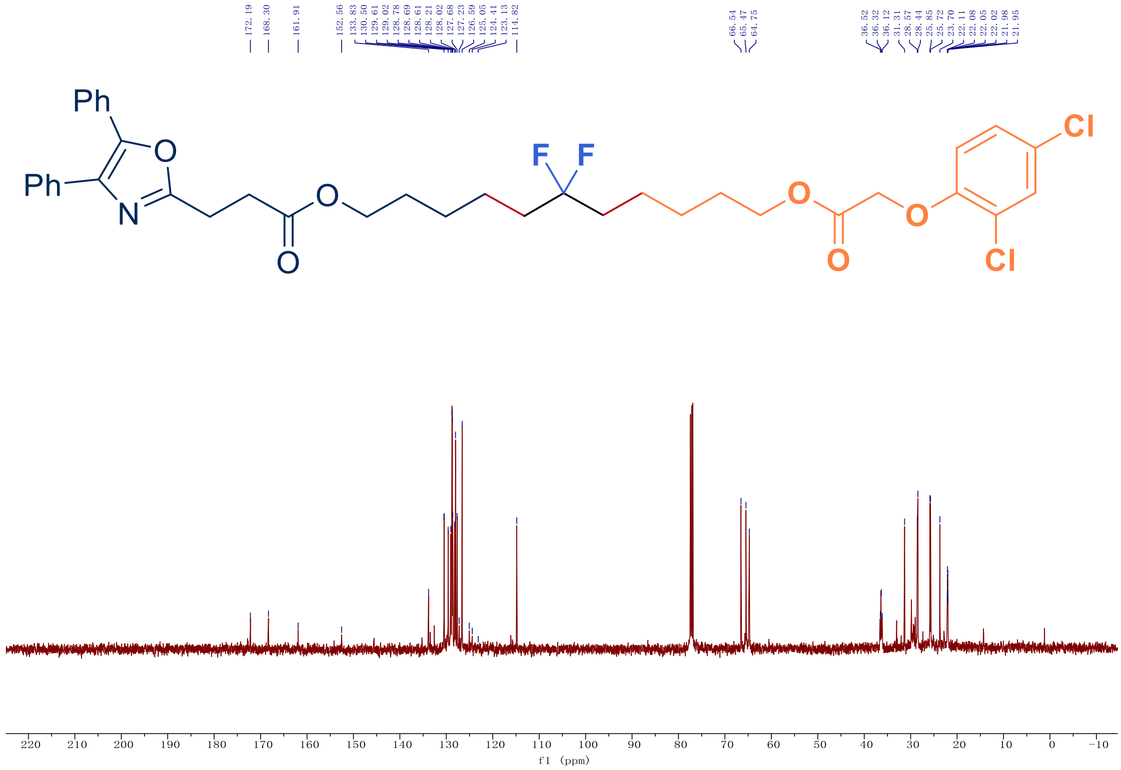

^{1}H NMR (400 MHz, $CDCl_3$) spectra for compound **3a**

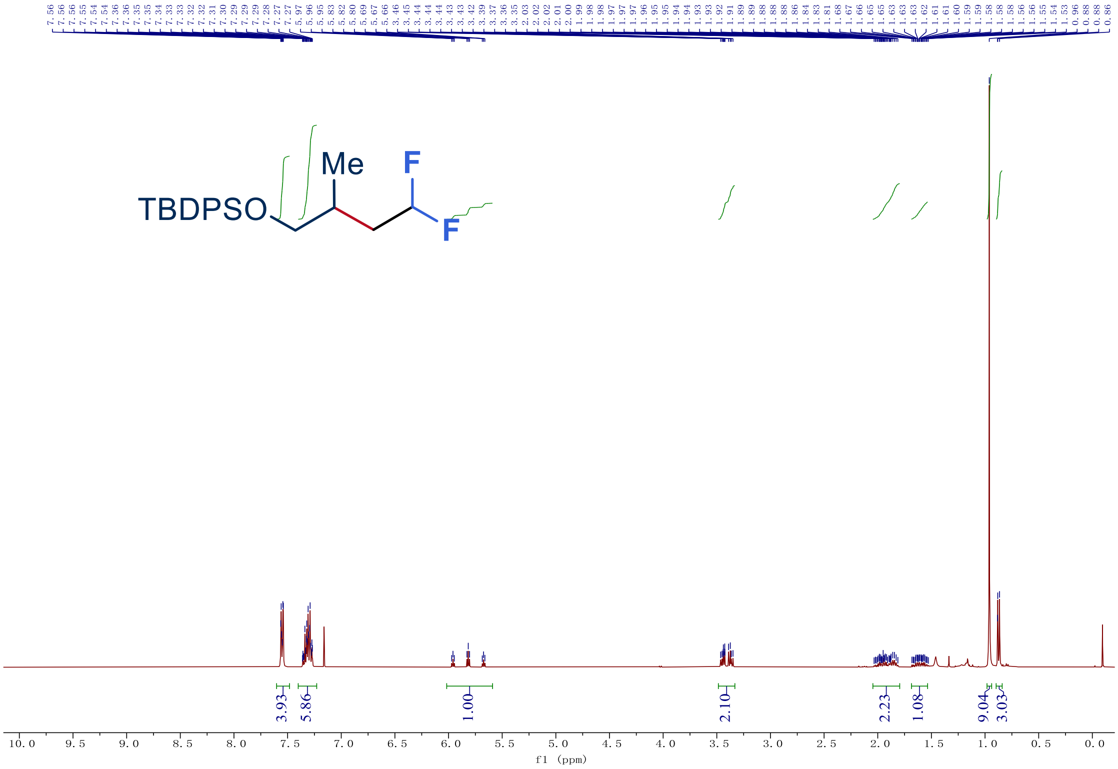

^{19}F NMR (377 MHz, $CDCl_3$) spectra for compound **3a**

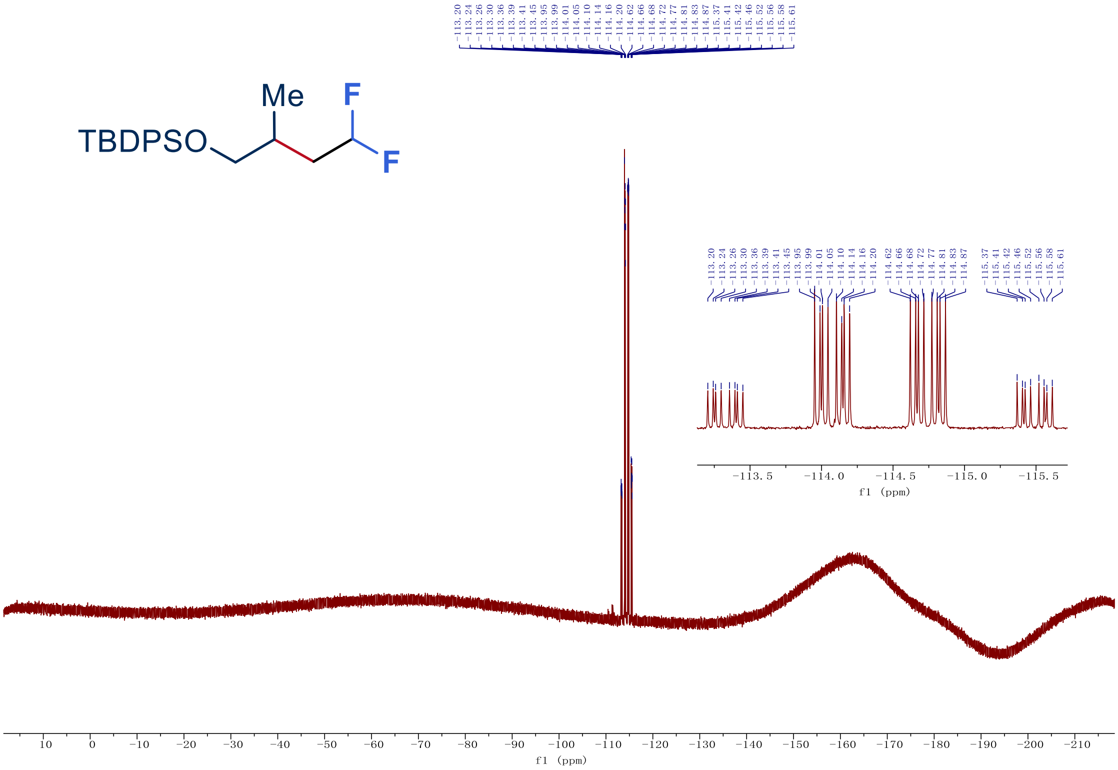

^{1}H NMR (400 MHz, $CDCl_3$) spectra for compound **3b**

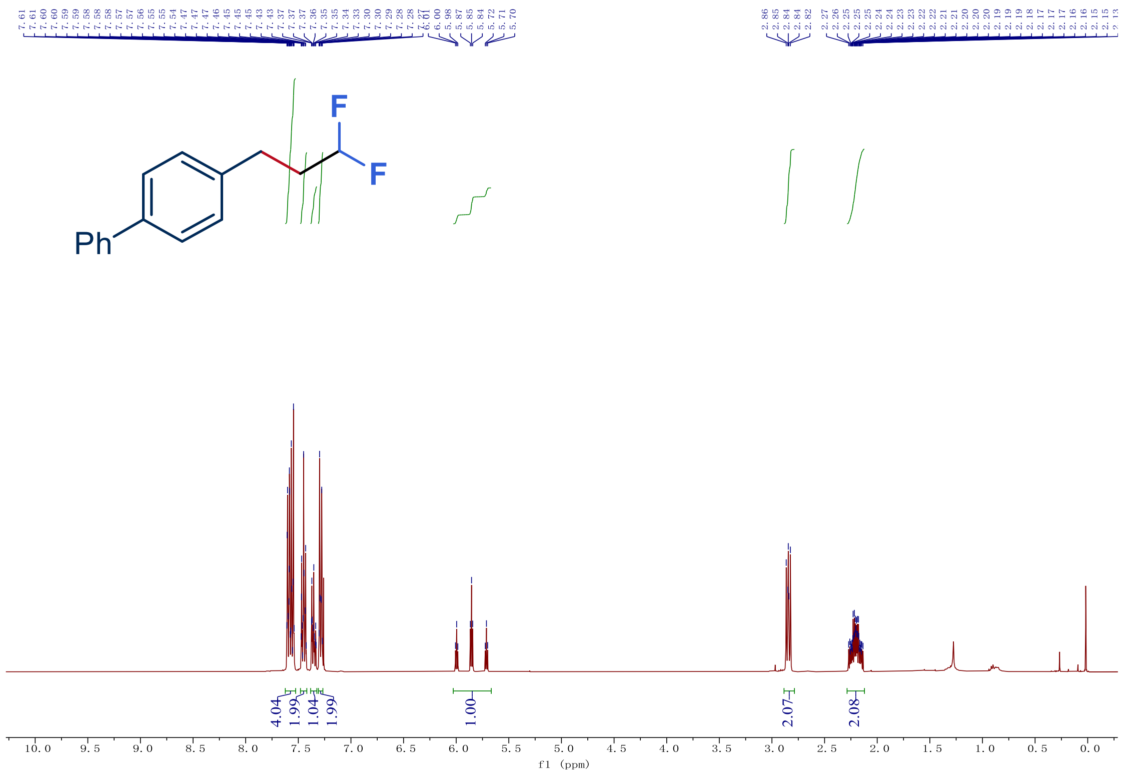

^{19}F NMR (377 MHz, $CDCl_3$) spectra for compound **3b**

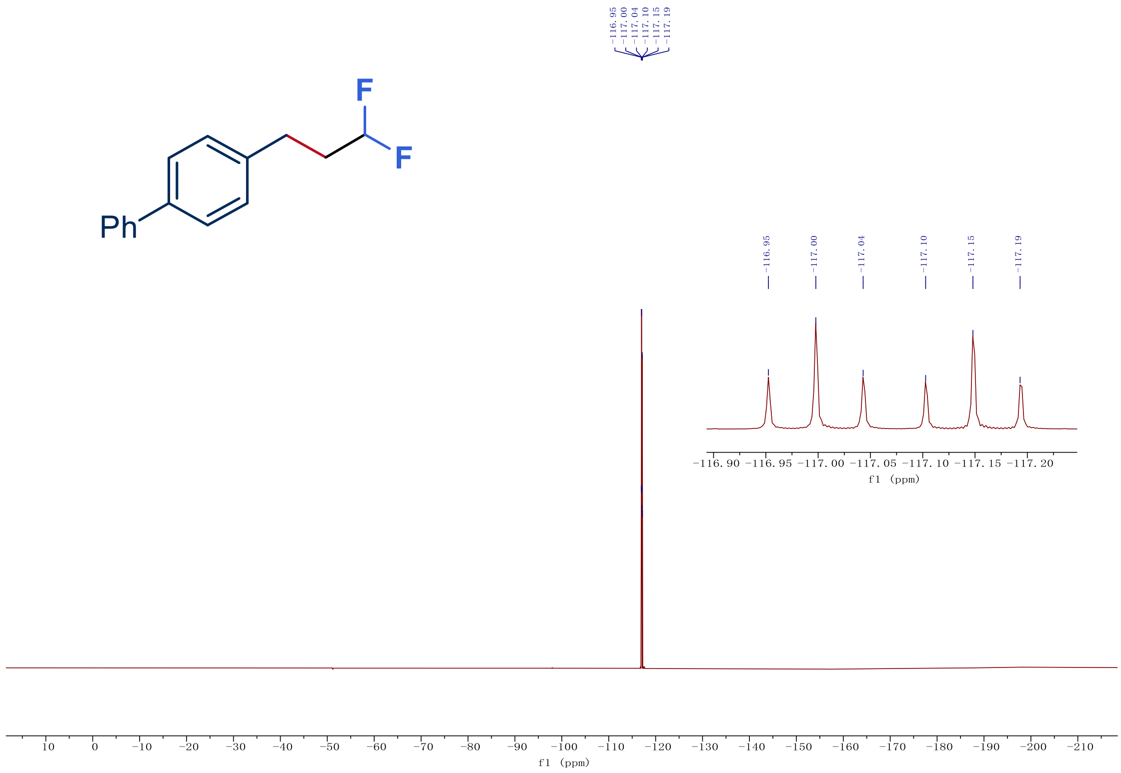

^{13}C NMR (126 MHz, $CDCl_3$) spectra for compound **3b**

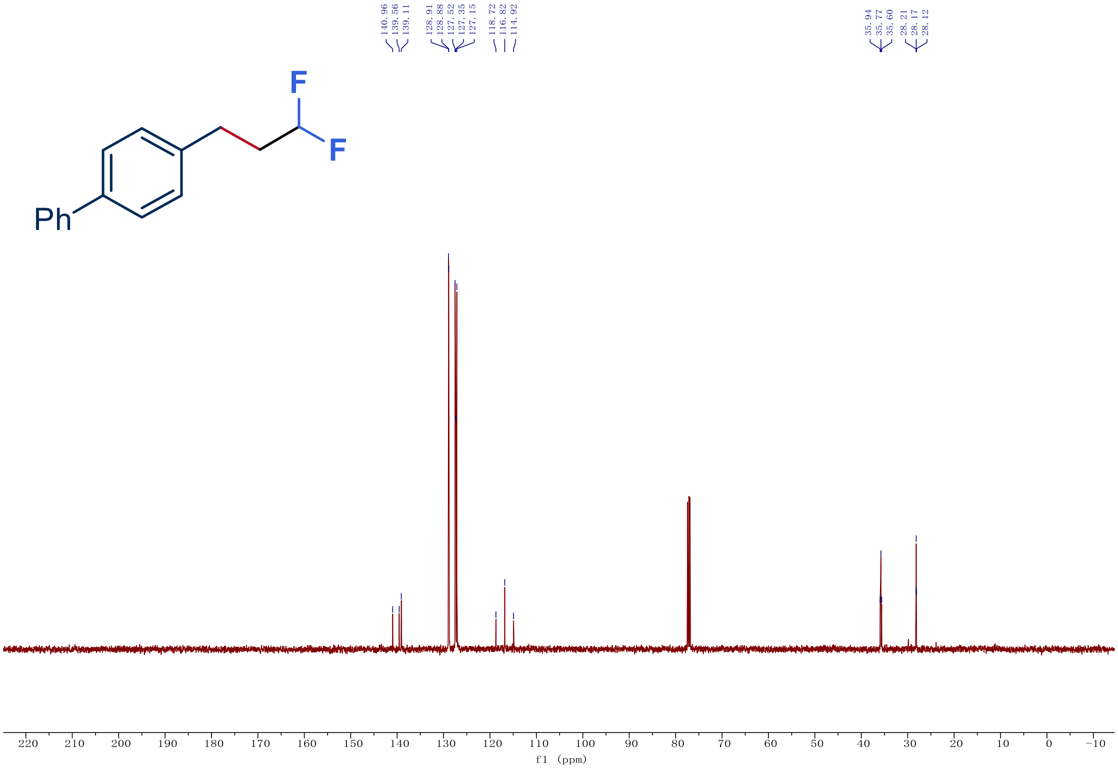

^{1}H NMR (400 MHz, $CDCl_3$) spectra for compound **3c**

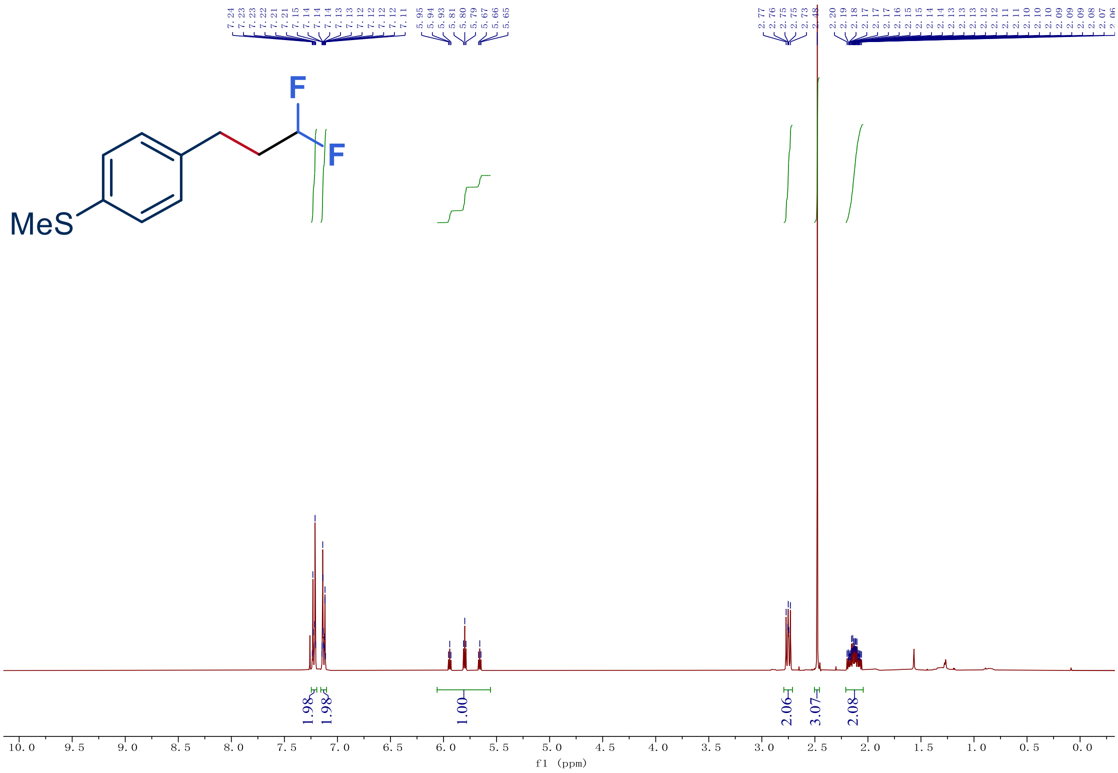

^{19}F NMR (377 MHz, $CDCl_3$) spectra for compound **3c**

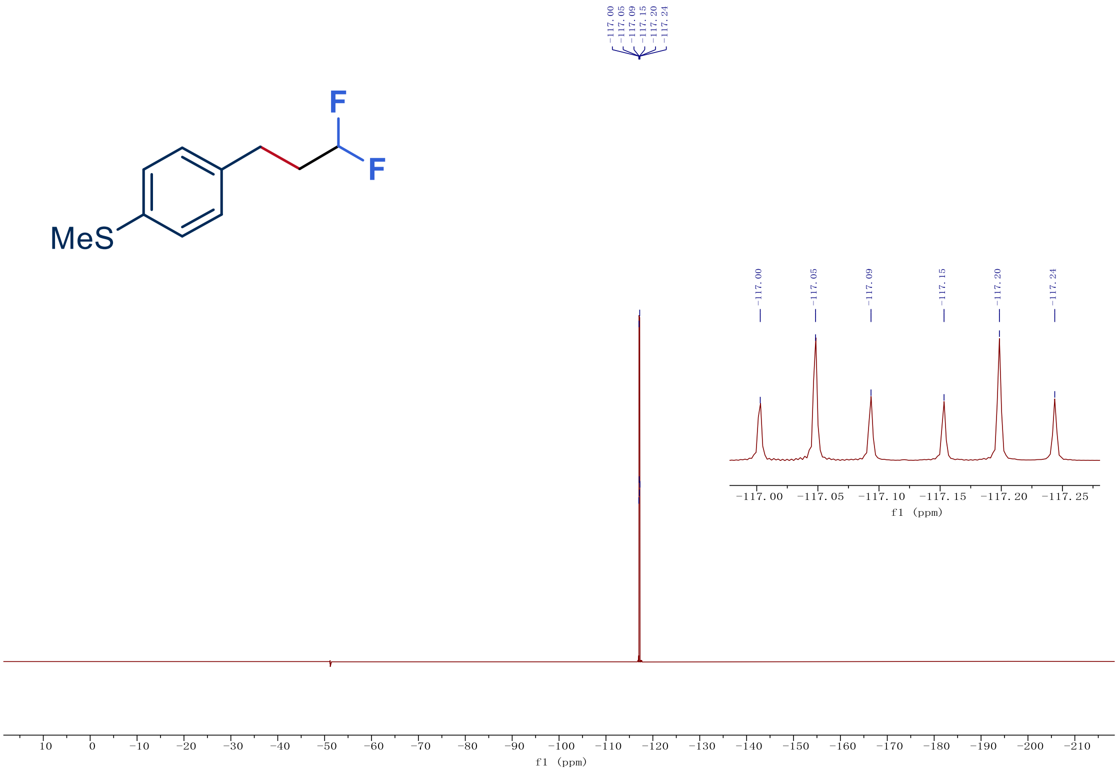

^{13}C NMR (126 MHz, $CDCl_3$) spectra for compound **3c**

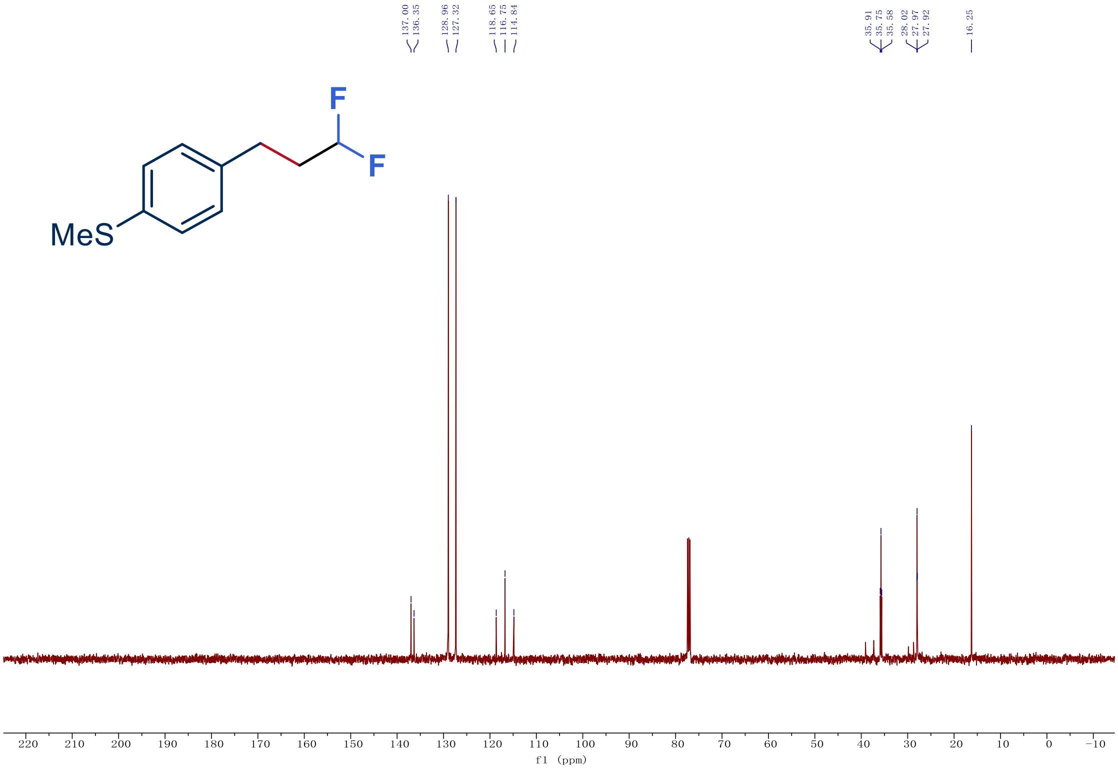

^{1}H NMR (400 MHz, $CDCl_3$) spectra for compound **3d**

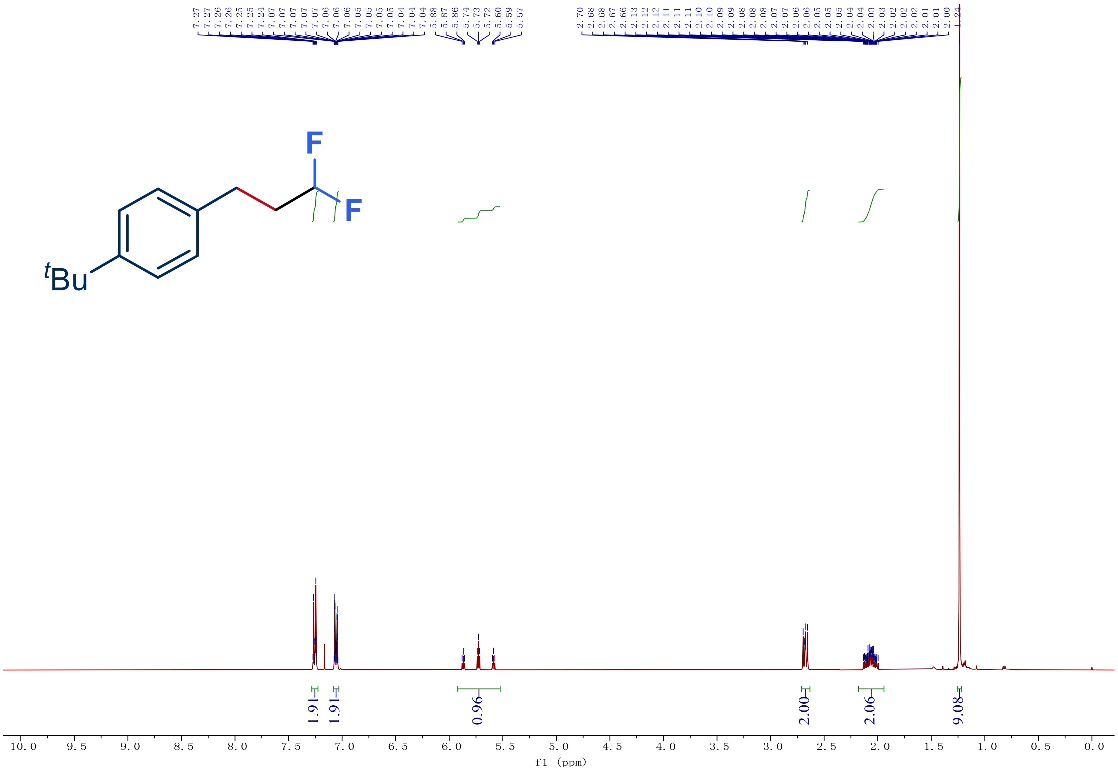

^{19}F NMR (377 MHz, $CDCl_3$) spectra for compound **3d**

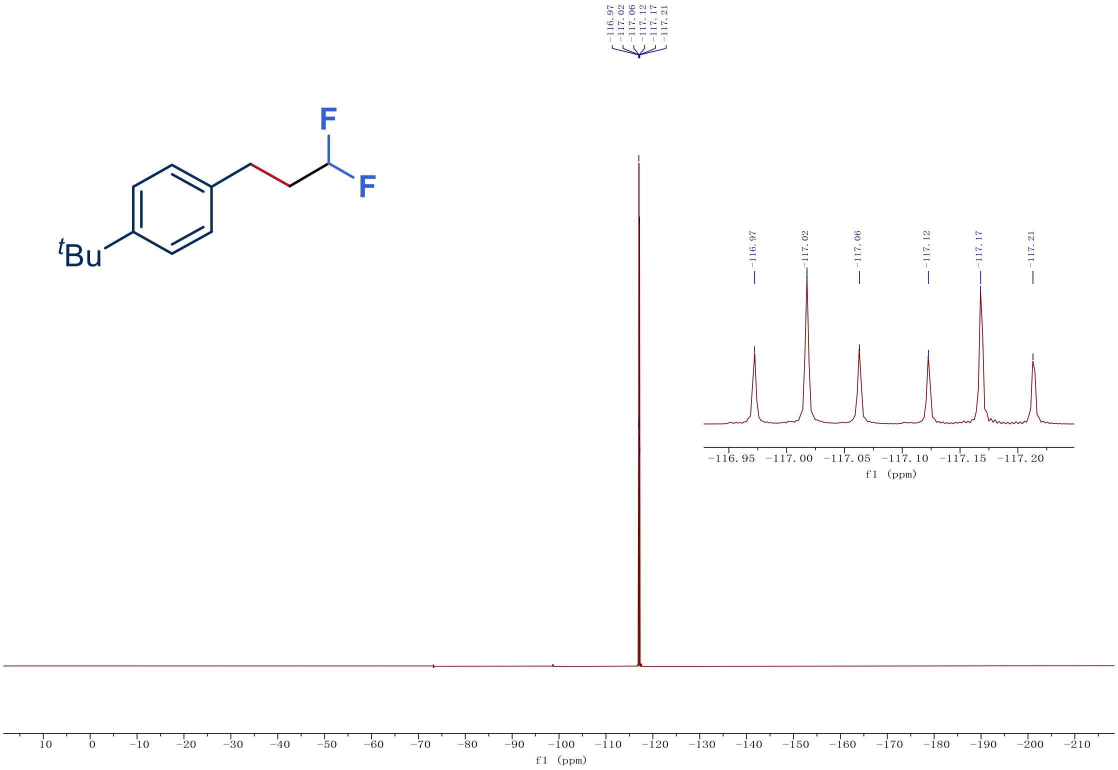

^{13}C NMR (126 MHz, $CDCl_3$) spectra for compound **3d**

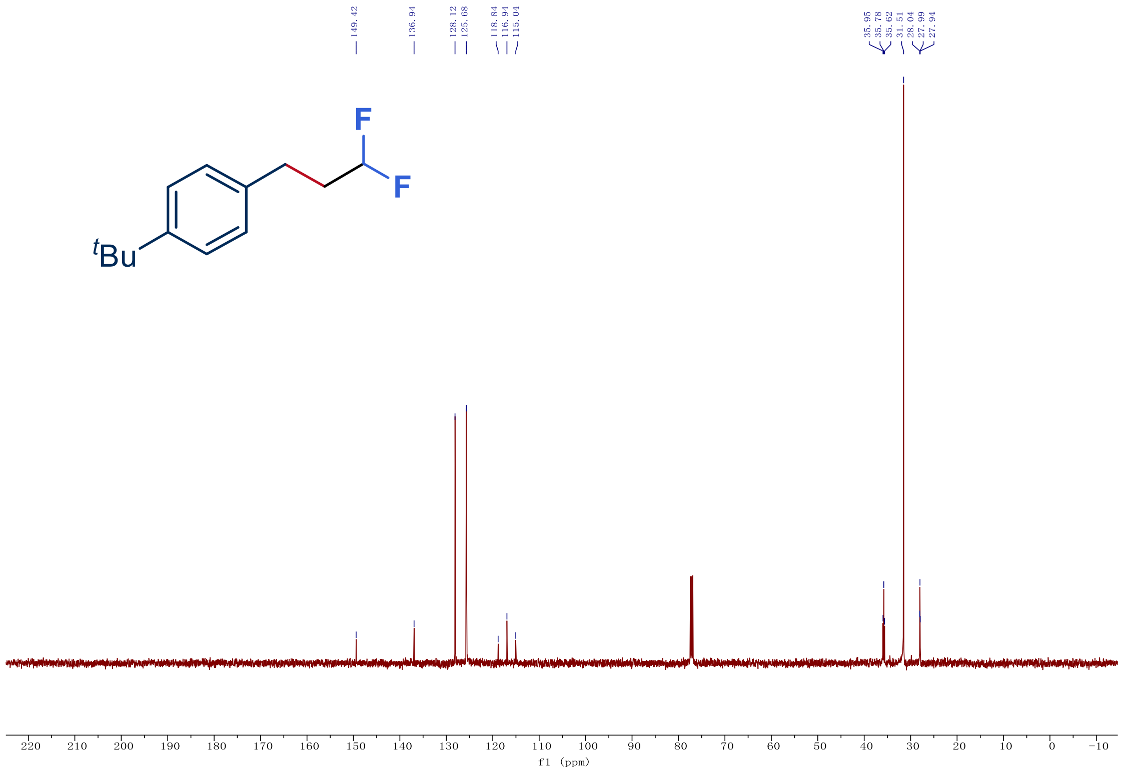

^{1}H NMR (400 MHz, $CDCl_3$) spectra for compound **3e**

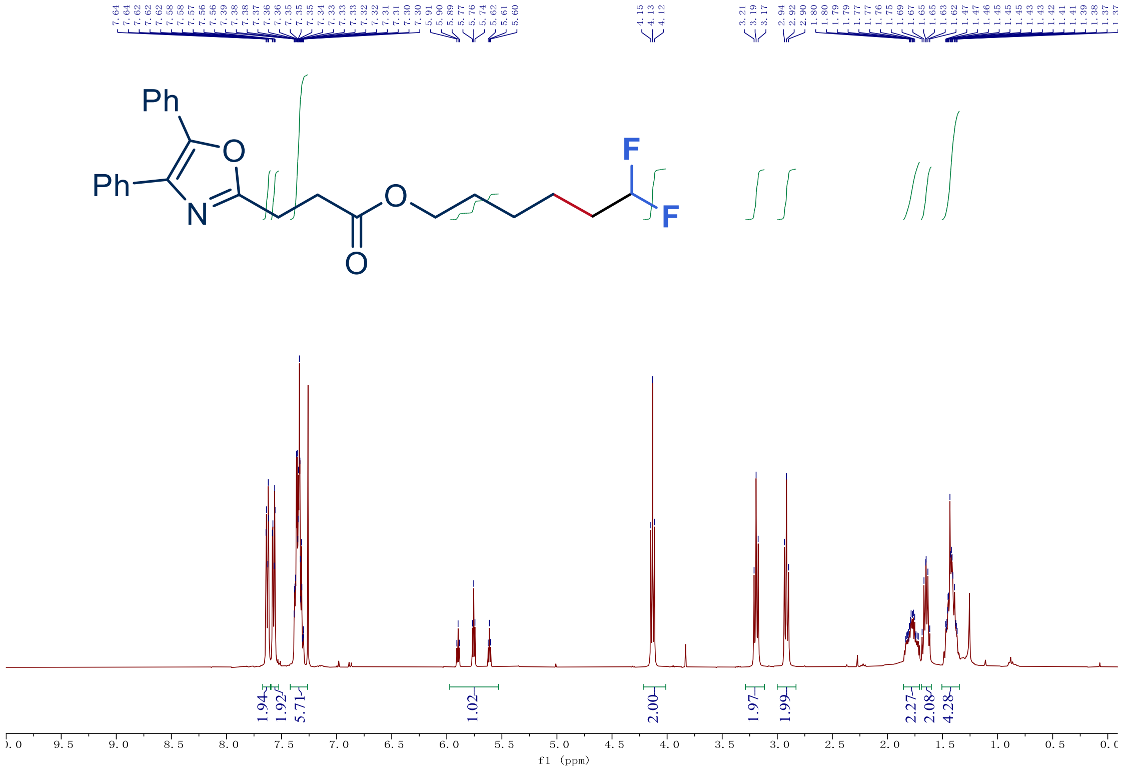

^{19}F NMR (377 MHz, $CDCl_3$) spectra for compound **3e**

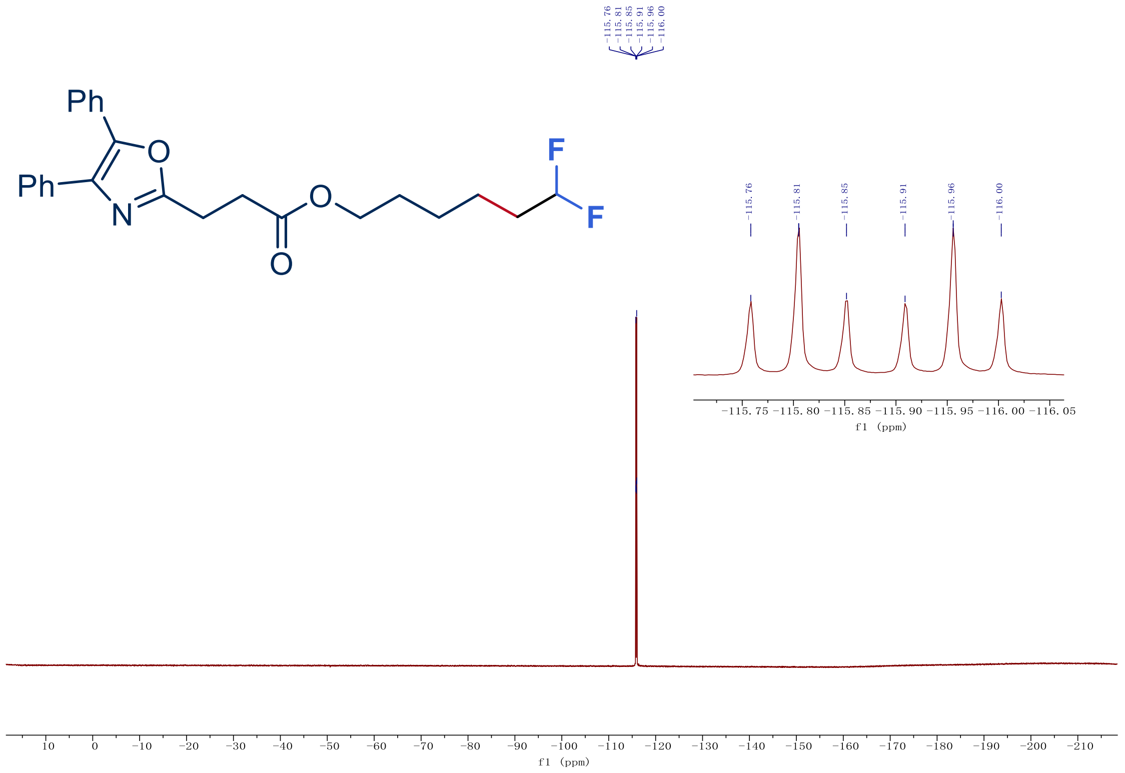

^{13}C NMR (126 MHz, $CDCl_3$) spectra for compound **3e**

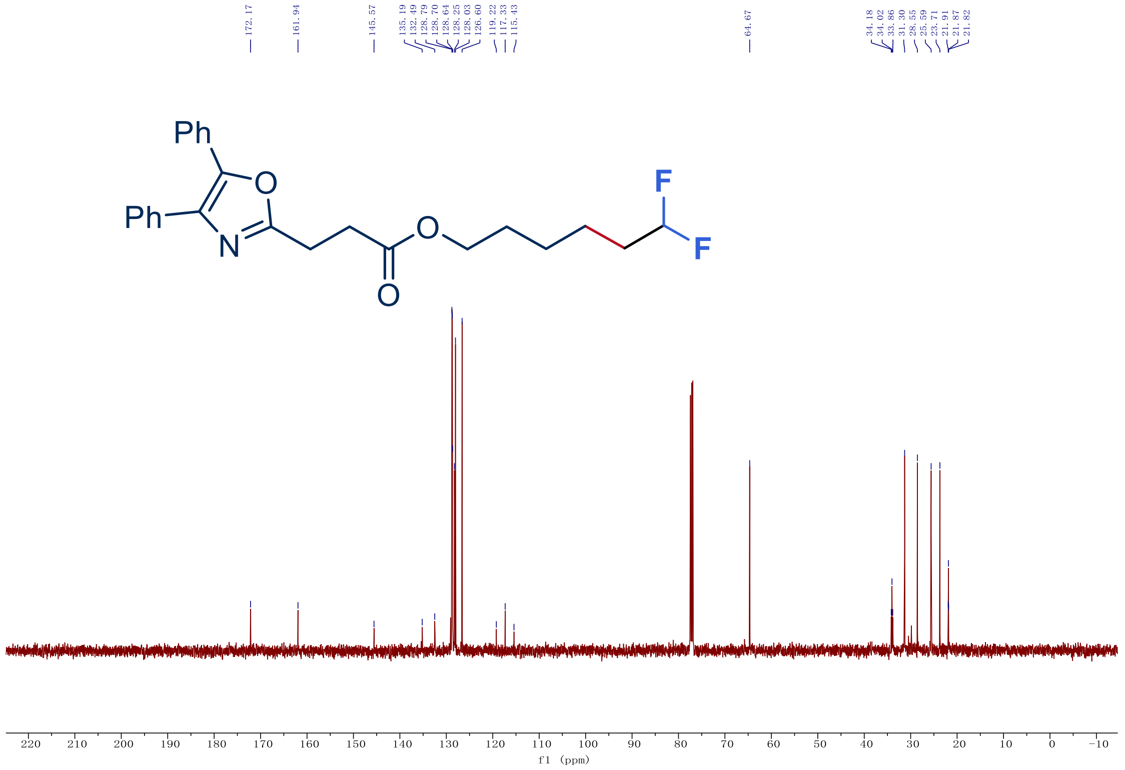

^{1}H NMR (400 MHz, $CDCl_3$) spectra for compound **3f**

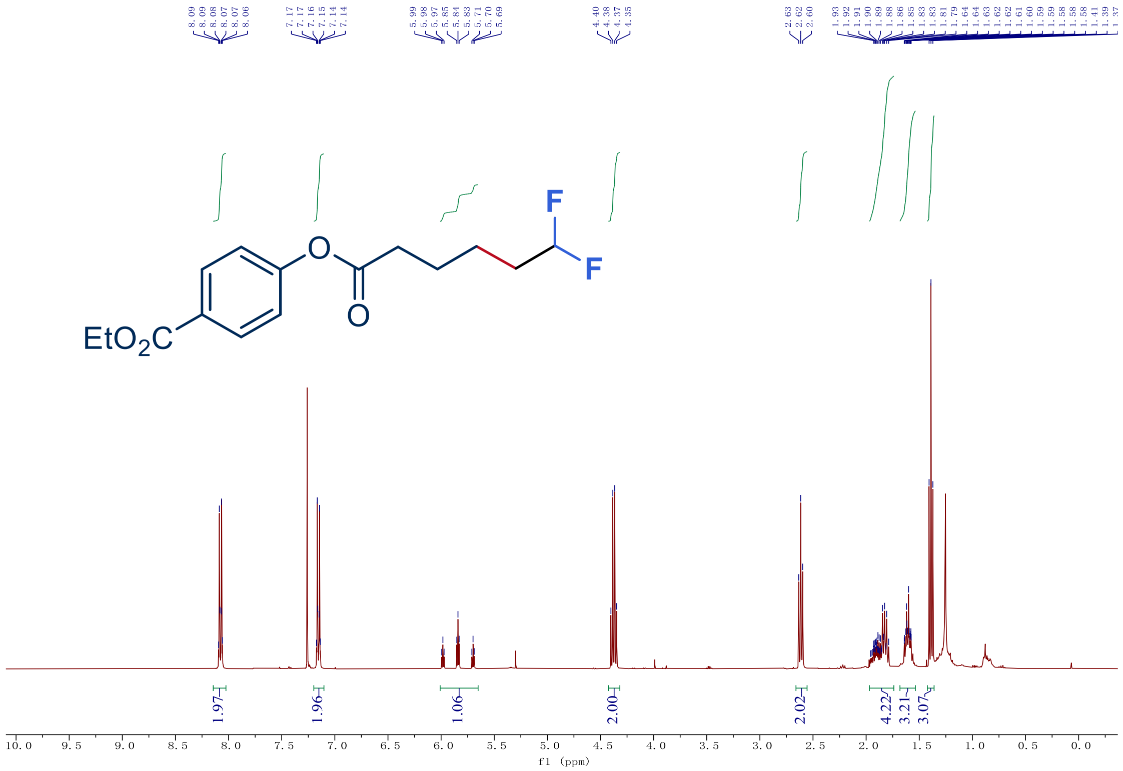

^{19}F NMR (377 MHz, $CDCl_3$) spectra for compound **3f**

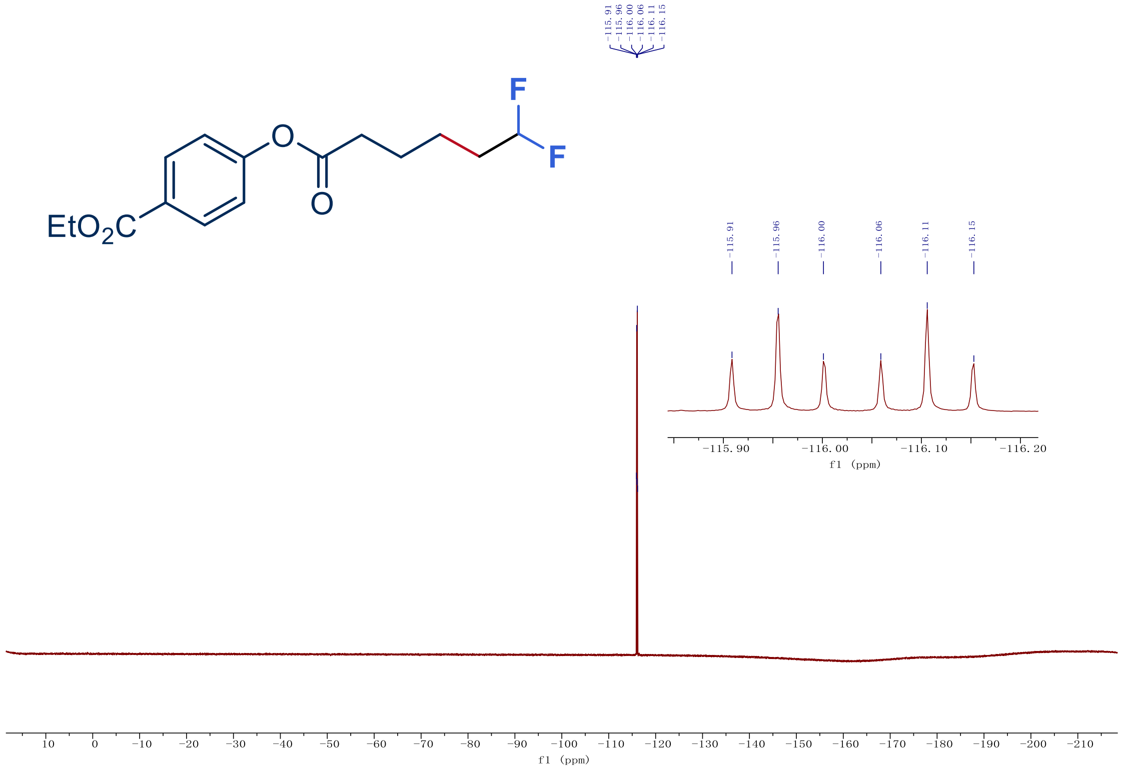

^{1}H NMR (400 MHz, $CDCl_3$) spectra for compound **3g**

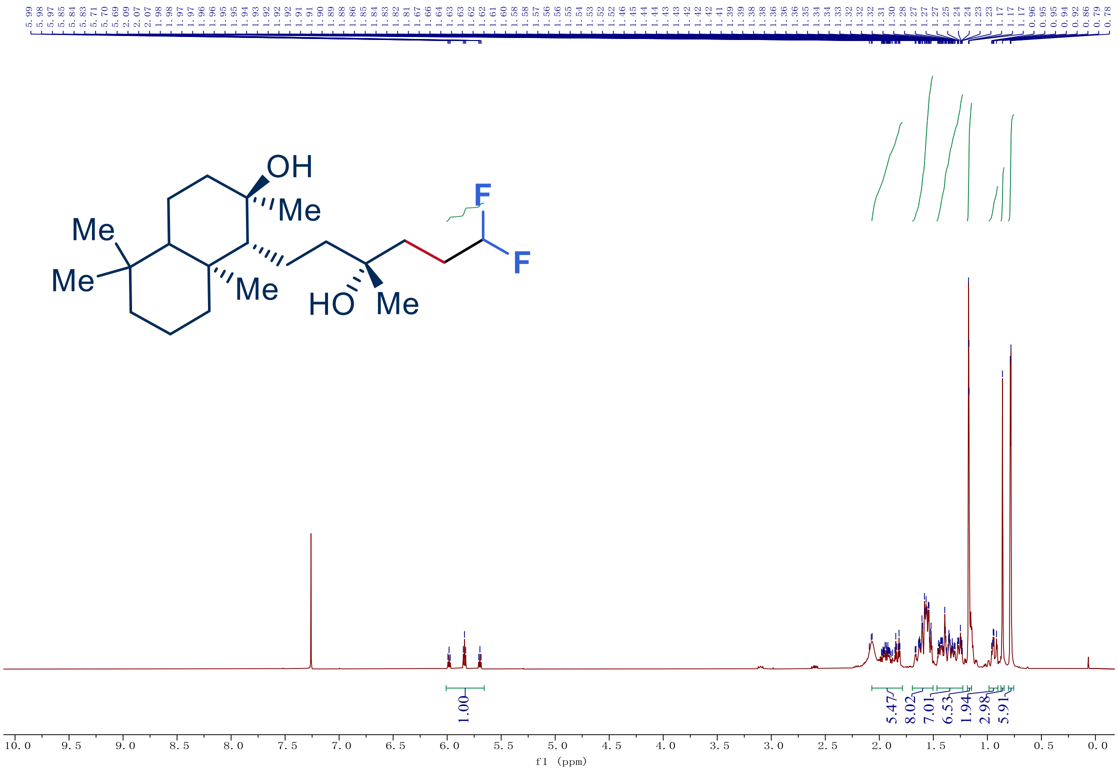

^{19}F NMR (377 MHz, $CDCl_3$) spectra for compound **3g**

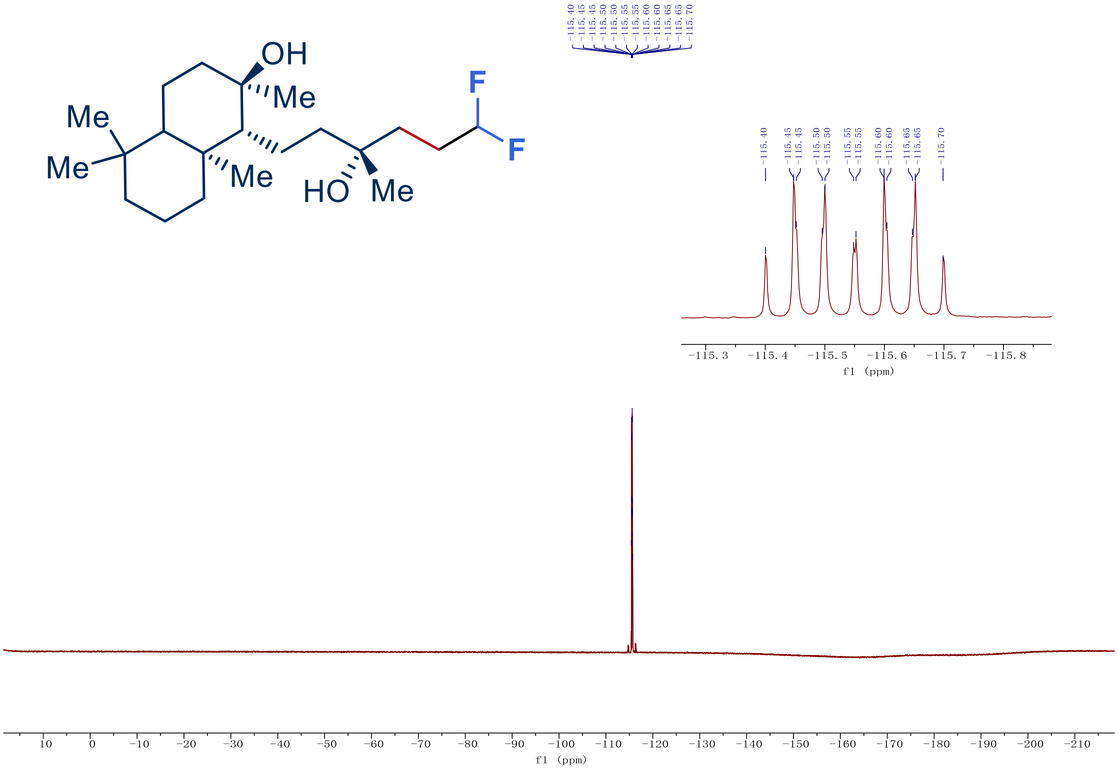

^{1}H NMR (400 MHz, $CDCl_3$) spectra for compound **3h**

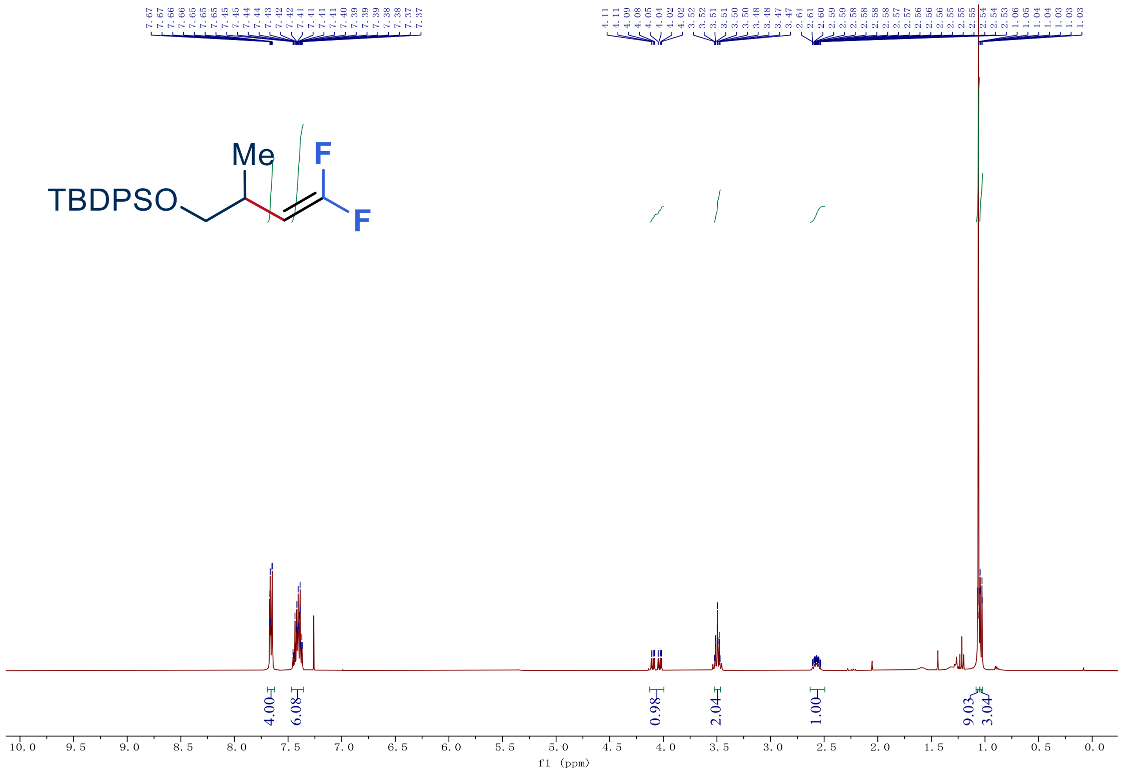

^{19}F NMR (377 MHz, $CDCl_3$) spectra for compound **3h**

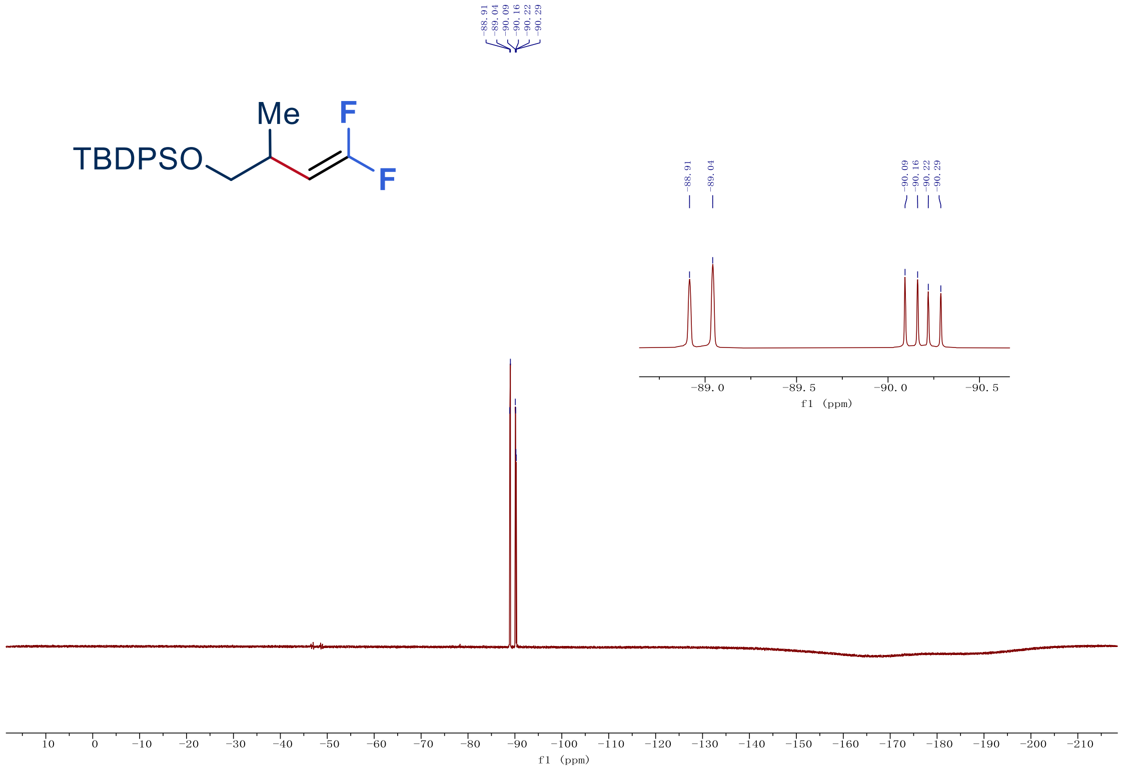

^{13}C NMR (126 MHz, $CDCl_3$) spectra for compound **3h**

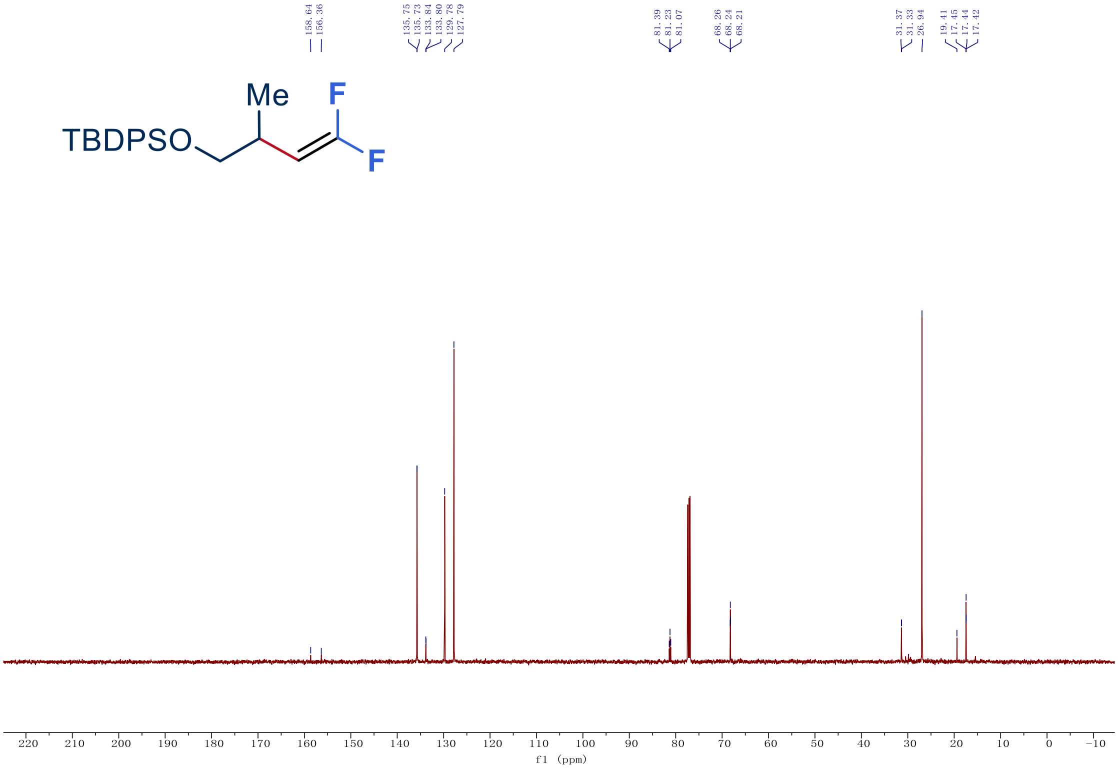

^{1}H NMR (400 MHz, $CDCl_3$) spectra for compound **3i**

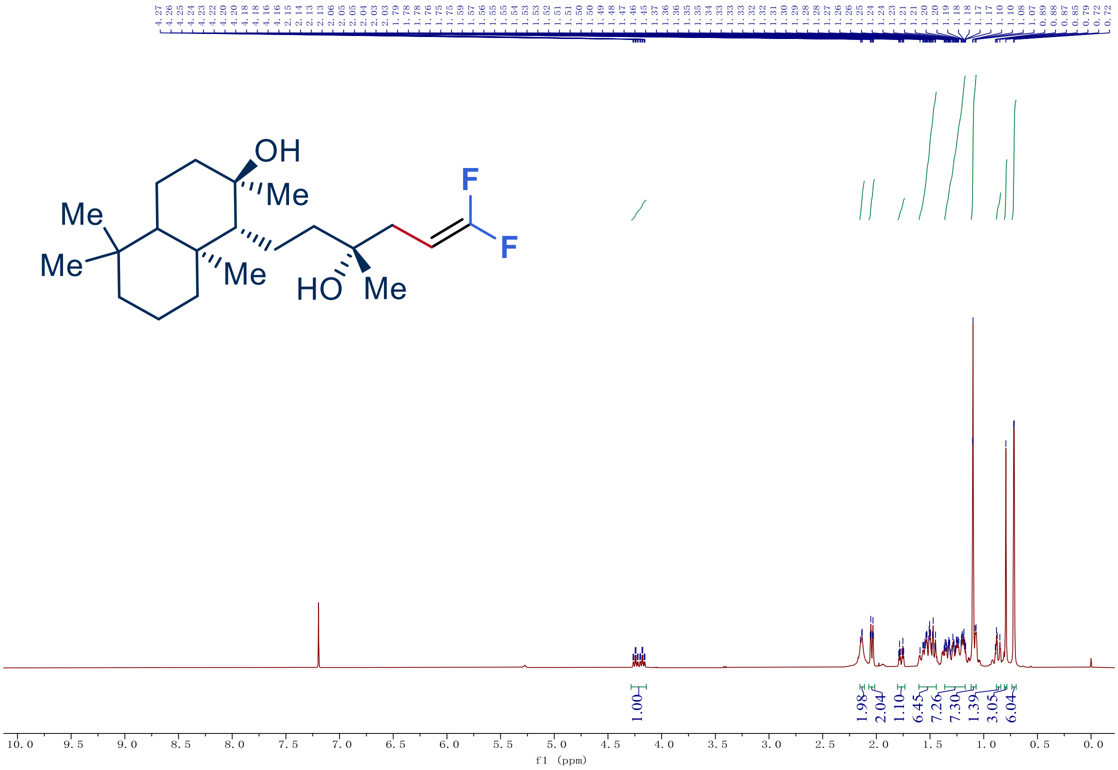

^{19}F NMR (377 MHz, $CDCl_3$) spectra for compound **3i**

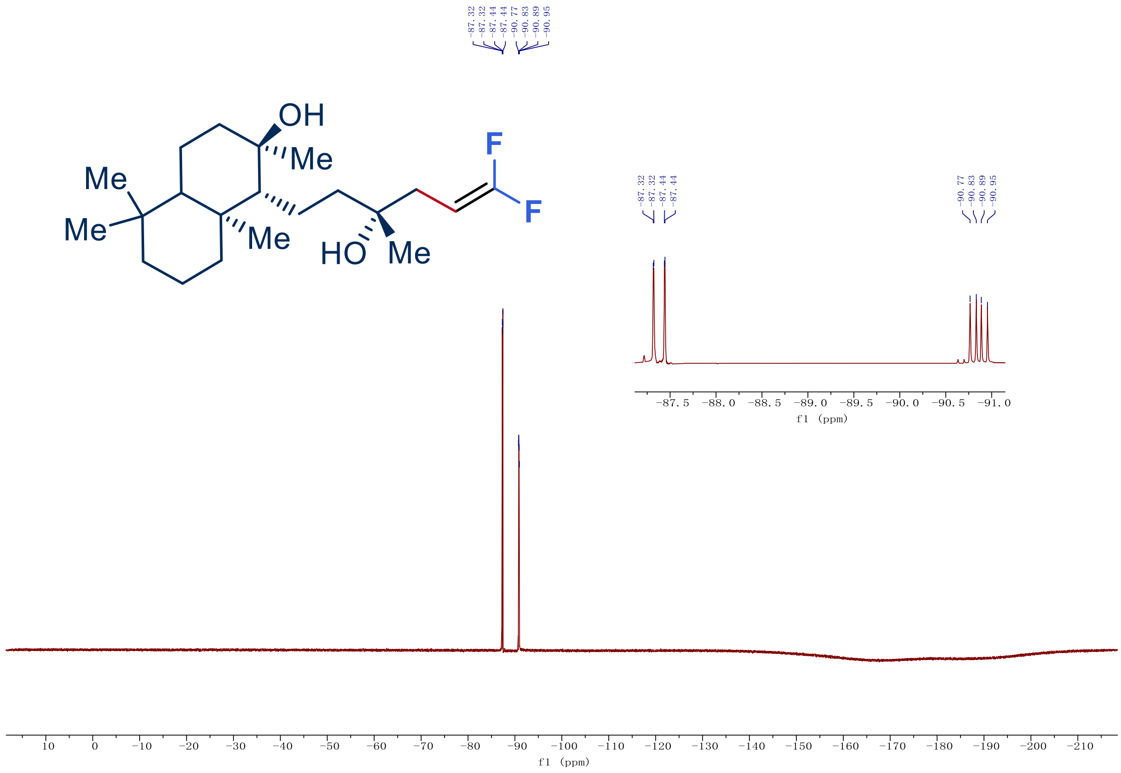

^{13}C NMR (126 MHz, $CDCl_3$) spectra for compound **3i**

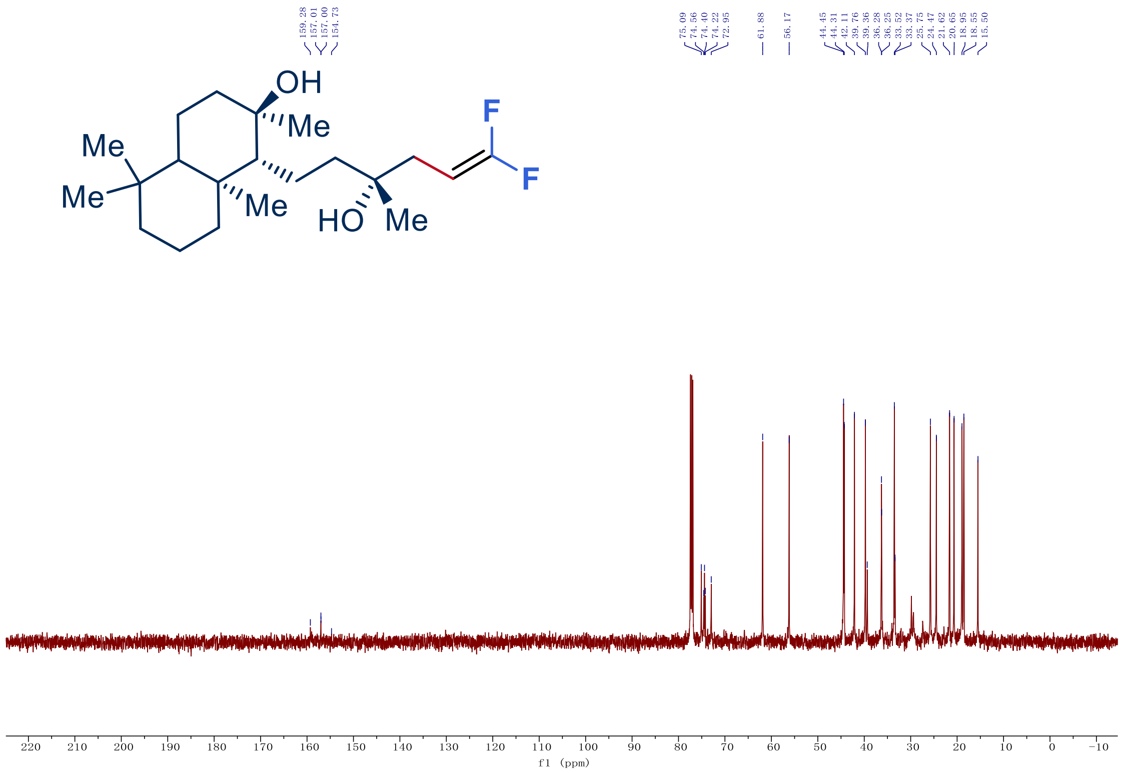

^{1}H NMR (400 MHz, $CDCl_3$) spectra for compound **3j**

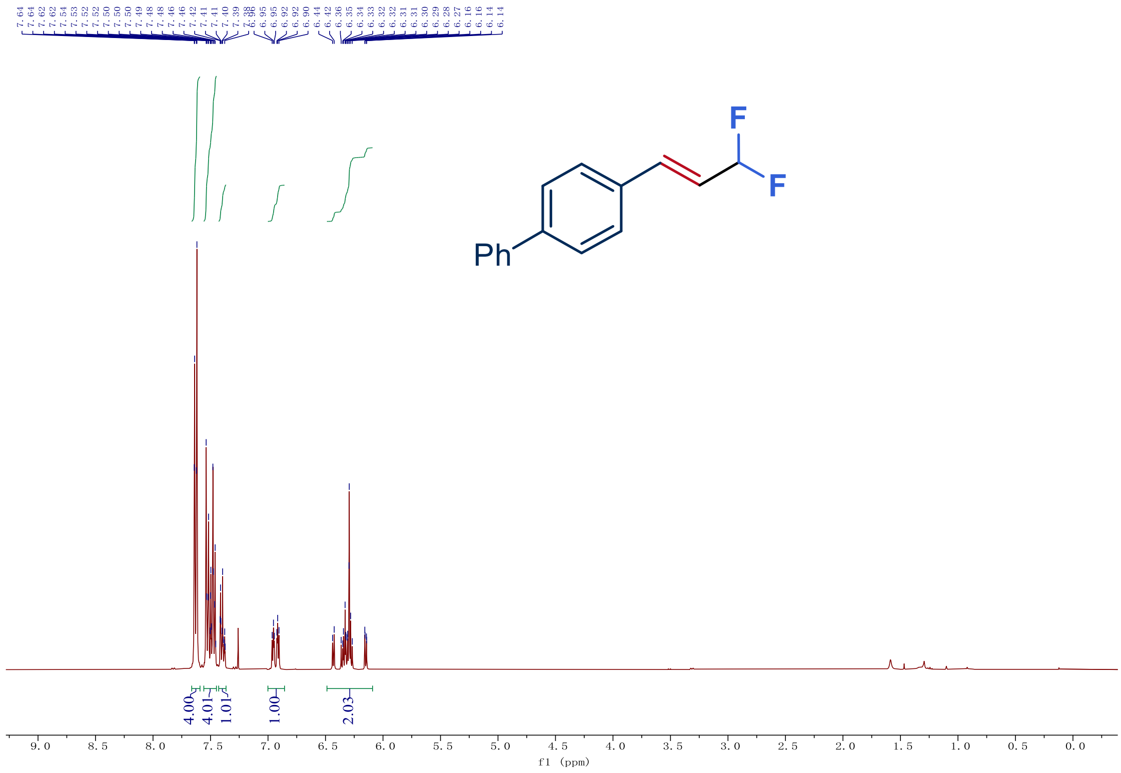

^{19}F NMR (377 MHz, $CDCl_3$) spectra for compound **2j**

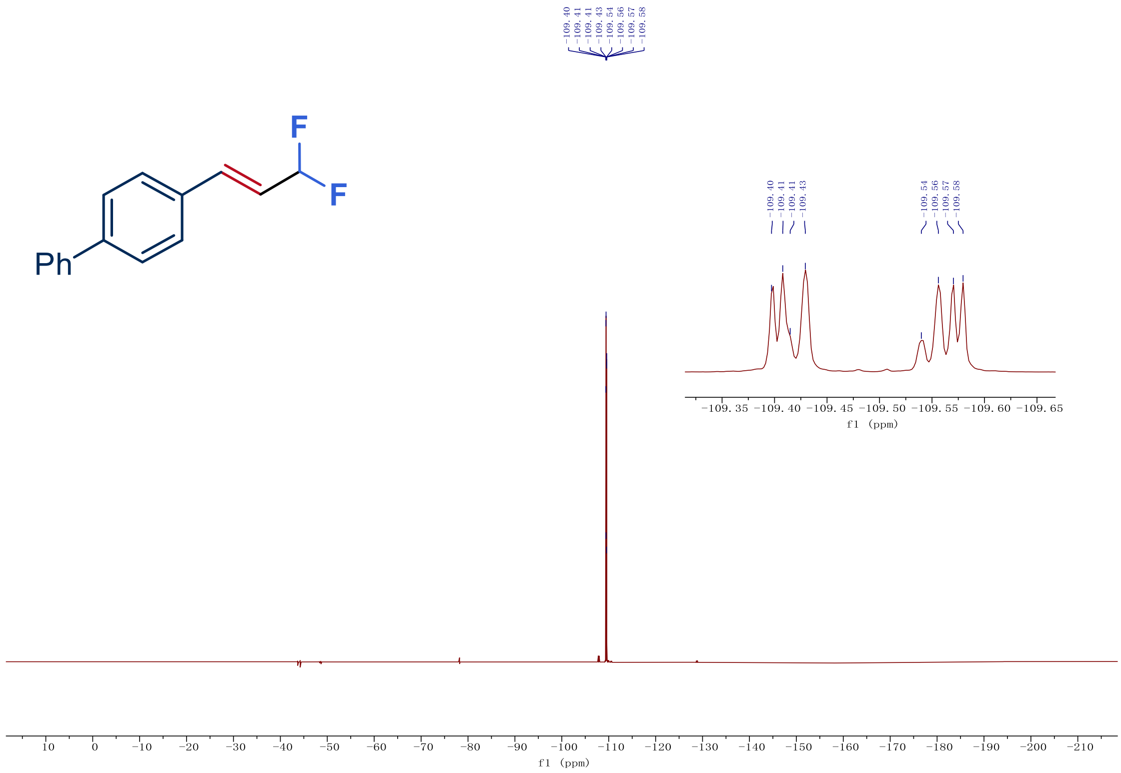

^{13}C NMR (126 MHz, CDCl$_3$) spectra for compound **3j**

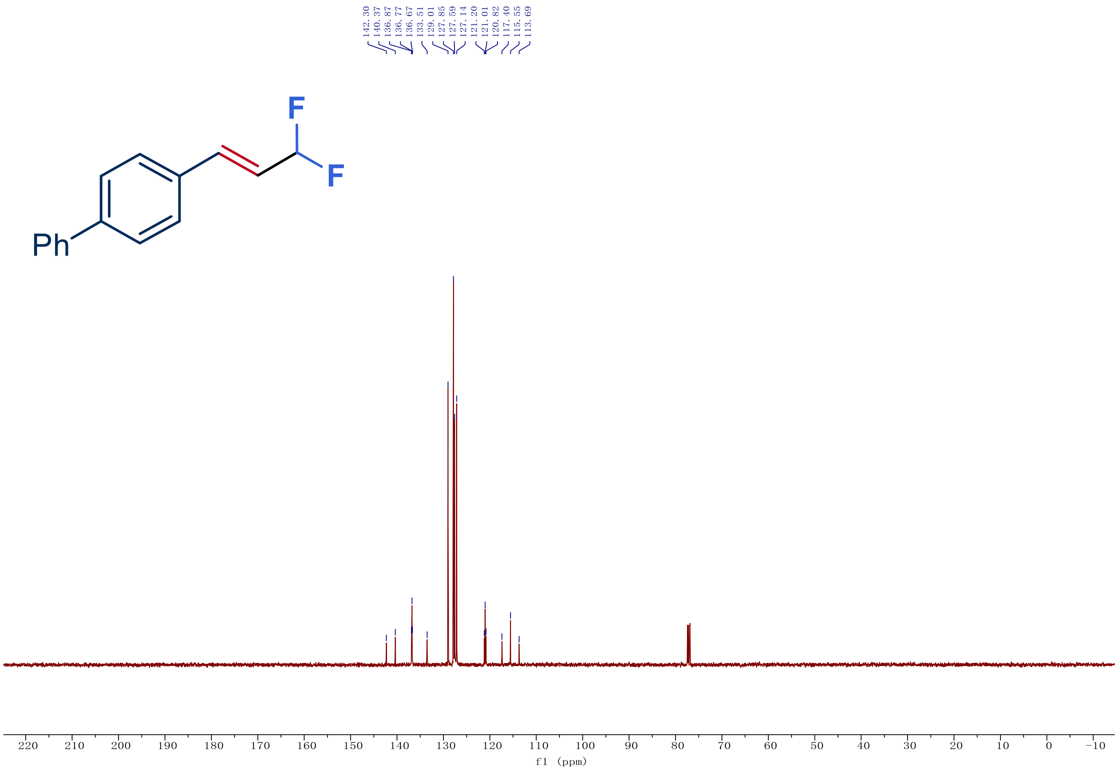

^{1}H NMR (400 MHz, CDCl$_3$) spectra for compound **3k**

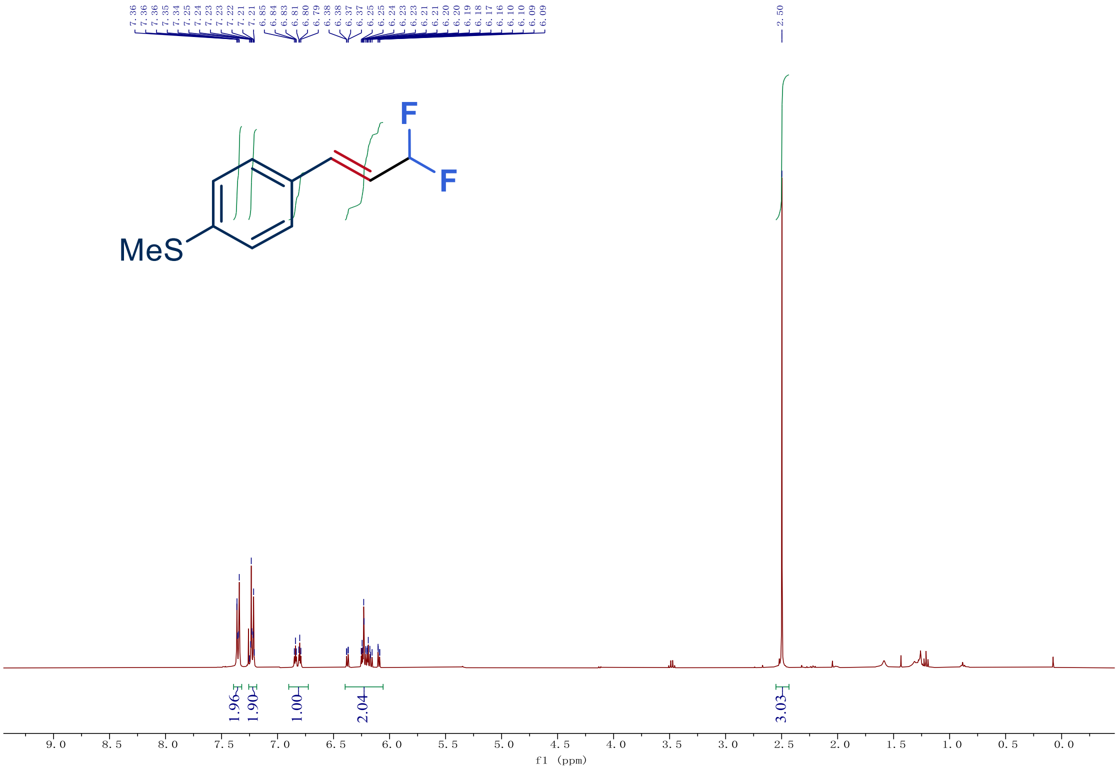

^{19}F NMR (377 MHz, $CDCl_3$) spectra for compound **3k**

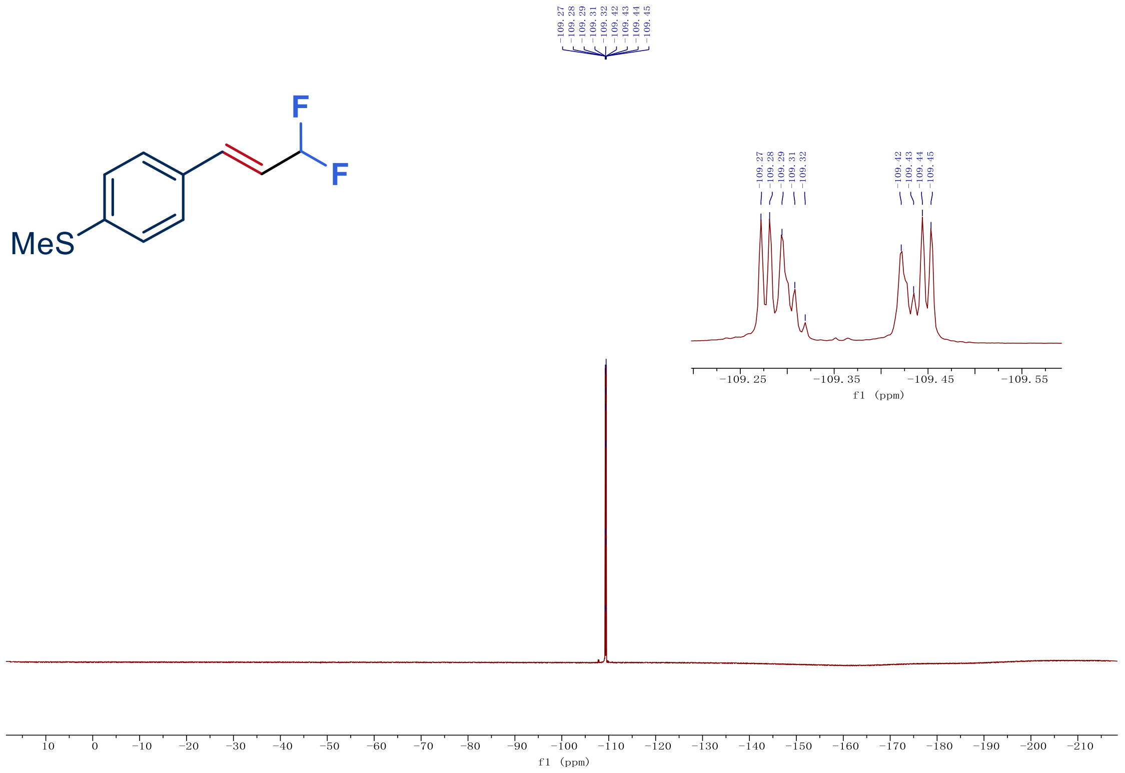

^{13}C NMR (126 MHz, $CDCl_3$) spectra for compound **3k**

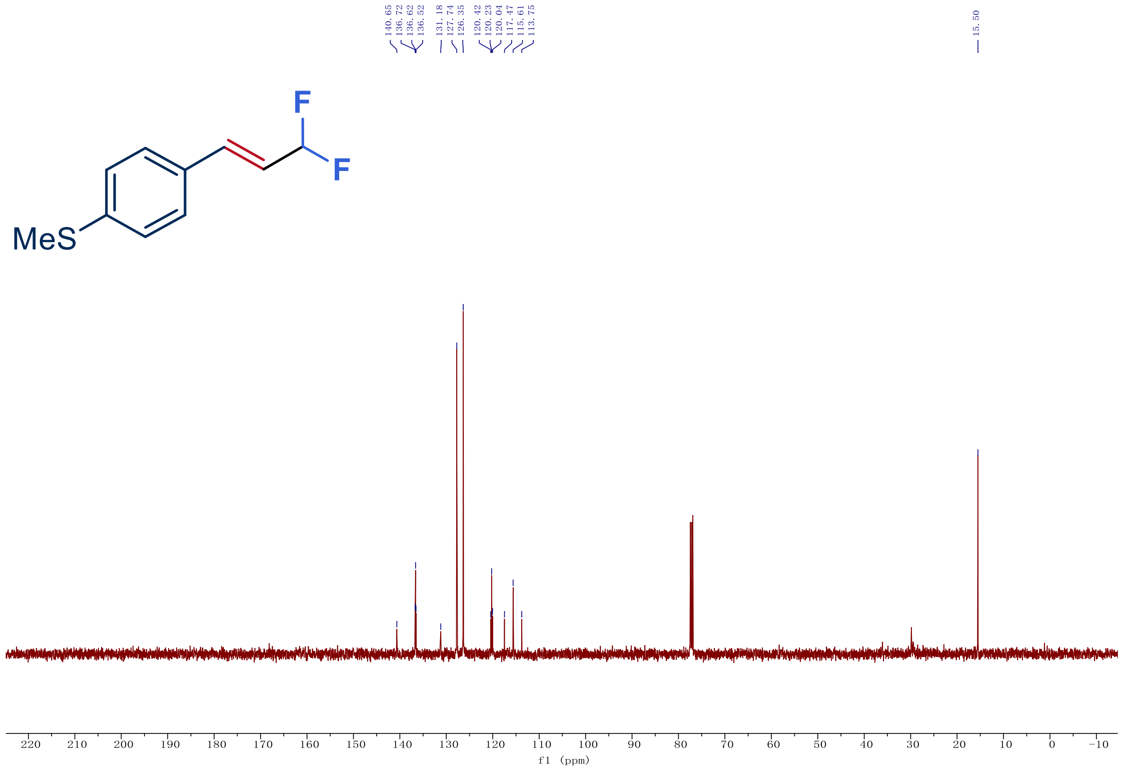

^{1}H NMR (400 MHz, $CDCl_3$) spectra for compound **3l**

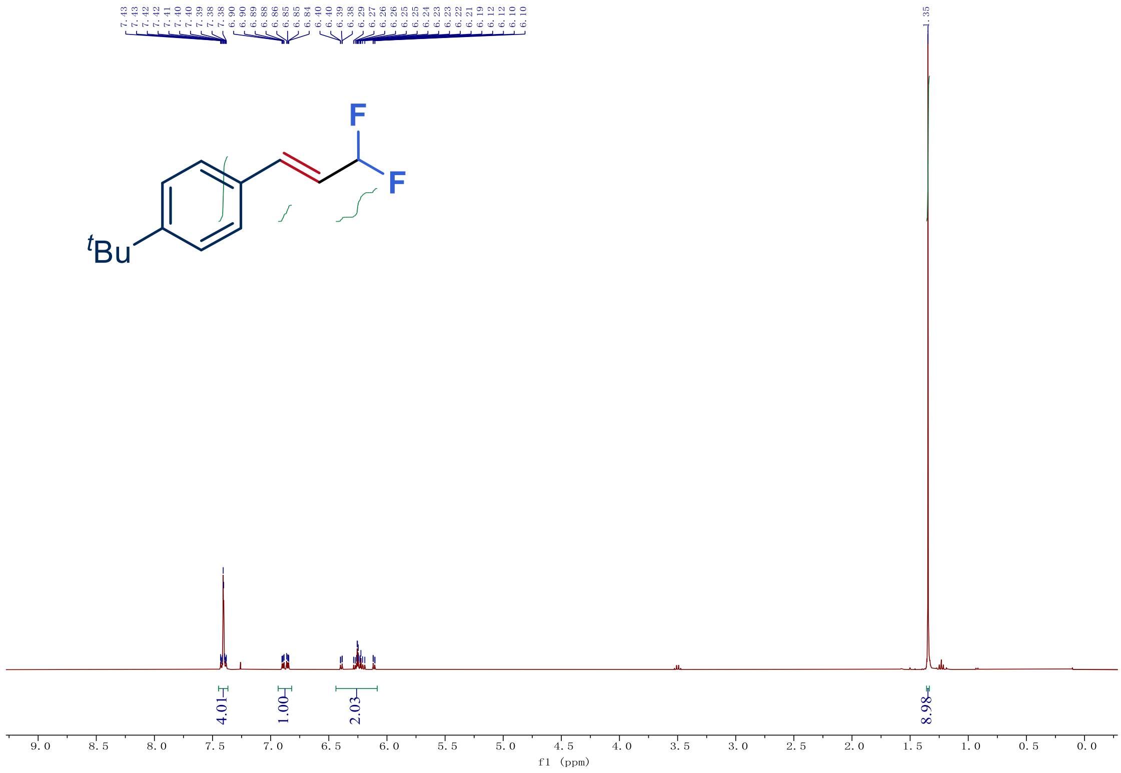

^{19}F NMR (377 MHz, $CDCl_3$) spectra for compound **3l**

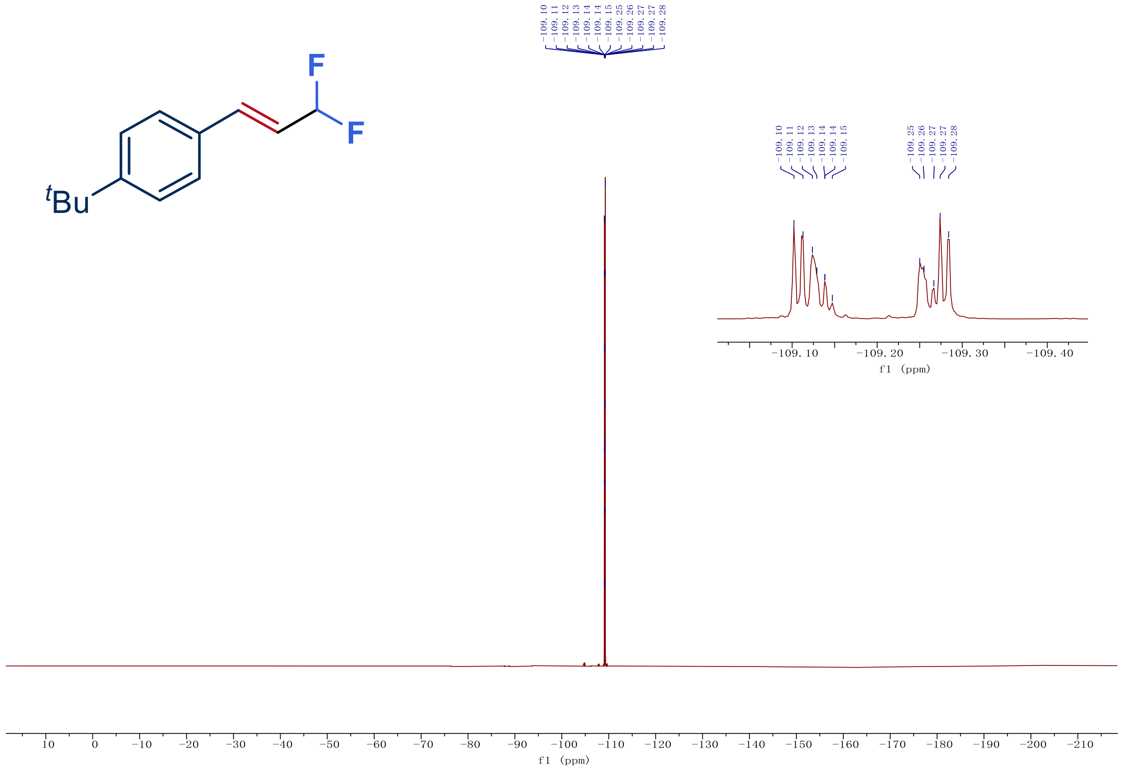

^{13}C NMR (126 MHz, $CDCl_3$) spectra for compound **3l**

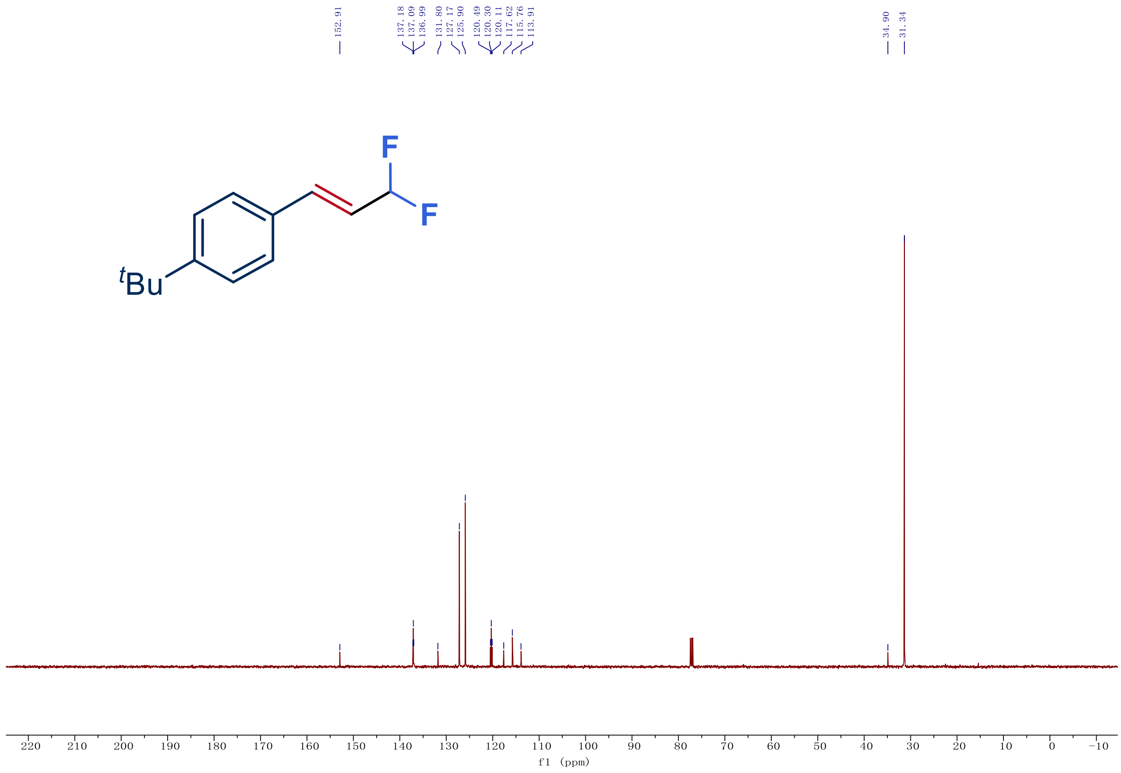

^{1}H NMR (400 MHz, $CDCl_3$) spectra for compound **3m**

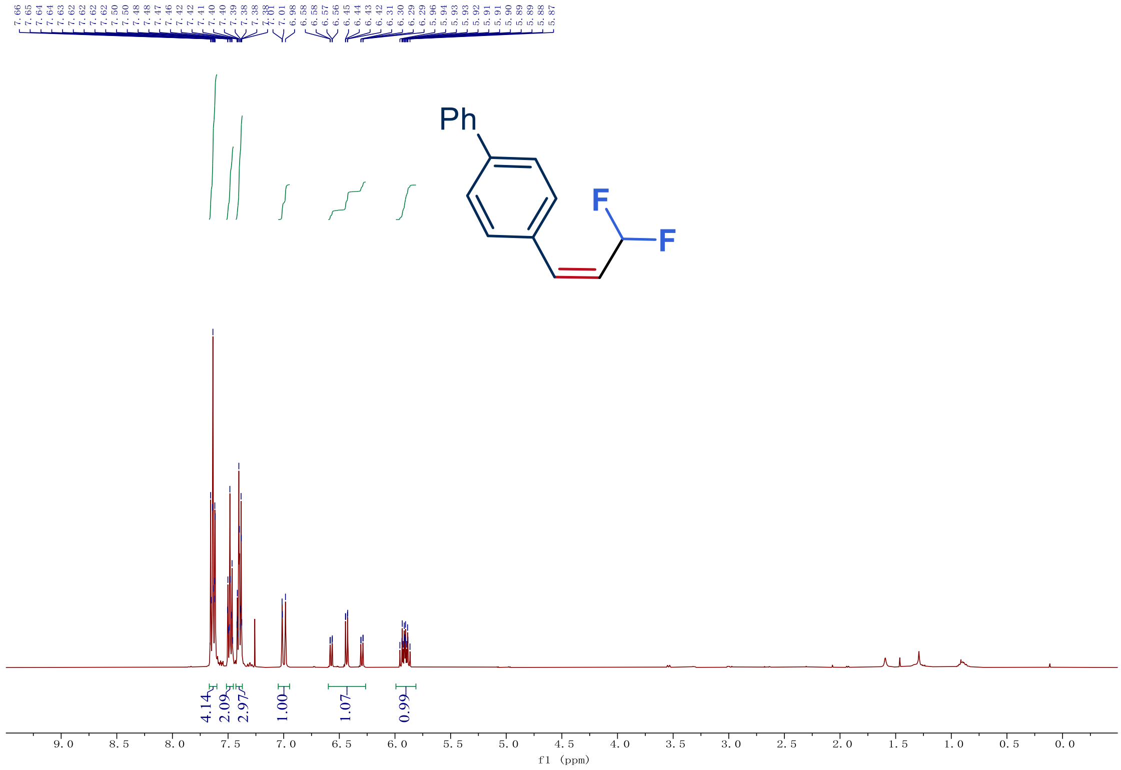

^{19}F NMR (377 MHz, $CDCl_3$) spectra for compound **3m**

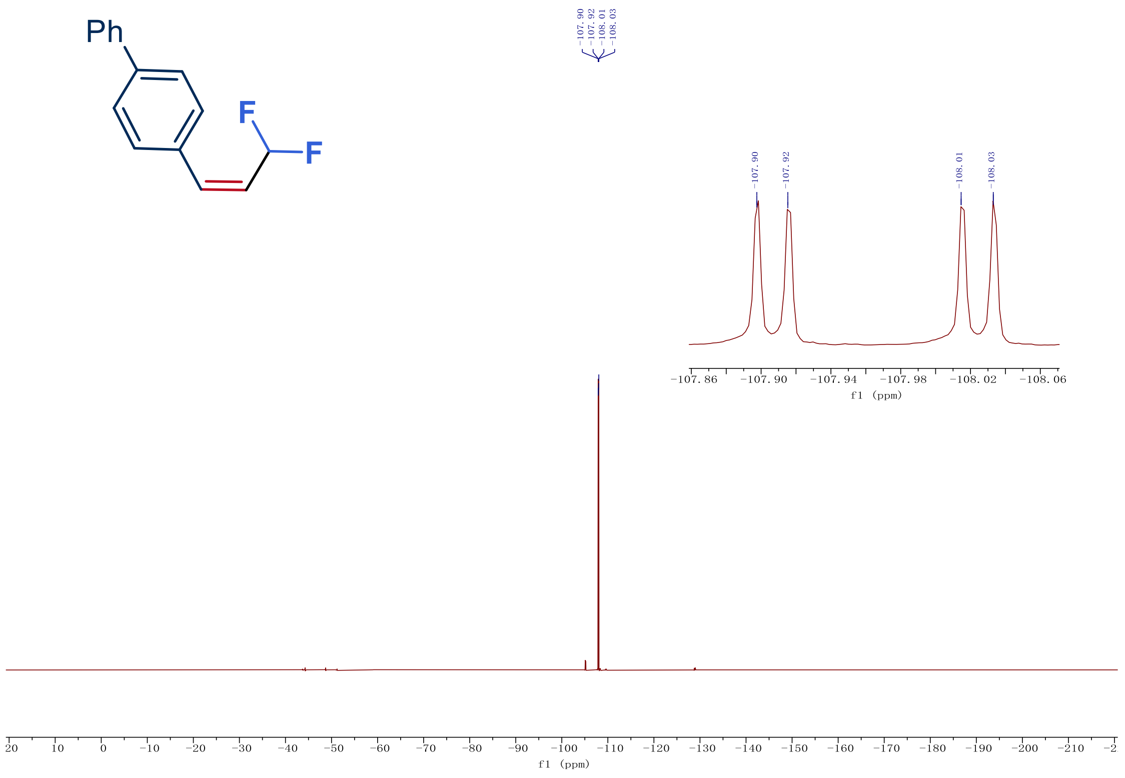

^{13}C NMR (126 MHz, $CDCl_3$) spectra for compound **3m**

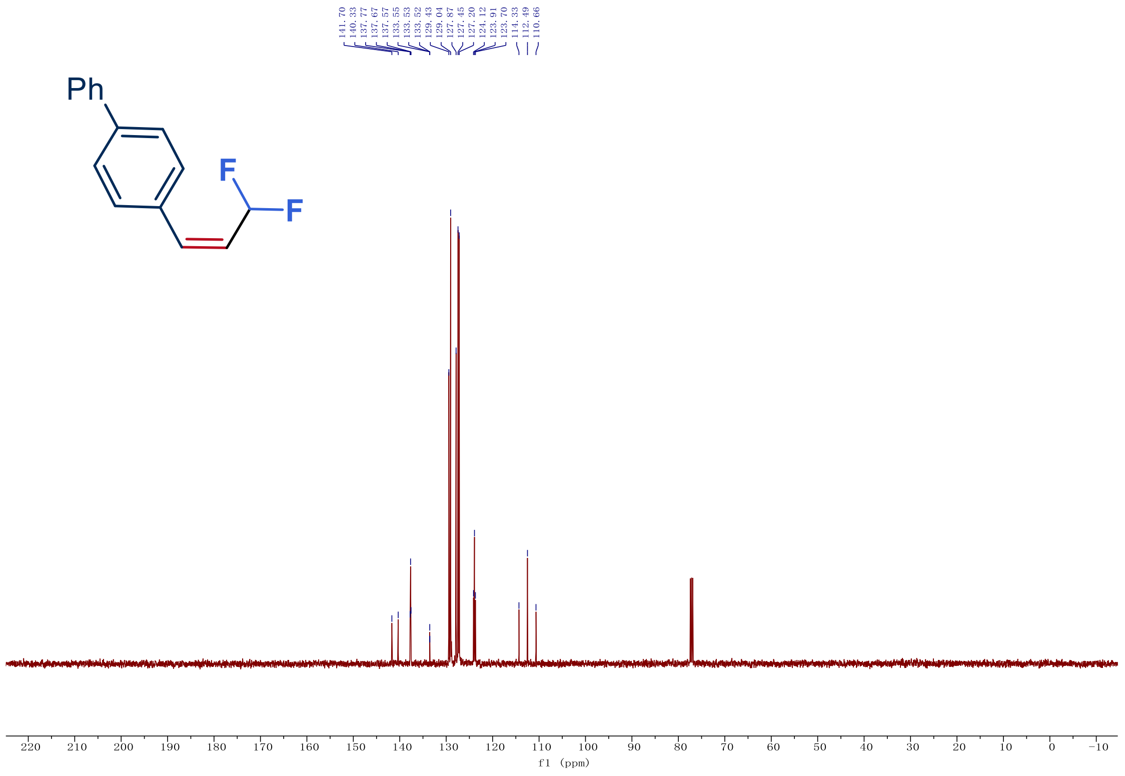

^{1}H NMR (400 MHz, $CDCl_3$) spectra for compound **3n**

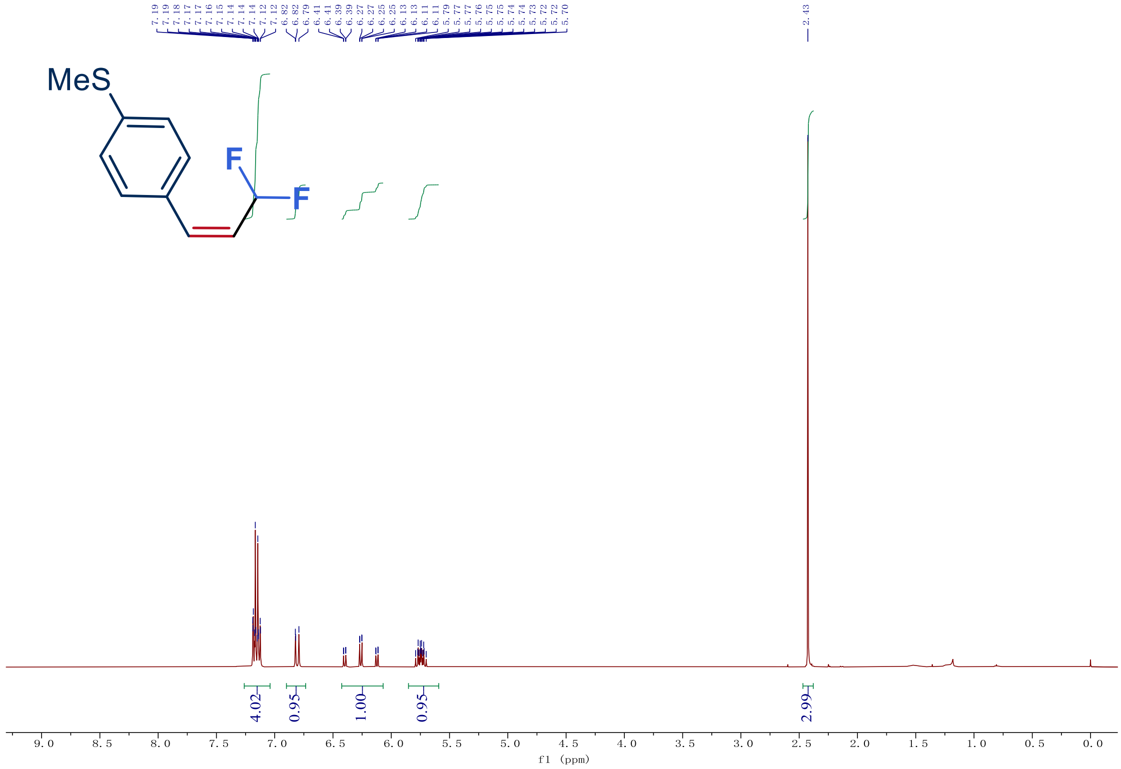

^{19}F NMR (377 MHz, $CDCl_3$) spectra for compound **3n**

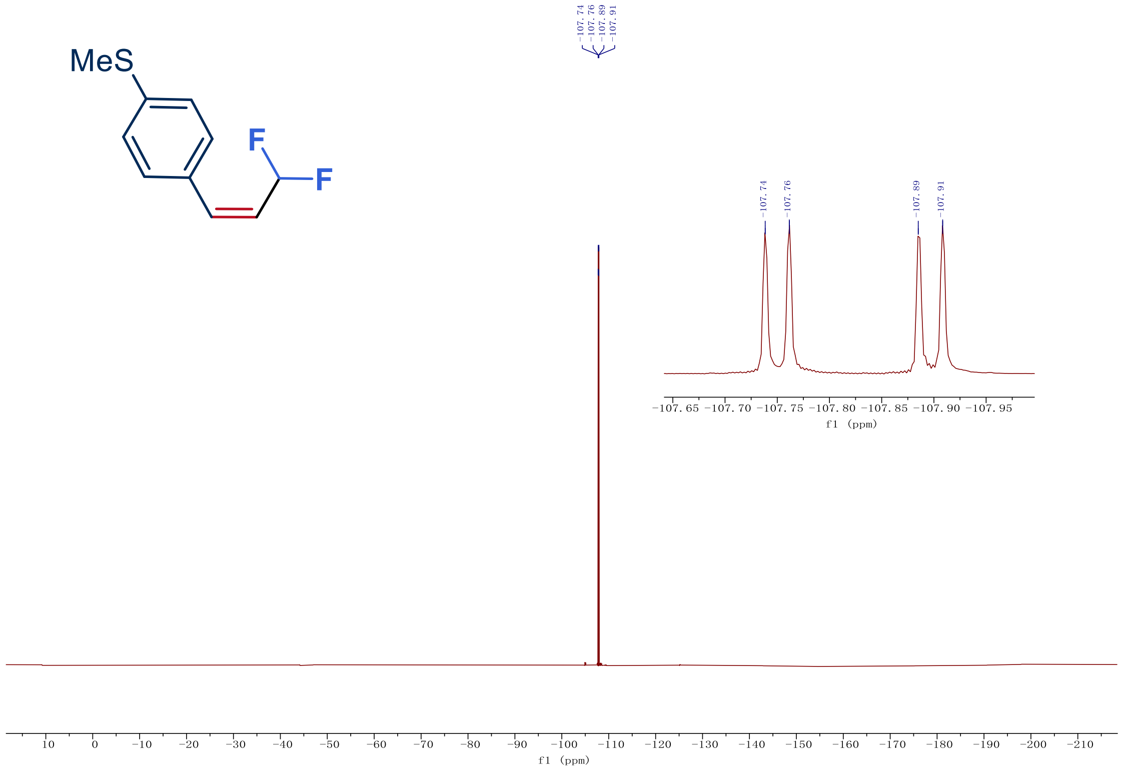

^{13}C NMR (126 MHz, CDCl3) spectra for compound **3n**

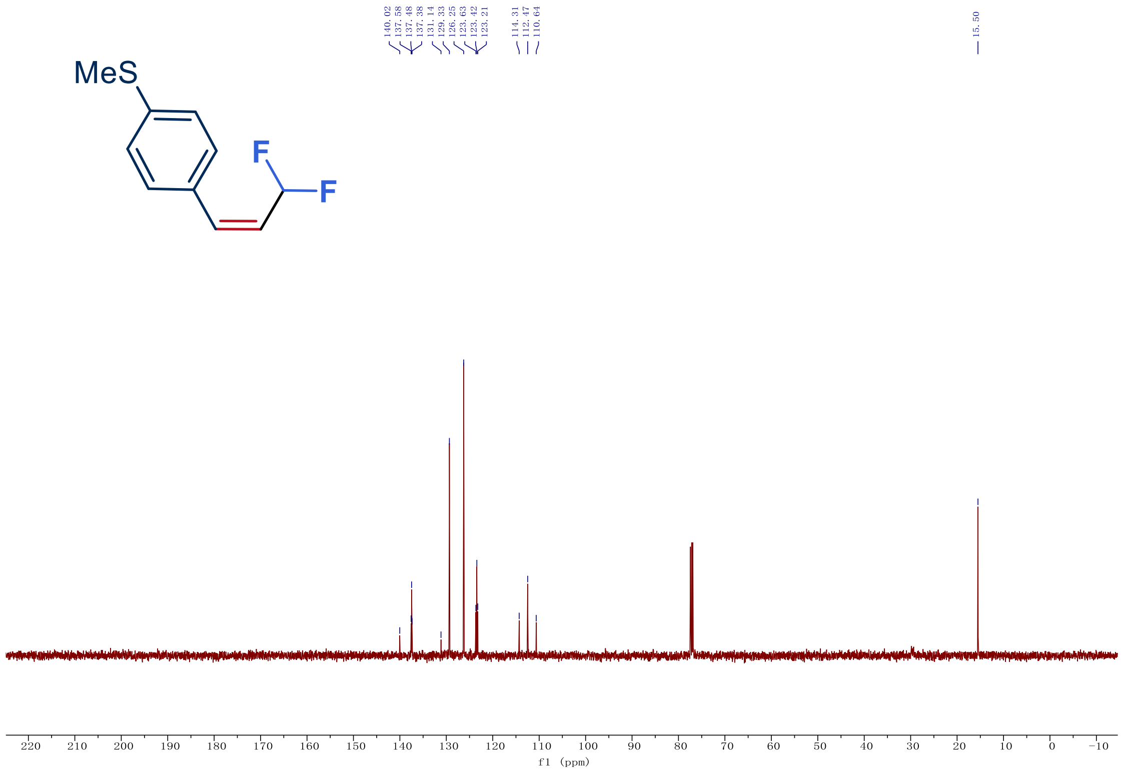

^{1}H NMR (400 MHz, CDCl3) spectra for compound **3o**

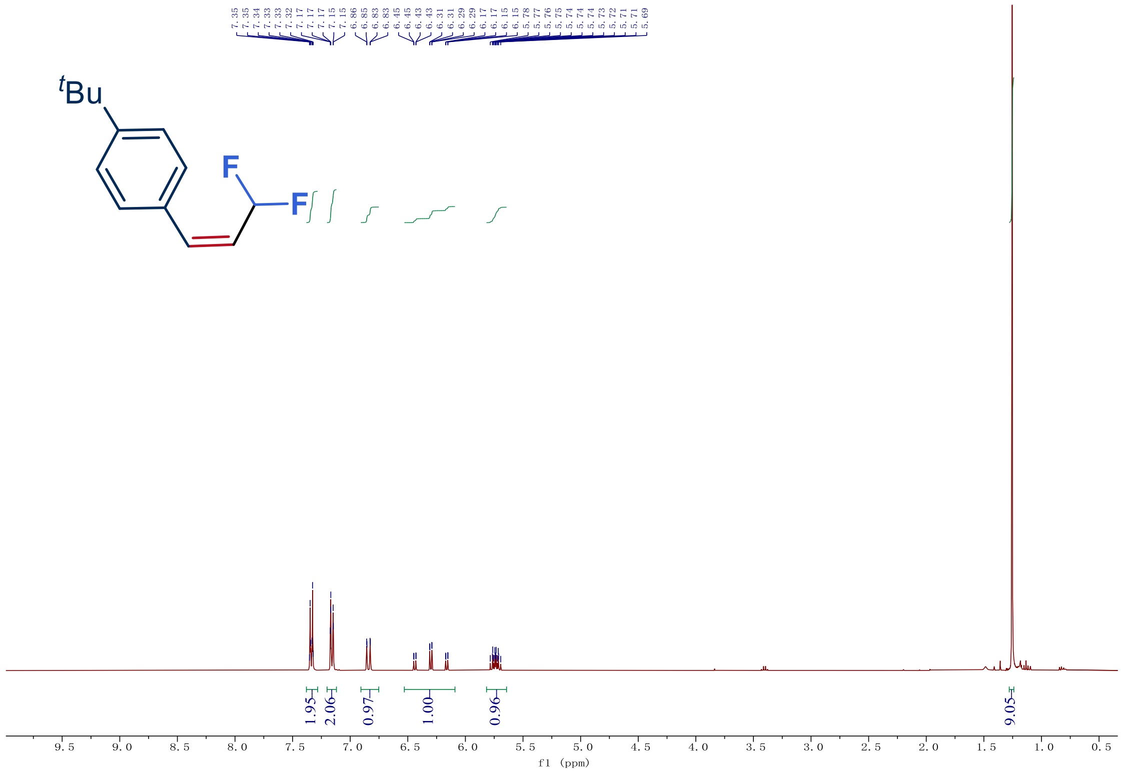

^{19}F NMR (377 MHz, $CDCl_3$) spectra for compound **3o**

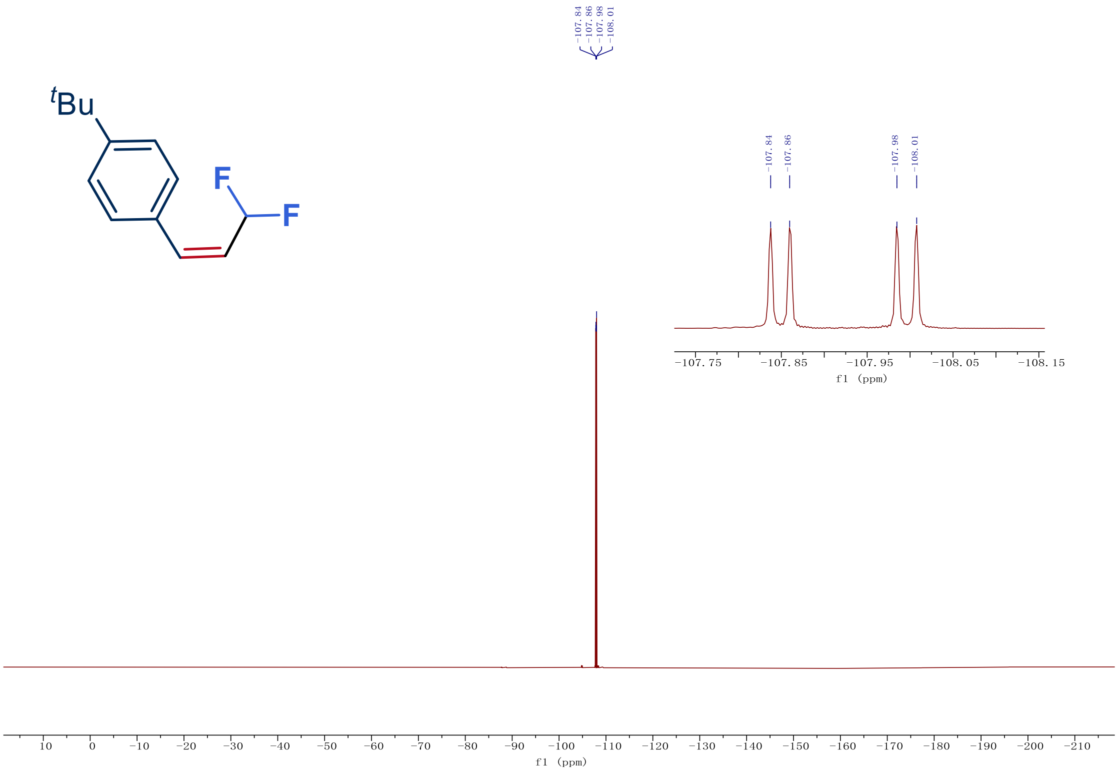

^{13}C NMR (101 MHz, $CDCl_3$) spectra for compound **3o**

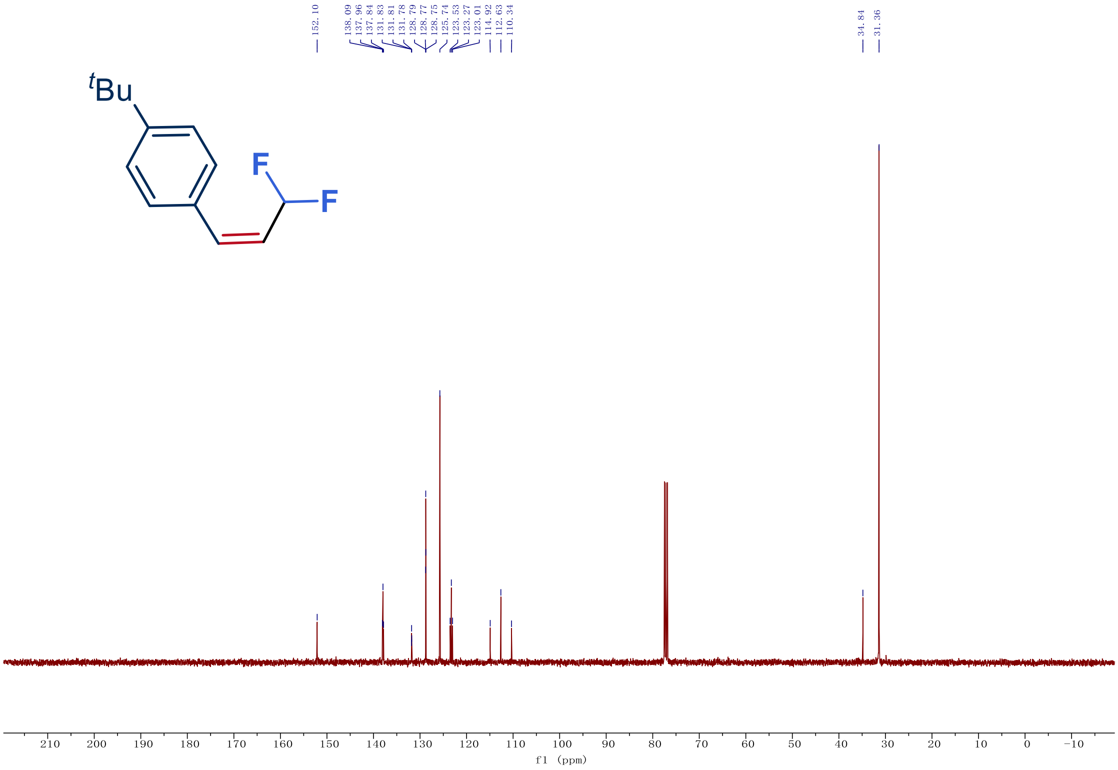

^{1}H NMR (400 MHz, $CDCl_3$) spectra for compound **4**

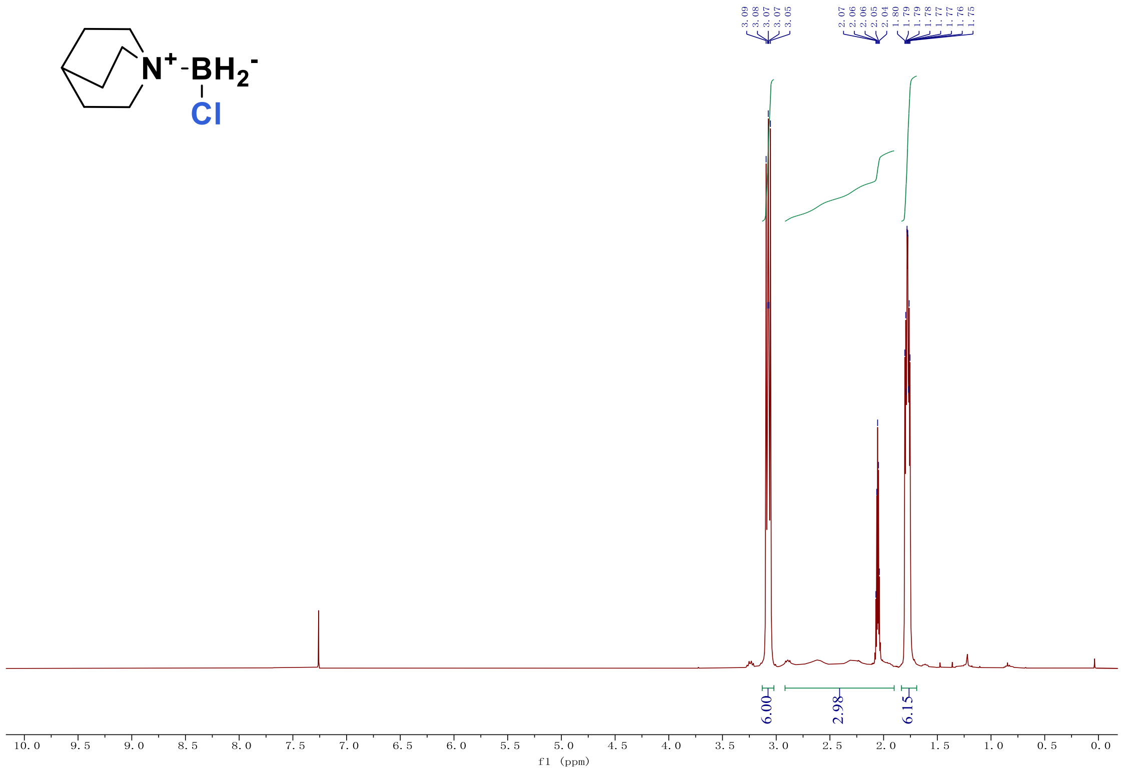

^{11}B NMR (128 MHz, $CDCl_3$) spectra for compound **4**

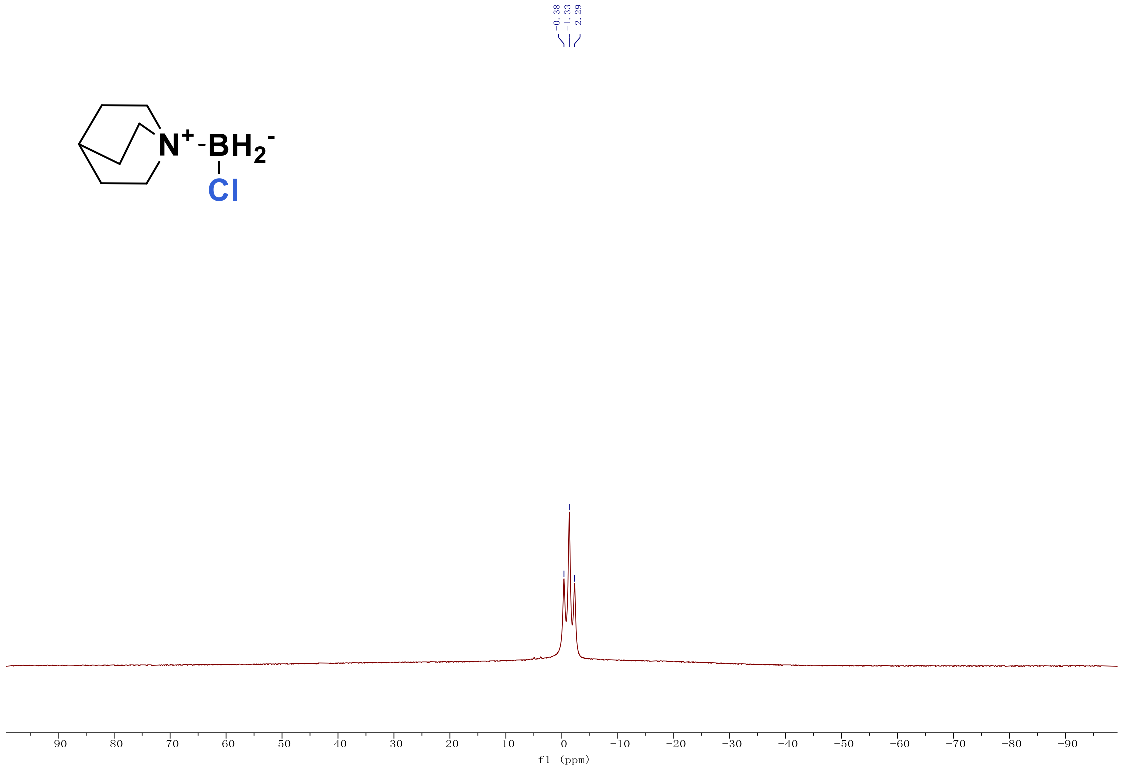

^{13}C NMR (126 MHz, $CDCl_3$) spectra for compound **4**

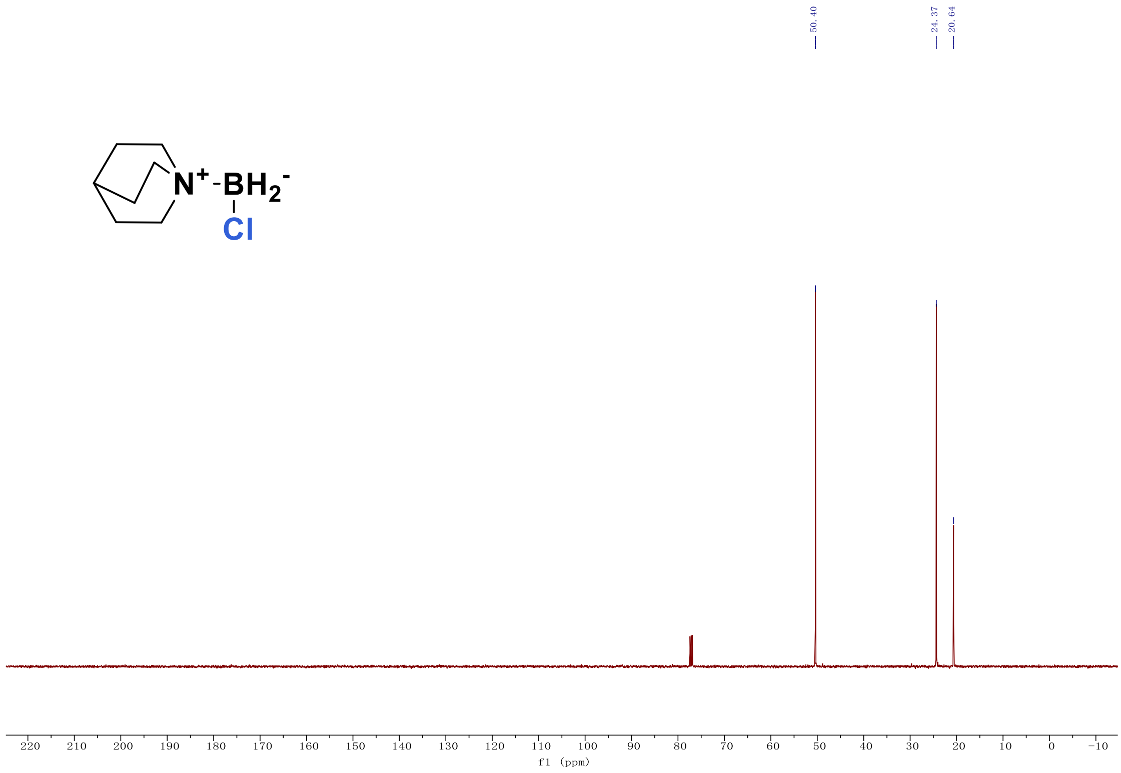

^{1}H NMR (400 MHz, $CDCl_3$) spectra for compound **5**

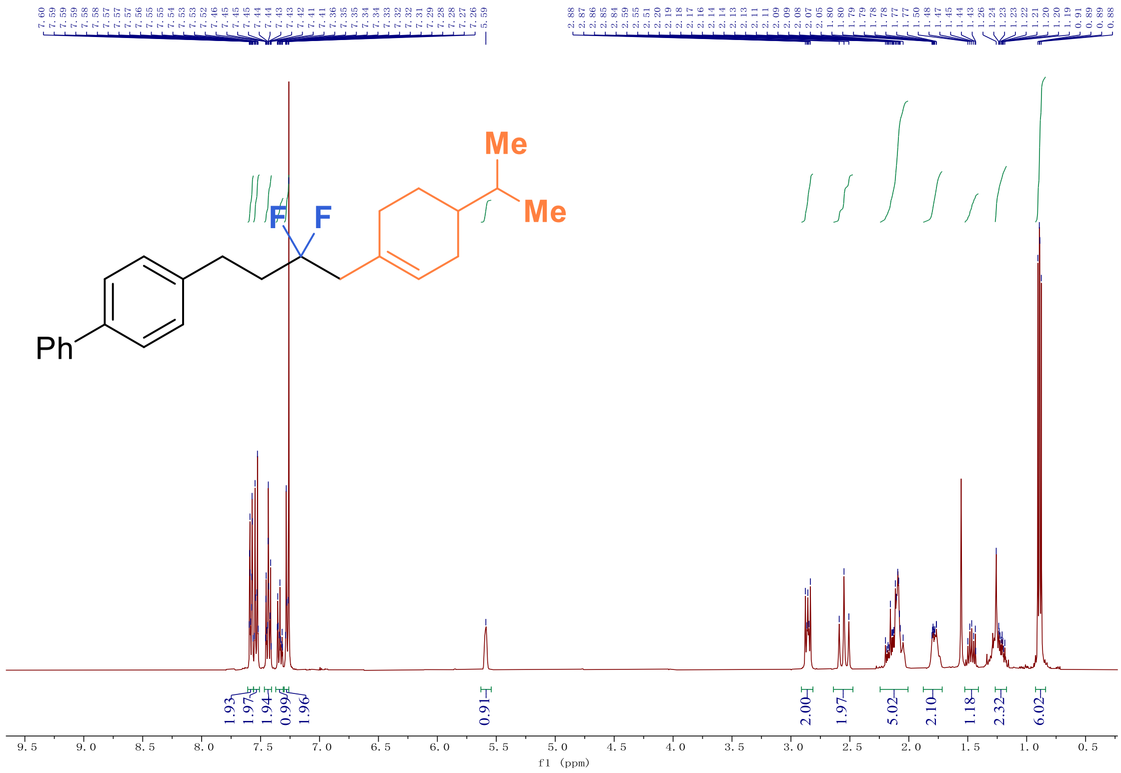

^{19}F NMR (377 MHz, $CDCl_3$) spectra for compound **5**

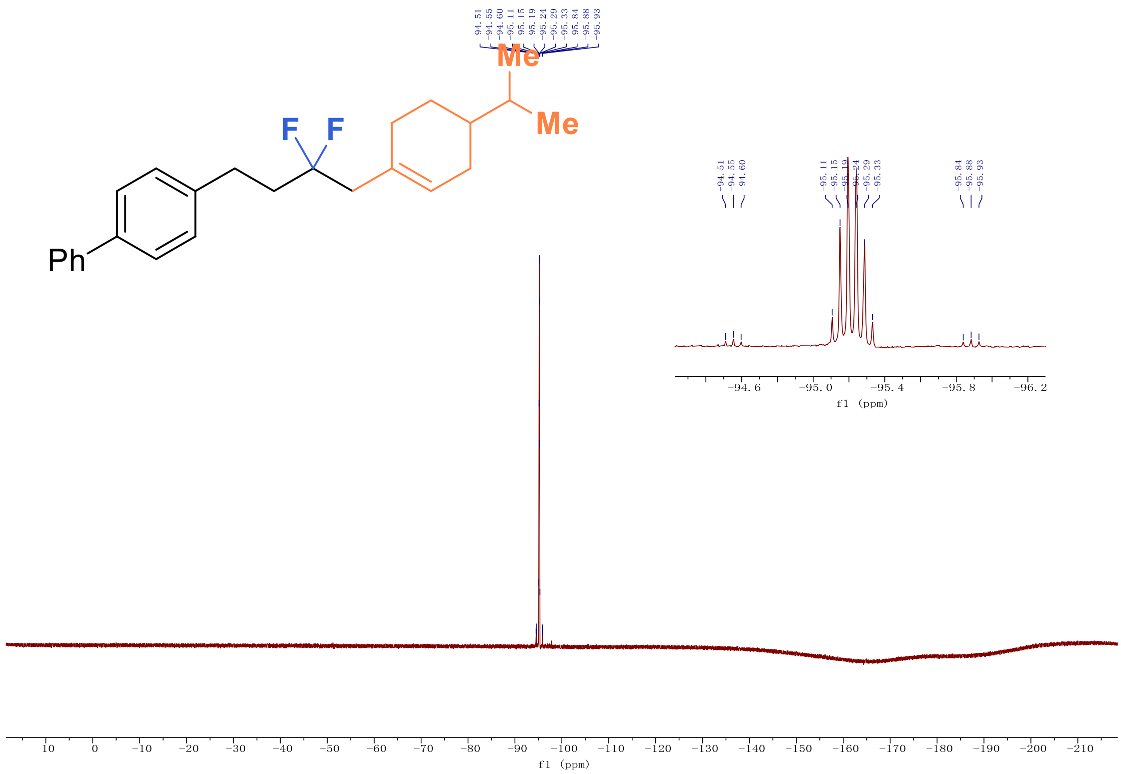

^{13}C NMR (126 MHz, $CDCl_3$) spectra for compound **5**

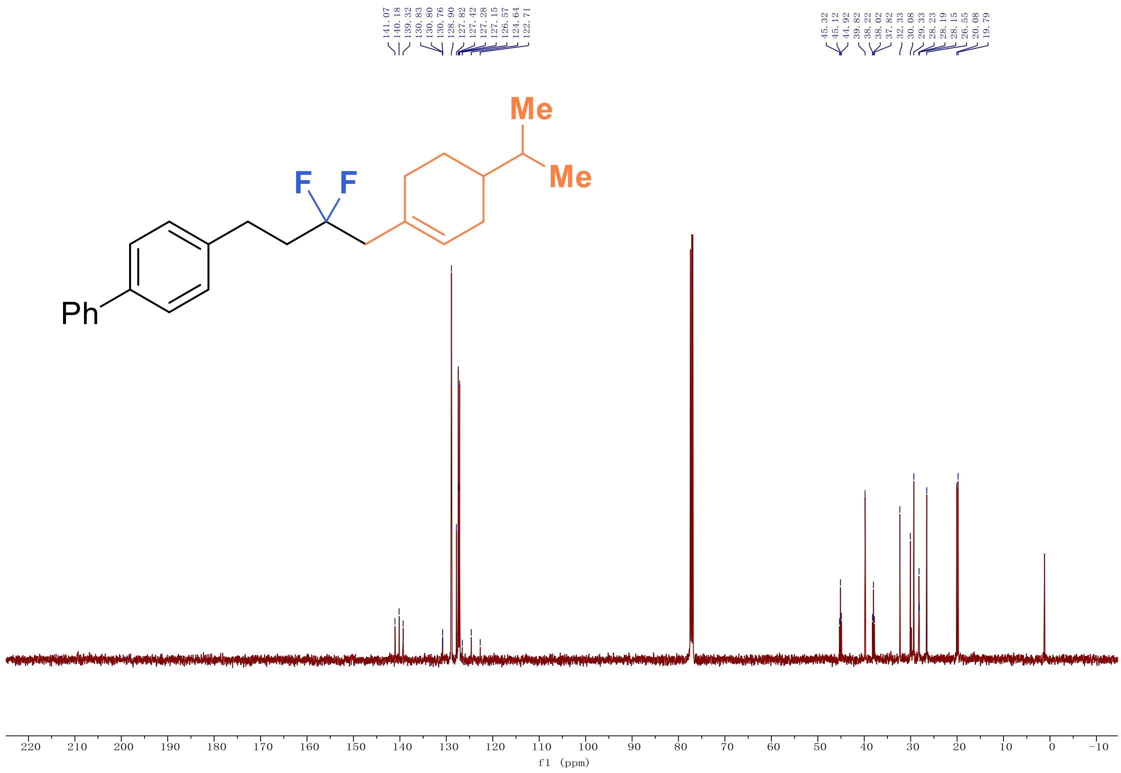

Supplement: SC-014-D3SC03951J-s001 [file SC-014-D3SC03951J-s001.pdf]
